# Ligand Design Enables the Palladium-Catalyzed Intermolecular Carbochlorocarbonylation of Alkynes and Cyclopentenone Formation

**DOI:** 10.1021/jacs.5c01707

**Published:** 2025-07-14

**Authors:** Elliott H. Denton, Hendrik L. Schmitt, Olivera Stepanović, Patrick Müller, Alexander F. Müller, Daniel Svoboda, Bill Morandi

**Affiliations:** Laboratorium für Organische Chemie, ETH Zürich, 8093 Zürich, Switzerland

## Abstract

Herein, we demonstrate that ligand design enables the direct addition of acid chlorides across alkynes in a single step with complete atom economy to afford α,β-unsaturated acid chloride products. This carbochlorocarbonylation reaction, which proceeds through the formal cleavage and reassembly of C–COCl bonds, was developed and explored for a range of acid chlorides and alkynes. During the course of this work, the formation of synthetically useful cyclopentenones through a formal C–H functionalization step was serendipitously observed at elevated temperatures. After optimizing for this divergent reactivity, we explored the substrate scope and undertook experiments to investigate the underlying mechanistic pathway for the formation of this product. Overall, this work expands the carbochlorocarbonylation reaction from activated substrates to alkynes. Further, it provides insight into ligand design for the exploration of fundamental reactivity.

## Graphical abstract

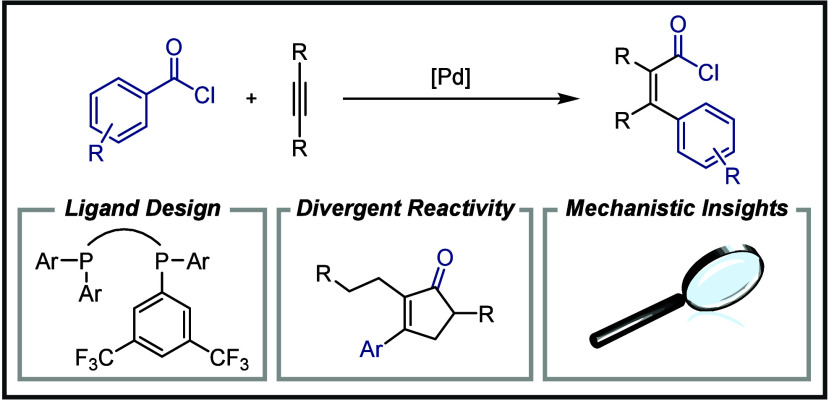

## Introduction

One remarkable feature of transition metal catalysis is the ability to perform functionalization reactions of unsaturated bonds. Hydrofunctionalization reactions, in which a hydrogen and functional group are added across an unsaturated C–C bond, are well-known.^1,2^ By contrast, carbofunctionalization reactions, in which a carbon atom is added instead of a hydrogen atom, tend to be more challenging due to higher kinetic barriers for the insertion of olefins into metal–alkyl bonds as compared to metal-hydride bonds.^3^ This can be attributed to increased steric demand from C–C bond formation, poorer orbital overlap of the more directional M–C bond with the C–C π* orbital, as well as less stabilizing β-agostic interactions during insertion.

In spite of these barriers, there have been several challenging intra- and intermolecular carbofunctionalization reactions developed resulting in the formation of C–C and C–X bonds in a single step.^4–6^ Among these, dicarbofunctionalizations are of particular interest, as they can be leveraged to greatly increase molecular complexity in a single synthetic step.^6–8^ While synthetically powerful, many dicarbofunctionalizations rely on three-component systems, directing groups, or both, leading to stoichiometric waste or a limited range of applicable substrates. By contrast, two-component dicarbofunctionalizations could enable reactions with complete atom economy and preclude waste formation.^9–12^ Realizing such reactions remains a major challenge, as it requires the identification of a catalyst system that is capable of both C–C bond activation and migratory insertion into M–C bonds. As a result, few instances of this type of dicarbofunctionalization reaction have been disclosed, with many examples being restricted to activated or intramolecular systems.^13–23^ Intermolecular examples include the use of strained cyclic systems,^15,23^ or (transient)^24,25^ directing groups,^26^ to enable C–C bond activation. Alternatively, C–CN groups,^27–32^ or C–C­(acyl) bonds in amides^33^ can be added across unsaturated hydrocarbons. In previous work, our group has leveraged a palladium-Xantphos catalyst system to formally cleave the C–COCl bond of acid chlorides and insert it across unfunctionalized norbornene and norbornadiene, leading to a carbochlorocarbonylation reaction (Figure 1).^34^ The latter reactivity was strictly limited to either activated alkenes or intramolecular processes involving substantial substrate engineering.

**Figure 1:**
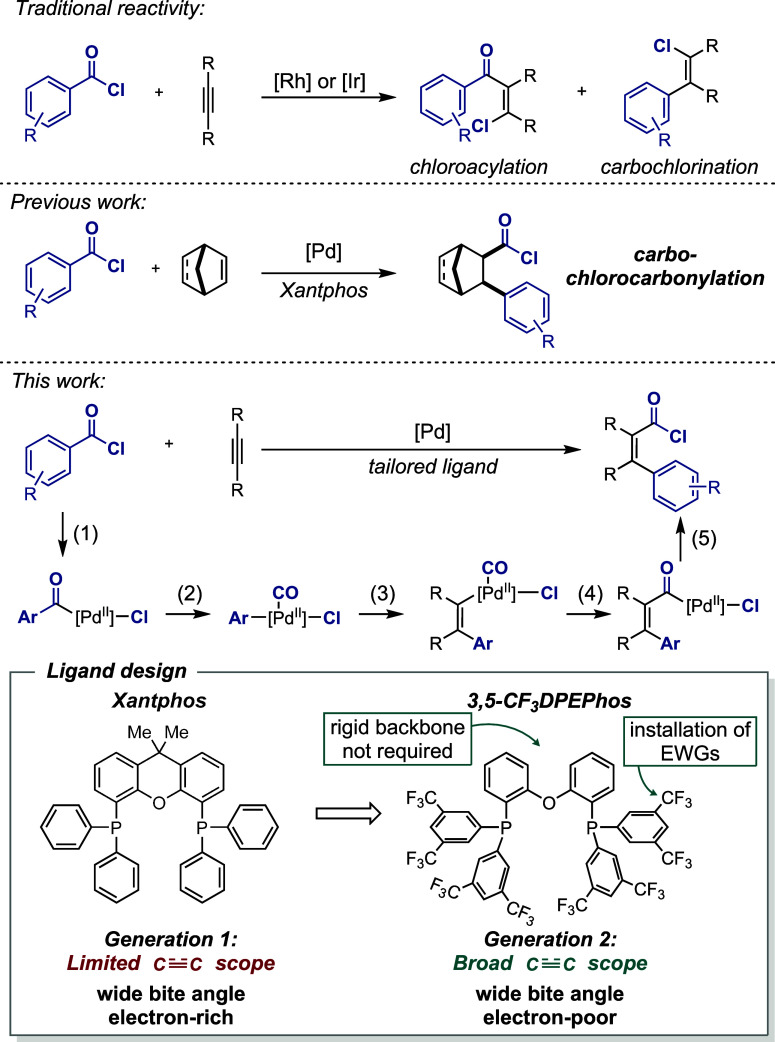
Preliminary considerations to enable the development of a general intermolecular carbochlorocarbonylation reaction via ligand design based on previous work.

Here, we report our investigation of ligand design to achieve the intermolecular carbochlorocarbonylation of alkynes. During our investigation, we serendipitously discovered and explored the formation of cyclopentenones at elevated temperatures. The pathway for the formation of these products was investigated by mechanistic control experiments.

## Results and Discussion

### Ligand Design and Evaluation

We began our investigations by attempting the carbochlorocarbonylation reaction of 5-decyne **2a** with 2,4,6-trimethylbenzoyl chloride **1a** as model substrates under palladium catalysis. Initially, we tested a range of commercially available phosphine ligands, yet the desired reactivity was observed in low amounts (see Supporting Information for the full list). While Xantphos and DPEPhos have previously been shown to disassemble and reconstruct acid chlorides,^34–38^ here only 3–5% yield of the desired product was obtained (as the corresponding methyl ester after quenching with methanol, Table 1, entries 1–2), clearly showing the need for a new approach.

**Table 1:**
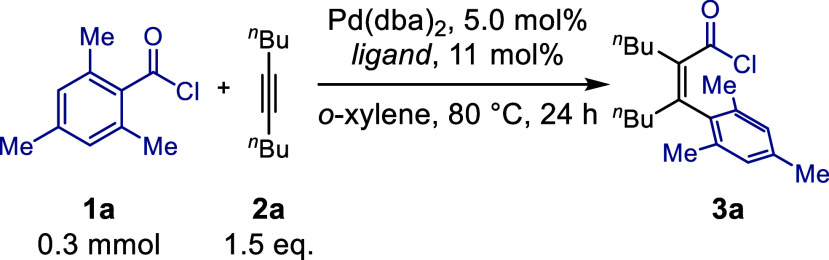

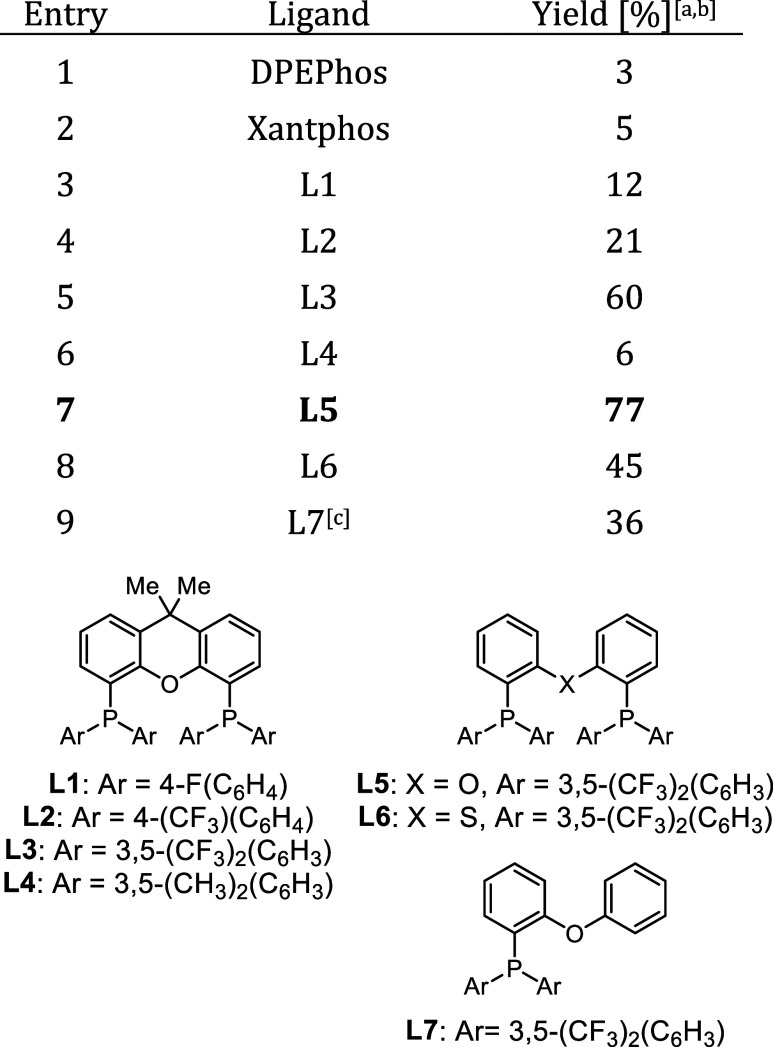
Optimization of the Carbochlorocarbonylation.

a Reactions were performed on a 0.3 mmol scale (0.6 M in *o*-xylene).

b Yields were determined by GC-analysis of the crude reaction mixture using dodecane as internal standard after derivatization to the methyl ester (see SI for more details).

c 22 mol % of ligand L7.

Since the desired intermolecular addition of acid chlorides across alkynes gave very poor yields with commercially available phosphines, we considered tailoring a ligand to the specific needs of this reaction. To achieve this, we initially sought to determine the potential pitfalls in a putative catalytic cycle. From a mechanistic perspective, the reaction is thought to proceed through the following steps: (1) oxidative addition, (2) carbon monoxide deinsertion, (3) migratory insertion, (4) reinsertion of carbon monoxide, and finally, (5) reductive elimination of the C­(acyl)–Cl bond (Figure 1). Orchestrating this sequence of steps is challenging. For example, if step (2) is suppressed, the reaction leads to chloroacylation.^39–42^ Conversely, the chloroarylation product is obtained upon irreversible loss of carbon monoxide (Figure 1).^40–43^

In previous work by our group, acid chlorides have been used in the carboformylation of alkynes using a palladium phosphine catalyst which readily allowed for migratory insertions of C­(aryl)–Pd species across the alkyne (step 3) and reinsertion of carbon monoxide (step 4).^8^ However, the catalyst was incapable of reductively eliminating the C­(acyl)–Cl bond (step 5). In another work on the carbochlorocarbonylation of norbornadiene, both migratory insertion (step 3) and reductive elimination (step 5) were realized.^34^ However, DFT calculations showed that migratory insertion (step 3) has a high energy barrier, with the release of strain energy making a significant contribution to the driving force of this step and the overall reaction. Based on these observations, we anticipated that migratory insertion across the alkyne and reductive elimination to form the acid chloride, steps (3) and (5) respectively, would be the most challenging. We hypothesized that a more electron-poor catalyst would favor both of these elementary steps as these species preferentially undergo migratory insertion and reductive elimination.^3^ To realize this, we considered leveraging the limited reactivity of Xantphos and DPEPhos ligands and placing electron-withdrawing substituents on the phosphine in an effort to enhance their reactivity.

Therefore, we prepared a series of tailored ligand derivatives based on the 2,2′-bis­(phosphino)­diaryl ether motif. Modifications of this scaffold have previously been shown to be suitable to achieve unprecedented reactivities.^44–47^ The introduction of electron-withdrawing groups on the phosphine (4-fluoro **L1** and 4-trifluoromethyl **L2**) resulted in promising improvements, with yields of 12% and 21%, respectively (Table 1, entries 3 and 4). The more electron-poor 3,5-bis­(trifluoromethylphenyl) Xantphos derivative **L3** significantly increased the yield to 60% of the desired product (entry 5).

Arriving at this point in our tailored ligand screening, we turned to structural modification of **L3**, in order to better understand its key features. To confirm that this increase in yield predominately correlated with electronic modifications, we tested 3,5-dimethyl Xantphos **L4**, which gave only a 6% yield (entry 6). Next, we evaluated the 3,5-bis­(trifluoromethyl)­phenyl derivative of DPEPhos **L5**, bearing a more flexible backbone compared with the xanthene backbone. It delivered the highest yield of 77% under standard reaction conditions, highlighting once again the significant role of electron-withdrawing aryl groups (entry 7). Changing the oxygen to sulfur (**L6**) reduced the yield to 45%. Interestingly, the monodentate derivative **L7** yielded 36% of product **3a** (entry 9), and therefore outperformed all other monodentate phosphines and demonstrated that bisphosphine coordination is not strictly necessary. Increasing the reaction temperature above 80 °C improved conversion of the acid chloride but led to the formation of complex mixtures, including various alkene isomers and cyclization products (*vide infra*). To enhance the stereoselectivity of the reaction, we maintained the reaction temperature at 80 °C. In some cases, extending the reaction time proved beneficial, without significant side reactions occurring.

### Carbochlorocarbonylation Reaction and Scope

With the optimized conditions in hand, we set out to determine the scope of both acid chlorides and alkynes in the carbochlorocarbonylation reaction (Figure 2). We began by examining the influence of substituents on the aromatic ring of the acid chlorides. The product from our model reaction **3a** was isolated in 63% yield. Similarly, *o-*tolyl chloride gave a 52% yield of **3b**. When benzoyl chloride was tested, we were able to obtain the product in 9% yield (see Supporting Information for details). Analysis of the crude reaction mixture revealed that limited conversion of the acid chloride occurred, predominantly yielding methyl benzoate upon quenching the reaction.

**Figure 2:**
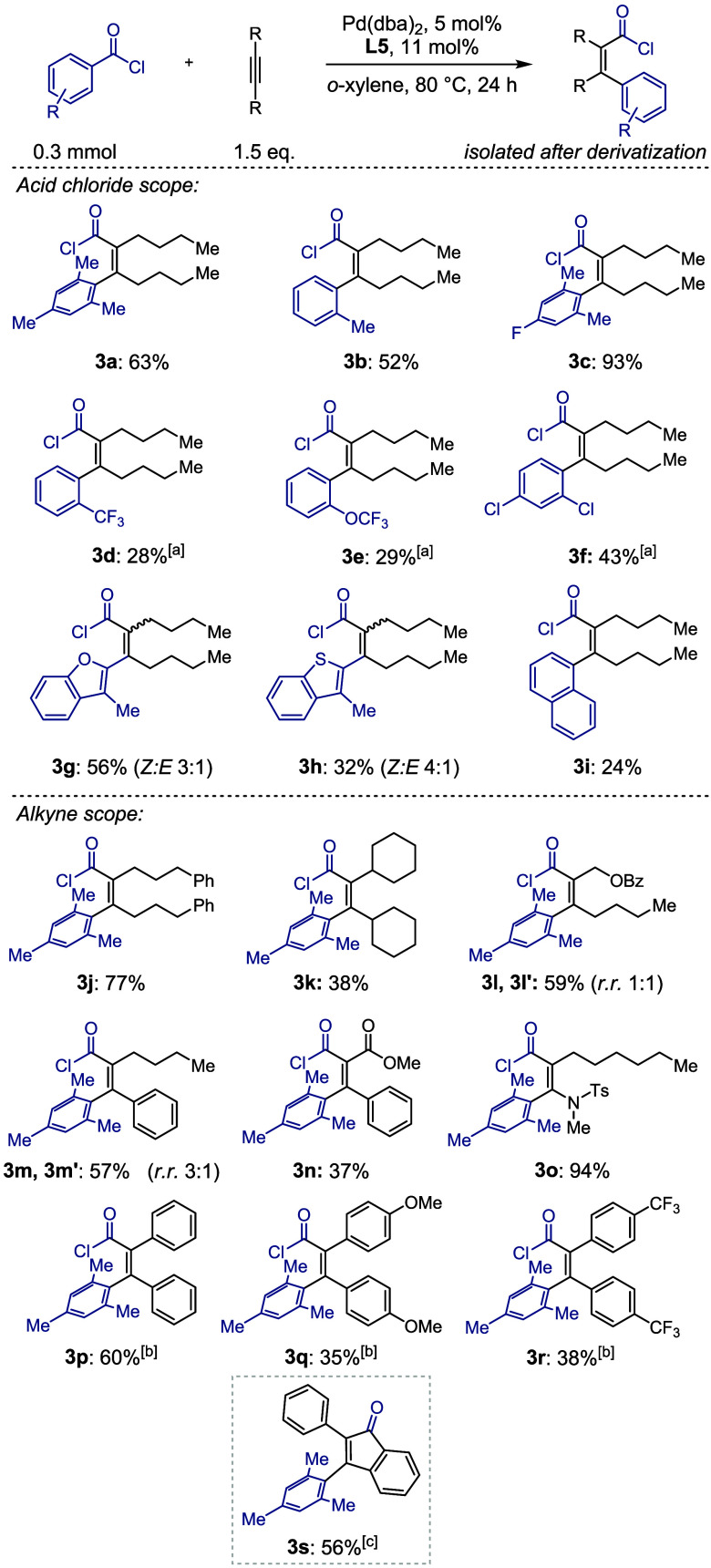
Scope entries for the carbochlorocarbonylation reaction of acid chlorides with alkynes and their isolated yields after derivatization to the methyl ester: ^a^ 80 °C, 72 h; ^b^ 150 °C, 24 h; ^c^ 150 °C, 96 h.

One of the highest yielding examples was observed with 4-fluoro-2,6-dimethylbenzoyl chloride, producing 93% of **3c**. Substrates with more complex electronic profiles (**3d**–**3f**) showed variable reactivity. For example, 2-Trifluoromethylbenzoyl chloride resulted in reduced yields. Its conversion was enhanced by raising the temperature; however, complex mixtures were obtained. Therefore, the highest isolated yield of **3d** remained 28% after 72 h. 2-trifluoromethoxybenzoyl chloride also gave a moderate yield of **3e** in 29%, while 2,4-dichlorobenzoyl chloride provided a higher yield of **3f** in 43% after a prolonged reaction time. Heterocyclic acid chlorides were also evaluated. 3-Methylbenzofuran-2-carbonyl chloride resulted in a yield of 56% (**3g**, *Z*:*E* = 3:1). Similarly, 3-methylbenzo­[*b*]­thiophene-2-carbonyl chloride provided a mixture of *Z* and *E* isomers in a combined yield of 32% (**3h**, *Z*:*E* 4:1), highlighting the tolerance of the method toward heterocycles. For these heterocyclic examples, the isomerization of products was observed, preferentially yielding the *Z*-isomer. The inclusion of extended aromatic systems, such as 1-naphthoyl chloride, resulted in a modest 24% yield of **3i**, confirming that the method is compatible with extended aromatic systems.

The alkyne scope was explored next, showcasing the reaction’s versatility with a range of dialkyl-, diaryl-, and alkylaryl- substituted substrates. Symmetrical dialkyl alkynes reacted smoothly, providing clean products in good yields (63% **3a**, 77% **3j**). Dicyclohexylacetylene also reacted to give 38% of desired product **3k** despite the steric demands of secondary alkyl groups next to the triple bond. Many asymmetrically substituted alkynes produced regioisomeric mixtures, which were separable and fully characterized, with regioselectivity resembling that observed in the Mizoroki–Heck reaction, and our own previous work.^8,48–50^ For example, **3l** yielded 59% with a regioisomeric ratio of 1:1, while other examples showed greater regioselectivity (**3m** 57% 3:1, **3n** 37% exclusively one regioisomer). In addition, ynamide afforded the product **3o** in an excellent yield (94%) with complete regioselectivity. For diaryl alkynes (**3p**–**3s**), elevated temperatures (150 °C, 24 h) were required to achieve acceptable yields. However, side reactions such as Friedel–Crafts acylation became prominent at extended reaction time, particularly in the case of indenone formation (**3s**), which involved double bond isomerization after carbochlorocarbonylation followed by Friedel–Crafts reactivity. This divergent reactivity, similar to Miura’s indenone synthesis,^51^ appeared to originate from the intramolecular reaction of the acid chloride with the phenyl substituent of the alkyne. Additional examples illustrating the broader substrate scope, including less reactive or lower-yielding examples, are also reported (see SI for full list).

### Cyclopentenone Formation

During our optimization of the carbochlorocarbonylation reaction, we observed a new species forming at reaction temperatures above 120 °C. After successful isolation and characterization by NMR and X-ray crystallography, we identified the unexpected product as cyclopentenone **4a** resulting from a formal intramolecular C–H functionalization (Figure 3). Modification of reaction parameters and addition of di-*tert*-butyl pyridine (DTBP) further increased the yield of the cyclopentenone (see Supporting Information for reaction optimization). This novel annulation reaction utilizes easily accessible acid chlorides and alkynes, to directly access cyclopentenones, a key synthetic building block and common motif in natural products and bioactive compounds.^52–57^ Intrigued by this reactivity, we set out to explore the substrate scope of this reaction. We were able to isolate product **4a** from our model reaction, combining 2,4,6-trimethyl benzoyl chloride and 5-decyne in a yield of 68%. Other 2-substituted acid chlorides afforded the corresponding cyclopentenones, ranging from oxo-derived compounds (**4d, 4e**) to chloro-substituents (**4f**). Beyond phenyl-derived substrates, heterocyclic acid chlorides such as thiophene (**4g**), oxazole (**4i**), and pyridine (**4j**) also gave good to moderate yields of the respective cyclopentenones. Benzofuran afforded the product in a 12% yield (**4h**). Additionally, we successfully engaged the acid chloride derivative of the drug febuxostat, yielding the desired cyclopentenone **4k** in a yield of 31%.^58^ Similar to the carbochlorocarbonylation reaction, reduced yields were observed when acid chlorides that do not bear an ortho-substituent were employed in the reaction. In this case, the presence of an electron-withdrawing group seems to enhance the reactivity (**4m** and **4n**). Attempts to increase the yield for these substrates through, e.g., the introduction of a cleavable functionality, were unsuccessful (see Supporting Information for more details).

**Figure 3:**
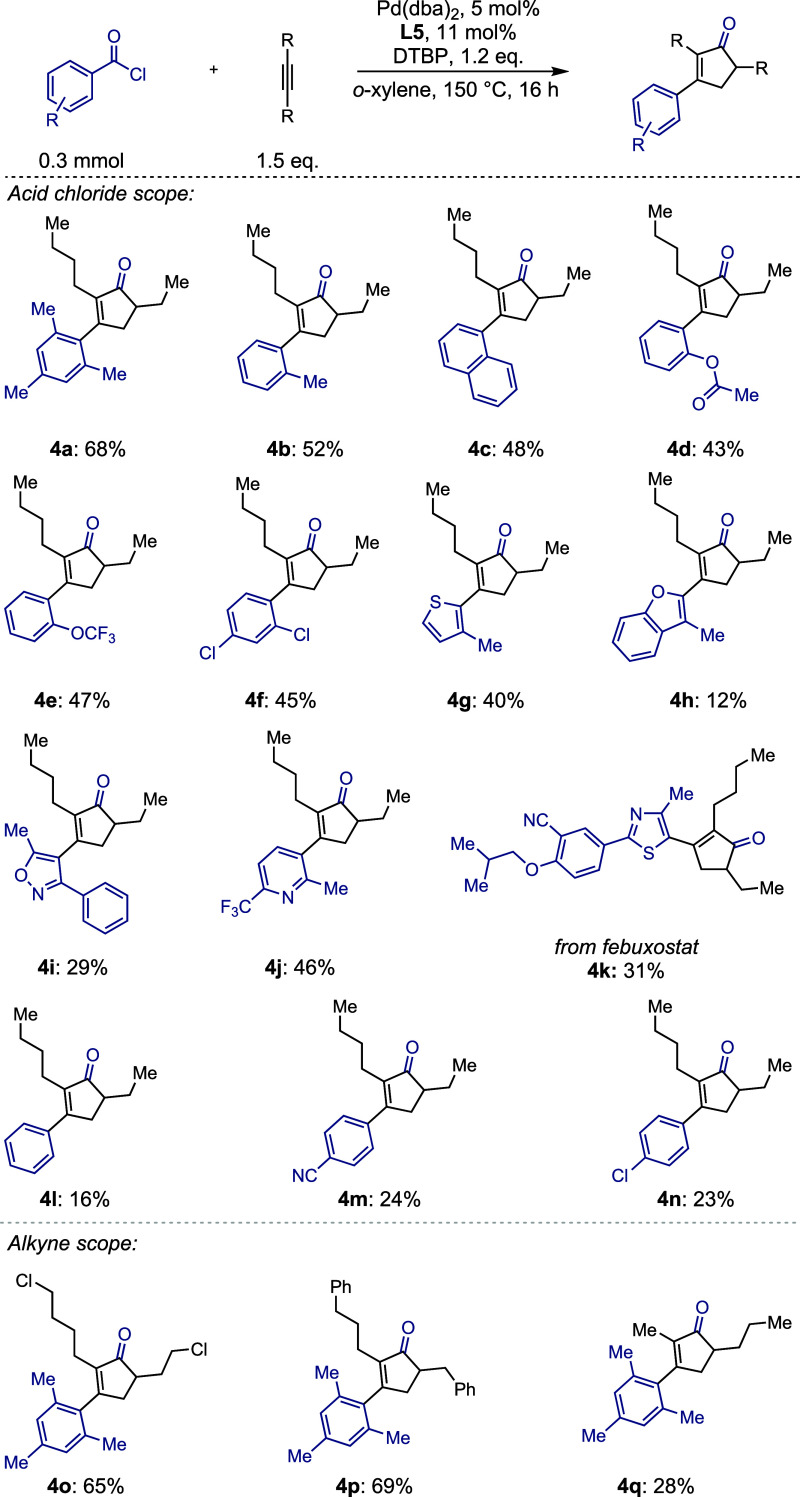
Scope of the cyclopentenone forming reaction from aryl acid chlorides and alkynes.

Other symmetrical alkynes afforded yields comparable to those of 5-decyne (**4o** and **4p**). When an asymmetric alkyne, such as in **4q**, is used, migratory insertion can produce two regioisomers. However, only one of these isomers contains a suitable δ-methylene unit necessary for cyclopentenone ring closure. As a result, in addition to the desired cyclopentenone, various methyl ester byproducts were observed upon quenching the reaction with MeOH. Choosing asymmetric alkynes that do not possess a dimethylene sequence on both sides of the alkyne might, hence, be utilized for the regioselective synthesis of cyclopentenones with varying substituents in the 2- and 5-position.

### Mechanistic Experiments

To elucidate the underlying mechanism of formation of the cyclopentenones, we conducted several control experiments.

During the optimization for carbochlorocarbonylation, we observed that increasing the reaction temperature beyond 80 °C results in the formation of several alkene isomers (Scheme 1, A). The formation of isomer **5** was particularly intriguing, and suggested a path to possibly promote the otherwise challenging functionalization of the δ-methylene position (necessary to access the cyclopentenone product) by virtue of the allylic nature of this position in isomer **5**.

**Scheme 1:**
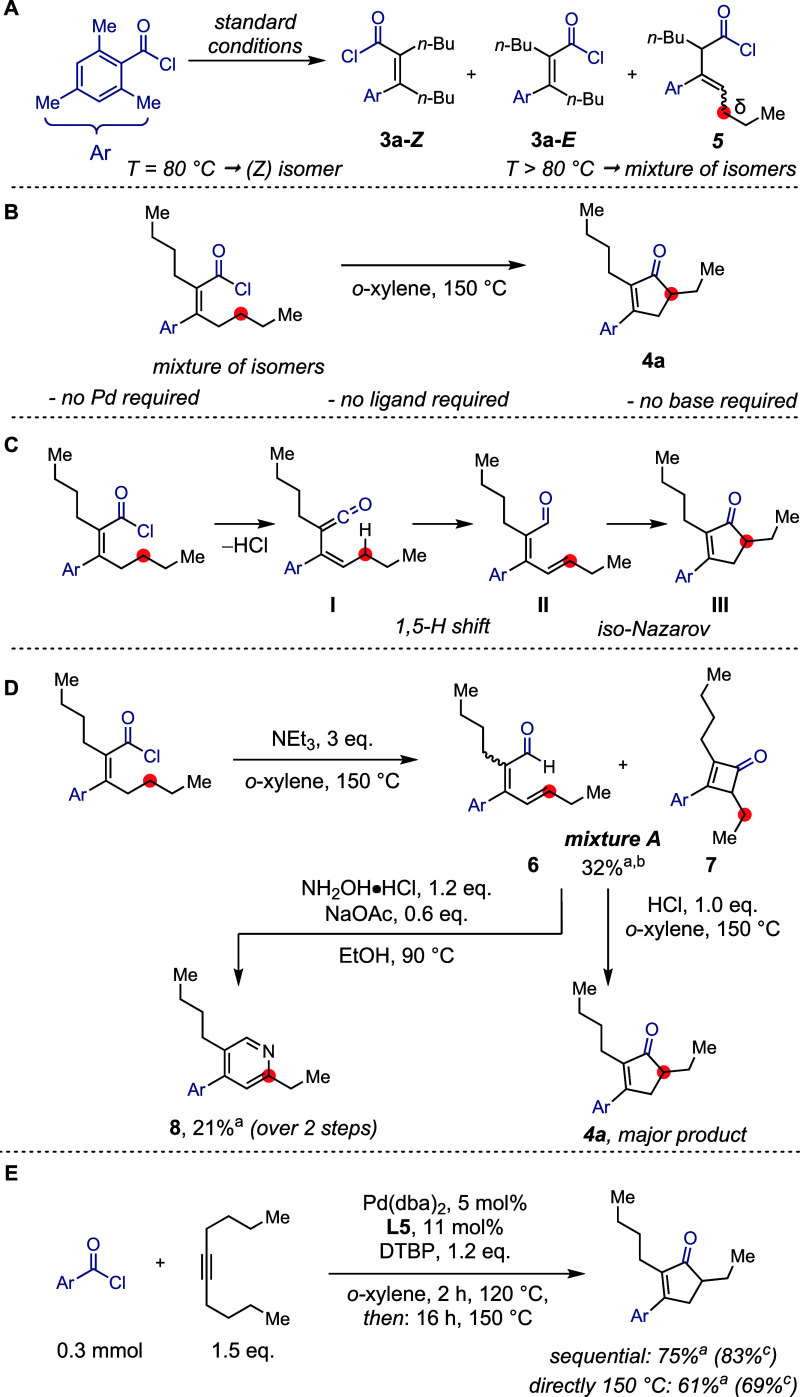
Mechanistic Experiments.

a Isolated yield.
b Isolated 6:7 in a 1:1.4 ratio.
c NMR yield, with 1,3,5-trimethoxybenzene as internal standard.

Subsequently, we wanted to confirm that the cyclopentenone was formed from the α,β-unsaturated acid chloride **3a**, as opposed to a pathway independent of carbochlorocarbonylation. When **3a** was heated to 150 °C in the absence of palladium,**L5**, and DTBP, cyclopentenone **4a** was observed (Scheme 1, B). Given these results, we considered whether the cyclopentenone might arise from initial elimination of hydrochloric acid from acid chloride **3a** leading to an intermediate ketene **I** (Scheme 1, C).^59–62^ Such an elimination mechanism might also be consistent with the observed formation of isomer **5** through reprotonation of this putative intermediate.^63^ Following a 1,5-hydride shift, dienal **II** could then be formed from the ketene,^64–67^ which would then be prone to undergo an acid-catalyzed iso-Nazarov cyclization to result in the formation of the cyclopentenone **III**.^68–73^ To test our hypothesis, we heated acid chloride **3a** at 150 °C in the presence of triethylamine to scavenge any hydrochloric acid in an effort to interrupt the reaction to determine whether an aldehyde could be observed (Scheme 1, D). Triethylamine was chosen as opposed to the frequently employed DTBP due to its considerably increased basicity.^74,75^ Under these conditions, we could isolate mixture **A**, which is constituted of aldehyde **6** and the coeluting cyclobutenone **7**. The latter product is strongly suggestive of ketene intermediate **I** being generated under the reaction conditions. When mixture **A** was independently subjected to anhydrous hydrochloric acid in *o*-xylene at 150 °C for 2 h, the *iso*-Nazarov reactivity was regenerated and cyclopentenone **4a** was formed as confirmed by NMR analysis of the crude mixture.

Intrigued by the potential of the underlying synthetic sequence, we hypothesized that diverging from the obtained dienal **6** could enable access to 2,4,5-trisubstituted pyridines. Indeed, subjecting acid chloride **3a** to 150 °C in the presence of triethylamine, followed by solvent exchange and condensation with hydroxylamine hydrochloride,^66,76^ allowed us to prepare pyridine **8** with an unusual substitution pattern.

As the formation of the cyclopentenone is independent of the palladium catalyst, we questioned whether it might be possible to use this insight to further optimize the reaction. The standard conditions of cyclopentenone formation resulted in incomplete conversion of the acid chloride starting material, even in cases where full consumption of the latter was observed in the carbochlorocarbonylation reaction. We concluded that at elevated temperatures, catalyst decomposition might impede the overall efficiency of the reaction. Therefore, a reaction was performed at 120 °C to achieve higher conversion of the acid chloride in the carbochlorocarbonylation step, followed by 150 °C to access the cyclopentenone **3a**. This reaction sequence enabled us to moderately increase the yield for our model substrate from 61% (69% NMR yield) to 75% (83% NMR yield) (Scheme 1, E).

## Conclusion

Through ligand design, we successfully realized the addition of acid chlorides across alkynes via formal cleavage of the C–COCl bond in a single step with complete atom economy. The resulting tetrasubstituted alkene products retain the acid chloride functionality from the starting material, a versatile functional handle that could be transformed into an array of valuable α,β-unsaturated carbonyl compounds.

During our investigation, we discovered that elevated temperatures resulted in the unexpected formation of trisubstituted cyclopentenones. This reaction was further optimized to result in a single step synthesis of these complex motifs using a variety of acid chlorides and alkynes. To gain an understanding of how these cyclopentenone products were formed, we conducted a series of mechanistic experiments. From this, we propose a mechanism that begins with the palladium-catalyzed carbochlorocarbonylation. Subsequently, through elimination of hydrochloric acid, a ketene is formed, which then undergoes a 1,5-H shift. Finally, an iso-Nazarov reaction completes the formation of the cyclopentenone. This represents a rare example of the use of widely available reagents in a cascade fashion to obtain these products in a single step.

## Supplementary Material



## Data Availability

Compound characterization data can be accessed under 10.5281/zenodo.15630820.

## Notes

Additional optimization, characterization and mechanistic data, as well as full synthetic (PDF)
