# Supplementary material for: Ligand Design Enables the Palladium-Catalyzed Intermolecular Carbochlorocarbonylation of Alkynes and Cyclopentenone Formation
Source: J Am Chem Soc. 2025 Jul 14;147(29):25215–23. doi: 10.1021/jacs.5c01707 (PMC12291460; doi:10.1021/jacs.5c01707)
Supplement: Supplementary file 1 [file ja5c01707_si_001.pdf]

Ligand Design Enables the Palladium-Catalyzed Intermolecular Carbochlorocarbonylation of Alkynes and Cyclopentenone Formation

Elliott H. Denton,[‡] Hendrik L. Schmitt,[‡] Olivera Stepanović,[‡] Patrick Müller, Alexander F. Müller,[#] Daniel Svoboda,[#] and Bill Morandi*

bill.morandi@org.chem.ethz.ch

Laboratorium für Organische Chemie, ETH Zürich, 8093 Zürich, Switzerland

Table of Contents

1. General Information....................1
2. Ligand Synthesis....................2
3. Optimization....................12
4. Extended Scope....................36
5. Carbochlorocarbonylation....................39

General procedure A1....................39

6. Cyclopentanone formation....................57

General procedure B1....................57

7. Attempts to increase yield for non-ortho substituted acid chlorides....................70
8. Starting material synthesis....................71
9. Mechanistic investigations....................72
10. X-ray Crystal Structures....................79

General....................79

11. References....................87
12. NMR spectra....................89

Ligand synthesis....................89

Carbochlorocarbonylation....................117

Cyclopentenones....................205

Mechanistic investigations....................226

General Information

Unless otherwise noted, all reactions were carried out under argon in oven-dried 4 mL screw-cap glass vials using anhydrous solvents. The anhydrous solvents were prepared by distillation over appropriate drying agents or by using a solvent purification system (LC Technology Solutions, Inc.) under N_2 atmosphere (H_2O content: below 10 ppm, as determined by Karl Fischer titration) and stored over molecular sieves prior to use. All commercially available compounds were used as received from common suppliers (Sigma-Aldrich, Strem Chemicals, abcr, TCI, Fluorochem, Acros Organics, Alfa Aesar and Apollo Scientific).

Thin layer chromatography (TLC): Aluminum TLC plate, silica gel coated with fluorescent indicator F254 (TLC Silica gel 60 F254, Merck). Visualization was accomplished using UV light (254 nm) or $KMnO_4$ stain.

Flash column chromatography: SiliaFlash P60 silica gel (60 Å, 40−63 µm, SiliCycle Inc.) with reagent grade solvents.

NMR: Spectra were recorded on Bruker AVANCE III 400, Neo 400, Neo 500, or 600 spectrometers at room temperature, unless indicated otherwise; the chemical shifts are reported with respect to internal solvent: δH = 7.26 ppm, and δC = 77.16 (t) ppm ($CDCl_3$); δH = 7.16 ppm, and δC = 128.06 (t) ppm (C_6D_6); δH = 2.50 (p) ppm, and δC = 39.52 (hept) ppm (DMSO-d_6). Multiplicities are indicated by s (singlet), d (doublet), t (triplet), q (quartet), p (quintet), h (sextet), hept (septet), m (multiplet), br (broad), or combinations thereof. 2D spectra are always listed in the sequence: COSY, NOESY, HSQC, HMBC without exception.

GC/FID and GC/MS: Shimadzu GC-2025 (capillary column: Macherey-Nagel OPTIMA 5, 30.0 m × 0.25 mm × 0.25 µm; carrier gas: H_2); To determine GC yields, calibration curves were generated using *n*-dodecane as an internal standard. Shimadzu GCMS-QP2020 (capillary column: Macherey-Nagel OPTIMA 5, 30.0 m × 0.25 mm × 0.25 µm; carrier gas: He).

High-resolution MS (HRMS): Thermo scientific Q-Exactive GC Orbitrap for EI. Bruker Daltonics maXis ESI-QTOF or solariX ESI-FTICR-MS for ESI. HRMS data were obtained by the mass spectrometry service (MoBiAS, Molecular and Biomolecular Analysis Service) in the Laboratorium für Organische Chemie at ETH Zürich.

Ligand Synthesis

Discussion

The synthesis of the xantphos-based ligands (**L01-L04, L06**) proceeded smoothly following a literature procedure involving lithiation of the xanthene backbone, followed by trapping the desired diarylchlorophosphine.[1] If the diarylchlorophosphine was not commercially available, it was prepared according to a reported procedure.[2] This method enabled the preparation of sufficient amounts of ligand for screening. While this procedure afforded sufficient quantities of ligand **L05** for screening, we found that attempts to increase the scale of this reaction were met with diminishing returns. Investigating this, we found that gram-scale reactions produced the desired ligand **L05** along with **L07**, phosphazole **SI02**, and triarylphosphine (bis-3,5-$CF_3Ph)_3P$. Further, we found that the reaction varied depending on the quality of the ^{s}BuLi, solvents, and phosphine chloride.

In order to produce sufficient quantities of **L05**, a protocol based on another literature procedure was developed.[3,4] Lithiation of diphenyl ether followed by trapping with $(Et_2N)_2PCl$ resulted in a phosphoramide, which was converted to dichlorophosphine. Treating this intermediate with the corresponding aryl Grignard reagent afforded **L05**.

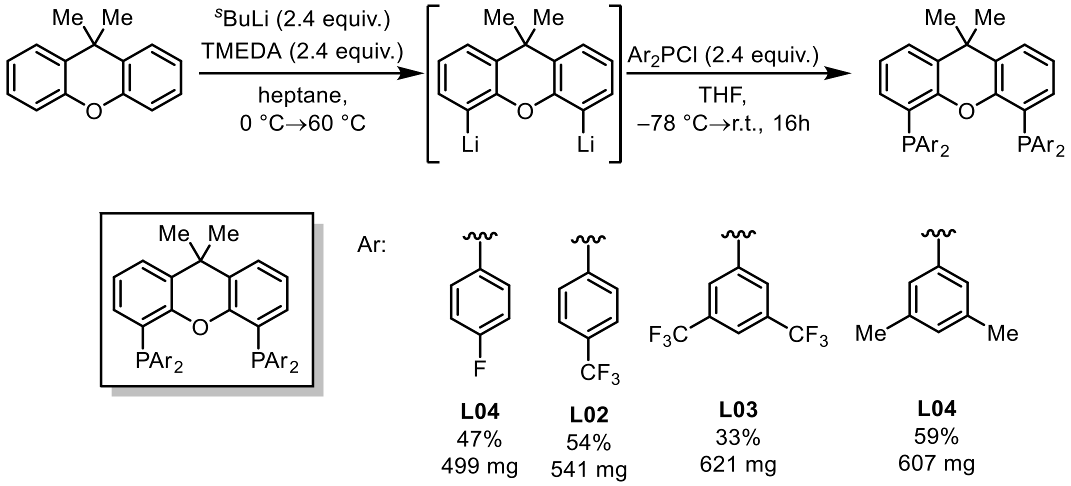

Figure S1: Synthetic protocol and yields for ligands **L01** - L**04**

(9,9-dimethyl-9*H*-xanthene-4,5-diyl)bis(4-fluorophenyl)phosphane) L01

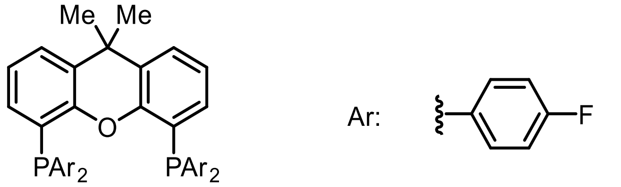

The product was obtained as a white solid (47%, 499 mg).

^{1}H NMR (400 MHz, $CDCl_3$) δ 7.43 (dd, J = 7.8, 1.6 Hz, 2H), 7.12 – 7.06 (m, 8H), 6.98 (t, J = 7.7 Hz, 2H), 6.92 (tt, J = 8.7, 2.4 Hz, 8H), 6.46 (dq, J = 7.5, 1.7 Hz, 2H), 1.65 (s, 6H) ppm.

^{13}C{^{1}H} (100 MHz, $CDCl_3$) δ 163.4 (d, J = 248.4 Hz), 152.4 (dd, J = 11.2, 8.6 Hz), 136.3 – 135.1 (m), 132.6 (t, J = 8.0 Hz), 131.8, 130.2, 126.8, 125.5 (dd, J = 10.9, 7.8 Hz), 123.8, 115.6 (dt, J = 20.9, 3.8 Hz), 34.6, 31.9 ppm.

^{19}F{^{1}H} (376.5 MHz, $CDCl_3$) δ –113.01 (t, J = 2.3 Hz) ppm.

^{31}P{^{1}H} (162 MHz, $CDCl_3$) δ –19.25 (p, J = 2.3 Hz) ppm.

The characterization data match the literature.[5]

(9,9-dimethyl-9*H*-xanthene-4,5-diyl)bis(4-(trifluoromethyl)phenyl)phosphane) L02

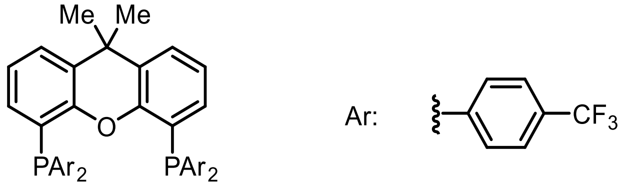

The product was obtained as a white solid (54%, 541 mg).

^{1}H NMR (400 MHz, $CDCl_3$) δ 7.58 – 7.51 (m, 10H), 7.32 – 7.28 (m, 8H), 7.08 (t, J = 7.7 Hz, 2H), 6.54 (dq, J = 7.6, 1.8 Hz, 2H), 1.74 (s, 6H) ppm.

^{13}C{^{1}H} (100 MHz, $CDCl_3$) δ 152.6 (t, J = 9.7 Hz), 141.5 (t, J = 7.8 Hz), 134.2 (dd, J = 12.5, 8.9 Hz), 132.0, 131.0 (q, J = 32.5 Hz), 130.6 (t, J = 1.1 Hz), 127.5, 125.7 – 125.4 (m), 125.3, (p, J = 3.7 Hz), 124.2 (q, J = 272.3 Hz), 123.7 – 123.4 (m), 34.8, 31.9 ppm.

^{19}F{^{1}H} (376.5 MHz, $CDCl_3$) δ – 62.78 ppm.

^{31}P{^{1}H} (162 MHz, $CDCl_3$) δ –17.5 ppm.

TLC: 0.48 (SiO_2, 90:10 hexane:CH_2Cl_2)

The characterization data match the literature.[5]

(9,9-dimethyl-9*H*-xanthene-4,5-diyl)bis(3,5-bis(trifluoromethyl)phenyl)phosphane) L03

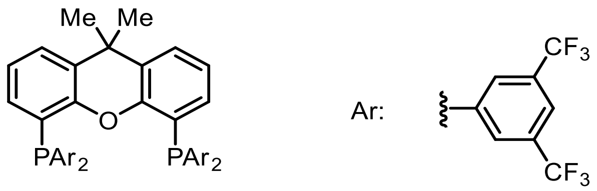

The product was obtained as a white solid (33%, 621 mg).

^{1}H NMR (400 MHz, $CDCl_3$) δ 7.88 (br s, 4H), 7.62 – 7.60 (m, 8H), 7.57 (dd, *J* = 7.9, 1.5 Hz, 2H), 7.13 (t, *J* = 7.7 Hz, 2H), 6.41 – 6.38 (m, 2H), 1.67 (s, 6H) ppm.

^{13}C{^{1}H} (100 MHz, $CDCl_3$) δ 152.2, 138.9 (dd, *J* = 10.9, 8.0 Hz), 133.6, 132.4 (qt, *J* = 33.4, 3.0 Hz), 131.3, 131.2, 128.4, 125.2, 123.6, 123.1 (q, *J* = 273.1 Hz), 121.3, 34.9, 31.2.

^{19}F{^{1}H} (376.5 MHz, $CDCl_3$) δ – 63.11 ppm.

^{31}P{^{1}H} (162 MHz, $CDCl_3$) δ –14.5 ppm.

TLC: R_f = 0.21 (SiO_2, 95:5 hexane:CH_2Cl_2)

The characterization data match the literature.[1]

(9,9-dimethyl-9*H*-xanthene-4,5-diyl)bis(bis(3,5-dimethylphenyl)phosphane) L04

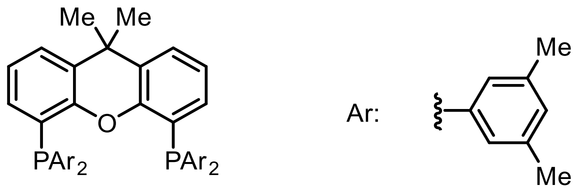

The product was obtained as a white solid (59%, 607 mg).

^{1}H NMR (400 MHz, $CDCl_3$) δ 7.39 (dd, *J* = 7.7, 1.6 Hz, 2H), 6.97 (t, *J* = 7.6 Hz, 2H), 6.85 (br s, 4H), 6.81 – 6.79 (m, 8H), 6.56 (dq, *J* = 7.5, 1.6 Hz, 2H), 2.19 (s, 24H), 1.65 (s, 6H) ppm.

$^{13}C\{^1H\}$ (100 MHz, $CDCl_3$) δ 153.2 (t, *J* = 9.8 Hz), 137.4 (dd, *J* = 6.9, 5.8 Hz), 137.2 (t, *J* = 3.6 Hz), 132.1, 131.7 (t, *J* = 10.5 Hz), 130.3, 130.0, 126.7 (dd, *J* = 11.6, 9.0 Hz), 125.7, 123.4, 34.7, 31.1, 21.5 ppm.

$^{31}P\{^1H\}$ (162 MHz, $CDCl_3$) δ –17.4 ppm.

TLC: 0.56 (SiO_2, 90:10 hexane:CH_2Cl_2)

The characterization data match the literature.[6]

(Oxydi-2,1-phenylene)bis(dichlorophosphine) (SI01)

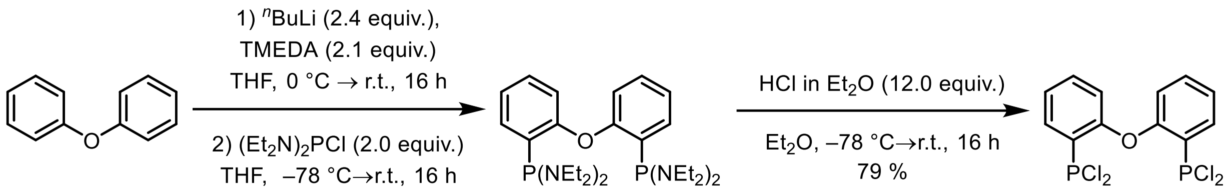

According to reported literature procedures,[3,4] under an atmosphere of dry nitrogen, TMEDA (7.5 mL, 50 mmol, 2.1 equiv.) was added dropwise to a flask containing ^{n}BuLi (1.6 M in hexane, 35.0 mL, 56 mmol, 2.4 equiv.) at room temperature. The obtained cloudy mixture was cooled down by an ice bath and a solution of diphenylether (4.008 g, 23.5 mmol, 1.0 equiv.) in THF (15 mL) was added dropwise. After the addition, the reaction mixture was warmed up to room temperature and stirred overnight. The obtained yellow slurry was cooled down to – 78 °C by a dry ice - acetone bath and a solution of bis(diethylamino)chlorophosphine (10.1 mL, 48 mmol, 2.0 equiv.) in THF (15 mL) was added over the course of 30 min. The reaction mixture was allowed to warm up to room temperature overnight before it was concentrated under reduced pressure. The formed red-brown oil was redissolved in toluene (20 mL) and filtered through a Celite plug. The filter residue was washed with toluene (2 x 10 mL) and the combined filtrate was concentrated under reduced pressure to obtain 1,1'-(oxybis(2,1-phenylene))bis(*N,N,N',N'*-tetraethylphosphanediamine) [$\delta_{^{31}P}$= 91.5 ppm] as a red-brown oil that was directly used in the next step.

The phosphanediamine was dissolved in diethylether (150 mL) and cooled down by a dry ice – acetone bath. A hydrogenchloride solution (2.0 M in ether, 141.0 mL, 282 mmol, 12.0 equiv.) was added dropwise. After the formed white slurry was allowed to warm to room temperature overnight, the mixture was filtered through a cannula and the residue was washed with ether (3 x 30 mL). The colorless filtrate was concentrated under reduced pressure to obtain SI01 as a slightly yellow semi-solid (6.94 g, 18.7 mmol, 79 % yield) that can be used for the next step without further purification.

Washing the obtained semi-solid with pentane affords the product as a white crystalline powder.

Crystals suitable for single-crystal X-ray diffraction were grown by slow evaporation of a diethyl ether solution at room temperature.

^{1}H NMR (500 MHz, C_6D_6) δ 7.85 (dqd, *J* = 7.7, 1.8, 0.3 Hz, 2H), 6.89 – 6.85 (m, 1H), 6.82 – 6.77 (m, 2H), 6.39 (dddd, *J* = 8.1, 2.9, 2.5, 1.1 Hz, 2H) ppm.

$^{13}C\{^1H\}$ (126 MHz, C_6D_6) δ 158.0 – 157.7 (m), 134.3, 131.5 (t, *J* = 3.7 Hz), 131.2 (dd, *J* = 63.8, 2.9 Hz), 125.3, 118.4 ppm.

$^{31}P\{^1H\}$ (202 MHz, C_6D_6) δ 157.5 ppm.

(Oxydi-2,1-phenylene)bis{bis[3,5-bis(trifluoromethyl)phenyl]phosphine} L05

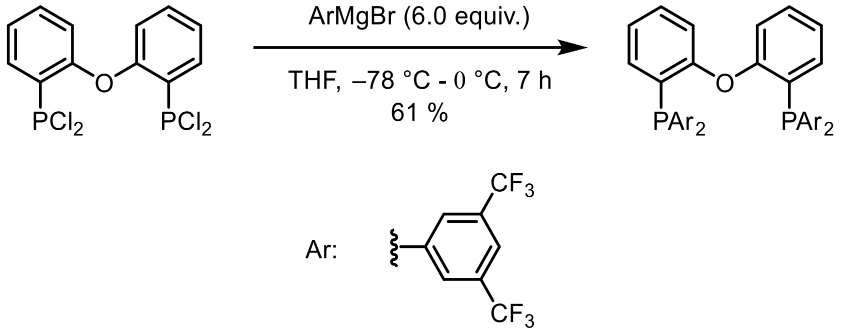

Under an atmosphere of dry nitrogen, (3,5-bis(trifluoromethyl)phenyl)magnesium bromide (1.0 M in THF, 72.0 mL, 72.0 mmol, 6.0 equiv.) prepared freshly from magnesium turnings and 1-bromo-3,5-bis(trifluoromethyl)benzene was added in the course of 1.5 h to a solution of SI01 (4.45 g, 12.0 mmol, 1.0 equiv.) at – 78 °C. The brown reaction mixture was stirred at – 78 °C for 2 h, before it was warmed up to 0 °C and stirred for 3.5 h at this temperature. After the reaction mixture was opened to air, sat. NH_4Cl (100 mL) was added, and the mixture was extracted with ether (3 x 100 mL). The combined extract solutions were dried over Na_2SO_4 and concentrated under reduced pressure to obtain a brown oil. The crude product was filtered through a pad of Silica with DCM and the slightly yellow filtrate was concentrated under reduced pressure. The residue was dissolved in refluxing methanol (approx. 80 mL) and upon cooling a beige powder formed that was isolated by filtration. After washing with methanol (4 x 5 mL), the product (7.86 g, 7.26 mmol, 61 % yield) was obtained as a white powder.

Crystals suitable for single-crystal X-ray diffraction were grown by layering the crude product with methanol.

^1H NMR (500 MHz, C_6D_6) δ 7.69 (dq, *J* = 6.3, 1.7 Hz, 8H), 7.64 (hept, *J* = 1.6 Hz, 4H), 6.79 (ddd, *J* = 8.2, 7.3, 1.8 Hz, 2H), 6.59 (ddd, *J* = 7.8, 5.1, 1.8 Hz, 2H), 6.54 (dddd, *J* = 7.8, 7.1, 1.1, 0.7 Hz, 2H), 6.48 (ddd, *J* = 8.3, 4.6, 1.1 Hz, 2H) ppm.

$^{13}C\{^1H\}$ (126 MHz, C_6D_6) δ 158.7 – 158.4 (m), 139.1 – 138.9 (m), 134.1 – 133.9 (m), 133.5 (dd, *J* = 22.6, 2.6 Hz), 132.6, 133.1 – 132.0 (m), 125.5 (t, *J* = 1.0 Hz), 124.9 (d, *J* = 14.8 Hz), 123.6 (hept, *J* = 3.9 Hz), 123.4 (q, *J* = 273.2 Hz), 118.1 (t, *J* = 1.1 Hz) ppm.

^{19}**F{^{1}H}** (471 MHz, C_6D_6) δ – 63.0 ppm.

^{31}**P{^{1}H}** (202 MHz, C_6D_6) δ –13.7 ppm.

^{1}**H NMR** (500 MHz, $CDCl_3$) δ 7.84 (q, *J* = 1.7, 1.3 Hz, 4H), 7.62 (dd, *J* = 5.2, 1.6 Hz, 8H), 7.38 (ddd, *J* =8.2, 7.2, 1.6 Hz, 2H), 7.13 (tdd, *J* = 7.5, 1.1, 0.5 Hz, 2H), 6.77 (ddd, *J* = 8.2, 4.8, 1.0 Hz, 2H), 6.70 (ddd, *J* = 7.7, 4.9, 1.6 Hz, 2H) ppm.

^{13}**C{^{1}H}** (126 MHz, $CDCl_3$) δ 158.3 (d, *J* = 17.6 Hz), 138.5 (d, *J* = 18.0 Hz), 133.9, 133.5 (d, *J* = 22.2 Hz), 132.5, 132.3 (qt, *J* = 33.4, 3.4 Hz), 125.5, 124.6 (d, *J* = 14.0 Hz), 123.6, 123.0 (q, *J* = 273.2 Hz), 118.2 ppm.

^{19}**F{^{1}H}** (471 MHz, $CDCl_3$) δ – 63.1 ppm

^{31}**P{^{1}H}** NMR (202 MHz, CDC13) δ – 13.5 ppm

TLC: Rf = 0.18 (95:5 hexane:CH_2Cl_2)

HRMS (ESI, m/z): $[M+H]^+$ calcd. for $C_{44}H_{20}F_{24}OP_2$, 1083.0679, found 1083.0662

Bis(2-(bis(3,5-bis(trifluoromethyl)phenyl)phosphaneyl)phenyl)sulfane (L06)

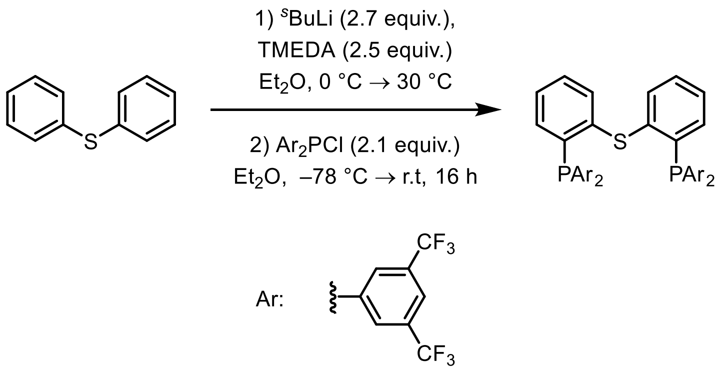

Under an atmosphere of dry nitrogen, diphenylsulfane (933.4 mg, 5.011 mmol, 1.0 equiv.) and TMEDA (1.456 g, 1.88 mL, 12.53 mmol, 2.5 equiv.) were dissolved in diethyl ether (37 mL) in a Schlenk flask. The solution was cooled to 0 °C, and sec-butyllithium (9.5 mL, 1.4 M in hexane, 13 mmol, 2.7 equiv.) was added dropwise, causing the reaction mixture to turn yellow. The reaction was then heated to 30 °C and stirred for 3 hours. By quenching an aliquot of the reaction mixture with D_2O, the progress of the lithiation was monitored by NMR. Additional ^{s}BuLi was added in case of incomplete lithiation. The reaction mixture was then cooled to –78 °C, and bis(3,5-bis(trifluoromethyl)phenyl)chlorophosphine (5.061 g, 10.27 mmol, 2.1

equiv.) dissolved in diethyl ether was added dropwise, resulting in the formation of a dark red mixture. The reaction was allowed to warm to room temperature and stirred overnight, resulting in a black reaction mixture.

The reaction mixture was diluted with ethyl acetate (~100 mL) and washed with water (2 x 110 mL). Brine was used to assist phase separation. The organic layer was dried over $MgSO_4$, filtered, and concentrated under reduced pressure to afford a dark brown-orange oil. This crude product was dissolved in dichloromethane and passed through a silica gel column eluting with dichloromethane. The solvent was removed under reduced pressure to yield an orange viscous oil. The obtained oil was mixed with methanol and sonicated resulting in precipitation of an off-white solid that was subsequently washed with ice-cold methanol. Recrystallization of the product was performed from methanol (32 mL per gram of crude material), yielding off-white crystalline **L06**. Yield: 377 mg (7%).

^{1}H NMR (500 MHz, $CDCl_3$) δ 7.91 – 7.86 (m, 4H), 7.63 – 7.59 (m, 8H), 7.34 (td, *J* = 7.6, 1.6 Hz, 2H), 7.28 (tdd, J = 7.5, 1.4, 0.4 Hz, 2H), 7.18 – 7.14 (m, 2H), 6.83 – 6.79 (m, 2H).

^{13}C{H} (126 MHz, $CDCl_3$) δ 141.2 (dd, *J* = 32.5, 4.3 Hz), 138.8 (d, *J* = 18.5 Hz), 135.9 (d, *J* = 8.6 Hz), 133.9, 133.5 (d, *J* = 21.8 Hz), 133.1 (t, *J* = 2.2 Hz), 132.4 (qt, *J* = 34.0, 2.9 Hz), 131.6, 129.1, 123.7 (d, *J* = 3.9 Hz), 123.0 (q, *J* = 273.2 Hz) ppm

^{19}F{H} (471 MHz, $CDCl_3$) δ – 63.1 ppm

^{31}P{H} (202 MHz, $CDCl_3$) δ – 11.5 ppm

HRMS (ESI, m/z): $[M+H]^+$ calcd. for for $C_{44}H_{21}F_{24}P_2S$, 1099.045; found 1099.045.

Bis(3,5-bis(trifluoromethyl)phenyl)(2-phenoxyphenyl)phosphane (L07) and 10-{3,5-Bis(trifluoromethyl)phenyl}-10*H*-phenoxaphosphinine (SI02)

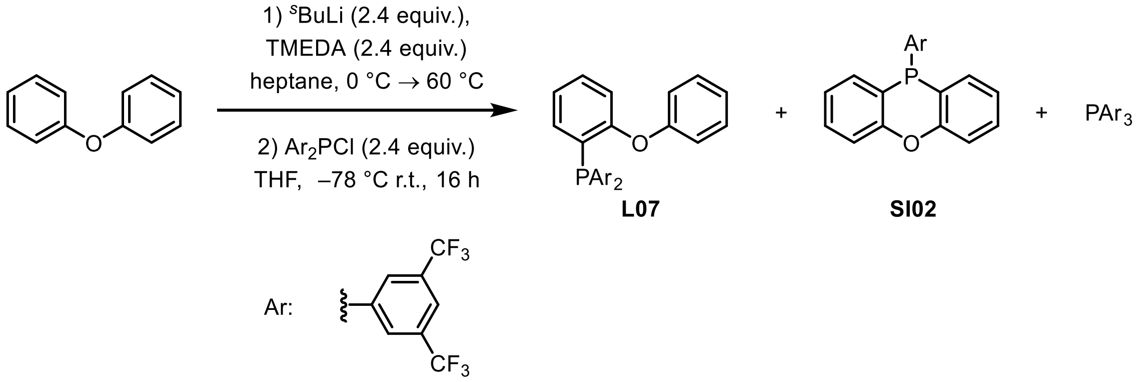

By reacting diphenylether in a process similar to the one used for the synthesis of ligand L01-L04,[1] phenoxaphosphinine **SI02** and ligand **L07** could be obtained. Due to low yields and non-optimized reaction conditions, no yields are reported.

Layering the obtained oily crude product with methanol resulted in precipitation of **SI02**. Crystals of SI02 suitable for single-crystal X-ray diffraction were obtained similarly.

After the formed solids were removed by filtration, the filtrate was concentrated under reduced pressure. The obtained residue was distilled by Kugelrohr distillation at 200 °C and 0.005 mbar to afford pure **L07** as a colorless oil that subsequently solidifies at room temperature.

Bis(3,5-bis(trifluoromethyl)phenyl)(2-phenoxyphenyl)phosphane (L07)

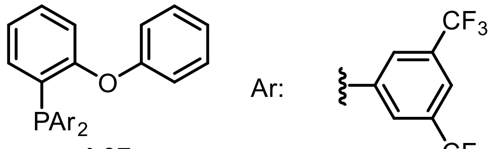

L07

^{1}H NMR (500 MHz, $CDCl_3$) δ 7.89 (tq, *J* = 1.7, 0.8 Hz, 2H), 7.82 (ddt, *J* = 6.7, 1.9, 0.6 Hz, 4H), 7.45 (ddd, *J* = 8.3, 7.4, 1.7 Hz, 1H), 7.31 – 7.24 (m, 3H), 7.17 (tt, J = 7.4, 1.1 Hz, 1H), 7.13 – 7.08 (m, 1H), 7.06 (td, *J* = 7.6, 1.7 Hz, 1H), 6.90 (ddd, *J* = 8.3, 3.9, 1.1 Hz, 1H), 6.77 – 6.73 (m, 2H) ppm.

^{13}C{H} (126 MHz, $CDCl_3$) δ 159.6 (d, *J* = 11.5 Hz), 155.4, 139.0 (d, *J* = 17.6 Hz), 135.1 (d, *J* = 14.6 Hz), 133.5 (d, *J* = 21.1 Hz), 132.7, 132.2 (qd, *J* = 33.4, 6.5 Hz), 130.0, 125.5, 124.4, 124.2 (d, *J* = 5.4 Hz), 123.4 (p, *J* = 3.9 Hz), 123.2 (q, *J* = 273.2 Hz), 119.1, 117.9 (d, *J* = 1.5 Hz) ppm.

^{19}F{H} (471 MHz, $CDCl_3$) δ – 63.0 ppm

^{31}P{H} (202 MHz, $CDCl_3$) δ – 10.6 ppm

HRMS (ESI, m/z): $[M+H]^+$ calcd. for for $C_{28}H_{16}F_{12}OP$ 1083.0679; found 1083.0662.

10-{3,5-Bis(trifluoromethyl)phenyl}-10*H*-phenoxaphosphinine (SI02)

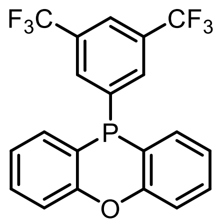

^{1}H NMR (400 MHz, C_6D_6) δ 7.57 (dd, *J* = 6.2, 1.7 Hz, 2H), 7.40 (tt, *J* = 1.8, 0.9 Hz, 1H), 7.31 (ddd, *J* = 10.8, 7.5, 1.7 Hz, 2H), 7.04 – 6.99 (m, 2H), 6.95 (ddd, *J* = 8.3, 7.1, 1.7 Hz, 2H), 6.77 (tt, *J* = 7.3, 1.5 Hz, 2H) ppm.

^{19}F{H} (377 MHz, C_6D_6) δ – 62.90 ppm.

^{31}P{H} (162 MHz, C_6D_6) δ – 56.37 ppm.

HRMS (ESI, m/z): $[M+H]^+$ calcd. for for $C_{20}H_{12}F_6OP$ 413.0524; found 413.0530.

Optimization

General procedure for bidentate ligand screening

In a glovebox, a 4 mL screw-cap vial equipped with a stirring bar was charged with $Pd(dba)_2$ (5.0 mol%, 15 µmol, 8.7 mg) and the indicated bisphosphine ligand (**SL01** – **SL13, L01** – **L06** 11 mol%, 33 µmol) and then *o*-xylene (0.5 mL). The solution was stirred at room temperature for 10 min. To a pre-mixed solution were added 5-decyne **2a** (1.5 equiv., 0.45 mmol) and mesitoyl chloride **1a** (1.0 equiv., 0.30 mmol). The reaction mixture was stirred at 80 °C for 24 h. The reaction was cooled to room temperature then diluted with MeOH (1 mL) and n-dodecane was added as an internal standard, and was stirred for additional 1 h. The resulting solution was filtered through a celite/silica plug and the filtrate was analyzed by GC/FID and GC/MS.

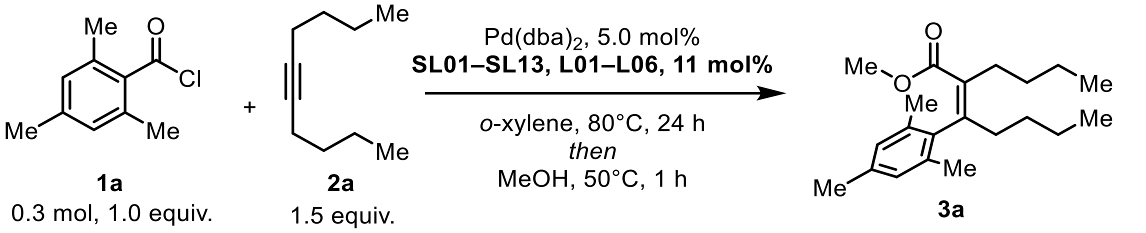

Entry	Ligand	3a %
1	SL01	5
2	SL02	0
3	SL03	0
4	SL04	0
5	SL05	1
6	SL06	0
7	SL07	3
8	SL08	1
9	SL09	5
10	SL10	3
11	SL11	1
12	SL12	3
13	SL13	0
14	L01	12
15	L02	21
16	L03	61
17	L04	6
**18**	**L05**	**77**
19	L06	45
20	No ligand	0

Reaction conditions: MesCOCl (X, 0.3 mmol, 1.0 equiv.), 5-decyne (Y, 0.45 mmol, 1.5 equiv.), $Pd(dba)_2$ (5.0 mol %), SL01-SL13 or L01-L06 (11 mol %), o-xylene, 80 °C, 24 h; then MeOH, 50 °C, 1 h; GC yield of **3a** obtained using n-dodecane as internal standard.

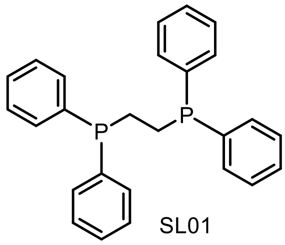

SL01

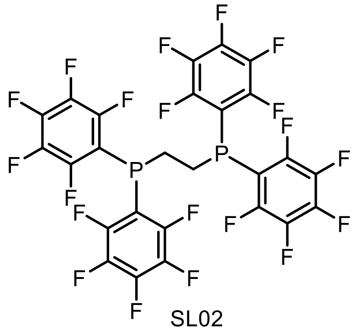

SL02

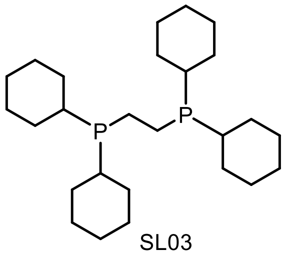

SL03

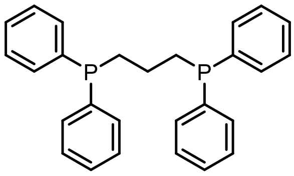

SL04

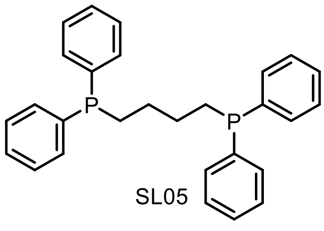

SL05

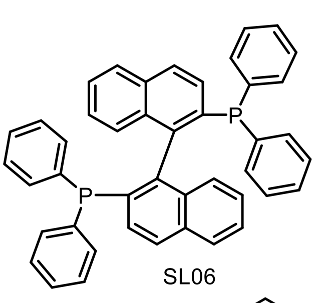

SL06

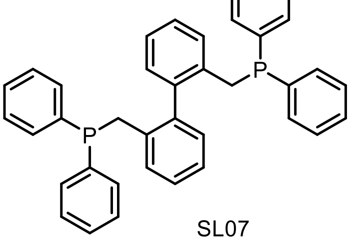

SL07

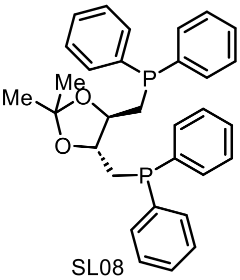

SL08

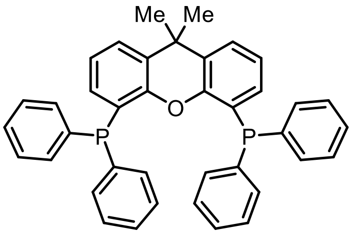

SL09

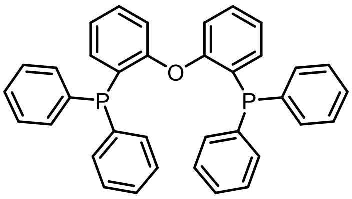

SL10

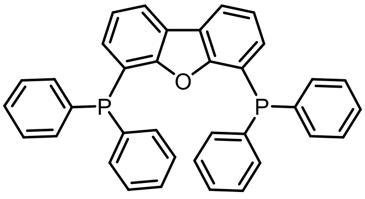

SL11

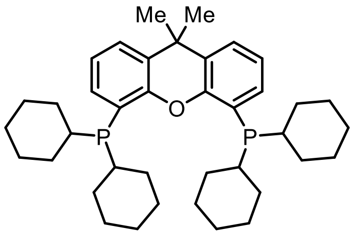

SL12

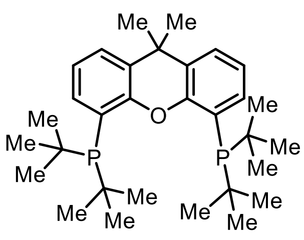

SL13

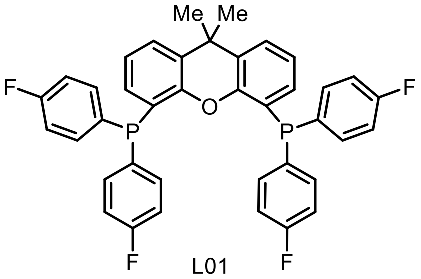

L01

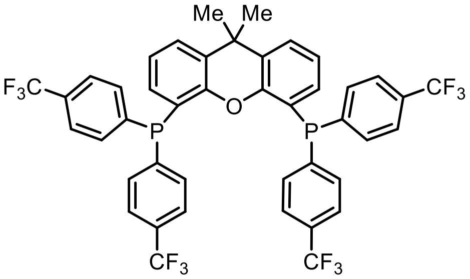

L02

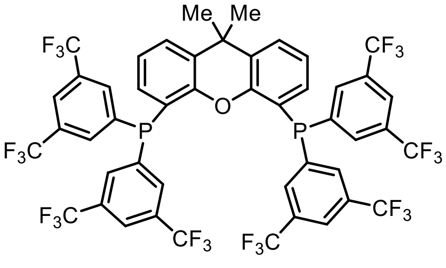

L03

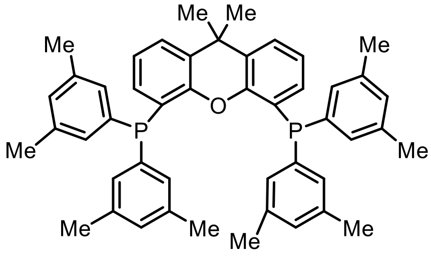

L04

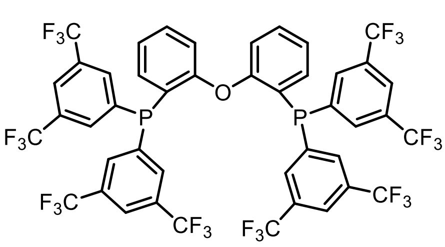

L05

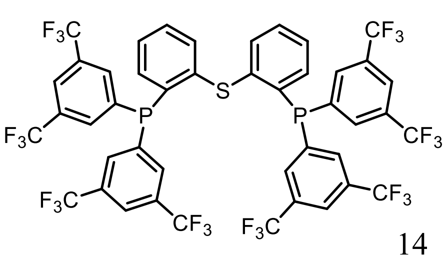

L06

General procedure for monodentate ligand screening

In a glovebox, a 4 mL screw-cap vial equipped with a stirring bar was charged with $Pd(dba)_2$ (5.0 mol%, 15 µmol, 8.7 mg) and the indicated monophosphine ligand (**SL20** – **SL34, L07**, 22 mol%, 22.50 µmol) and then *o*-xylene (0.5 mL). The solution was stirred at room temperature for 10 min. To a pre-mixed solution were 5-decyne **2a** (1.5 equiv., 0.45 mmol) and mesitoyl chloride **1a** (1.0 equiv., 0.30 mmol). The reaction mixture was stirred at 80 °C for 24 h. The reaction was cooled to room temperature then diluted with MeOH (1 mL) and n-dodecane was added as an internal standard, and was stirred for additional 1 h. The resulting solution was filtered through a celite/silica plug and the filtrate was analyzed by GC/FID and GC/MS.

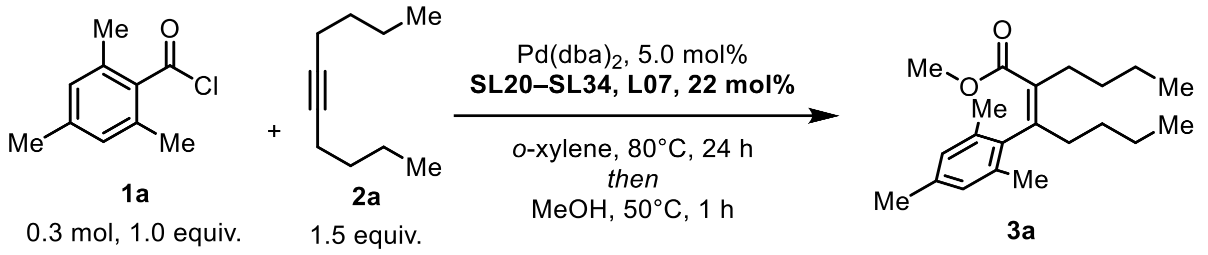

Entry	Ligand	3a %
21	SL20	4
21	SL21	3
23	SL22	6
24	SL23	0
25	SL24	2
26	SL25	0
27	SL26	0
28	SL27	0
29	SL28	3
30	SL29	0
31	SL30	0
32	SL31	0
33	SL32	0
34	SL33	0
35	SL34	0
36	L07	36

Reaction conditions: MesCOCl (X, 0.3 mmol, 1.0 equiv.), 5-decyne (Y, 0.45 mmol, 1.5 equiv.), $Pddba_2$ (5.0 mol %), **SL20-SL34** or **L07** (22 mol %), o-xylene, 80 °C, 24 h; then MeOH, 50 °C, 1 h; GC yield of **3a** obtained using n-dodecane as internal standard.

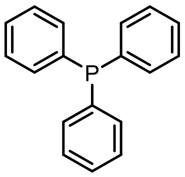

SL20

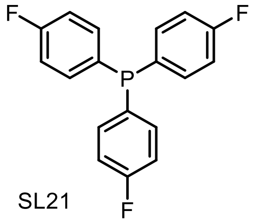

SL21

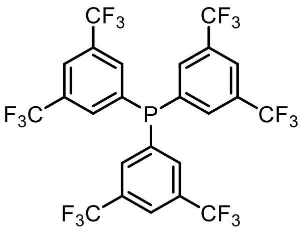

SL22

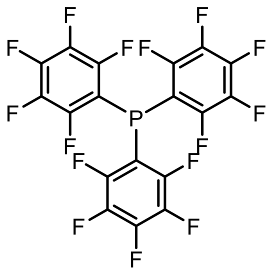

SL23

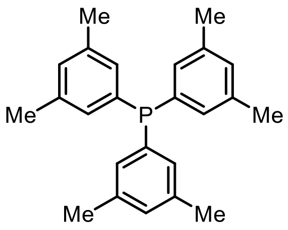

SL24

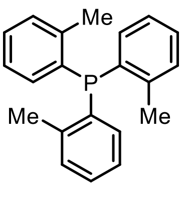

SL25

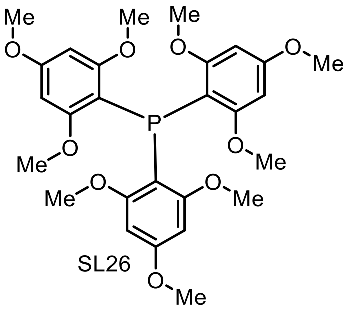

SL26

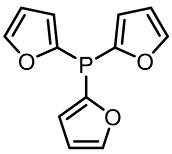

SL27

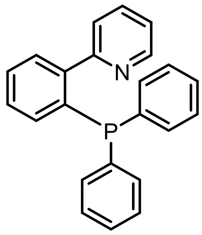

SL28

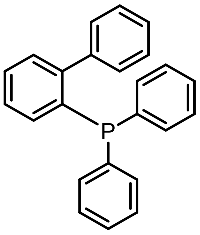

SL29

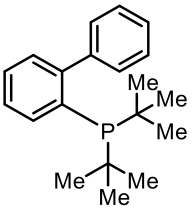

SL30

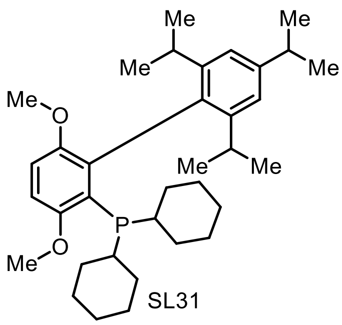

SL31

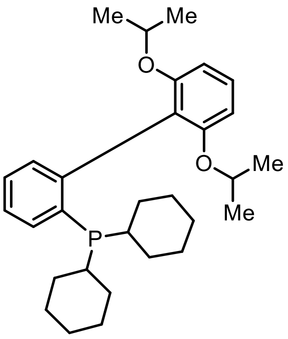

SL32

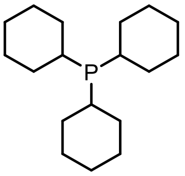

SL33

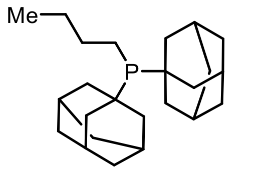

SL34

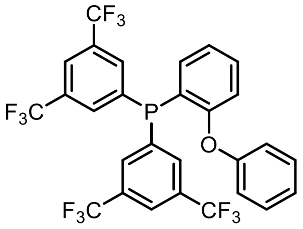

L07

General procedure for transition metal precursor screening

In a glovebox, a 4 mL screw-cap vial equipped with a stirring bar was charged with a transition metal (5 mol%, 15 µmol, as a metal atom) and the ligand (**L05,** 11 mol%, 33 µmol) then *o*-xylene (0.5 mL). The solution was stirred at room temperature for 10 min. To a pre-mixed solution were added 5-decyne **2a** (1.5 equiv., 0.45 mmol) and mesitoyl chloride **1a** (1.0 equiv., 0.30 mmol). The reaction mixture was stirred at 80 °C for 24 h. The reaction was cooled to room temperature then diluted with MeOH (1 mL) and n-dodecane was added as an internal standard, and was stirred for additional 1 h. The resulting solution was filtered through a celite/silica plug and the filtrate was analyzed by GC/FID and GC/MS.

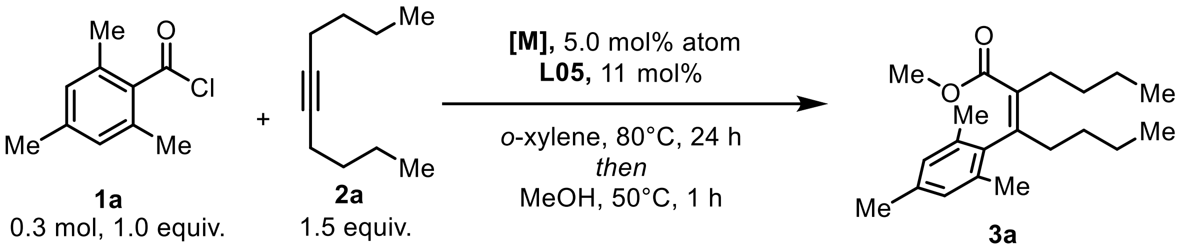

1a
0.3 mol, 1.0 equiv.

2a
1.5 equiv.

3a

.

Entry	Ligand	3a %
1	[Pd(allyl)Cl]$_2$	52
2	Pd$_2$(dba)$_3$	67
3	Pd$_2$(dba)$_3$·CHCl$_3$	71
**4**	Pd(dba)$_2$	**77**
5	PdCl$_2$	0
6	Pd(OAc)$_2$	0
7	NiCl$_2$	0
8	Ni(cod)$_2$	0
9	[Ir(cod)Cl]$_2$	0
10	[Ir(cod)OMel]$_2$	0
11	[Rh(cod)Cl]$_2$	0
12	RhCl$_3$	0
13	Ru(cod)Cl$_2$	0
14	K[PtCl$_3$(C$_2$H$_4$)]·H$_2$O	0
15	No metal	0
16	No metal, no ligand	0

Reaction conditions: MesCOCl (X, 0.3 mmol, 1.0 equiv.), 5-decyne (Y, 0.45 mmol, 1.5 equiv.), TM, 5.0 mol% atom 3,5-(CF$_3$)$_2$DPEphos (**L05**), 11 mol%, o-xylene, 80 °C, 24 h; then MeOH, 50 °C, 1 h; GC yield of **3a** obtained using n-dodecane as internal standard.

General procedure for catalytic loading screening

In a glovebox, a 4 mL screw-cap vial equipped with a stirring bar was charged with $Pddba_2$ (0.60 – 15.0 mol%) and the ligand (**L05**, 11 mol%, 33 µmol) then *o*-xylene (0.5 mL). The solution was stirred at room temperature for 10 min. To a pre-mixed solution were added 5-decyne **2a** (1.5 equiv., 0.45 mmol) and mesitoyl chloride **1a** (1.0 equiv., 0.30 mmol). The reaction mixture was stirred at 80 °C for 24 h. The reaction was cooled to room temperature then diluted with MeOH (1 mL) and n-dodecane was added as an internal standard, and was stirred for additional 1 h. The resulting solution was filtered through a celite/silica plug and the filtrate was analyzed by GC/FID and GC/MS.

.

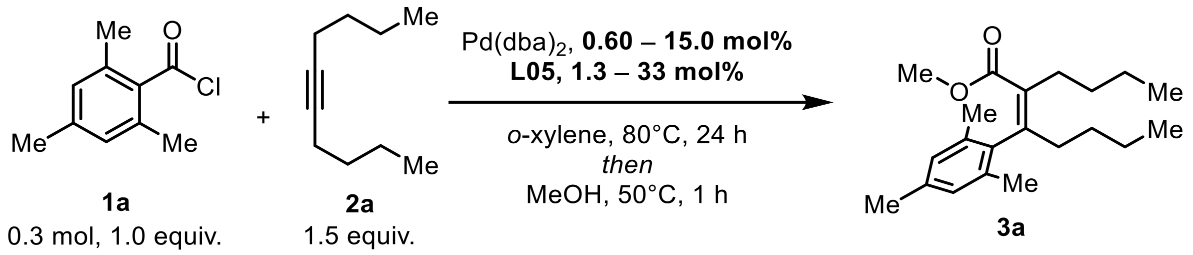

Entry	$Pd(dba)_2$ mol%	L05 mol%	3a %
1	0.6	1.32	2
2	1.0	2.2	13
4	2.5	5.5	26
5	4.0	8.8	74
**6**	**5.0**	**11**	**77**
7	7.5	16.5	39
8	10	22	26
9	15	33	41

Reaction conditions: MesCOCl (X, 0.3 mmol, 1.0 equiv.), 5-decyne (Y, 0.45 mmol, 1.5 equiv.), $Pd(dba)_2$, 0.60- 15.0 mol% atom, 3,5-$(CF_3)_2$DPEphos (L05), 11 mol%, o-xylene, 80 °C, 24 h; then MeOH, 50 °C, 1 h; GC yield of 3a obtained using n-dodecane as internal standard.

General procedure for P/Pd atomic ratio screening

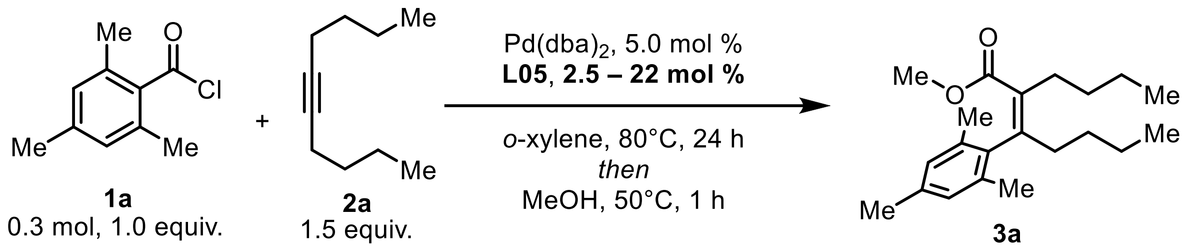

In a glovebox, a 4 mL screw-cap vial equipped with a stirring bar was charged with Pd(dba)2 (5.0 mol%, 15 μmol, 8.7 mg) and the ligand (**L05**, 2.5 – 22 mol%, 33 μmol) then *o*-xylene (0.5 mL). The solution was stirred at room temperature for 10 min. To a pre-mixed solution were added 5-decyne **2a** (1.5 equiv., 0.45 mmol) and mesitoyl chloride **1a** (1.0 equiv., 0.30 mmol). The reaction mixture was stirred at 80 °C for 24 h. The reaction was cooled to room temperature then diluted with MeOH (1 mL) and n-dodecane was added as an internal standard, and was stirred for additional 1 h. The resulting solution was filtered through a celite/silica plug and the filtrate was analyzed by GC/FID and GC/MS.

Entry	Ligand %	3a %
1	22	70
**2**	**11**	**77**
3	10	71
4	5	75
5	2.5	3

Reaction conditions: MesCOCl (X, 0.3 mmol, 1.0 equiv.), 5-decyne (Y, 0.45 mmol, 1.5 equiv.), Pd(dba)2, 5.0 mol% atom, (**L05**), 2.5-22 mol%, o-xylene, 80 °C, 24 h; then MeOH, 50 °C, 1 h; GC yield of **3a** obtained using n-dodecane as internal standard.

General procedure for solvent screening

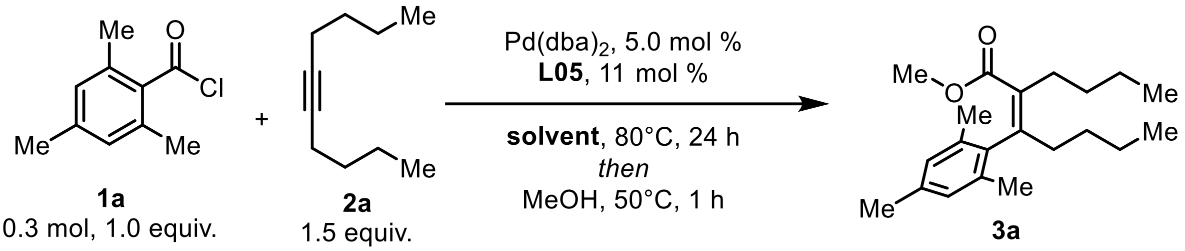

In a glovebox, a 4 mL screw-cap vial equipped with a stirring bar was charged with $Pd(dba)_2$ (5.0 mol%, 15 μmol, 8.7 mg) and the ligand (**L05** 11 mol%, 33 μmol), then indicated solvent (0.5 mL). The solution was stirred at room temperature for 10 min. To a pre-mixed solution were added 5-decyne **2a** (1.5 equiv., 0.45 mmol) and mesitoyl chloride **1a** (1.0 equiv., 0.30 mmol). The reaction mixture was stirred at 80 °C for 24 h. The reaction was cooled to room temperature then diluted with MeOH (1 mL) and n-dodecane was added as an internal standard, and was stirred for additional 1 h. The resulting solution was filtered through a celite/silica plug and the filtrate was analyzed by GC/FID and GC/MS.

Entry	Ligand %	3a %
1	toluene	66
2	Dioxane	40
3	Chlorobenzene	28
4	Trifluorotoluene	63
5	Benzonitrile	0
6	Acetonitrile	61
7	N-methylpyrrolidine	5
8	DCE	4
9	Heptane	72
10	nitrobenzene	3
**9**	***o*-xylene**	**77**

Reaction conditions: MesCOCl (X, 0.3 mmol, 1.0 equiv.), 5-decyne (Y, 0.45 mmol, 1.5 equiv.), $Pd(dba)_2$, 5.0 mol% atom, 3,5-$(CF_3)_2$DPEphos (**L05**), 11 mol%, solvent, 80 °C, 24 h; then MeOH, 50 °C, 1 h; GC yield of **3a** obtained using n-dodecane as internal standard.

General procedure for substrate equivalent ratio screening

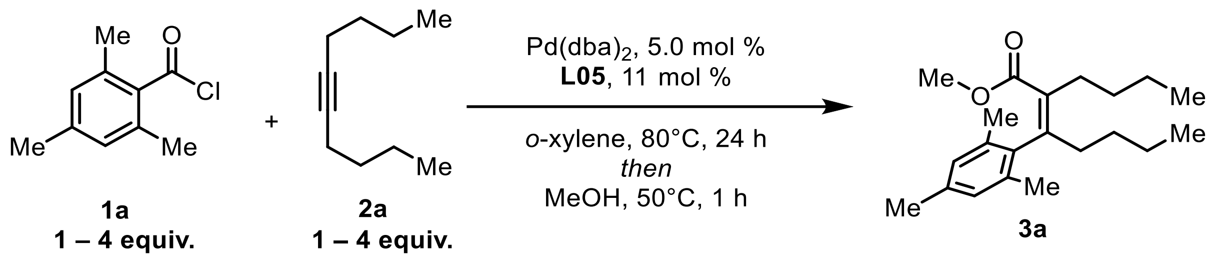

1.0 equiv. = 0.3 mol

In a glovebox, a 4 mL screw-cap vial equipped with a stirring bar was charged with Pd(dba)$_2$ (5.0 mol%, 15 µmol, 8.7 mg) and the ligand (**L05**, 11 mol%, 33 µmol), then *o*-xylene (0.5 mL). The solution was stirred at room temperature for 10 min. To a pre-mixed solution were added 5-decyne **2a** (1.0 – 4.0 equiv., 1 equiv. = 0.30 mmol) and mesitoyl chloride **1a** (1.0 – 4.0 equiv., 1 equiv. = 0.30 mmol). The reaction mixture was stirred at 80 °C for 24 h. The reaction was cooled to room temperature then diluted with MeOH (1 mL) and n-dodecane was added as an internal standard, and was stirred for additional 1 h. The resulting solution was filtered through a celite/silica plug and the filtrate was analyzed by GC/FID and GC/MS.

Entry	Y:X	3a %
1	4:1	61
**2**	**2:1**	**77**
3	1:1	28
4	1:2	41
5	1:4	49

Reaction conditions: MesCOCl (X, 1 – 4 equiv.), 5-decyne (Y, 1 – 4 equiv.), 1.0 equiv. = 0.3 mmol, Pd(dba)$_2$, 5.0 mol%, 3,5-(CF$_3$)$_2$DPEphos (**L05**), 11 mol%, o-xylene, 80 °C, 24 h; then MeOH, 50 °C, 1 h; GC yield of **3a** obtained using n-dodecane as internal standard.

General procedure for time screening

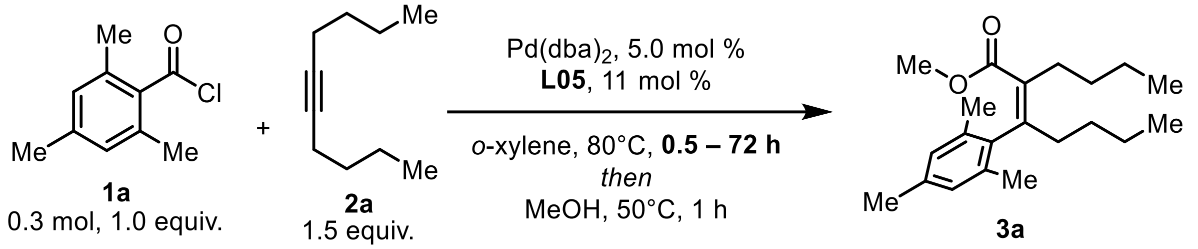

In a glovebox, a 4 mL screw-cap vial equipped with a stirring bar was charged with Pd(dba)2 (5.0 mol%, 15 μmol, 8.7 mg) and the ligand (**L05**, 11 mol%, 33 μmol), then *o*-xylene (0.5 mL). The solution was stirred at room temperature for 10 min. To a pre-mixed solution were added 5-decyne **2a** (1.5 equiv., 0.45 mmol) and mesitoyl chloride **1a** (1.0 equiv., 0.30 mmol). The reaction mixture was stirred at 80 °C for a designated amount of time (0.5 – 72 h). The reaction was cooled to room temperature then diluted with MeOH (1 mL) and n-dodecane was added as an internal standard, and was stirred for additional 1 h. The resulting solution was filtered through a celite/silica plug and the filtrate was analyzed by GC/FID and GC/MS.

Entry	Time (h)	3a %
1	0.5	32
2	1	78
3	2	76
**4**	**24**	**77**
5	72	72

Reaction conditions: MesCOCl (X, 0.3 mmol, 1.0 equiv.), 5-decyne (Y, 0.45 mmol, 1.5 equiv.), Pd(dba)2, 5.0 mol% atom, 3,5-(CF3)2DPEphos (**L05**), 11 mol%, o-xylene, 80 °C, 0.5 – 72 h; then MeOH, 50 °C, 1 h; GC yield of **3a** obtained using n-dodecane as internal standard.

General procedure for temperature screening

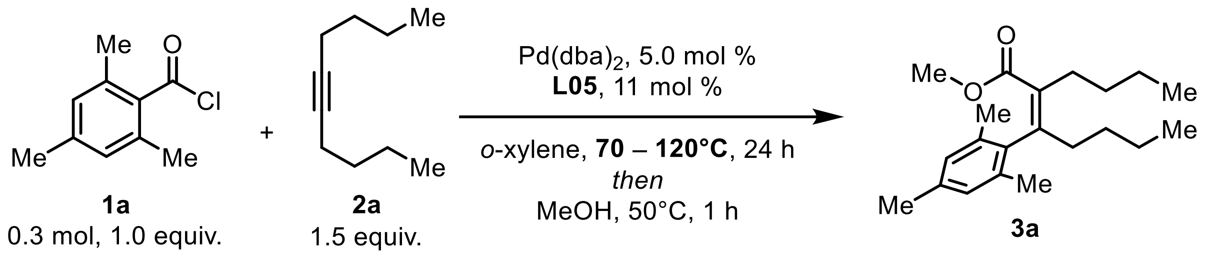

In a glovebox, a 4 mL screw-cap vial equipped with a stirring bar was charged with Pddba$_2$ (2.5 mol%, 7.5 µmol, 2.7 mg) and the ligand (**L05**, 11 mol%, 33 µmol) then *o*-xylene (0.5 mL). The solution was stirred at room temperature for 10 min. To a pre-mixed solution were added 5-decyne **2a** (1.5 equiv., 0.45 mmol) and mesitoyl chloride **1a** (1.0 equiv., 0.30 mmol). The reaction mixture was stirred at designated temperature (70 – 120 °C) for 24 h. The reaction was cooled to room temperature then diluted with MeOH (1 mL) and n-dodecane was added as an internal standard, and was stirred for additional 1 h. The resulting solution was filtered through a celite/silica plug and the filtrate was analyzed by GC/FID and GC/MS.

Entry	Temperature (°C)	3a %
1	70	55
**2**	**80**	**77**
3	90	82*
4	100	55**
5	110	31**
7	120	13**

Reaction conditions: MesCOCl (X, 0.3 mmol, 1.0 equiv.), 5-decyne (Y, 0.45 mmol, 1.5 equiv.), Pd(dba)$_2$, 5.0 mol% atom, 3,5-(CF$_3$)$_2$DPEphos (**L05**), 11 mol%, o-xylene, 70-120 °C, 24 h; then MeOH, 50 °C, 1 h; GC yield of **3a** obtained using n-dodecane as internal standard.

Even though 90 °C seemed like a more optimal temperature, in the substrate scope 80 °C was used since many different acid chlorides gave inseparable isomers at 90 °C (see mechanistic investigations).

*****Other isomers and cyclization products start to appear. For more details on the isomerization products, refer to the mechanistic investigations or the cyclopentenone formation.***

General procedure for ligand screening

In a glovebox, a 4 mL screw-cap vial equipped with a stirring bar was charged with $[Pd(allyl)Cl]_2$ (2.5 mol%, 7.5 µmol, 2.7 mg) and the ligand (11 mol% for bidentate phosphines, 22 mol% for monodentate ligands) and then *o*-xylene (0.5 mL). The solution was stirred at room temperature for 10 min. To a pre-mixed solution were added 5-decyne (2.0 equiv., 0.60 mmol, 83.0 mg), followed sequentially by mesitoyl chloride (1.0 equiv., 0.30 mmol, 54.8 mg) di-*tert*-butylpyridine (1.0 equiv., 0.3 mmol, 57.4 mg). The vial was then capped, removed from the glovebox, and placed on a preheated block with stirring at 150 °C for 16 h. After the time, the reaction was taken off the heating block and allowed to cool to room temperature. Then methanol (0.2 mL) was added, followed by dodecane (0.3 mmol, 68.1 µL).[a] A crude aliquot was then filtered through a silica plug rinsing with dichloromethane and analyzed by GC-FID.

Notes:[a]Due to solids forming at the top of the vial, the vial was vigorously shaken prior to opening after the reaction.

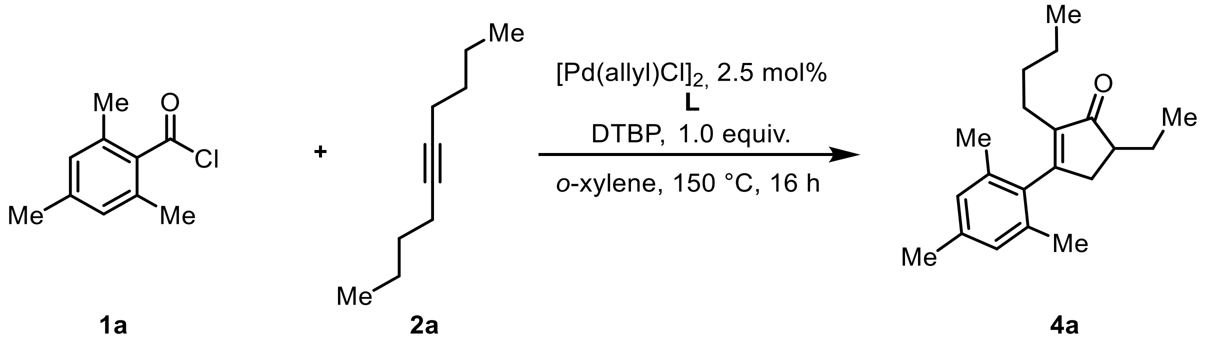

Entry	Ligand	4a %
1	SL01	0
2	SL02	0
3	SL06	0
4	SL07	9
5	SL09	19
6	SL10	21
7	SL11	0
8	SL12	1
9	SL13	0
10	SL20	0

11	SL22	26
12	SL32	0
13	SL33	0
14	L01	22
15	L02	50
16	L03	66
17	L04	18
**18**	**L05**	**73**

General procedure for metal precursor screening

In a glovebox, a 4 mL screw-cap vial equipped with a stirring bar was charged with metal precursor (5 mol% in [M], 7.5 μmol) and the 3,5-CF_3DPEPhos (11 mol%, 0.033 mmol, 37.0 mg) and then *o*-xylene (0.5 mL). The solution was stirred at room temperature for 10 min. To a pre-mixed solution were added 5-decyne (2.0 equiv., 0.60 mmol, 83.0 mg), followed sequentially by mesitoyl chloride (1.0 equiv., 0.30 mmol, 54.8 mg) di-*tert*-butylpyridine (1.0 equiv., 0.3 mmol, 57.4 mg). The vial was then capped, removed from the glovebox, and placed on a preheated block with stirring at 150 °C for 16 h. After the time, the reaction was taken off the heating block and allowed to cool to room temperature. Then methanol (0.2 mL) was added, followed by dodecane (0.3 mmol, 68.1 μL).[a] A crude aliquot was then filtered through a silica plug rinsing with dichloromethane and analyzed by GC-FID.

Notes:[a]Due to solids forming at the top of the vial, the vial was vigorously shaken prior to opening after the reaction.

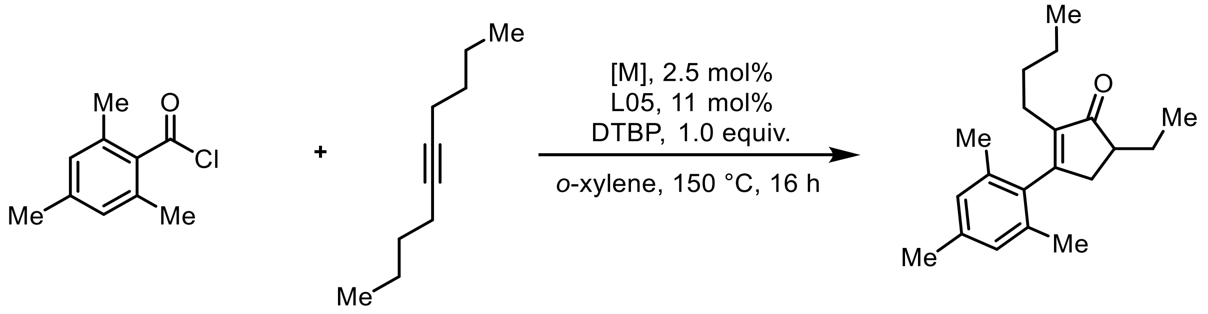

Entry	Metal Precursor	4a %
1	$[Pd(allyl)Cl]_2$	67
2	$Pd_2(dba)_3$	76
**3**	**$Pd(dba)_2$**	**78**
4	$PdCl_2$	66
5	$Pd(OAc)_2$	64
6	$Ni(cod)_2$	0
7	$[Rh(cod)Cl]_2$	0
8	$[Rh(cod)Cl]_2$	0
9	No metal	0

General procedure for ligand loading screening

In a glovebox, a 4 mL screw-cap vial equipped with a stirring bar was charged with $Pd(dba)_2$ (5 mol%, 7.5 µmol, 8.6 mg) and the 3,5-CF_3DPEPhos (X mol%) and then o-xylene (0.5 mL). The solution was stirred at room temperature for 10 min. To a pre-mixed solution were added 5-decyne (2.0 equiv., 0.60 mmol, 83.0 mg), followed sequentially by mesitoyl chloride (1.0 equiv., 0.30 mmol, 54.8 mg) di-*tert*-butylpyridine (1.0 equiv., 0.3 mmol, 57.4 mg). The vial was then capped, removed from the glovebox, and placed on a preheated block with stirring at 150 °C for 16 h. After the time, the reaction was taken off the heating block and allowed to cool to room temperature. Then methanol (0.2 mL) was added, followed by dodecane (0.3 mmol, 68.1 µL).[a] A crude aliquot was then filtered through a silica plug rinsing with dichloromethane and analyzed by GC-FID.

Notes: [a]Due to solids forming at the top of the vial, the vial was vigorously shaken prior to opening after the reaction.

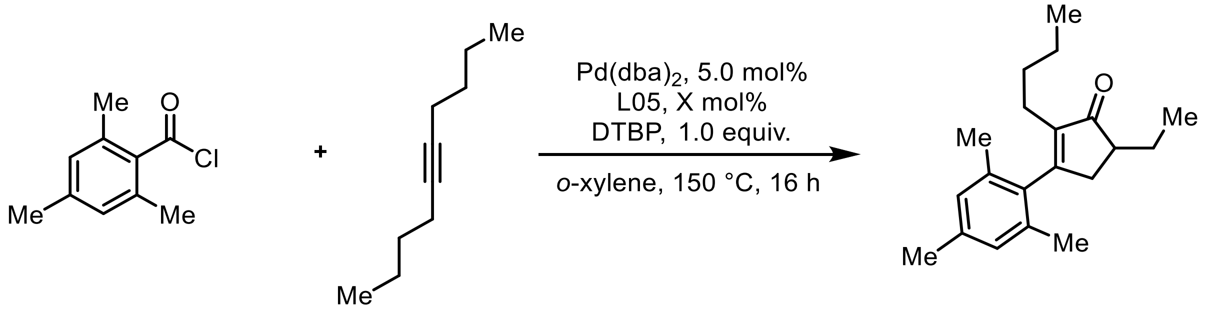

1a 2a 4a

Entry	Ligand Loading (mol%)	4a %
1	2.5	8
2	5.0	45
3	10	65
4	11	69
5	25	71

General procedure for catalyst loading at a constant Pd:ligand ratio

In a glovebox, a 4 mL screw-cap vial equipped with a stirring bar was charged with $Pd(dba)_2$ (X mol%) and the 3,5-CF_3DPEPhos (2.2 × X mol%) and then *o*-xylene (0.5 mL). The solution was stirred at room temperature for 10 min. To a pre-mixed solution were added 5-decyne (2.0 equiv., 0.60 mmol, 83.0 mg), followed sequentially by mesitoyl chloride (1.0 equiv., 0.30 mmol, 54.8 mg) and di-*tert*-butylpyridine (1.0 equiv., 0.3 mmol, 57.4 mg). The vial was then capped, removed from the glovebox, and placed on a preheated block with stirring at 150 °C for 16 h. After the time, the reaction was taken off the heating block and allowed to cool to room temperature. Then methanol (0.2 mL) was added, followed by dodecane (0.3 mmol, 68.1 µL).[a] A crude aliquot was then filtered through a silica plug rinsing with dichloromethane and analyzed by GC-FID.

Notes: [a]*Due to solids forming at the top of the vial, the vial was vigorously shaken prior to opening after the reaction.*

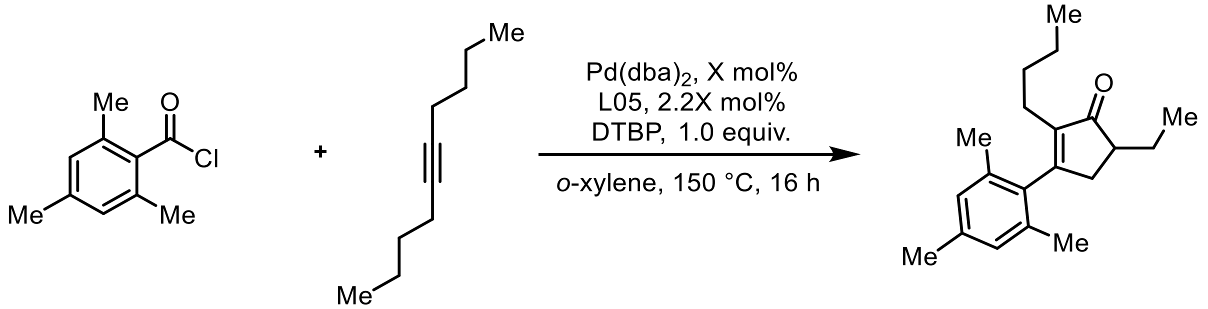

1a 2a 4a

Entry	Catalyst Loading (mol% for Pd)	4a %
1	1	8
2	2	23
3	4	49
**4**	**5**	**66**
5	10	74
6	15	70

General procedure for acid chloride:alkyne ratio

In a glovebox, a 4 mL screw-cap vial equipped with a stirring bar was charged with $Pd(dba)_2$ (5 mol%, 7.5 µmol, 8.6 mg) and the 3,5-CF_3DPEPhos (11 mol%, 0.033 mmol, 37.0 mg) and then *o*-xylene (0.5 mL). The solution was stirred at room temperature for 10 min. To a pre-mixed solution were added 5-decyne (X equiv), followed sequentially by mesitoyl chloride (X equiv.)[a] and di-*tert*-butylpyridine (1.0 equiv., 0.3 mmol, 57.4 mg). The vial was then capped, removed from the glovebox, and placed on a preheated block with stirring at 150 °C for 16 h. After the time, the reaction was taken off the heating block and allowed to cool to room temperature. Then methanol (0.2 mL) was added, followed by dodecane (0.3 mmol, 68.1 µL).[b] A crude aliquot was then filtered through a silica plug rinsing with dichloromethane and analyzed by GC-FID.

Notes: [a]The limiting reagent in the reaction was used at 0.3 mmol. [b]Due to solids forming at the top of the vial, the vial was vigorously shaken prior to opening after the reaction.

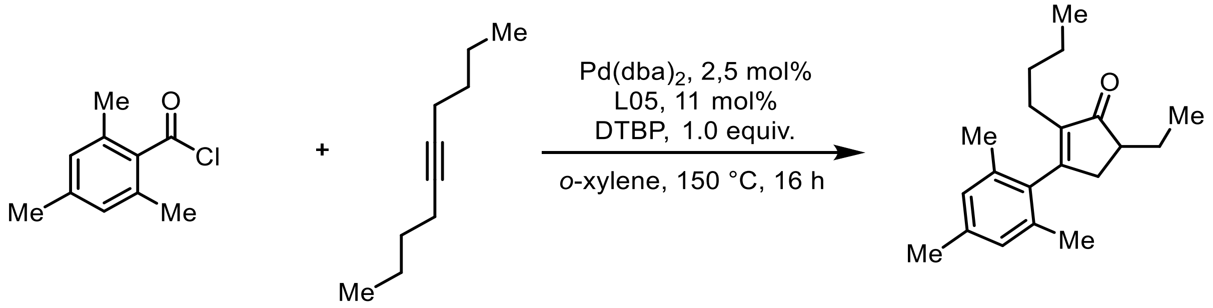

1a **2a** **4a**

Entry	Acid chloride:alkyne ration	4a %
1	4:1	31
2	2:1	46
3	1.5:1	51
4	1:1	59
**5**	**1:1.5**	**72**
6	1:2	69
7	1:4	59

General procedure for solvent screening

In a glovebox, a 4 mL screw-cap vial equipped with a stirring bar was charged with Pd(dba)$_2$ (5 mol%, 7.5 µmol, 8.6 mg) and the 3,5-CF$_3$DPEPhos (11 mol%, 0.033 mmol, 37.0 mg) and then solvent (0.5 mL). The solution was stirred at room temperature for 10 min. To a pre-mixed solution were added 5-decyne (1.5 equiv., 0.45 mmol, 62.2 mg), followed sequentially by mesitoyl chloride (1.0 equiv., 0.30 mmol, 54.8 mg) and di-*tert*-butylpyridine (1.0 equiv., 0.3 mmol, 57.4 mg). The vial was then capped, removed from the glovebox, and placed on a preheated block with stirring at 150 °C for 16 h. After the time, the reaction was taken off the heating block and allowed to cool to room temperature. Then methanol (0.2 mL) was added, followed by dodecane (0.3 mmol, 68.1 µL).[a] A crude aliquot was then filtered through a silica plug rinsing with dichloromethane and analyzed by GC-FID.

Notes: [a]Due to solids forming at the top of the vial, the vial was vigorously shaken prior to opening after the reaction.

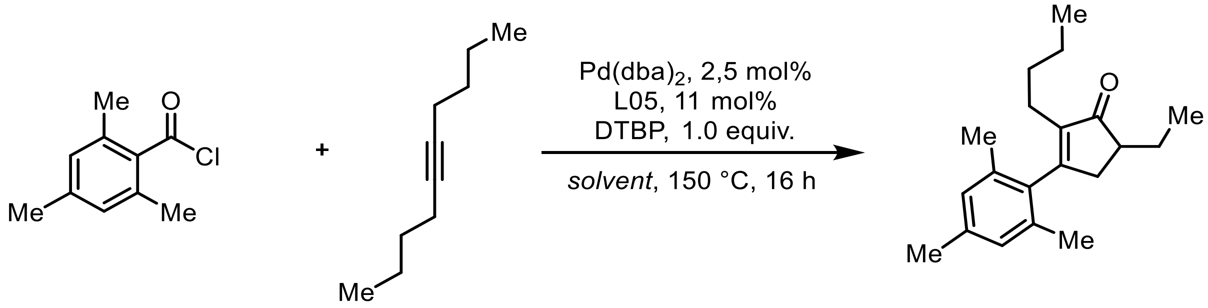

1a **2a** **4a**

Entry	Solvent	4a %
**1**	***o*-xylene**	**66**
2	*p*-xylene	56
3	Toluene	59
4	Dioxane	36
5	PhCl	30
6	PhCF$_3$	26
7	DMI	16
8	DMA	18
9	NMP	20

General procedure for concentration screening

In a glovebox, a 4 mL screw-cap vial equipped with a stirring bar was charged with Pd(dba)2 (5 mol%, 7.5 µmol, 8.6 mg) and the 3,5-CF3DPEPhos (11 mol%, 0.033 mmol, 37.0 mg) and then *o*-xylene (X mL). The solution was stirred at room temperature for 10 min. To a pre-mixed solution were added 5-decyne (1.5 equiv., 0.45 mmol, 62.2 mg), followed sequentially by mesitoyl chloride (1.0 equiv., 0.30 mmol, 54.8 mg) and di-*tert*-butylpyridine (1.0 equiv., 0.3 mmol, 57.4 mg). The vial was then capped, removed from the glovebox, and placed on a preheated block with stirring at 150 °C for 16 h. After the time, the reaction was taken off the heating block and allowed to cool to room temperature. Then methanol (0.2 mL) was added, followed by dodecane (0.3 mmol, 68.1 µL).[a] A crude aliquot was then filtered through a silica plug rinsing with dichloromethane and analyzed by GC-FID.

Notes: [a]Due to solids forming at the top of the vial, the vial was vigorously shaken prior to opening after the reaction.

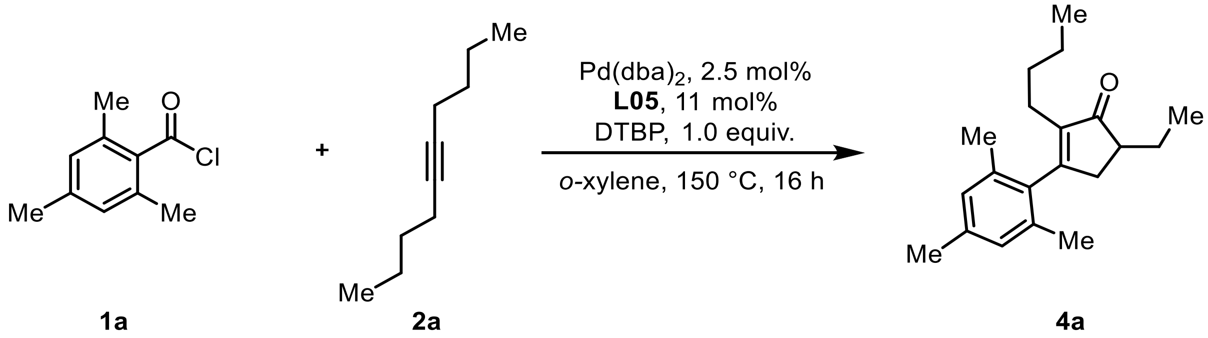

Entry	Concentration	4a %
1	3.0 M (0.1 mL)	43
2	1.5 M (0.2 mL)	51
**3**	**0.6 M (0.5 mL)**	**63**
4	0.3 M (1.0 mL)	58
5	0.15 M (2.0 mL)	41

General procedure for base screening

In a glovebox, a 4 mL screw-cap vial equipped with a stirring bar was charged with $Pd(dba)_2$ (5 mol%, 7.5 µmol, 8.6 mg) and the 3,5-CF_3DPEPhos (11 mol%, 0.033 mmol, 37.0 mg) and then *o*-xylene (0.5 mL). The solution was stirred at room temperature for 10 min. To a pre-mixed solution were added 5-decyne (1.5 equiv., 0.45 mmol, 62.2 mg), followed sequentially by mesitoyl chloride (1.0 equiv., 0.30 mmol, 54.8 mg) and base (1.0 equiv., 0.3 mmol). The vial was then capped, removed from the glovebox, and placed on a preheated block with stirring at 150 °C for 16 h. After the time, the reaction was taken off the heating block and allowed to cool to room temperature. Then methanol (0.2 mL) was added, followed by dodecane (0.3 mmol, 68.1 µL).[a] A crude aliquot was then filtered through a silica plug rinsing with dichloromethane and analyzed by GC-FID.

Notes: [a]Due to solids forming at the top of the vial, the vial was vigorously shaken prior to opening after the reaction.

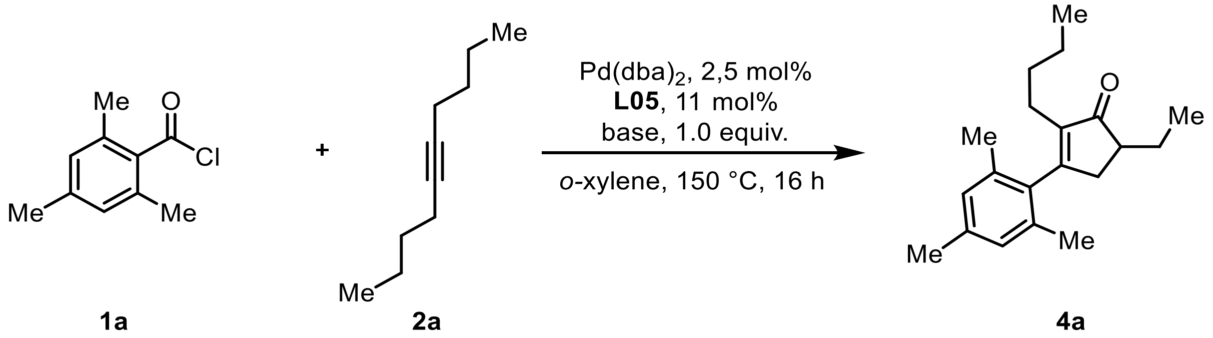

Entry	Base	4a %
**1**	**DTBP**	**63**
2	DIPEA	59
3	K_3PO_4	2
4	Na_2CO_3	20
5	LiOAc	12
6	2,6-lutidine	62

General procedure for base equivalent screening

In a glovebox, a 4 mL screw-cap vial equipped with a stirring bar was charged with $Pd(dba)_2$ (5 mol%, 7.5 µmol, 8.6 mg) and the 3,5-CF_3DPEPhos (11 mol%, 0.033 mmol, 37.0 mg) and then *o*-xylene (0.5 mL). The solution was stirred at room temperature for 10 min. To a pre-mixed solution were added 5-decyne (1.5 equiv., 0.45 mmol, 62.2 mg), followed sequentially by mesitoyl chloride (1.0 equiv., 0.30 mmol, 54.8 mg) and di-*tert*-butylpyridine (X equiv.). The vial was then capped, removed from the glovebox, and placed on a preheated block with stirring at 150 °C for 16 h. After the time, the reaction was taken off the heating block and allowed to cool to room temperature. Then methanol (0.2 mL) was added, followed by dodecane (0.3 mmol, 68.1 µL).[a] A crude aliquot was then filtered through a silica plug rinsing with dichloromethane and analyzed by GC-FID.

Notes: [a]Due to solids forming at the top of the vial, the vial was vigorously shaken prior to opening after the reaction.

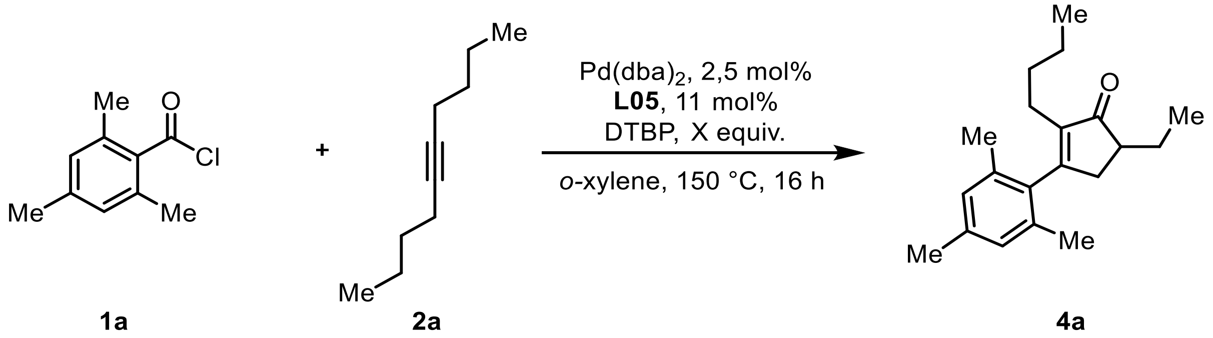

Entry	Base equiv	4a %
1	0	37
2	0.5	54
3	1.0	63
**4**	**1.2**	**65**
5	1.5	63
6	2.0	64

General procedure for temperature screening

In a glovebox, a 4 mL screw-cap vial equipped with a stirring bar was charged with Pd(dba)2 (5 mol%, 7.5 µmol, 8.6 mg) and the 3,5-CF_3DPEPhos (11 mol%, 0.033 mmol, 37.0 mg) and then *o*-xylene (0.5 mL). The solution was stirred at room temperature for 10 min. To a pre-mixed solution were added 5-decyne (1.5 equiv., 0.45 mmol, 62.2 mg), followed sequentially by mesitoyl chloride (1.0 equiv., 0.30 mmol, 54.8 mg) and di-*tert*-butylpyridine (1.2 equiv., 0.36 mmol, 68.9 mg). The vial was then capped, removed from the glovebox, and placed on a preheated block with stirring at the stated temperature for 16 h. After the time, the reaction was taken off the heating block and allowed to cool to room temperature. Then methanol (0.2 mL) was added, followed by dodecane (0.3 mmol, 68.1 µL).[a] A crude aliquot was then filtered through a silica plug rinsing with dichloromethane and analyzed by GC-FID.

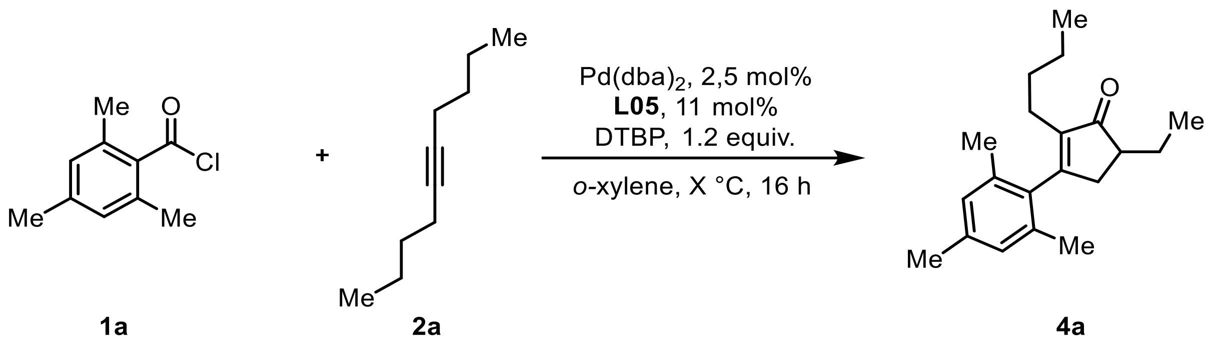

Entry	Temperature (°C)	4a %
1	160	71
**2**	**150**	**72**
3	140	40
4	130	23
5	120	6
6	110	3
7	100	1

General procedure for time screening

In a glovebox, a 4 mL screw-cap vial equipped with a stirring bar was charged with $Pd(dba)_2$ (5 mol%, 7.5 μmol, 8.6 mg) and the 3,5-CF_3DPEPhos (11 mol%, 0.033 mmol, 37.0 mg) and then *o*-xylene (0.5 mL). The solution was stirred at room temperature for 10 min. To a pre-mixed solution were added 5-decyne (1.5 equiv., 0.45 mmol, 62.2 mg), followed sequentially by mesitoyl chloride (1.0 equiv., 0.30 mmol, 54.8 mg) and di-*tert*-butylpyridine (1.2 equiv., 0.36 mmol, 68.9 mg). The vial was then capped, removed from the glovebox, and placed on a preheated block with stirring at 150 °C for the stated time. After the time, the reaction was taken off the heating block and allowed to cool to room temperature. Then methanol (0.2 mL) was added, followed by dodecane (0.3 mmol, 68.1 μL).[a] A crude aliquot was then filtered through a silica plug rinsing with dichloromethane and analyzed by GC-FID.

Notes: [a]Due to solids forming at the top of the vial, the vial was vigorously shaken prior to opening after the reaction.

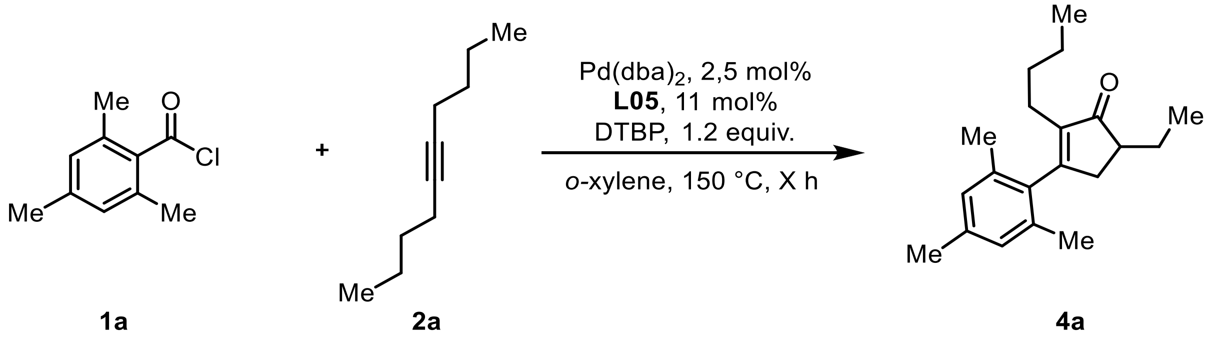

Entry	Time (h)	4a %
1	1	4
2	2	10
3	4	32
4	8	56
5	16	72
**6**	**24**	**78**

Extended Scope

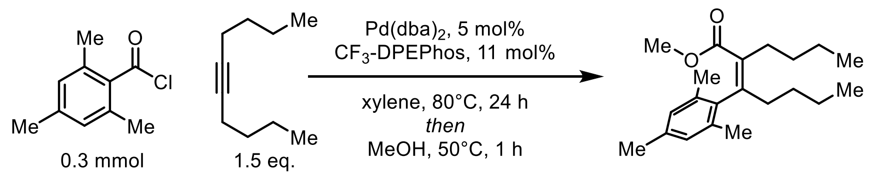

Successful examples:

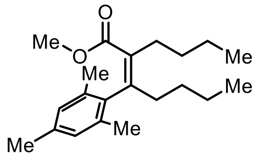

3a 63 %

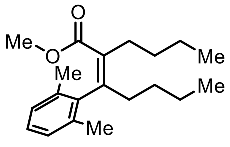

S3a 75 %

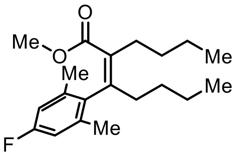

3c 93 %

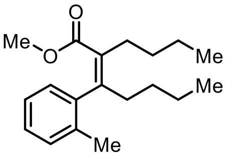

3b 52%

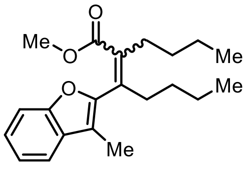

3g 56 % (E:Z 1:3)

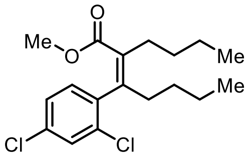

3f 43%*

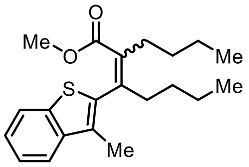

3h 32 % (E:Z 1:4)

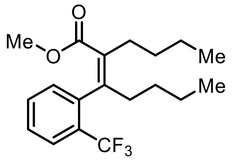

3d 28%*

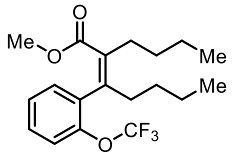

3e 29%*

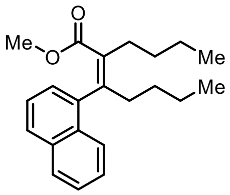

3i 24%

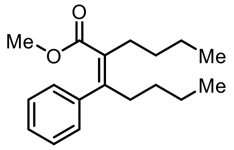

S3b 9%

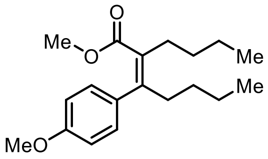

S3c 0%

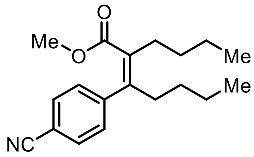

S3d 0%

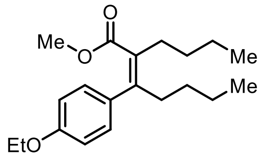

S3e 0%

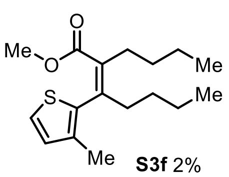

S3f 2%

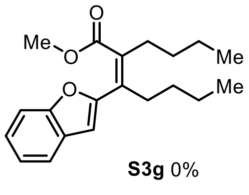

S3g 0%

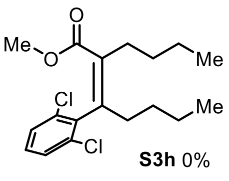

S3h 0%

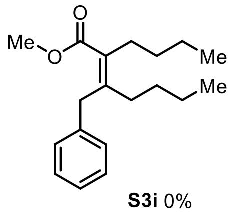

S3i 0%

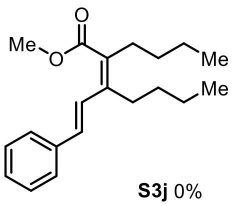

S3j 0%

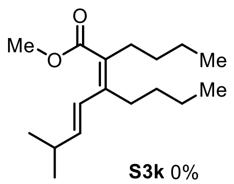

S3k 0%

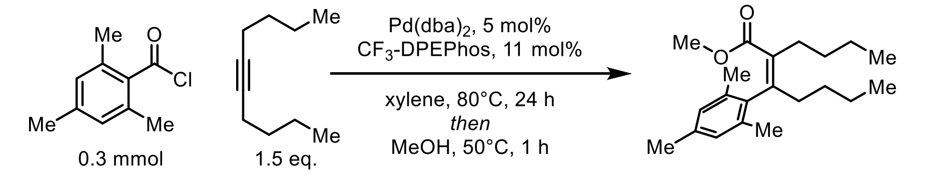

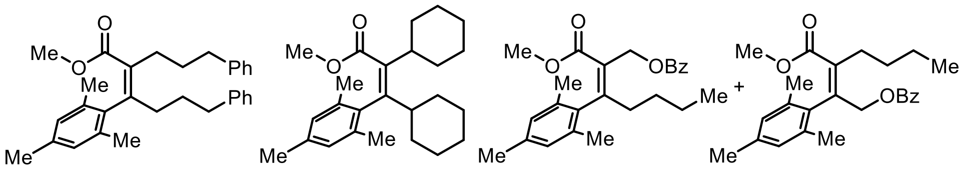

3j 77% **3k** 38 % **3l** 59% (1:1)

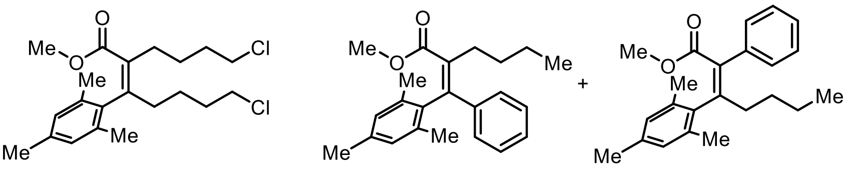

S3l 51% **3m, 3m'** 57 % (1:3)

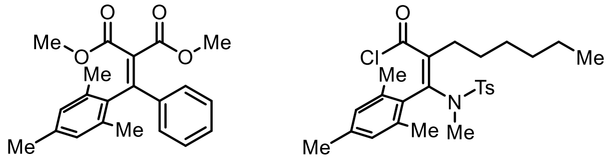

3n 37% **3o**: 94%

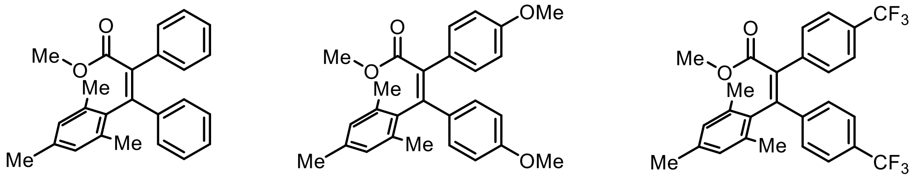

3p 60% **3q** 35% **3r** 38%

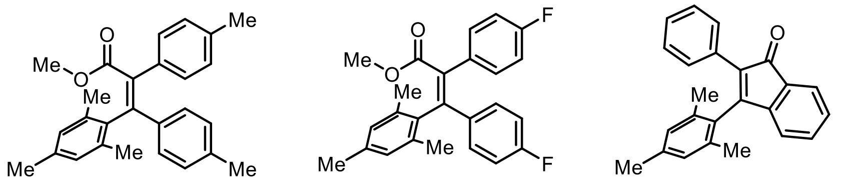

S3m 36% **S3n** 43% **3s** 56 %*

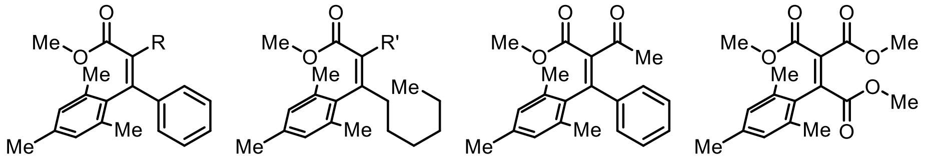

R = H, **S3o**
Bpin, **S3p**
TMS, **S3q**
allyl **S3r**
0%

R' = H. **S3t**
TIPS, **S3u**
0%

S3v 0%

S3w 0%

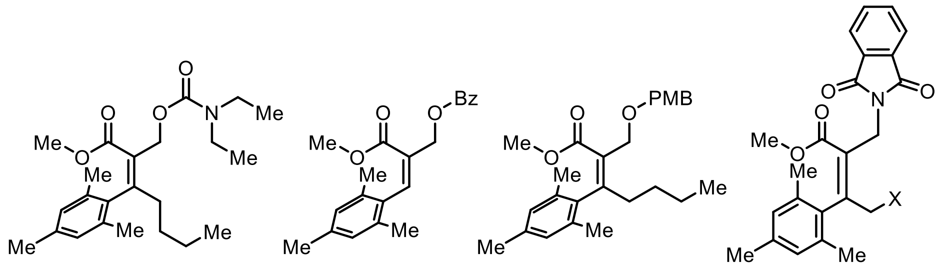

S3x
0%

S3y
0%

S3z
0%

X = Cl: **S3aa**, 0%
X = H: **S3ab**, 0%

Carbochlorocarbonylation

General procedure A1

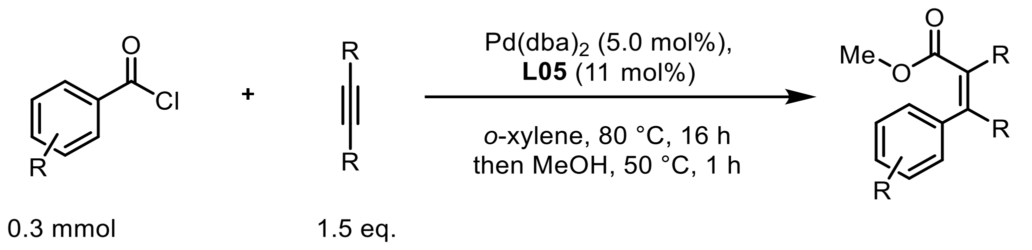

In a glovebox, to an oven dried 4 mL vial with a stirrer bar is added Pd(dba)$_2$ (8.60 mg, 15.0 μmol, 5.00 mol%), followed by **L05** (35.7 mg, 33.0 μmol, 11.0 mol%), and *o*-xylene (0.5 mL). The mixture is stirred for 10 min before addition of the corresponding alkyne (0.45 mmol, 1.50 equiv), acid chloride (0.30 mmol, 1.00 eq.), and 2,6-ditertbutyl pyridine (68.9 mg, 0.36 mmol,). The vial is then capped and removed from the glovebox and placed in a pre-heated stirring block at 80 °C 1200 rpm for 16 h. After the reaction has cooled, methanol (0.2 mL) is added and stirred for 10 min. The solution is then filtered through a plug of silica and rinsed with EtOAc and concentrated under reduced pressure. Subsequently purification is achieved by means of column chromatography or preparative TLC.

Methyl (Z)-2-butyl-3-mesitylhept-2-enoate (3a)

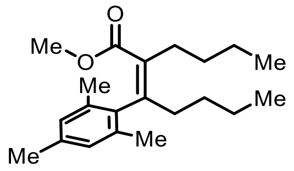

The title compound was synthesized according to general procedure **A1** from 2,4,6-trimethylbenzoylchloride **1a** and 5-decyne **2a**. Purification by column chromatography (SiO$_2$, 5% EtOAc in pentane) afforded **3a** as a yellow oil (60.1 mg, 0.19 mmol, 63%).

^{1}H NMR (400 MHz, CDCl$_3$) δ 6.80 (h, *J* = 0.6 Hz, 2H), 3.36 (s, 3H), 2.47 (t, *J* = 8.2 Hz, 2H), 2.33 (t, *J* = 8.1 Hz, 2H), 2.25 (s, 3H), 2.12 (s, 6H), 1.55 – 1.34 (m, 4H), 1.37 – 1.22 (m, 4H), 0.95 (t, *J* = 6.8 Hz, 3H), 0.87 (t, *J* = 6.8 Hz, 3H) ppm.

^{13}C{^{1}H} (101 MHz, CDCl$_3$) δ 170.2, 145.7, 138.9, 135.6, 134.7, 132.0, 128.0, 51.2, 34.8, 31.4, 30.0, 29.3, 23.5, 22.8, 21.2, 20.1, 14.1, 14.0 ppm.

HRMS (ESI, m/z): $[M+Na]^+$ calcd. for $C_{21}H_{32}NaO_2$, 339.2295; found 339.2291.

Note: The stereochemistry of the product was determined by XRD and 2D-NMR analysis.

Methyl (Z)-2-butyl-3-(o-tolyl)hept-2-enoate (3b)

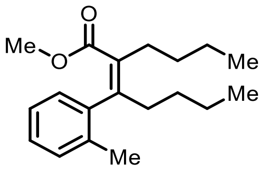

The title compound was synthesized according to general procedure **A1** from 2-methylbenzoylchloride **1b** and 5-decyne **2a**. Purification by preparative TLC (SiO_2, 5% EtOAc in hexane) afforded **3b** as a yellow oil (48.8 mg, 156 µmol, 52%).

^{1}H NMR (400 MHz, $CDCl_3$) δ 7.17 – 7.12 (m, 2H), 7.12 – 7.05 (m, 1H), 6.90 (d, J = 7.0 Hz, 1H), 3.30 (s, 3H), 2.55 – 2.49 (m, 1H), 2.46 (t, J = 7.4 Hz, 2H), 2.21 (s, 3H), 2.18 (m, 1H), 1.52 – 1.21 (m, 8H), 0.95 (t, J = 7.0 Hz, 3H), 0.86 (t, J = 6.8, 5.9 Hz, 3H) ppm.

^{13}C{^{1}H} (101 MHz, $CDCl_3$) δ 170.4, 147.0, 142.4, 135.0, 131.8, 129.7, 127.9, 126.8, 125.1, 51.1, 34.2, 31.4, 29.8, 29.2, 23.1, 22.7, 19.6, 14.1, 14.0 ppm.

HRMS (ESI, m/z): $[M+Na]^+$ calcd. for $C_{19}H_{28}NaO_2$, 311.1982; found 311.1979.

Note: The stereochemistry of the product was supported by 2D-NMR analysis.

Methyl (Z)-2-butyl-3-(4-fluoro-2,6-dimethylphenyl)hept-2-enoate (3c)

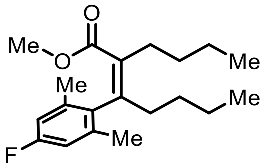

The title compound was synthesized according to general procedure **A1** from 4-fluoro-2,6-dimethylbenzoylchloride **1c** and 5-decyne **2a**. Purification by preparative TLC (SiO_2, 5% EtOAc in hexane) afforded **3c** as a yellow oil (87.0 mg, 275 µmol, 92%).

^{1}**H NMR** (400 MHz, $CDCl_3$) δ 6.69 (dt, *J* = 9.7, 0.5 Hz, 2H), 3.36 (s, 3H), 2.53 – 2.42 (m, 2H), 2.38 – 2.27 (m, 2H), 2.15 (d, *J* = 0.7 Hz, 6H), 1.54 – 1.36 (m, 4H), 1.34 – 1.22 (m, 4H), 0.95 (t, *J* = 7.1 Hz, 3H), 0.89 – 0.82 (m, 3H) ppm.

^{13}**C NMR** (101 MHz, $CDCl_3$) δ 170.0, 161.09 (d, *J* = 243.2 Hz), 144.3, 137.3 (d, *J* = 3.1 Hz), 137.1 (d, *J* = 8.0 Hz), 132.7, 113.6 (d, *J* = 20.6 Hz), 51.1, 34.4, 31.1, 29.8, 29.2, 23.3, 22.7, 20.2, 20.2, 14.0, 13.9 ppm.

^{19}**F{^{1}H}** (377 MHz, $CDCl_3$) δ – 118.0 ppm.

HRMS (ESI, m/z): $[M+Na]^+$ calcd. for $C_{20}H_{29}FNaO_2$, 343.2044; found 343.2042.

Note: The stereochemistry of the product was supported by 2D-NMR analysis.

Methyl (Z)-2-butyl-3-(2-(trifluoromethyl)phenyl)hept-2-enoate (3d)

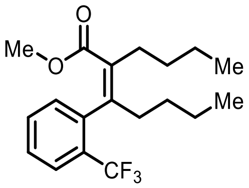

The title compound was synthesized according to general procedure **A1,** with a prolonged reaction time of 72 h from 2-trifluoromethyl-benzoyl chloride **1d** and 5-decyne **2a**. Purification by preparative TLC (SiO_2, 5% EtOAc in hexane) afforded **3d** as a colorless oil (28.9 mg, 84.1 μmol, 28%).

^{1}**H NMR** (400 MHz, $CDCl_3$) δ 7.63 (ddd, *J* = 7.4, 1.5, 0.8 Hz, 1H), 7.45 (tdd, *J* = 7.6, 1.5, 0.7 Hz, 1H), 7.36 (tt, *J* = 7.6, 1.1 Hz, 1H), 7.04 (ddt, *J* = 7.7, 1.4, 0.7 Hz, 1H), 3.33 (s, 3H), 2.70 – 2.58 (m, 1H), 2.51 – 2.38 (m, 2H), 2.14 (ddt, *J* = 15.3, 9.9, 4.6 Hz, 1H), 1.50 – 1.20 (m, 8H), 0.95 (t, *J* = 7.1 Hz, 3H), 0.90 – 0.84 (m, 3H) ppm.

^{13}**C{^{1}H}** (126 MHz, $CDCl_3$) δ 169.1, 147.2, 141.9 (q, J = 2.1 Hz), 131.9 (q, J = 1.4 Hz), 131.5 (q, J = 27.8 Hz), 131.0 (q, J = 1.2 Hz), 129.7, 126.9, 126.5 (q, J = 5.0 Hz), 124.5 (q, J = 273.9 Hz), 35.1 (q, J = 2.0 Hz), 31.2, 29.9, 29.1, 23.1, 22.8, 14.1, 14.0 ppm.

^{19}**F{^{1}H}** (377 MHz, $CDCl_3$) δ -58.8 ppm.

HRMS (ESI, m/z): $[M+Na]^+$ calcd. for $C_{19}H_{25}F_3NaO_2$, 365.1699; found 365.1699.

Note: The stereochemistry of the product was supported by 2D-NMR analysis.

Methyl (Z)-2-butyl-3-(2-(trifluoromethoxy)phenyl)hept-2-enoate (3e)

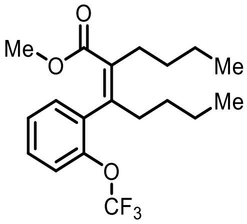

The title compound was synthesized according to general procedure **A1** from 2-(trifluoromethoxy)benzoylchloride **1e** and 5-decyne **2a**, with a prolonged reaction time of 72 h. Purification by preparative TLC (SiO_2, 5% EtOAc in hexane) afforded **3e** as a yellow oil (32.2 mg, 87 µmol, 29%).

^{1}H NMR (500 MHz $CDCl_3$) δ) δ 7.29 – 7.25 (m, 1H), 7.24 – 7.20 (m, 1H), 7.18 (td, J = 7.3, 1.4 Hz, 1H), 7.06 (ddd, J = 7.6, 1.7, 0.4 Hz, 1H), 3.34 (s, 2H), 2.47 (t, J = 7.3 Hz, 1H), 2.36 – 2.25 (m, 1H), 1.50 – 1.34 (m, 4H), 1.30 – 1.20 (m, 6H), 0.94 (t, J = 7.1 Hz, 3H), 0.85 (dd, J = 7.7, 6.3 Hz, 3H) ppm.

^{13}C{^{1}H} (126 MHz, $CDCl_3$) δ 169.8, 146.2 (q, J = 1.5 Hz), 143.3, 135.5, 133.4, 130.3, 128.3, 125.9, 123.8, 121.7, 120.7 (q, J = 257.8 Hz), 119.4 (q, J = 1.8 Hz), 51.2, 33.7, 31.3, 29.9, 29.4, 22.9, 22.6, 14.1, 14.0 ppm.

^{19}F{^{1}H} NMR (377 MHz, $CDCl_3$) δ – 56.4 ppm.

HRMS (ESI, m/z): $[M+Na]^+$ calcd. for $C_{19}H_{25}F_3NaO_3$, 381.1648; found 381.1641.

Note: The stereochemistry of the product was supported by 2D-NMR analysis.

methyl (Z)-2-butyl-3-(2,4-dichlorophenyl)hept-2-enoate (3f)

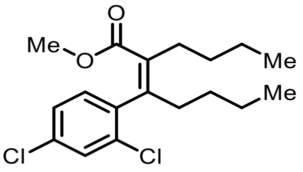

The title compound was synthesized according to general procedure **A1** from 2,4-dichlorobenzoyl chloride **1f** and 5-decyne **2a** with a prolonged reaction time of 72 h.

Purification by preparative TLC (SiO_2, 5% EtOAc in hexane) afforded **3f** as a colorless oil (44.4 mg, 129 µmol, 43%).

^{1}H NMR (400 MHz, $CDCl_3$) δ 7.37 (d, J = 2.1 Hz, 1H), 7.17 (dd, J = 8.2, 2.1 Hz, 1H), 6.94 (d, J = 8.2 Hz, 1H), 3.41 (s, 3H), 2.56 – 2.38 (m, 3H), 2.38 – 2.24 (m, 1H), 1.50 – 1.35 (m, 4H), 1.27 (ddq, J = 12.4, 9.7, 6.7, 5.6 Hz, 4H), 0.94 (t, J = 7.6, 6.7 Hz, 3H), 0.86 (t, J = 7.3, 6.8 Hz, 3H) ppm.

^{13}C{^{1}H} (101 MHz, $CDCl_3$) δ 169.3, 145.0, 140.3, 139.2, 133.2, 133.1, 130.3, 129.2, 126.6, 51.5, 33.8, 31.3, 29.7, 29.2, 23.0, 22.7, 14.1, 14.0 ppm.

HRMS (ESI, m/z): $[M+H]^+$ calcd. for $C_{18}H_{25}Cl_2O_2$, 343.1226; found 343.1224.

Note: The stereochemistry of the product was supported by 2D-NMR analysis.

Methyl (Z/E)-2-butyl-3-(3-methylbenzofuran-2-yl)hept-2-enoate (Z-3g and E-3g)

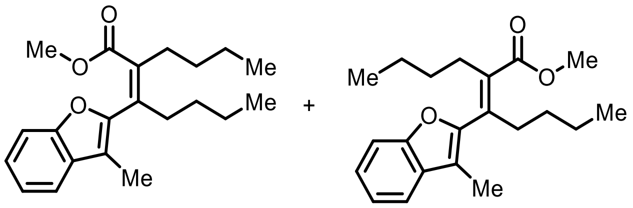

The title compounds were synthesized according to general procedure **A1** from 3-methylbenzofuran-2-carbonyl chloride **1g** and 5-decyne **2a**. Purification by preparative TLC (SiO_2, 5% EtOAc in hexane) afforded the two isomers **Z-3g** and **E-3g** as colorless oils as separate isomers in a 0.3:1 ratio (**Z**-isomer: 14.0 mg, 42 µmol, 14%; **E-**isomer: 42.1 mg, 126 µmol, 42%; *combined yield of 56%*).

Methyl (Z)-2-butyl-3-(3-methylbenzofuran-2-yl)hept-2-enoate (Z-3g)

^{1}H NMR (400 MHz, $CDCl_3$) δ 7.50 (ddd, J = 7.1, 1.7, 0.7 Hz, 1H), 7.43 (ddd, J = 8.0, 1.4, 0.7 Hz, 1H), 7.31 – 7.26 (m, 1H), 7.26 – 7.22 (m, 1H), 3.82 (s, 3H), 2.53 (t, J = 7.4 Hz, 2H), 2.28 – 2.22 (m, 2H), 2.15 (s, 3H), 1.38 – 1.25 (m, 6H), 1.25 – 1.13 (m, 2H), 0.85 – 0.79 (m, 3H), 0.77 (t, J = 7.3 Hz, 3H) ppm.

^{13}C{^{1}H} (101 MHz, $CDCl_3$) δ 170.2, 154.4, 150.9, 136.5, 135.0, 130.0, 124.2, 122.4, 119.4, 112.9, 111.2, 51.7, 33.7, 31.7, 30.7, 30.7, 22.6, 22.5, 14.0, 13.9, 8.9 ppm.

HRMS (ESI, m/z): $[M+Na]^+$ calcd. for $C_{21}H_{28}NaO_3$, 351.1931; found 351.1932.

Note: The stereochemistry of the product was supported by 2D-NMR analysis.

Methyl (E)-2-butyl-3-(3-methylbenzofuran-2-yl)hept-2-enoate (E-3g)

^{1}H NMR (400 MHz, $CDCl_3$) δ 7.45 (ddd, *J* = 7.3, 1.6, 0.7 Hz, 1H), 7.41 (ddd, *J* = 8.0, 1.2, 0.7 Hz, 1H), 7.29 – 7.17 (m, 2H), 3.46 (s, 3H), 2.51 (t, *J* = 7.5 Hz, 4H), 1.54 – 1.35 (m, 4H), 1.32 – 1.22 (m, 4H), 0.96 (t, *J* = 7.2 Hz, 3H), 0.88 – 0.82 (m, 3H) ppm.

^{13}C{^{1}H} (101 MHz, $CDCl_3$) δ 170.8, 154.2, 151.6, 137.1, 135.3, 130.2, 124.2, 122.3, 119.4, 112.9, 111.1, 51.9, 31.6, 31.2, 30.4, 30.1, 22.8, 22.7, 14.1, 14.0, 8.8 ppm.

HRMS (ESI, m/z): $[M+Na]^+$ calcd. for $C_{21}H_{28}NaO_3$, 351.1931; found 351.1922.

Note: The stereochemistry of the product was supported by 2D-NMR analysis.

Methyl (Z/E)-2-butyl-3-(3-methylbenzo[b]thiophen-2-yl)hept-2-enoate (Z-3h and E-3h)

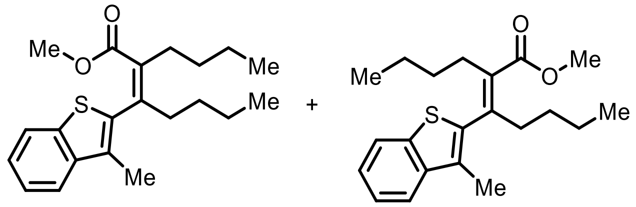

The title compounds were synthesized according to general procedure **A1** from 3-methylbenzo[b]thiohene-2-carbonyl chloride **1h** and 5-decyne **2a**. Purification by preparative TLC (SiO_2, 5% EtOAc in hexane) afforded the two isomers **Z-3h** and **E-3h** as colorless oils as separate isomers in a 0.25:1 ratio (**Z**-isomer: 7.5 mg, 42 μmol, 6%; **E-**isomer: 28.0 mg, 203 μmol, 26%; *combined yield of 32%*).

Methyl (Z)-2-butyl-3-(3-methylbenzo[b]thiophen-2-yl)hept-2-enoate (Z-3h)

^{1}H NMR (500 MHz, $CDCl_3$) δ 7.76 (ddd, J = 7.9, 1.2, 0.7 Hz, 1H), 7.62 (ddd, J = 8.0, 1.3, 0.7 Hz, 1H), 7.35 (ddd, J = 8.1, 7.1, 1.2 Hz, 1H), 7.30 (ddd, J = 7.9, 7.1, 1.3 Hz, 1H), 3.38 (s, 3H), 2.53 – 2.47 (m, 2H), 2.47 – 2.41 (m, 2H), 2.21 (s, 3H), 1.48 (dddd, J = 11.9, 10.6, 5.7, 2.3 Hz, 2H), 1.44 – 1.38 (m, 2H), 1.38 – 1.28 (m, 4H), 0.95 (t, J = 7.2 Hz, 3H), 0.86 (t, J = 7.3, 6.8 Hz, 3H) ppm.

^{13}C{^{1}H} (126 MHz, $CDCl_3$) δ 170.5, 140.3, 139.4, 138.6, 138.3, 136.5, 128.4, 124.1, 123.9, 122.3, 122.0, 51.7, 34.9, 31.3, 30.2, 30.1, 22.9, 22.8, 14.1, 14.0, 12.6 ppm.

HRMS (ESI, m/z): $[M+Na]^+$ calcd. for $C_{21}H_{28}NaO_2S$, 367.1702; found 367.1700.

Note: The stereochemistry of the product was supported by 2D-NMR analysis.

Methyl (E)-2-butyl-3-(3-methylbenzo[b]thiophen-2-yl)hept-2-enoate (E-3h)

^{1}H NMR (500 MHz, $CDCl_3$) δ 7.80 (ddd, J = 7.9, 1.1, 0.7 Hz, 1H), 7.67 (ddd, J = 8.0, 1.3, 0.7 Hz, 1H), 7.39 (ddd, J = 8.0, 7.1, 1.2 Hz, 1H), 7.33 (ddd, J = 7.9, 7.1, 1.3 Hz, 1H), 3.82 (s, 3H), 2.52 – 2.45 (m, 2H), 2.26 (s, 3H), 2.20 – 2.15 (m, 2H), 1.45 – 1.21 (m, 6H), 1.21 – 1.10 (m, 2H), 0.83 (t, J = 7.3 Hz, 3H), 0.76 (t, J = 7.3 Hz, 3H) ppm.

^{13}C{^{1}H} (126 MHz, $CDCl_3$) δ 170.3, 140.3, 139.5, 138.7, 137.2, 135.7, 128.2, 124.3, 124.1, 122.4, 122.0, 51.7, 36.6, 31.5, 30.6, 30.5, 29.9, 22.8, 22.6, 14.0, 12.7 ppm.

HRMS (ESI, m/z): $[M+Na]^+$ calcd. for $C_{21}H_{28}NaO_2S$, 367.1702; found 367.1701.

Note: The stereochemistry of the product was supported by 2D-NMR analysis.

Methyl (Z)-2-butyl-3-(naphthalen-1-yl)hept-2-enoate (3i)

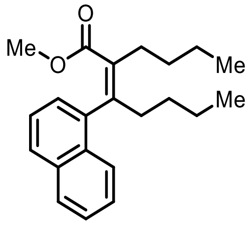

The title compound was synthesized according to general procedure **A1** from 1-(chloro-carbonyl)naphthalene **1i** and 5-decyne **2a**. Purification by preparative TLC (SiO_2, 5% EtOAc in hexane) afforded **3i** as a colorless oil (27.6 mg, 720 µmol, 24%).

^{1}H NMR (300 MHz, $CDCl_3$) δ 7.86 – 7.76 (m, 2H), 7.73 (dt, *J* = 8.4, 1.1 Hz, 1H), 7.46 (dt, *J* = 6.3, 3.4 Hz, 2H), 7.39 (dd, *J* = 8.2, 7.0 Hz, 1H), 7.12 (dd, *J* = 7.0, 1.2 Hz, 1H), 3.08 (s, 3H), 2.71 – 2.62 (m, 1H), 2.58 (dd, *J* = 8.5, 6.5 Hz, 2H), 2.46 – 2.32 (m, 1H), 1.63 – 1.39 (m, 4H), 1.39 – 1.19 (m, 4H), 0.99 (t, *J* = 7.1 Hz, 3H), 0.86 – 0.75 (m, 3H) ppm.

^{13}**C{^{1}H}** (101 MHz, $CDCl_3$) δ 170.3, 146.0, 140.8, 133.6, 133.4, 131.3, 128.4, 127.2, 125.9, 125.7, 125.7, 125.1, 124.8, 51.1, 34.9, 31.6, 30.4, 29.5, 23.0, 22.9, 14.2, 14.0 ppm.

HRMS (ESI, m/z): $[M+Na]^+$ calcd. for $C_{22}H_{28}NaO_2$, 347.1982; found 347.1973.

Note: The stereochemistry of the product was supported by 2D-NMR analysis.

Methyl (Z)-3-mesityl-6-phenyl-2-(3-phenylpropyl)hex-2-enoate (3j)

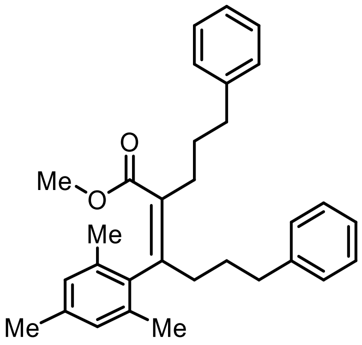

The title compound was synthesized according to general procedure **A1** from 2,4,6-trimethylbenzoylchloride **1a** and 1,8-diphenyloct-4-yne **2j**. Purification by preparative TLC (SiO_2, 5% EtOAc in hexane) afforded **3j** as a colorless oil (105 mg, 231 μmol, 77%)

^{1}**H NMR** (400 MHz, $CDCl_3$) δ 7.34 – 7.23 (m, 4H), 7.23 – 7.14 (m, 4H), 7.13 – 7.06 (m, 2H), 6.79 (s, 2H), 3.34 (s, 3H), 2.62 (t, J = 7.7 Hz, 2H), 2.51 (t, J = 7.6 Hz, 2H), 2.45 – 2.36 (m, 2H), 2.32 – 2.26 (m, 2H), 2.24 (s, 3H), 2.10 (s, 6H), 1.82 – 1.71 (m, 2H), 1.67 – 1.60 (m, 2H) ppm.

^{13}**C{^{1}H}** (101 MHz, $CDCl_3$) δ 169.8, 146.1, 142.3, 142.0, 138.6, 135.8, 134.6, 131.8, 128.6, 128.5, 128.5, 128.5, 128.1, 126.0, 126.0, 51.3, 36.5, 35.9, 34.6, 31.0, 29.5, 29.2, 21.2, 20.2 ppm.

HRMS (ESI, m/z): $[M+H]^+$ calcd. for $C_{31}H_{37}O_2$, 441.2788; found 441.2784.

Note: The stereochemistry of the product was supported by 2D-NMR analysis.

Methyl (Z)-2,3-dicyclohexyl-3-mesitylacrylate (3k)

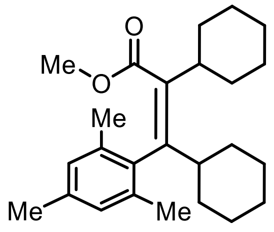

The title compound was synthesized according to general procedure **A1** from 2,4,6-trimethylbenzoyl chloride **1a** and 1,2-dicyclohexylethyne **2k**. Purification preparative TLC (SiO_2, 5% EtOAc in hexane) afforded **3k** as a colorless oil (42.4 mg, 114 µmol, 38%)

^{1}H NMR (400 MHz, $CDCl_3$) δ 6.75 (s, 2H), 3.22 (s, 3H), 2.69 – 2.53 (m, 2H), 2.21 (s, 3H), 2.17 (s, 6H), 1.84 – 1.77 (m, 4H), 1.76 – 1.65 (m, 6H), 1.45 – 1.22 (m, 6H), 1.22 – 0.97 (m, 4H) ppm.

^{13}C{^{1}H} (101 MHz, $CDCl_3$) δ 170.5, 142.3, 137.9, 136.6, 136.2, 135.7, 127.9, 50.6, 41.7, 38.9, 32.6, 31.7, 27.1, 26.9, 26.2, 26.0, 21.3, 21.1 ppm.

HRMS (ESI, m/z): $[M+Na]^+$ calcd. for for $C_{25}H_{36}NaO_2$, 391.2608; found 391.2599.

Note: The stereochemistry of the product was supported by 2D-NMR analysis.

(Z)-3-mesityl-2-(methoxycarbonyl)hept-2-en-1-yl benzoate (3l) and (E)-2-mesityl-3-(methoxycarbonyl)hept-2-en-1-yl benzoate (3l')

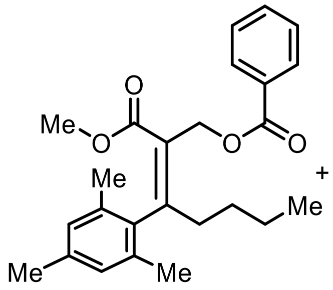

3l

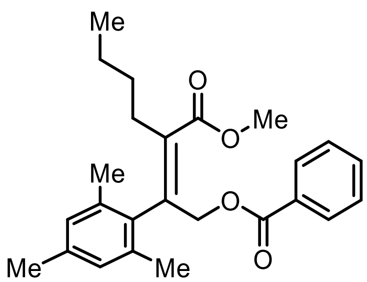

3l'

The title compounds were synthesized according to general procedure **A1** from 2,4,6-trimethylbenzoyl chloride **1a** and hept-2-yn-1-yl benzoate **2l**. Purification by preparative TLC (SiO_2, 5% EtOAc in hexane) afforded two different regioisomers (**3l, 3l'**), in a ratio 1:1. **3l** was isolated as a brown oil (32.5 mg, 87 µmol, 29%), and **3l'** as a colorless oil (35.2 mg, 90 µmol, 30%); *combined yield of 59%.*

(Z)-3-mesityl-2-(methoxycarbonyl)hept-2-en-1-yl benzoate (3l)

^{1}H NMR (400 MHz, $CDCl_3$) δ 7.87 – 7.81 (m, 2H), 7.52 (tt, J = 7.4, 1.2 Hz, 1H), 7.38 (tt, J = 7.6, 1.3 Hz, 2H), 6.80 (d, J = 1.2 Hz, 2H), 5.09 (s, 2H), 3.41 (s, 3H), 2.57 (t, J = 7.8 Hz, 2H), 2.23 (s, 3H), 2.20 (s, 6H), 1.56 – 1.48 (m, 2H), 1.48 – 1.34 (m, 2H), 0.93 (t, J = 7.2 Hz, 3H).

^{13}C{^{1}H} (101 MHz, $CDCl_3$) δ 169.6, 166.5, 137.6, 137.4, 136.6, 135.5, 135.5, 133.1, 130.0, 129.7, 128.5, 128.1, 64.0, 51.5, 31.3, 29.7, 22.8, 21.2, 20.1, 14.1.

HRMS (ESI, m/z): $[M+Na]^+$ calcd. for $C_{25}H_{30}NaO_4$, 417.2036; found 417.2024.

Note: The stereochemistry of the product was supported by 2D-NMR analysis.

(E)-2-mesityl-3-(methoxycarbonyl)hept-2-en-1-yl benzoate (3l')

^{1}H NMR (400 MHz, $CDCl_3$) δ 8.09 – 8.00 (m, 2H), 7.56 (tt, J = 7.4, 1.4 Hz, 1H), 7.44 (tt, J = 7.4, 1.6 Hz, 2H), 6.83 (s, 2H), 5.27 (s, 2H), 3.45 (s, 3H), 2.57 – 2.49 (m, 2H), 2.27 (s, 3H), 2.14 (s, 6H), 1.43 – 1.22 (m, 4H), 0.84 (t, J = 7.1 Hz, 3H).

^{13}C{^{1}H} (101 MHz, $CDCl_3$) δ 167.7, 166.6, 155.7, 138.0, 136.3, 133.9, 133.2, 130.3, 129.8, 128.6, 128.3, 126.4, 61.5, 51.7, 35.9, 30.4, 23.4, 21.2, 20.0, 14.0.

HRMS (ESI, m/z): $[M+Na]^+$ calcd. for $C_{25}H_{30}NaO_4$, 417.2036; found 417.2025.

Note: The stereochemistry of the product was supported by 2D-NMR analysis.

Methyl (Z)-2-(mesityl(phenyl)methylene)hexanoate (3m) and Methyl (Z)-3-mesityl-2-phenylhept-2-enoate (3m')

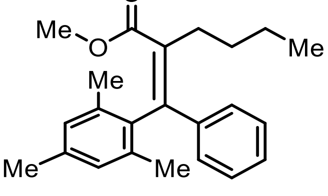

3m

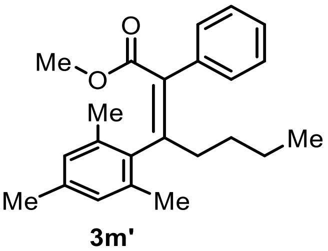

3m'

The title compounds were synthesized according to general procedure **A1** from 2,4,6-trimethylbenzoyl chloride **1a** and hept-2-yn-1-yl benzoate **ml**. Purification by preparative TLC (SiO_2, 5% EtOAc in hexane) afforded two different regioisomers (**3m, 3m'**), in a ratio 4.4:1.

3m as a colorless oil (58.0 mg, 171 μmol, 57%), and **3m'** as an orange oil (13.7 mg, 38 μmol, 13%).*combined yield of 70%.*

Methyl (Z)-2-(mesityl(phenyl)methylene)hexanoate (3m)

^{1}H NMR (400 MHz, $CDCl_3$) δ 7.32 – 7.15 (m, 5H), 6.80 (dd, *J* = 1.4, 0.7 Hz, 2H), 3.39 (s, 3H), 2.61 – 2.50 (m, 2H), 2.24 (s, 3H), 2.16 (s, 6H), 1.52 (ddt, *J* = 10.5, 7.9, 6.1 Hz, 4H), 1.36 (dp, *J* = 8.9, 7.3 Hz, 2H), 0.90 (t, *J* = 7.3 Hz, 3H) ppm.

^{13}C{^{1}H} (101 MHz, $CDCl_3$) δ 171.0, 143.3, 139.2, 138.7, 136.5, 135.6, 134.7, 129.2, 128.2, 128.1, 127.6, 51.4, 31.5, 30.8, 22.9, 21.2, 20.3, 14.0 ppm.

HRMS (ESI, m/z): $[M+Na]^+$ calcd. for for $C_{23}H_{28}NaO_2$, 359.1982; found 359.1974.

Note: The stereochemistry of the product was supported by 2D-NMR analysis.

Methyl (Z)-3-mesityl-2-phenylhept-2-enoate (3m')

^{1}H NMR (400 MHz, $CDCl_3$) δ 7.45 – 7.32 (m, 5H), 6.85 (ddd, *J* = 3.5, 1.3, 0.7 Hz, 2H), 3.37 (s, 3H), 2.28 (s, 6H), 2.27 (s, 3H), 1.33 – 1.19 (m, 4H), 1.15 – 1.04 (m, 2H), 0.70 (t, *J* = 7.3 Hz, 3H) ppm.

^{13}C{^{1}H} (126 MHz, $CDCl_3$) δ 168.6, 149.1, 137.3, 136.2, 135.6, 134.8, 133.1, 129.3, 128.5, 128.3, 127.6, 51.7, 35.5, 30.0, 23.3, 21.2, 20.1, 13.8 ppm.

HRMS (ESI, m/z): $[M+Na]^+$ calcd. for $C_{23}H_{28}NaO_2$, 359.1982; found 359.1973.

Note: The stereochemistry of the product was supported by 2D-NMR analysis.

Dimethyl 2-(mesityl(phenyl)methylene)malonate (3n)

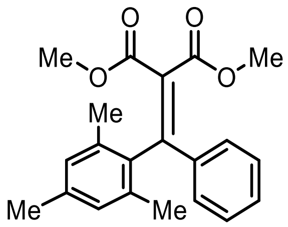

The title compound was synthesized according to general procedure **A1** from 2,4,6-trimethylbenzoyl chloride **1a** and methyl 3-phenylpropiolate **2n**. Purification by preparative TLC (SiO_2, 5% EtOAc in hexane) afforded **3n** as a colorless oil (37.2 mg, 111 µmol, 37%).

^{1}H NMR (400 MHz, $CDCl_3$) δ 7.34 – 7.27 (m, 3H), 7.23 – 7.18 (m, 2H), 6.86 (dt, J = 1.3, 0.7 Hz, 2H), 3.66 (s, 3H), 3.56 (s, 3H), 2.28 (d, J = 0.7 Hz, 3H), 2.14 (d, J = 0.6 Hz, 6H) ppm.

^{13}C{^{1}H} (101 MHz, $CDCl_3$) δ 167.4, 165.0, 155.6, 138.2, 137.7, 136.2, 134.8, 129.6, 128.6, 128.5, 128.4, 126.9, 52.5, 52.3, 21.3, 20.0 ppm.

HRMS (ESI, m/z): $[M+Na]^+$ calcd. for $C_{21}H_{22}NaO_4$, 361.141; found 361.1402.

Note: The stereochemistry of the product was supported by 2D-NMR analysis.

Methyl (E)-2-(((N,4-dimethylphenyl)sulfonamido)(mesityl)methylene)octanoate (3o)

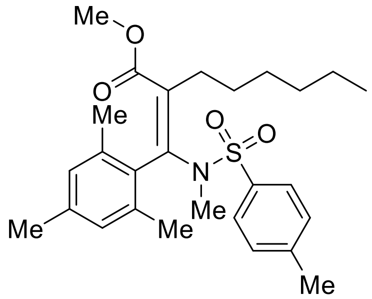

The title compound was synthesized according to general procedure **A1** from 2,4,6-trimethylbenzoylchloride **1a** and N,4-dimethyl-N-(oct-1-yn-1-yl)benzenesulfonamide. Purification by preparative TLC (SiO_2, 8% EtOAc in hexane) afforded **3o** as a yellow oil (133 mg, 282 µmol, 94%).

^{1}H NMR (500 MHz, $CDCl_3$) δ 7.06 – 6.85 (m, 4H), 6.65 (s, 2H), 3.34 (s, 3H), 3.28 (s, 3H), 2.56 – 2.48 (m, 2H), 2.36 (s, 3H), 2.25 (s, 3H), 2.01 (s, 6H), 1.58 – 1.49 (m, 2H), 1.44 – 1.36 (m, 2H), 1.35 – 1.28 (m, 4H), 0.96 – 0.85 (m, 4H).ppm.

^{13}C{^{1}H} (125 MHz, $CDCl_3$) δ 169.6, 145.0, 143.1, 138.4, 138.0, 136.0, 133.0, 132.2, 129.0, 128.5, 127.3, 51.6, 41.7, 31.6, 30.8, 29.9, 28.1, 22.8, 21.6, 21.2, 20.7, 14.2 ppm.

HRMS (ESI, m/z): $[M+Na]^+$ calcd. for $C_{27}H_{37}NNaO_4S$, 494.2336; found 494.2335.

Note: The stereochemistry of the product was supported by 2D-NMR analysis (in this case particularly NOESY spectra helped guide our assignment).

Methyl (Z)-3-mesityl-2,3-diphenylacrylate (3p)

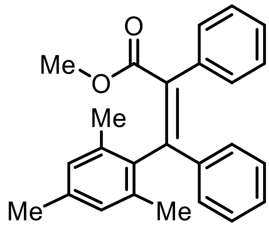

The title compound was synthesized according to general procedure **A1** from 2,4,6-trimethylbenzoyl chloride **1a** and 1,2-diphenylethyne **2p**, at a higher temperature (150 °C). Purification by preparative TLC (SiO_2, 5% EtOAc in hexane) afforded **3p** as a light yellow solid (64 mg, 180 µmol, 60%)

^{1}H NMR (500 MHz, $CDCl_3$) δ 7.25 – 7.23 (m, 5H), 7.11 – 7.04 (m, 3H), 6.97 – 6.95 (m, 2H), 6.87 – 6.86 (m, 2H), 3.44 (s, 3H), 2.29 (s, 3H), 2.27 (s, 6H) ppm.

^{13}C{^{1}H} (125 MHz, $CDCl_3$) δ 170.0, 144.4,138.4 137.8, 137.3, 137.1, 136.0, 134.3, 130.4, 130.0, 128.6, 128.4, 127.7, 127.7, 127.65, 51.9, 21.3, 20.1 ppm.

TLC: R_f = 0.13 (97.5:2.5 hexane:EtOAc)

HRMS (ESI, m/z): $[M+Na]^+$ calcd. for $C_{25}H_{24}O_2Na$, 379.1669; found 379.1668.

Note: The stereochemistry of the product was supported by X-ray analysis.

Methyl (Z)-2,3-bis(4-methoxy)-3-mesitylacrylate (3q)

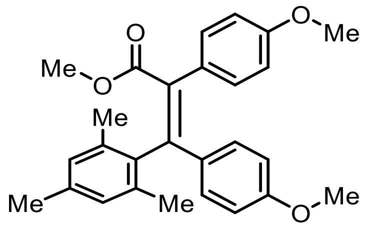

The title compound was synthesized according to general procedure **A1** from 2,4,6-trimethylbenzoyl chloride **1a** and 1,2-bis(4-methoxyphenyl)ethyne **2q** at a higher temperature (150 °C). Purification by preparative TLC (SiO_2, 5% EtOAc in hexane) afforded **3q** as an orange solid (45.6 mg, 104 µmol, 35%)

^{1}H NMR (400 MHz, $CDCl_3$) δ 7.19 –7.15 (m, 2H, ArH), 6.92 – 6.86 (m, 2H, ArH), 6.86 (m, 2H, ArH), 6.82 – 6.78 (m, 2H, ArH), 6.63 – 6.59 (m, 2H, ArH), 3.80 (s, 3H, OCH_3), 3.72 (s, 3H, OCH_3), 3.42 (s, 3H, CO_2CH_3), 2.29 (s, 3H, $ArCH_3$), 2.24 (s, 3H, 2 ×$ArCH_3$) ppm.

^{13}C{^{1}H} (100 MHz, $CDCl_3$) δ 170.4, 159.0, 158.9, 143.3, 138.1, 136.9, 136.0, 132.3, 131.7, 131.2, 130.9, 130.0, 128.3, 114.1, 113.3, 55.3, 55.2, 51.8, 21.3, 20.0 ppm.

TLC: R_f = 0.28 (90:10 hexane:EtOAc)

HRMS (ESI, m/z): $[M+Na]^+$ calcd. for $C_{27}H_{28}O_4Na$, 439.188; found 439.1873.

Methyl (Z)-3-mesityl-2,3-bis(4-(trifluoromethyl)phenyl)acrylate (3r)

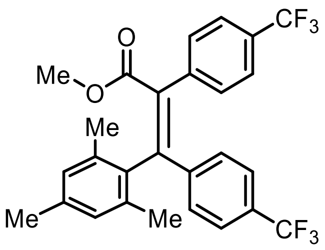

The title compound was synthesized according to general procedure **A1** from 2,4,6-trimethylbenzoyl chloride **1a** and 1,2-bis(4-methoxyphenyl)ethyne **2r** at a higher temperature (150 °C). Purification by preparative TLC (SiO_2, 5% EtOAc in hexane) afforded **3r** as an orange solid (45.6 mg, 104 µmol, 35%)

^{1}H NMR (400 MHz, $CDCl_3$) δ 7.19 –7.15 (m, 2H, ArH), 6.92 – 6.86 (m, 2H, ArH), 6.86 (m, 2H, ArH), 6.82 – 6.78 (m, 2H, ArH), 6.63 – 6.59 (m, 2H, ArH), 3.80 (s, 3H, OCH_3), 3.72 (s, 3H, OCH_3), 3.42 (s, 3H, CO_2CH_3), 2.29 (s, 3H, $ArCH_3$), 2.24 (s, 3H, 2 ×$ArCH_3$) ppm.

^{13}C{^{1}H} (100 MHz, $CDCl_3$) δ 170.4, 159.0, 158.9, 143.3, 138.1, 136.9, 136.0, 132.3, 131.7, 131.2, 130.9, 130.0, 128.3, 114.1, 113.3, 55.3, 55.2, 51.8, 21.3, 20.0 ppm.

^{19}F{^{1}H} (471 MHz, $CDCl_3$) δ – 62.7, – 62.8 ppm.

TLC: R_f = 0.28 (90:10 hexane:EtOAc)

HRMS (ESI, m/z): $[M+Na]^+$ calcd. for $C_{27}H_{28}O_4Na$, 439.188; found 439.1873.

3-mesityl-2-phenyl-1H-inden-1-one (3s)

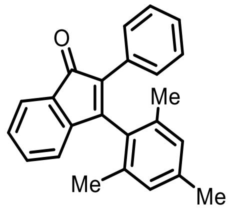

The title compound was synthesized according to general procedure **A1** from mesitoyl chloride **1a** and diphenylacetylene **2p**, after running the reaction for prolonged time (72 h) at higher temperature (150 °C) Purification by preparative TLC (SiO_2, 5% EtOAc in hexane) afforded **3s** as an orange solid (54.6 mg, 168 µmol, 56%).

^{1}H NMR (500 MHz, $CDCl_3$) δ 7.57 (ddd, J = 6.9, 1.3, 0.7 Hz, 1H), 7.37 – 7.33 (m, 2H), 7.33 – 7.28 (m, 1H), 7.28 – 7.20 (m, 4H), 6.94 (dd, J = 1.4, 0.7 Hz, 2H), 6.66 (dt, J = 7.2, 0.9 Hz, 1H), 2.34 (s, 3H), 2.08 (s, 6H) ppm.

^{13}C{^{1}H }(126 MHz, $CDCl_3$) δ 197.0, 156.6, 145.6, 138.2, 135.3, 134.0, 132.6, 131.6, 130.6, 129.6, 129.0, 128.8, 128.6, 128.3, 127.9, 122.8, 121.0, 21.3, 20.0.

HRMS (ESI, m/z): $[M+H]^+$ calcd. for, $C_{24}H_{20}O$ 324.1509; found 324.1503.

Note: The stereochemistry of the product was supported by 2D-NMR analysis.

Methyl (Z)-2-butyl-3-(2,6-dimethylphenyl)hept-2-enoate (S3a)

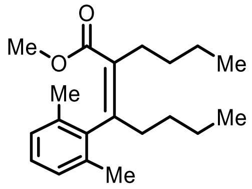

The title compound was synthesized according to general procedure **A1** from 2,6-dimethylbenzoylchloride **S1a** and 5-decyne **2a**. Purification by preparative TLC (SiO_2, 5% EtOAc in hexane) afforded **S3a** as a yellow oil (68.2 mg, 225 µmol, 75%).

^{1}H NMR (400 MHz, $CDCl_3$) δ 7.07 – 6.89 (m, 3H), 3.32 (s, 3H), 2.52 – 2.43 (m, 2H), 2.39 – 2.30 (m, 2H), 2.16 (s, 6H), 1.51 – 1.35 (m, 3H), 1.35 – 1.20 (m, 5H), 0.95 (t, J = 7.2 Hz, 3H), 0.87 (td, J = 10.3, 7.3 Hz, 3H) ppm.

^{13}C{^{1}H} (101 MHz, $CDCl_3$) δ 170.2, 145.6, 141.8, 134.9, 131.9, 127.2, 126.4, 51.2, 34.6, 31.3, 30.0, 29.2, 23.5, 22.8, 20.2, 14.1, 14.0 ppm.

HRMS (ESI, m/z): $[M+Na]^+$ calcd. for $C_{20}H_{30}NaO_2$, 325.2138; found 325.2136.

Note: The stereochemistry of the product was supported by 2D-NMR analysis.

Methyl (Z)-2-butyl-3-phenylhept-2-enoate (S3b)

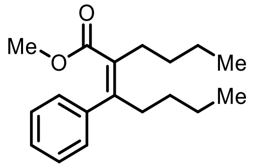

The title compound was synthesized according to general procedure **A1** from benzoyl chloride **S1b** and 5-decyne **2a**. Purification by preparative TLC (SiO_2, 5% EtOAc in hexane) afforded **S3b** as a colorless oil (7.2 mg, 27 µmol, 9%).

^{1}H NMR (400 MHz, $CDCl_3$) δ 7.31 – 7.18 (m, 3H), 7.14 – 7.09 (m, 2H), 3.33 (s, 3H), 2.44 (td, J = 8.0, 2.8 Hz, 4H), 1.47 – 1.32 (m, 4H), 1.26 (dddd, J = 10.7, 8.5, 5.6, 2.5 Hz, 4H), 0.93 (t, J = 7.1 Hz, 3H), 0.85 (t, J = 6.5, 6.1 Hz, 3H) ppm.

^{13}C{^{1}H} (101 MHz, $CDCl_3$) δ 171.5, 146.3, 142.8, 131.8, 128.0, 127.6, 127.0, 51.3, 34.0, 31.3, 30.2, 30.1, 22.8, 22.8, 14.1, 14.0 ppm.

HRMS (ESI, m/z): $[M+Na]^+$ calcd. for $C_{18}H_{26}NaO_2$, 297.1825; found 297.1821.

Note: The stereochemistry of the product was supported by 2D-NMR analysis.

Methyl (Z)-7-chloro-2-(4-chlorobutyl)-3-mesitylhept-2-enoate (S3l)

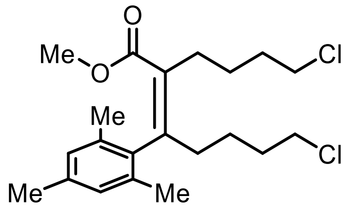

The title compound was synthesized according to general procedure **A1** from 2,4,6-trimethylbenzoyl chloride **1a** and 1,10-dichlorodec-5-yne **S2l**. Purification by preparative TLC (SiO_2, 5% EtOAc in hexane) afforded **S3l** as a colorless oil (57.5 mg, 150 µmol, 50%).

^{1}H NMR (400 MHz, $CDCl_3$) δ 6.80 (s, 2H), 3.59 (t, *J* = 6.6 Hz, 2H), 3.50 (t, *J* = 6.6 Hz, 2H), 3.36 (s, 3H), 2.54 – 2.46 (m, 2H), 2.43 – 2.30 (m, 2H), 2.25 (s, 3H), 2.12 (s, 6H), 1.88 (dq, *J* = 8.2, 6.7 Hz, 2H), 1.76 (p, *J* = 6.9 Hz, 2H), 1.70 – 1.61 (m, 2H), 1.48 (dtd, *J* = 9.4, 7.9, 7.4, 5.1 Hz, 2H) ppm.

^{13}C{^{1}H} (101 MHz, $CDCl_3$) δ 169.7, 145.7, 138.2, 136.0, 134.6, 131.8, 128.2, 51.4, 44.9, 44.7, 34.2, 33.1, 32.5, 28.8, 26.4, 25.1, 21.2, 20.2 ppm.

HRMS (ESI, m/z): $[M+Na]^+$ calcd. for $C_{21}H_{30}Cl_2NaO_2$, 407.1515; found 407.1514.

Note: The stereochemistry of the product was supported by 2D-NMR analysis.

Methyl (Z)-3-mesityl-2,3-di-p-tolylacrylate (S3m)

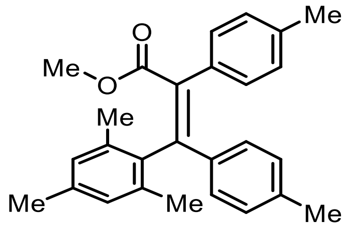

The title compound was synthesized according to general procedure **A1** from 2,4,6-trimethylbenzoyl chloride and 1,2-di-p-tolylethyne at a higher temperature (150 °C). Purification by preparative TLC (SiO_2, 5% EtOAc in hexane) afforded **S3m** as a yellow solid (41.2 mg, 108 µmol, 36%)

^{1}H NMR (500 MHz, $CDCl_3$) δ 7.13 (dt, *J* = 8.1, 1.9 Hz, 2H), 7.06 (dt, *J* = 7.9, 1.0Hz, 2H), 6.91 – 6.83 (m, 6H), 3.42 (s, 3H), 2.33 (s, 3H), 2.28 (s, 3H), 2.25 (d, *J* = 0.6 Hz, 6H), 2.24 (s, 3H).

^{13}C{^{1}H} (126 MHz, $CDCl_3$) δ 170.27, 143.73, 138.10, 137.43, 137.38, 136.89, 136.09, 135.58, 134.51, 133.52, 130.26, 129.78, 129.31, 128.61, 128.29, 51.77, 21.43, 21.31, 21.27, 20.09.

HRMS (ESI, m/z): $[M+Na]^+$ calcd. for for $C_{27}H_{28}NaO_2$, 407.1982; found 407.1973.

Methyl (Z)-2,3-bis(4-fluorophenyl)-3-mesitylacrylate (S3n)

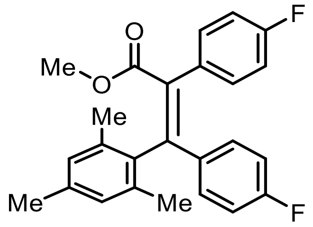

The title compound was synthesized according to general procedure **A1** from 2,4,6-trimethylbenzoyl chloride and 1,2-bis(4-fluorophenyl)ethyne at a higher temperature (150 °C). Purification by preparative TLC (SiO_2, 5% EtOAc in hexane) afforded **S3n** as an orange solid (50.1 mg, 129 µmol, 43%)

^{1}H NMR (500 MHz, $CDCl_3$) δ 7.22 – 7.16 (m, 2H), 6.99 – 6.89 (m, 4H), 6.89 – 6.85 (m, 2H), 6.81 – 6.74 (m, 2H), 3.44 (s, 3H), 2.29 (s, 3H), 2.24 (s, 6H).

^{13}C{^{1}H} (126 MHz, $CDCl_3$) δ 169.65, 162.25 (dd, *J* = 248.4, 27.4 Hz), 143.91, 133.72 (dd, *J* = 136.1, 3.4 Hz), 137.44, 137.38, 135.85, 133.72 (dd, *J* = 136.1, 3.4 Hz), 131.92 (dd, *J* = 48.0, 8.1 Hz), 128.50, 115.43 (dd, *J* = 89.7, 21.6 Hz), 51.95, 21.26, 20.06.

^{19}F{^{1}H} (471 MHz, $CDCl_3$) δ -113.3, -113.6.

HRMS (ESI, m/z): $[M+Na]^+$ calcd. for for $C_{25}H_{22}F_2NaO_2$, 415.1480; found 415.1475.

Cyclopentanone formation

General procedure B1

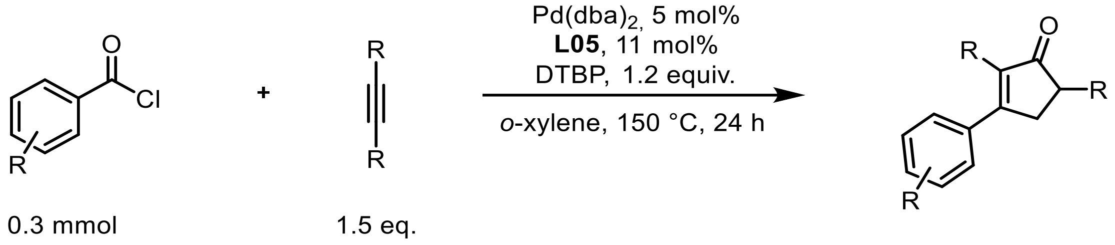

In a glovebox, to an oven dried 4 mL vial with a stirrer bar is added Pd(dba)$_2$ (8.60 mg, 15.0 µmol, 5.00 mol%), followed by 3,5-trifluoromethylphenyl DPEPhos (35.7 mg, 33.0 µmol, 11.0 mol%), and *o*-xylene (0.5 mL). The mixture is stirred for 10 min before addition of the corresponding alkyne (0.45 mmol, 1.50 equiv), acid chloride (0.30 mmol, 1.00 equiv.), and 2,6-ditertbutyl pyridine (68.9 mg, 0.36 mmol, 1.20 equiv.). The vial is then capped and removed from the glovebox and placed in a pre-heated stirring block at 150 °C 1200 rpm for 24 h. After the reaction has cooled, methanol (0.2 mL) is added and stirred for 10 min. The solution is then filtered through a plug of silica and rinsed with EtOAc and concentrated under reduced pressure. Subsequently purification is achieved by means of column chromatography or preparative TLC.

2-butyl-5-ethyl-3-mesitylcyclopent-2-en-1-one (4a)

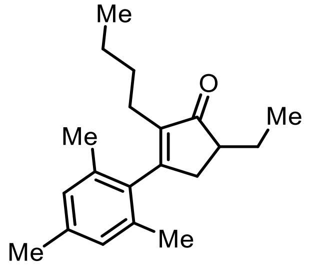

The title compound was synthesized according to general procedure **B1** from 2,4,6-trimethylbenzoylchloride and 5-decyne. Purification by column chromatography (SiO_2, 1.5% EtOAc in pentane) afforded **4a** as a yellow oil (52.0 mg, 0.18 mmol, 61%).

^{1}H NMR (400 MHz, $CDCl_3$) δ 6.91 (q, *J* = 0.6 Hz, 2H), 2.81 (ddt, *J* = 18.5, 6.6, 1.3 Hz, 1H), 2.49 – 2.40 (m, 1H), 2.39 – 2.32 (m, 1H), 2.31 (s, 3H), 2.12 (s, 3H), 2.10 (s, 3H), 1.96 (tt, *J* = 7.8, 1.3 Hz, 2H), 1.92 – 1.82 (m, 1H), 1.51 (ddt, *J* = 13.6, 8.8, 7.3 Hz, 1H), 1.31 – 1.23 (m, 2H), 1.18 (dddd, *J* = 9.0, 7.9, 5.8, 1.0 Hz, 2H), 0.99 (t, *J* = 7.4 Hz, 3H), 0.78 (t, *J* = 7.2 Hz, 3H).

^{13}C{^{1}H} (101 MHz, $CDCl_3$) δ 211.8, 170.1, 142.4, 137.5, 133.8, 133.8, 133.7, 128.5, 46.8, 37.2, 29.7, 24.8, 24.0, 23.0, 21.2, 19.6, 19.6, 13.9, 11.6.

Note: the mesitylene ring is not experiencing homogeneous shielding of the methyl groups in the ortho-position of the ring. Therefore, their carbon peak is split into two (both peaks at 19.6 ppm), as well as impacting the peak of the ortho-carbons in the ring splitting into two distinct signals (both peaks at133.8 ppm)

HRMS (ESI, m/z): $[M+H]^+$ calcd. for $C_{20}H_{28}O$, 284.2135; found 284.2136.

2-butyl-5-ethyl-3-(o-tolyl)cyclopent-2-en-1-one (4b)

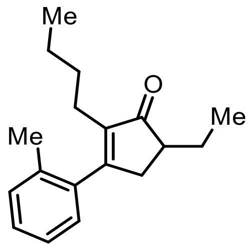

The title compound was synthesized according to general procedure **B1** from 2-methylbenzoylchloride and 5-decyne. Purification by preparative TLC (SiO_2, 4% EtOAc in hexane) afforded **4b** as a yellow oil (40.1 mg, 156 µmol, 52%).

^{1}H NMR (400 MHz, $CDCl_3$) δ 7.28 – 7.19 (m, 3H), 7.04 (m, 1H), 2.96 – 2.82 (m, 1H), 2.47 – 2.39 (m, 2H), 2.20 (s, 3H), 2.07 – 2.01 (m, 2H), 1.89 (dqd, *J* = 13.5, 7.5, 4.3 Hz, 1H), 1.52 (ddq, *J* = 13.6, 8.7, 7.4 Hz, 1H), 1.35 – 1.22 (m, 2H), 1.19 – 1.09 (m, 2H), 0.98 (t, *J* = 7.4 Hz, 3H), 0.74 (t, *J* = 7.3 Hz, 3H).

^{13}C{^{1}H} (101 MHz, $CDCl_3$) δ 211.7, 169.8, 142.1, 137.5, 134.0, 130.4, 128.2, 126.8, 125.9, 46.7, 38.3, 30.0, 24.8, 23.7, 22.7, 19.6, 11.4.

HRMS (ESI, m/z): $[M+H]^+$ calcd. for $C_{18}H_{25}O$, 257.1900; found 257.1898.

2-butyl-5-ethyl-3-(naphthalen-1-yl)cyclopent-2-en-1-one (4c)

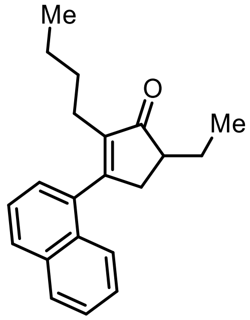

The title compound was synthesized according to general procedure **B1** from 1-(chloro-carbonyl)naphthalene and 5-decyne. Purification by preparative TLC (SiO_2, 2% EtOAc in hexane) afforded **4c** as a brown oil (40.9 mg, 140 μmol, 47%).

^{1}H NMR (400 MHz, $CDCl_3$) δ 7.94 – 7.89 (m, 1H), 7.87 (dd, *J* = 8.3, 1.1 Hz, 1H), 7.65 (dt, *J* = 2.0, 1.0 Hz, 1H), 7.59 – 7.45 (m, 3H), 7.29 (dd, *J* = 7.1, 1.2 Hz, 1H), 3.14 – 3.03 (m, 1H), 2.69 – 2.58 (m, 1H), 2.55 (dtt, *J* = 6.7, 4.4, 2.2 Hz, 1H), 2.06 (t, *J* = 7.7 Hz, 2H), 2.01 – 1.89 (m, 1H), 1.68 – 1.51 (m, 1H), 1.31 – 1.19 (m, 2H), 1.14 – 1.06 (m, 2H), 1.03 (t, *J* = 7.4 Hz, 3H), 0.66 (t, *J* = 7.3 Hz, 3H).

^{13}C{^{1}H} (101 MHz, $CDCl_3$) δ 211.5, 168.2, 143.5, 135.9, 133.7, 129.8, 128.7, 128.6, 126.6, 126.3, 125.4, 125.0, 124.2, 47.0, 39.3, 30.2, 25.0, 24.0, 22.6, 13.8, 11.5.

HRMS (ESI, m/z): $[M+H]^+$ calcd. for $C_{21}H_{25}O$, 293.1900; found 293.1895.

2-(2-butyl-4-ethyl-3-oxocyclopent-1-en-1-yl)phenyl acetate (4d)

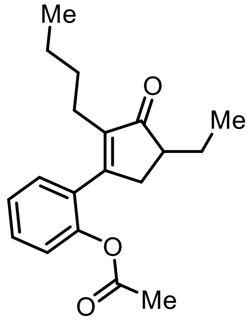

The title compound was synthesized according to general procedure **B1** from acetylsalicyloyl chloride and 5-decyne. Purification by preparative TLC (SiO_2, 12% EtOAc in hexane) afforded **4d** as a yellow oil (38.8 mg, 129 μmol, 43%).

^{1}H NMR (500 MHz, $CDCl_3$) δ 7.41 (ddd, *J* = 8.1, 7.4, 1.8 Hz, 1H), 7.32 (td, *J* = 7.5, 1.2 Hz, 1H), 7.24 (dd, *J* = 7.6, 1.8 Hz, 1H), 7.15 (dd, *J* = 8.1, 1.2 Hz, 1H), 2.97 – 2.83 (m, 1H), 2.53 – 2.45 (m, 1H), 2.41 (ddt, *J* = 8.8, 4.4, 2.3 Hz, 1H), 2.17 (s, 3H), 2.15 – 2.08 (m, 2H), 1.86 (dqd, *J* = 13.5, 7.5, 4.4 Hz, 1H), 1.55 – 1.43 (m, 1H), 1.34 – 1.24 (m, 2H), 1.16 (dt, *J* = 8.2, 7.0 Hz, 2H), 0.96 (t, *J* = 7.4 Hz, 3H), 0.76 (t, *J* = 7.3 Hz, 3H).

^{13}C{^{1}H} (125 MHz, $CDCl_3$) δ 211.5, 169.1, 164.6, 147.0, 142.9, 131.0, 129.8, 128.5, 126.3, 122.9, 46.7, 37.4, 30.0, 24.8, 23.9, 22.7, 21.0, 13.8, 11.3.

HRMS (ESI, m/z): $[M+H]^+$ calcd. for $C_{19}H_{25}O_3$, 301.1798; found 301.1794.

2-butyl-5-ethyl-3-(2-(trifluoromethoxy)phenyl)cyclopent-2-en-1-one (4e)

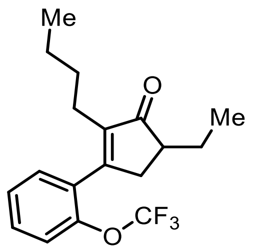

The title compound was synthesized according to general procedure **B1** from 2-(trifluoromethoxy)benzoylchloride and 5-decyne. Purification by preparative TLC (SiO_2, 2% EtOAc in hexane) afforded **4e** as a yellow oil (45.8 mg, 140 µmol, 47%).

^{1}H NMR (400 MHz, $CDCl_3$) δ 7.48 – 7.31 (m, 3H), 7.25 – 7.22 (m, 1H), 3.06 – 2.74 (m, 1H), 2.60 – 2.49 (m, 1H), 2.45 (dddd, *J* = 8.9, 6.8, 4.4, 2.4 Hz, 1H), 2.17 – 2.07 (m, 2H), 1.87 (dqd, *J* = 13.6, 7.5, 4.5 Hz, 1H), 1.52 (ddt, *J* = 13.5, 8.6, 7.2 Hz, 1H), 1.36 – 1.26 (m, 2H), 1.22 – 1.12 (m, 2H), 0.97 (t, *J* = 7.4 Hz, 3H), 0.78 (t, *J* = 7.2 Hz, 3H).

^{13}C{^{1}H} (101 MHz, $CDCl_3$) δ 211.4, 163.4, 145.8, 143.5, 131.2, 130.1, 129.4, 127.1, 120.5 (q, *J* = 258.2 Hz), 121.3 (q, *J* = 1.4 Hz) 46.7, 37.3, 29.9, 24.7, 23.9, 22.8, 13.8, 11.2.

^{19}F{^{1}H} (376 MHz, $CDCl_3$) δ -57.3

HRMS (ESI, m/z): $[M+H]^+$ calcd. for $C_{18}H_{22}F_3O_2$, 327.1566; found 327.1560.

2-butyl-5-ethyl-3-mesitylcyclopent-2-en-1-one (4f)

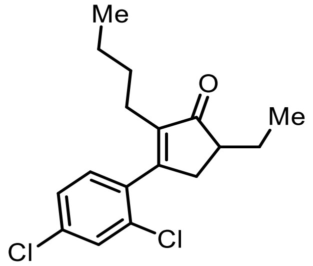

The title compound was synthesized according to general procedure **B1** from 2,4-dichlorobenzoyl chloride and 5-decyne. Purification by preparative TLC (SiO_2, 2% EtOAc in hexane) afforded **4f** as a brown oil (41.9 mg, 135 µmol, 45%).

^{1}H NMR (400 MHz, $CDCl_3$) δ 7.48 (d, *J* = 2.0 Hz, 1H), 7.31 (dd, *J* = 8.3, 2.1 Hz, 1H), 7.08 (d, *J* = 8.2 Hz, 1H), 2.99 – 2.85 (m, 1H), 2.54 – 2.47 (m, 1H), 2.44 (ddt, *J* = 8.9, 4.5, 2.3 Hz, 1H), 2.12 – 2.00 (m, 2H), 1.87 (dqd, *J* = 13.6, 7.5, 4.4 Hz, 1H), 1.53 (ddq, *J* = 13.6, 8.7, 7.3 Hz, 1H), 1.33 – 1.23 (m, 2H), 1.23 – 1.11 (m, 2H), 0.97 (t, *J* = 7.4 Hz, 3H), 0.76 (t, *J* = 7.2 Hz, 3H).

^{13}C{^{1}H} (101 MHz, $CDCl_3$) δ 211.2, 165.1, 143.6, 135.4, 134.9, 132.5, 130.0, 129.6, 127.4, 46.7, 37.2, 30.0, 24.7, 23.7, 22.7, 13.8, 11.4.

HRMS (ESI, m/z): $[M+H]^+$ calcd. for $C_{17}H_{21}Cl_2O$, 311.0964; found 311.0959.

2-butyl-5-ethyl-3-mesitylcyclopent-2-en-1-one (4g)

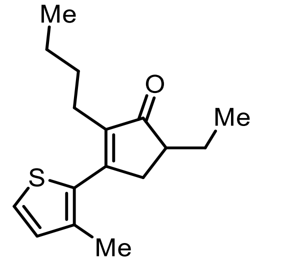

The title compound was synthesized according to general procedure **B1** from 3-methylthiophene-2-carbonyl chloride and 5-decyne. Purification by preparative TLC (SiO_2, 8% EtOAc in hexane) afforded **4g** as a yellow oil (31.7 mg, 121 µmol, 40%).

^{1}H NMR (500 MHz, $CDCl_3$) δ 7.35 (d, *J* = 5.1 Hz, 1H), 6.92 (d, *J* = 5.0 Hz, 1H), 3.03 (dd, *J* = 17.9, 6.8 Hz, 1H), 2.61 – 2.52 (m, 1H), 2.42 (dddd, *J* = 9.1, 6.9, 4.6, 2.5 Hz, 1H), 2.31 (ddd, *J* = 9.2, 6.5, 1.2 Hz, 2H), 2.27 (s, 3H), 1.88 (dqd, *J* = 13.6, 7.5, 4.4 Hz, 1H), 1.55 – 1.45 (m, 1H),

1.39 (ddd, J = 10.2, 4.9, 2.4 Hz, 2H), 1.31 – 1.22 (m, 2H), 0.97 (t, J = 7.4 Hz, 3H), 0.83 (t, J = 7.3 Hz, 3H).

^{13}C{^{1}H} (125 MHz, $CDCl_3$) δ 211.2, 160.4, 142.1, 136.4, 133.1, 131.3, 126.2, 46.7, 38.5, 30.0, 24.9, 24.5, 23.0, 16.0, 13.9, 11.4.

HRMS (ESI, m/z): $[M+H]^+$ calcd. for $C_{16}H_{23}OS$, 263.1464; found 263.1461.

2-butyl-5-ethyl-3-(3-methylbenzofuran-2-yl)cyclopent-2-en-1-one (4h)

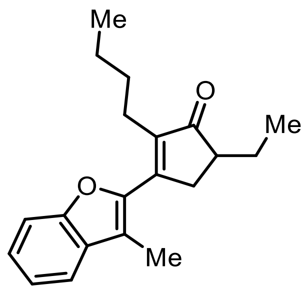

The title compound was synthesized according to general procedure **B1** from 3-methylbenzofuran-2-carbonyl chloride and 5-decyne. Purification by preparative TLC (SiO_2, 5% EtOAc in hexane) afforded **4h** as a yellow oil (11.0 mg, 37.1 μmol, 12%).

^{1}H NMR (400 MHz, $CDCl_3$) δ 7.58 (ddd, J = 7.8, 1.3, 0.7 Hz, 1H), 7.46 (dt, J = 8.3, 0.9 Hz, 1H), 7.38 (ddd, J = 8.3, 7.1, 1.3 Hz, 1H), 7.29 (ddd, J = 8.1, 7.1, 1.0 Hz, 1H), 3.26 – 3.17 (m, 1H), 2.75 – 2.66 (m, 3H), 2.52 (s, 3H), 2.46 (dddd, J = 8.8, 7.1, 4.5, 2.6 Hz, 1H), 1.95 – 1.88 (m, 1H), 1.56 – 1.45 (m, 3H), 1.43 – 1.37 (m, 2H), 1.00 (t, J = 7.4 Hz, 3H), 0.94 (t, J = 7.3 Hz, 3H).

^{13}C{^{1}H} (101 MHz, $CDCl_3$) δ 210.9, 154.7, 153.0, 148.8, 141.5, 130.2, 126.5, 122.9, 120.1, 119.2, 111.3, 46.3, 34.5, 31.3, 25.0, 24.5, 23.1, 14.1, 11.5, 10.1.

HRMS (ESI, m/z): $[M+H]^+$ calcd. for $C_{20}H_{25}O_2$, 297.1849; found 297.1851.

2-butyl-5-ethyl-3-(5-methyl-3-phenylisoxazol-4-yl)cyclopent-2-en-1-one (4i)

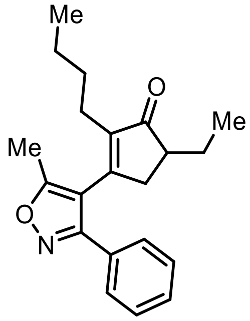

The title compound was synthesized according to general procedure **B1** from 5-methyl-3-phenylisoxazole-4-carbonyl chloride and 5-decyne. Purification by preparative TLC (SiO_2, 60% DCM in hexane) afforded **4i** as a yellow oil (27.9 mg, 86.3 µmol, 29%).

^{1}H NMR (500 MHz, $CDCl_3$) δ 7.54 (dd, *J* = 7.9, 1.8 Hz, 2H), 7.46 – 7.37 (m, 3H), 2.71 – 2.61 (m, 1H), 2.39 (s, 4H), 2.30 – 2.18 (m, 1H), 2.14 – 2.02 (m, 2H), 1.87 – 1.75 (m, 1H), 1.51 – 1.41 (m, 1H), 1.36 – 1.27 (m, 2H), 1.24 – 1.16 (m, 2H), 0.88 (t, *J* = 7.4 Hz, 3H), 0.79 (t, *J* = 7.2 Hz, 3H).

^{13}C{^{1}H} (125 MHz, $CDCl_3$) δ 211.0, 166.1, 160.9, 157.5, 144.9, 130.1, 129.0, 127.7, 111.8, 46.7, 36.9, 29.6, 24.6, 24.3, 23.0, 13.8, 12.1, 11.2.

HRMS (ESI, m/z): $[M+H]^+$ calcd. for $C_{21}H_{26}NO_2$, 324.1958; found 324.1960.

2-butyl-5-ethyl-3-(2-methyl-6-(trifluoromethyl)pyridin-3-yl)cyclopent-2-en-1-one (4j)

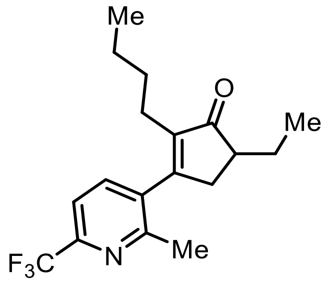

The title compound was synthesized according to general procedure **B1** from 2-methyl-6-trifluoromethyl-nicotinoyl chloride and 5-decyne. Purification by preparative TLC (SiO_2, 10% EtOAc in hexane) afforded **4j** as a orange oil (45.1 mg, 139 µmol, 46%).

^{1}H NMR (500 MHz, $CDCl_3$) δ 7.58 (d, *J* = 7.9 Hz, 1H), 7.54 (d, *J* = 7.9 Hz, 1H), 2.92 (dd, *J* = 17.2, 6.7 Hz, 1H), 2.55 – 2.41 (m, 5H), 2.03 (dd, *J* = 9.0, 6.9 Hz, 2H), 1.90 (ddq, *J* = 14.9, 7.5,

3.8 Hz, 1H), 1.61 – 1.48 (m, 1H), 1.33 – 1.23 (m, 2H), 1.23 – 1.11 (m, 2H), 0.98 (t, *J* = 7.4 Hz, 3H), 0.76 (t, *J* = 7.2 Hz, 3H).

^{13}C{^{1}H} (125 MHz, CDCl3) δ 210.5, 164.5, 155.7, 147.5 (q, *J* = 34.7 Hz), 143.8, 136.2, 135.9 (d, *J* = 1.3 Hz), 121.5 (q, *J* = 274.2 Hz), 117.8 (q, *J* = 2.8 Hz), 117.4, 46.7, 37.8, 30.0, 24.7, 23.8, 22.8, 13.8, 11.3.

HRMS (ESI, m/z): $[M+H]^+$ calcd. for $C_{18}H_{23}F_3NO$, 326.1726; found 326.1724.

5-(5-(2-butyl-4-ethyl-3-oxocyclopent-1-en-1-yl)-4-methylthiazol-2-yl)-2-isobutoxybenzonitrile (4k)

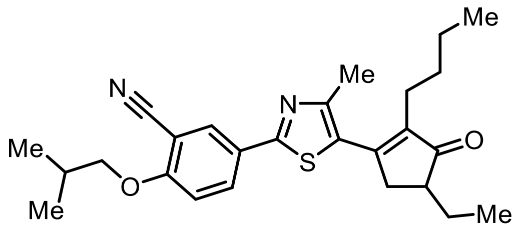

The title compound was synthesized according to general procedure **B1** from 2-(3-cyano-4-isobutoxyphenyl)-4-methylthiazole-5-carbonyl chloride and 5-decyne. Purification by column chromatography (SiO_2, gradient 8 to 12% EtOAc in hexane) afforded **4k** as an orange solid (39.9 mg, 91.4 µmol, 31%).

^{1}H NMR (500 MHz, CDCl3) δ 8.14 (d, *J* = 2.2 Hz, 1H), 8.07 (dd, *J* = 8.8, 2.3 Hz, 1H), 7.01 (d, *J* = 8.9 Hz, 1H), 3.90 (d, *J* = 6.5 Hz, 2H), 3.11 – 3.01 (m, 1H), 2.63 – 2.54 (m, 1H), 2.51 (s, 3H), 2.48 – 2.43 (m, 1H), 2.36 – 2.30 (m, 2H), 2.20 (hept, *J* = 6.7 Hz, 1H), 1.89 (ddd, *J* = 13.7, 7.5, 4.5 Hz, 1H), 1.55 – 1.47 (m, 1H), 1.44 – 1.36 (m, 2H), 1.32 – 1.23 (m, 2H), 1.09 (d, *J* = 6.8 Hz, 6H), 0.98 (t, *J* = 7.4 Hz, 3H), 0.86 (t, *J* = 7.3 Hz, 3H).

^{13}C{^{1}H} (125 MHz, CDCl3) δ 210.5, 165.0, 162.2, 156.6, 152.1, 143.4, 132.3, 131.9, 127.3, 126.4, 115.7, 112.8, 103.0, 75.8, 46.7, 38.3, 30.0, 28.3, 24.8, 24.6, 23.0, 19.2, 17.7, 13.9, 11.4.

HRMS (ESI, m/z): $[M+H]^+$ calcd. for $C_{26}H_{33}N_2O_2S$, 437.2257; found 437.2247.

2-butyl-5-ethyl-3-phenylcyclopent-2-en-1-one (4l)

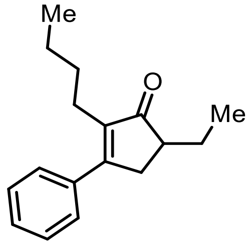

The title compound was synthesized according to general procedure **B1** from benzoyl chloride and 5-decyne. Purification by preparative TLC (SiO_2, 5% EtOAc in hexane) afforded **4l** as a yellow oil (11.8 mg, 48.7 µmol, 16%).

^{1}H NMR (400 MHz, $CDCl_3$) δ 7.49 – 7.36 (m, 5H, ArH), 3.05 (ddt, *J* = 17.9, 6.8 Hz, 1.2 Hz, 1H, CH_2), 2.57 (ddt, *J* = 17.9, 2.5, 1.2 Hz, 1H, CH_2), 2.45 –2.41 (m, 1H, C(Et)H), 2.40 – 2.35 (m, 2H, CH_2), 1.95 – 1.85 (m, 1H, CH_2), 1.53 – 1.42 (m, 3H, CH_2), 1.36 – 1.27 (m, 2H, CH_2), 0.99 (t, *J* = 7.4 Hz, 3H, CH_3), 0.87 (t, *J* = 7.3 Hz, 3H, CH_3) ppm.

^{13}C{^{1}H} (101 MHz, $CDCl_3$) δ 211.8, 165.6, 140.9, 136.9, 129.4, 128.7, 127.4, 46.4, 36.5, 32.1, 30.6, 29.8, 29.8, 29.5, 24.9, 24.0, 23.1, 22.8, 14.3, 14.0, 11.5 ppm.

HRMS (ESI, m/z): $[M+Na]^+$ calcd. for $C_{17}H_{22}ONa$, 265.1563; found 265.1558.

4-(2-butyl-4-ethyl-3-oxocyclopent-1-en-1-yl)benzonitrile (4m)

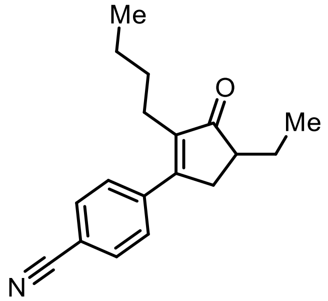

The title compound was synthesized according to general procedure **B1** from 4-cyanobenzoyl chloride and 5-decyne. Purification by preparative TLC (SiO_2, 80% DCM in hexane) afforded **4m** as a white solid (19.5 mg, 72.9 µmol, 24%).

^{1}H NMR (500 MHz, $CDCl_3$) δ 7.77 – 7.72 (m, 2H), 7.58 – 7.51 (m, 2H), 3.03 (dd, *J* = 17.9, 6.9 Hz, 1H), 2.63 – 2.52 (m, 1H), 2.45 (dddd, *J* = 9.2, 7.0, 4.5, 2.5 Hz, 1H), 2.32 (tt, *J* = 7.4,

1.2 Hz, 2H), 1.90 (dqd, J = 13.5, 7.5, 4.5 Hz, 1H), 1.50 (ddq, J = 13.6, 8.9, 7.3 Hz, 1H), 1.43 – 1.36 (m, 2H), 1.35 – 1.25 (m, 2H), 0.99 (t, J = 7.4 Hz, 3H), 0.86 (t, J = 7.3 Hz, 3H).

^{13}C{^{1}H} (125 MHz, $CDCl_3$) δ 211.0, 162.8, 143.0, 141.5, 132.6, 128.0, 118.5, 112.8, 46.5, 36.4, 30.5, 24.7, 23.9, 23.0, 13.9, 11.5.

HRMS (ESI, m/z): $[M+H]^+$ calcd. for $C_{18}H_{22}NO$, 268.1696; found 268.1698.

2-butyl-5-ethyl-3-mesitylcyclopent-2-en-1-one (4n)

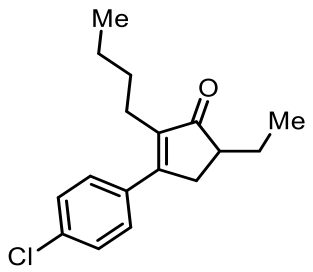

The title compound was synthesized according to general procedure **B1** from 4-chlorobenzoyl chloride and 5-decyne. Purification by preparative TLC (SiO_2, 5% EtOAc in hexane) afforded **4n** as a brown oil (19.3 mg, 69.7 µmol, 23%).

^{1}H NMR (500 MHz, $CDCl_3$) δ 7.47 – 7.37 (m, 4H), 3.01 (dd, J = 17.8, 6.8 Hz, 1H), 2.54 (ddt, J = 17.8, 2.4, 1.2 Hz, 1H), 2.42 (dddd, J = 9.2, 6.9, 4.5, 2.5 Hz, 1H), 2.38 – 2.29 (m, 2H), 1.89 (dqd, J = 13.6, 7.5, 4.5 Hz, 1H), 1.53 – 1.45 (m, 1H), 1.44 – 1.39 (m, 2H), 1.34 – 1.29 (m, 2H), 0.98 (t, J = 7.4 Hz, 3H), 0.87 (t, J = 7.3 Hz, 3H).

^{13}C{^{1}H} (125 MHz, $CDCl_3$) δ 211.4, 164.0, 141.3, 135.3, 135.3, 129.0, 128.7, 46.4, 36.4, 30.5, 24.8, 24.0, 23.0, 13.9, 11.5.

HRMS (ESI, m/z): $[M+Na]^+$ calcd. for $C_{17}H_{21}ClNaO$, 299.1173; found 299.1165.

2-(4-chlorobutyl)-5-(2-chloroethyl)-3-mesitylcyclopent-2-en-1-one (4o)

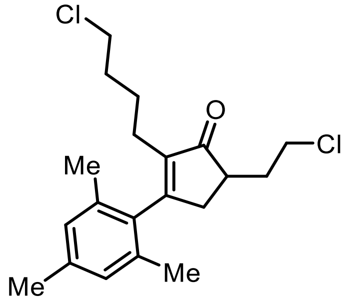

The title compound was synthesized according to general procedure **B1** from 2,4,6-trimethylbenzoylchloride and 1,10-dichlorodec-5-yne. Purification by preparative TLC (SiO_2, 7% EtOAc in hexane) afforded **4o** as a yellow oil (69.2 mg, 196 µmol, 65%).

^{1}H NMR (400 MHz, $CDCl_3$) δ 6.92 (s, 2H), 3.79 (dt, *J* = 10.9, 6.6 Hz, 1H), 3.75 – 3.65 (m, 1H), 3.41 (t, *J* = 6.7 Hz, 2H), 3.02 – 2.88 (m, 1H), 2.81 – 2.65 (m, 1H), 2.47 – 2.34 (m, 2H), 2.31 (s, 3H), 2.11 (s, 3H), 2.09 (s, 3H), 2.00 (dtd, *J* = 8.4, 7.2, 1.4 Hz, 2H), 1.92 – 1.82 (m, 1H), 1.68 – 1.59 (m, 2H), 1.54 – 1.37 (m, 2H).

^{13}C{^{1}H} (101 MHz, $CDCl_3$) δ 210.2, 170.7, 141.4, 137.9, 133.7, 133.5, 133.0, 128.6, 128.6, 44.7, 43.3, 43.2, 37.9, 34.7, 32.6, 24.8, 23.4, 21.2, 19.6, 19.6.

Note: the mesitylene ring is not experiencing homogeneous shielding of the methyl groups in the ortho-position of the ring. Therefore, their carbon peak is split into two (both peaks at 19.6 ppm), as well as impacting the peak of the ortho-carbons (133.7 and 133.5 ppm) and meta-carbons (both 128.6 ppm).

HRMS (ESI, m/z): $[M+H]^+$ calcd. for $C_{20}H_{27}Cl_2O$, 353.1433; found 353.1429.

5-benzyl-3-mesityl-2-(3-phenylpropyl)cyclopent-2-en-1-one (4p)

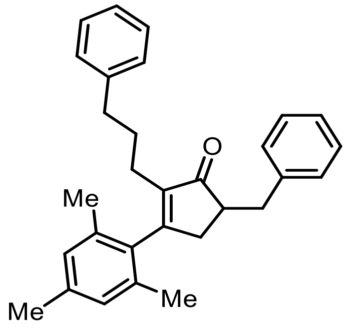

The title compound was synthesized according to general procedure **B1** from 2,4,6-trimethylbenzoylchloride and 1,8-diphenyloct-4-yne. Purification by preparative TLC (SiO_2, 5% EtOAc in hexane) afforded **4p** as a white solid (85.1 mg, 208 µmol, 69%).

^{1}H NMR (400 MHz, $CDCl_3$) δ 7.25 – 7.12 (m, 8H), 7.08 – 7.02 (m, 2H), 6.88 (s, 1H), 6.85 (s, 1H), 3.18 (dd, *J* = 13.5, 4.5 Hz, 1H), 2.92 (dd, *J* = 13.5, 8.2 Hz, 1H), 2.83 (dddd, *J* = 8.3, 6.7, 4.5, 2.3 Hz, 1H), 2.75 – 2.67 (m, 1H), 2.57 – 2.44 (m, 2H), 2.46 – 2.39 (m, 1H), 2.30 (s, 3H), 2.06 (s, 3H), 2.04 – 2.00 (m, 2H), 1.76 (s, 3H), 1.69 – 1.59 (m, 2H).

^{13}C{^{1}H} (101 MHz, $CDCl_3$) δ 210.9, 171.2, 142.2, 142.1, 139.1, 137.5, 133.7, 133.6, 133.4, 129.4, 128.5, 128.4, 128.4, 128.3, 128.3, 126.5, 125.7, 46.4, 37.1, 36.4, 35.9, 28.9, 24.0, 21.1, 19.6, 19.2.

Note: the mesitylene ring is not experiencing homogeneous shielding of the methyl groups in the ortho-position of the ring. Here, this results in distinct chemical shifts for each carbon in this ring, as well as for the two ortho methyl groups.

HRMS (ESI, m/z): $[M+H]^+$ calcd. for $C_{30}H_{33}O$, 409.2526; found 409.2524.

3-mesityl-2-methyl-5-propylcyclopent-2-en-1-one (4q)

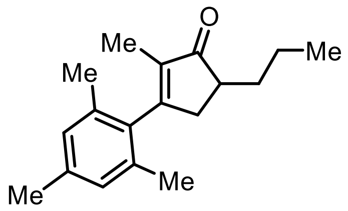

The title compound was synthesized according to general procedure **B1** from 2,4,6-trimethylbenzoylchloride and 2-octyne. Purification by preparative TLC (SiO_2, 5% EtOAc in hexane) afforded **4q** as an orange oil (21.2 mg, 82.7 µmol, 28%).

^{1}H NMR (400 MHz, $CDCl_3$) δ 6.92 (s, 2H), 2.92 – 2.79 (m, 1H), 2.51 (dt, *J* = 6.7, 2.3 Hz, 1H), 2.40 – 2.33 (m, 1H), 2.31 (s, 3H), 2.09 (d, *J* = 1.6 Hz, 6H), 1.94 – 1.81 (m, 1H), 1.53 (t, *J* = 2.1 Hz, 3H), 1.47 – 1.37 (m, 3H), 0.96 (t, *J* = 7.1 Hz, 3H).

^{13}C{^{1}H} (101 MHz, $CDCl_3$) δ 212.0, 170.3, 138.2, 137.6, 133.9, 133.7, 133.5, 128.4, 45.3, 37.7, 34.1, 21.1, 20.8, 19.4, 14.2, 8.7.

Note: splitting of the aromatic carbons bearing the ortho-methyl groups was observed (this results in two distinct carbon peaks at 133.7 and 133.9 ppm)

HRMS (ESI, m/z): $[M+Na]^+$ calcd. for $C_{18}H_{24}NaO$, 279.1719; found 279.1714.

Attempts to increase yield for non-ortho substituted acid chlorides

Due to the reduced yields of the cyclopentenone products with acid chlorides in the absence of ortho-substituents, several attempts to increase the yields for such scaffolds were undertaken.

One included the installation of a "transient bulk" moiety ortho to the acid chloride functionality. We reasoned that silyl or boryl substituents could protodesilylate or protodeborylate under the employed conditions as HCl was formed as a byproduct. However, upon subjecting the corresponding acid chlorides to our standard conditions, only ever the methyl esters after MeOH quench could be observed.

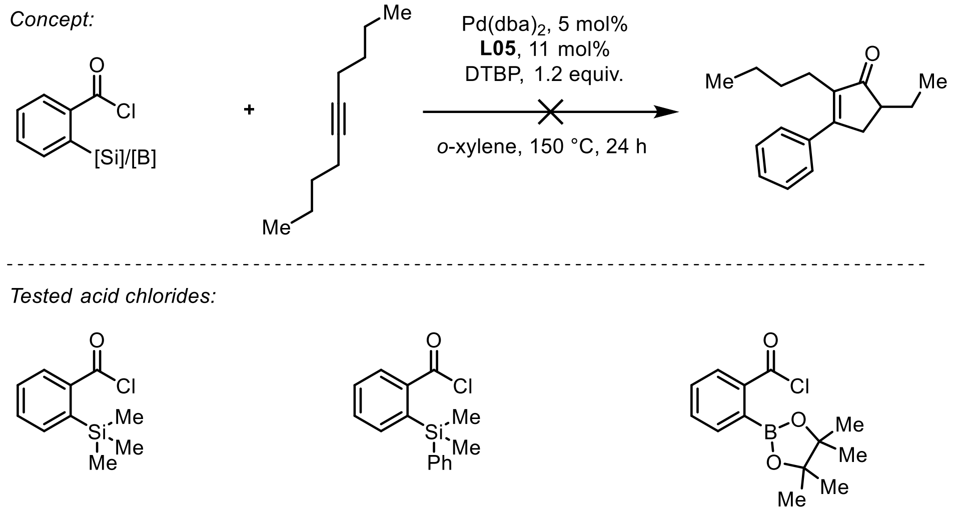

Starting material synthesis

2-(3-cyano-4-isobutoxyphenyl)-4-methylthiazole-5-carbonyl chloride (SM-1)

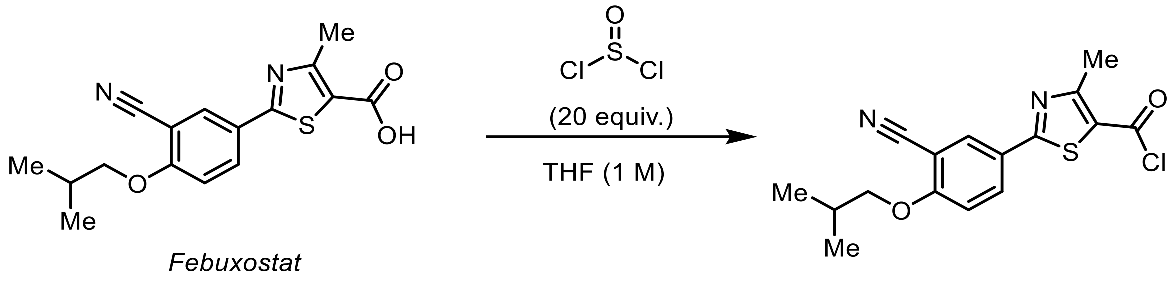

To a solution of 2-(3-cyano-4-isobutoxyphenyl)-4-methylthiazole-5-carboxylic acid (633 mg, 2.00 mmol, 1.00 equiv.) in THF (2.0 mL) was added thionyl chloride (2.92 mL, 40.0 mmol, 20.0 equiv.). The resulting mixture was stirred for 12 h at room temperature. Subsequently, volatiles were removed *in vacuo* and the resulting acid chloride used in the next step without further purification.

Mechanistic investigations

Isomers obtained under standard conditions:

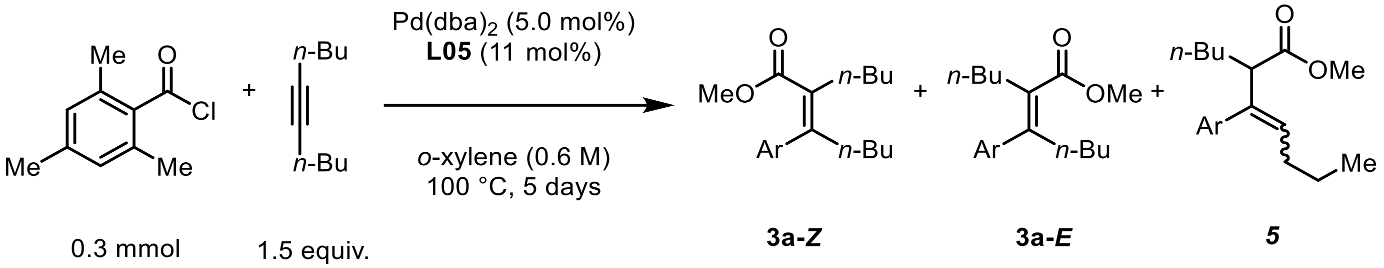

To obtain isomers of the carbochlorocarbonylation, the general procedure **A1** was followed, with the difference that the reaction temperature was increased to 100 °C and the reaction was stirred for 5 days before quenching with MeOH. Purification by preparative TLC afforded the three isomers. A ratio of Z:E:"alkene" of 0.30:0.46:0.38 was determined from the crude mixture after isolation of the corresponding compounds. The assignment of the Z-isomer was in line with the previously reported spectra (**3a**).

Methyl (E)-2-butyl-3-mesitylhept-2-enoate (3a-*E*)

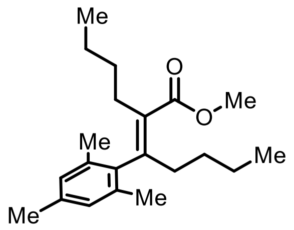

^{1}H NMR (500 MHz, $CDCl_3$) δ 6.93 – 6.80 (m, 2H), 3.80 (s, 3H), 2.47 – 2.38 (m, 2H), 2.28 (s, 3H), 2.15 (s, 6H), 1.99 – 1.87 (m, 2H), 1.34 – 1.18 (m, 6H), 1.17 – 1.08 (m, 2H), 0.83 (t, J = 7.1 Hz, 3H), 0.74 (t, J = 7.1 Hz, 3H).

^{13}C{^{1}H} (125 MHz, $CDCl_3$) δ 170.6, 145.9, 137.5, 136.1, 134.8, 131.4, 128.4, 51.4, 36.1, 31.0, 30.5, 30.2, 23.4, 22.7, 21.1, 20.1, 14.0, 13.9.

Methyl-2-butyl-3-mesitylhept-3-enoate (5)

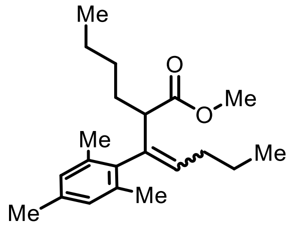

^{1}**H NMR** (500 MHz, $CDCl_3$) δ 6.85 (d, *J* = 3.2 Hz, 2H), 5.69 (td, *J* = 7.1, 1.2 Hz, 1H), 3.64 (s, 3H), 3.01 (ddd, *J* = 11.5, 3.7, 1.1 Hz, 1H), 2.27 (s, 3H), 2.13 (s, 3H), 2.09 (s, 3H), 2.01 – 1.84 (m, 1H), 1.65 (ddd, *J* = 7.9, 7.0, 0.9 Hz, 2H), 1.63 – 1.53 (m, 1H), 1.41 – 1.11 (m, 6H), 0.86 (t, *J* = 7.1 Hz, 3H), 0.80 (t, *J* = 7.4 Hz, 3H).

^{13}**C{^{1}H}** (125 MHz, $CDCl_3$) δ 174.9, 136.9, 136.5, 136.1, 135.7, 135.6, 129.5, 128.4, 128.3, 52.8, 51.7, 31.6, 31.3, 30.6, 22.8, 22.4, 21.1, 20.2, 19.7, 14.1, 14.1.

Absolute assignment of the (E)- or the (Z)-isomer was not possible based on the obtained analytical data. However, the general aptitude to access the alkene isomer is most significant.

Control reactions:

2-butyl-3-mesitylhept-2-enoyl chloride

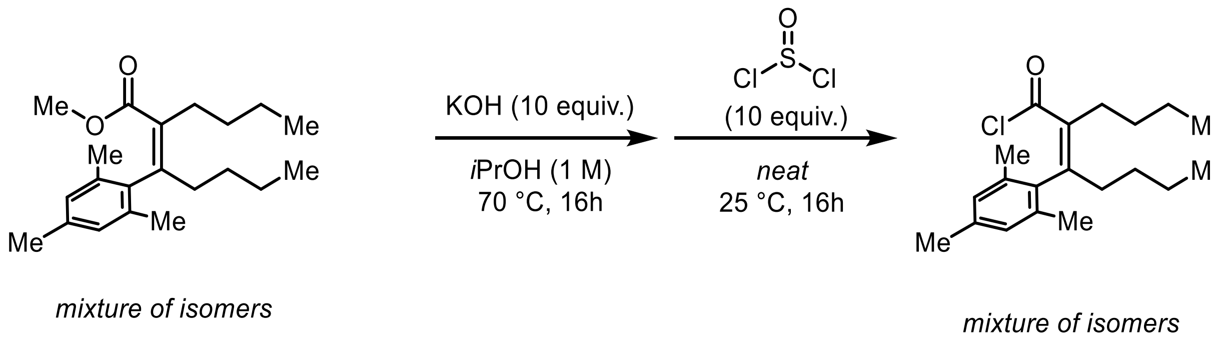

The title compound was synthesized from previously obtained **3a**, as previously described.

The methyl ester of **3a** (mixture of (E) and (Z) isomers; 247 mg, 0.90 mmol, 1.00 equiv. was dissolved in iPrOH (0.9 mL), and KOH (505 mg, 9.00 mmol, 10.0 equiv.) was added. The resulting mixture was stirred at 70 °C for 16 h. Subsequently, equivalent amounts of water and EtOAc (20 mL) were added and the aqueous phase acidified through the addition of 1 M HCl. The aqueous phase was washed with EtOAc (3 × 20 mL). The combined organic phases were dried over $MgSO_4$, volatiles removed *in vacuo*, and the resulting residue purified through column chromatography (SiO_2, 10% EtOAc in hexane), to obtain the intermediate acid in sufficient purity to proceed with the next step

The acid intermediate (391 mg, 1.50 mmol, 1.00 equiv.) was dissolved in thionyl chloride (1.09 mL, 15.0 mmol, 10.0 equiv.). The resulting solution was stirred for 16 h at room

temperature. Subsequently, volatiles were removed *in vacuo*. The resulting acid chloride was used without further purification.

Control reactions.

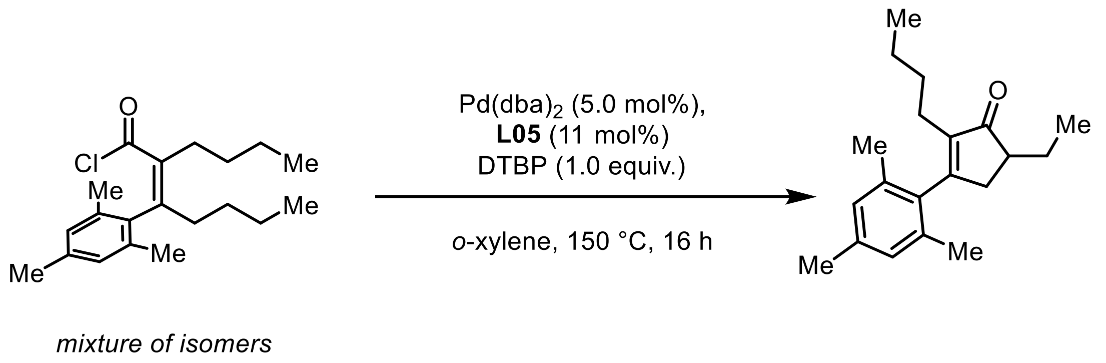

entry	**Deviation from conditions**	**yield (%)[a]**
1	none	53
2	no Pd	35
3	no Pd, no ligand	46
4	no Pd, no ligand, no base	91

Cinnamoyl chloride (0.10 mmol, 1.00 equiv.), Pd(dba)2 (5.00 µmol, 5.00 mol%), L05 (11.0 µmol, 11 mol%), DTBP (0.10 mmol, 1.00 equiv.) *o*-xylene (0.6 M) [a]NMR yield using 1,3,5-trimethoxybenzene (0.33 equiv.) as internal standard.

Interrupted reaction:

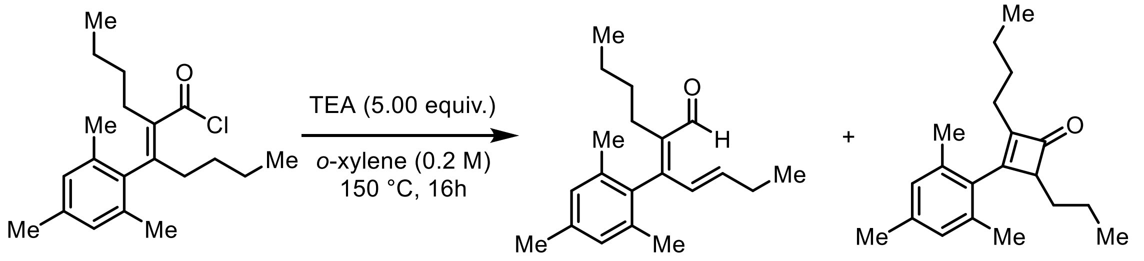

6 **7**

1 : 1.4

In a 4 mL drum vial in a glovebox, the acid chloride **3a** (100 mg, 312 µmol, 1.00 equiv.), was dissolved in *o*-xylene (1.6 mL) and closed with a septum cap. Upon removal from the glovebox, triethylamine (217 µL, 1.56 mmol, 5.00 equiv.) was added and the resulting mixture heated to 150 °C for 16 h. Upon completion, MeOH (1 mL) was added and the mixture stirred for 10 min at room temperature. Volatiles were removed in vaccuo and the remains purified by preparative TLC (SiO_2, 4% EtOAc in hexane – DNP stain was helpful to identify the aldehyde product). The thereby obtained products were identified as an inseparable mixture of the aldehyde product **6** and the cyclobutenone product **7** (ratio 1:1.4) (28.4 mg, 99.8 µmol, 32%).

Due to overlap in the aliphatic region, we chose to assign the key proton and carbon signals of the two species below (assignments were based on COSY, HSQC and HMBC observations)

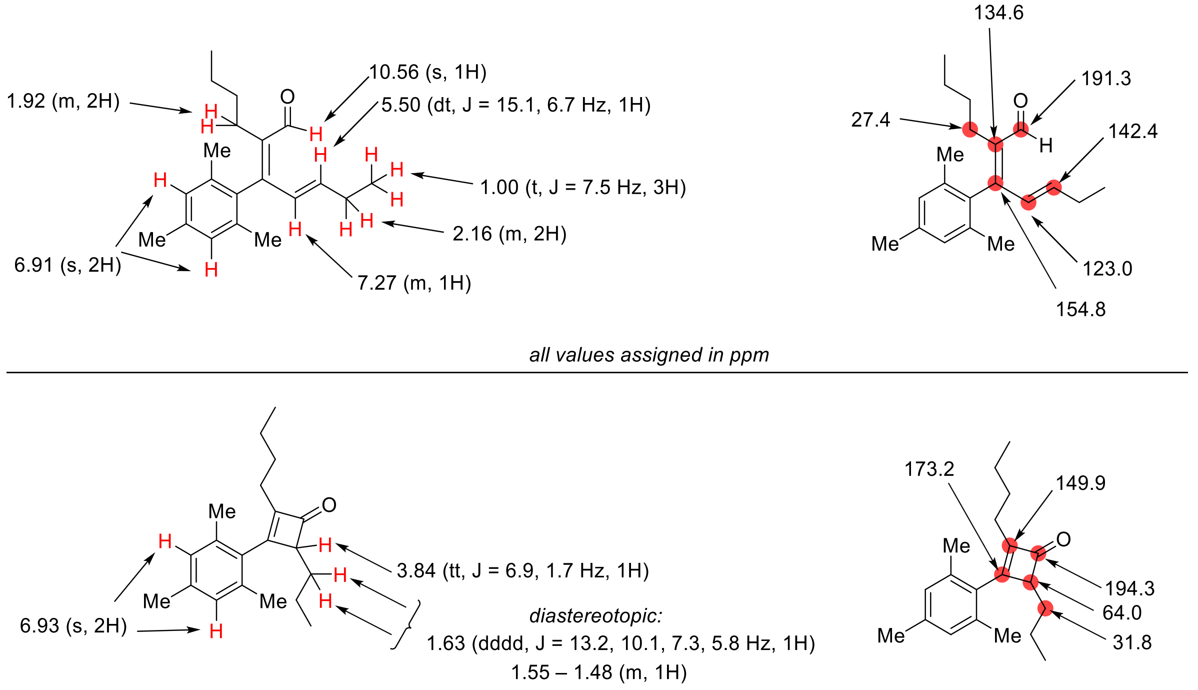

all values assigned in ppm

HRMS (ESI, m/z): $[M+H]^+$ calcd. for $C_{20}H_{29}O$, 285.2213; found 285.2207.

Recovery of reactivity:

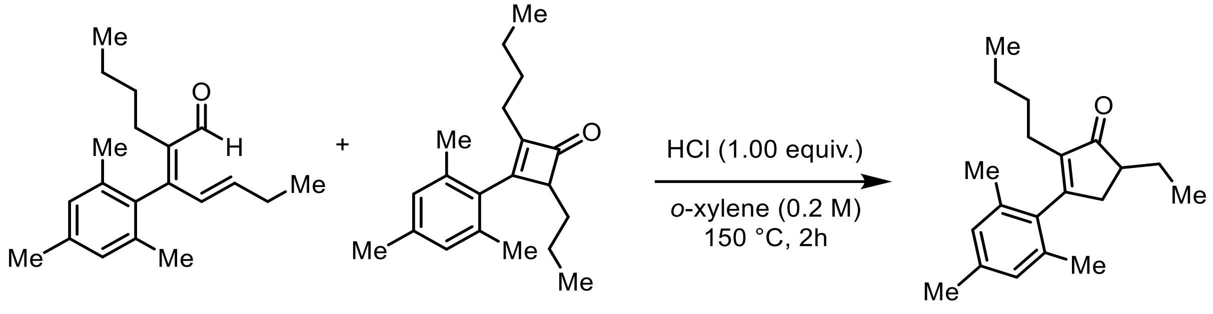

4a

The previously obtained mixture (28.4 mg, 99.8 µmol, 1.00 equiv.) was dissolved in *o*-xylene (0.5 mL), and HCl was added (4 M solution in dioxane, 25.0 µL, 99.8 µmol, 1.00 equiv.). The resulting mixture was heated to 150 °C for 2 h. Volatiles were subsequently removed *in vacuo*. Analysis of the resulting residue by NMR showed complete conversion to the previously obtained **4a**.

Synthesis of highly substituted pyridines:

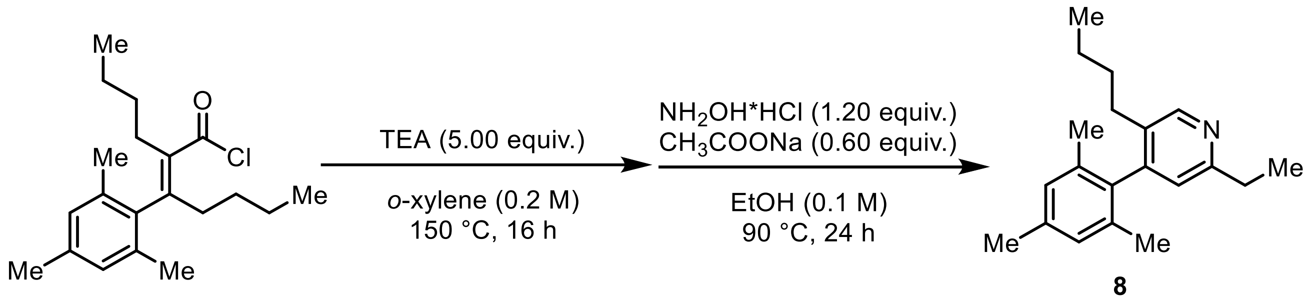

8

The second step in the synthetic sequence was adapted from the literature.

In a 4 mL drum vial in a glovebox, acid chloride (100 mg, 312 µmol, 1.00 equiv.), was dissolved in *o*-xylene (1.6 mL) and closed with a septum cap. Upon removal from the glovebox, triethylamine (217 µL, 1.56 mmol, 5.00 equiv.) was added and the resulting mixture heated to 150 °C for 16 h. Upon completion, MeOH (1 mL) was added and the mixture stirred for 10 min at room temperature. Volatiles were removed in vaccuo. The thereby obtained residue was redissolved in EtOH (3.2 mL) and hydroxylamine hydrochloride (26.4 mg, 380 µmol, 1.20 equiv.) as well as sodium acetate (15.6 mg, 190 µmol, 0.60 equiv.) were added. After heating to 90 °C for 24 h, sat. $NaHCO_3$-solution (5 mL) was added, and the aqueous phase extracted with EtOAc (3 × 5 mL). The combined organic residues were dried over $MgSO_4$, and

volatiles removed *in vacuo*. Purification by preparative TLC (SiO_2, 12% EtOAc in hexane) afforded the pyridine product **8** as a yellow oil (18.4 mg, 65.4 μmol, 21%).

^{1}H NMR (500 MHz, $CDCl_3$) δ 8.45 (s, 1H), 7.01 – 6.90 (m, 2H), 6.83 (s, 1H), 2.82 (q, J = 7.6 Hz, 2H), 2.33 (s, 3H), 2.31 – 2.24 (m, 2H), 1.92 (s, 6H), 1.41 – 1.35 (m, 2H), 1.30 (t, J = 7.6 Hz, 3H), 1.28 – 1.14 (m, 2H), 0.79 (t, J = 7.3 Hz, 3H).

^{13}C{^{1}H} (125 MHz, $CDCl_3$) δ 161.0, 149.8, 149.3, 137.2, 135.7, 135.1, 133.4, 128.4, 122.8, 32.1, 30.8, 29.8, 22.6, 21.2, 20.5, 14.0, 13.9.

HRMS (ESI, m/z): $[M+H]^+$ calcd. for $C_{20}H_{28}N$, 282.2216; found 282.2218.

X-ray Crystal Structures

General

Single crystalline samples were measured on a Rigaku Oxford Diffraction XtaLAB Synergy-S Dualflex kappa diffractometer equipped with a Dectris Pilatus 300 HPAD detector and using microfocus sealed tube Cu-Kα or Mo-Kα radiation with mirror optics.

Crystals were suspended in perfluoropolyalkyl ether oil (ABCR) in a glovebox under Ar atmosphere and kept in a closed Ar-filled vessel until final sample preparation. Specimen were mounted on Kapton sample holders (MiTeGen) for measurement.

All measurements were carried out at 100K using an Oxford Cryosystems Cryostream 800 sample cryostat. Data were integrated using CrysAlisPro and corrected for absorption effects using a combination of empirical (ABSPACK) and numerical corrections. The structures were solved using SHELXS[7] or SHELXT[8] and refined by full-matrix least-squares analysis (SHELXL)[7,9], using the program package OLEX2[10]. All non-hydrogen atoms were refined anisotropically. Hydrogen atoms were constrained to ideal geometries and refined with fixed isotropic displacement parameters (in terms of a riding model). CCDC 2127243-2127258 and 2127259-2127273 contain the supplementary crystallographic data for this paper, including structure factors and refinement instructions. These data can be obtained free of charge from The Cambridge Crystallographic Data Centre, 12 Union Road, Cambridge CB2 1EZ, UK (fax: +44(1223)-336-033; e-mail: deposit@ccdc.cam.ac.uk), or *via* https://www.ccdc.cam.ac.uk/structures.

(Oxydi-2,1-phenylene)bis(dichlorophosphine) **SI01**

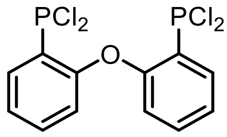

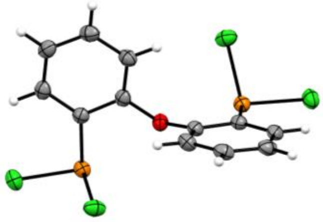

SI01 **SI01**
X-Ray

CCDC #	2415470
Empirical formula	$C_{12}H_8Cl_4OP_2$
Formula weight	371.92
Temperature/K	100.0(1)
Crystal system	monoclinic
Space group	*I*2/*a* (15)
a/Å	13.3658(3)
b/Å	8.8568(2)
c/Å	13.3140(3)
α/°	90
β/°	106.552(2)
γ/°	90
Volume/Å^3	1510.78(6)
Z	4
ρ_{calc} g/cm^3	1.635
μ/mm^{-1}	9.031
F(000)	744
Crystal size/mm^3	0.163×0.121×0.094
Crystal colour	clear colourless
Crystal shape	block
Radiation	Cu *K*α (λ=1.54184 Å)
2Θ range/°	12.15 to 158.84
Index ranges	−16 ≤ h ≤ 14
	−11 ≤ k ≤ 10
	−16 ≤ l ≤ 16
Reflections collected	16231
Independent reflections	1558
	*R*int = 0.0665
	*R*sigma = 0.0256
Data / Restraints / Param.	1558/0/87
Goodness-of-fit on F^2	1.084
Final *R* indexes [$I \geq 2\sigma(I)$]	*R*1 = 0.0377
	w*R*2 = 0.1032
Final *R* indexes [all data]	*R*1 = 0.0390
	w*R*2 = 0.1045
Largest peak/hole /eÅ^3	0.57/-0.65

(Oxydi-2,1-phenylene)bis{bis[3,5-bis(trifluoromethyl)phenyl]phosphine} **L05**

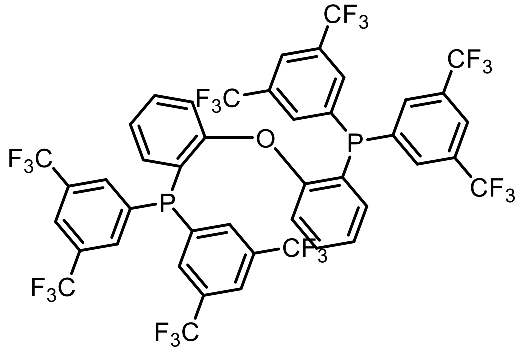

L05

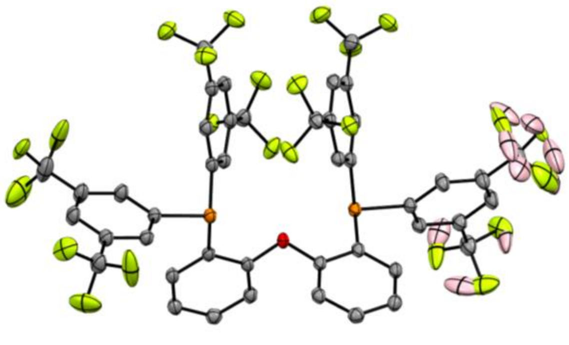

L05
X-Ray

CCDC #	2415471
Empirical formula	$C_{44}H_{20}F_{24}OP_2$
Formula weight	1082.49
Temperature/K	100.0(1)
Crystal system	monoclinic
Space group	*C2/c* (15)
a/Å	14.35080(10)
b/Å	10.98560(10)
c/Å	27.4216(2)
α/°	90
β/°	97.9340(10)
γ/°	90
Volume/Å^3	4281.69(6)
Z	4
ρ_{calc} g/cm^3	1.679
μ/mm^{-1}	2.241
F(000)	2152
Crystal size/mm^3	0.226×0.172×0.117
Crystal colour	clear yellow
Crystal shape	block
Radiation	Cu K_α (λ=1.54184 Å)
2Θ range/°	10.18 to 159.46
Index ranges	−17 ≤ h ≤ 17<br>−13 ≤ k ≤ 13<br>−34 ≤ l ≤ 31
Reflections collected	66351
Independent reflections	4454<br>R_{int} = 0.0384<br>R_{sigma} = 0.0153
Data / Restraints / Param.	4454/586/406
Goodness-of-fit on F^2	1.045
Final *R* indexes [*I*≥2σ(*I*)]	R_1 = 0.0319<br>wR_2 = 0.0829
Final *R* indexes [all data]	R_1 = 0.0337<br>wR_2 = 0.0841
Largest peak/hole /eÅ^3	0.36/-0.28

10-{3,5-Bis(trifluoromethyl)phenyl}-10*H*-phenoxaphosphinine **SI02**

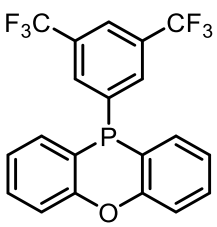

SI02

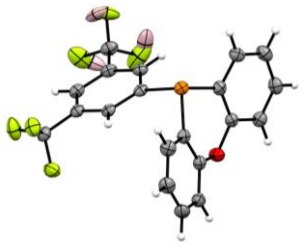

SI02
X-Ray

CCDC #	2415472
Empirical formula	$C_{20}H_{11}F_6OP$
Formula weight	412.26
Temperature [K]	100.0(1)
Crystal system	triclinic
Space group (number)	*P-1* (2)
a [Å]	4.9384(3)
b [Å]	12.0189(5)
c [Å]	14.9207(5)
α [°]	93.424(3)
β [°]	91.606(4)
γ [°]	95.752(4)
Volume [Å^3]	879.06(7)
Z	2
ρ_{calc} [gcm^{-3}]	1.558
μ [mm^{-1}]	2.042
F(000)	416
Crystal size [mm^3]	0.235×0.053×0.039
Crystal colour	clear colourless
Crystal shape	plate
Radiation	Cu K_α (λ=1.54184 Å)
2θ range [°]	7.41 to 159.91
Index ranges	$-6 \le h \le 4$<br>$-15 \le k \le 14$<br>$-19 \le l \le 18$
Reflections collected	12806
Independent reflections	3567<br>R_{int} = 0.0597<br>R_{sigma} = 0.0456
Data / Restraints / Parameters	3567/87/281
Goodness-of-fit on F^2	1.079
Final *R* indexes [$I \ge 2\sigma(I)$]	R_1 = 0.0457<br>wR_2 = 0.1255
Final *R* indexes [all data]	R_1 = 0.0523<br>wR_2 = 0.1303
Largest peak/hole [eÅ^{-3}]	0.54/-0.51

Bis{2-[bis(3,5-bis(trifluoromethyl)phenyl)phosphino]phenyl} sulfide **L06**

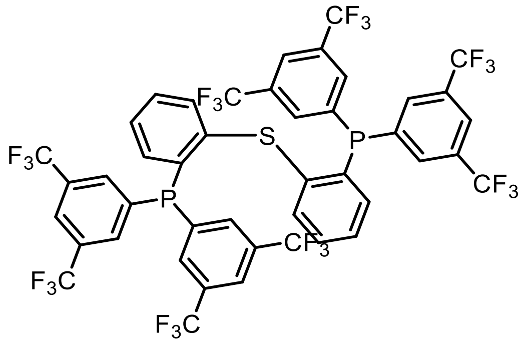

L06

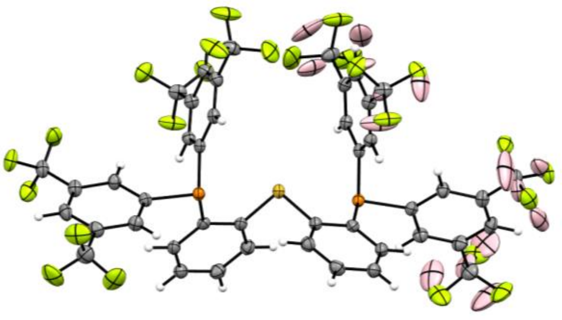

L06
X-Ray

CCDC #	2415473
Empirical formula	$C_{44}H_{20}F_{24}P_2S$
Formula weight	1098.60
Temperature [K]	100.0(1)
Crystal system	monoclinic
Space group (number)	*C2/c* (15)
a [Å]	15.6565(2)
b [Å]	10.19320(10)
c [Å]	28.3373(3)
α [°]	90
β [°]	97.4220(10)
γ [°]	90
Volume [Å^3]	4484.46(9)
Z	4
ρ_{calc} [gcm^{-3}]	1.627
μ [mm^{-1}]	2.556
F(000)	2184
Crystal size [mm^3]	0.32×0.227×0.064
Crystal colour	clear colourless
Crystal shape	plate
Radiation	Cu K_α (λ=1.54184 Å)
2θ range [°]	10.38 to 158.94
Index ranges	−19 ≤ h ≤ 18<br>−12 ≤ k ≤ 12<br>−32 ≤ l ≤ 35
Reflections collected	27388
Independent reflections	4684<br>R_{int} = 0.0323<br>R_{sigma} = 0.0210
Data / Restraints / Parameters	4684/838/459
Goodness-of-fit on F^2	1.082
Final *R* indexes [$I \geq 2\sigma(I)$]	R_1 = 0.0357<br>wR_2 = 0.0852
Final *R* indexes [all data]	R_1 = 0.0386<br>wR_2 = 0.0867
Largest peak/hole [eÅ^{-3}]	0.33/-0.31

Bis[3,5-bis(trifluoromethyl)phenyl](2-phenoxyphenyl)phosphine **L07**

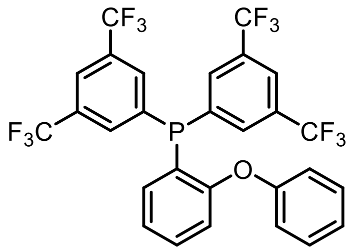

L07

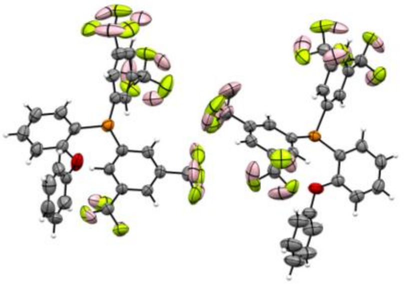

L07
X-Ray

CCDC #	2415474
Empirical formula	$C_{28}H_{15}F_{12}OP$
Formula weight	626.37
Temperature [K]	200.0(1)
Crystal system	triclinic
Space group (number)	*P-1* (2)
a [Å]	10.1603(2)
b [Å]	13.1699(2)
c [Å]	21.3714(3)
α [°]	76.5740(10)
β [°]	77.9530(10)
γ [°]	84.1650(10)
Volume [Å^3]	2716.04(8)
Z	4
ρ_{calc} [gcm^{-3}]	1.532
μ [mm^{-1}]	1.868
F(000)	1256
Crystal size [mm^3]	0.188×0.05×0.039
Crystal colour	clear colourless
Crystal shape	needle
Radiation	Cu K_α (λ=1.54184 Å)
2θ range [°]	6.91 to 162.06
Index ranges	$-10 \leq h \leq 12$<br>$-15 \leq k \leq 16$<br>$-25 \leq l \leq 26$
Reflections collected	47039
Independent reflections	10859<br>R_{int} = 0.0593<br>R_{sigma} = 0.0451
Data / Restraints / Parameters	10859/1552/995
Goodness-of-fit on F^2	1.077
Final *R* indexes [$I \geq 2\sigma(I)$]	R_1 = 0.0496<br>wR_2 = 0.1372
Final *R* indexes [all data]	R_1 = 0.0710<br>wR_2 = 0.1531
Largest peak/hole [eÅ^{-3}]	0.33/-0.31

Methyl (Z)-3-mesityl-2,3-diphenylacrylate **3o**

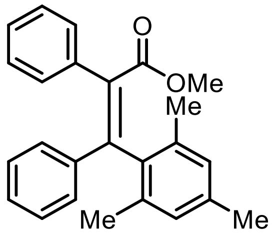

3o

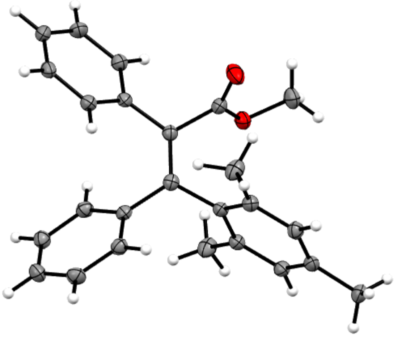

3o
X-Ray

CCDC #	2415475
Empirical formula	$C_{25}H_{24}O_2$
Formula weight	356.44
Temperature [K]	100.0(1)
Crystal system	monoclinic
Space group (number)	$P2_1/n$ (14)
a [Å]	9.66590(10)
b [Å]	9.35090(10)
c [Å]	21.5758(2)
α [°]	90
β [°]	98.0790(10)
γ [°]	90
Volume [Å^3]	1930.77(3)
Z	4
ρ_{calc} [gcm^{-3}]	1.226
μ [mm^{-1}]	0.595
F(000)	760
Crystal size [mm^3]	0.18×0.144×0.066
Crystal colour	clear colourless
Crystal shape	block
Radiation	Cu K_α (λ=1.54184 Å)
2θ range [°]	8.28 to 159.83
Index ranges	$-12 \leq h \leq 10$<br>$-11 \leq k \leq 11$<br>$-27 \leq l \leq 27$
Reflections collected	38737
Independent reflections	4174<br>R_{int} = 0.0483<br>R_{sigma} = 0.0235
Data / Restraints / Parameters	4174/0/248
Goodness-of-fit on F^2	1.049
Final R indexes [$I \geq 2\sigma(I)$]	R_1 = 0.0385<br>wR_2 = 0.0972
Final R indexes [all data]	R_1 = 0.0426<br>wR_2 = 0.1002
Largest peak/hole [eÅ^{-3}]	0.22/-0.21

2-Ethyl-3-mesityl-5-methylcyclopent-2-en-1-one **4a**

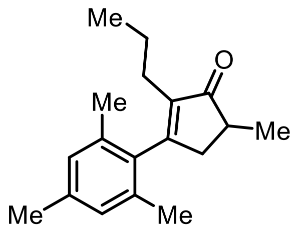

4a

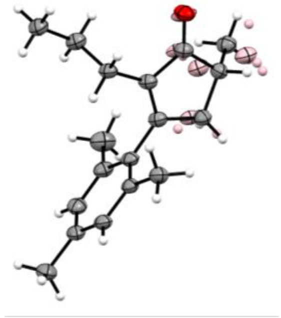

4a
X-Ray

CCDC #	2415476
Empirical formula	$C_{18}H_{24}O$
Formula weight	256.37
Temperature [K]	100.0(1)
Crystal system	triclinic
Space group (number)	*P-1* (2)
a [Å]	8.5214(2)
b [Å]	8.9855(2)
c [Å]	11.1011(3)
α [°]	82.786(2)
β [°]	74.562(2)
γ [°]	67.789(2)
Volume [Å^3]	758.24(3)
Z	2
ρ_{calc} [gcm^{-3}]	1.123
μ [mm^{-1}]	0.511
F(000)	280
Crystal size [mm^3]	0.174×0.105×0.063
Crystal colour	clear colourless
Crystal shape	block
Radiation	Cu K_α (λ=1.54184 Å)
2θ range [°]	8.27 to 159.46
Index ranges	$-10 \le h \le 10$<br>$-11 \le k \le 11$<br>$-14 \le l \le 12$
Reflections collected	23226
Independent reflections	3205<br>R_{int} = 0.0413<br>R_{sigma} = 0.0238
Data / Restraints / Parameters	3205/288/224
Goodness-of-fit on F^2	1.071
Final *R* indexes [$I \geq 2\sigma(I)$]	R_1 = 0.0463<br>wR_2 = 0.1234
Final *R* indexes [all data]	R_1 = 0.0535<br>wR_2 = 0.1289
Largest peak/hole [eÅ^{-3}]	0.19/-0.22

References

(1) Emmett, E. J.; Hayter, B. R.; Willis, M. C. Palladium-Catalyzed Three-Component Diaryl Sulfone Synthesis Exploiting the Sulfur Dioxide Surrogate DABSO. *Angew. Chemie Int. Ed.* **2013**, *52* (48), 12679–12683. https://doi.org/10.1002/anie.201305369.

(2) Metters, O. J.; Flynn, S. R.; Dowds, C. K.; Sparkes, H. A.; Manners, I.; Wass, D. F. Catalytic Dehydrocoupling of Amine–Boranes Using Cationic Zirconium(IV)–Phosphine Frustrated Lewis Pairs. *ACS Catal.* **2016**, *6* (10), 6601–6611. https://doi.org/10.1021/acscatal.6b02211.

(3) Guiu, E.; Caporali, M.; Muñoz, B.; Müller, C.; Lutz, M.; Spek, A. L.; Claver, C.; Van Leeuwen, P. W. N. M. Electronic Effect of Diphosphines on the Regioselectivity of the Palladium-Catalyzed Hydroesterification of Styrene. *Organometallics* **2006**, *25* (13), 3102–3104. https://doi.org/10.1021/om060121t.

(4) Zhu, Y.; Rawal, V. H. Palladium-Catalyzed C3-Benzylation of Indoles. *J. Am. Chem. Soc.* **2012**, *134* (1), 111–114. https://doi.org/10.1021/ja2095393.

(5) Shaw, L.; Somisara, D. M. U. K.; How, R. C.; Westwood, N. J.; Bruijnincx, P. C. A.; Weckhuysen, B. M.; Kamer, P. C. J. Electronic and Bite Angle Effects in Catalytic C–O Bond Cleavage of a Lignin Model Compound Using Ruthenium Xantphos Complexes. *Catal. Sci. Technol.* **2017**, *7* (3), 619–626. https://doi.org/10.1039/C6CY00518G.

(6) Ito, H.; Saito, T.; Miyahara, T.; Zhong, C.; Sawamura, M. Gold(I) Hydride Intermediate in Catalysis: Dehydrogenative Alcohol Silylation Catalyzed by Gold(I) Complex. *Organometallics* **2009**, *28* (16), 4829–4840. https://doi.org/10.1021/om900445w.

(7) Sheldrick, G. M. A Short History of SHELX. *Acta Crystallogr. Sect. A Found. Crystallogr.* **2008**, *64* (1), 112–122. https://doi.org/10.1107/S0108767307043930.

(8) Sheldrick, G. M. SHELXT – Integrated Space-Group and Crystal-Structure Determination. *Acta Crystallogr. Sect. A Found. Adv.* **2015**, *71* (1), 3–8. https://doi.org/10.1107/S2053273314026370.

(9) Sheldrick, G. M. Crystal Structure Refinement with SHELXL. *Acta Crystallogr. Sect.*

C Struct. Chem. **2015**, *71* (1), 3–8. https://doi.org/10.1107/S2053229614024218.

(10) Dolomanov, O. V.; Bourhis, L. J.; Gildea, R. J.; Howard, J. A. K.; Puschmann, H. OLEX2 : A Complete Structure Solution, Refinement and Analysis Program. *J. Appl. Crystallogr.* **2009**, *42* (2), 339–341. https://doi.org/10.1107/S0021889808042726.

NMR spectra

Ligand synthesis

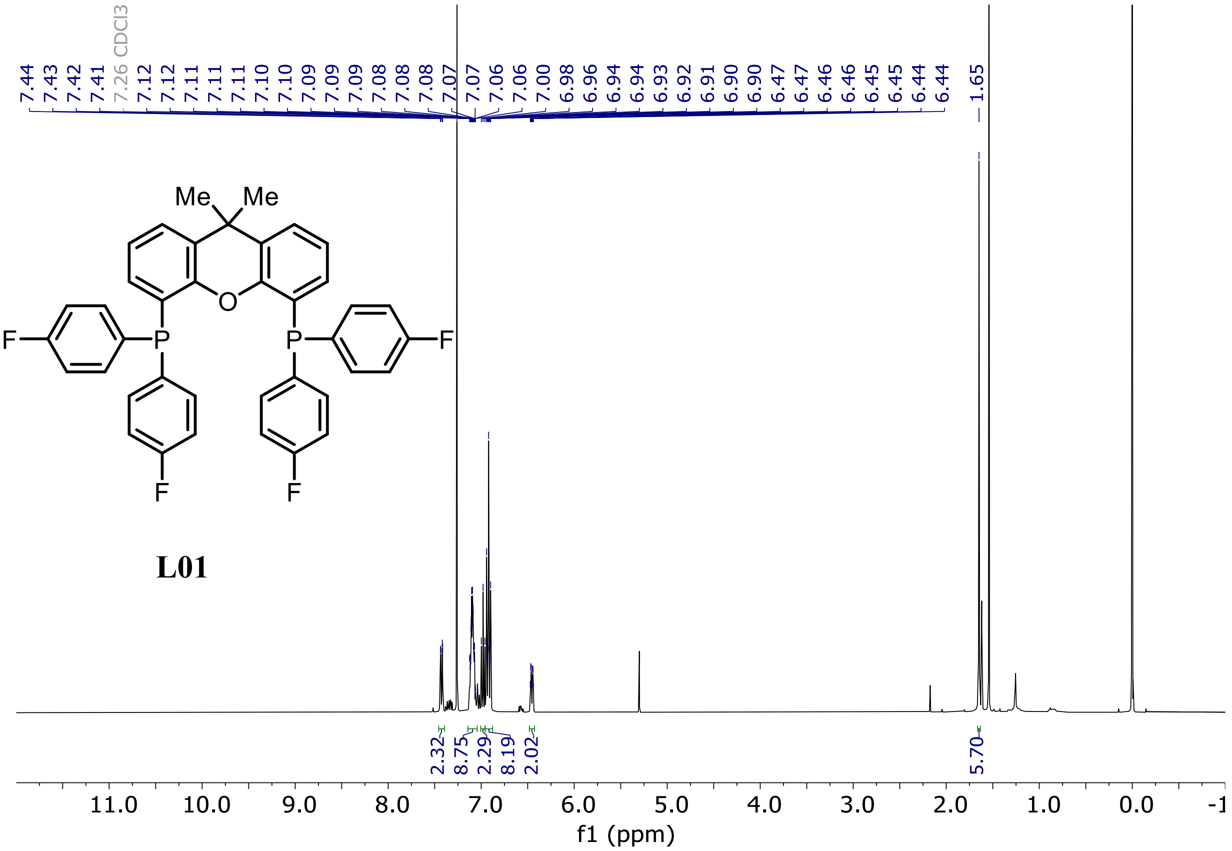

L01

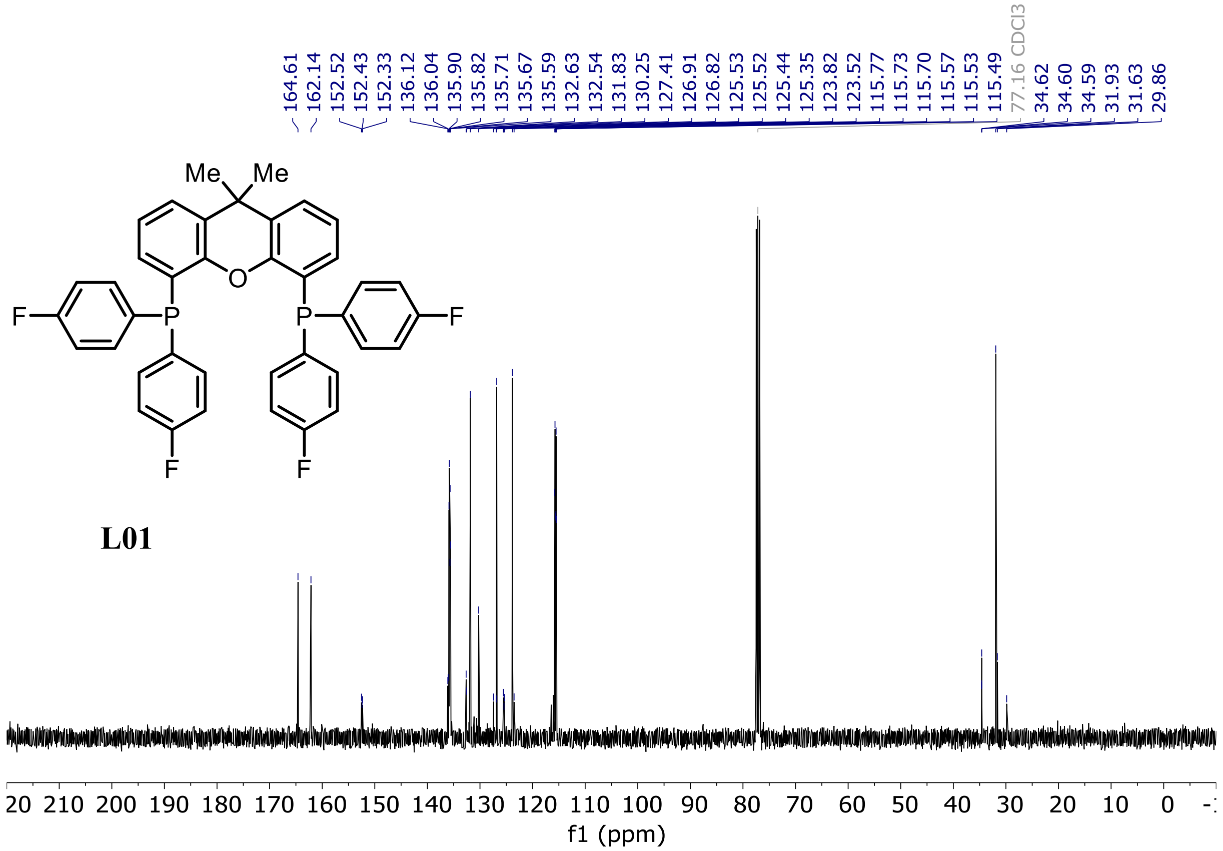

L01

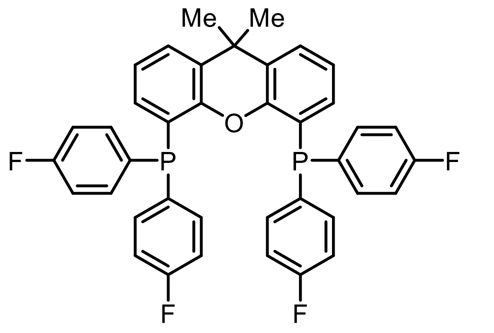
Me
Me
O
F
P
F
P
F
F

L01

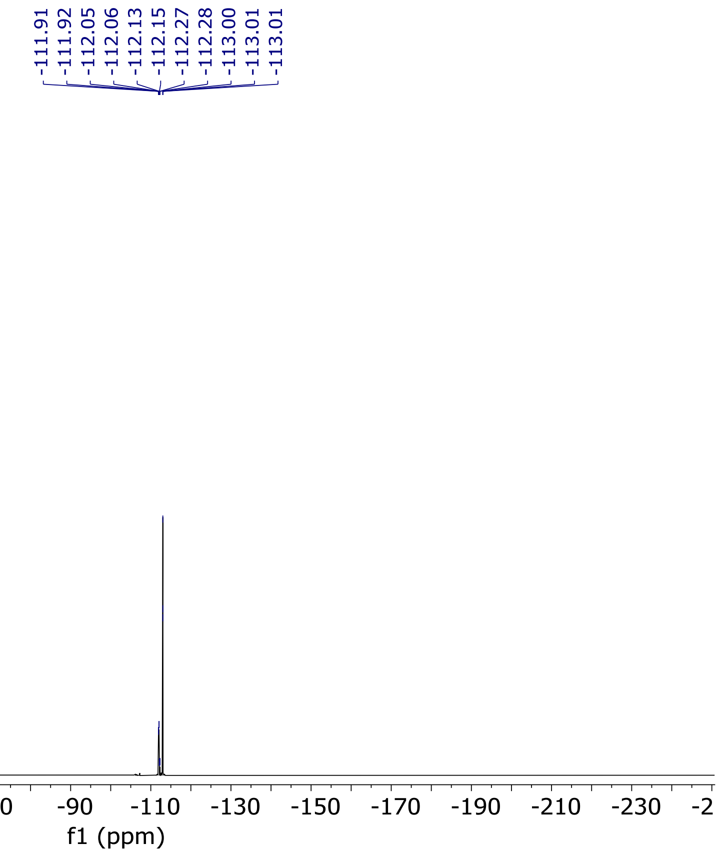
-111.91
-111.92
-112.05
-112.06
-112.13
-112.15
-112.27
-112.28
-113.00
-113.01
-113.01
30 10 -10 -30 -50 -70 -90 -110 -130 -150 -170 -190 -210 -230
f1 (ppm)

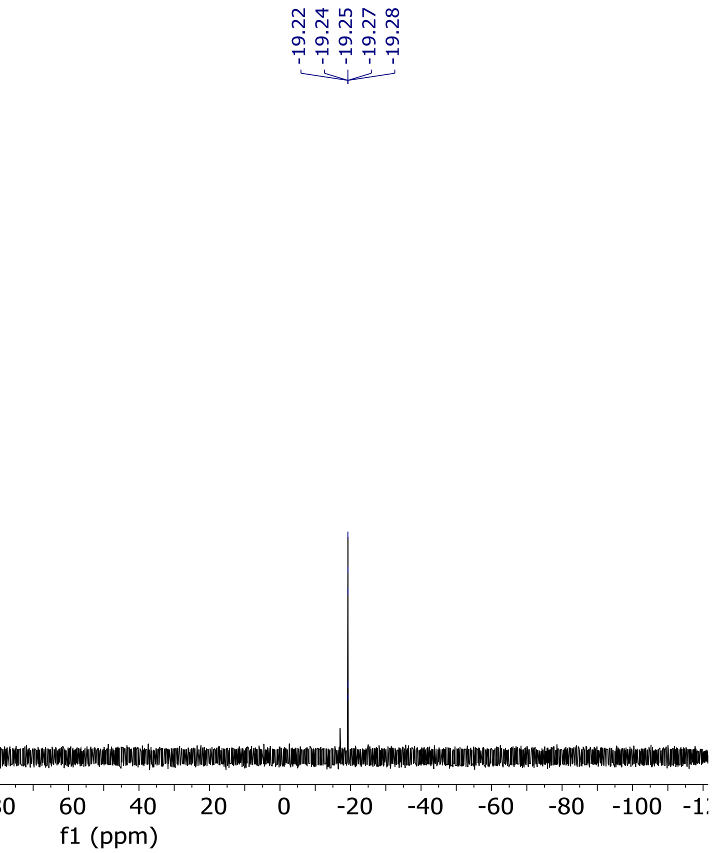
-19.22
-19.24
-19.25
-19.27
-19.28
200 180 160 140 120 100 80 60 40 20 0 -20 -40 -60 -80 -100
f1 (ppm)

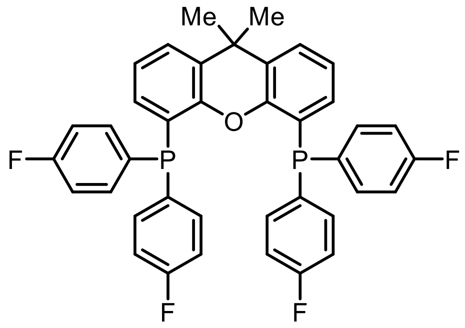
Me
Me
O
F
P
F
P
F
F

L01

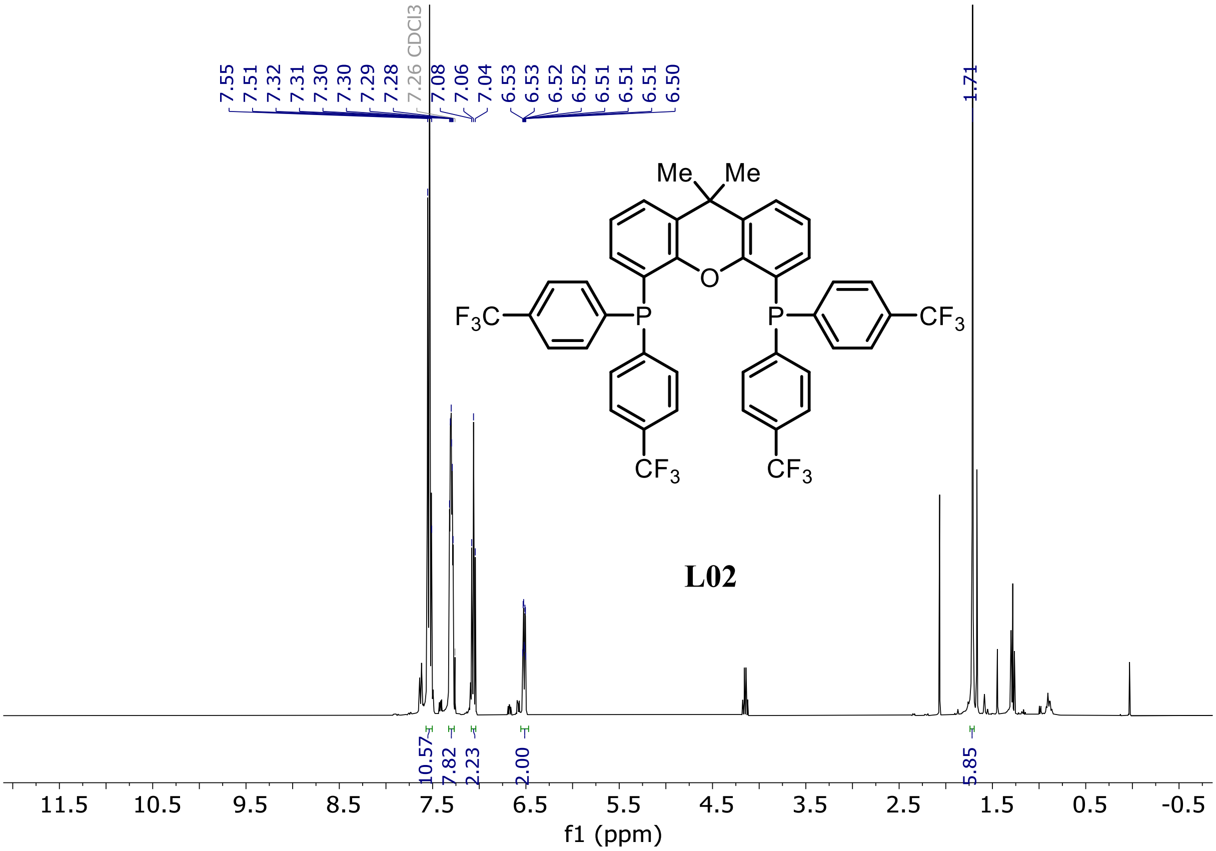
7.55 7.51 7.32 7.31 7.30 7.30 7.29 7.28 7.26 CDCl3 7.08 7.06 7.04 6.53 6.53 6.52 6.52 6.51 6.51 6.50
1.71
Me Me
O
F3C P P CF3
CF3 CF3
L02
10.57 7.82 2.23 2.00 5.85
11.5 10.5 9.5 8.5 7.5 6.5 5.5 4.5 3.5 2.5 1.5 0.5 -0.5
f1 (ppm)

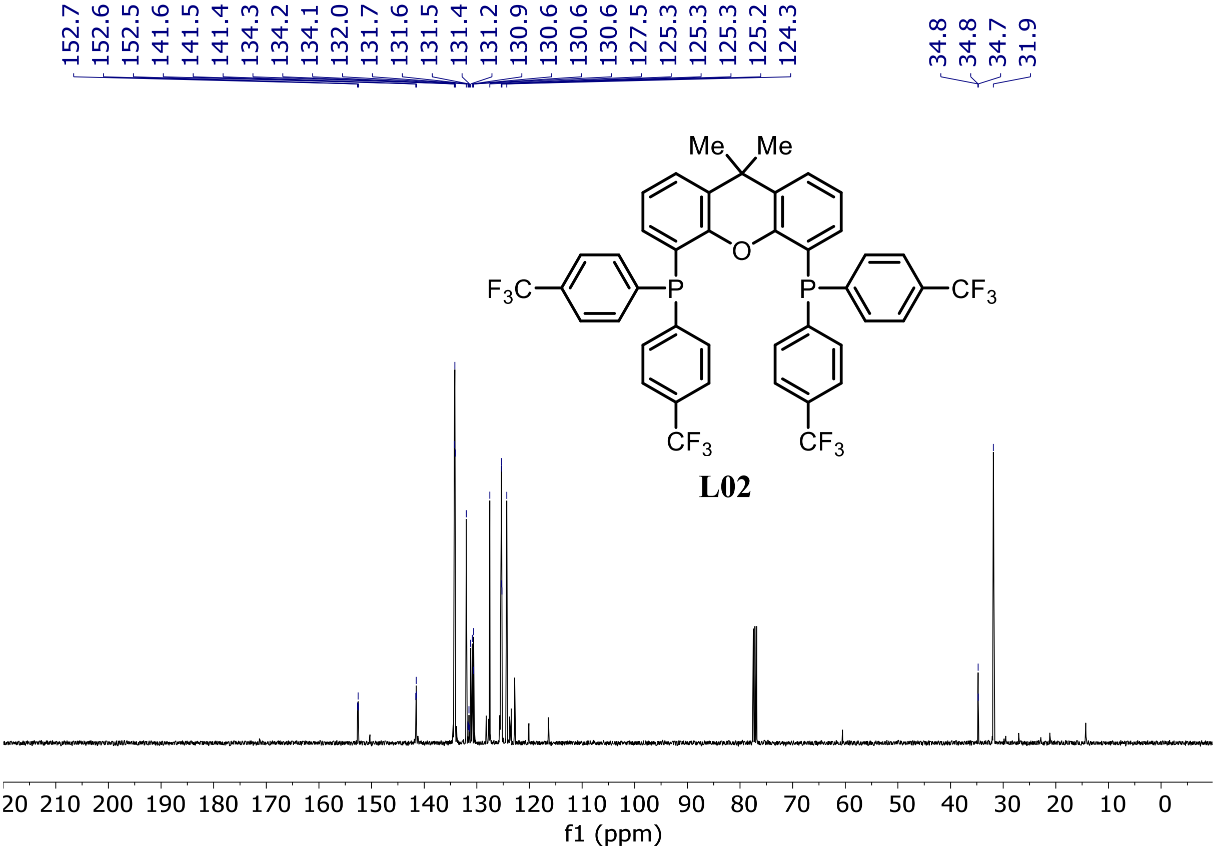
152.7 152.6 152.5 141.6 141.5 141.4 134.3 134.2 134.1 132.0 131.7 131.6 131.5 131.4 131.2 130.9 130.6 130.6 130.6 127.5 125.3 125.3 125.3 125.2 124.3
34.8 34.8 34.7 31.9
Me Me
O
F3C P P CF3
CF3 CF3
L02
20 210 200 190 180 170 160 150 140 130 120 110 100 90 80 70 60 50 40 30 20 10 0
f1 (ppm)

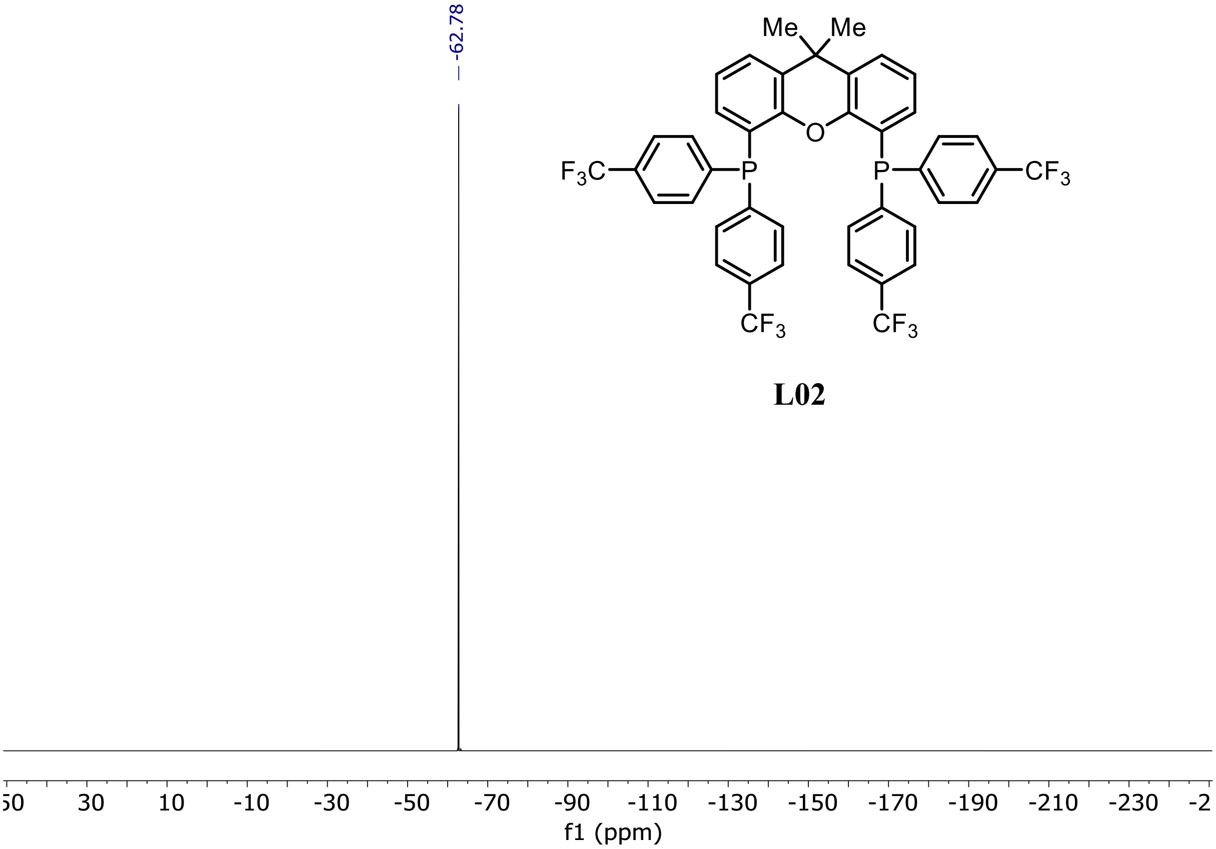
-62.78
Me
Me
O
P
P
F3C
CF3
CF3
CF3
L02
50
30
10
-10
-30
-50
-70
-90
-110
-130
-150
-170
-190
-210
-230
-2
f1 (ppm)

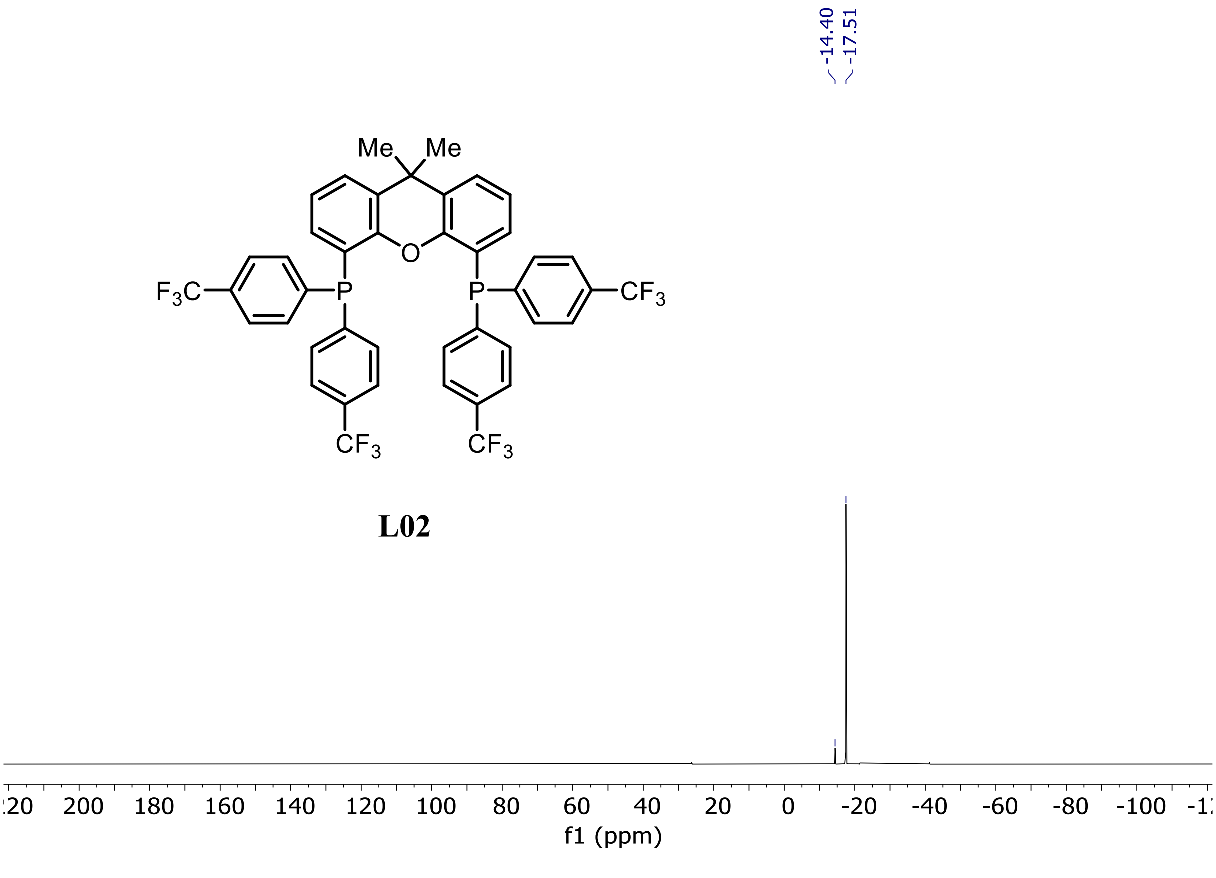
-14.40
-17.51
Me
Me
O
P
P
F3C
CF3
CF3
CF3
L02
220
200
180
160
140
120
100
80
60
40
20
0
-20
-40
-60
-80
-100
-1
f1 (ppm)

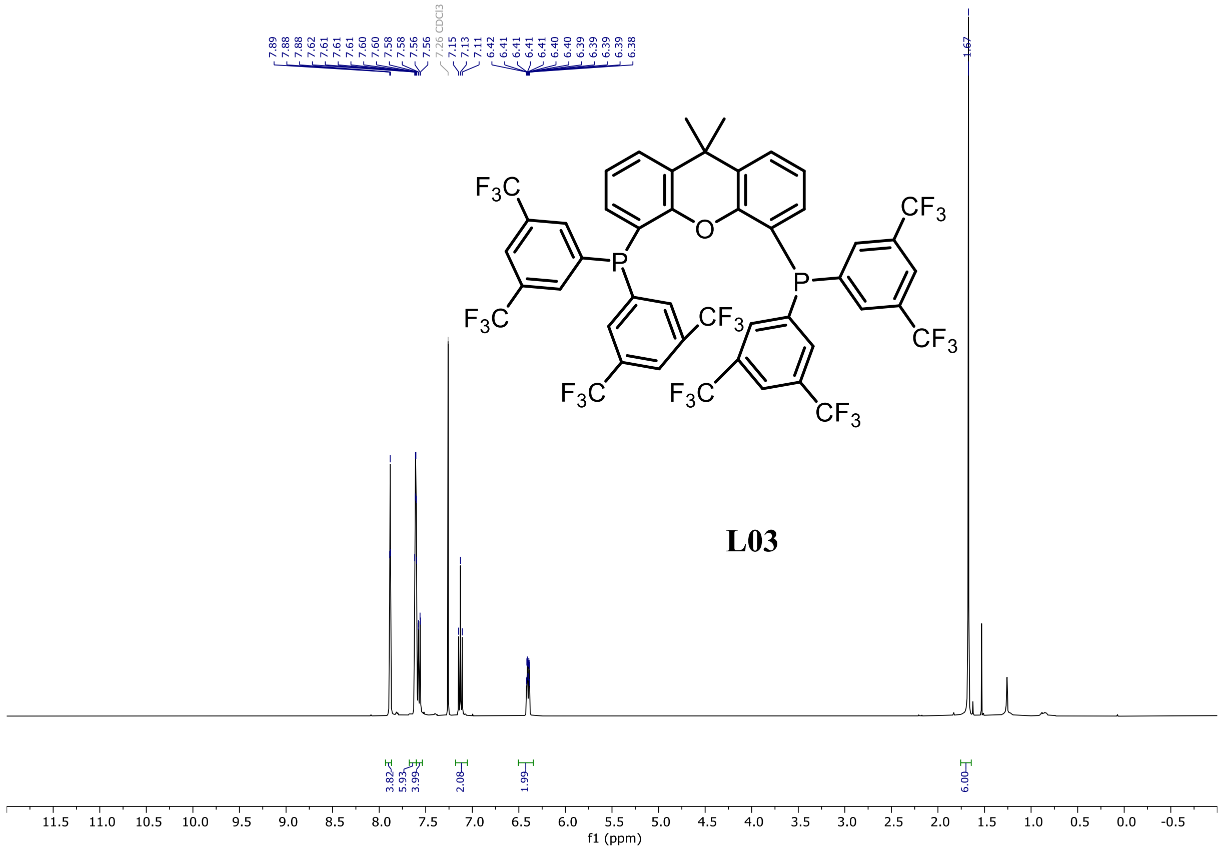
7.89 7.88 7.88 7.62 7.61 7.61 7.61 7.60 7.58 7.58 7.56 7.56
7.26 CDCl3
7.15 7.13 7.11
6.42 6.41 6.41 6.41 6.40 6.39 6.39 6.39 6.39 6.38
1.67
3.82 5.93 3.99 2.08 1.99 6.00
11.5 11.0 10.5 10.0 9.5 9.0 8.5 8.0 7.5 7.0 6.5 6.0 5.5 5.0 4.5 4.0 3.5 3.0 2.5 2.0 1.5 1.0 0.5 0.0 -0.5
f1 (ppm)

L03

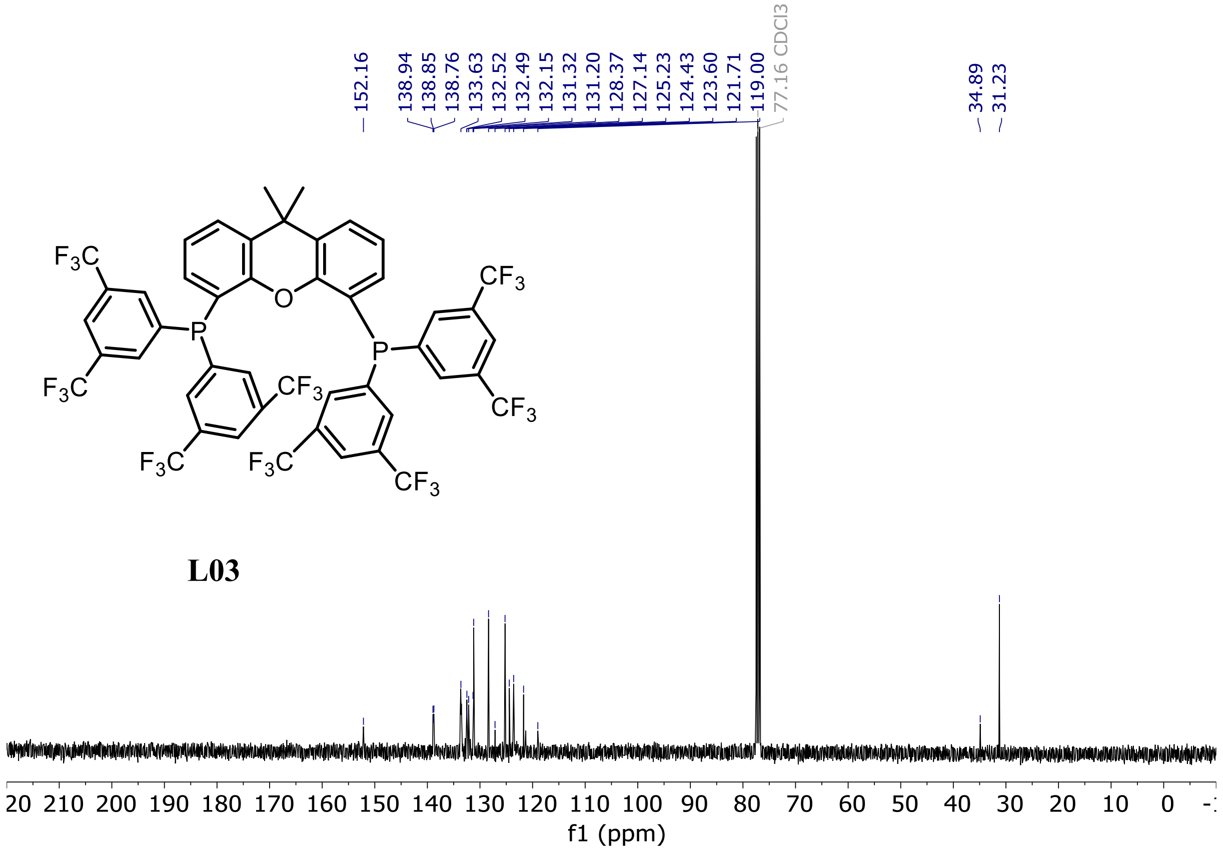
152.16
138.94 138.85 138.76 133.63 132.52 132.49 132.15 131.32 131.20 128.37 127.14 125.23 124.43 123.60 121.71 119.00
77.16 CDCl3
34.89 31.23
20 210 200 190 180 170 160 150 140 130 120 110 100 90 80 70 60 50 40 30 20 10 0
f1 (ppm)

L03

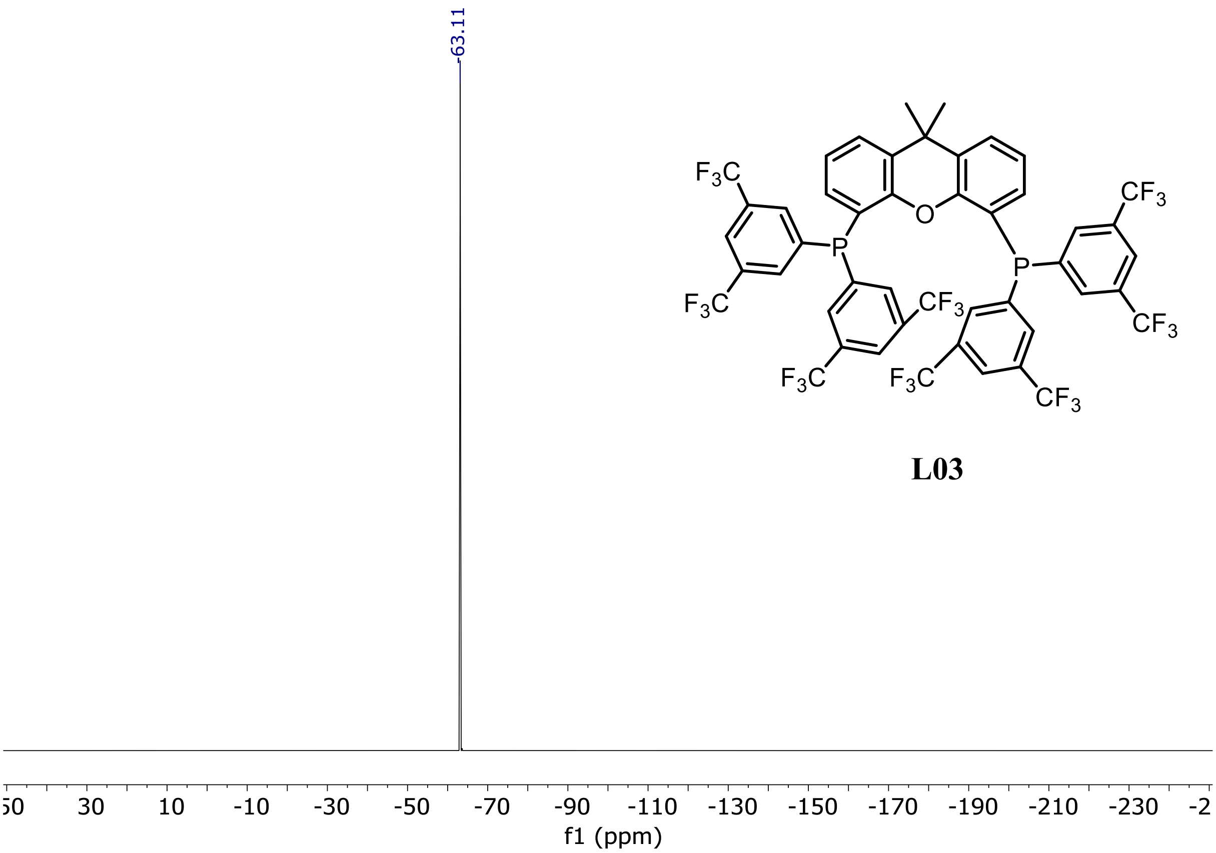
-63.11
F3C
CF3
F3C
CF3
CF3
F3C
F3C
CF3
L03
50 30 10 -10 -30 -50 -70 -90 -110 -130 -150 -170 -190 -210 -230 -2
f1 (ppm)

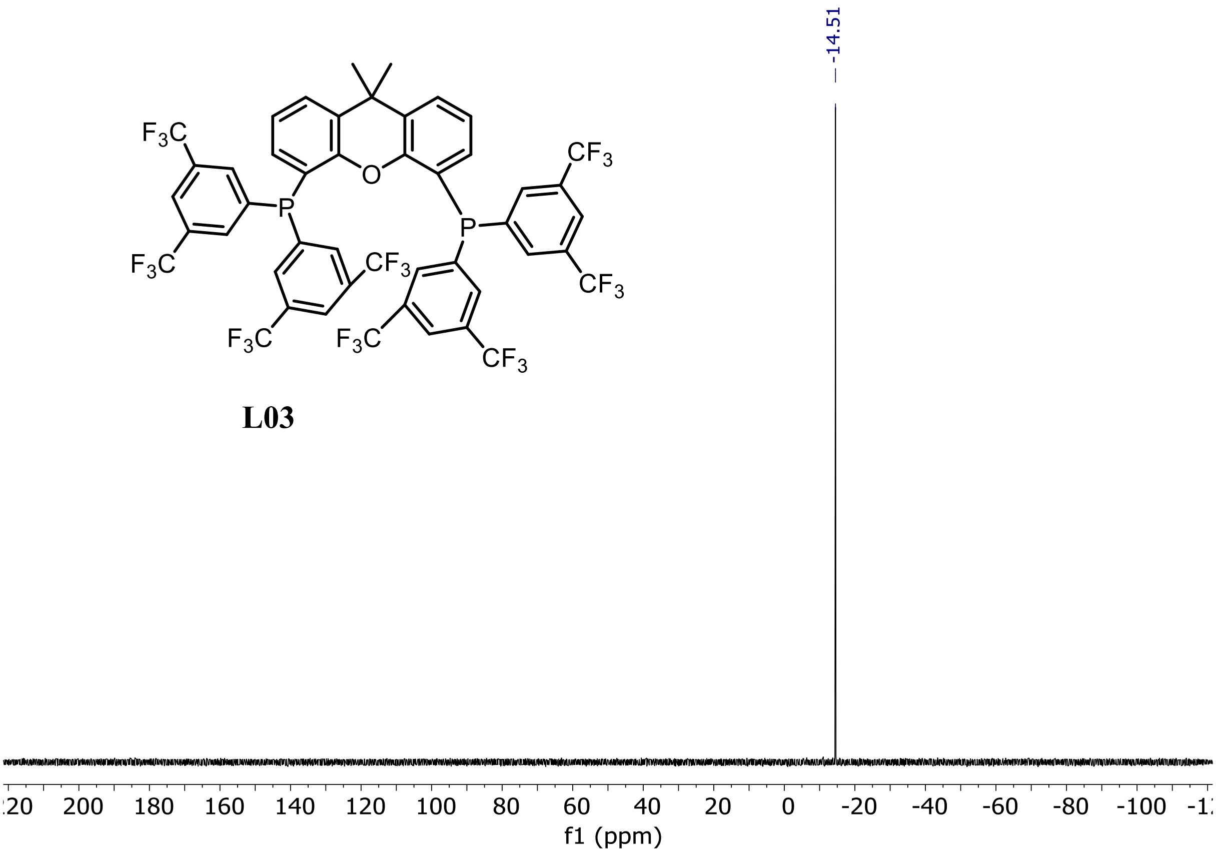
-14.51
F3C
CF3
F3C
CF3
CF3
F3C
F3C
CF3
L03
220 200 180 160 140 120 100 80 60 40 20 0 -20 -40 -60 -80 -100 -1
f1 (ppm)

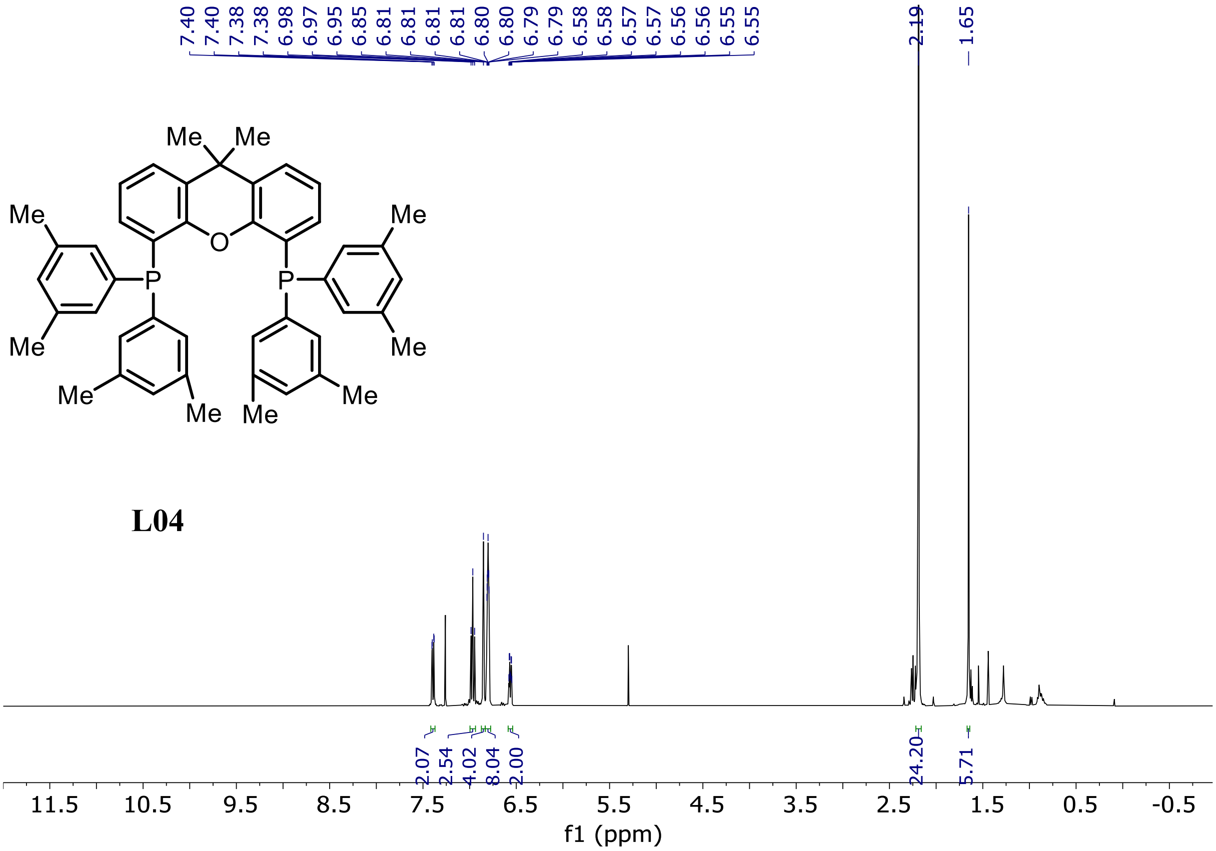
7.40
7.40
7.38
7.38
6.98
6.97
6.95
6.85
6.81
6.81
6.81
6.80
6.80
6.79
6.79
6.58
6.58
6.57
6.57
6.56
6.56
6.55
6.55
2.19
1.65
Me
Me
P
O
2.07
2.54
4.02
8.04
2.00
24.20
5.71
11.5 10.5 9.5 8.5 7.5 6.5 5.5 4.5 3.5 2.5 1.5 0.5 -0.5
f1 (ppm)

L04

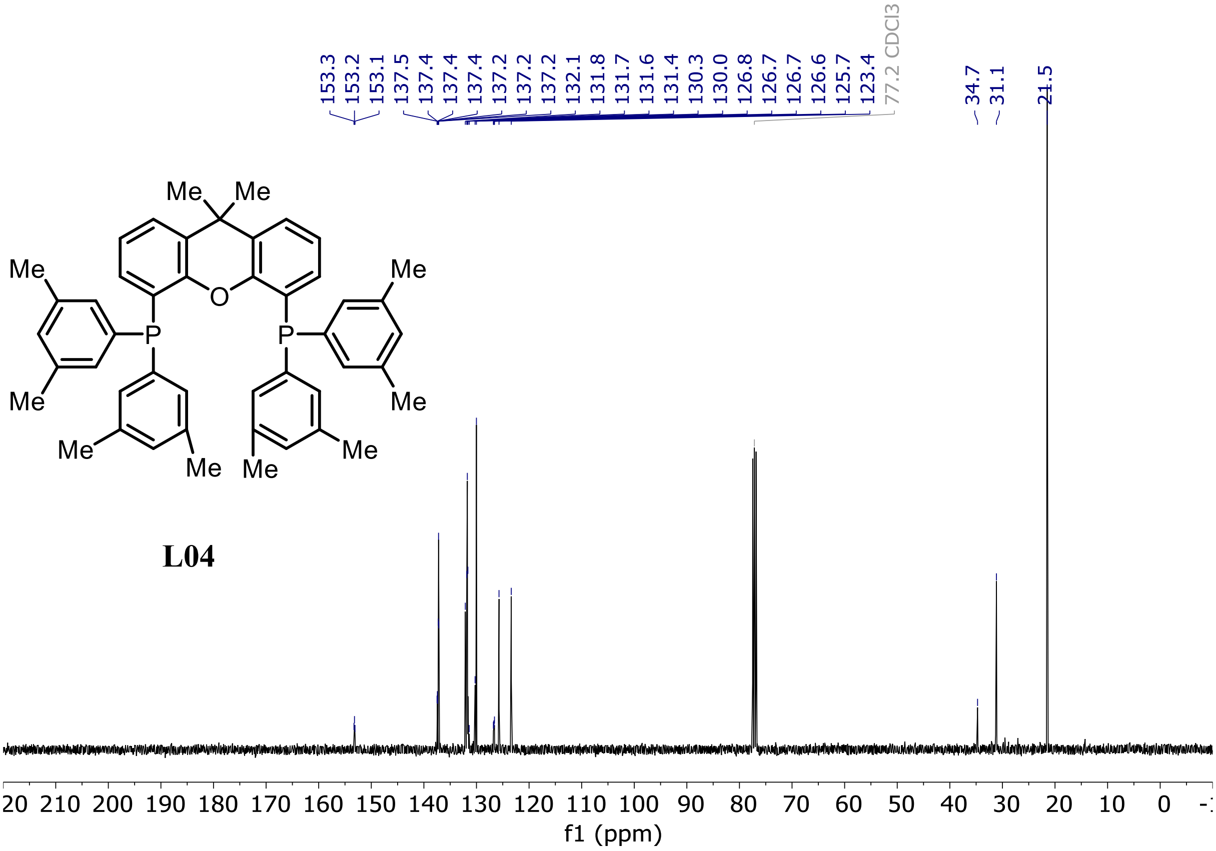
153.3
153.2
153.1
137.5
137.4
137.4
137.4
137.2
137.2
137.2
132.1
131.8
131.7
131.6
131.4
130.3
130.0
126.8
126.7
126.7
126.6
125.7
123.4
77.2 CDCl3
34.7
31.1
21.5
Me
Me
P
O
20 210 200 190 180 170 160 150 140 130 120 110 100 90 80 70 60 50 40 30 20 10 0
f1 (ppm)

L04

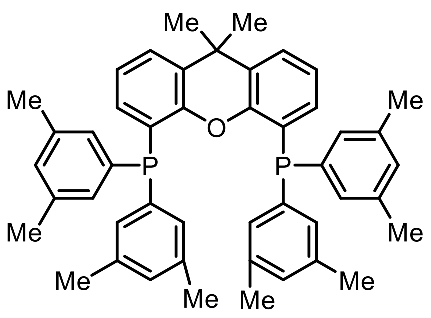

L04

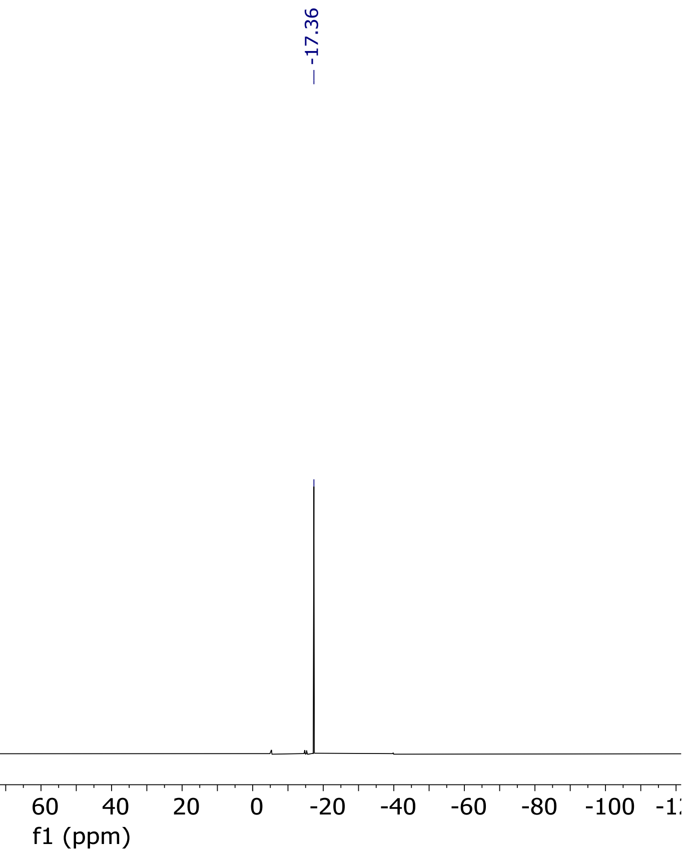
-17.36
20 200 180 160 140 120 100 80 60 40 20 0 -20 -40 -60 -80 -100 -1
f1 (ppm)

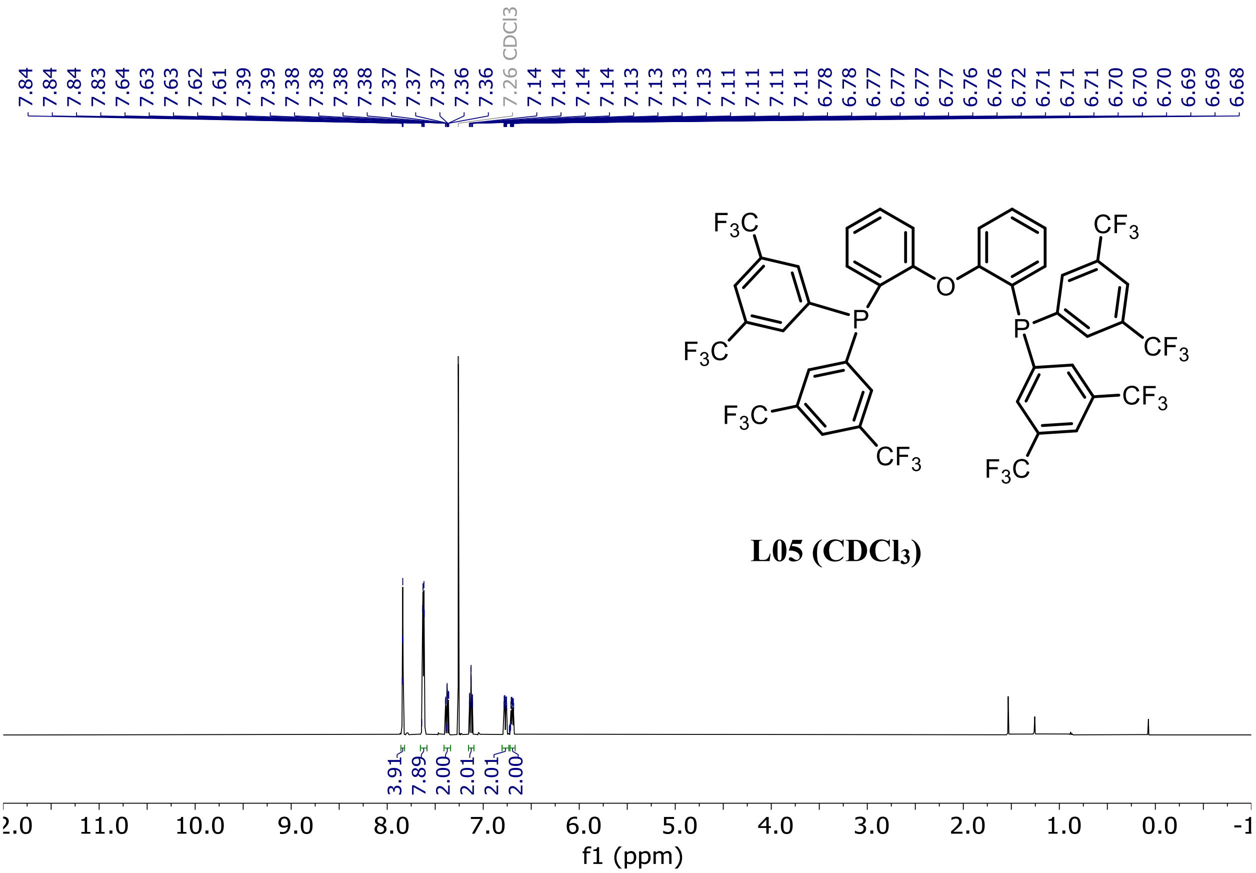
7.84 7.84 7.84 7.83 7.64 7.63 7.63 7.62 7.61 7.39 7.39 7.38 7.38 7.38 7.38 7.37 7.37 7.37 7.36 7.36 7.26 CDCl3 7.14 7.14 7.14 7.14 7.13 7.13 7.13 7.13 7.11 7.11 7.11 7.11 6.78 6.78 6.77 6.77 6.77 6.77 6.76 6.76 6.72 6.71 6.71 6.70 6.70 6.70 6.69 6.69 6.68
L05 (CDCl3)
3.91 7.89 2.00 2.01 2.01 2.00
f1 (ppm)

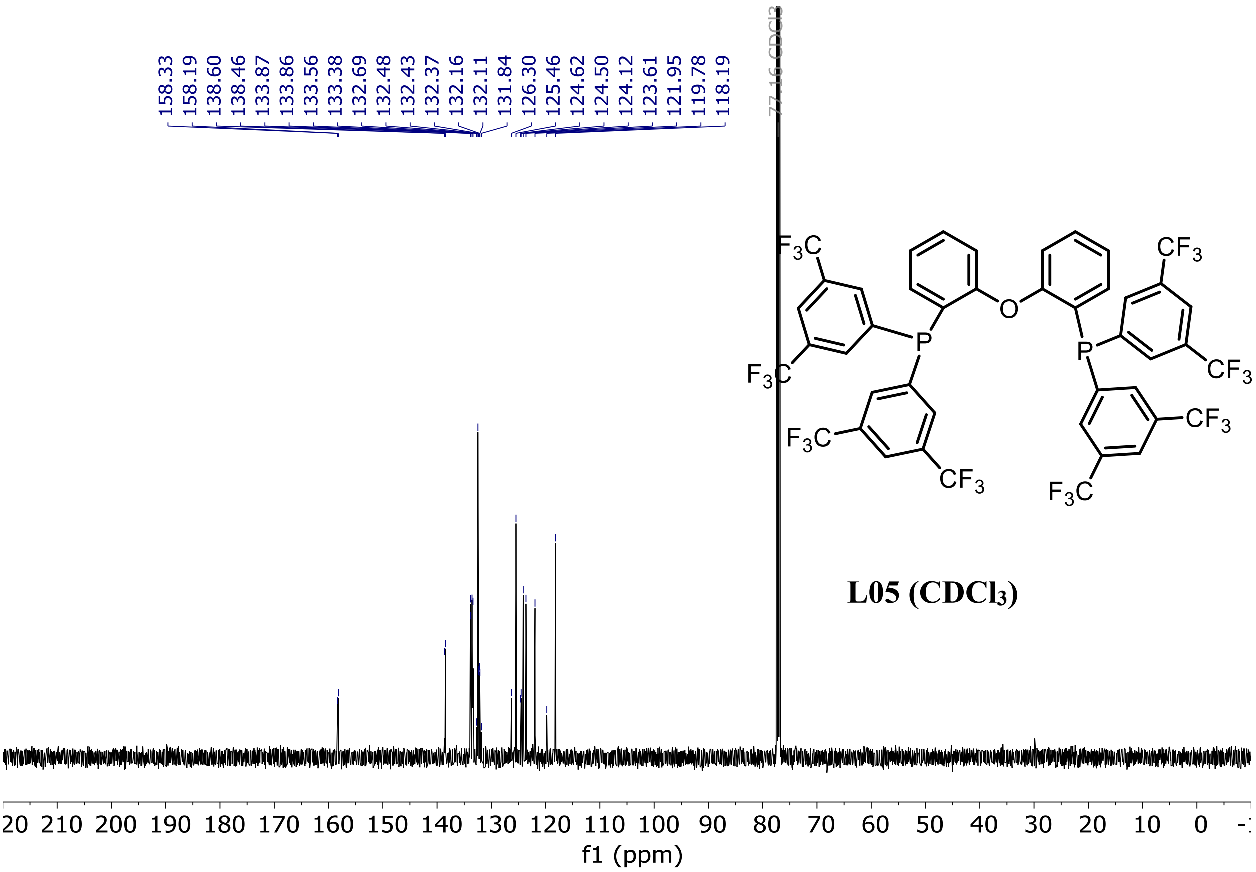
158.33 158.19 138.60 138.46 133.87 133.86 133.56 133.38 132.69 132.48 132.43 132.37 132.16 132.11 131.84 126.30 125.46 124.62 124.50 124.12 123.61 121.95 119.78 118.19
77.16 CDCl3
L05 (CDCl3)
f1 (ppm)

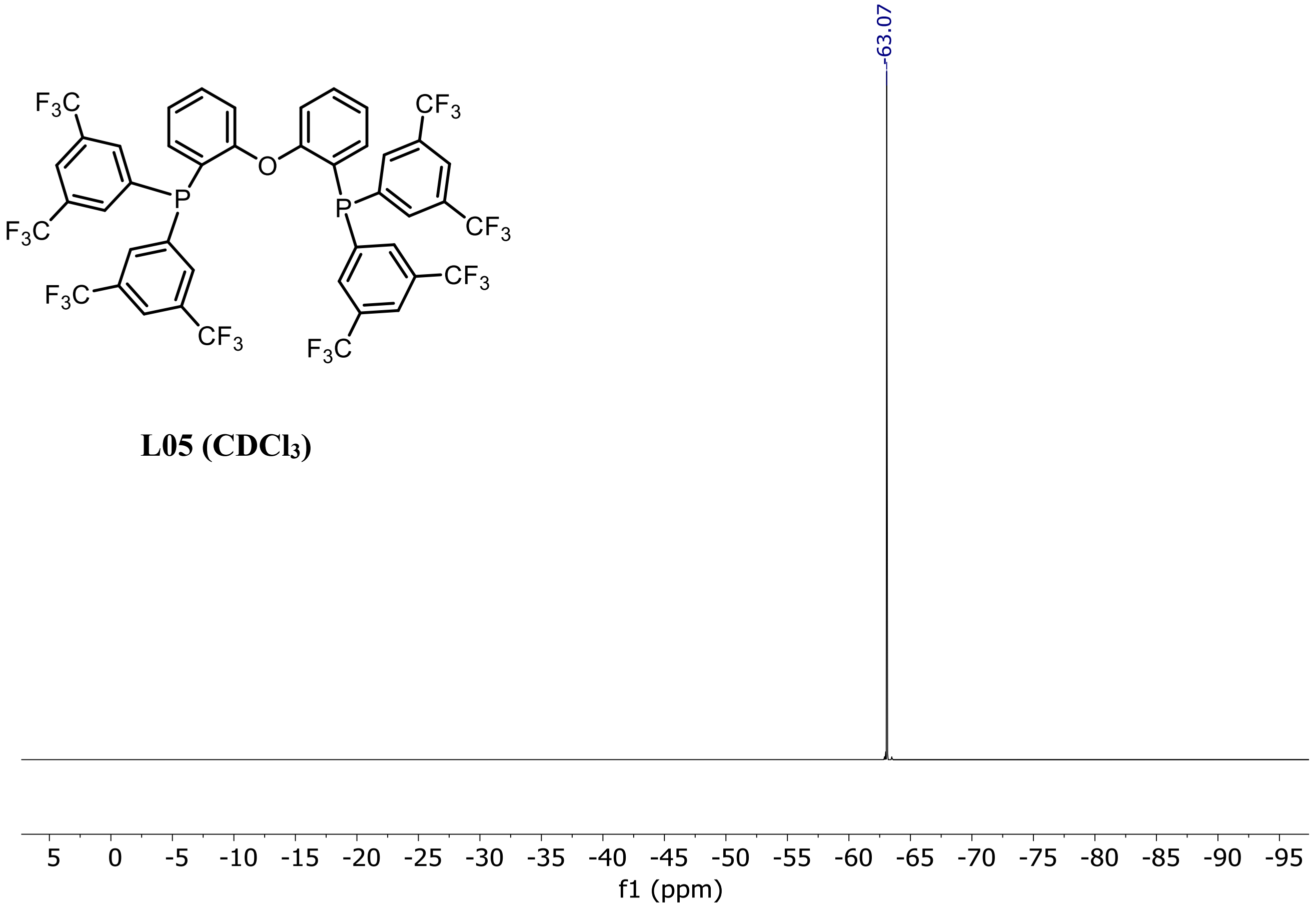
-63.07
F3C
CF3
F3C
CF3
F3C
CF3
CF3
F3C
P
O
P
L05 (CDCl3)
5 0 -5 -10 -15 -20 -25 -30 -35 -40 -45 -50 -55 -60 -65 -70 -75 -80 -85 -90 -95
f1 (ppm)

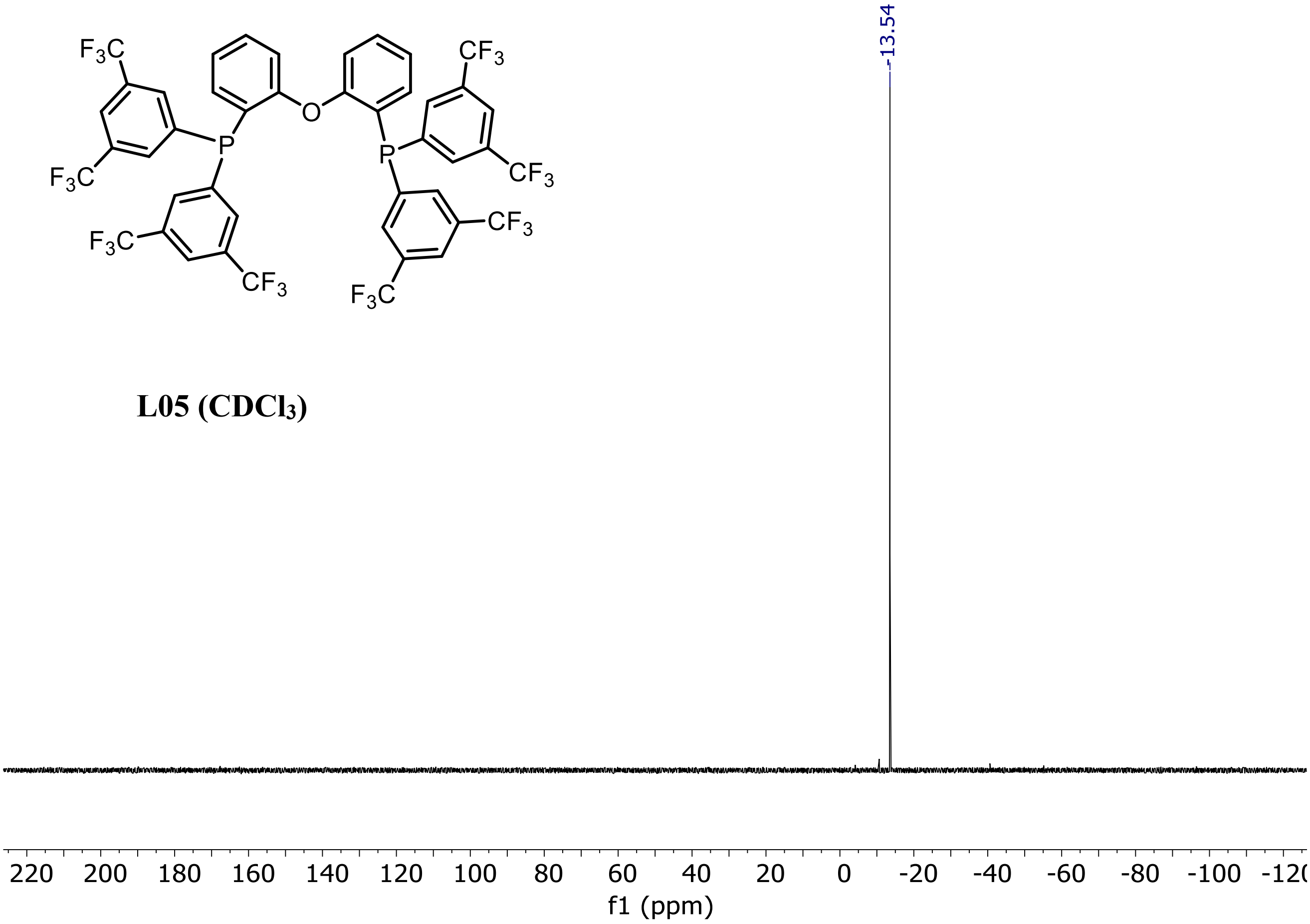
-13.54
F3C
CF3
F3C
CF3
F3C
CF3
CF3
F3C
P
O
P
L05 (CDCl3)
220 200 180 160 140 120 100 80 60 40 20 0 -20 -40 -60 -80 -100 -12(
f1 (ppm)

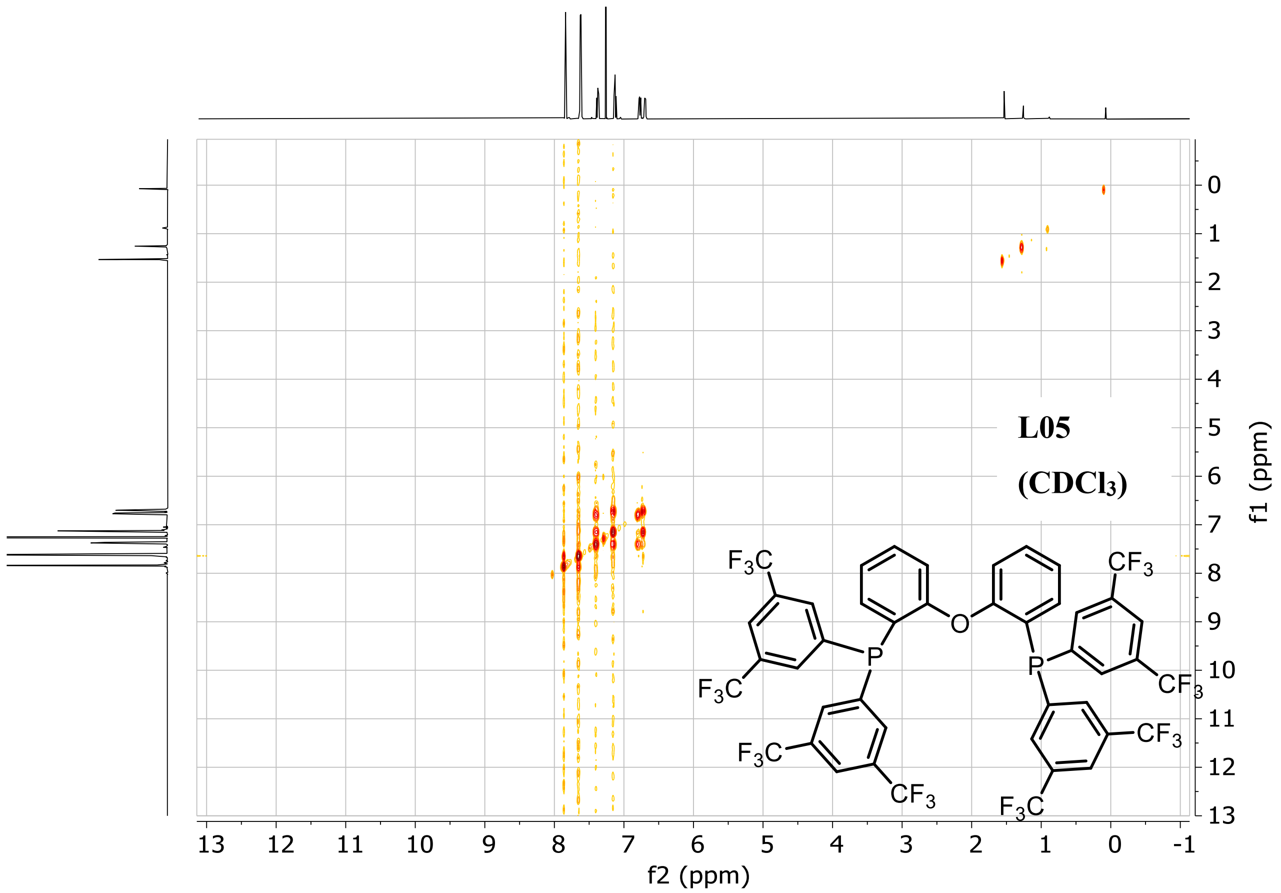
L05
(CDCl3)
f1 (ppm)
f2 (ppm)
F3C
CF3
O
P

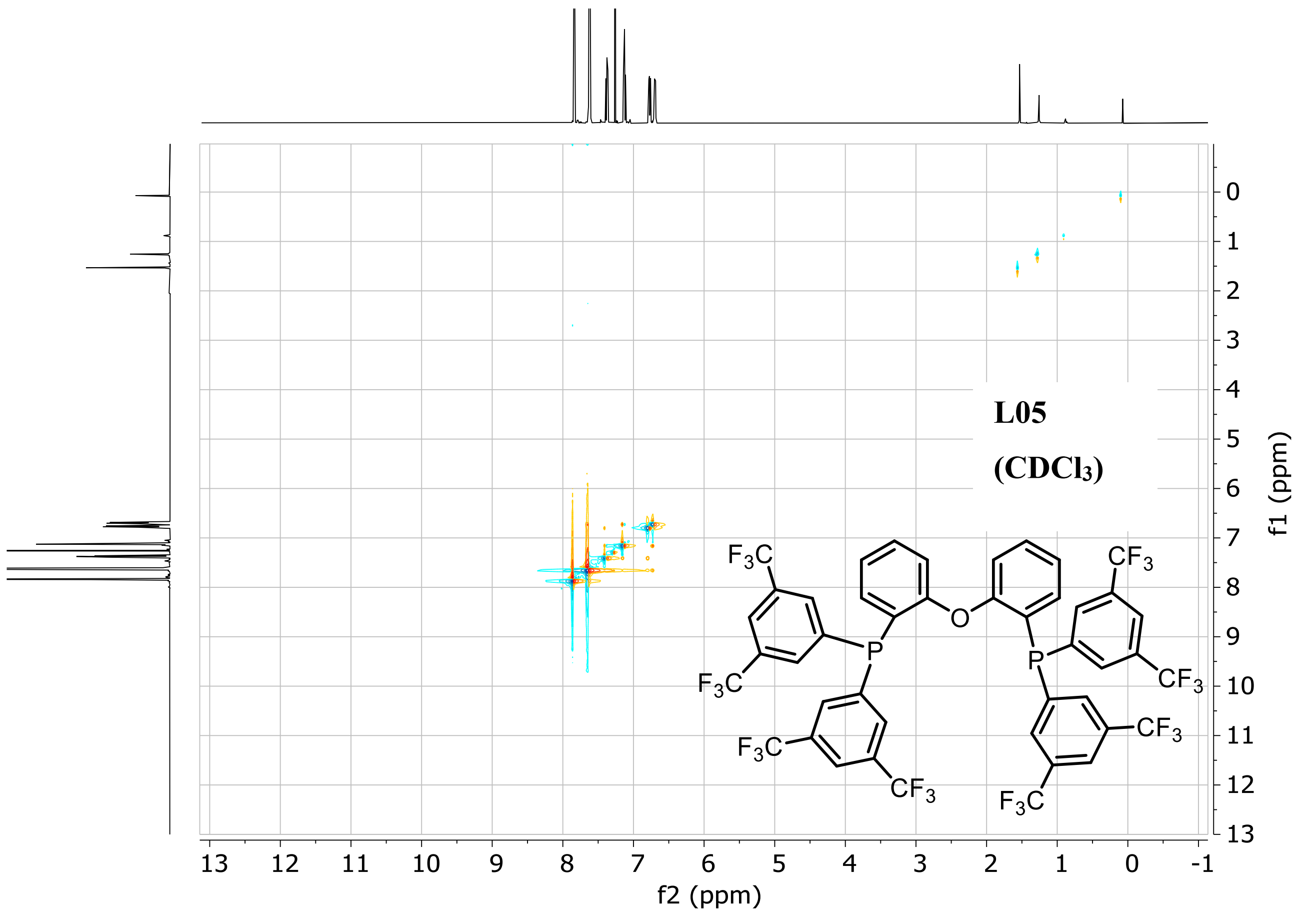
L05
(CDCl3)
f1 (ppm)
f2 (ppm)
F3C
CF3
O
P

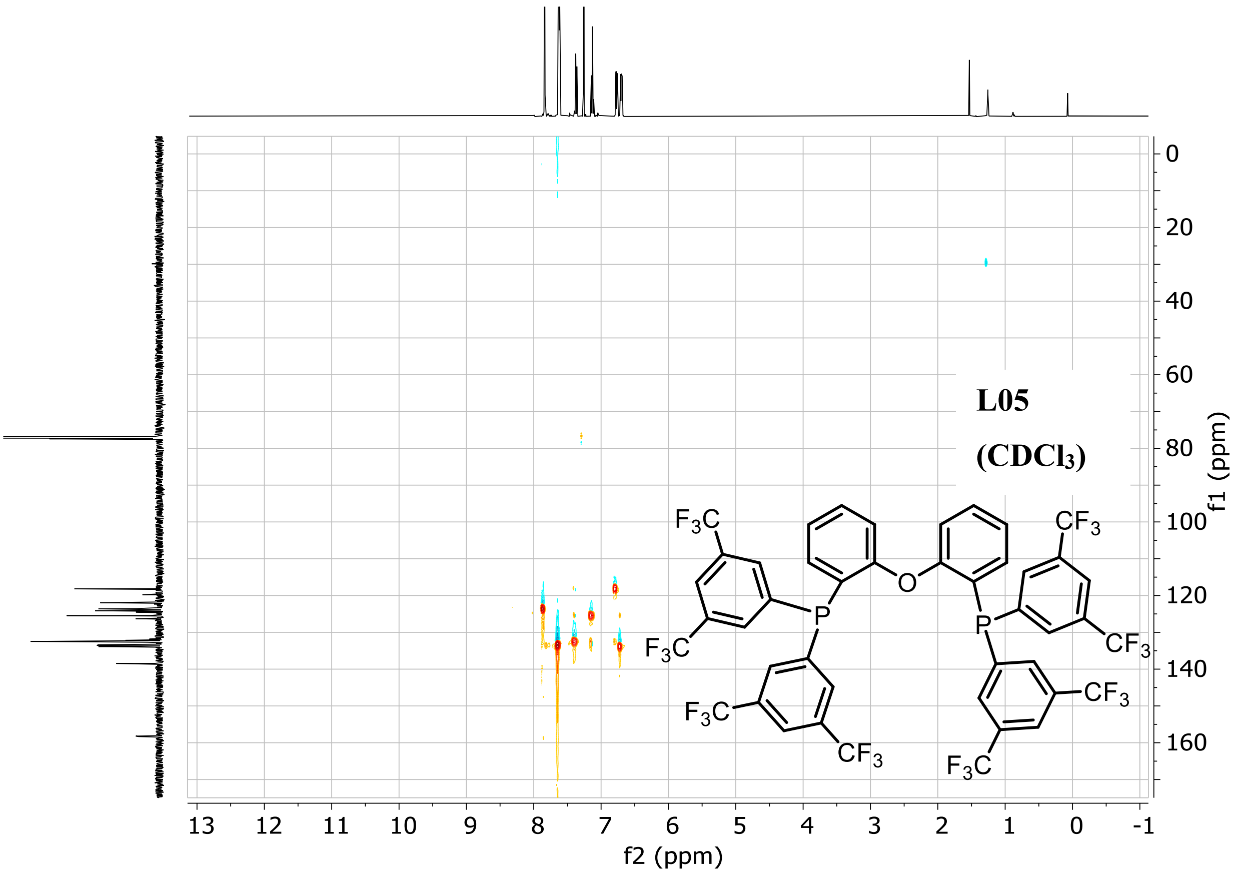

L05
(CDCl3)
f2 (ppm)
f1 (ppm)

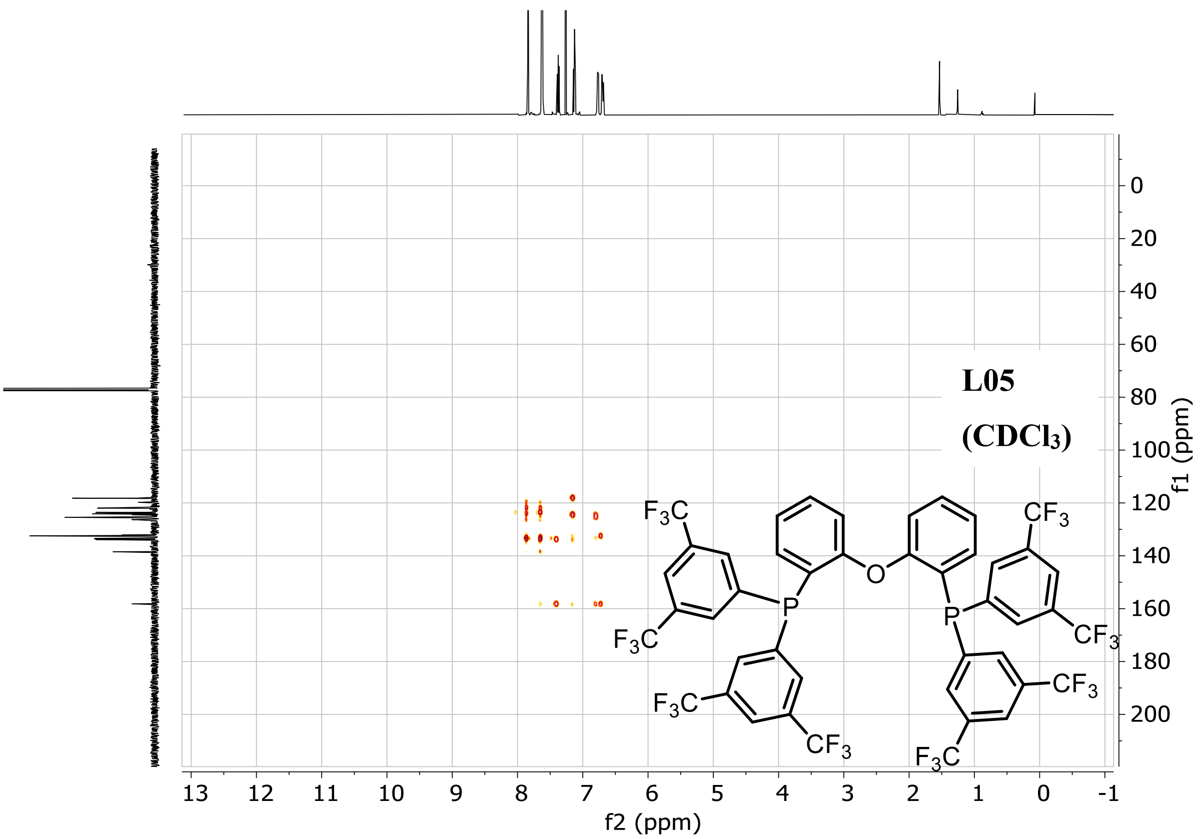

L05
(CDCl3)
f2 (ppm)
f1 (ppm)

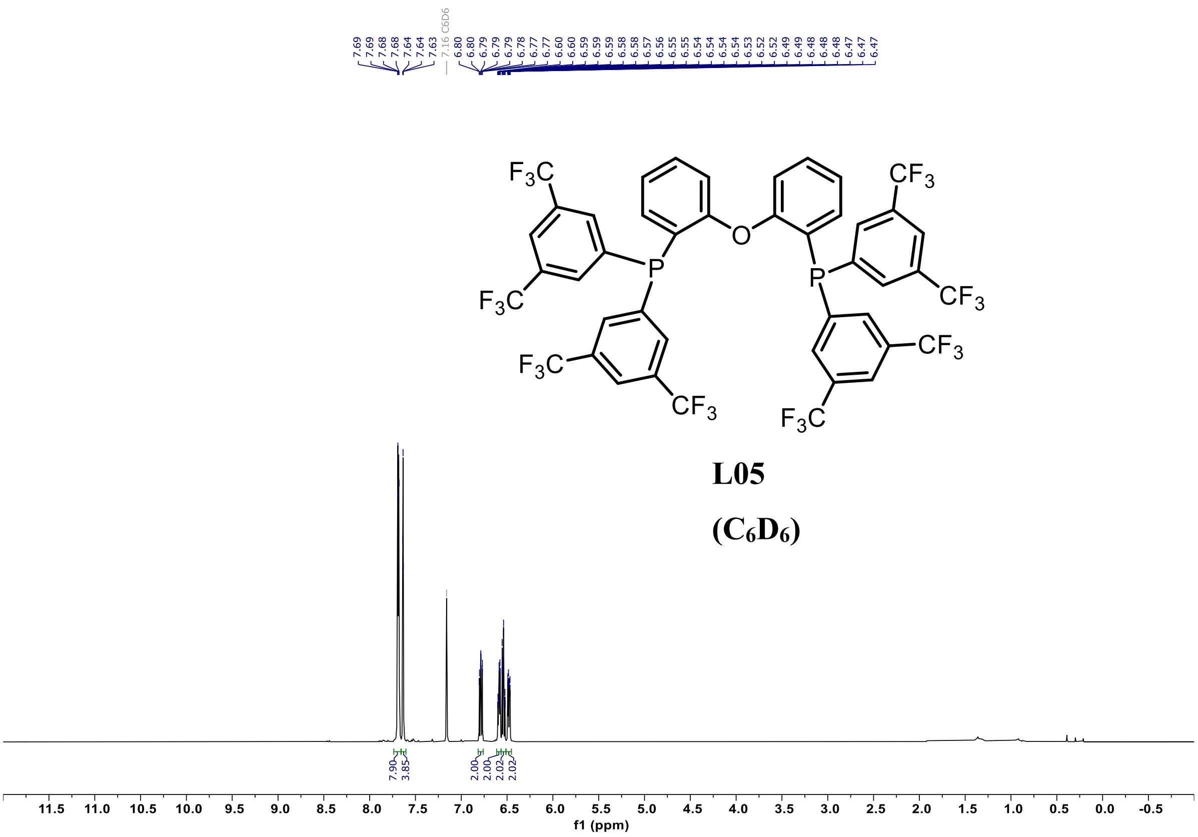
L05
(C_6D_6)
f1 (ppm)

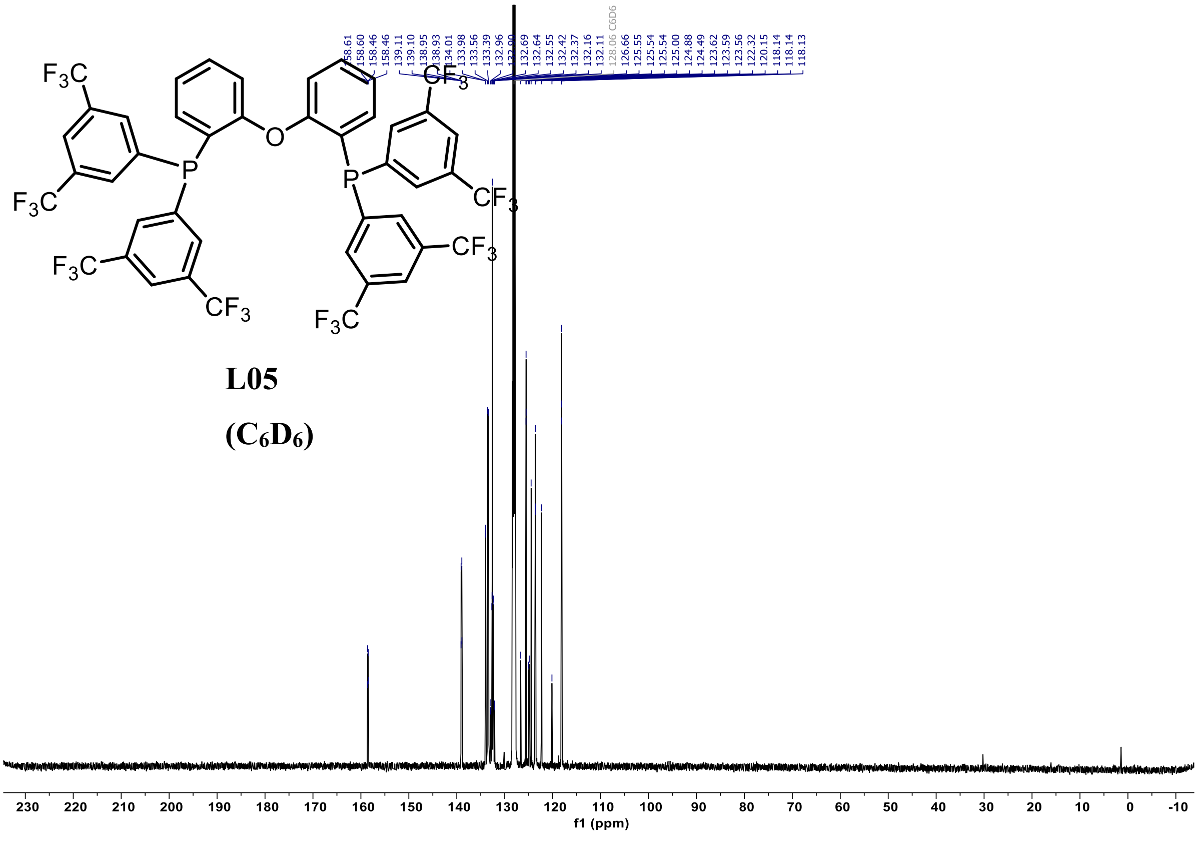
L05
(C_6D_6)
f1 (ppm)

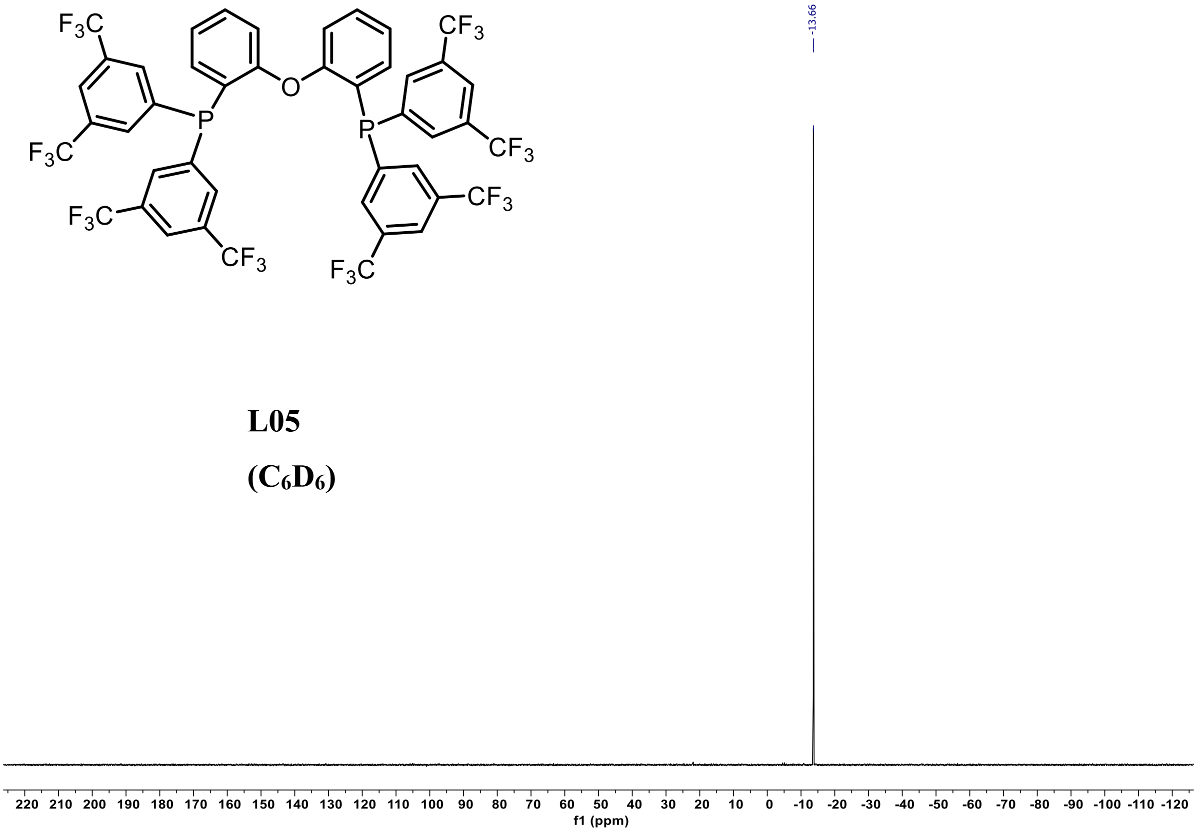

-13.66
F3C
CF3
O
P
L05
(C6D6)
220 210 200 190 180 170 160 150 140 130 120 110 100 90 80 70 60 50 40 30 20 10 0 -10 -20 -30 -40 -50 -60 -70 -80 -90 -100 -110 -120
f1 (ppm)

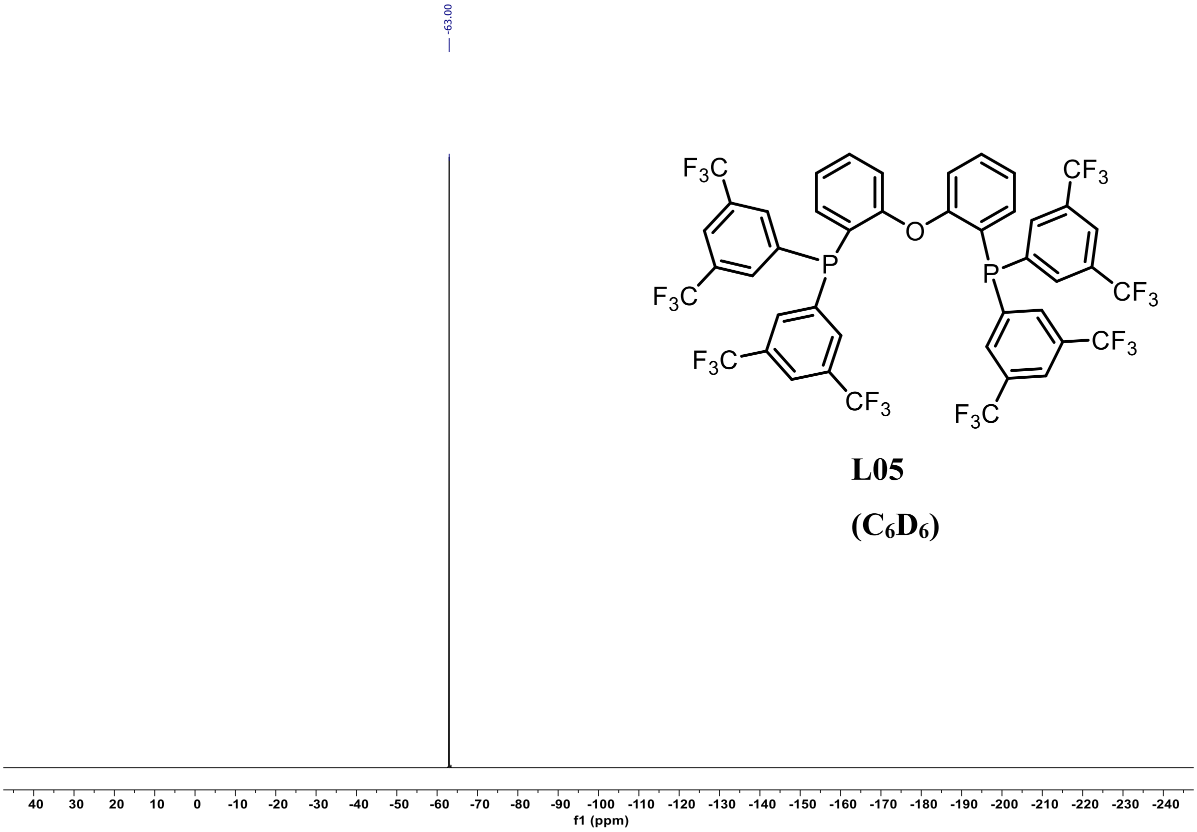

-63.00
F3C
CF3
O
P
L05
(C6D6)
40 30 20 10 0 -10 -20 -30 -40 -50 -60 -70 -80 -90 -100 -110 -120 -130 -140 -150 -160 -170 -180 -190 -200 -210 -220 -230 -240
f1 (ppm)

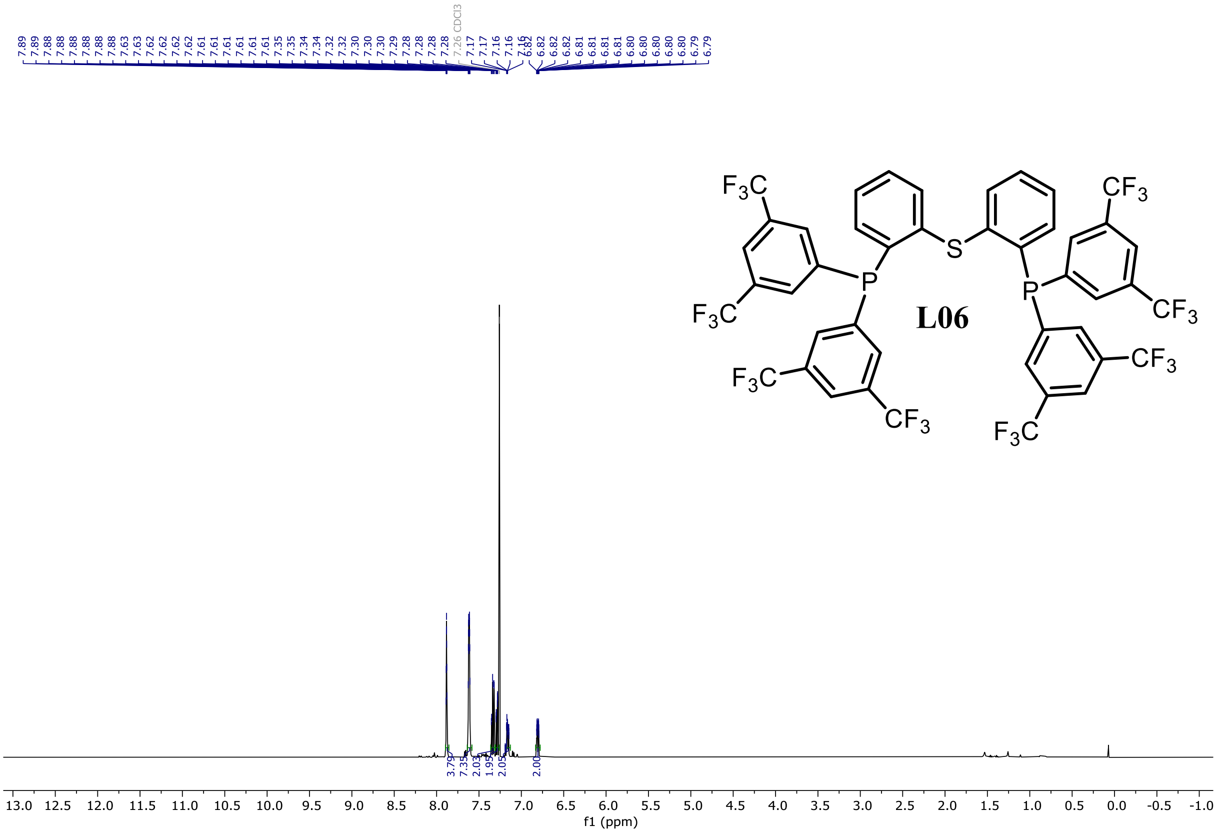

CDCl3
L06
3.79
7.35
2.03
1.95
2.05
2.00
f1 (ppm)

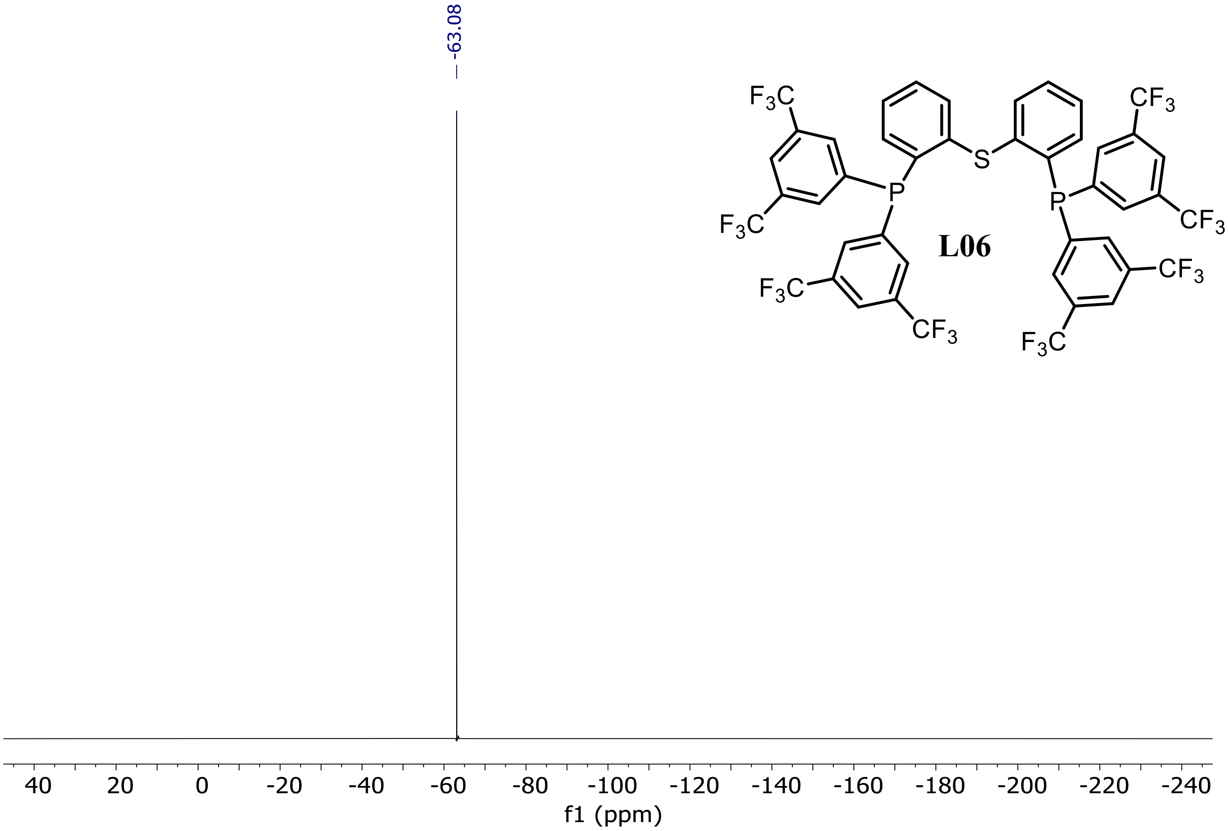

-63.08
L06
f1 (ppm)

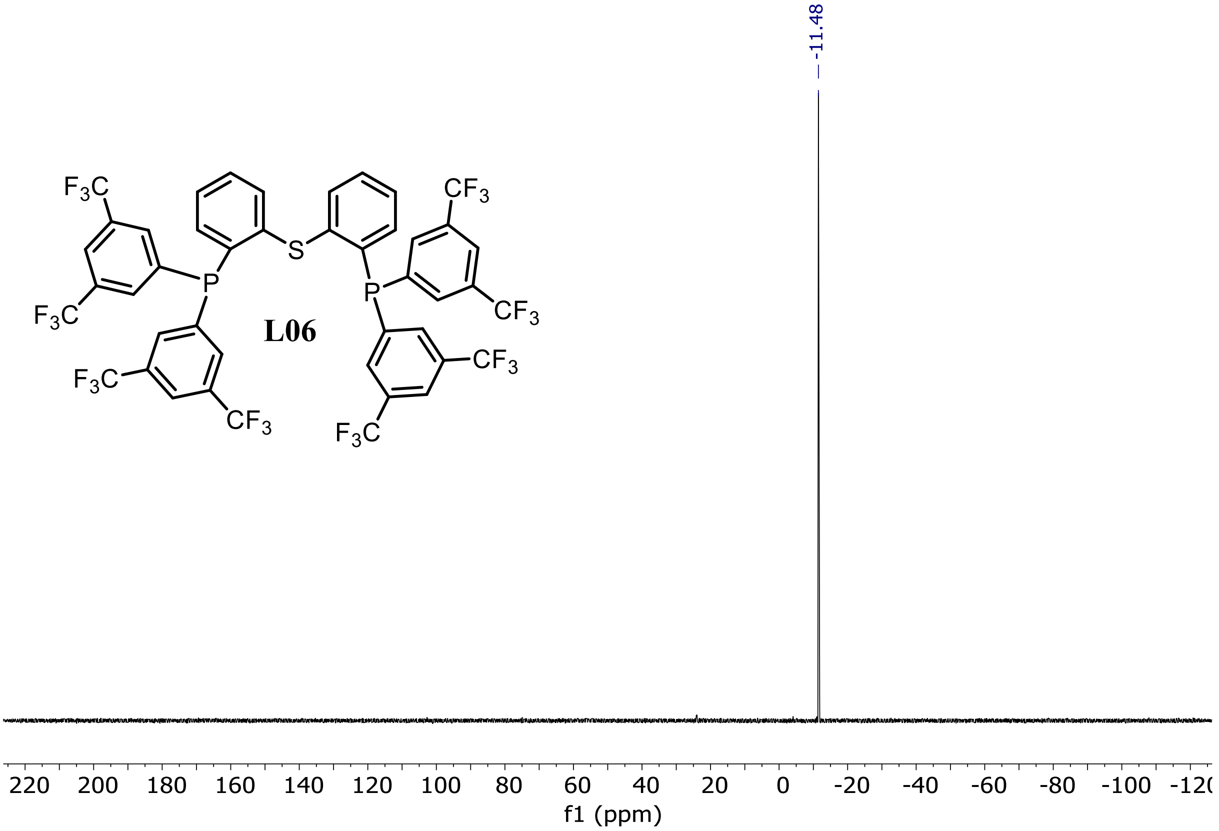
-11.48
F_3C
CF_3
L06
220 200 180 160 140 120 100 80 60 40 20 0 -20 -40 -60 -80 -100
f1 (ppm)

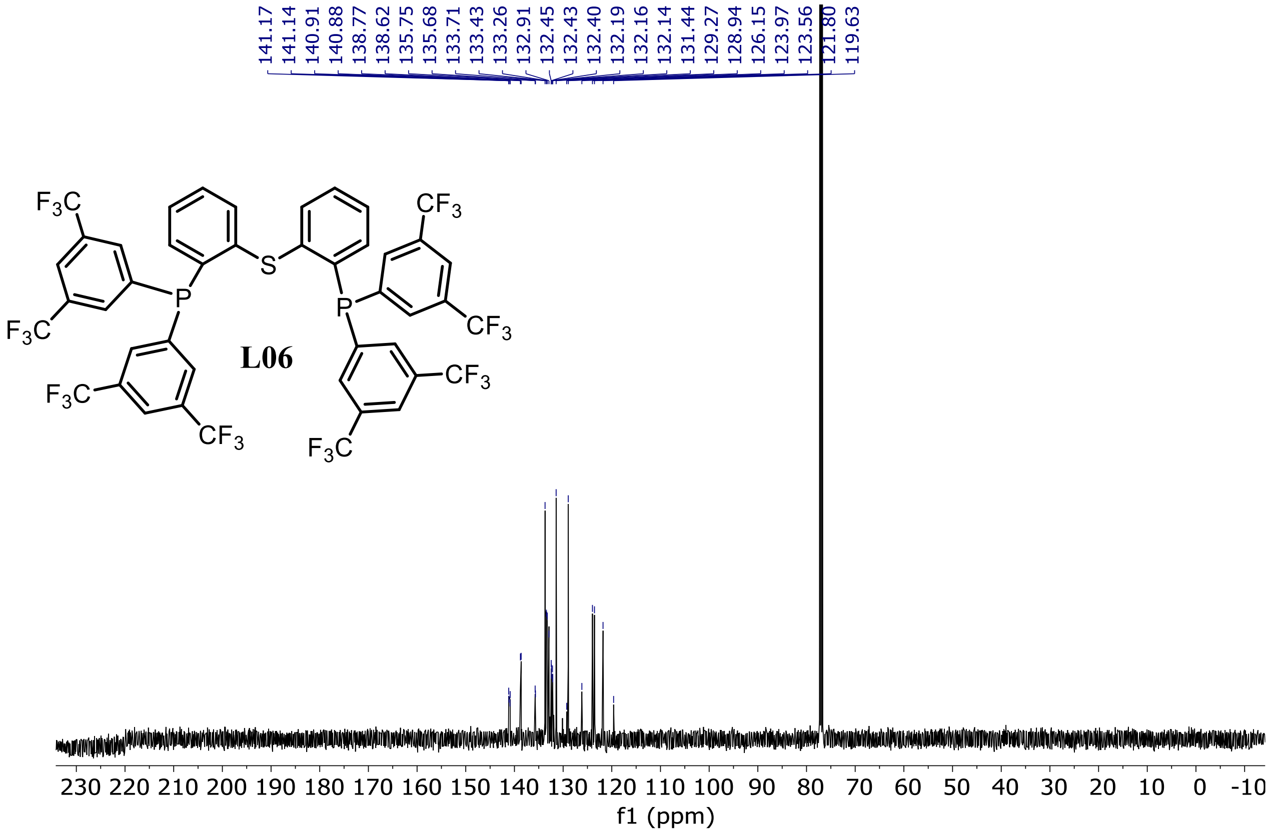
141.17
141.14
140.91
140.88
138.77
138.62
135.75
135.68
133.71
133.43
133.26
132.91
132.45
132.43
132.40
132.19
132.16
132.14
131.44
129.27
128.94
126.15
123.97
123.56
121.80
119.63
F_3C
CF_3
L06
230 220 210 200 190 180 170 160 150 140 130 120 110 100 90 80 70 60 50 40 30 20 10 0 -10
f1 (ppm)

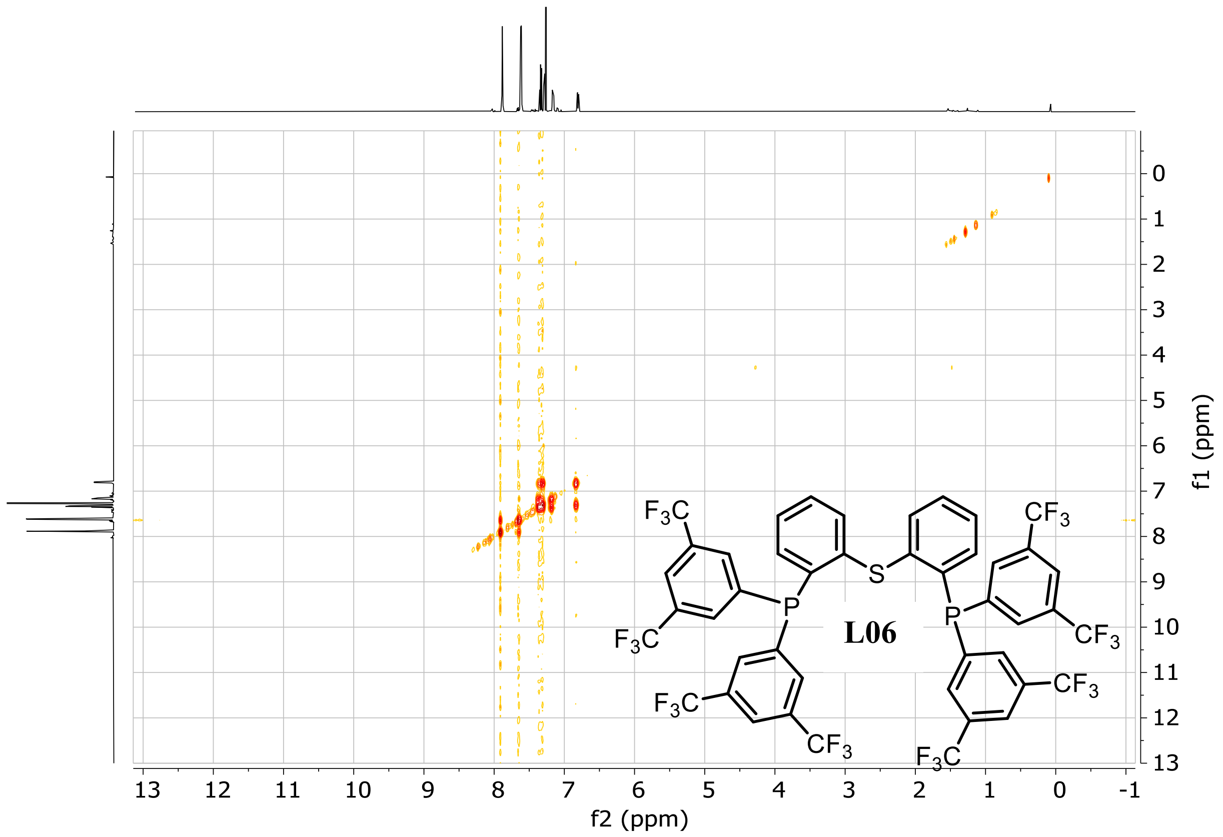
F3C
CF3
S
P
P
L06
F3C
CF3
F3C
CF3
F3C
CF3
f2 (ppm)
f1 (ppm)

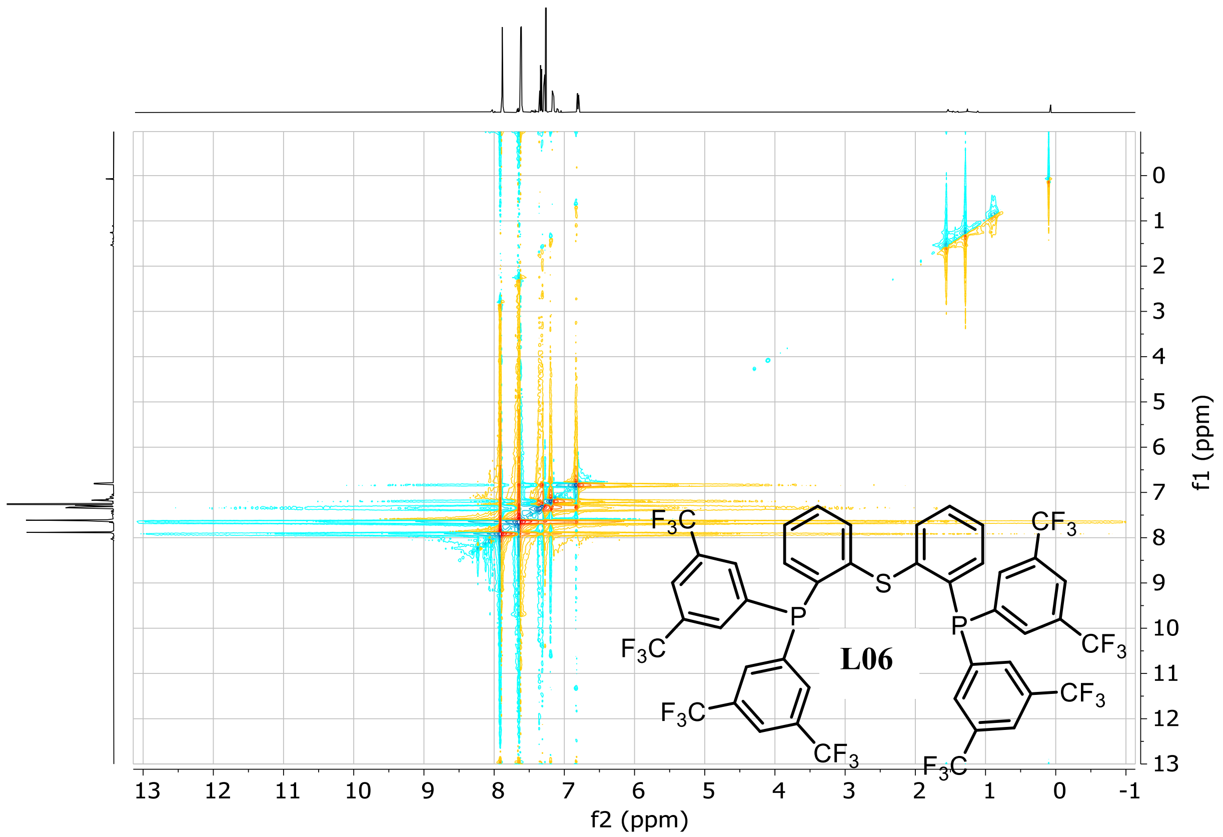
F3C
CF3
S
P
P
L06
F3C
CF3
F3C
CF3
F3C
CF3
f2 (ppm)
f1 (ppm)

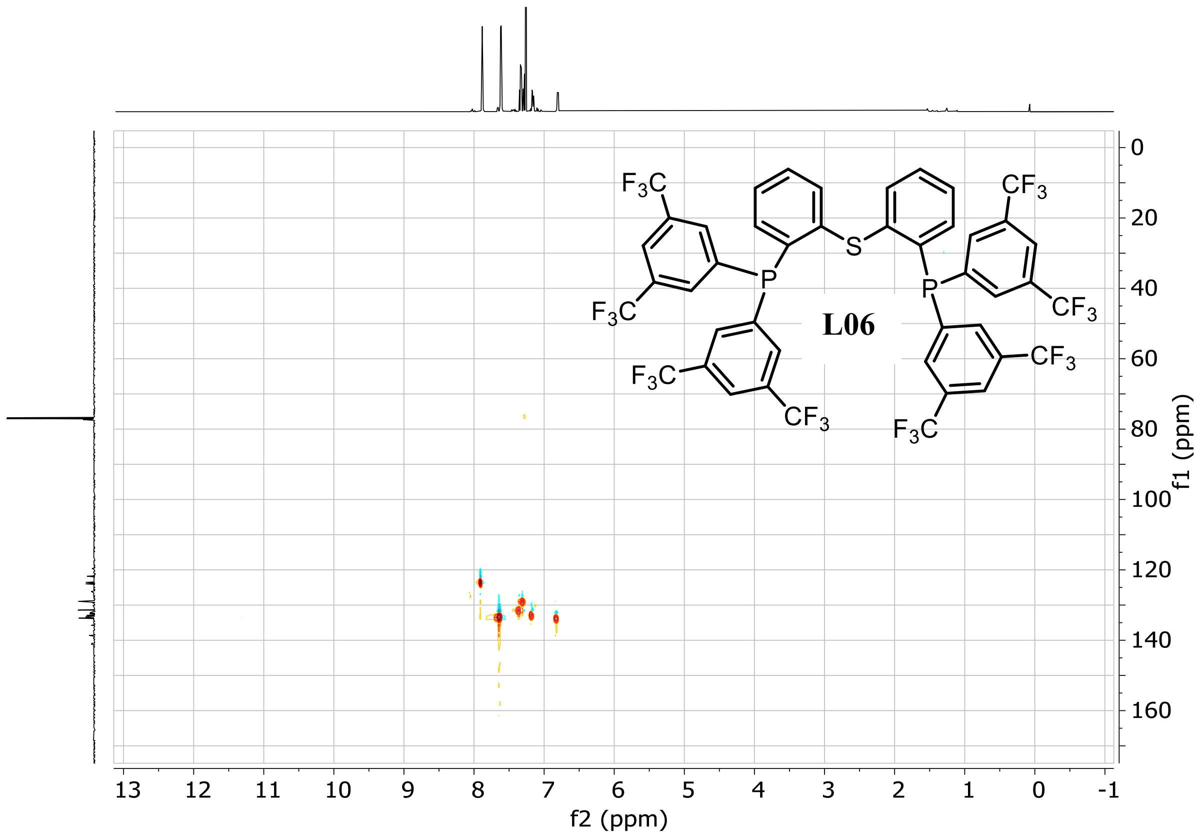
L06
F3C
CF3
S
P
f1 (ppm)
f2 (ppm)

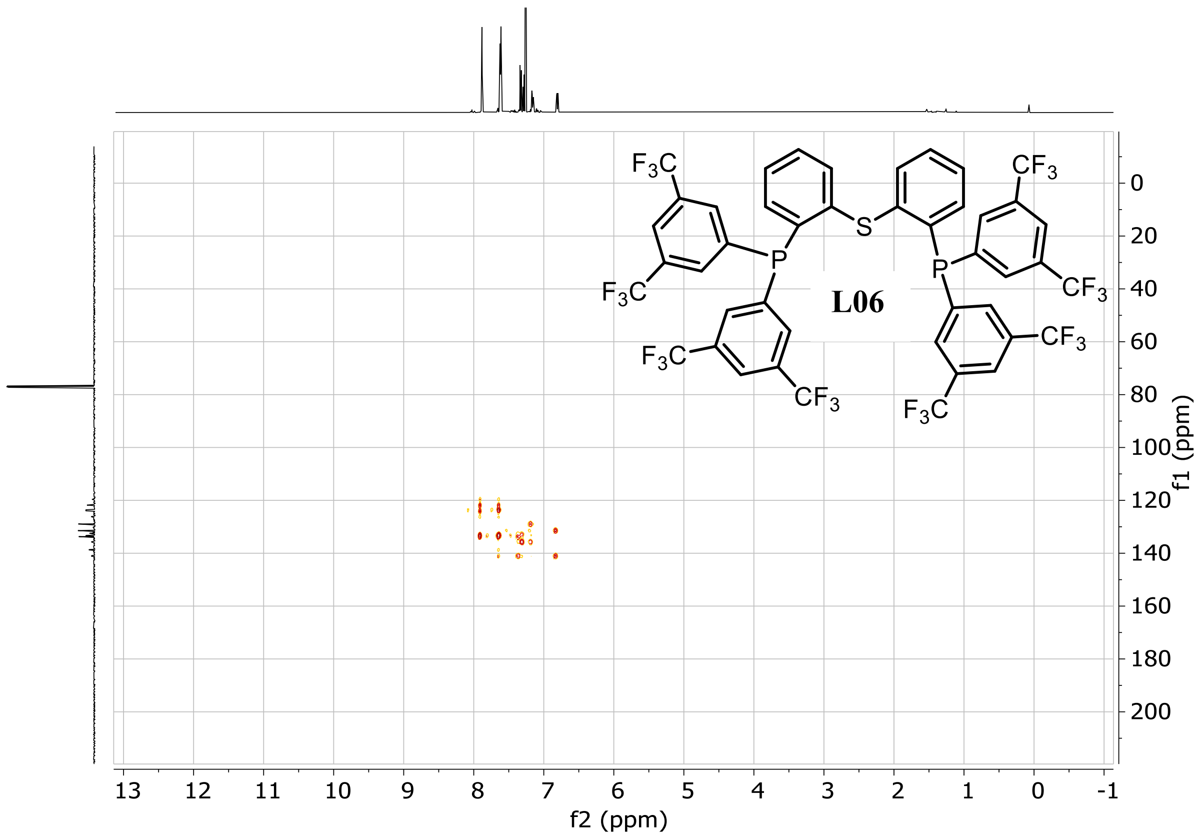
L06
F3C
CF3
S
P
f1 (ppm)
f2 (ppm)

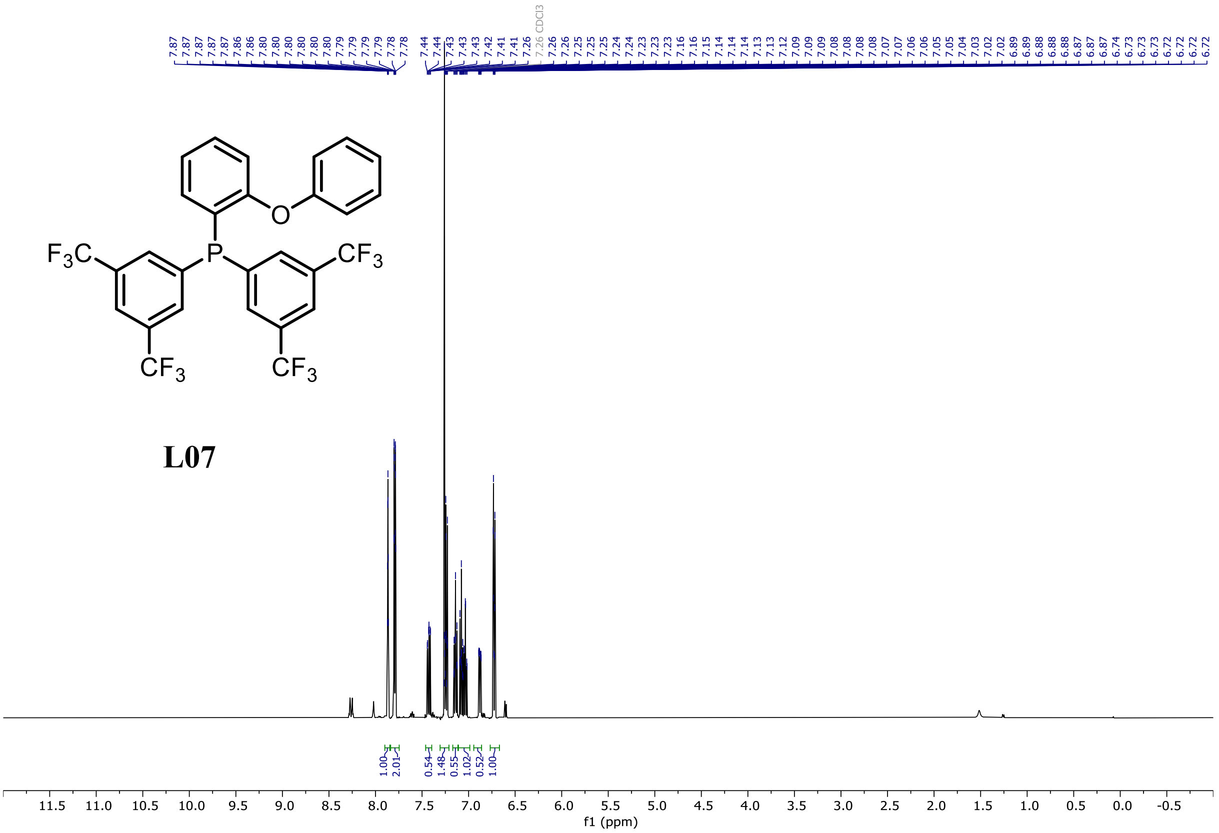
CDCl3
F_3C
CF_3
L07
f1 (ppm)

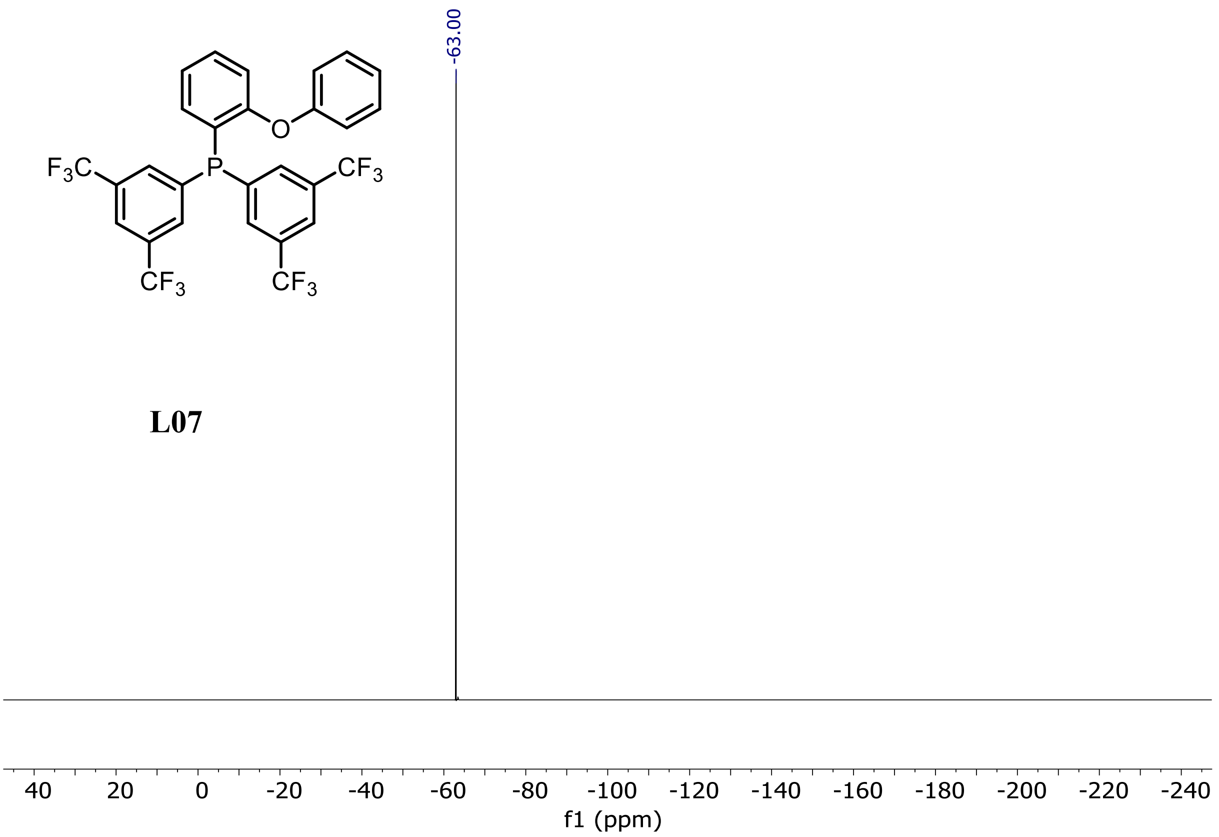
-63.00
F_3C
CF_3
L07
f1 (ppm)

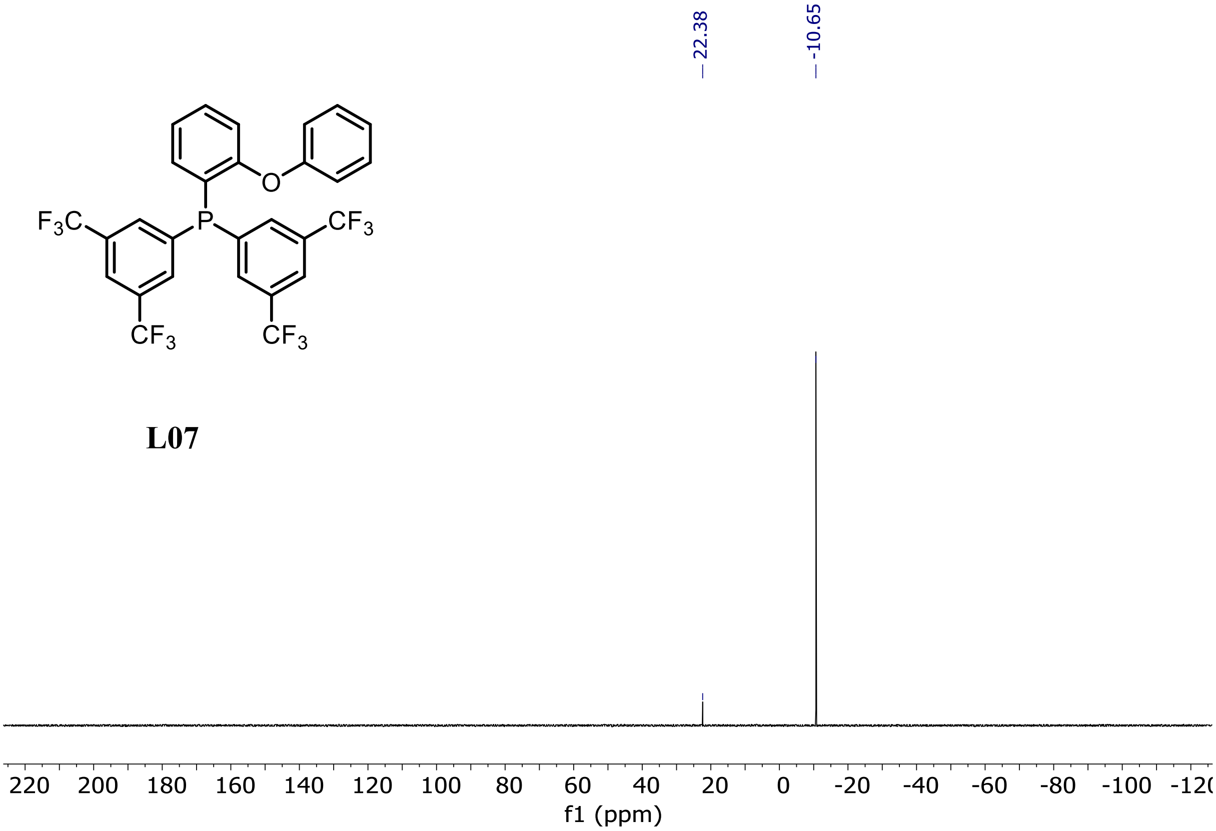

22.38
-10.65
F3C
CF3
CF3
CF3
P
O
220 200 180 160 140 120 100 80 60 40 20 0 -20 -40 -60 -80 -100 -120
f1 (ppm)

L07

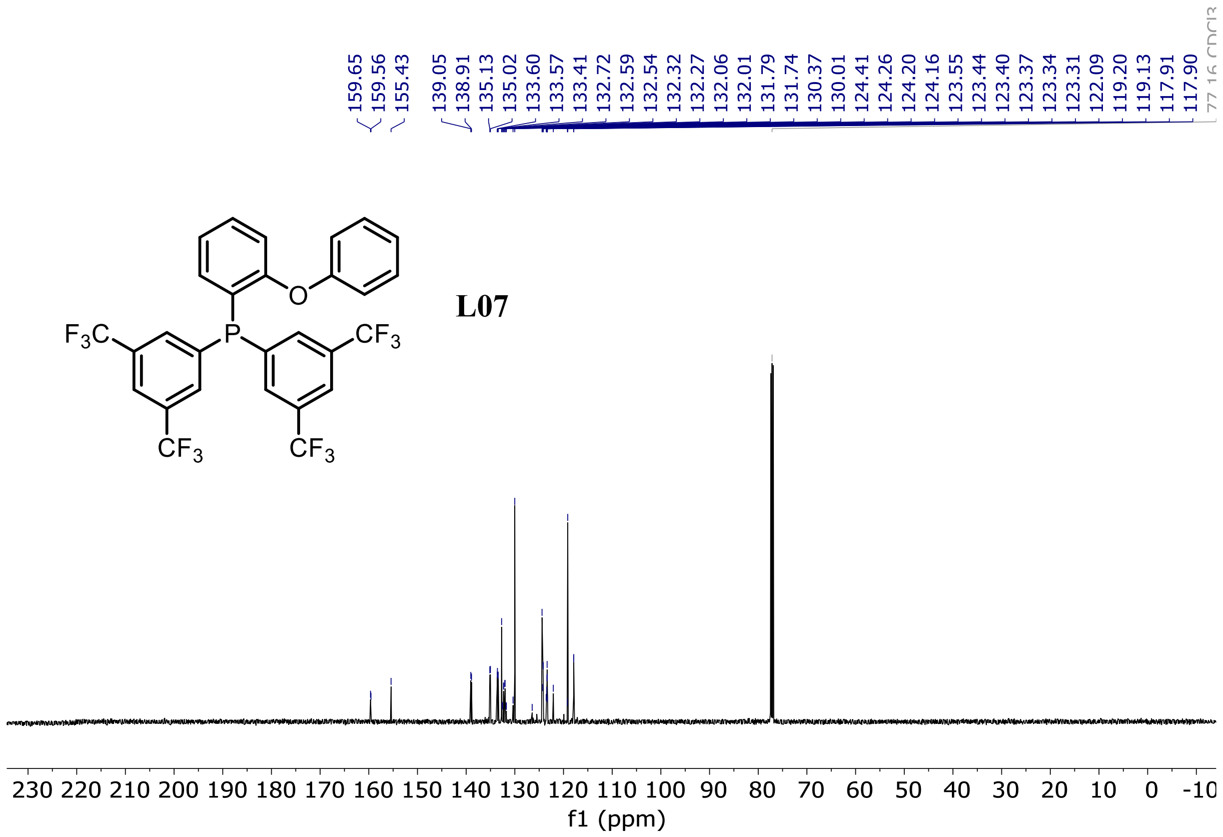
159.65
159.56
155.43
139.05
138.91
135.13
135.02
133.60
133.57
133.41
132.72
132.59
132.54
132.32
132.27
132.06
132.01
131.79
131.74
130.37
130.01
124.41
124.26
124.20
124.16
123.55
123.44
123.40
123.37
123.34
123.31
122.09
119.20
119.13
117.91
117.90
F$_3$C
CF$_3$
CF$_3$
CF$_3$
P
O
L07
230 220 210 200 190 180 170 160 150 140 130 120 110 100 90 80 70 60 50 40 30 20 10 0 -10
f1 (ppm)

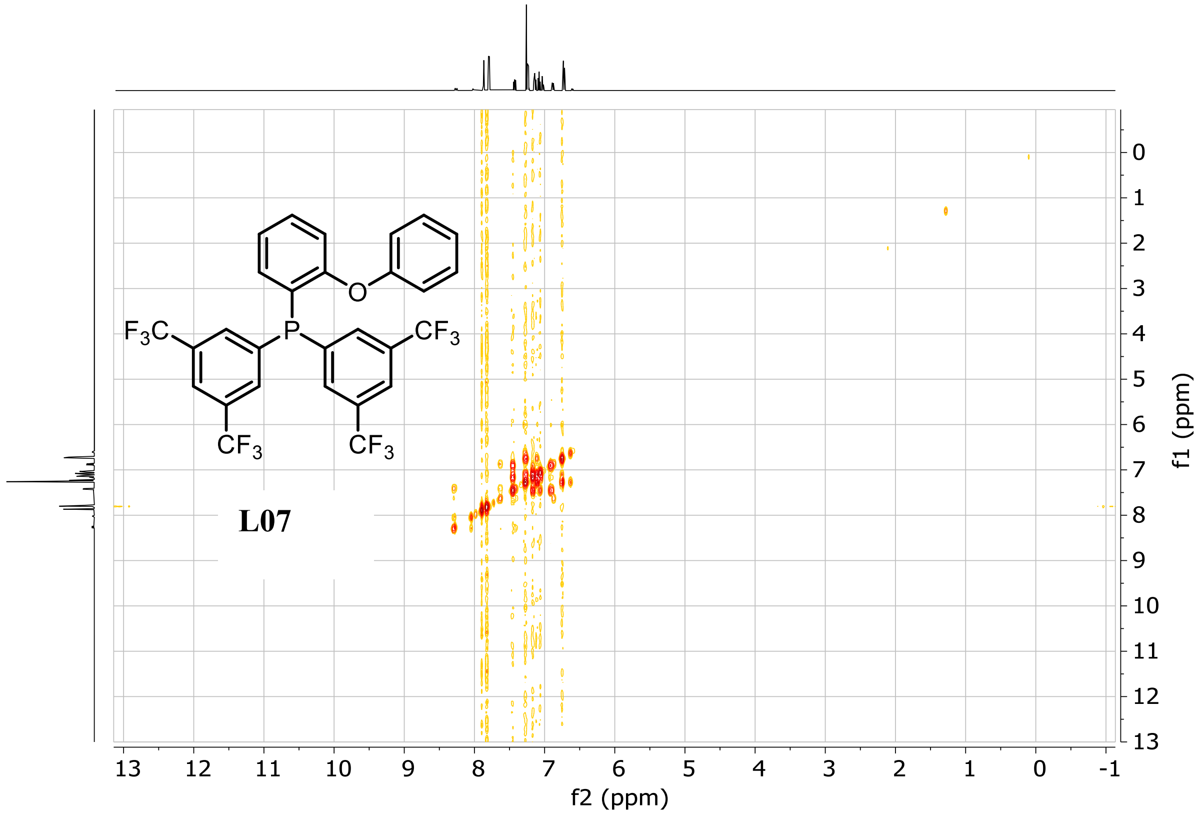
F$_3$C
CF$_3$
CF$_3$
CF$_3$
P
O
L07
13 12 11 10 9 8 7 6 5 4 3 2 1 0 -1
f2 (ppm)
0 1 2 3 4 5 6 7 8 9 10 11 12 13
f1 (ppm)

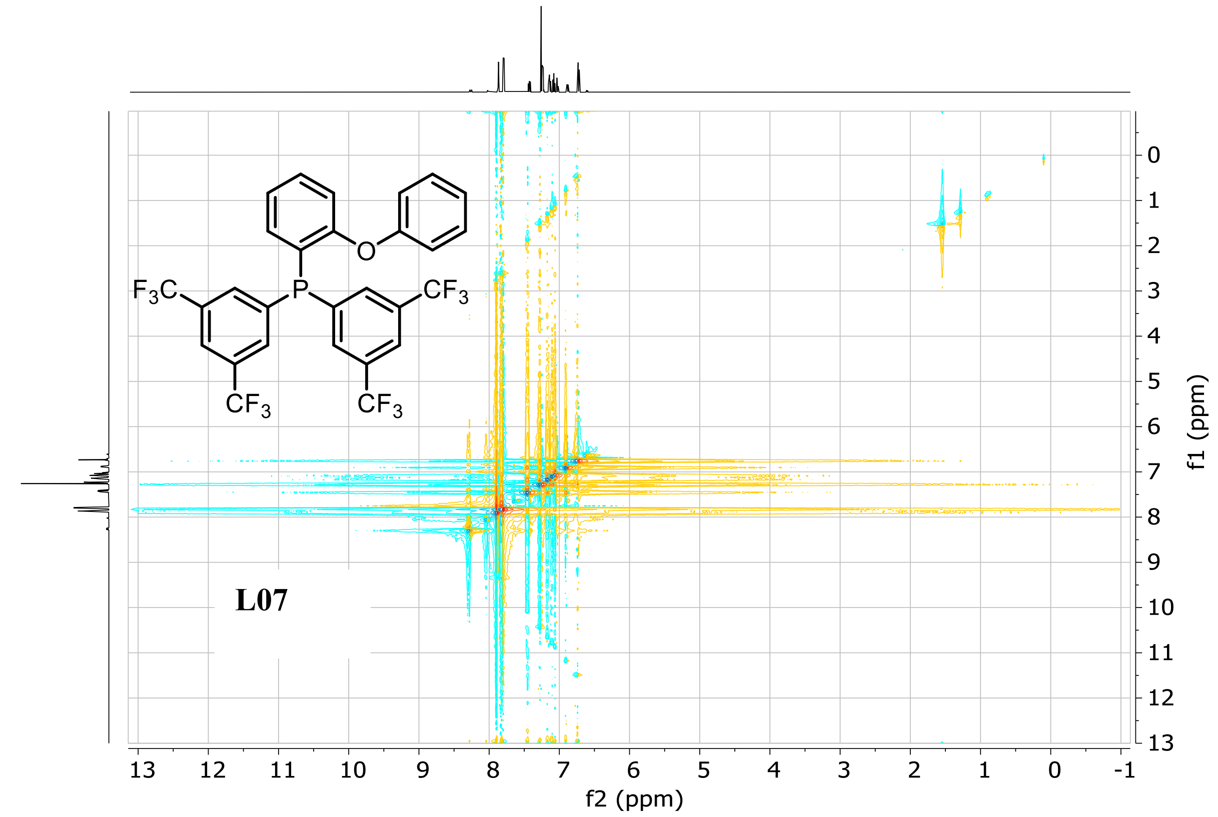
F3C
CF3
CF3
CF3
P
O
L07
f2 (ppm)
f1 (ppm)

..

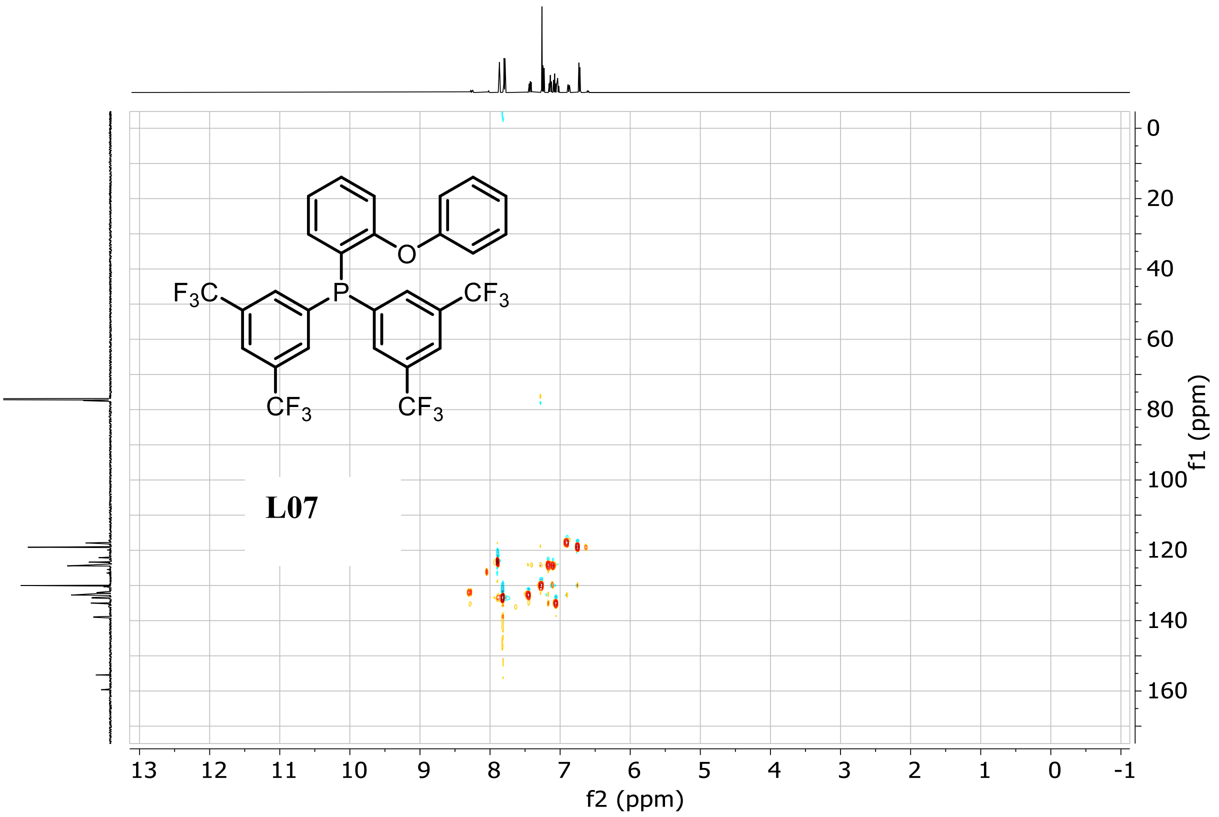
F3C
CF3
CF3
CF3
P
O
L07
f2 (ppm)
f1 (ppm)

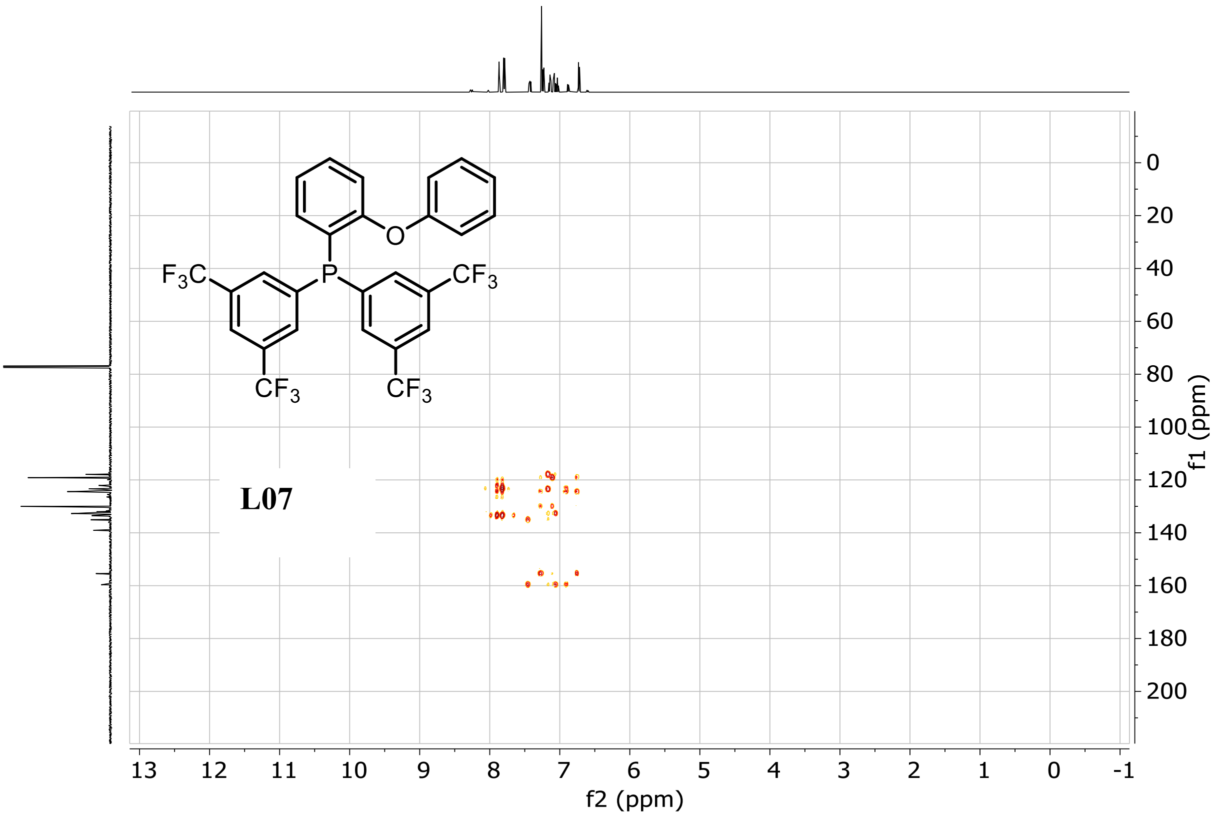
L07
f2 (ppm)
f1 (ppm)

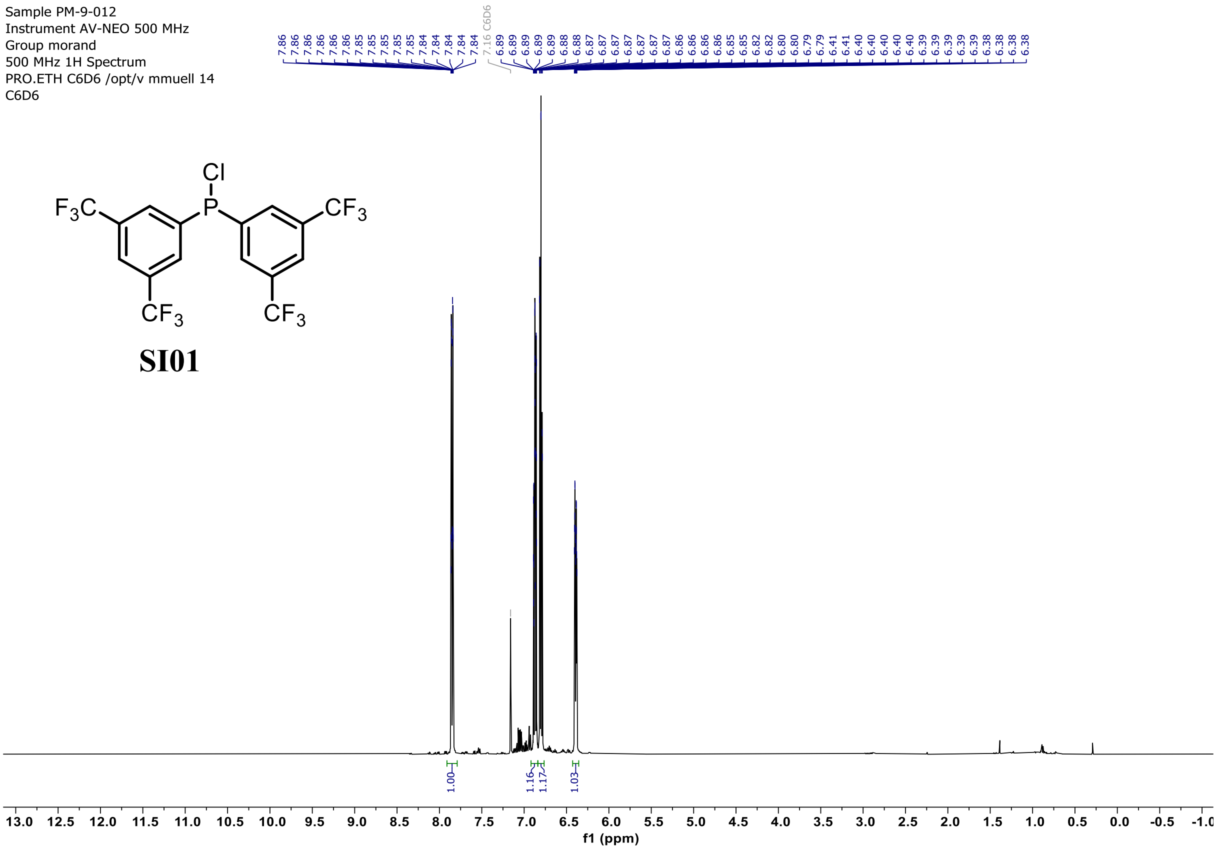
Sample PM-9-012
Instrument AV-NEO 500 MHz
Group morand
500 MHz 1H Spectrum
PRO.ETH C6D6 /opt/v mmuell 14
C6D6
SI01
7.16 C6D6
f1 (ppm)

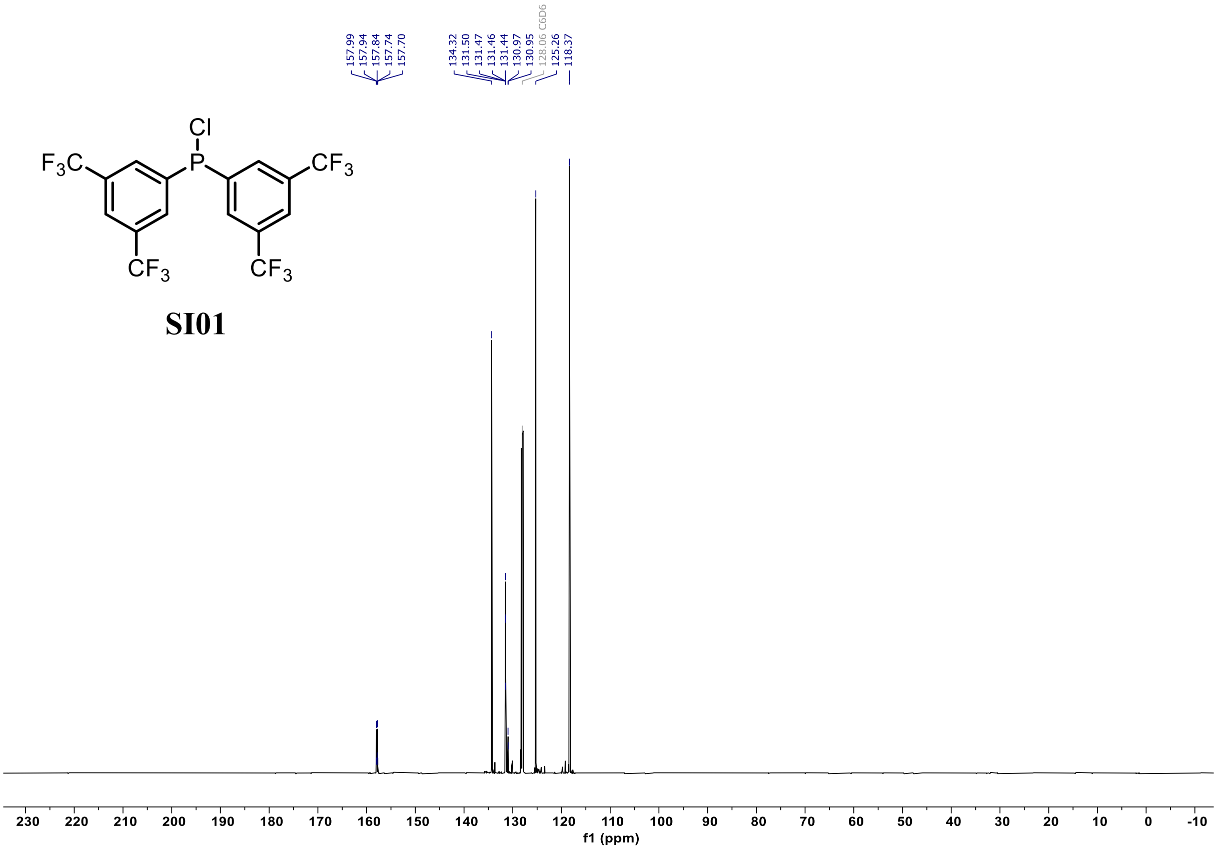
157.99
157.94
157.84
157.74
157.70
134.32
131.50
131.47
131.46
131.44
130.97
130.95
128.06 C6D6
125.26
118.37
Cl
P
F3C
CF3
CF3
CF3
SI01
230 220 210 200 190 180 170 160 150 140 130 120 110 100 90 80 70 60 50 40 30 20 10 0 -10
f1 (ppm)

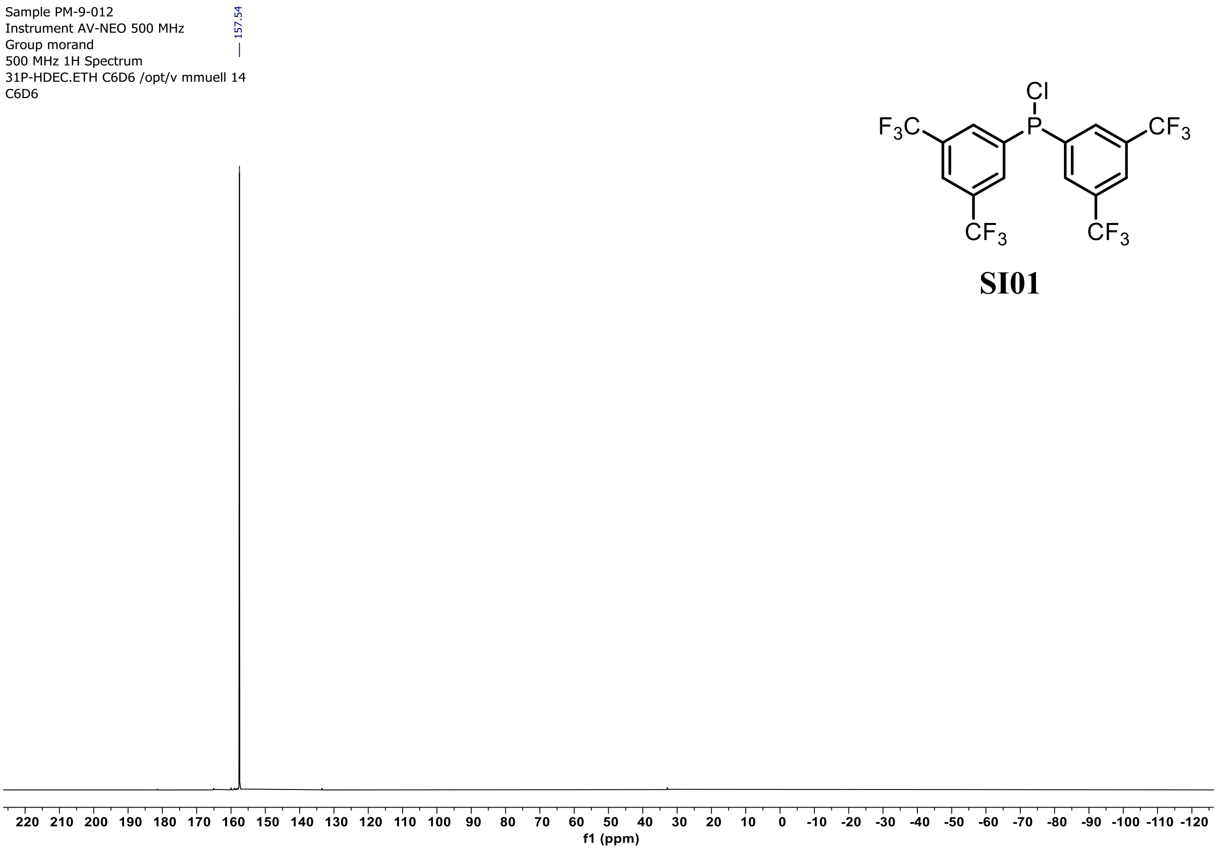
Sample PM-9-012
Instrument AV-NEO 500 MHz
Group morand
500 MHz 1H Spectrum
31P-HDEC.ETH C6D6 /opt/v mmuell 14
C6D6
157.54
Cl
P
F3C
CF3
CF3
CF3
SI01
220 210 200 190 180 170 160 150 140 130 120 110 100 90 80 70 60 50 40 30 20 10 0 -10 -20 -30 -40 -50 -60 -70 -80 -90 -100 -110 -120
f1 (ppm)

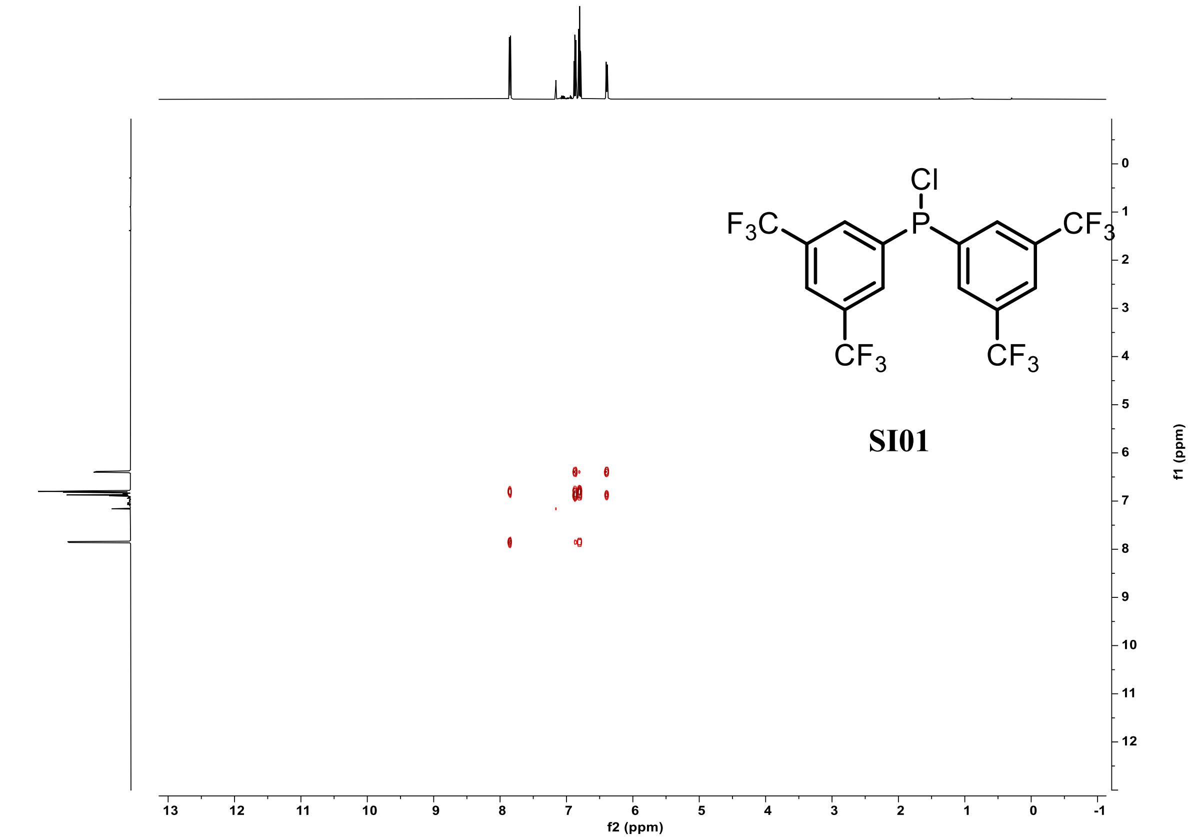

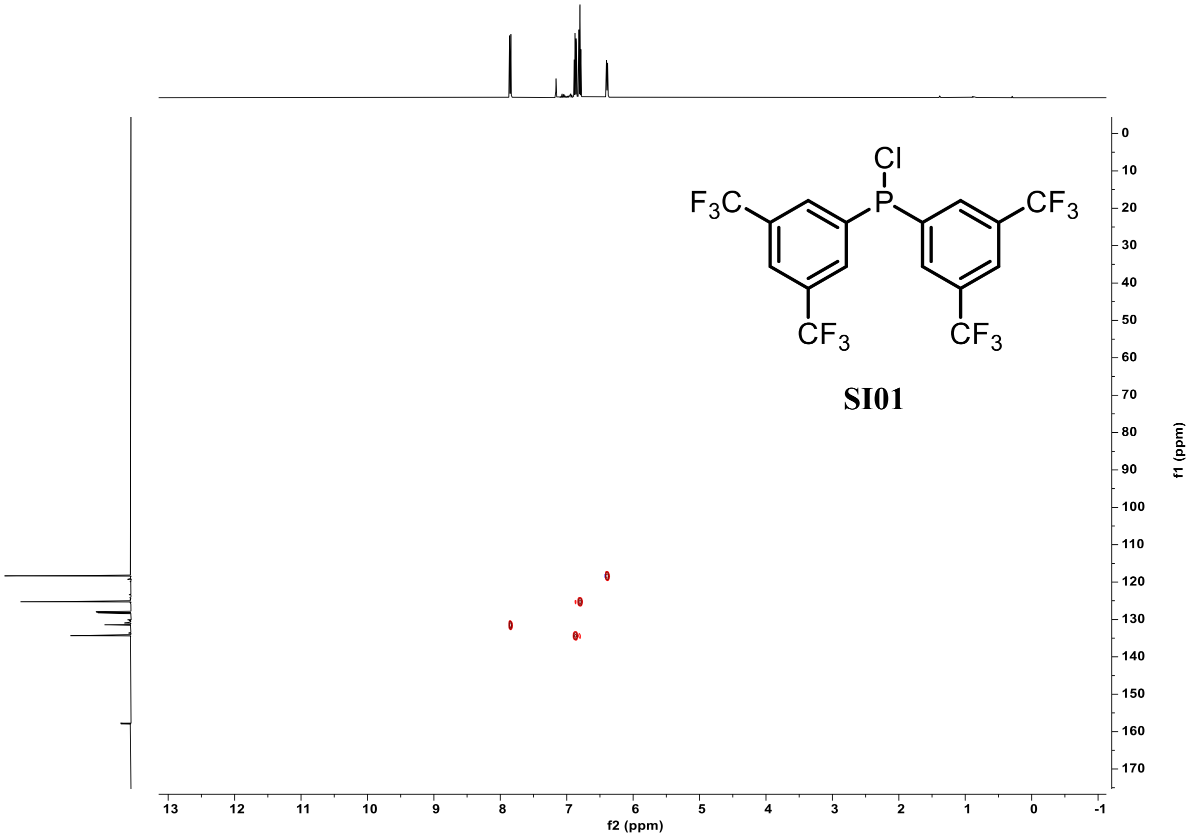

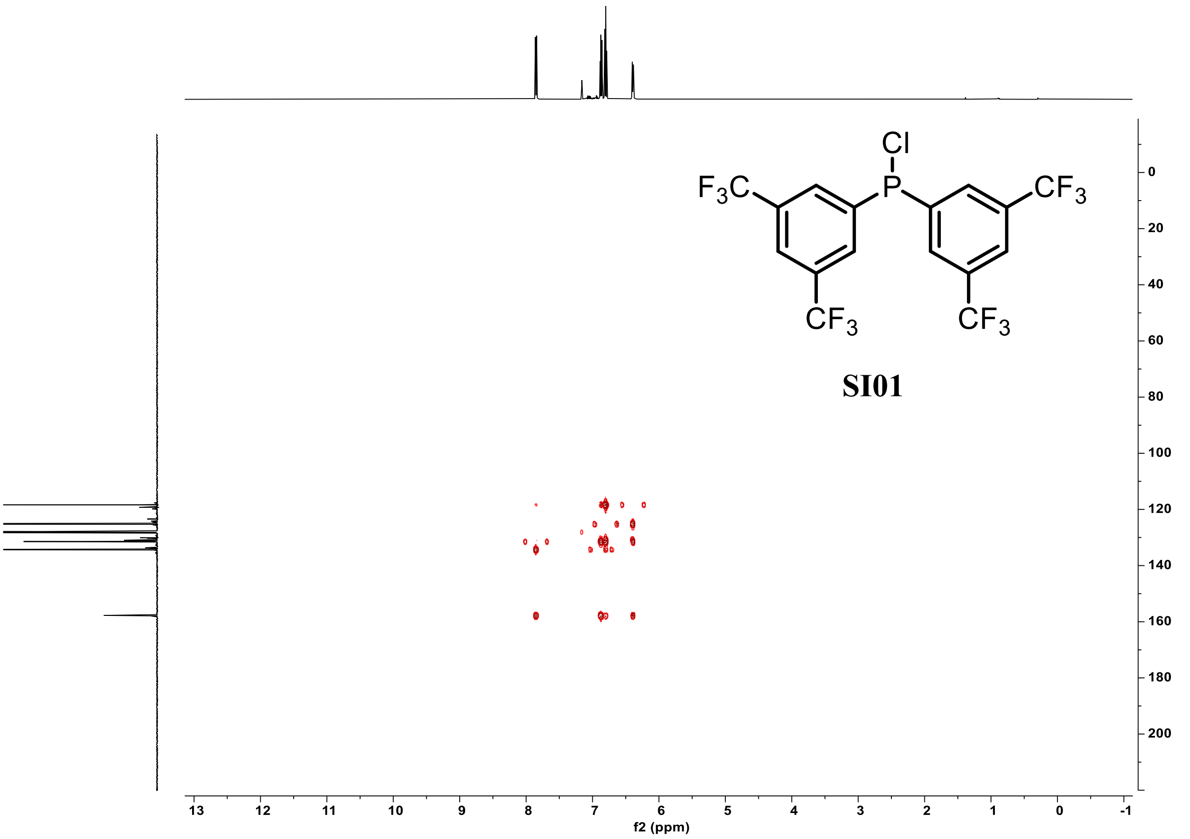

SI01

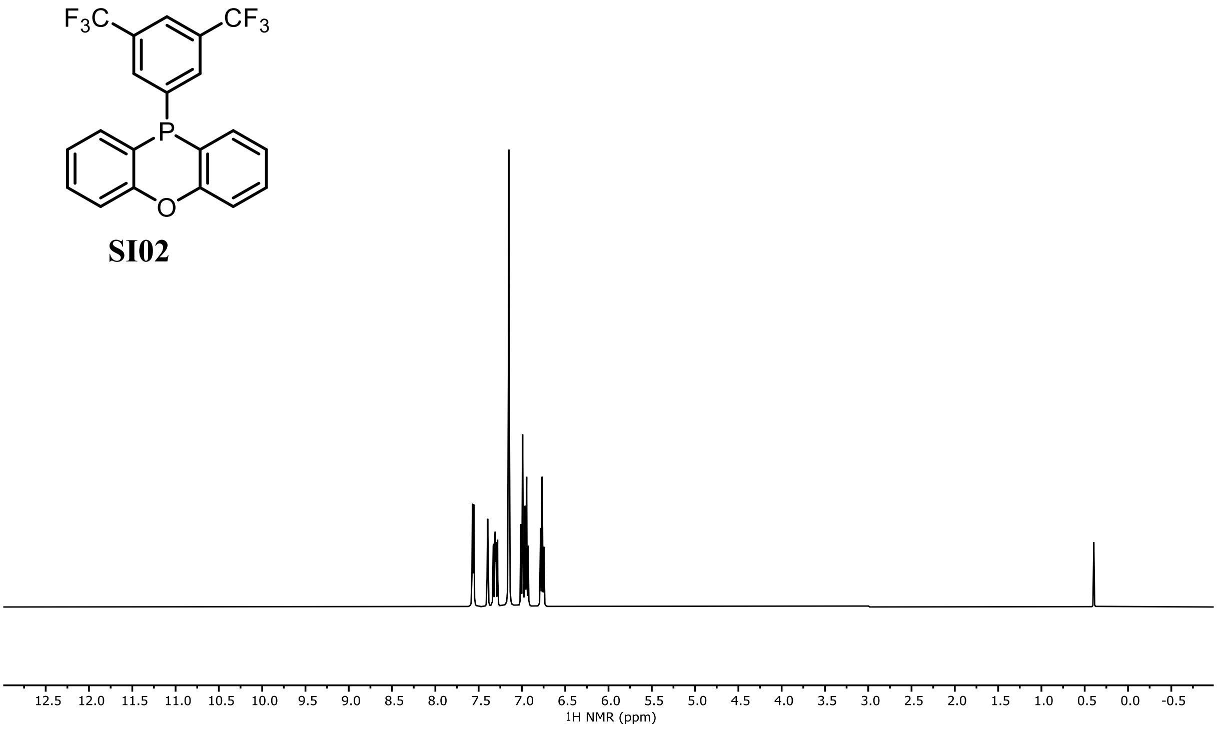

SI02

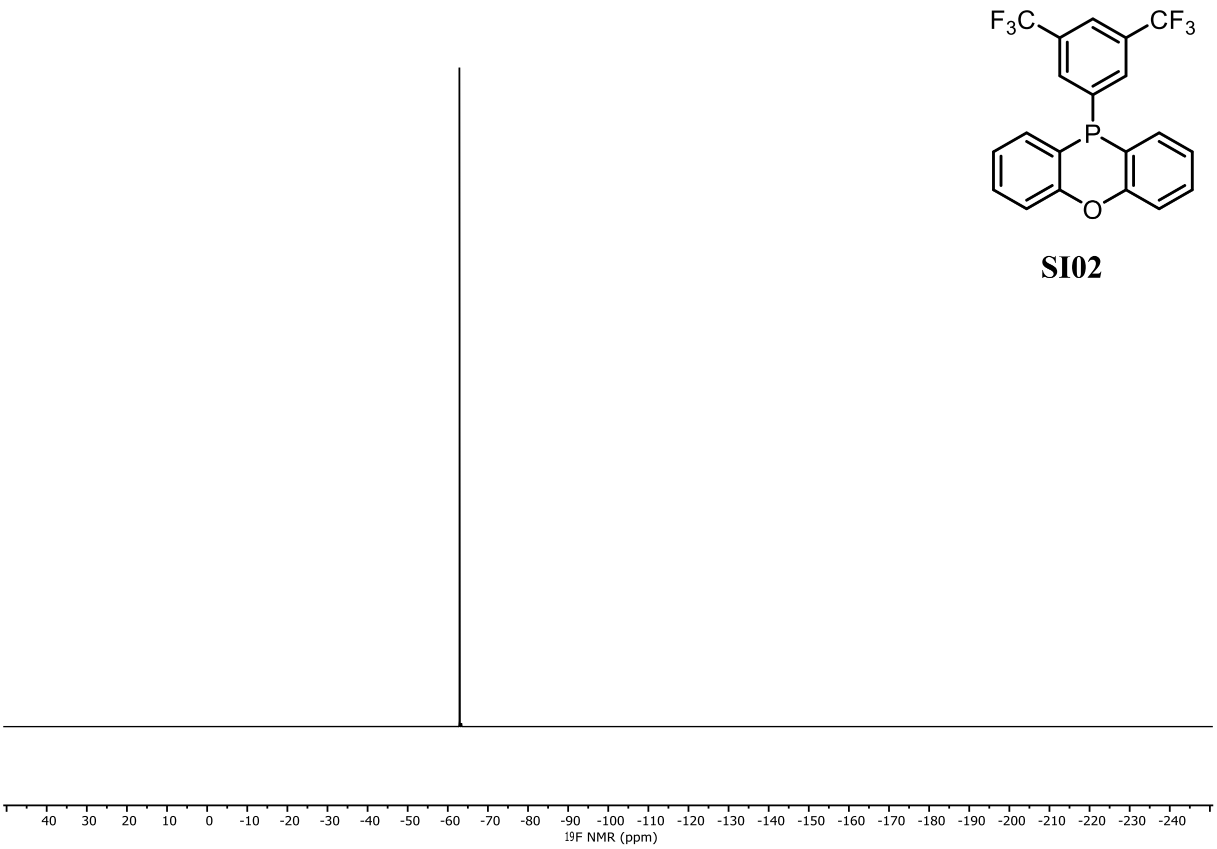
F3C
CF3
P
O
SI02
40 30 20 10 0 -10 -20 -30 -40 -50 -60 -70 -80 -90 -100 -110 -120 -130 -140 -150 -160 -170 -180 -190 -200 -210 -220 -230 -240
19F NMR (ppm)

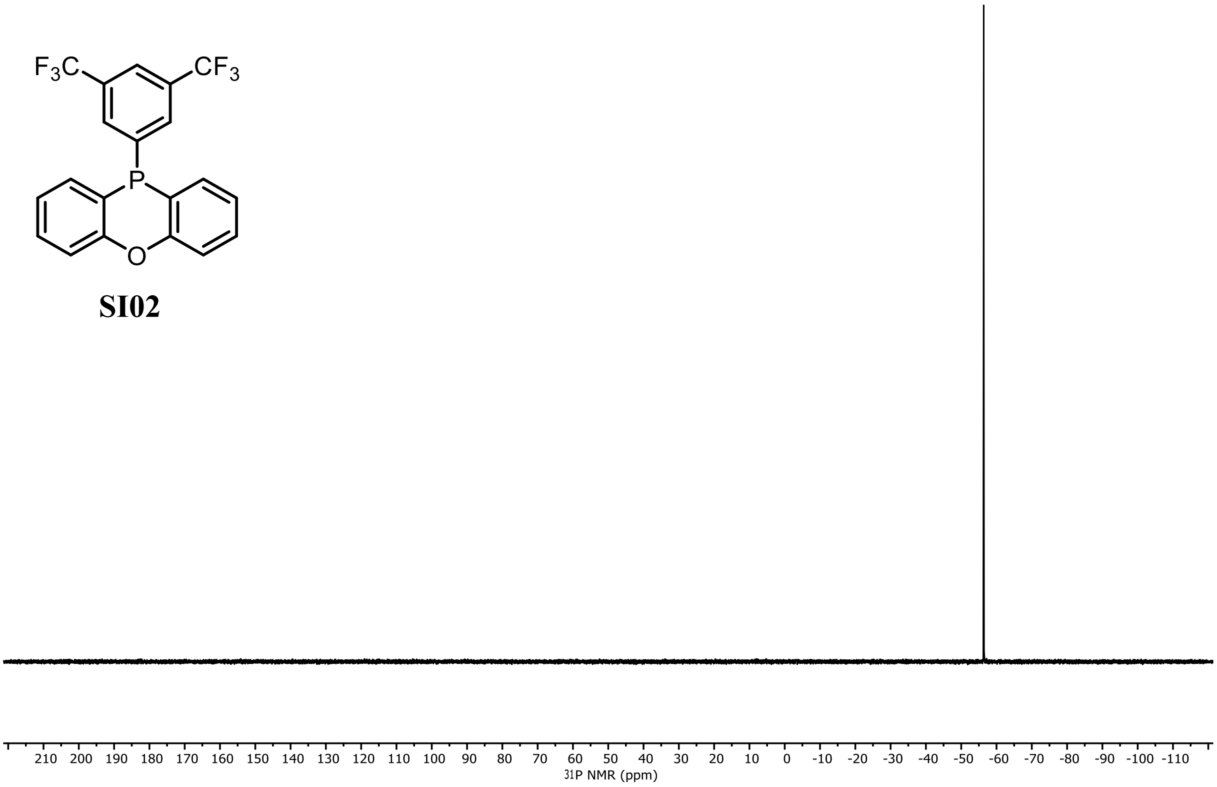
F3C
CF3
P
O
SI02
210 200 190 180 170 160 150 140 130 120 110 100 90 80 70 60 50 40 30 20 10 0 -10 -20 -30 -40 -50 -60 -70 -80 -90 -100 -110
31P NMR (ppm)

Carbochlorocarbonylation

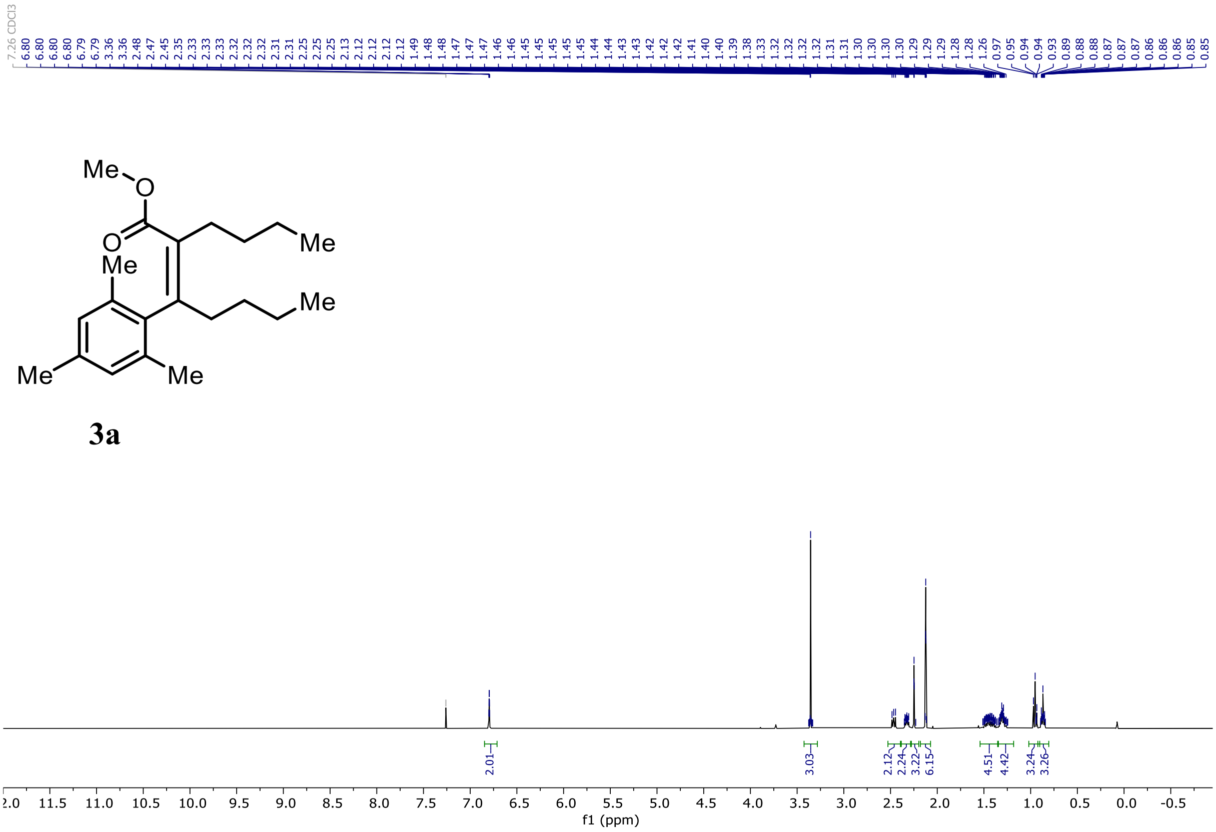

3a

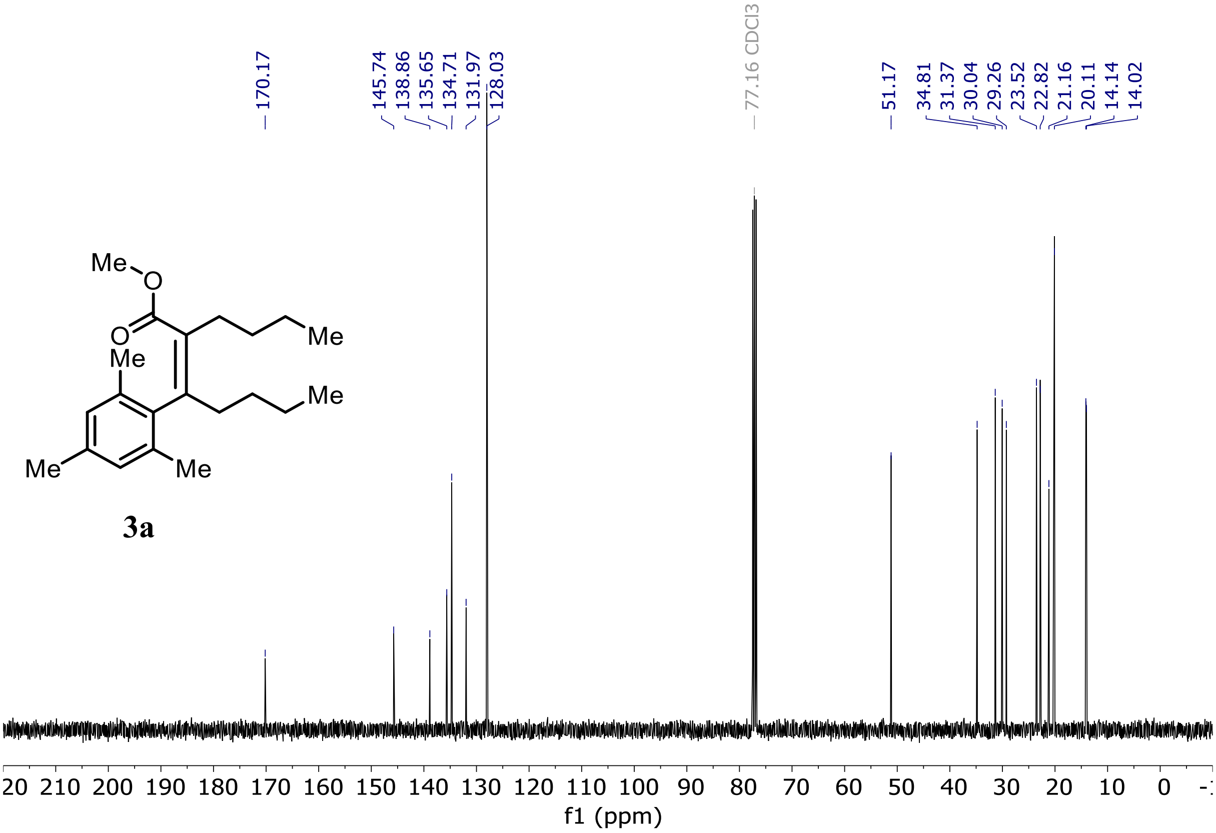

3a

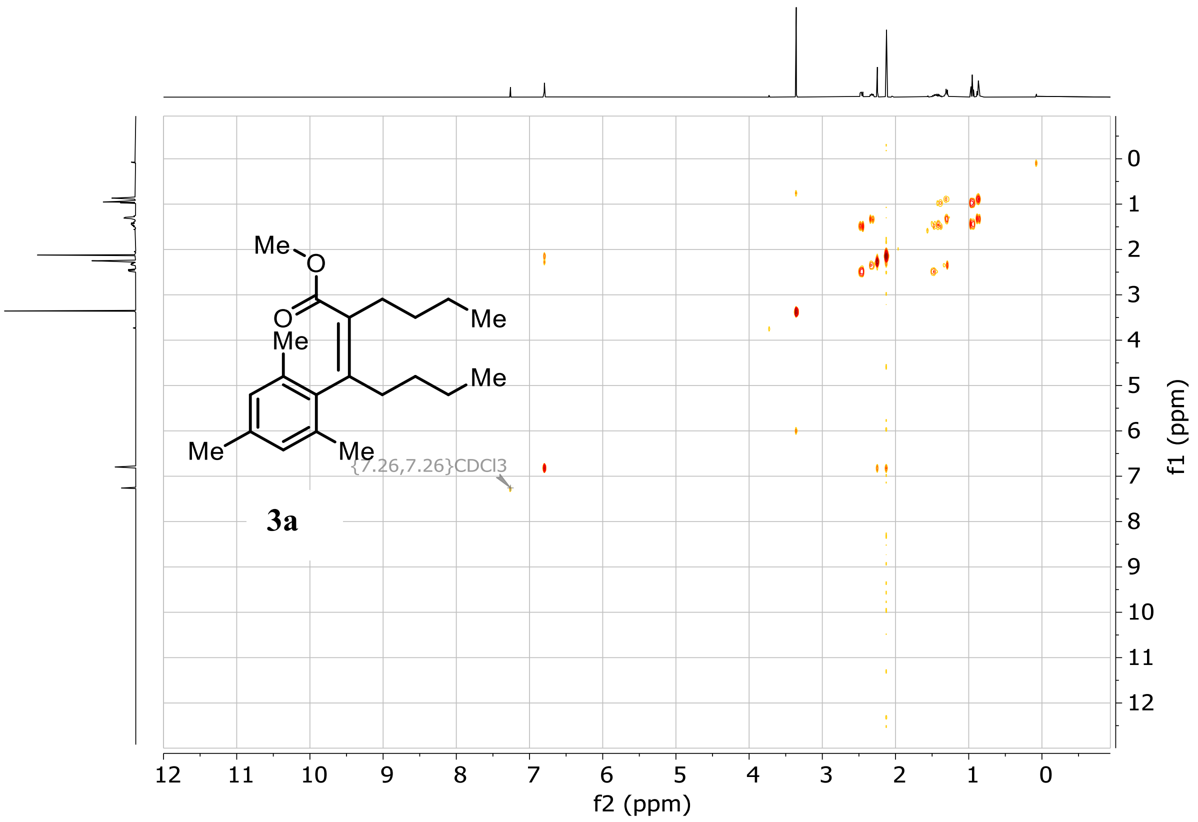

Me
O
O
Me
Me
Me
Me
Me
{7.26,7.26}CDCl3
3a
f2 (ppm)
f1 (ppm)

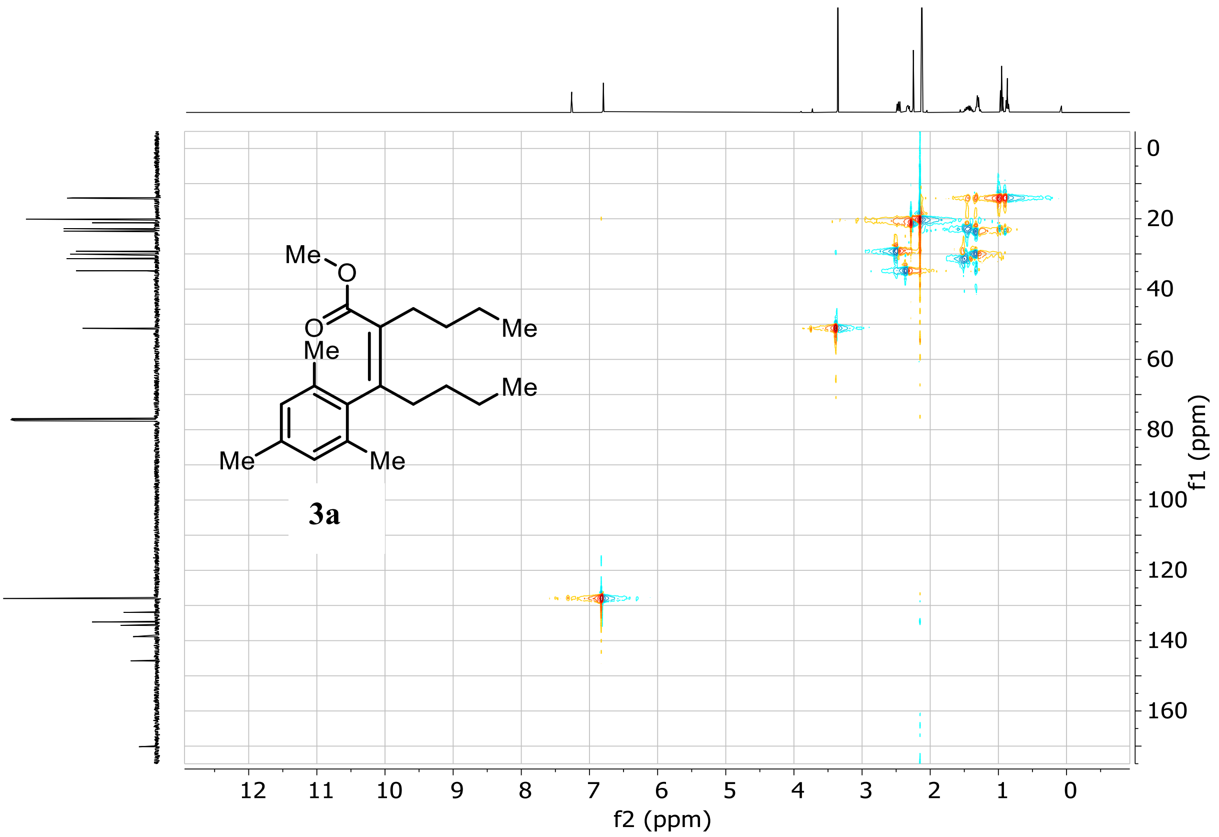

Me
O
O
Me
Me
Me
Me
Me
3a
f2 (ppm)
f1 (ppm)

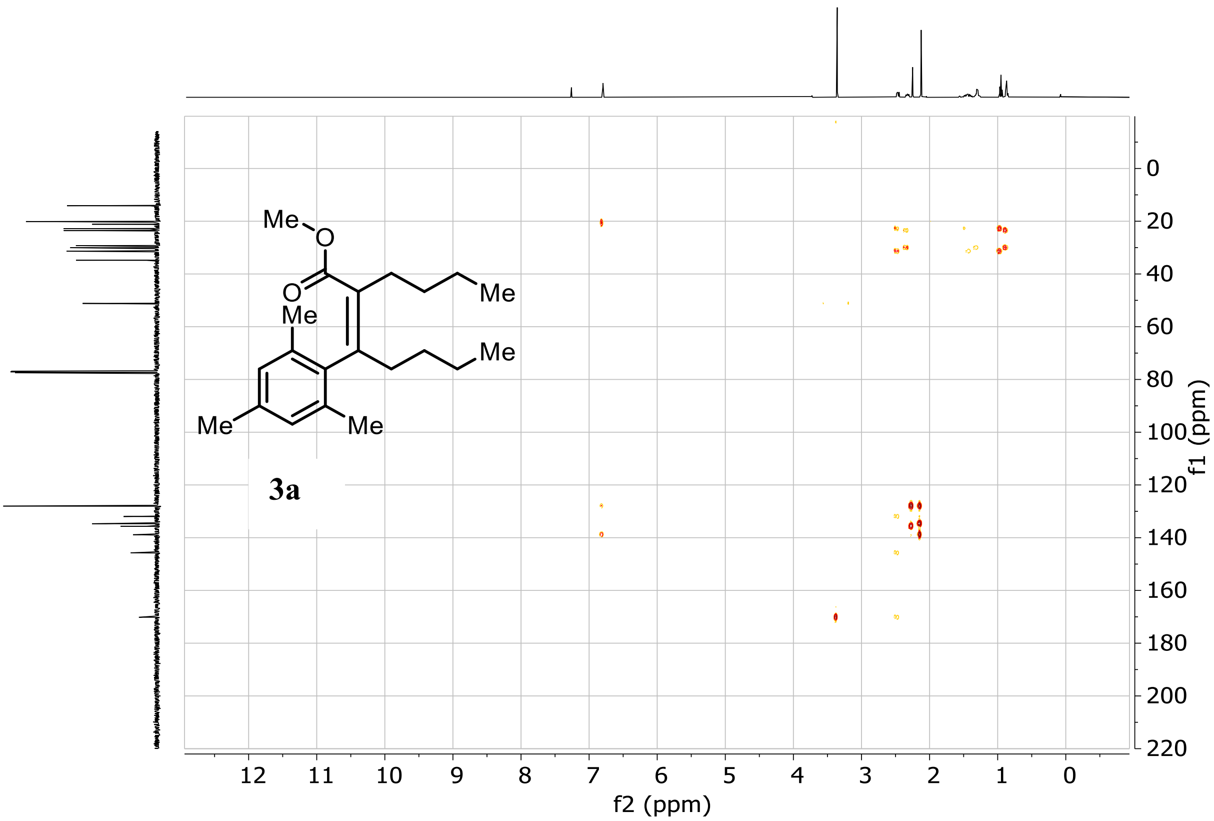

Me
O
O
Me
Me
Me
Me
Me
3a
f2 (ppm)
f1 (ppm)

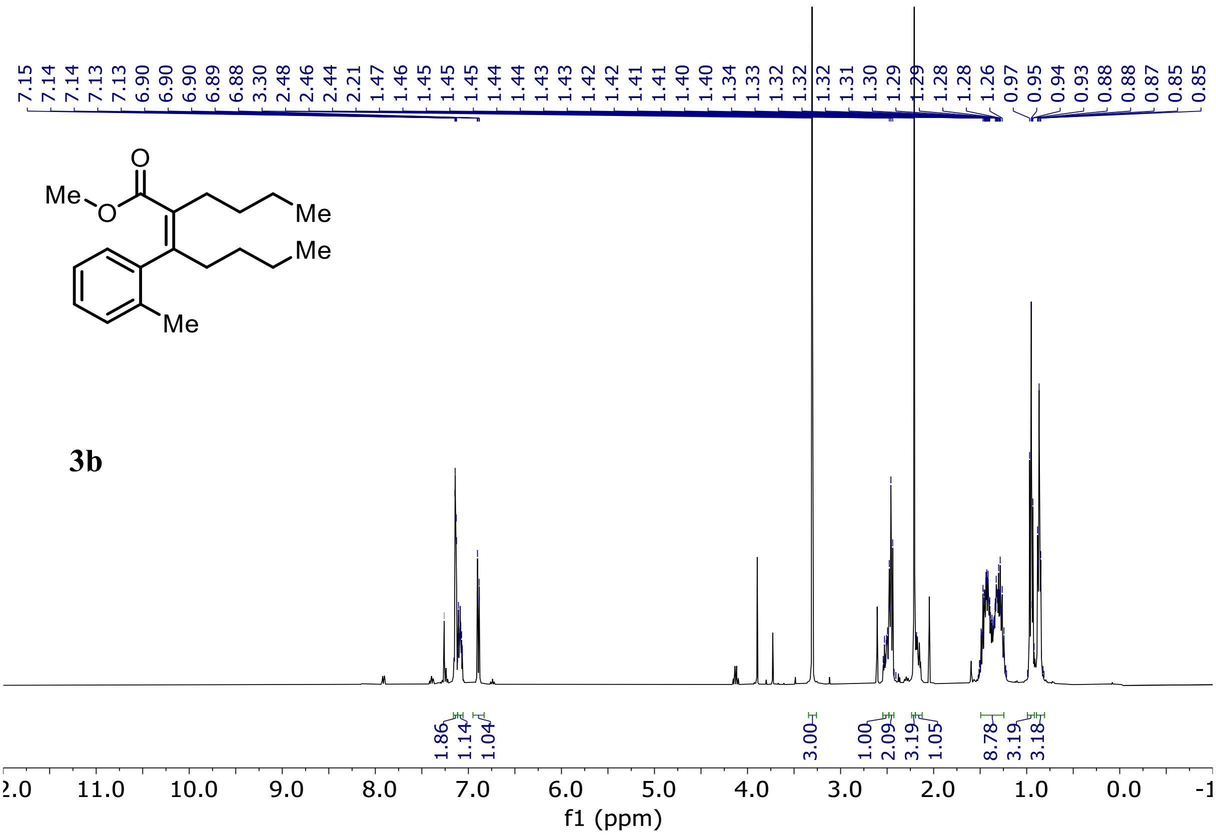

7.15 7.14 7.14 7.13 7.13 6.90 6.90 6.89 6.88 3.30 2.48 2.46 2.44 2.21 1.47 1.46 1.45 1.45 1.45 1.44 1.44 1.43 1.43 1.42 1.42 1.41 1.41 1.40 1.40 1.34 1.33 1.32 1.32 1.32 1.31 1.30 1.29 1.29 1.28 1.28 1.26 0.97 0.95 0.94 0.93 0.88 0.88 0.87 0.85 0.85
Me
O
Me
Me
Me
3b
1.86 1.14 1.04 3.00 1.00 2.09 3.19 1.05 8.78 3.19 3.18
f1 (ppm)

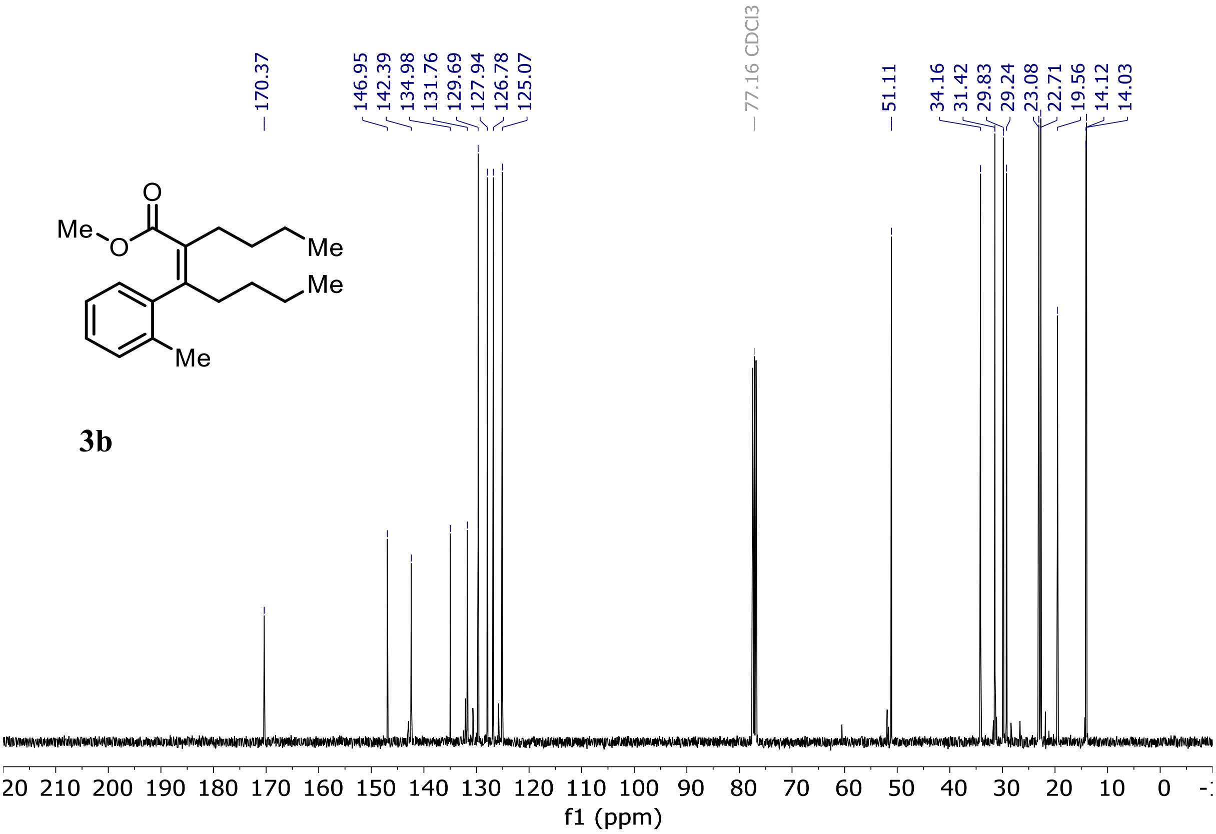

170.37
146.95 142.39 134.98 131.76 129.69 127.94 126.78 125.07
77.16 CDCl3
51.11
34.16 31.42 29.83 29.24 23.08 22.71 19.56 14.12 14.03
Me
O
Me
Me
Me
3b
f1 (ppm)

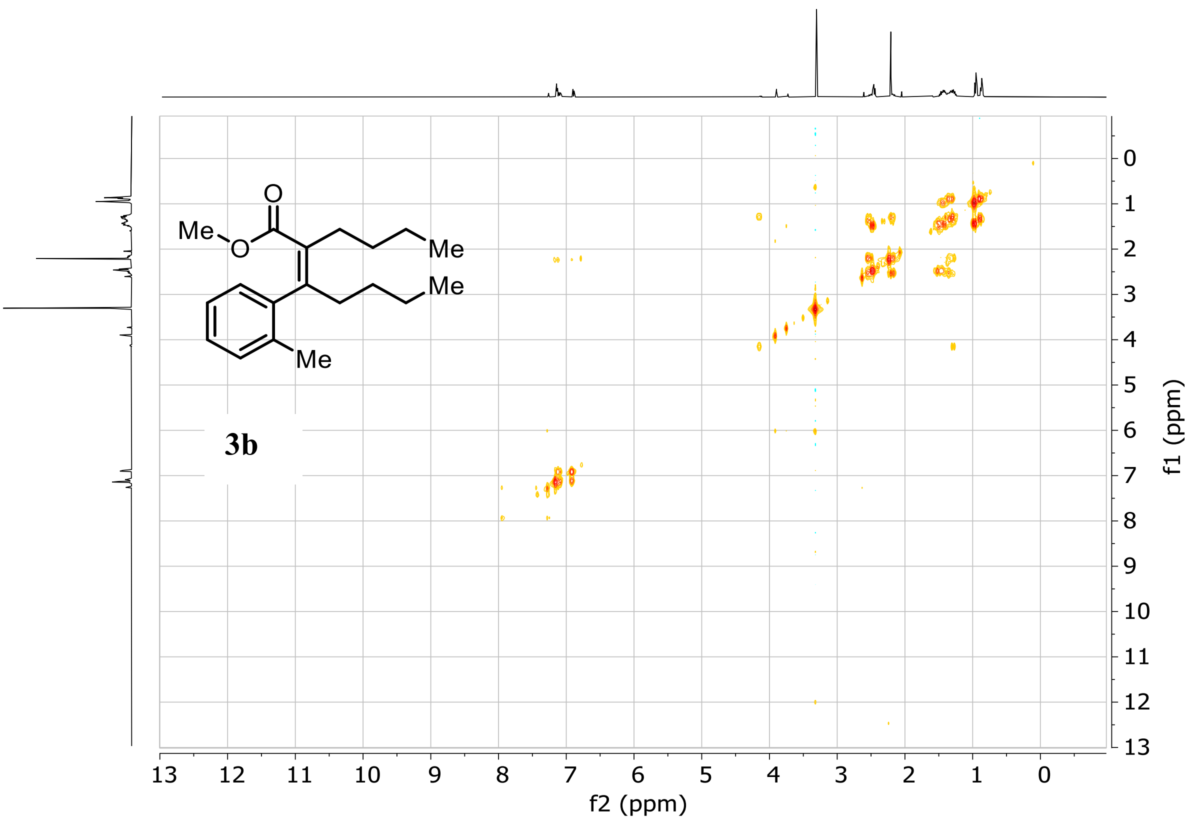
Me
O
O
Me
Me
Me
3b
f2 (ppm)
f1 (ppm)

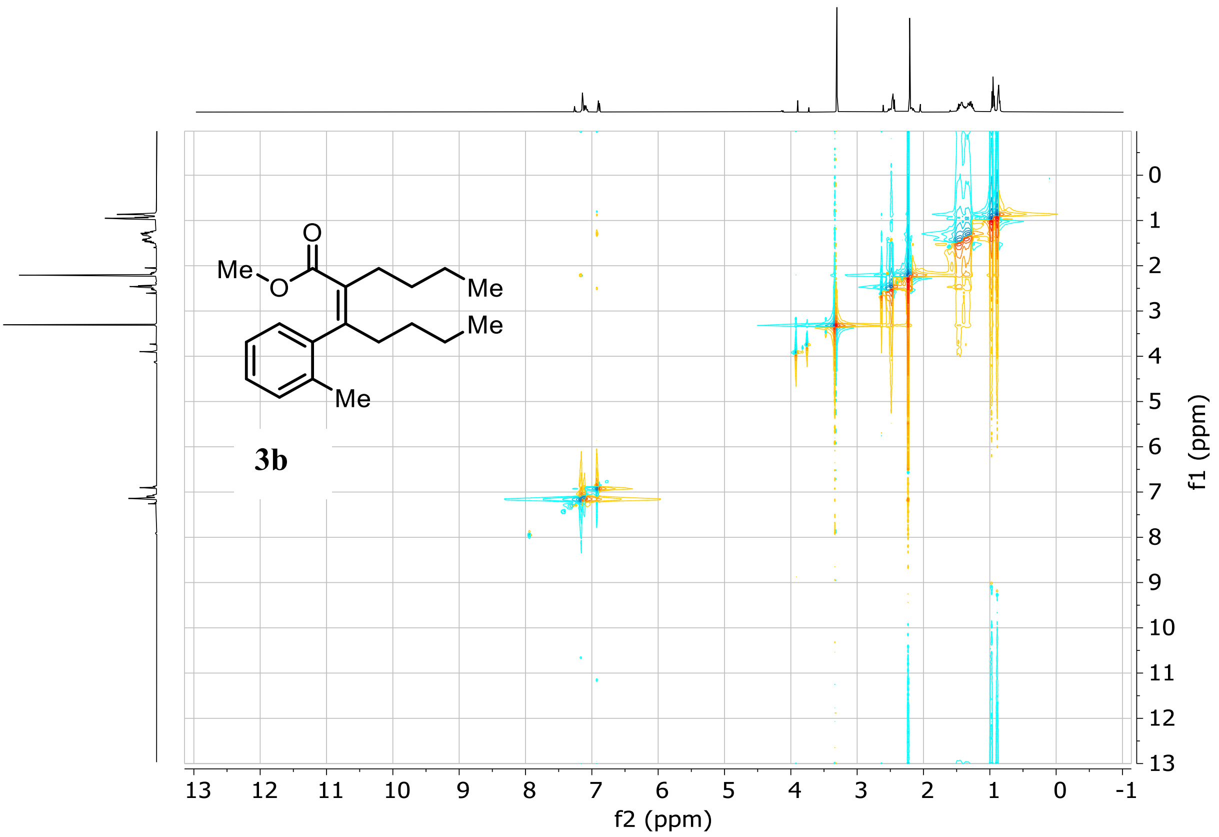
Me
O
O
Me
Me
Me
3b
f2 (ppm)
f1 (ppm)

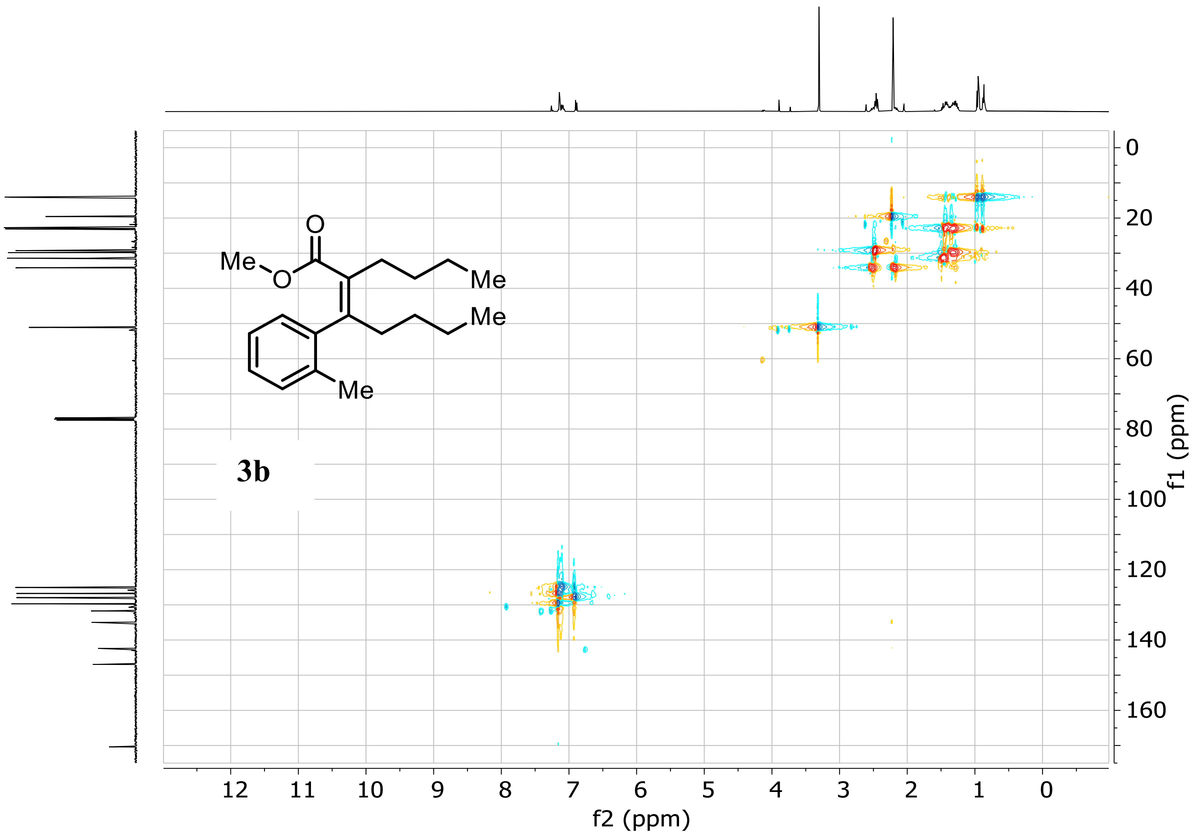

Me
O
O
Me
Me
Me
3b
f2 (ppm)
f1 (ppm)

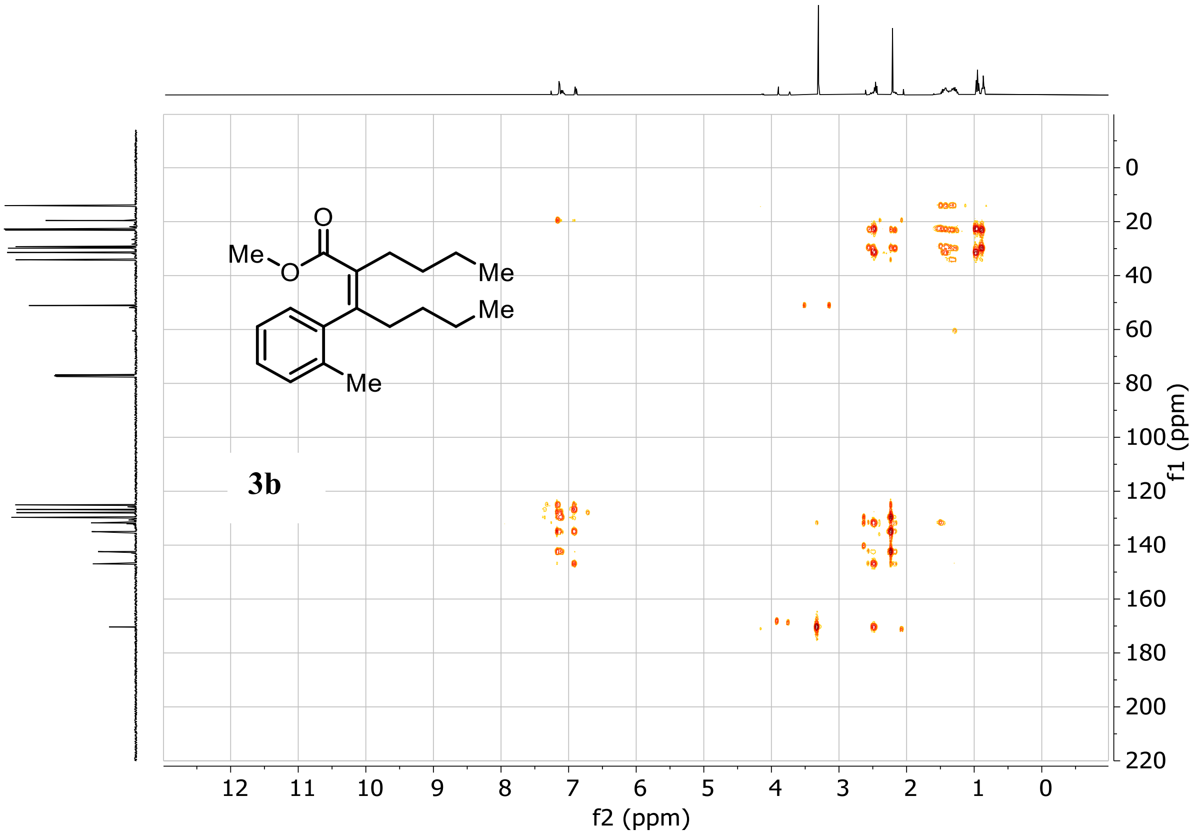

Me
O
O
Me
Me
Me
3b
f2 (ppm)
f1 (ppm)

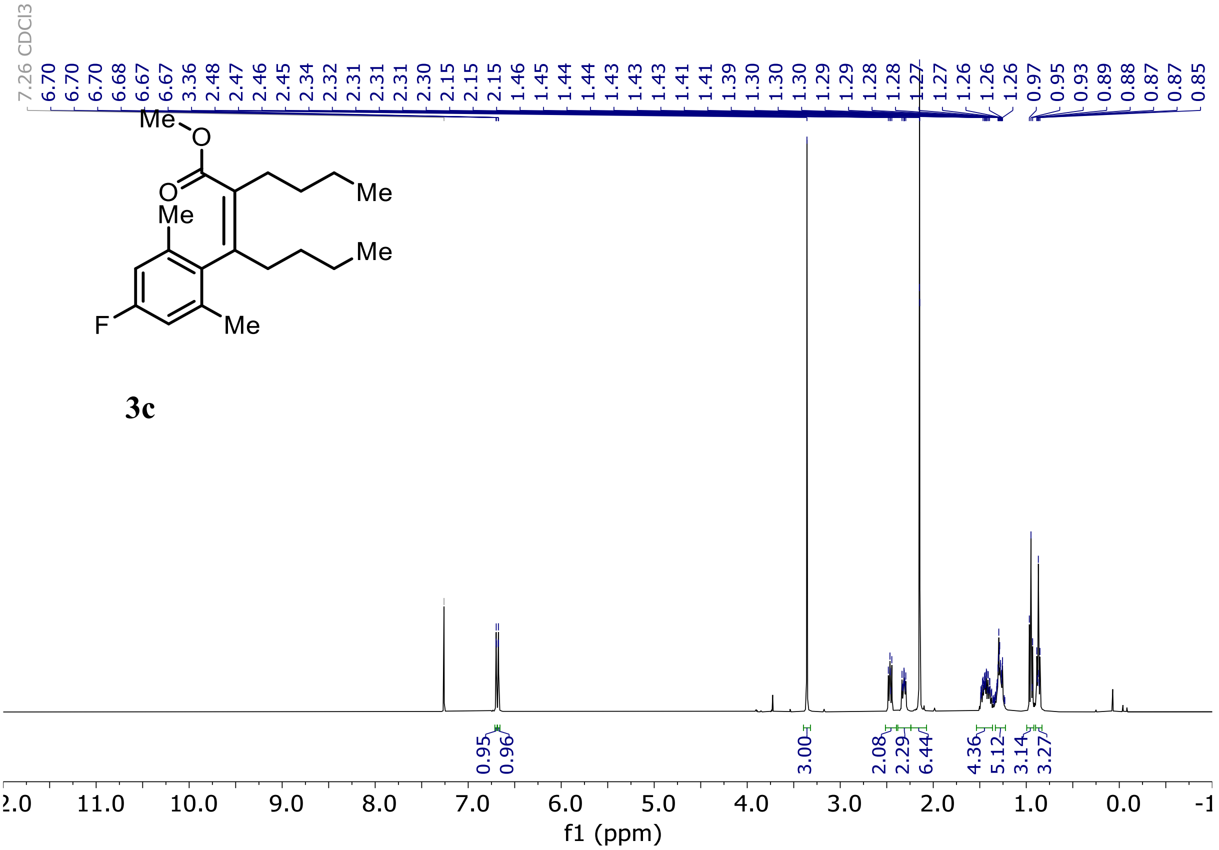
7.26 CDCl3
6.70
6.70
6.70
6.68
6.67
6.67
3.36
2.48
2.47
2.46
2.45
2.34
2.32
2.31
2.31
2.31
2.30
2.15
2.15
2.15
1.46
1.45
1.44
1.44
1.43
1.43
1.43
1.41
1.41
1.39
1.30
1.30
1.30
1.29
1.29
1.28
1.28
1.27
1.27
1.26
1.26
1.26
0.97
0.95
0.93
0.89
0.88
0.87
0.87
0.85
Me
O
O
Me
Me
Me
F
Me
3c
0.95
0.96
3.00
2.08
2.29
6.44
4.36
5.12
3.14
3.27
f1 (ppm)

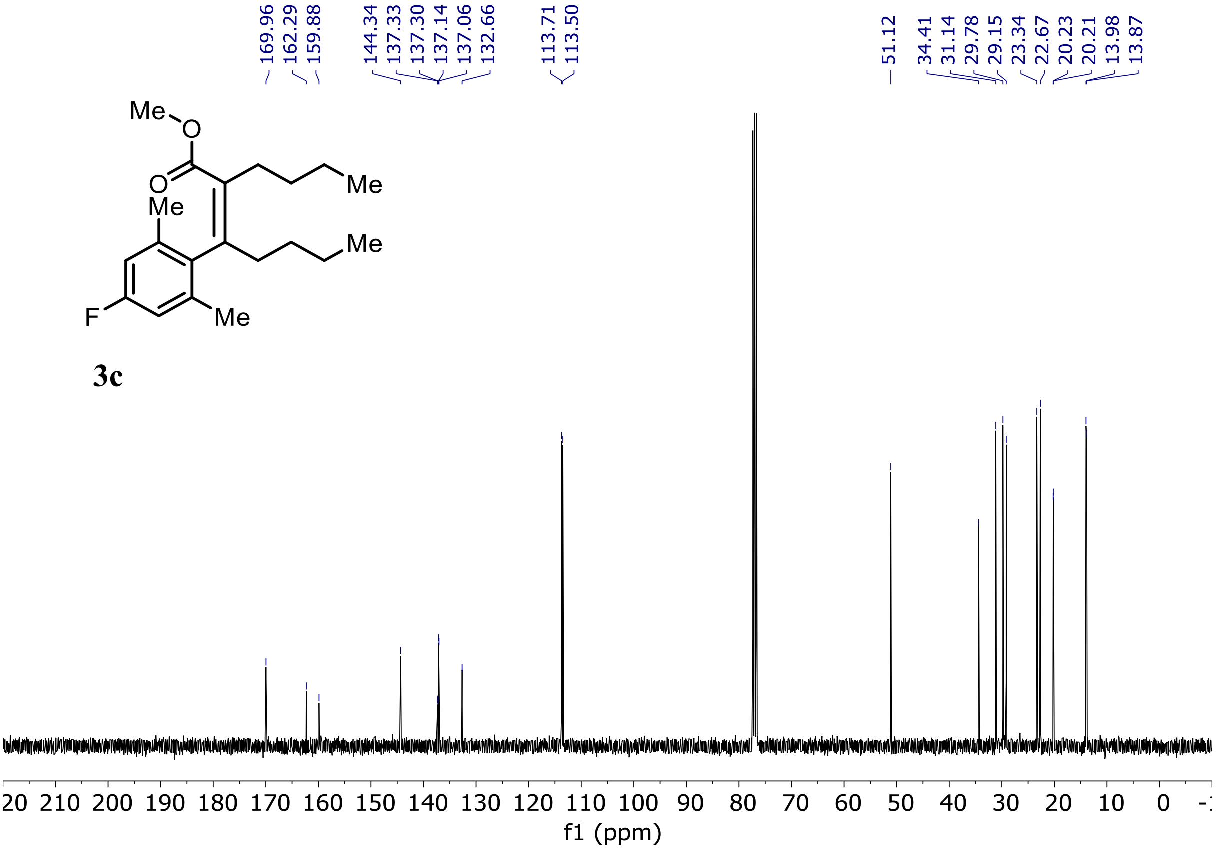
169.96
162.29
159.88
144.34
137.33
137.30
137.14
137.06
132.66
113.71
113.50
51.12
34.41
31.14
29.78
29.15
23.34
22.67
20.23
20.21
13.98
13.87
Me
O
O
Me
Me
Me
F
Me
3c
f1 (ppm)

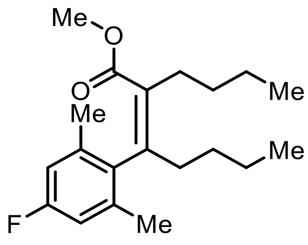

Me
O
O
Me
Me
Me
F
Me

3c

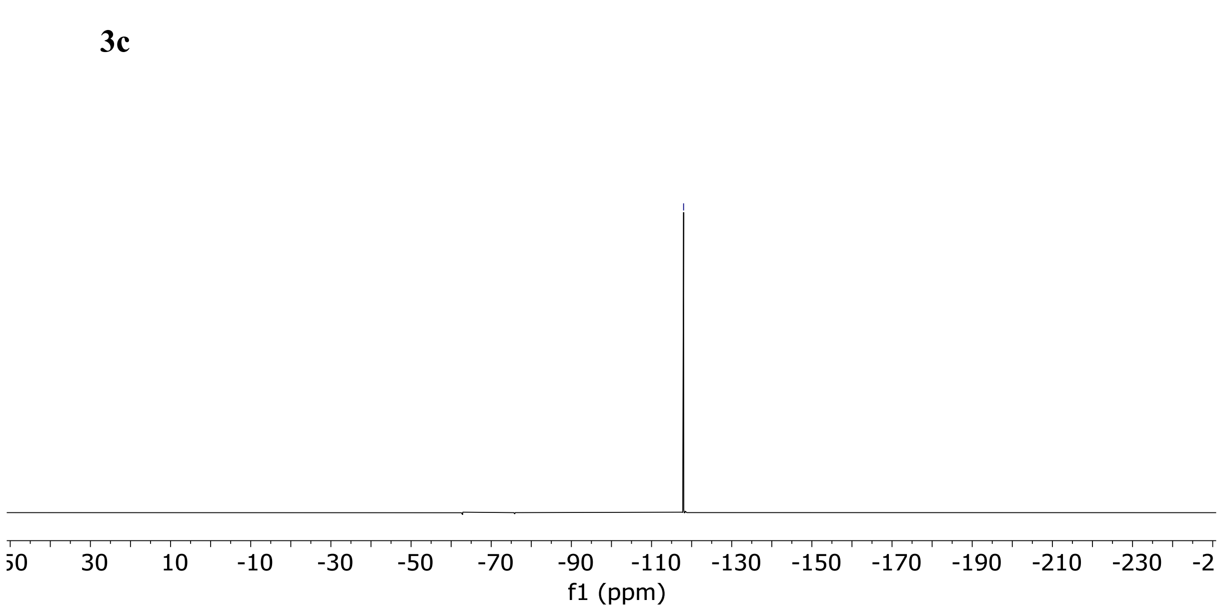

-117.99
50
30
10
-10
-30
-50
-70
-90
-110
-130
-150
-170
-190
-210
-230
-2
f1 (ppm)

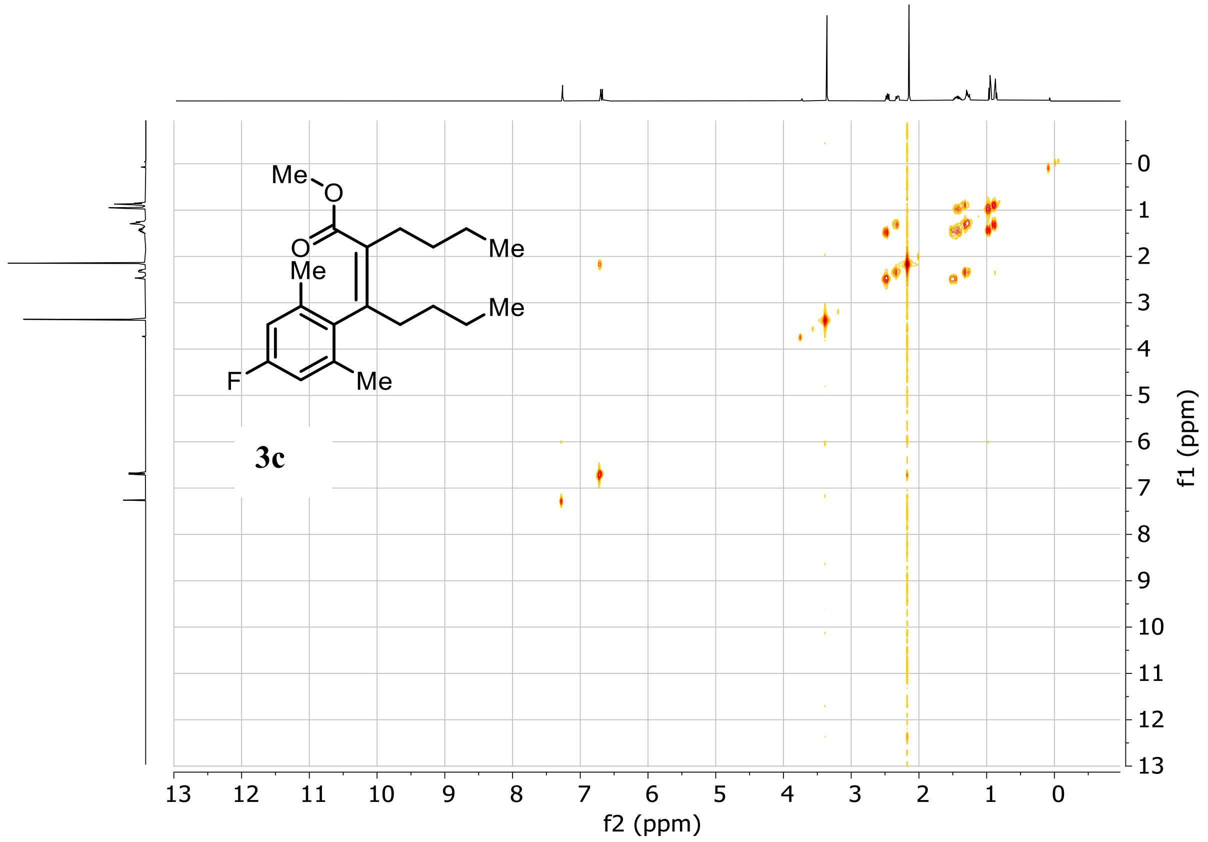

Me
O
O
Me
Me
Me
F
Me
3c
13
12
11
10
9
8
7
6
5
4
3
2
1
0
f2 (ppm)
f1 (ppm)

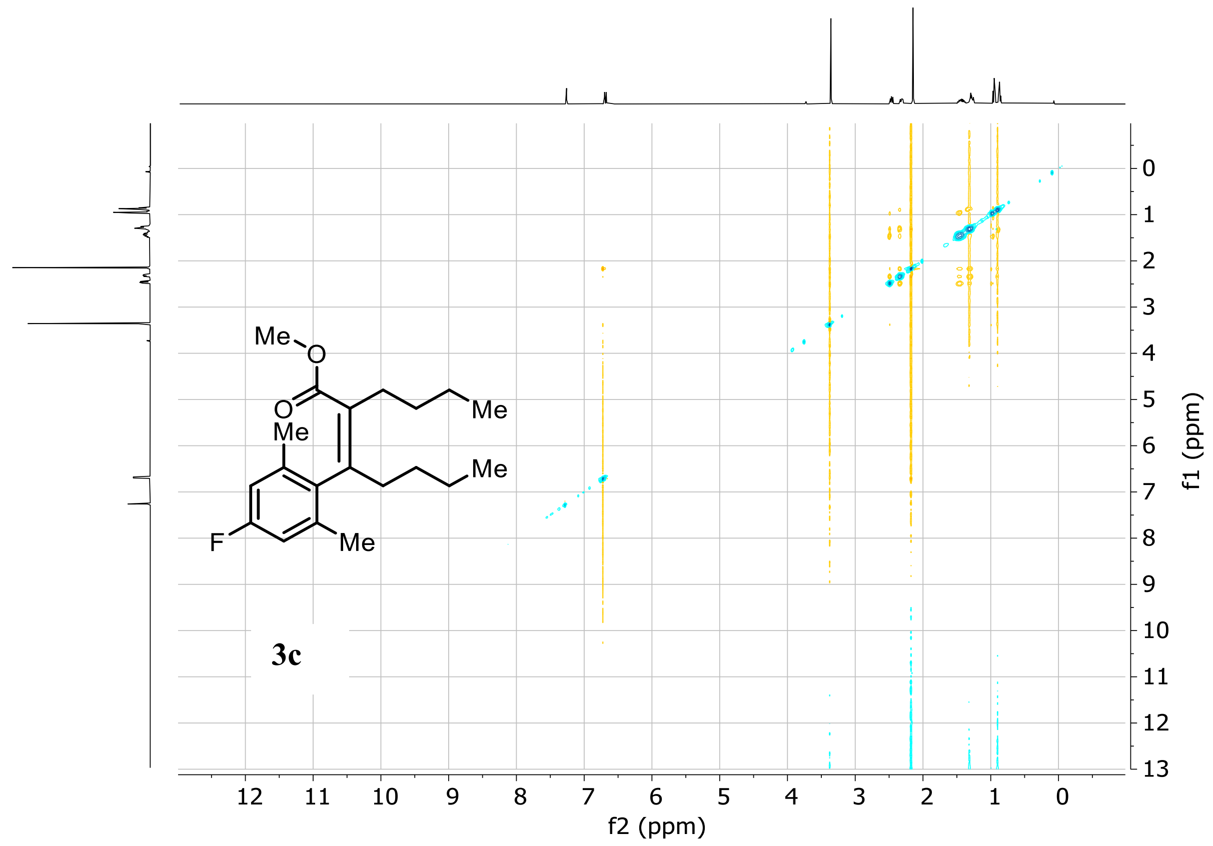
Me
O
O
Me
Me
Me
F
Me
3c
f1 (ppm)
f2 (ppm)

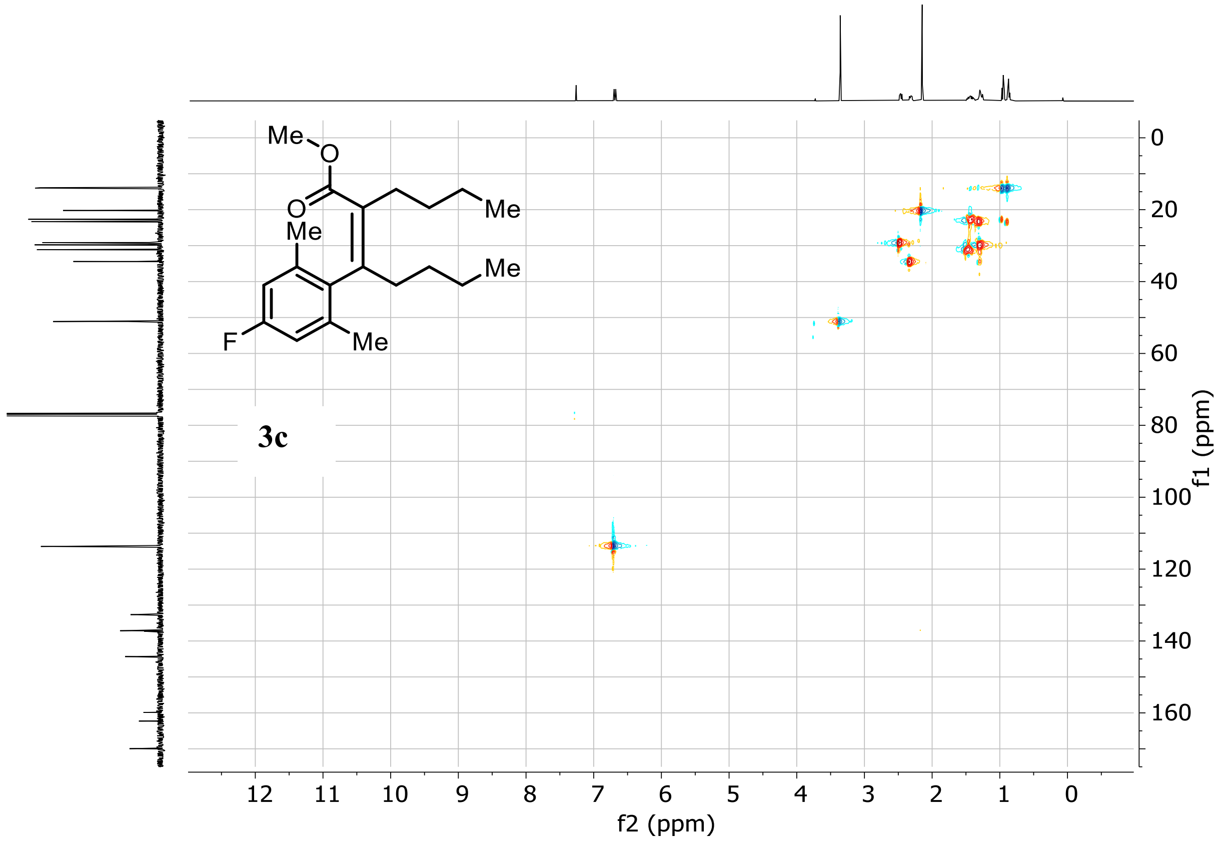
Me
O
O
Me
Me
Me
F
Me
3c
f1 (ppm)
f2 (ppm)

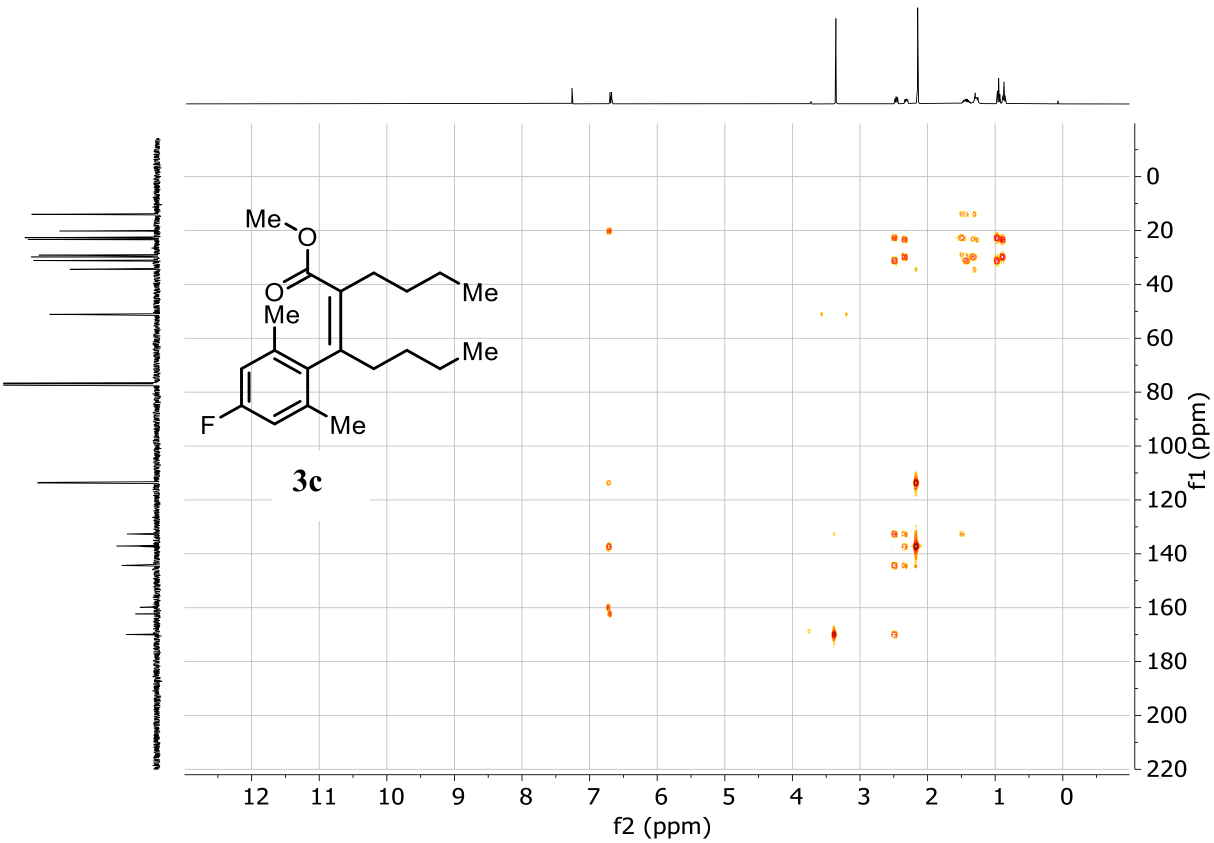
Me
O
O
Me
Me
Me
F
Me
3c
f2 (ppm)
f1 (ppm)

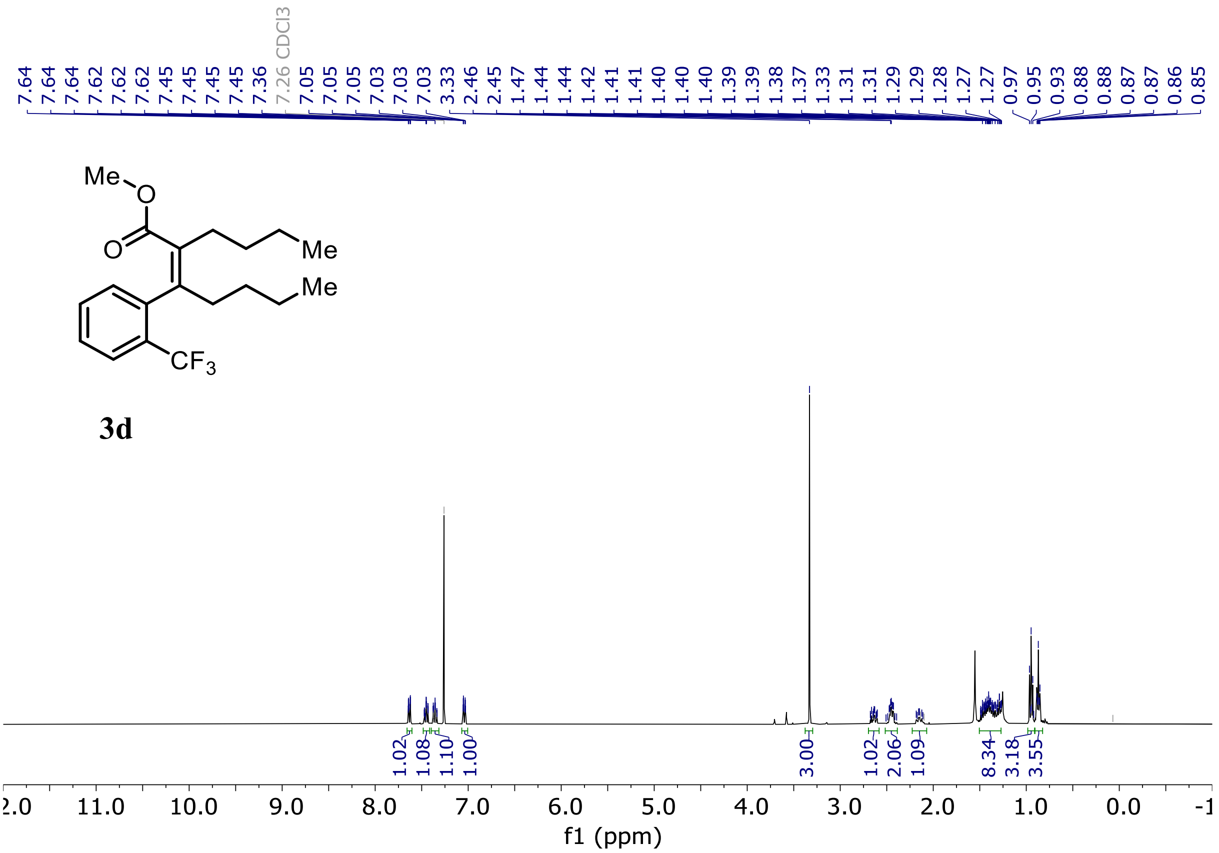
7.64
7.64
7.64
7.62
7.62
7.62
7.45
7.45
7.45
7.45
7.36
7.26 CDCl3
7.05
7.05
7.05
7.03
7.03
7.03
3.33
2.46
2.45
1.47
1.44
1.44
1.42
1.41
1.41
1.40
1.40
1.40
1.39
1.39
1.38
1.37
1.33
1.31
1.31
1.29
1.29
1.28
1.27
1.27
0.97
0.95
0.93
0.88
0.88
0.87
0.87
0.86
0.85
Me
O
O
Me
Me
CF3
3d
1.02
1.08
1.10
1.00
3.00
1.02
2.06
1.09
8.34
3.18
3.55
f1 (ppm)

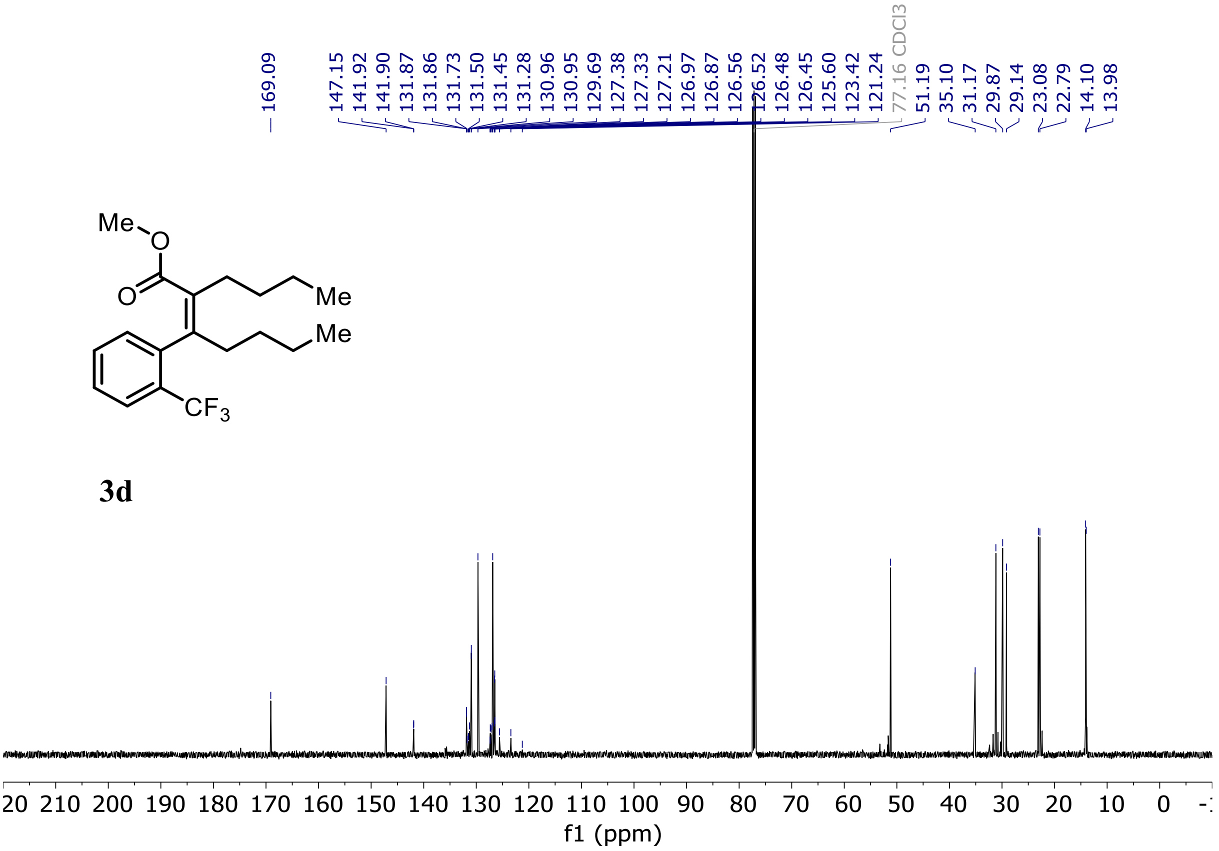
169.09
147.15
141.92
141.90
131.87
131.86
131.73
131.50
131.45
131.28
130.96
130.95
129.69
127.38
127.33
127.21
126.97
126.87
126.56
126.52
126.48
126.45
125.60
123.42
121.24
77.16 CDCl3
51.19
35.10
31.17
29.87
29.14
23.08
22.79
14.10
13.98
Me
O
O
Me
Me
CF3
3d
f1 (ppm)

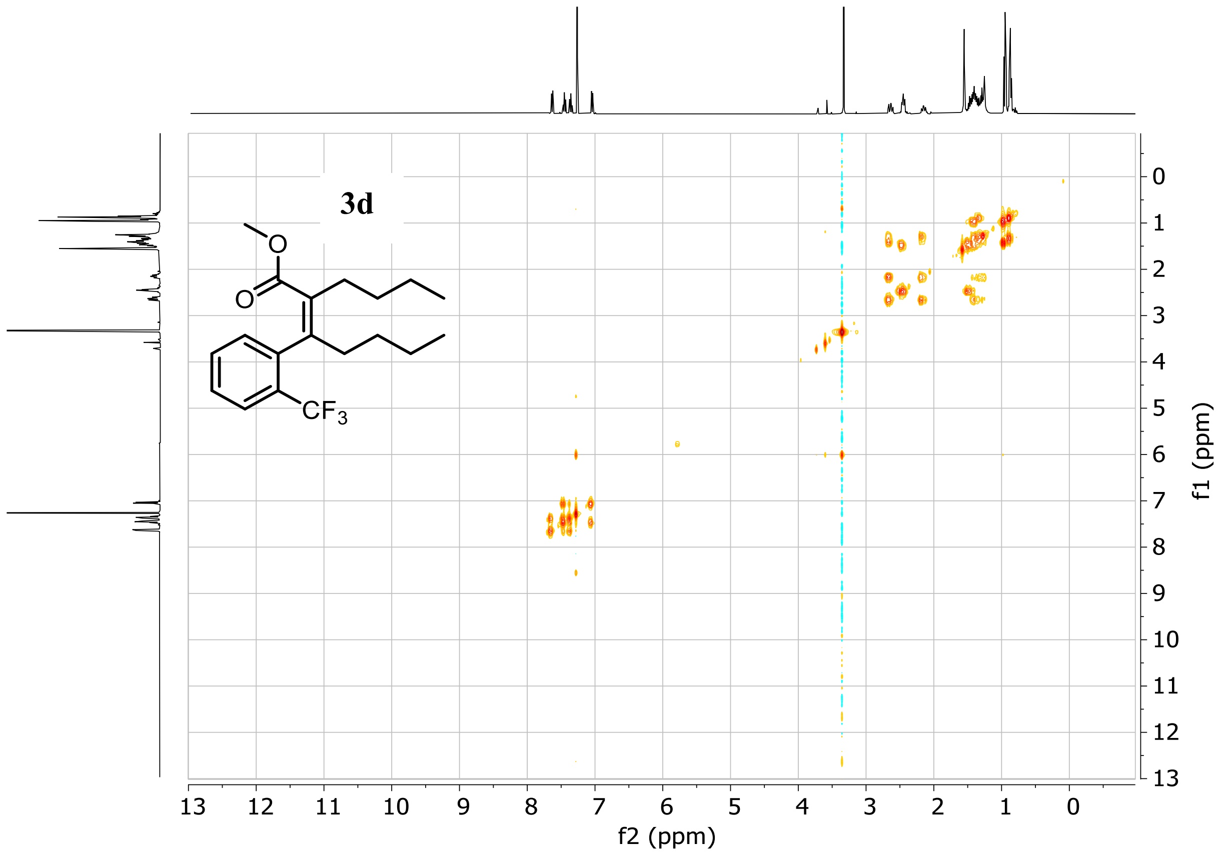
3d
O
O
CF3
f2 (ppm)
f1 (ppm)

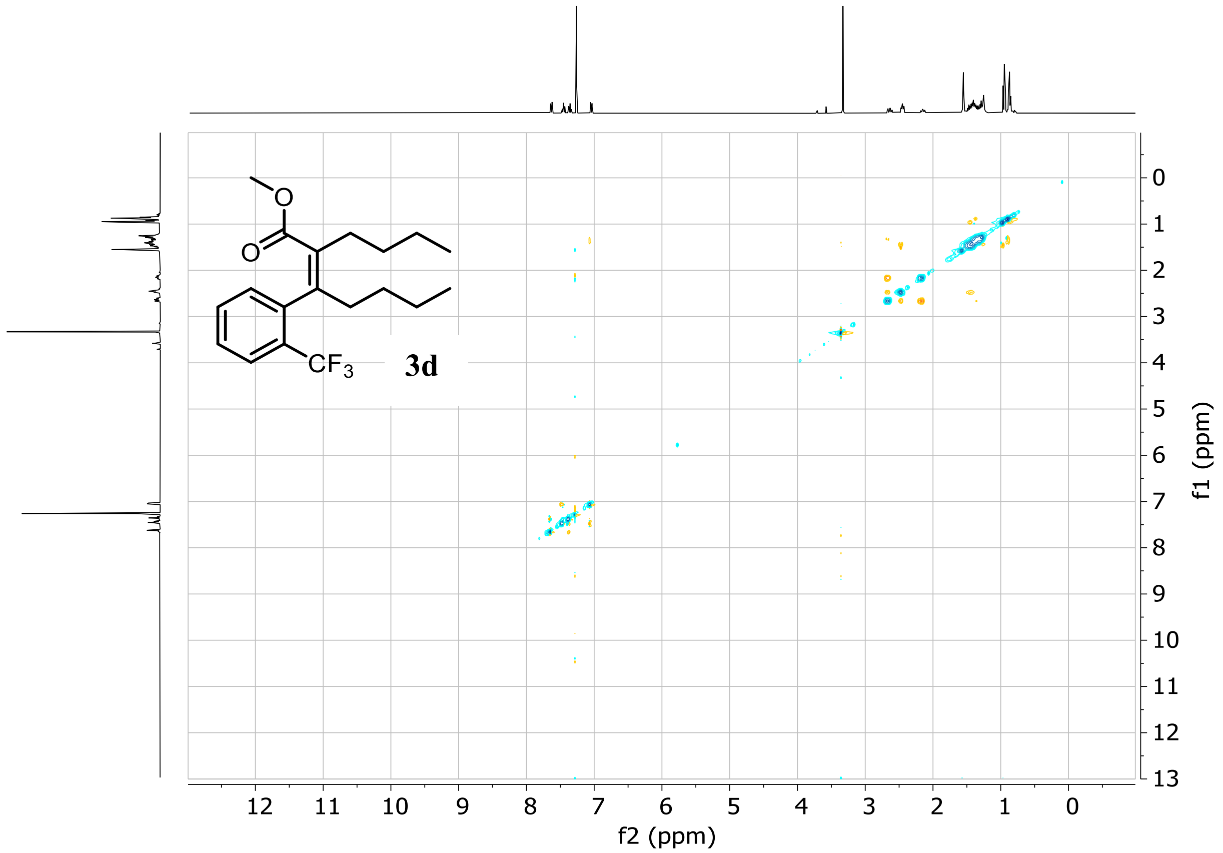
O
O
CF3
3d
f2 (ppm)
f1 (ppm)

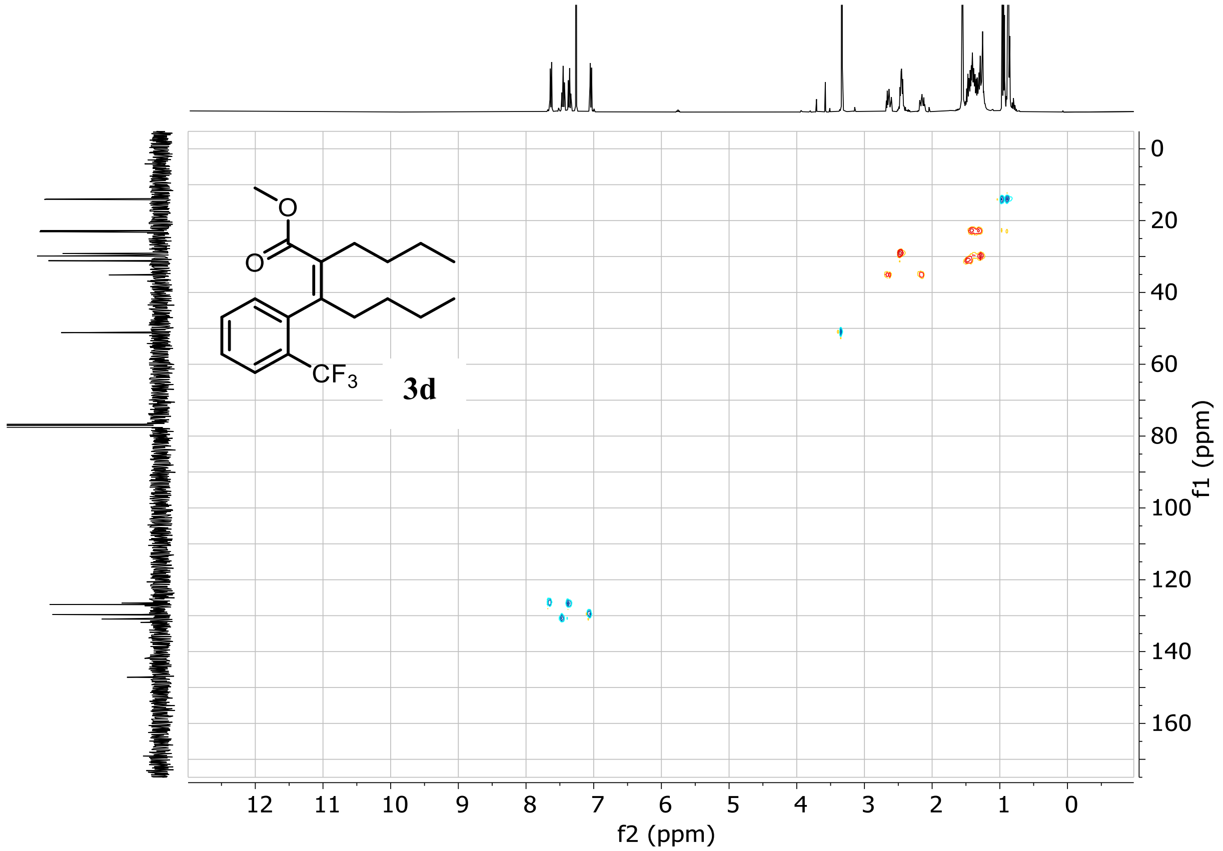

O
O
CF3
3d
f1 (ppm)
f2 (ppm)

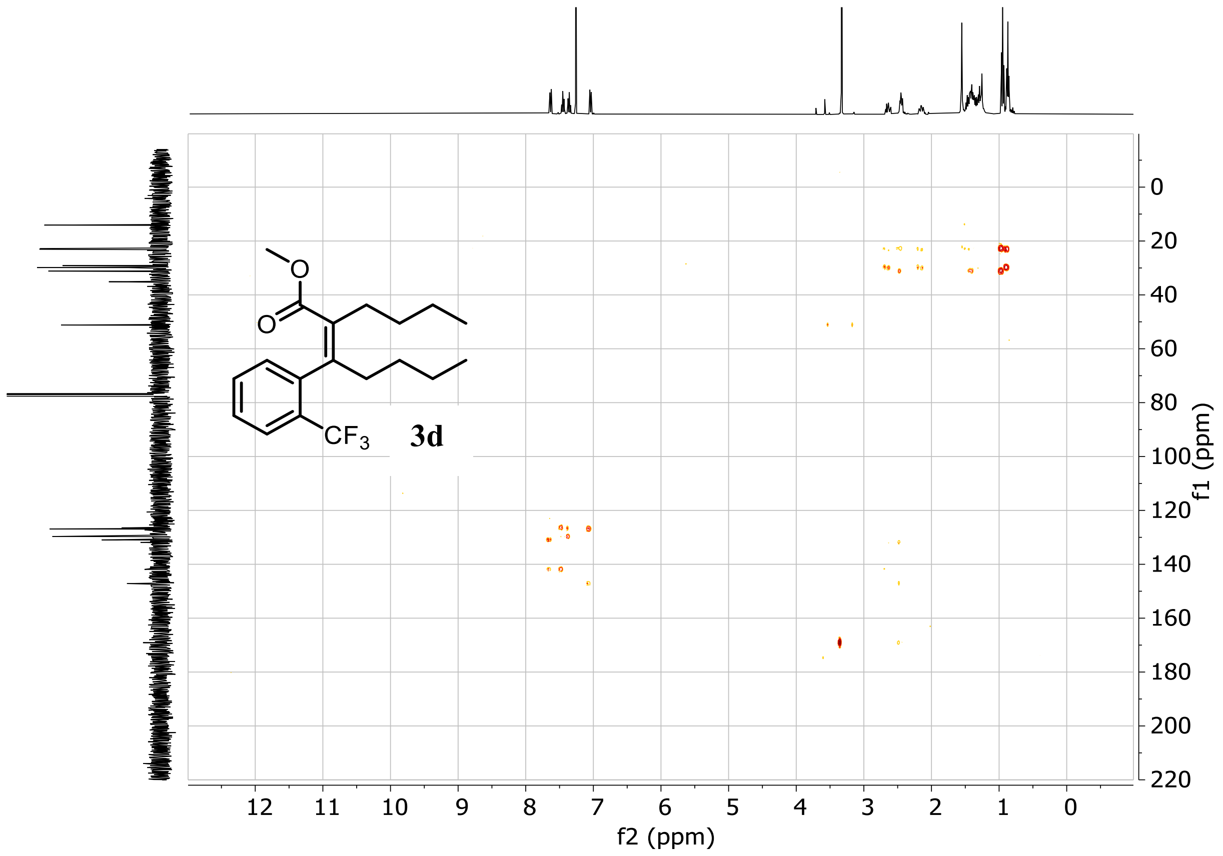

O
O
CF3
3d
f1 (ppm)
f2 (ppm)

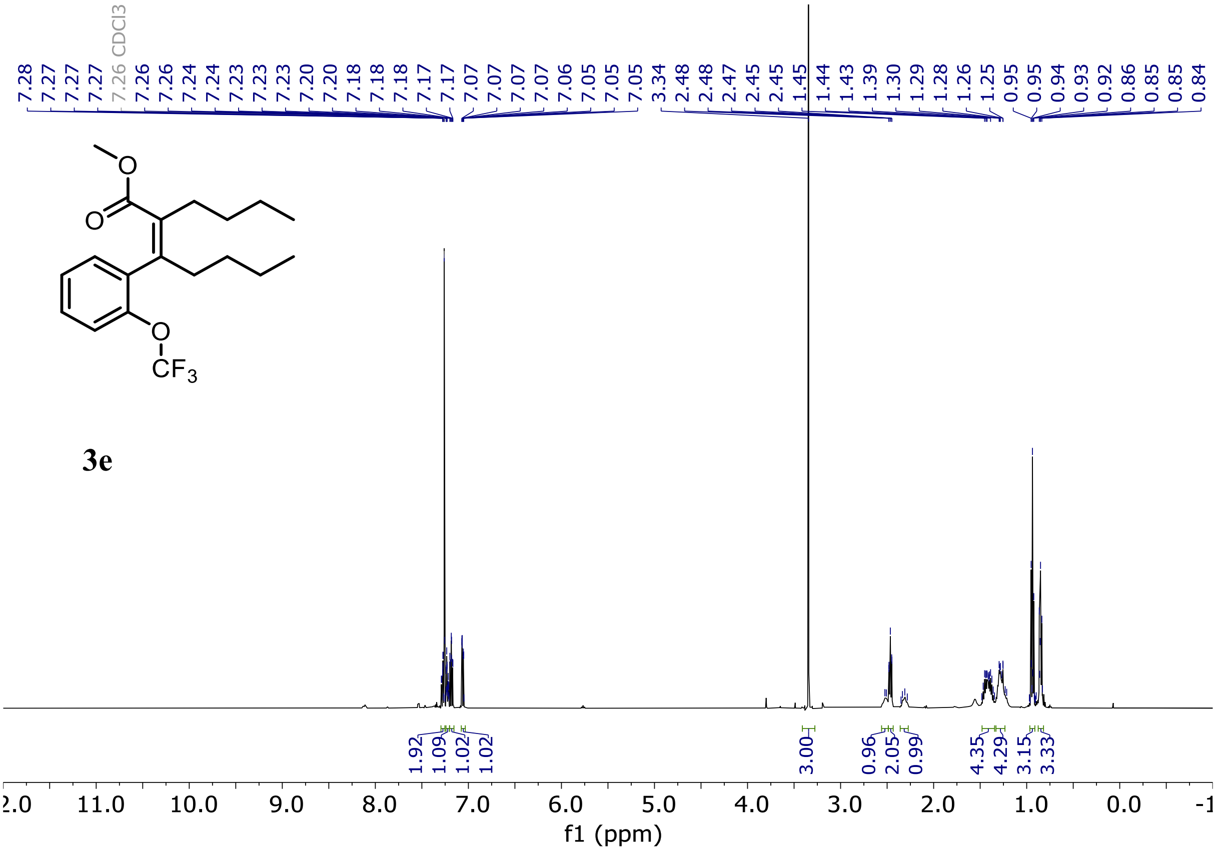
7.28 7.27 7.27 7.27 7.26 CDCl3 7.26 7.26 7.24 7.24 7.23 7.23 7.23 7.20 7.20 7.18 7.18 7.18 7.17 7.17 7.07 7.07 7.07 7.07 7.06 7.05 7.05 7.05 3.34 2.48 2.48 2.47 2.45 2.45 1.45 1.44 1.43 1.39 1.30 1.29 1.28 1.26 1.25 0.95 0.95 0.94 0.93 0.92 0.86 0.85 0.85 0.84
O
O
O
CF3
3e
1.92 1.09 1.02 1.02
3.00
0.96 2.05 0.99
4.35 4.29 3.15 3.33
2.0 11.0 10.0 9.0 8.0 7.0 6.0 5.0 4.0 3.0 2.0 1.0 0.0 -1
f1 (ppm)

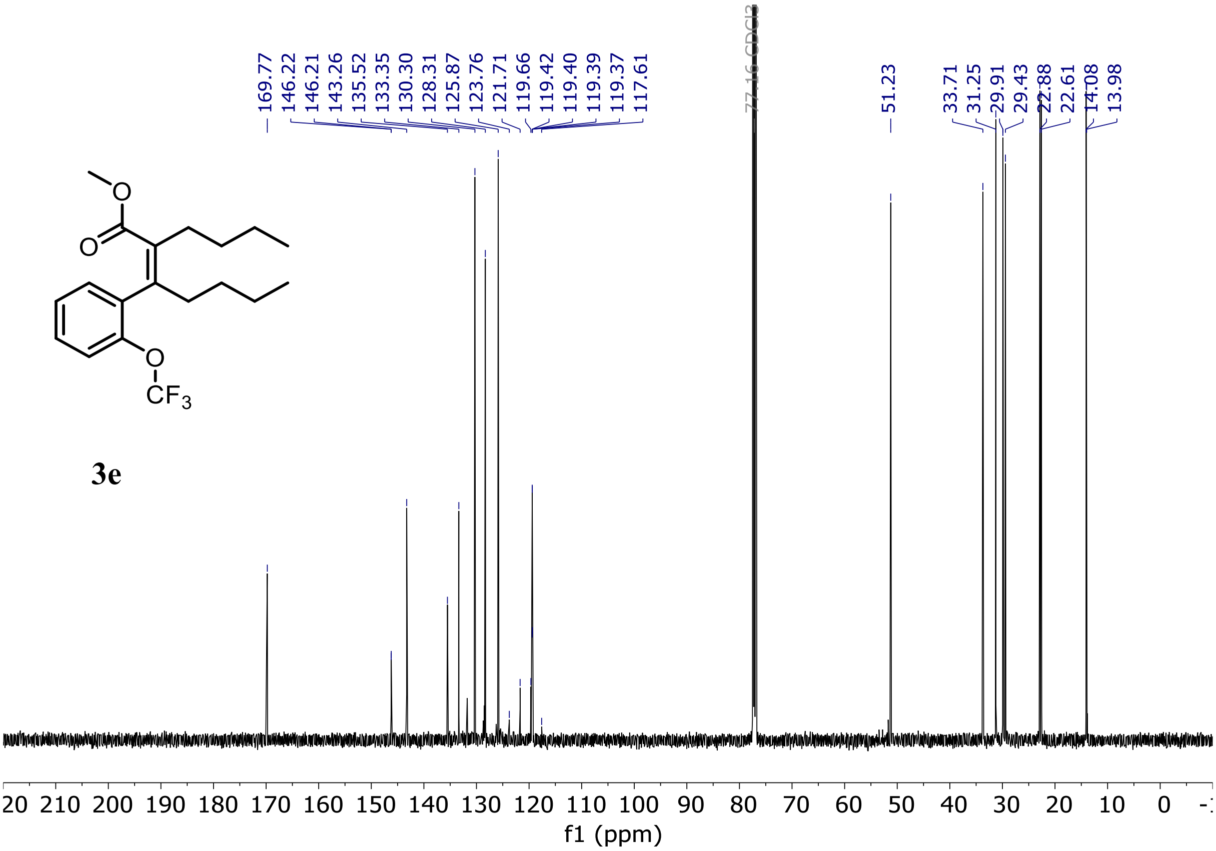
169.77 146.22 146.21 143.26 135.52 133.35 130.30 128.31 125.87 123.76 121.71 119.66 119.42 119.40 119.39 119.37 117.61
77.16 CDCl3
51.23
33.71 31.25 29.91 29.43 22.88 22.61 14.08 13.98
O
O
O
CF3
3e
20 210 200 190 180 170 160 150 140 130 120 110 100 90 80 70 60 50 40 30 20 10 0 -1
f1 (ppm)

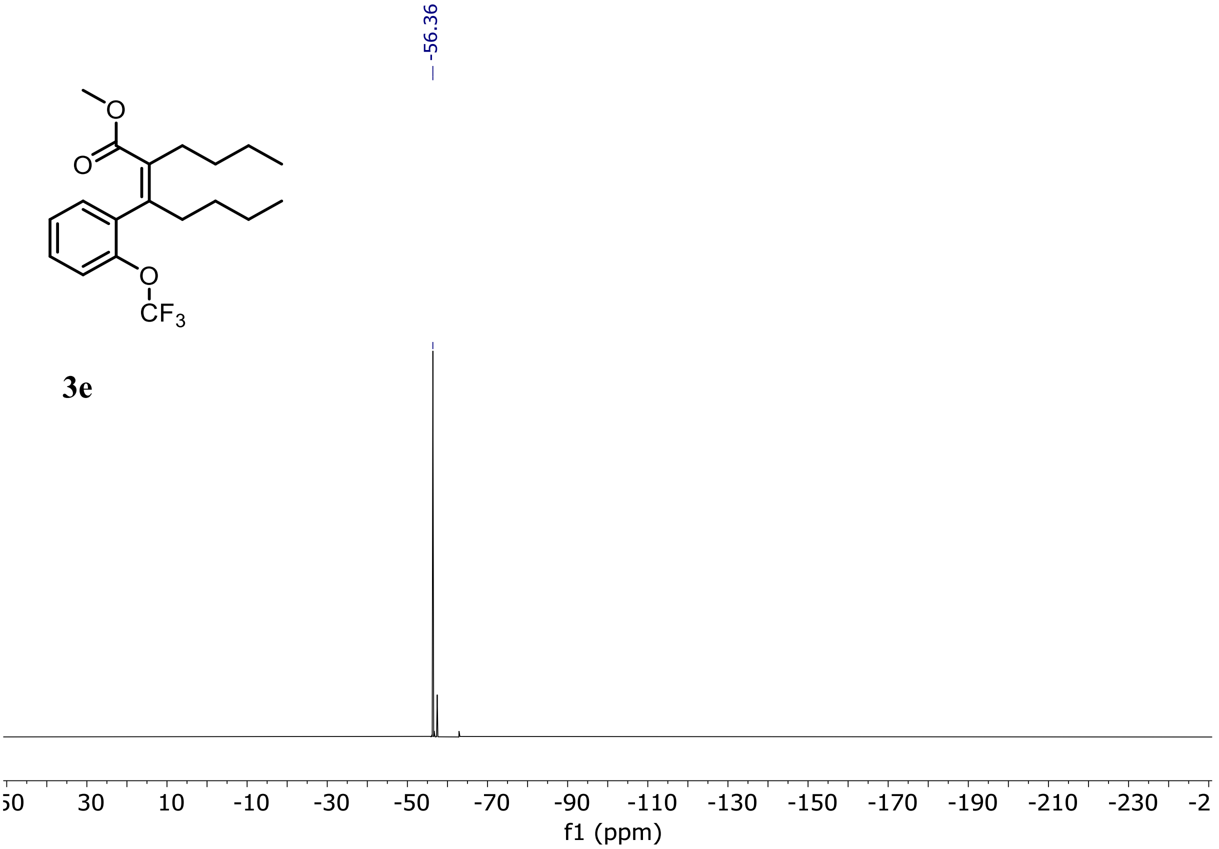
-56.36
O
O
O
CF3
3e
50
30
10
-10
-30
-50
-70
-90
-110
-130
-150
-170
-190
-210
-230
-2
f1 (ppm)

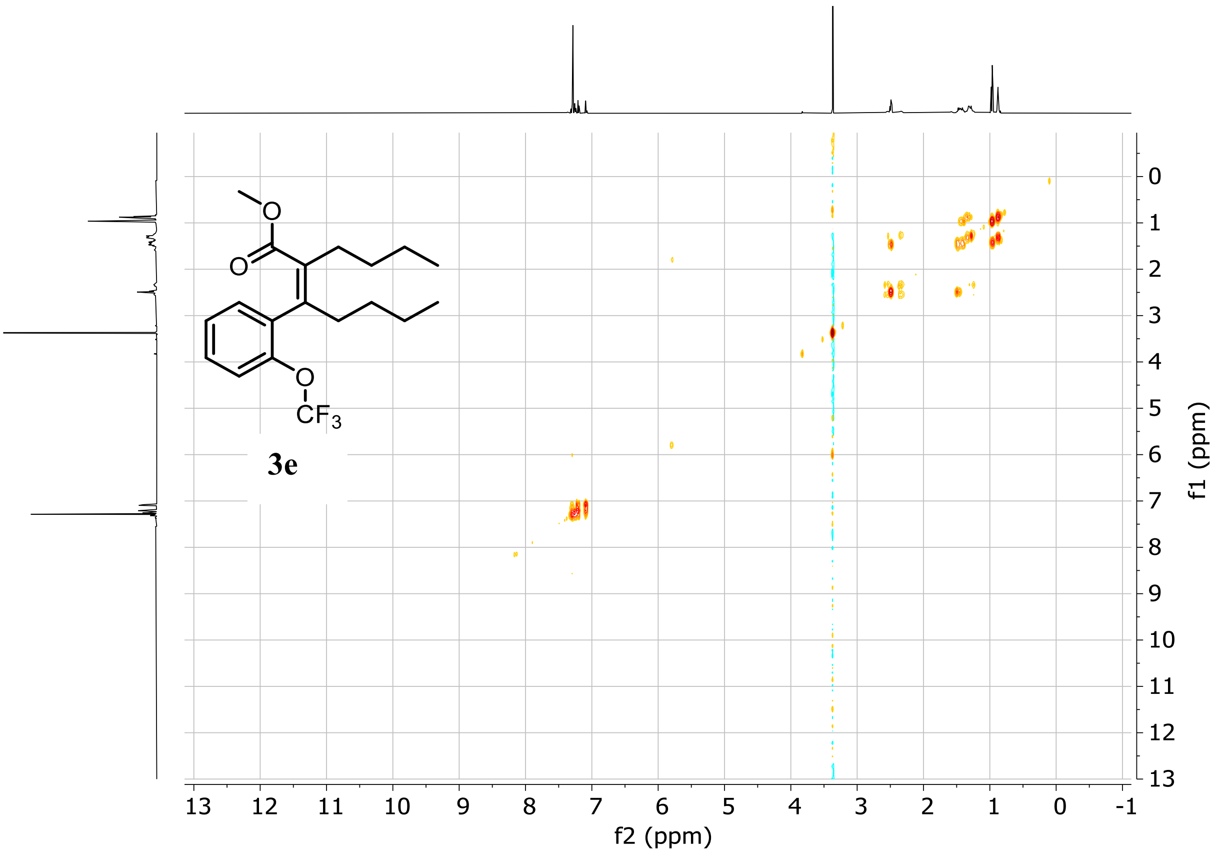
O
O
O
CF3
3e
13
12
11
10
9
8
7
6
5
4
3
2
1
0
-1
f2 (ppm)
f1 (ppm)

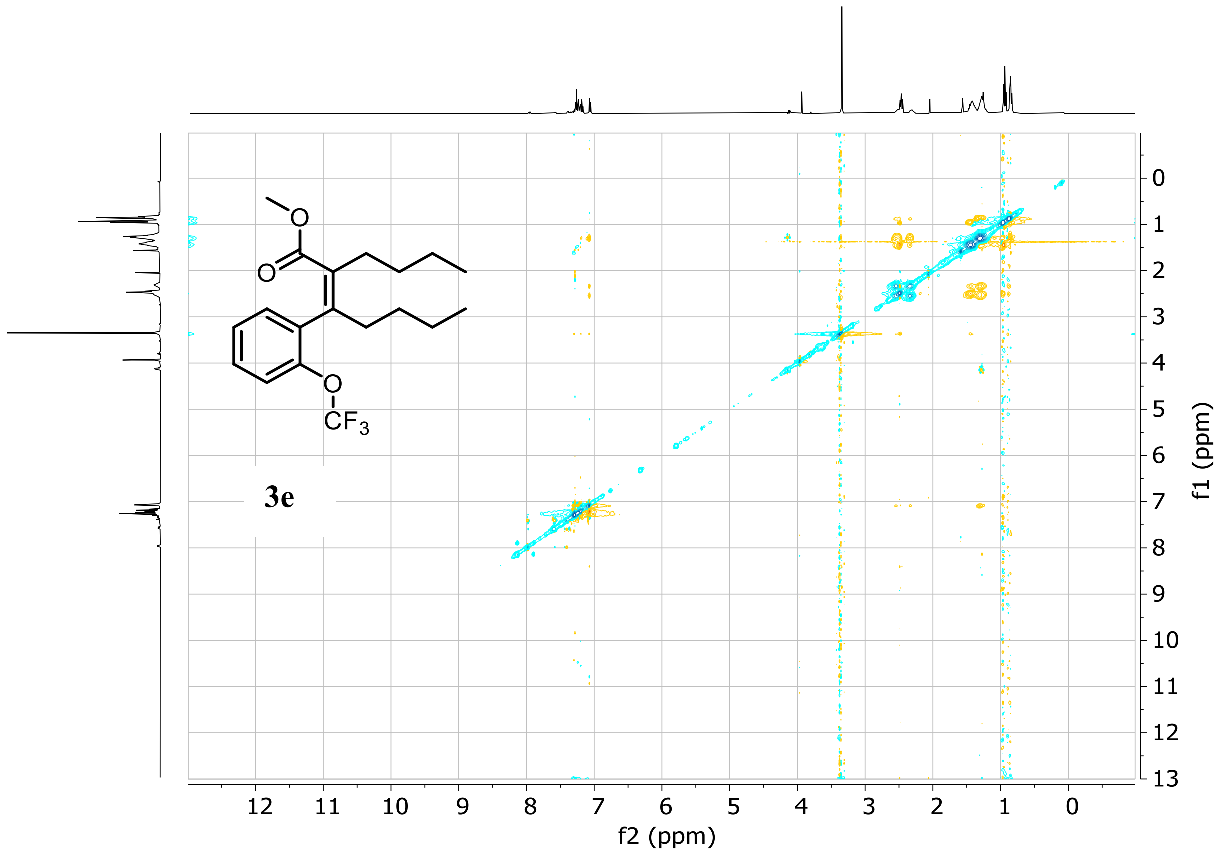
O
O
CF3
3e
f2 (ppm)
f1 (ppm)

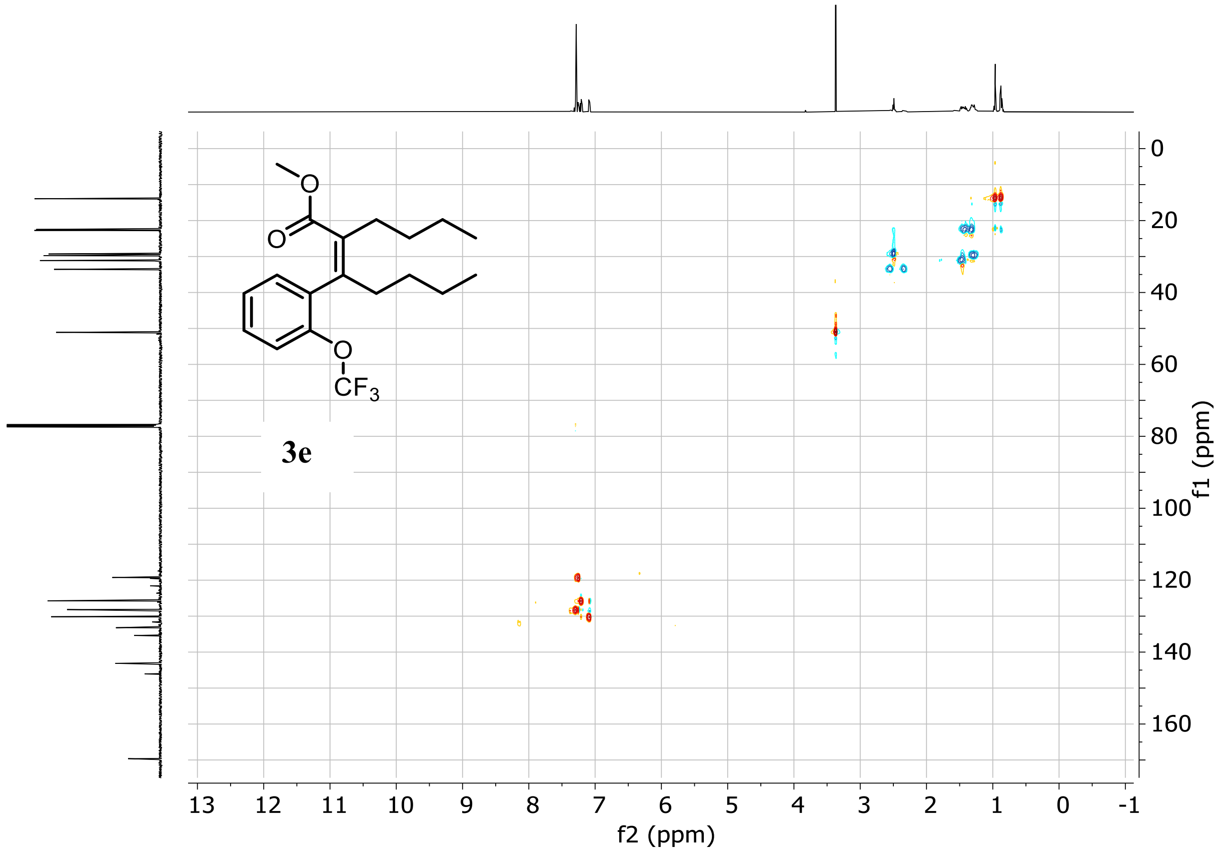
O
O
CF3
3e
f2 (ppm)
f1 (ppm)

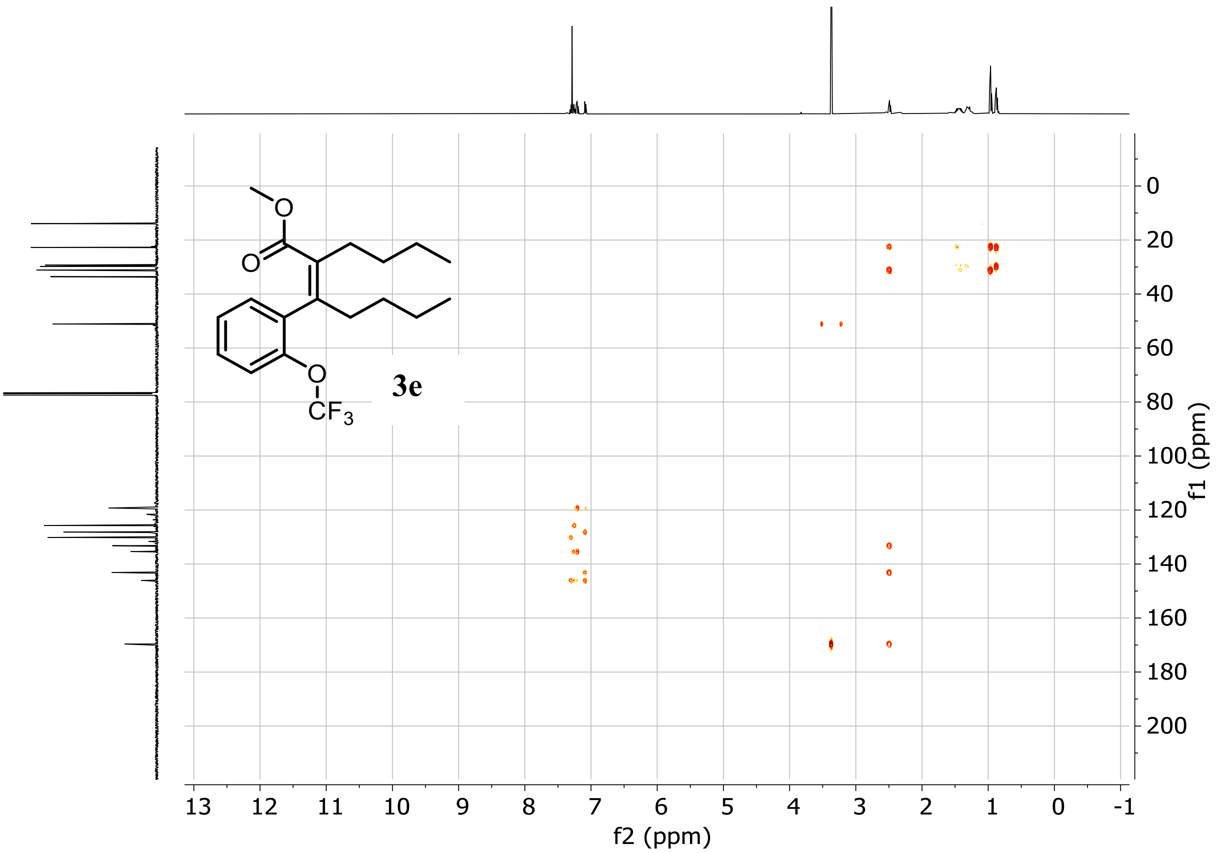
O
O
O
CF3
3e
0
20
40
60
80
100
120
140
160
180
200
f1 (ppm)
13
12
11
10
9
8
7
6
5
4
3
2
1
0
-1
f2 (ppm)

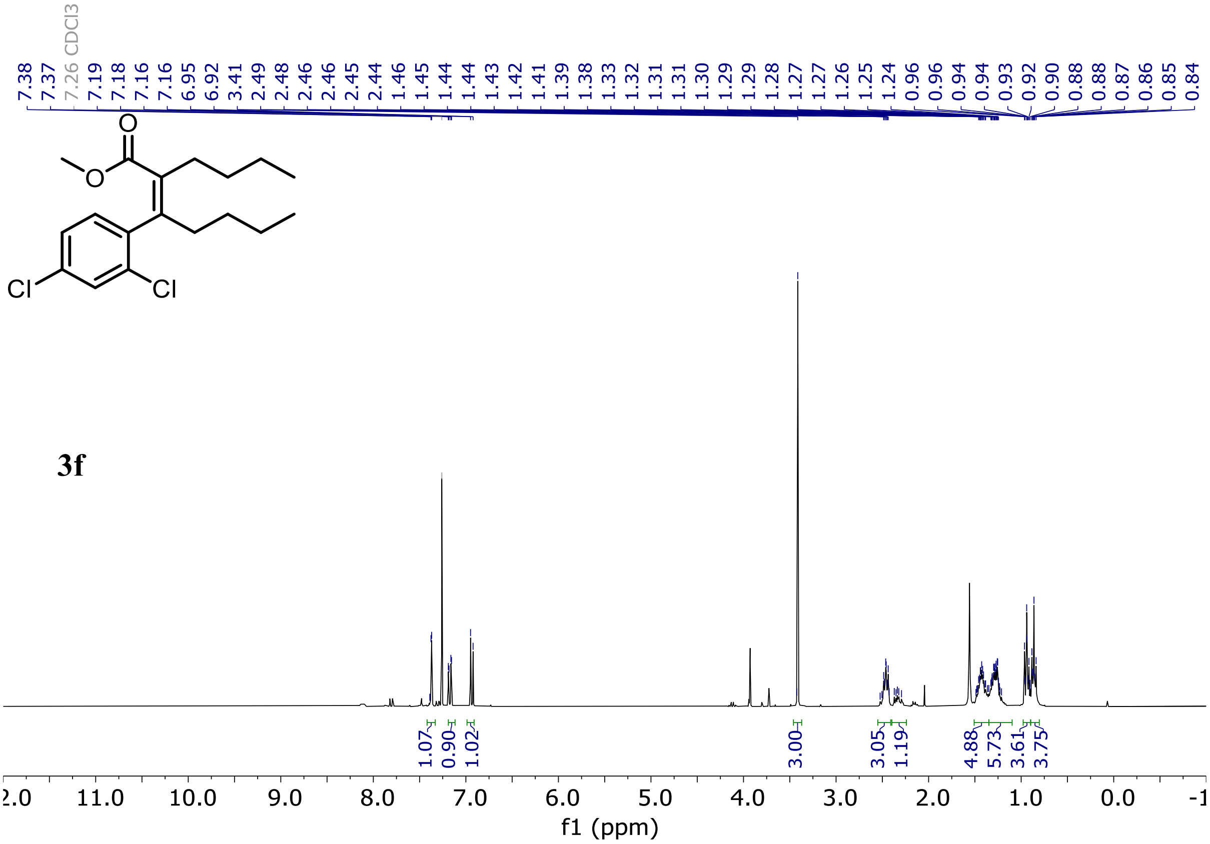
7.38 7.37 7.26 CDCl3 7.19 7.18 7.16 7.16 6.95 6.92 3.41 2.49 2.48 2.46 2.46 2.45 2.44 1.46 1.45 1.44 1.44 1.43 1.42 1.41 1.39 1.38 1.33 1.32 1.31 1.31 1.30 1.29 1.29 1.28 1.27 1.27 1.26 1.25 1.24 0.96 0.96 0.94 0.94 0.93 0.92 0.90 0.88 0.88 0.87 0.86 0.85 0.84
O
O
Cl
Cl
3f
1.07 0.90 1.02 3.00 3.05 1.19 4.88 5.73 3.61 3.75
11.0 10.0 9.0 8.0 7.0 6.0 5.0 4.0 3.0 2.0 1.0 0.0
f1 (ppm)

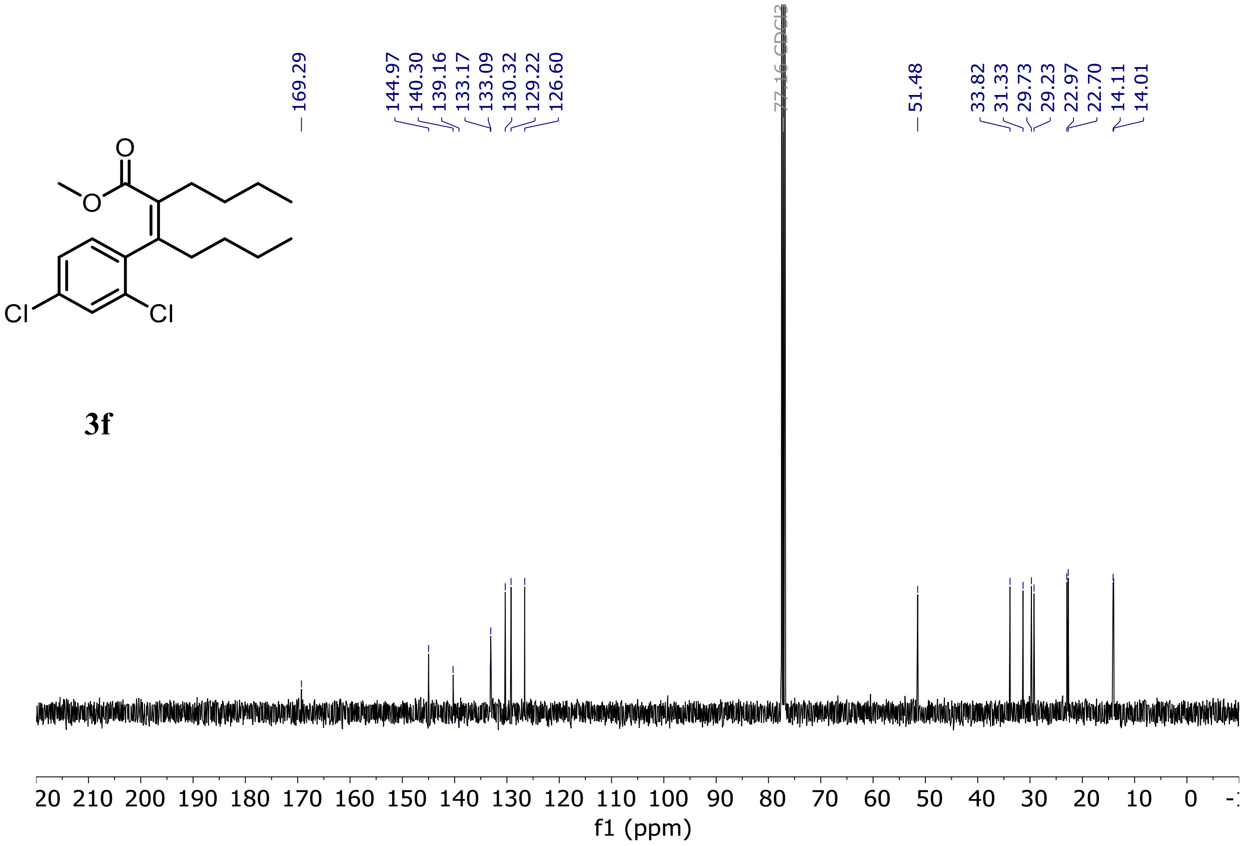
169.29
144.97 140.30 139.16 133.17 133.09 130.32 129.22 126.60
77.16 CDCl3
51.48
33.82 31.33 29.73 29.23 22.97 22.70 14.11 14.01
O
O
Cl
Cl
3f
210 200 190 180 170 160 150 140 130 120 110 100 90 80 70 60 50 40 30 20 10 0
f1 (ppm)

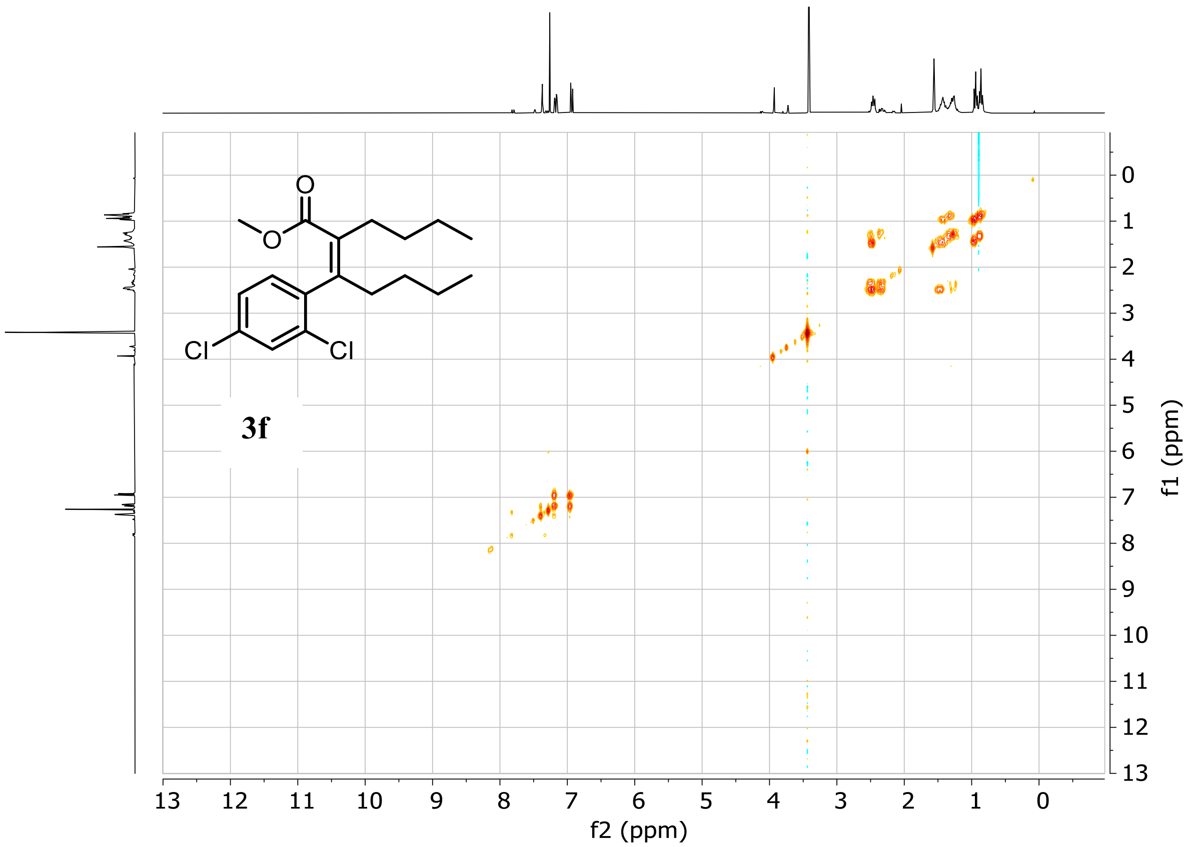
O
O
Cl
Cl
3f
f2 (ppm)
f1 (ppm)

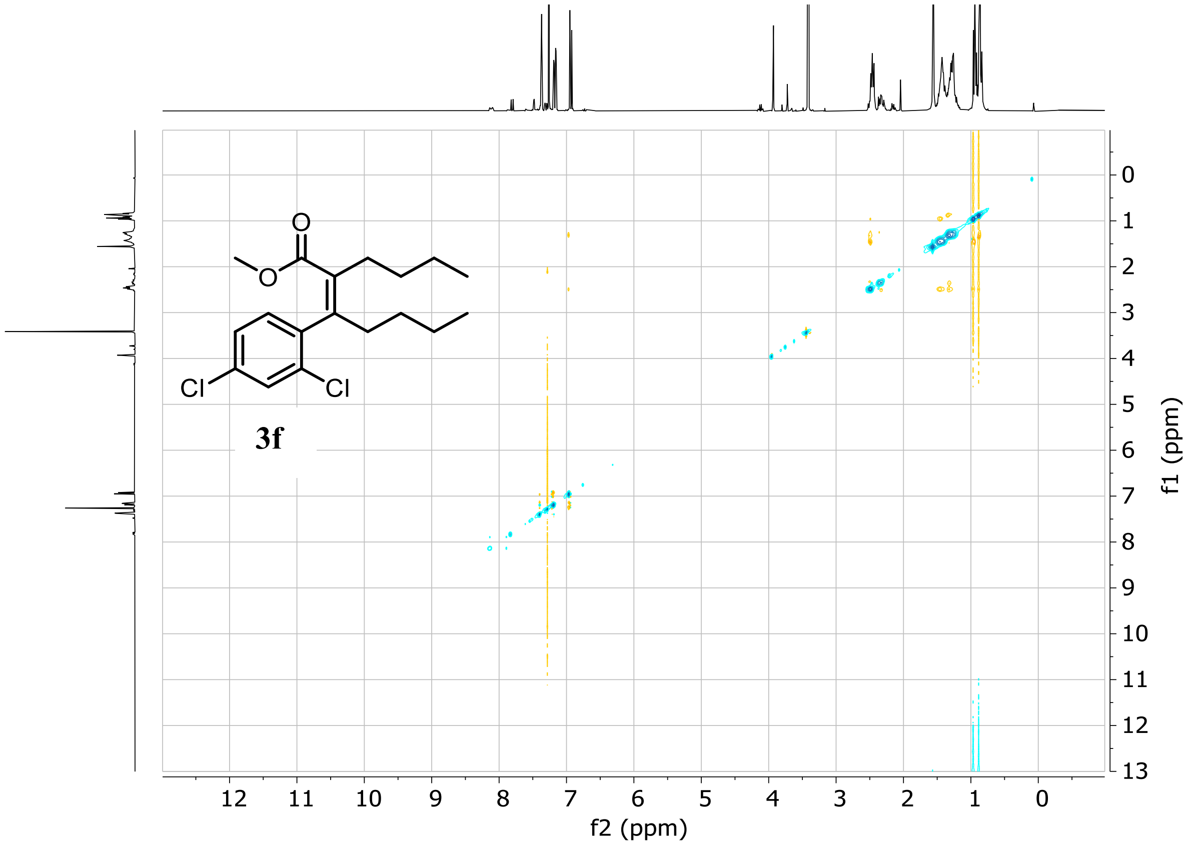
O
O
Cl
Cl
3f
f2 (ppm)
f1 (ppm)

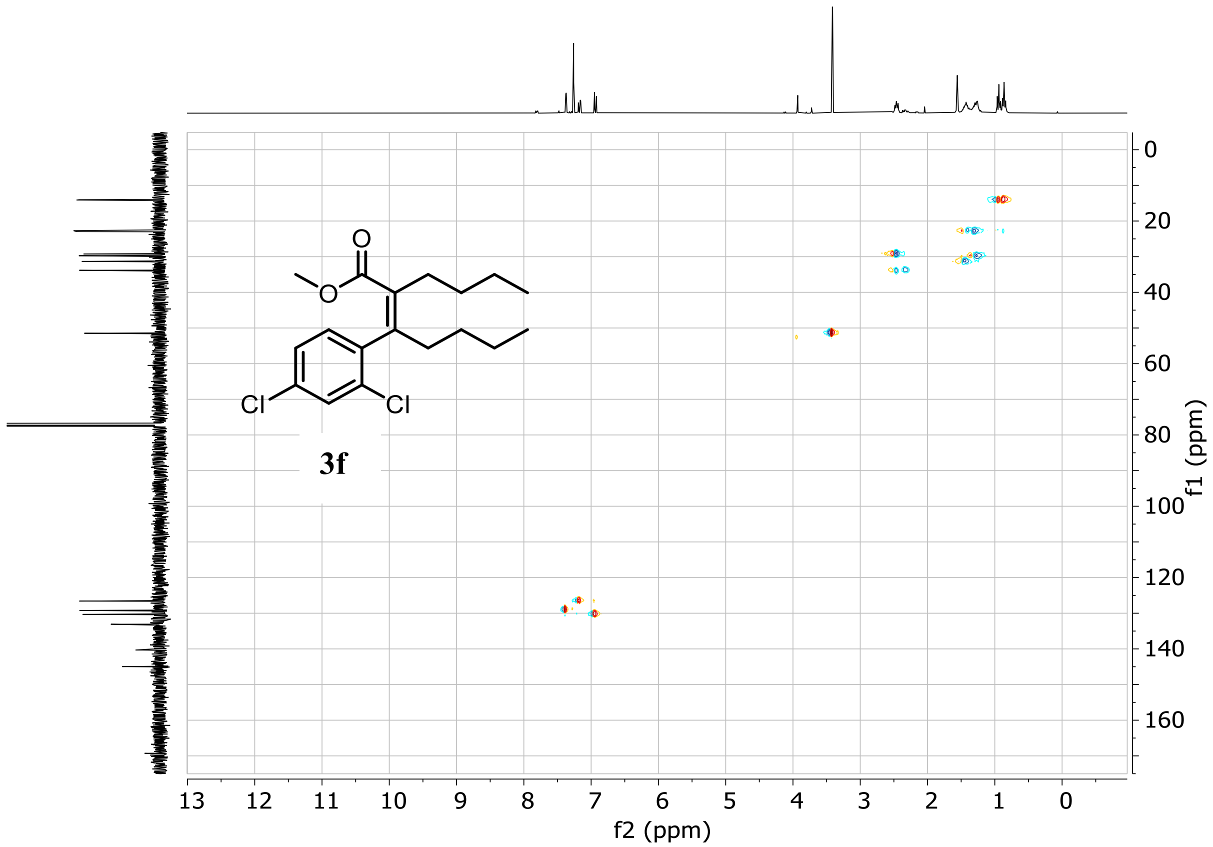
O
O
Cl
Cl
3f
f2 (ppm)
f1 (ppm)

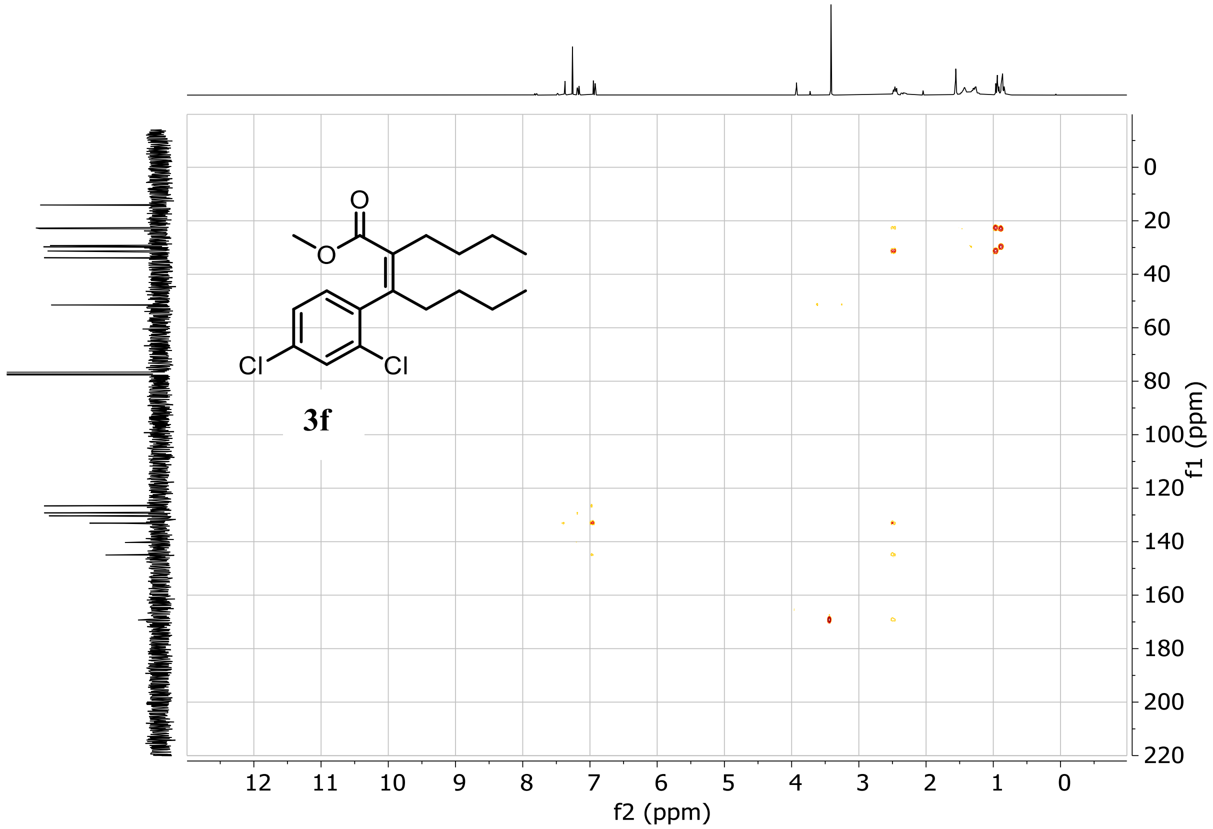
O
O
Cl
Cl
3f
f2 (ppm)
f1 (ppm)

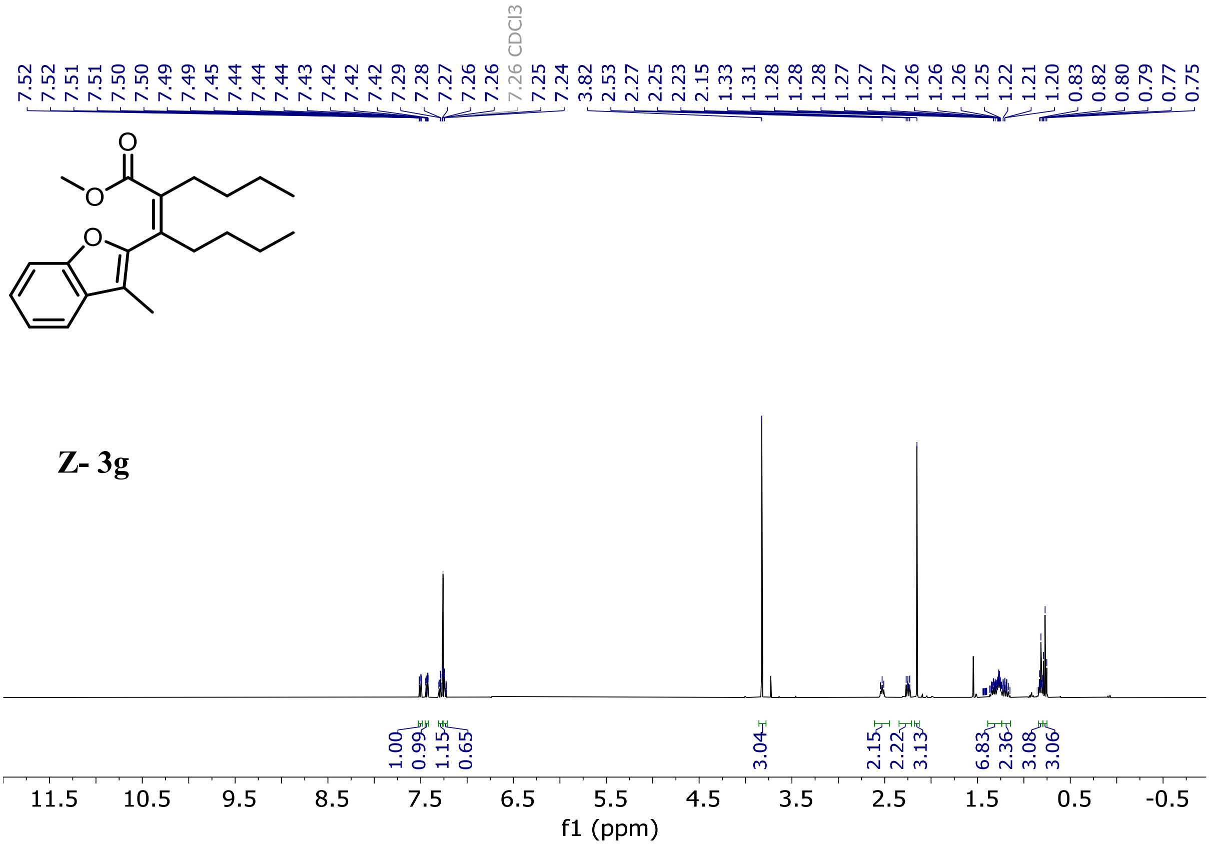
7.52
7.52
7.51
7.51
7.50
7.50
7.49
7.49
7.45
7.44
7.44
7.44
7.44
7.43
7.42
7.42
7.42
7.29
7.28
7.27
7.26
7.26
7.26 CDCl3
7.25
7.24
3.82
2.53
2.27
2.25
2.23
2.15
1.33
1.31
1.28
1.28
1.28
1.27
1.27
1.27
1.26
1.26
1.26
1.25
1.22
1.21
1.20
0.83
0.82
0.80
0.79
0.77
0.75
O
O
O
Z- 3g
1.00
0.99
1.15
0.65
3.04
2.15
2.22
3.13
6.83
2.36
3.08
3.06
11.5
10.5
9.5
8.5
7.5
6.5
5.5
4.5
3.5
2.5
1.5
0.5
-0.5
f1 (ppm)

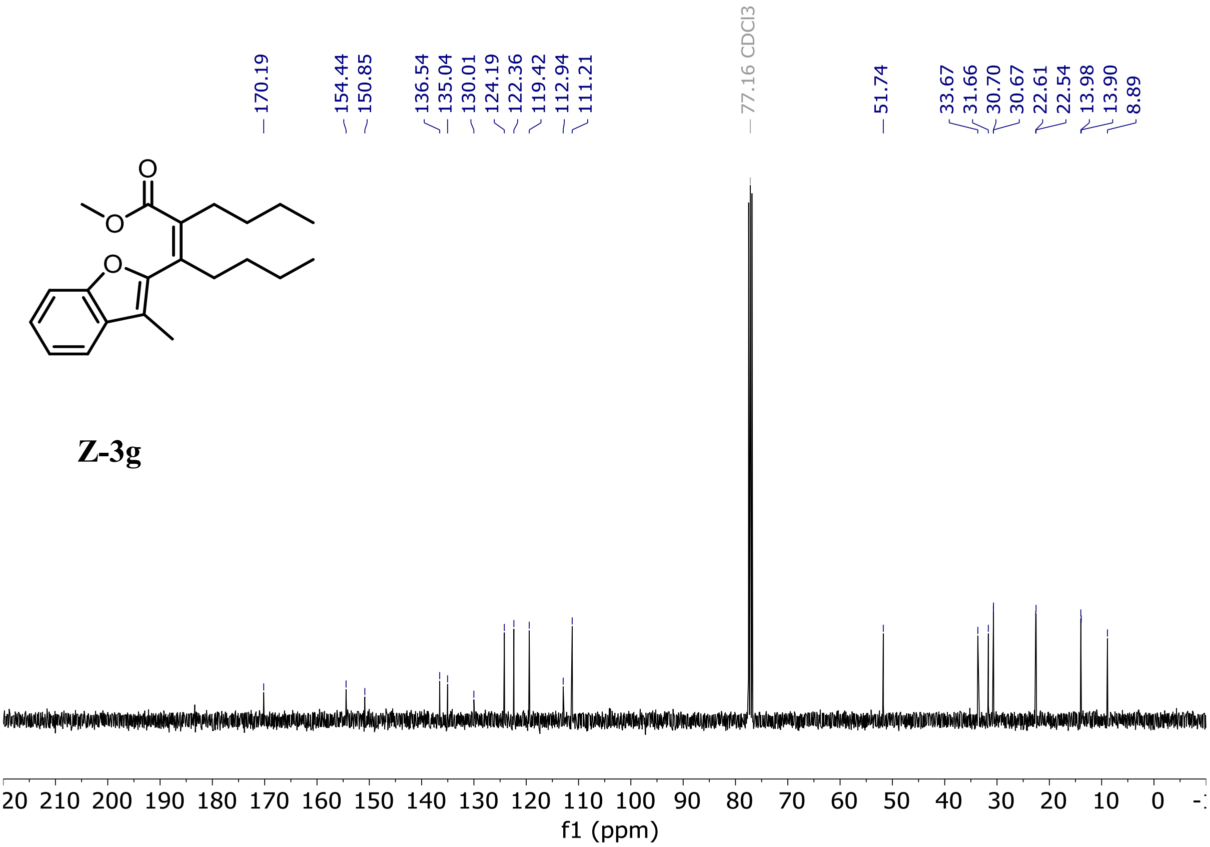
170.19
154.44
150.85
136.54
135.04
130.01
124.19
122.36
119.42
112.94
111.21
77.16 CDCl3
51.74
33.67
31.66
30.70
30.67
22.61
22.54
13.98
13.90
8.89
O
O
O
Z-3g
20 210 200 190 180 170 160 150 140 130 120 110 100 90 80 70 60 50 40 30 20 10 0
f1 (ppm)

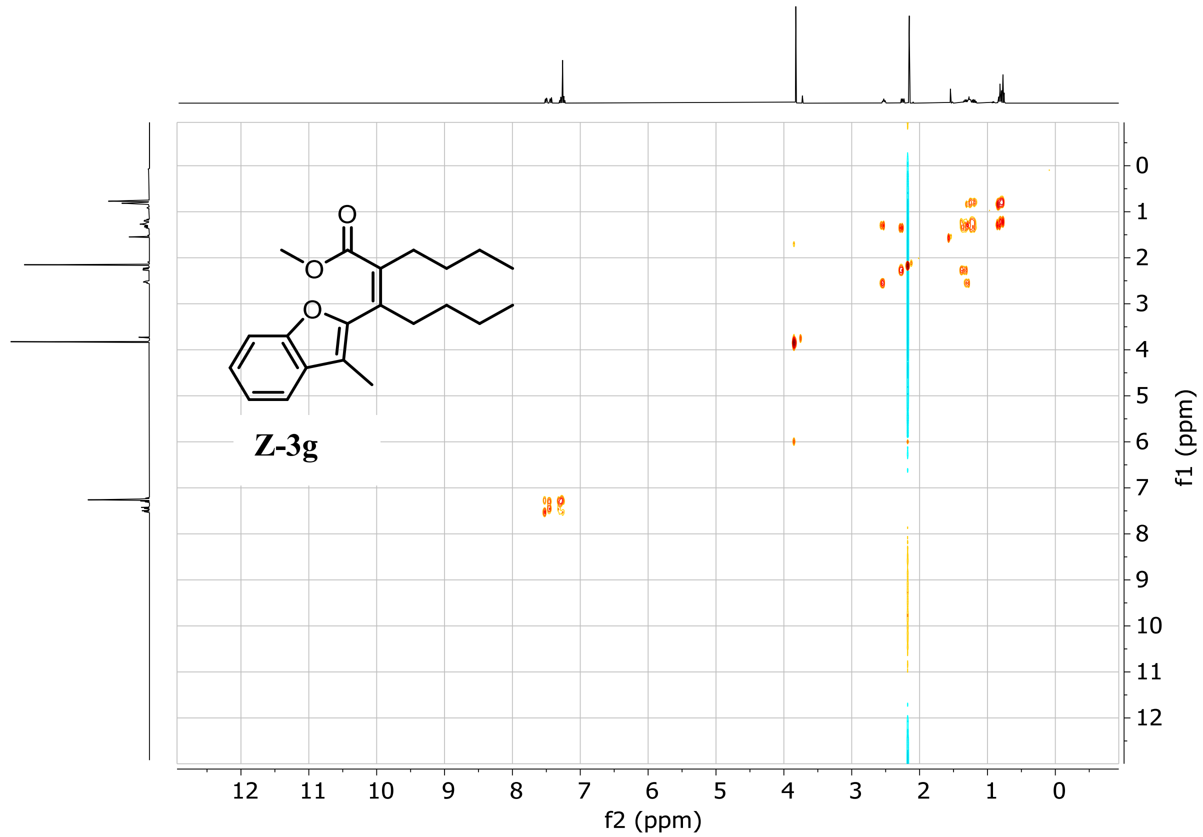

O
O
O
Z-3g
f2 (ppm)
f1 (ppm)

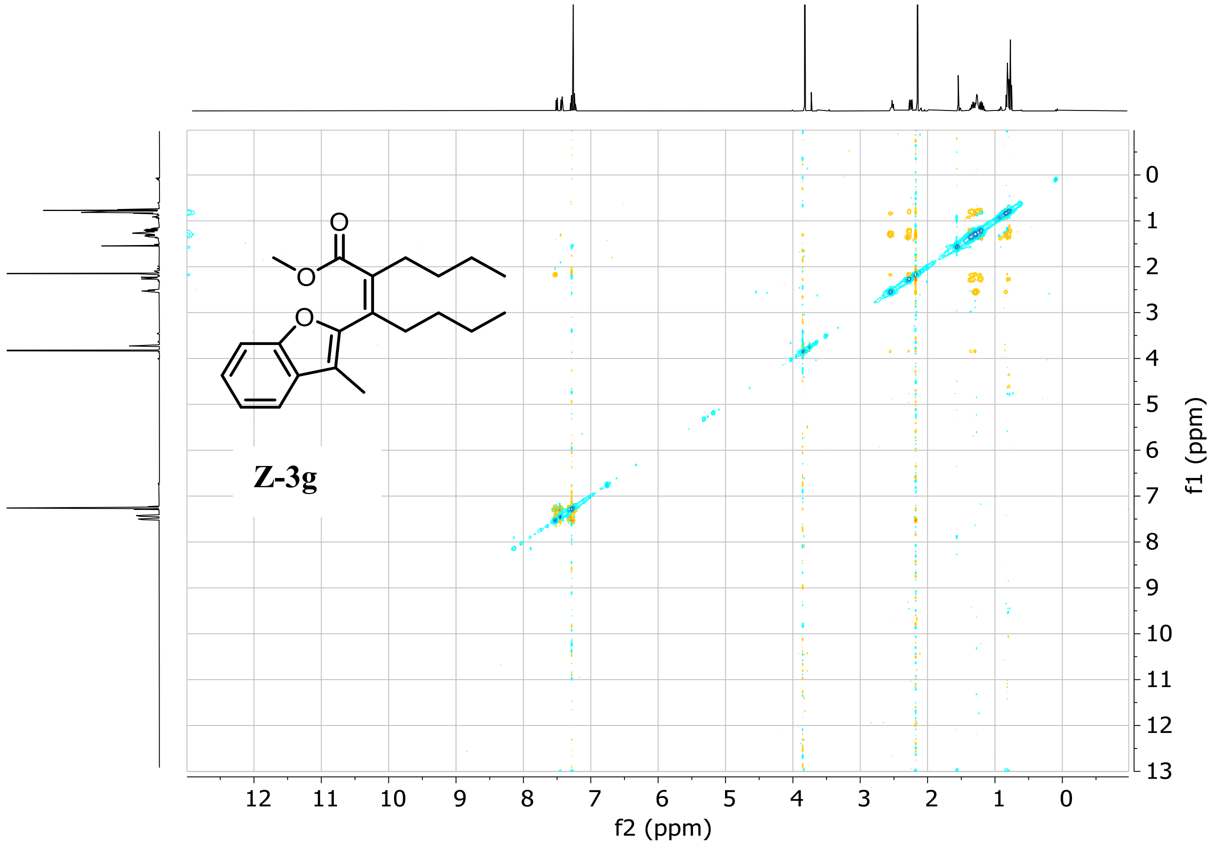

O
O
O
Z-3g
f2 (ppm)
f1 (ppm)

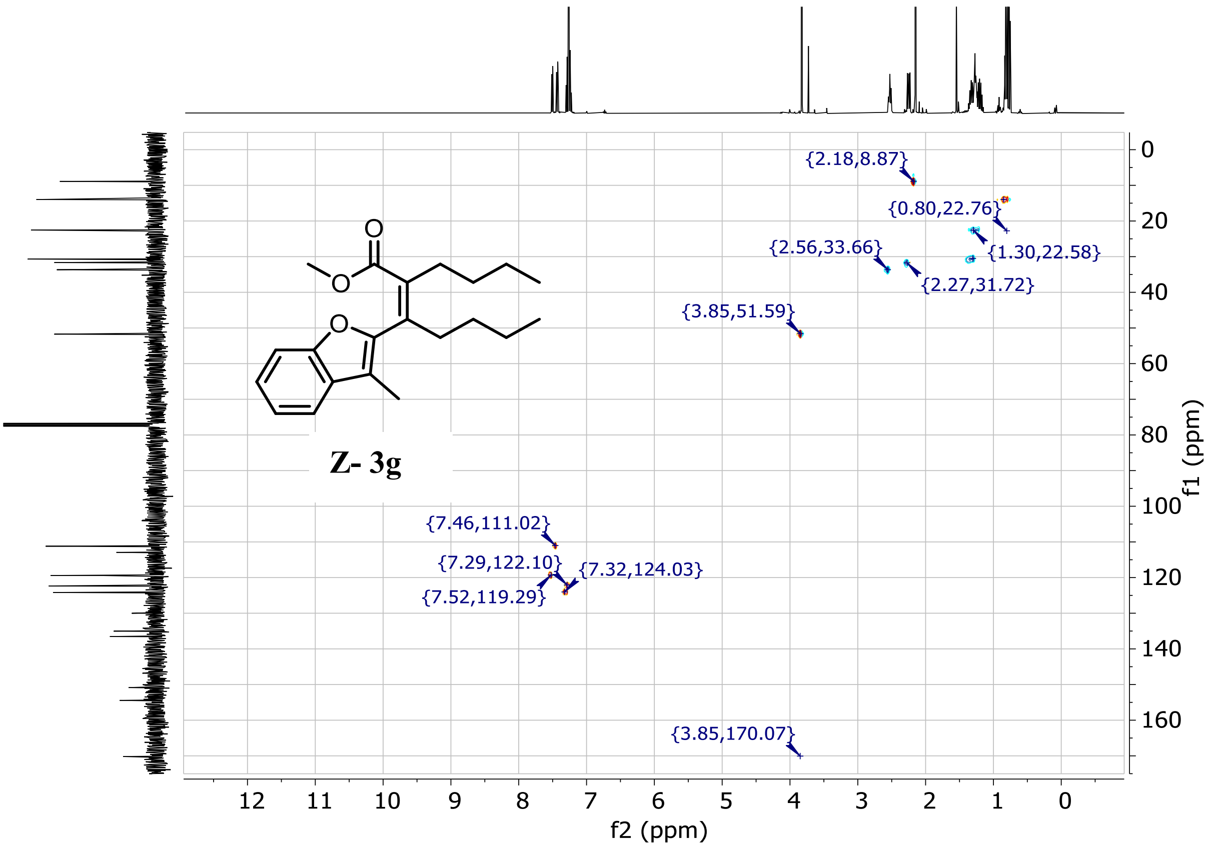
Z- 3g
{2.18,8.87}
{0.80,22.76}
{1.30,22.58}
{2.56,33.66}
{2.27,31.72}
{3.85,51.59}
{7.46,111.02}
{7.29,122.10}
{7.32,124.03}
{7.52,119.29}
{3.85,170.07}
f2 (ppm)
f1 (ppm)

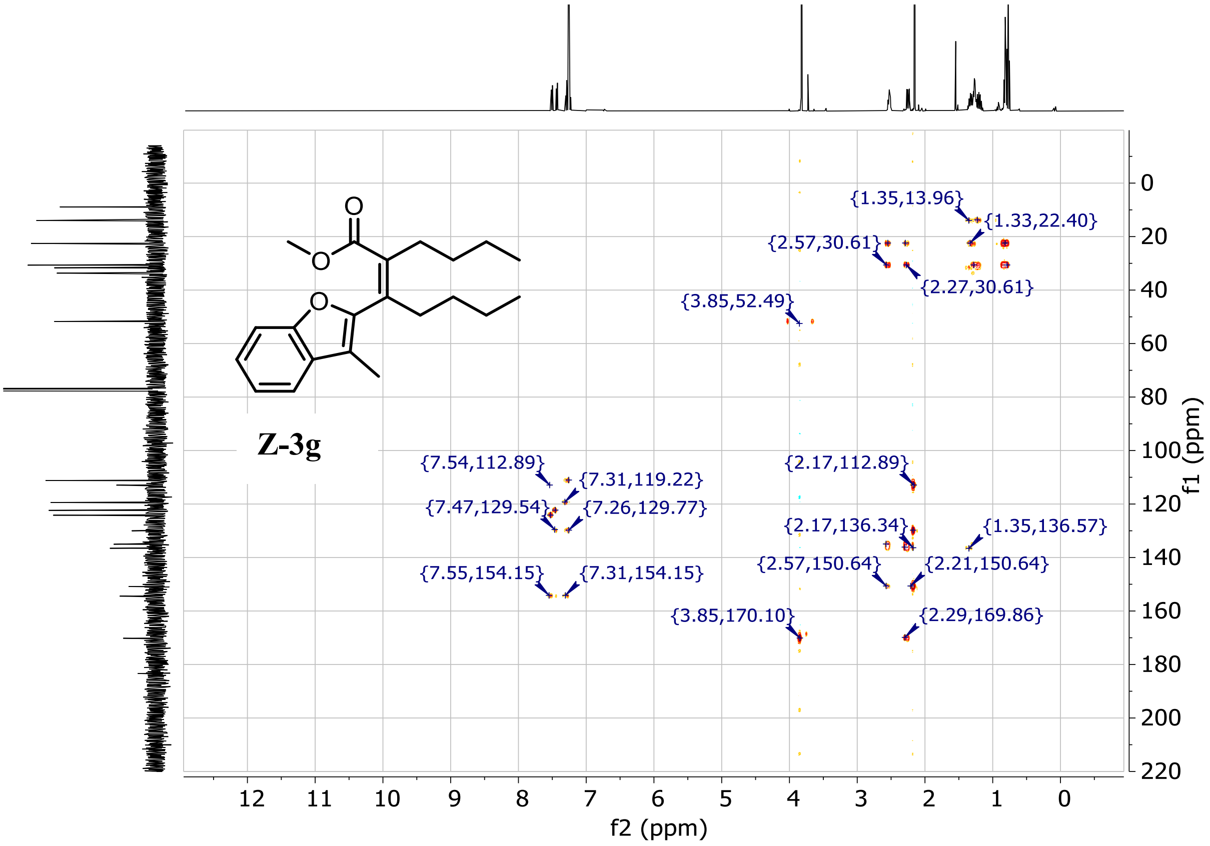
Z-3g
{1.35,13.96}
{1.33,22.40}
{2.57,30.61}
{2.27,30.61}
{3.85,52.49}
{7.54,112.89}
{7.31,119.22}
{7.47,129.54}
{7.26,129.77}
{7.55,154.15}
{7.31,154.15}
{2.17,112.89}
{2.17,136.34}
{1.35,136.57}
{2.57,150.64}
{2.21,150.64}
{3.85,170.10}
{2.29,169.86}
f2 (ppm)
f1 (ppm)

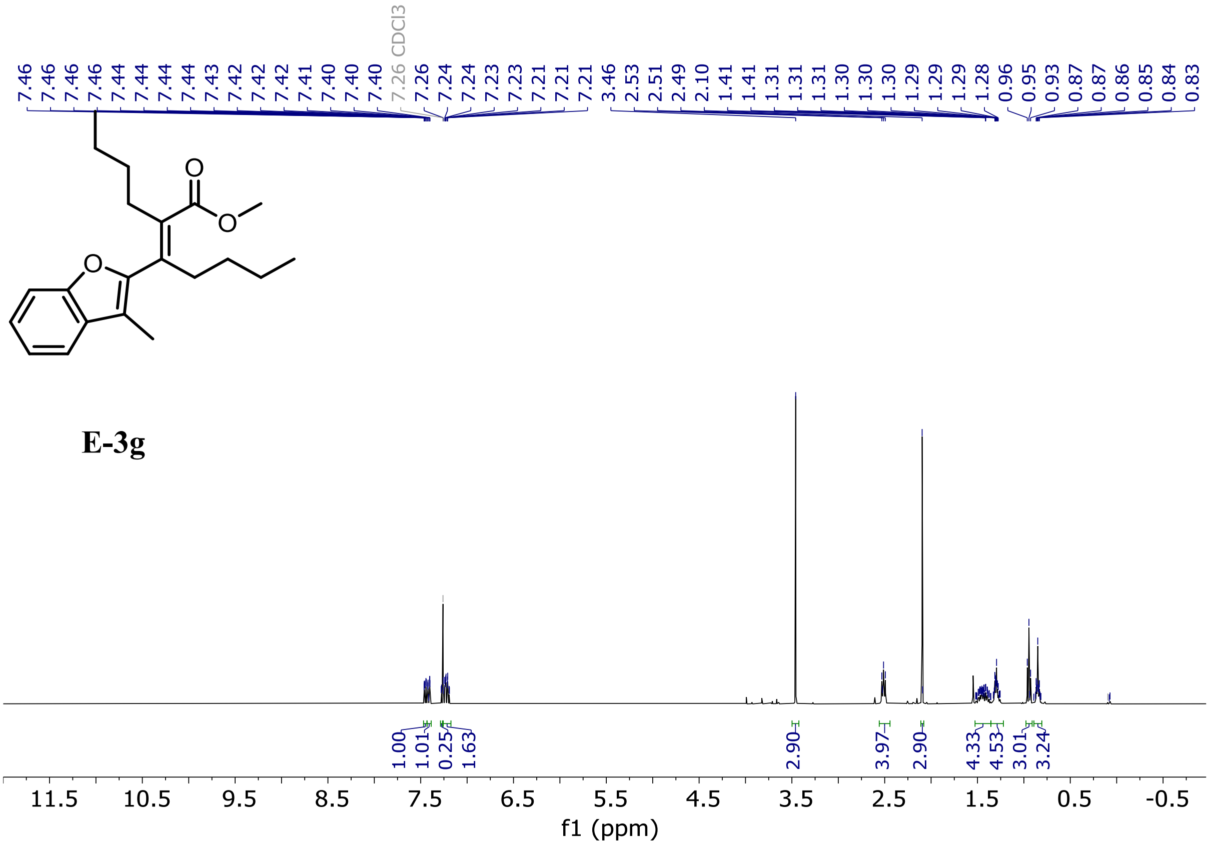
7.46 7.46 7.46 7.46 7.44 7.44 7.44 7.44 7.43 7.42 7.42 7.42 7.41 7.40 7.40 7.40
7.26 CDCl3
7.26 7.24 7.24 7.23 7.23 7.21 7.21 7.21 3.46 2.53 2.51 2.49 2.10 1.41 1.41 1.31 1.31 1.31 1.30 1.30 1.30 1.29 1.29 1.29 1.28 0.96 0.95 0.93 0.87 0.87 0.86 0.85 0.84 0.83
O
O
O
E-3g
1.00 1.01 0.25 1.63 2.90 3.97 2.90 4.33 4.53 3.01 3.24
11.5 10.5 9.5 8.5 7.5 6.5 5.5 4.5 3.5 2.5 1.5 0.5 -0.5
f1 (ppm)

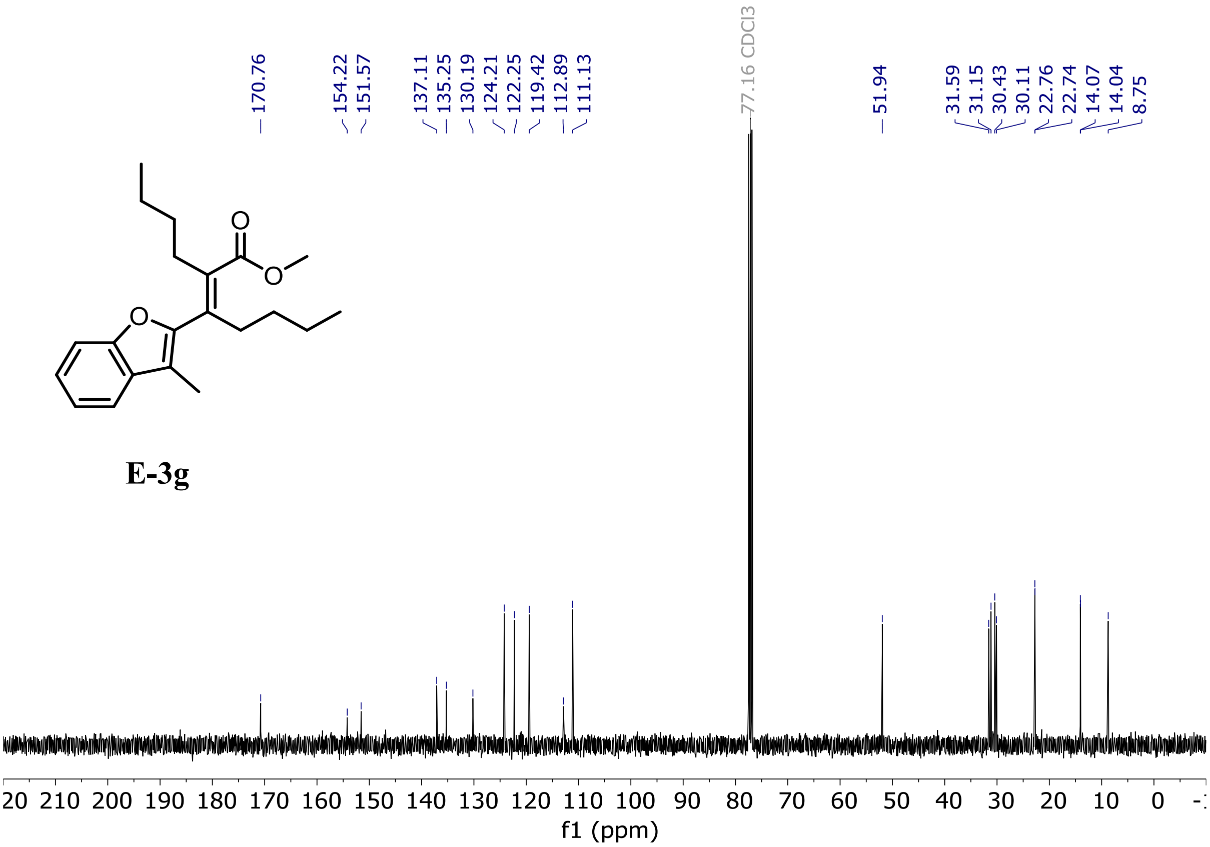
170.76
154.22 151.57
137.11 135.25 130.19 124.21 122.25 119.42 112.89 111.13
77.16 CDCl3
51.94
31.59 31.15 30.43 30.11 22.76 22.74 14.07 14.04 8.75
O
O
O
E-3g
20 210 200 190 180 170 160 150 140 130 120 110 100 90 80 70 60 50 40 30 20 10 0
f1 (ppm)

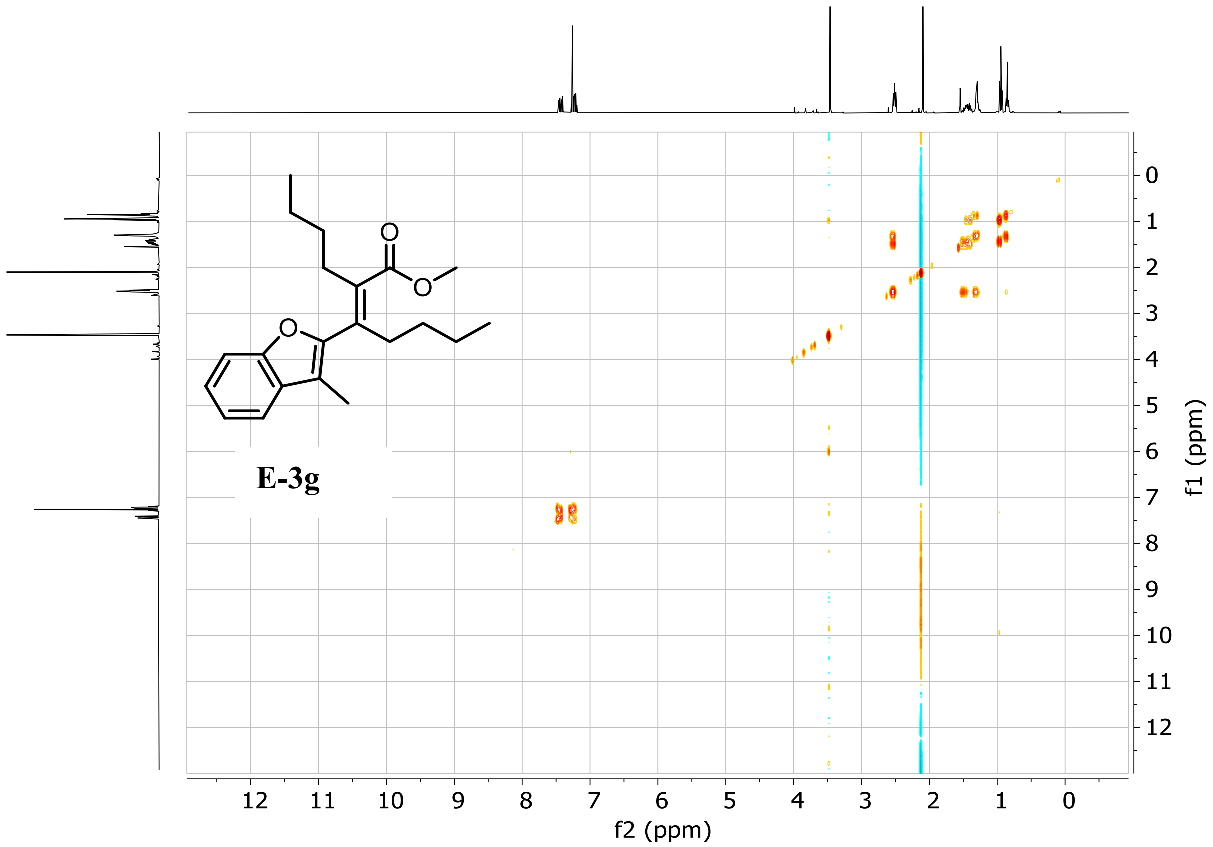
E-3g
f2 (ppm)
f1 (ppm)

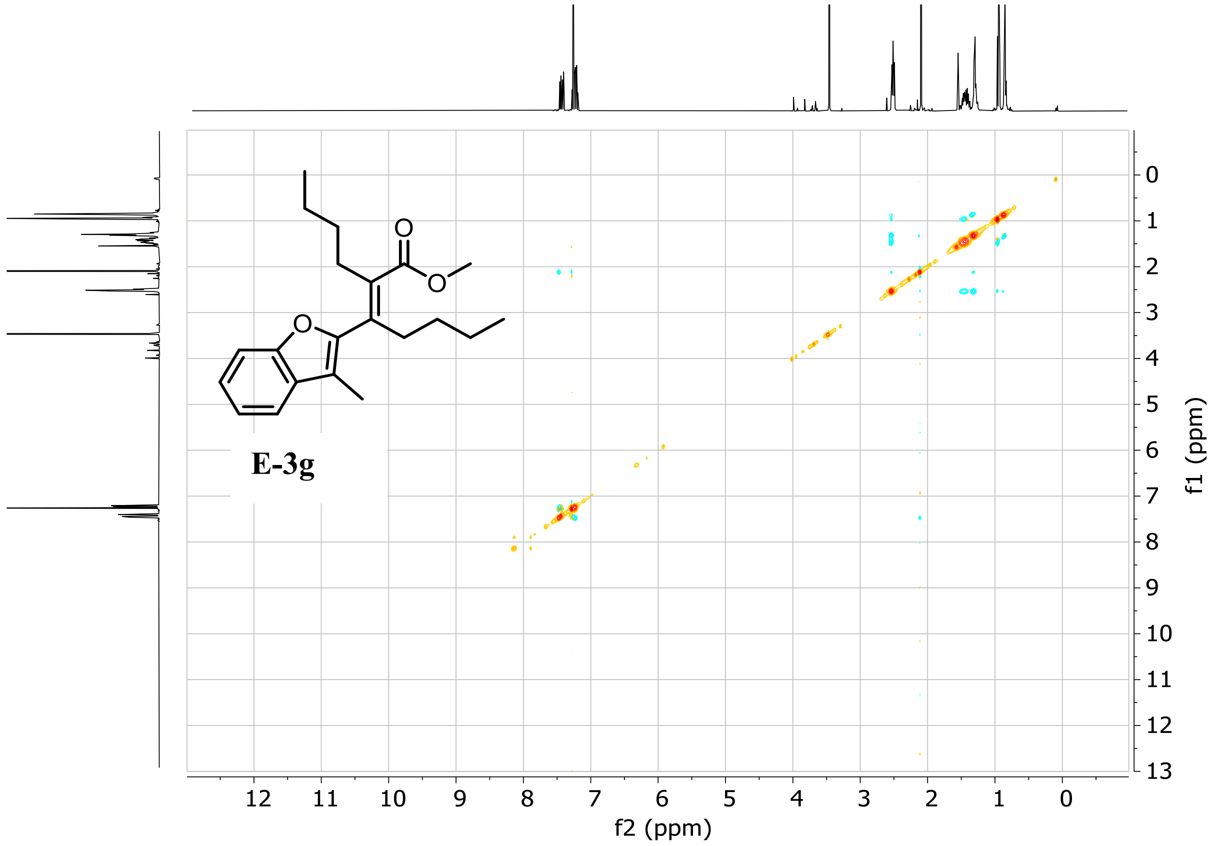
E-3g
f2 (ppm)
f1 (ppm)

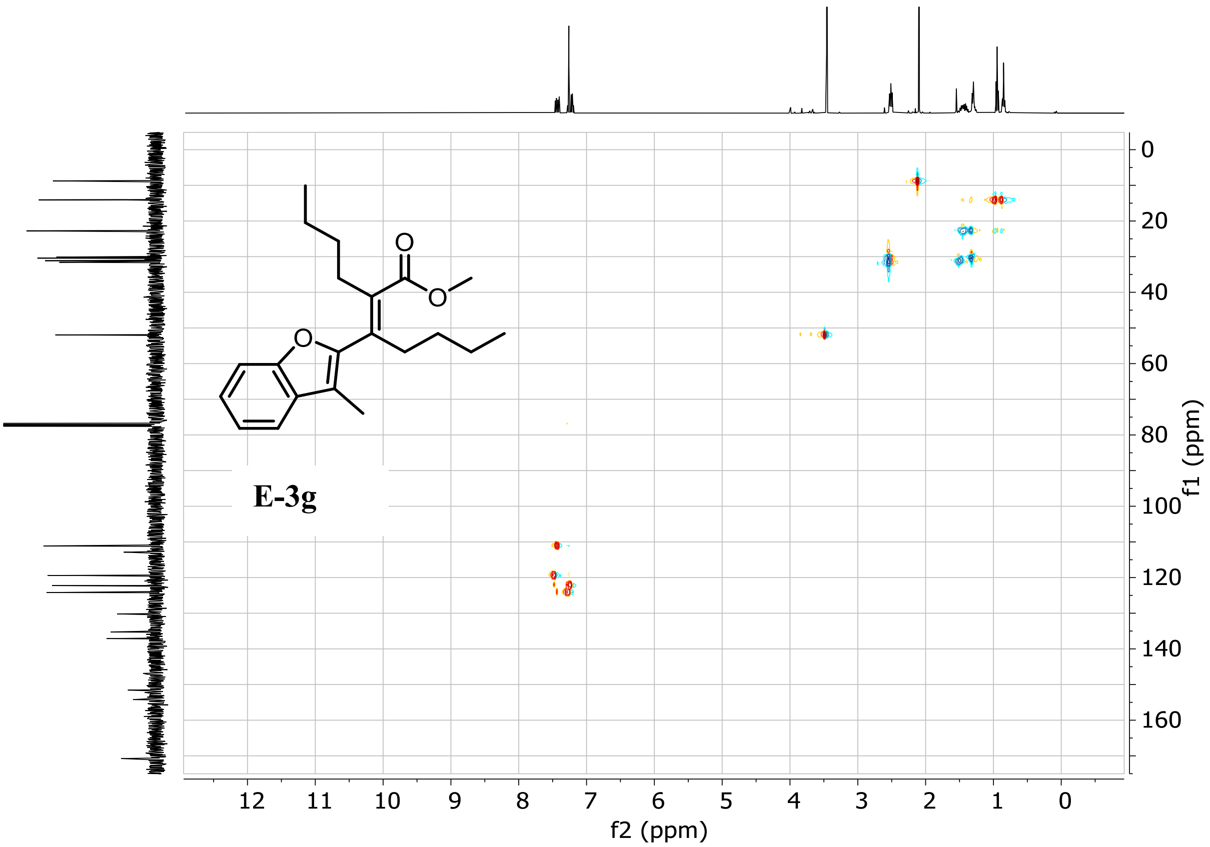

E-3g
f2 (ppm)
f1 (ppm)

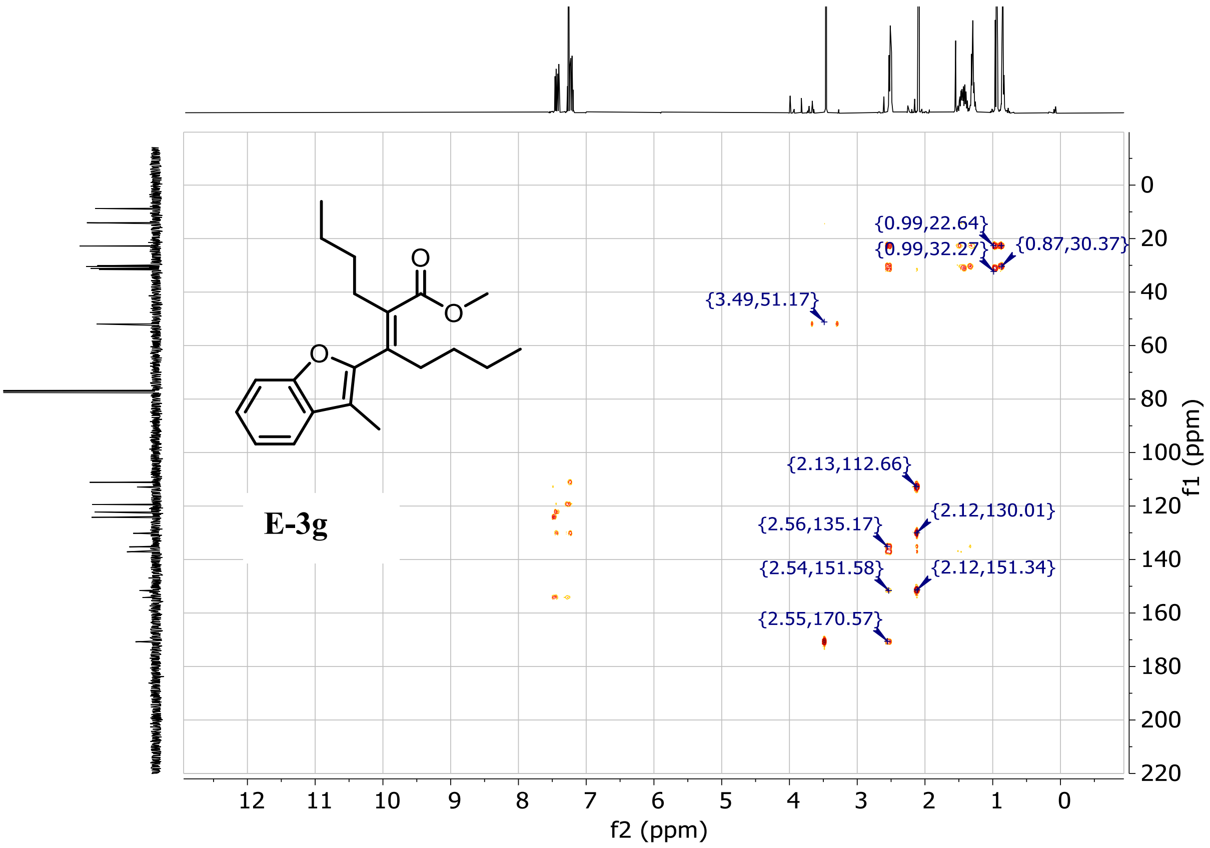

E-3g
{0.99,22.64}
{0.99,32.27}
{0.87,30.37}
{3.49,51.17}
{2.13,112.66}
{2.12,130.01}
{2.56,135.17}
{2.54,151.58}
{2.12,151.34}
{2.55,170.57}
f2 (ppm)
f1 (ppm)

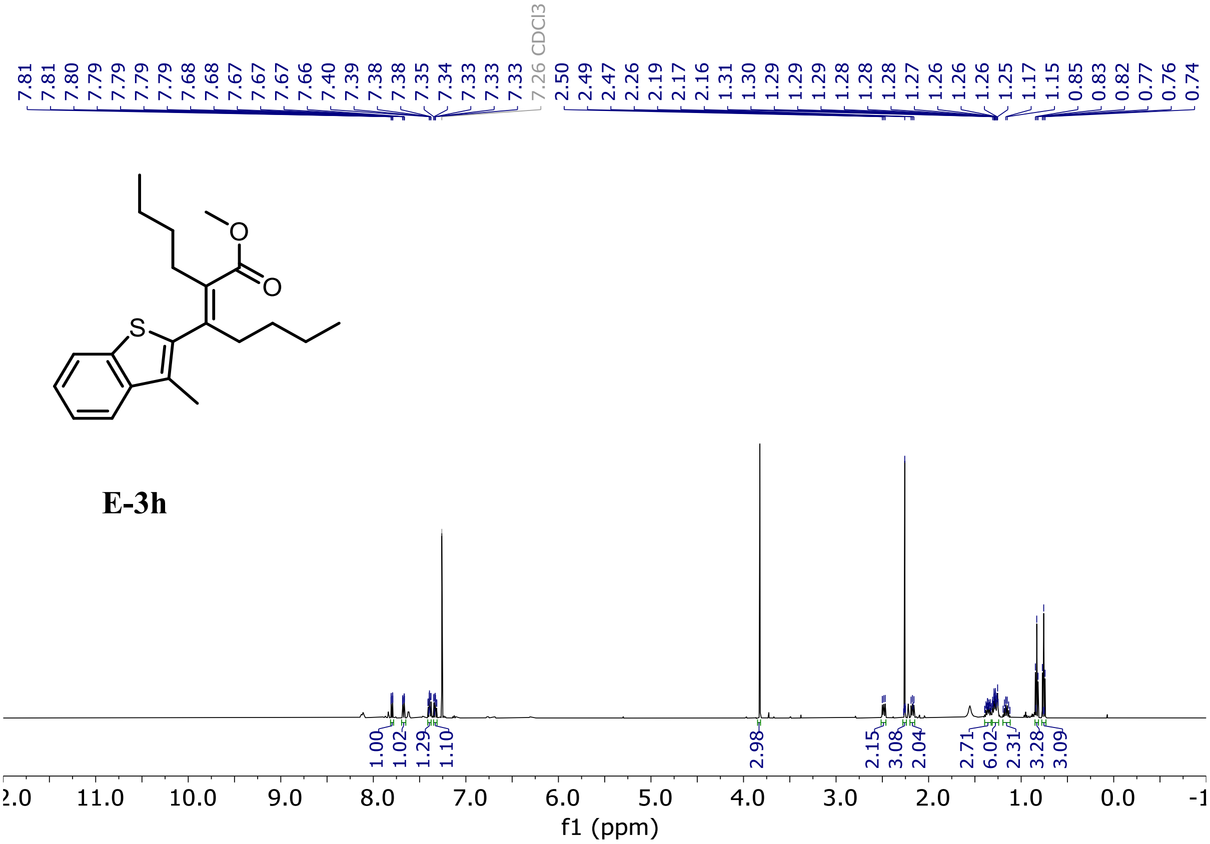
7.81 7.81 7.80 7.79 7.79 7.79 7.68 7.68 7.67 7.67 7.67 7.66 7.40 7.39 7.38 7.38 7.35 7.34 7.33 7.33 7.33 7.26 CDCl3 2.50 2.49 2.47 2.26 2.19 2.17 2.16 1.31 1.30 1.29 1.29 1.29 1.28 1.28 1.28 1.27 1.26 1.26 1.26 1.25 1.17 1.15 0.85 0.83 0.82 0.77 0.76 0.74
E-3h
1.00 1.02 1.29 1.10 2.98 2.15 3.08 2.04 2.71 6.02 2.31 3.28 3.09
f1 (ppm)

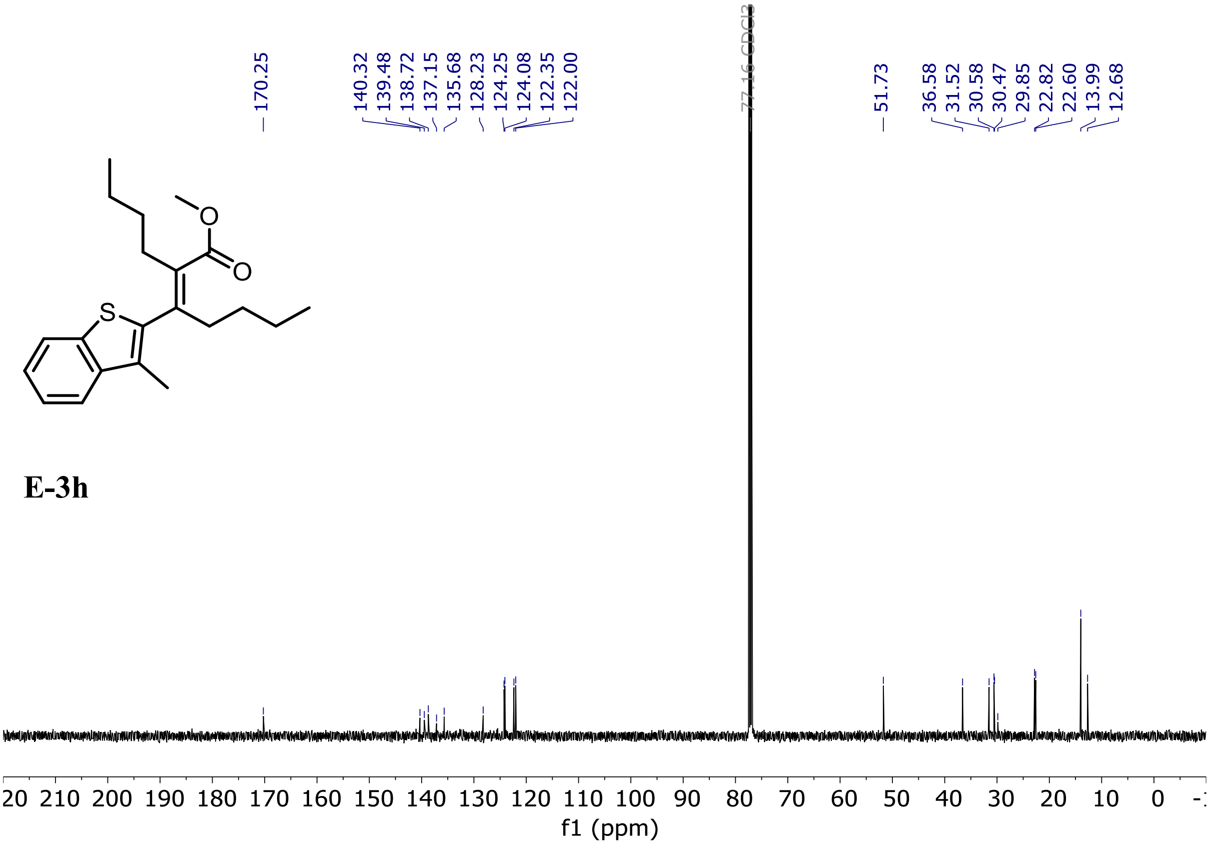
170.25
140.32 139.48 138.72 137.15 135.68 128.23 124.25 124.08 122.35 122.00
77.16 CDCl3
51.73
36.58 31.52 30.58 30.47 29.85 22.82 22.60 13.99 12.68
E-3h
f1 (ppm)

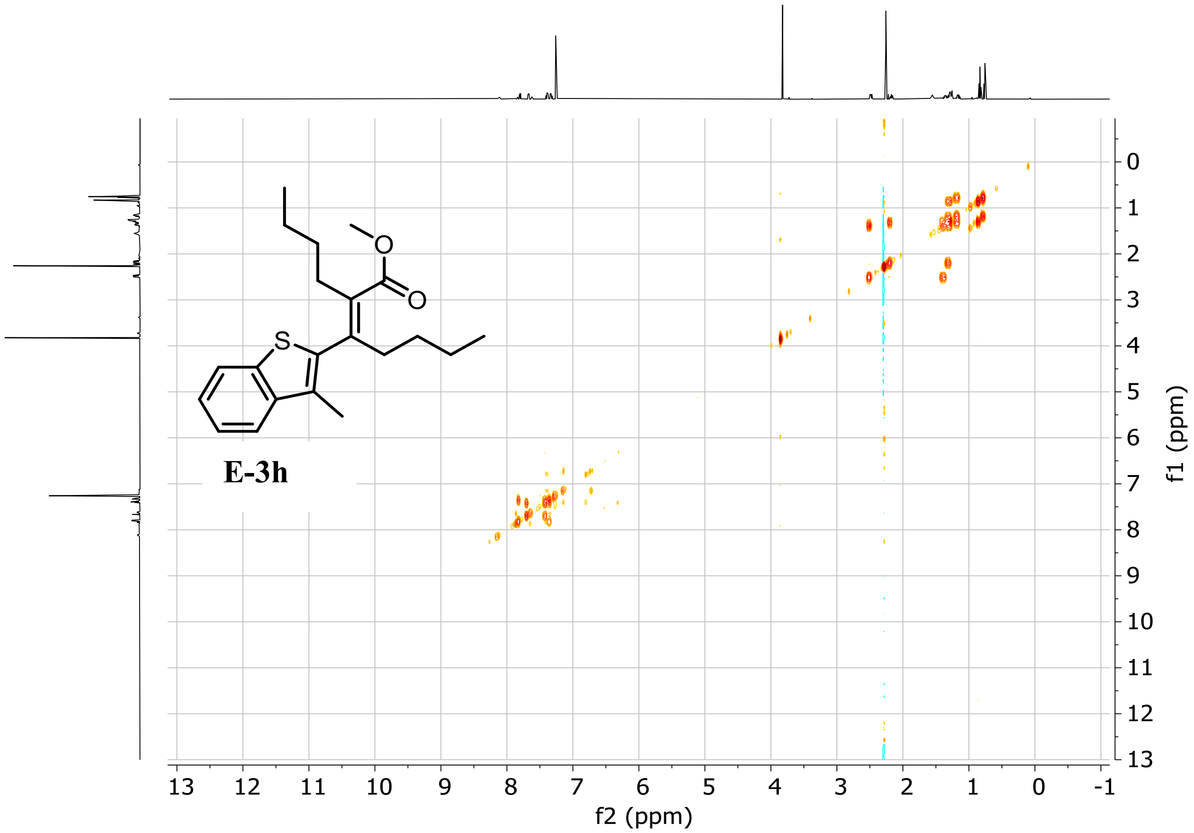
E-3h
f2 (ppm)
f1 (ppm)

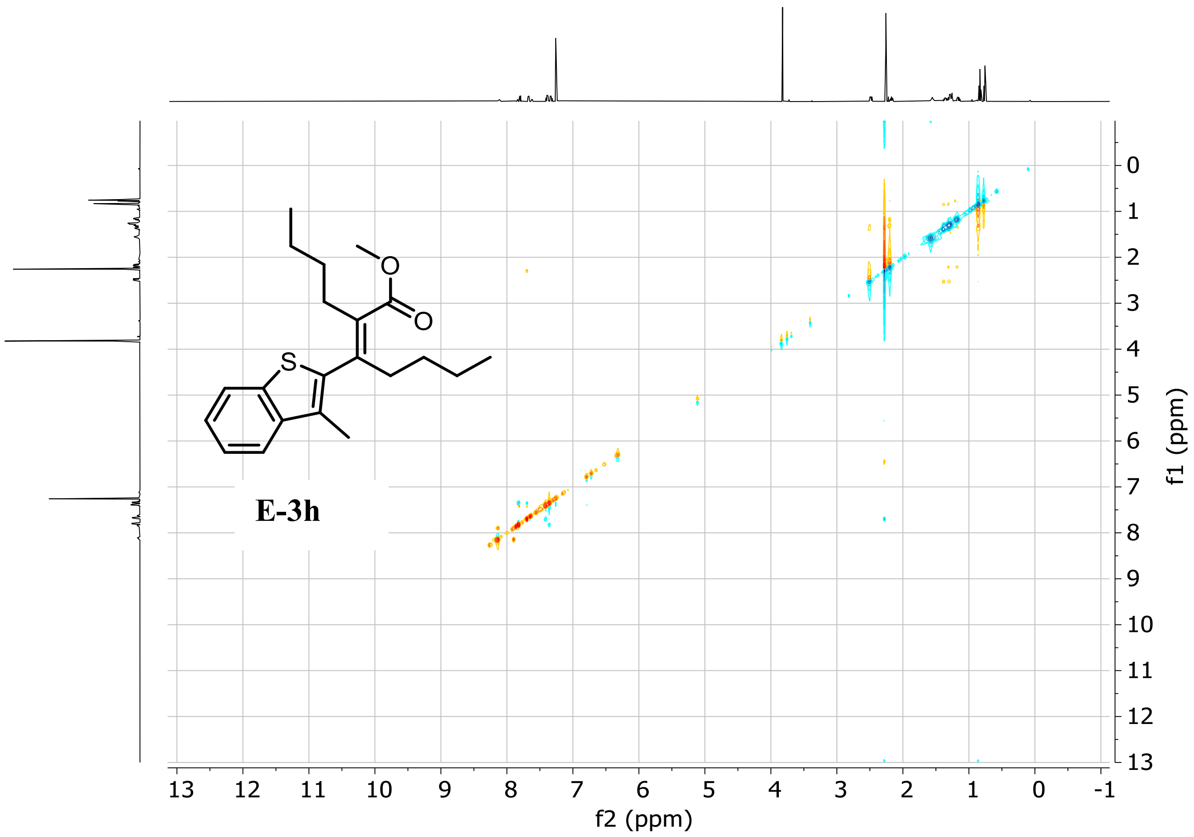
E-3h
f2 (ppm)
f1 (ppm)

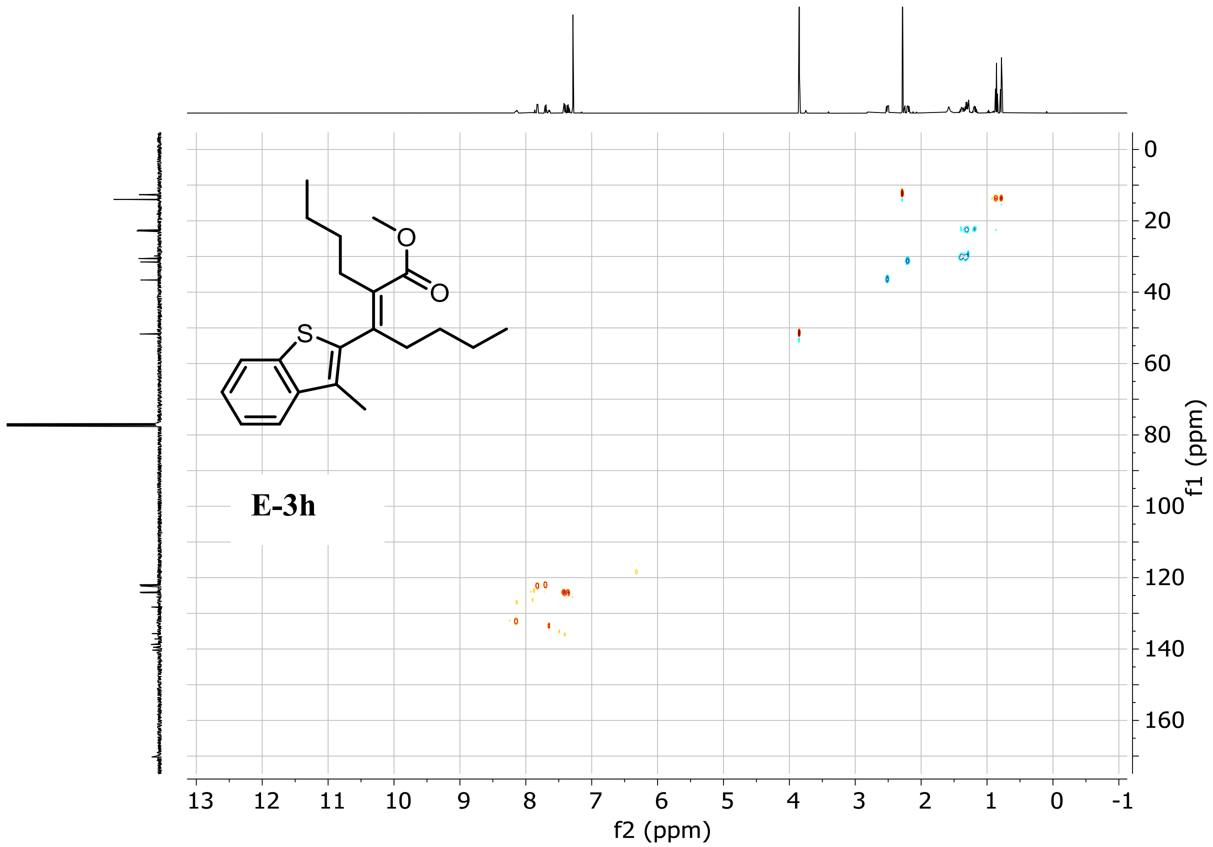

E-3h
f2 (ppm)
f1 (ppm)

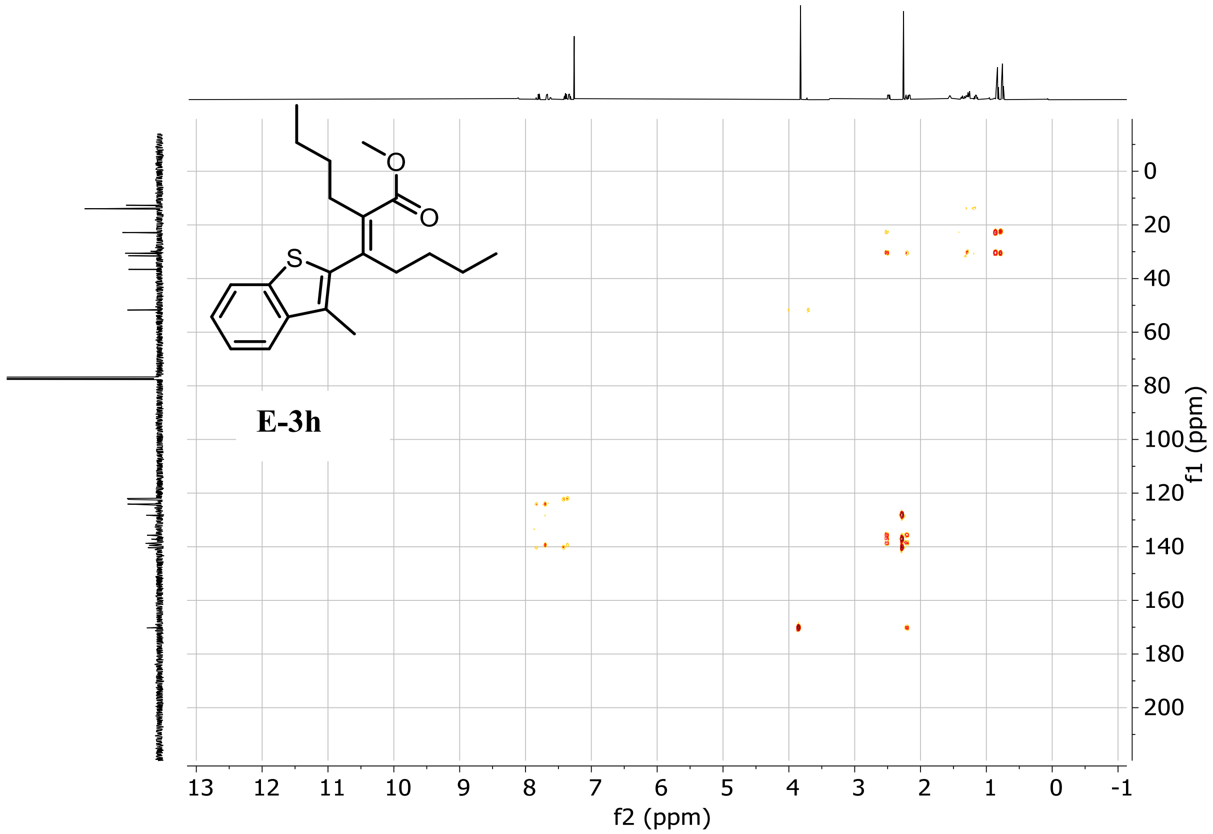

E-3h
f2 (ppm)
f1 (ppm)

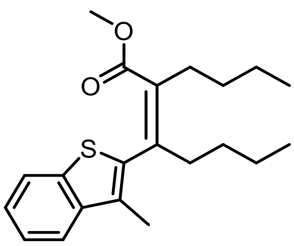

Z-3h

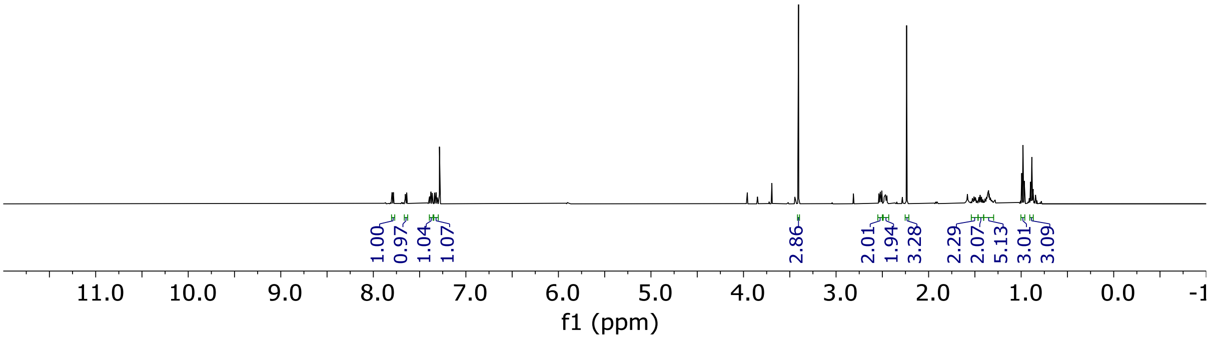
1.00
0.97
1.04
1.07
2.86
2.01
1.94
3.28
2.29
2.07
5.13
3.01
3.09
11.0 10.0 9.0 8.0 7.0 6.0 5.0 4.0 3.0 2.0 1.0 0.0 -1
f1 (ppm)

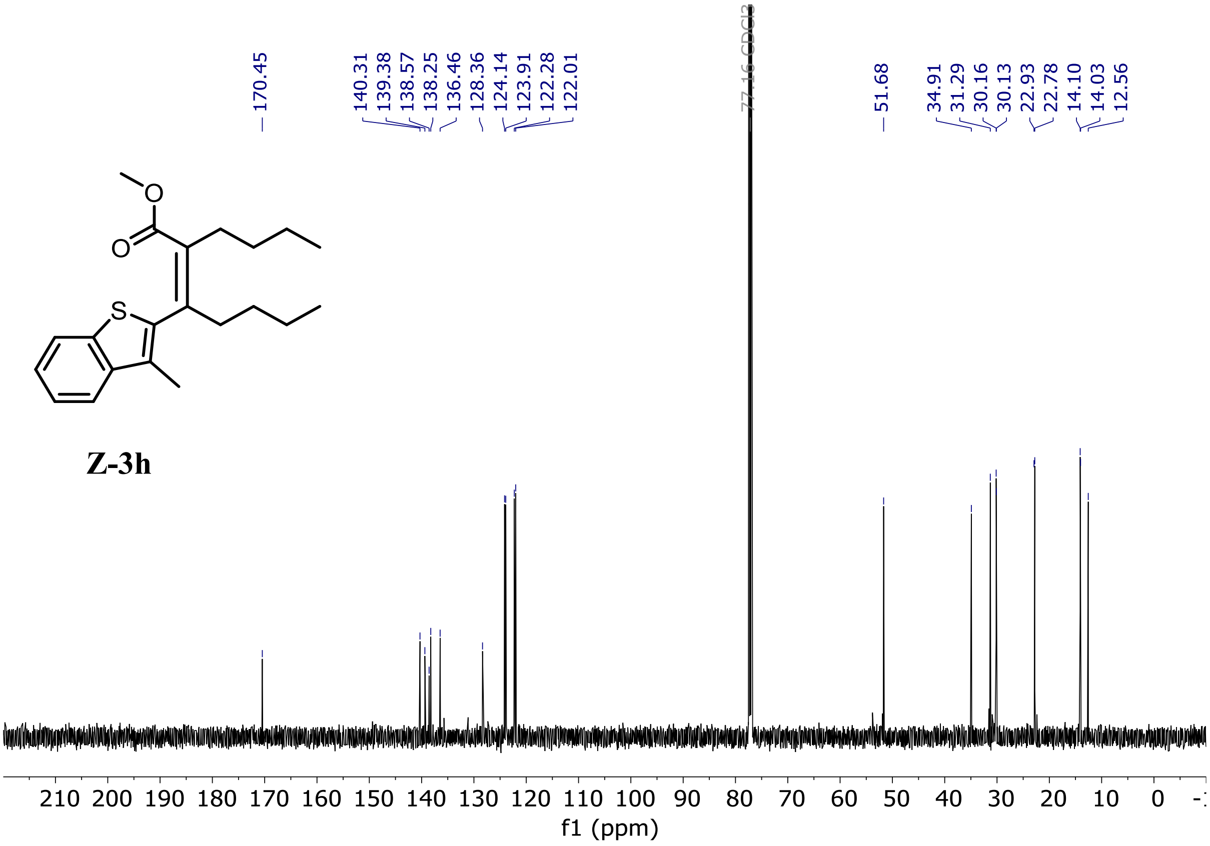
170.45
140.31
139.38
138.57
138.25
136.46
128.36
124.14
123.91
122.28
122.01
77.16 CDCl3
51.68
34.91
31.29
30.16
30.13
22.93
22.78
14.10
14.03
12.56
Z-3h
210 200 190 180 170 160 150 140 130 120 110 100 90 80 70 60 50 40 30 20 10 0
f1 (ppm)

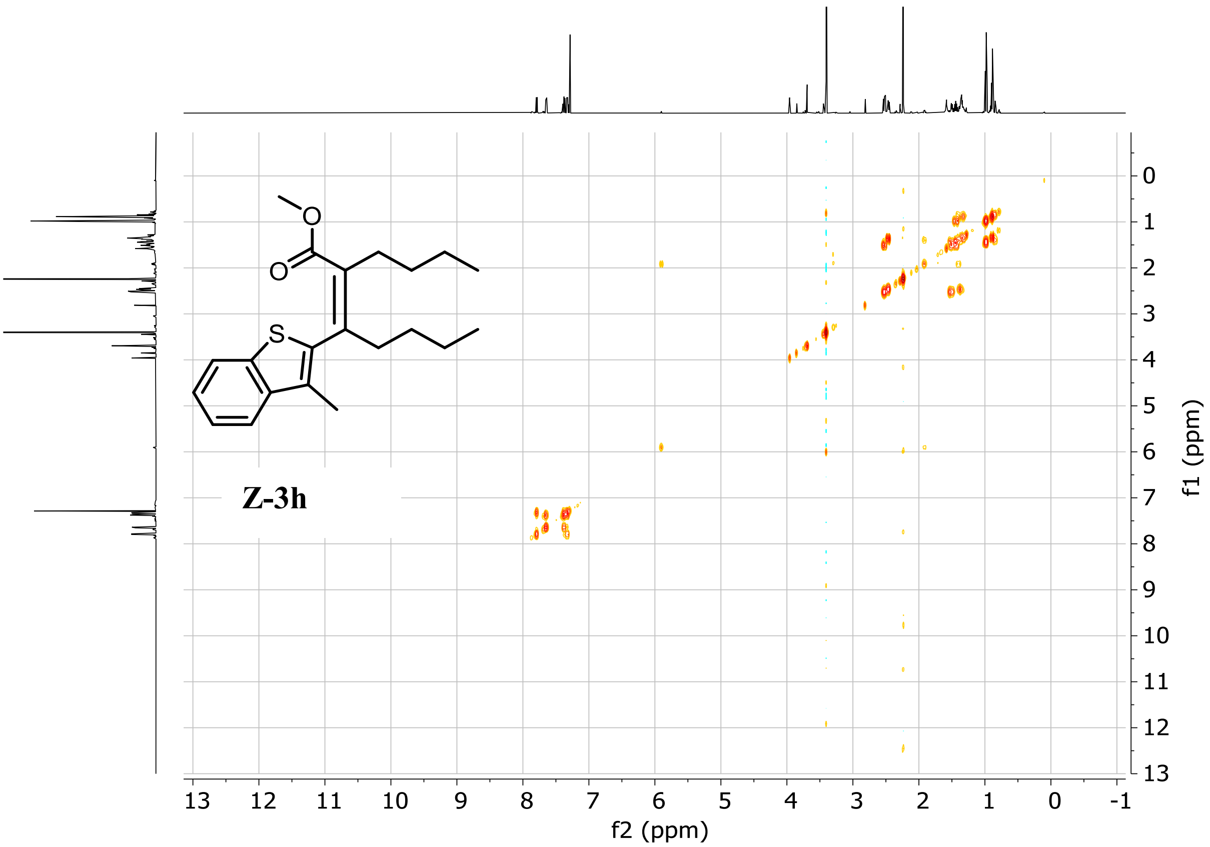

Z-3h
f2 (ppm)
f1 (ppm)

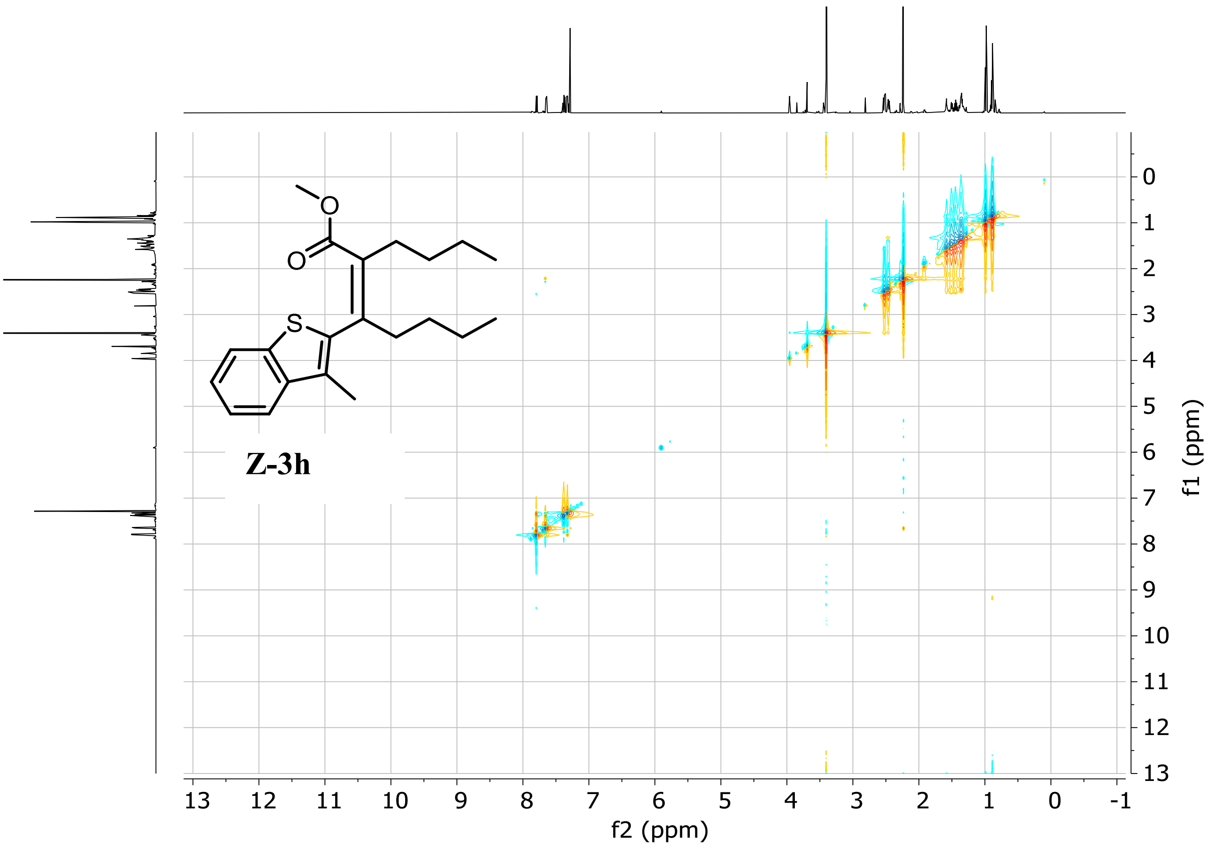

Z-3h
f2 (ppm)
f1 (ppm)

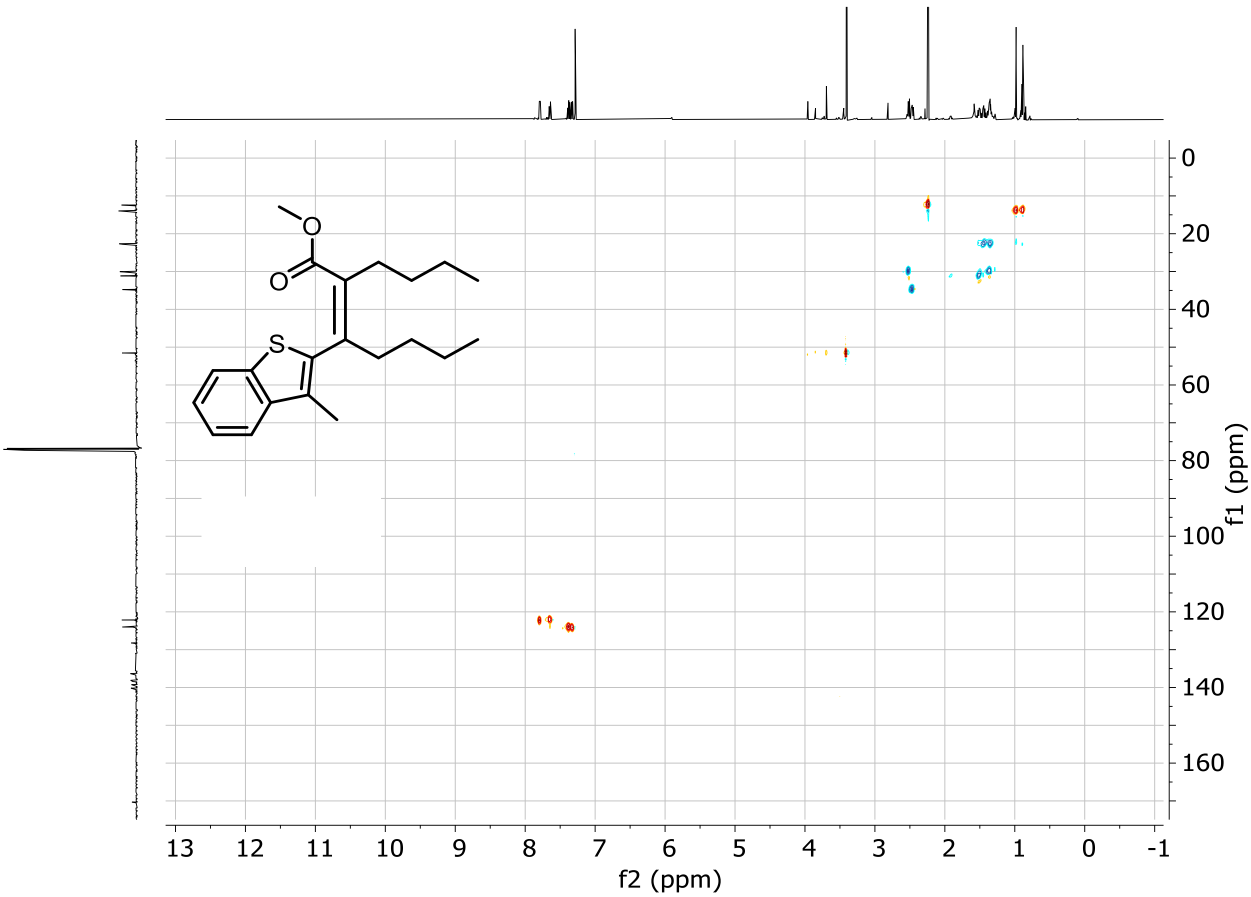
O
O
S
f1 (ppm)
f2 (ppm)

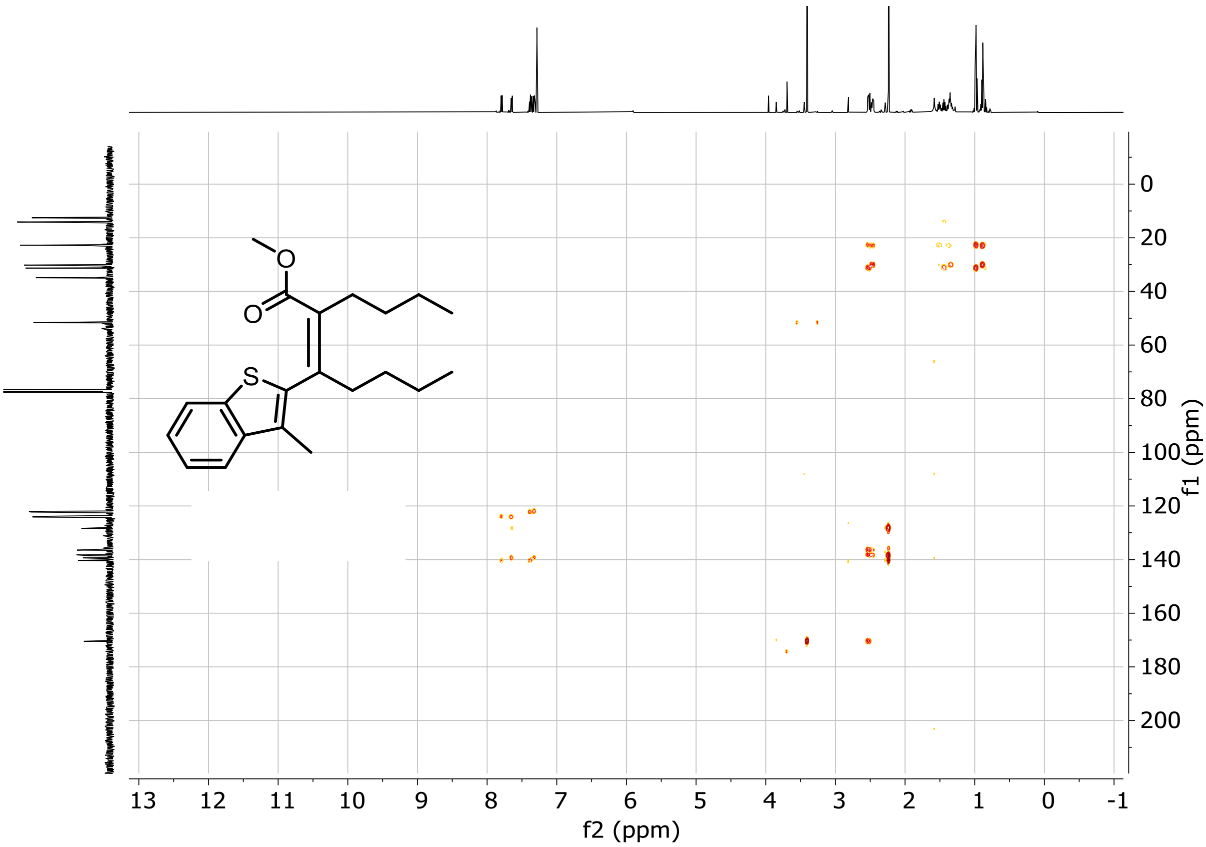
O
O
S
f1 (ppm)
f2 (ppm)

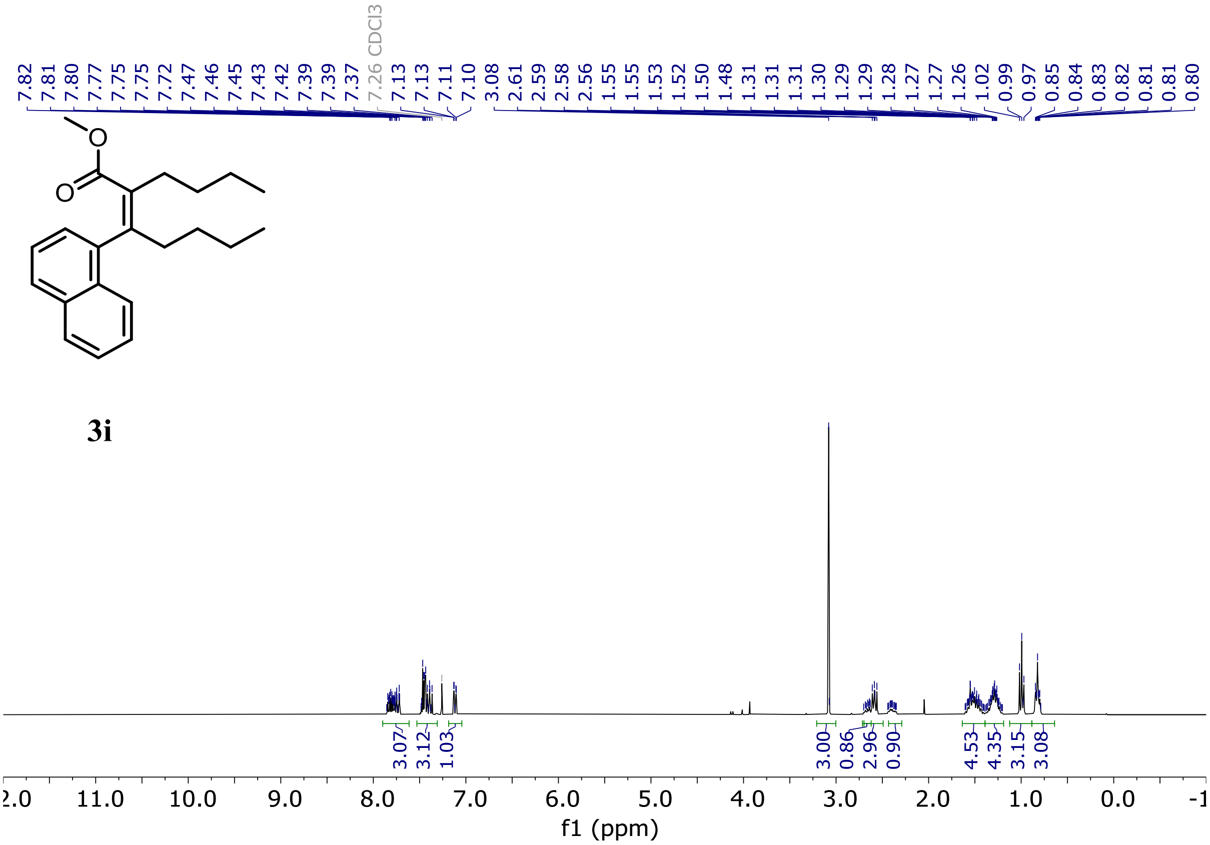

3i

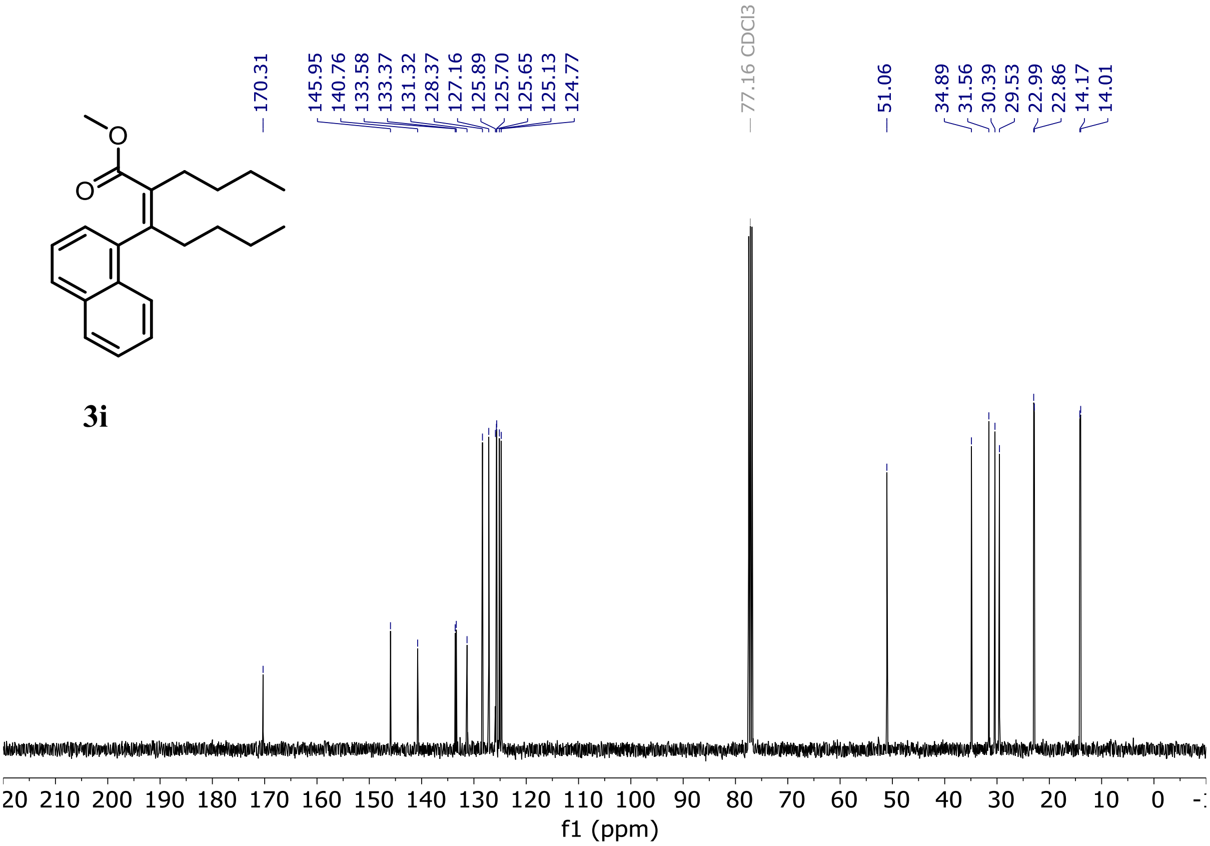

3i

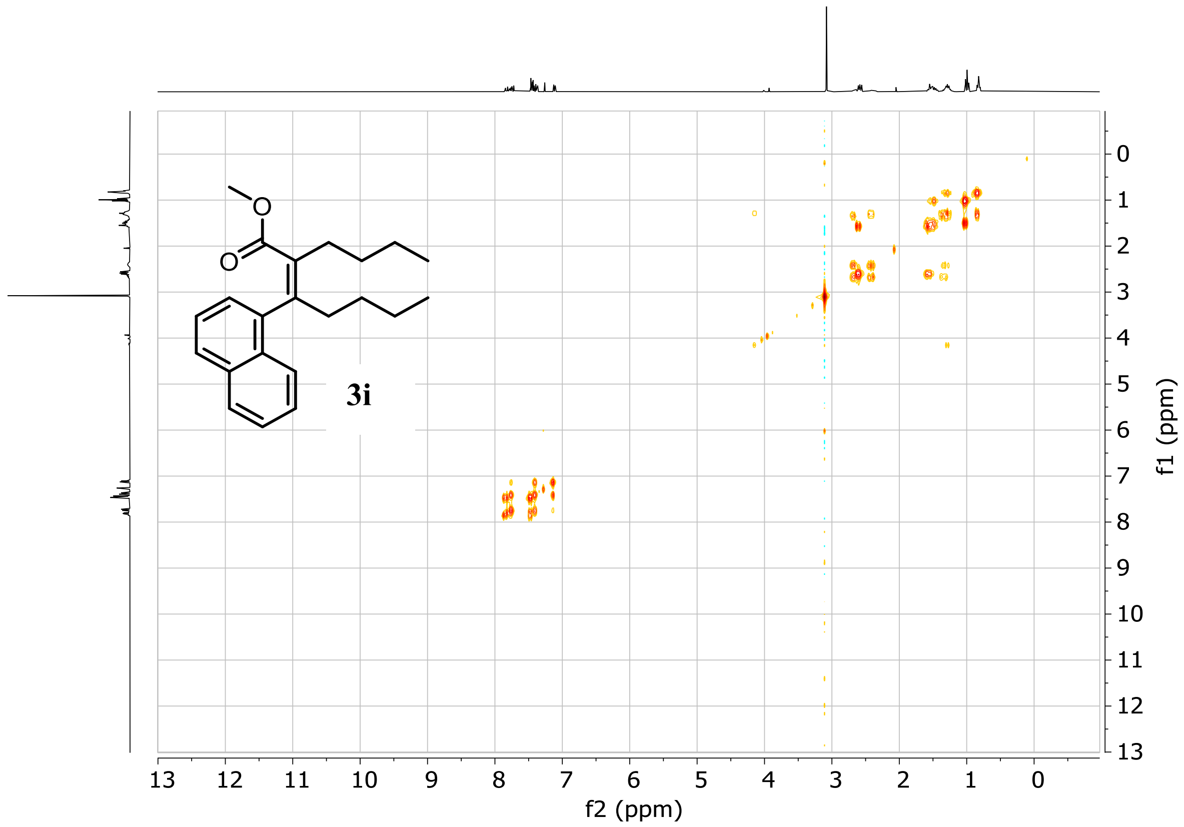
O
O
3i
f2 (ppm)
f1 (ppm)

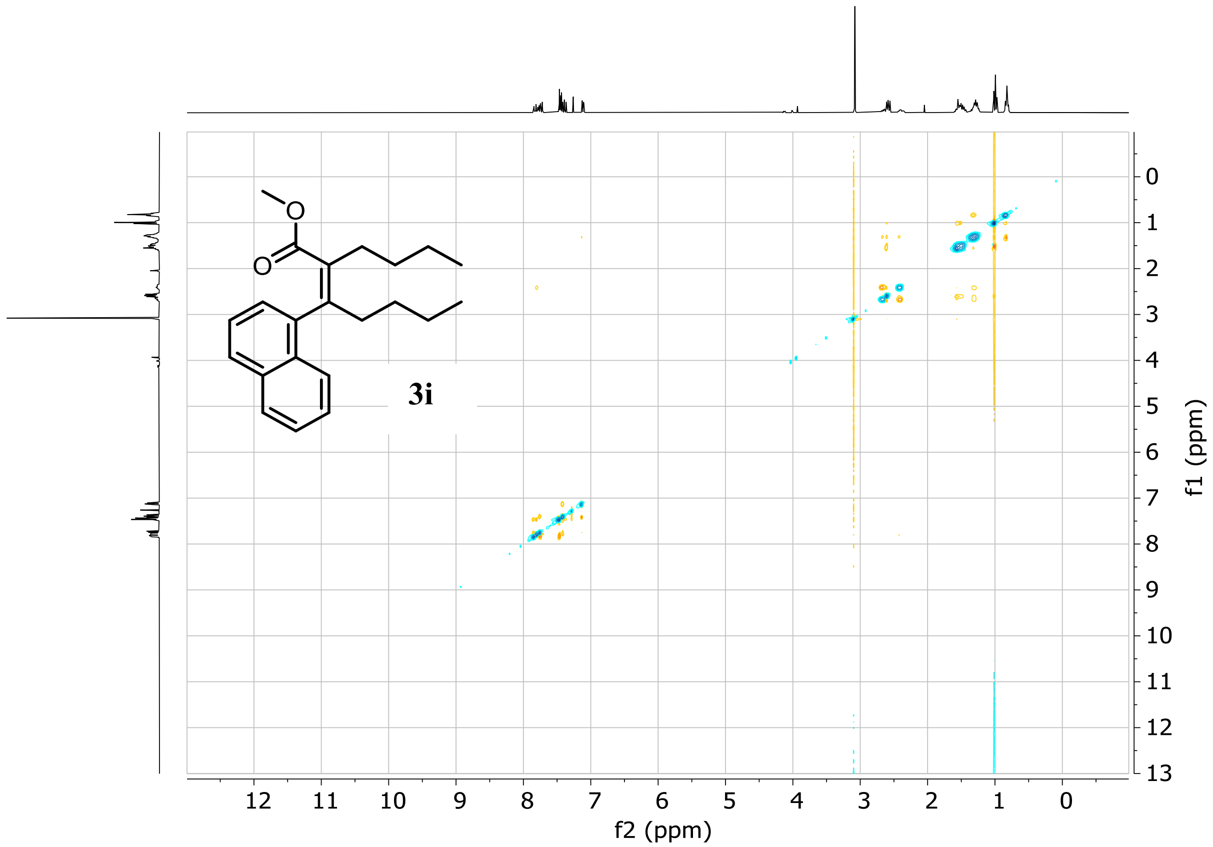
O
O
3i
f2 (ppm)
f1 (ppm)

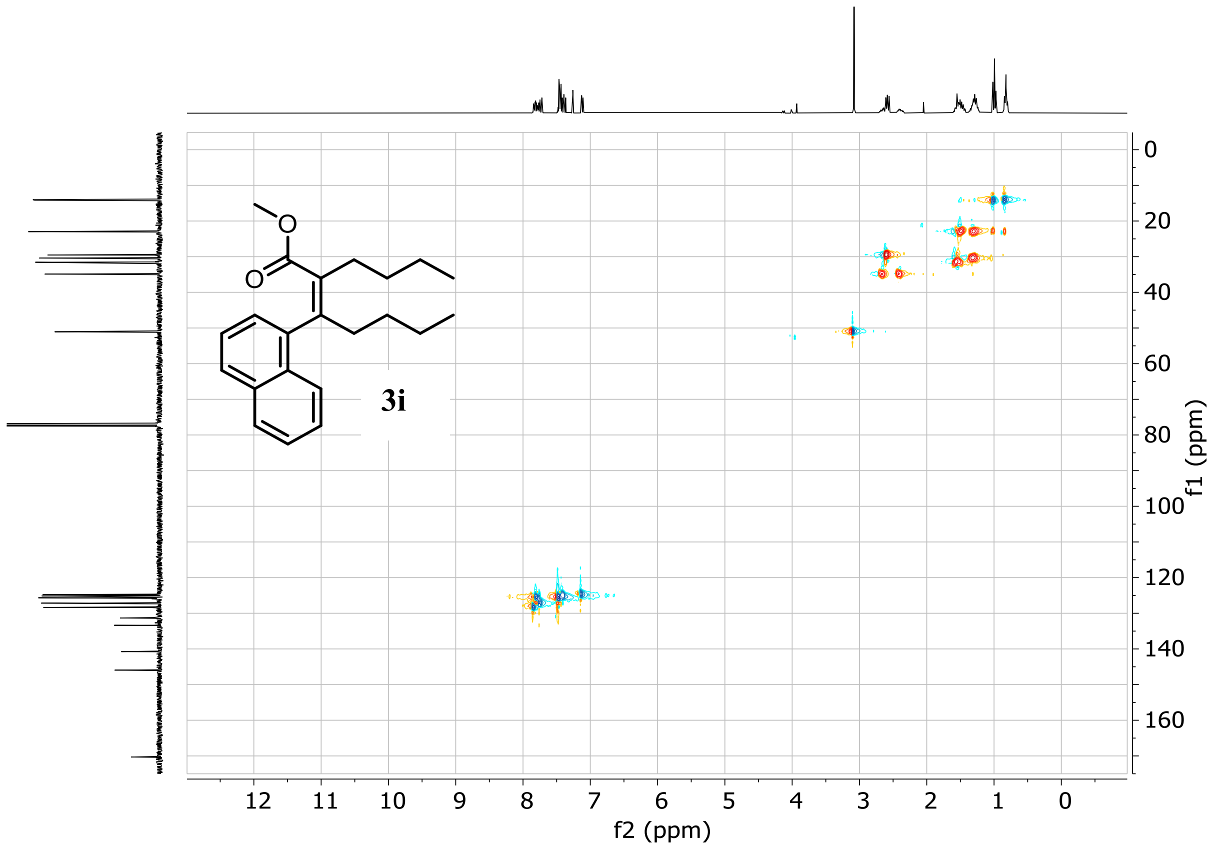
3i
12 11 10 9 8 7 6 5 4 3 2 1 0
f2 (ppm)
0 20 40 60 80 100 120 140 160
f1 (ppm)

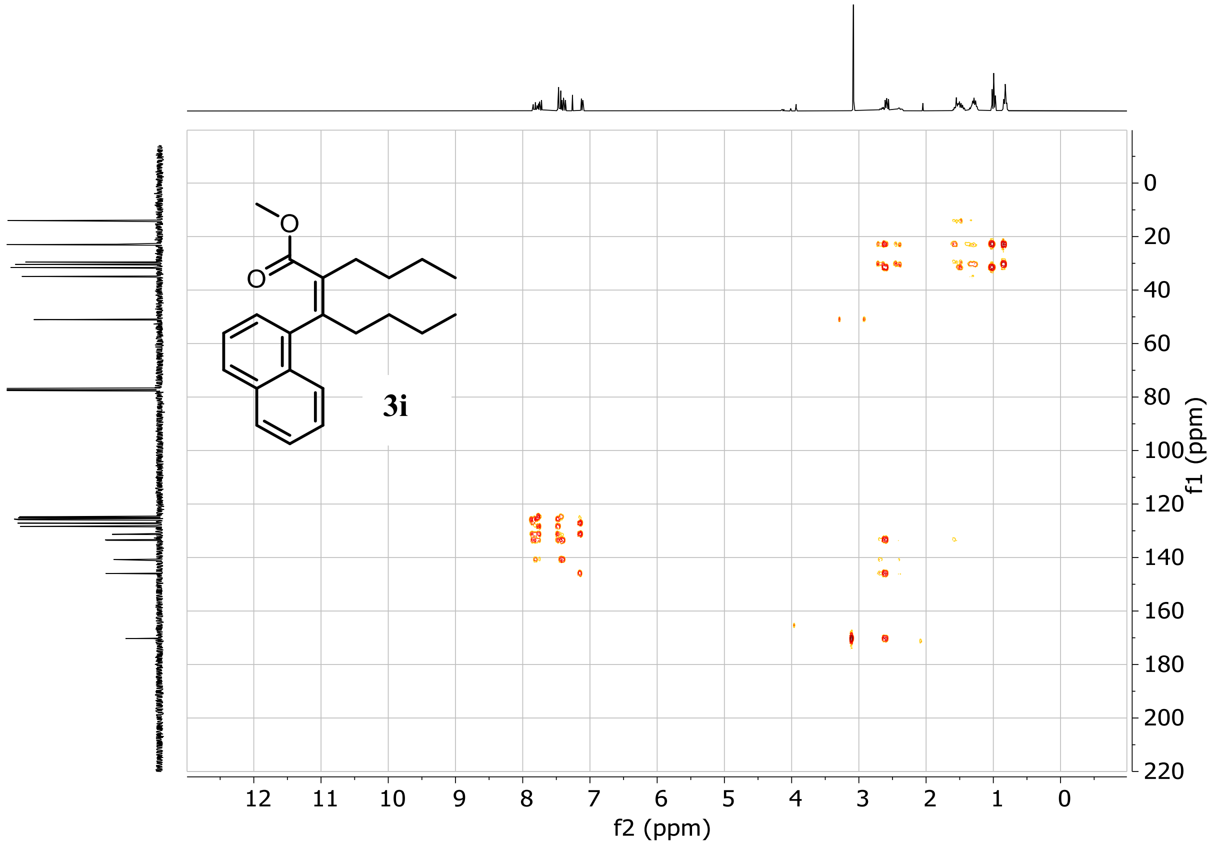
3i
12 11 10 9 8 7 6 5 4 3 2 1 0
f2 (ppm)
0 20 40 60 80 100 120 140 160 180 200 220
f1 (ppm)

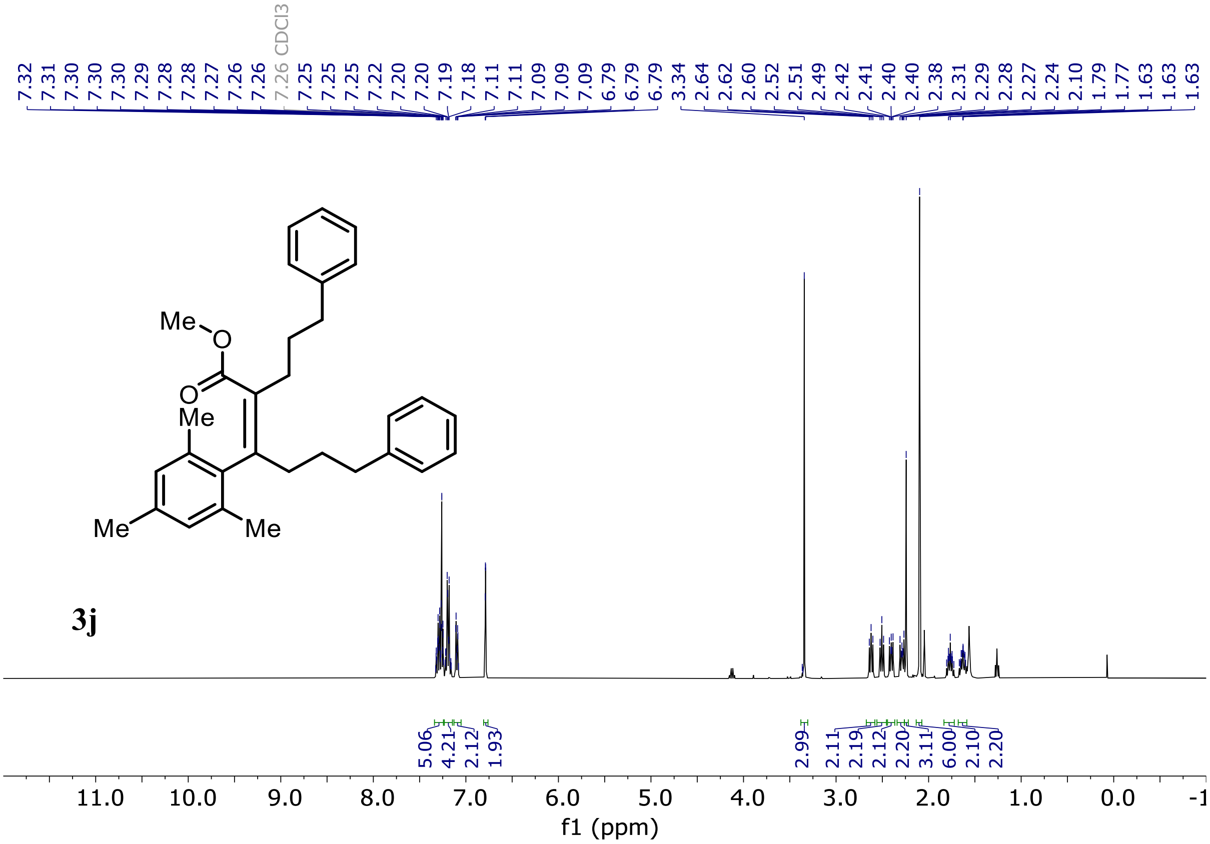
7.32
7.31
7.30
7.30
7.30
7.29
7.28
7.28
7.27
7.26
7.26
7.26 CDCl3
7.25
7.25
7.25
7.22
7.20
7.20
7.19
7.18
7.11
7.11
7.09
7.09
7.09
6.79
6.79
6.79
3.34
2.64
2.62
2.60
2.52
2.51
2.49
2.42
2.41
2.40
2.40
2.38
2.31
2.29
2.28
2.27
2.24
2.10
1.79
1.77
1.63
1.63
Me
O
O
Me
Me
Me
5.06
4.21
2.12
1.93
2.99
2.11
2.19
2.12
2.20
3.11
6.00
2.10
2.20
f1 (ppm)

3j

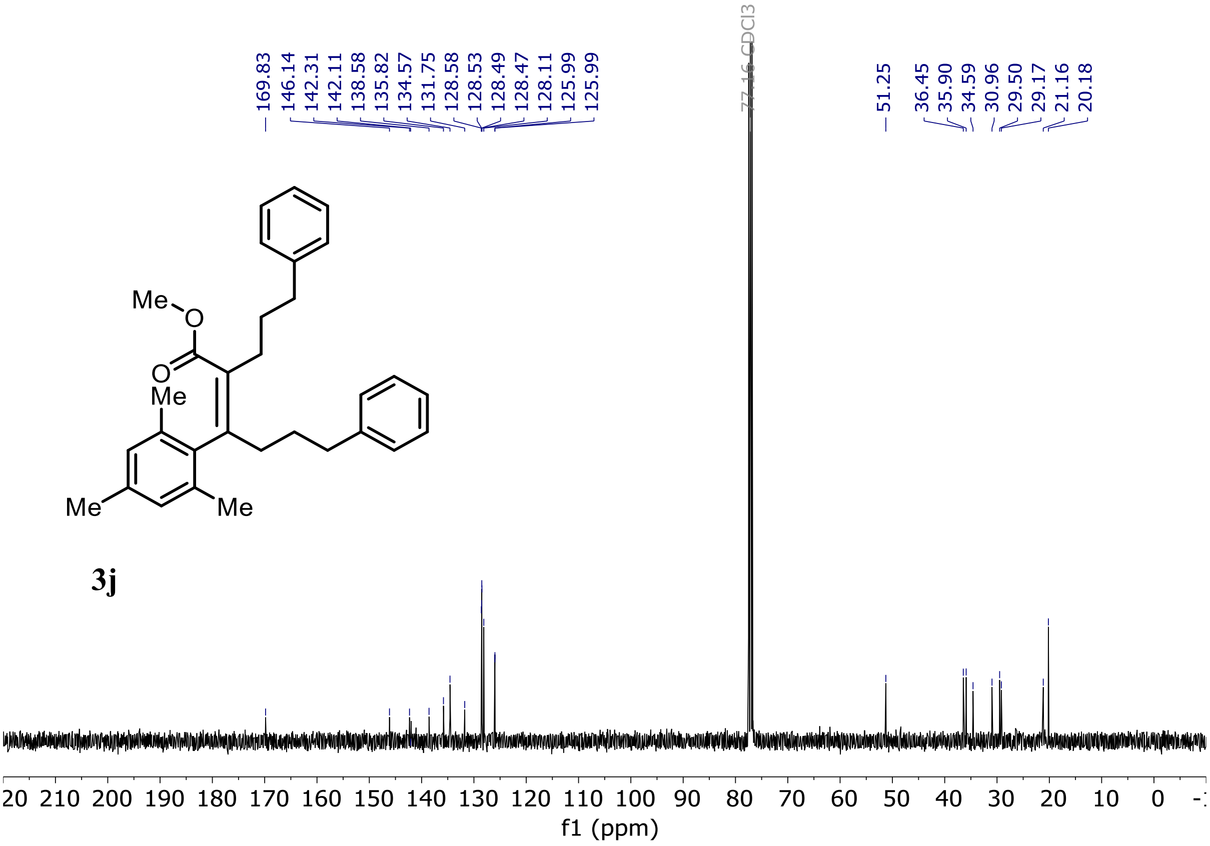
169.83
146.14
142.31
142.11
138.58
135.82
134.57
131.75
128.58
128.53
128.49
128.47
128.11
125.99
125.99
77.16 CDCl3
51.25
36.45
35.90
34.59
30.96
29.50
29.17
21.16
20.18
Me
O
O
Me
Me
Me
f1 (ppm)

3j

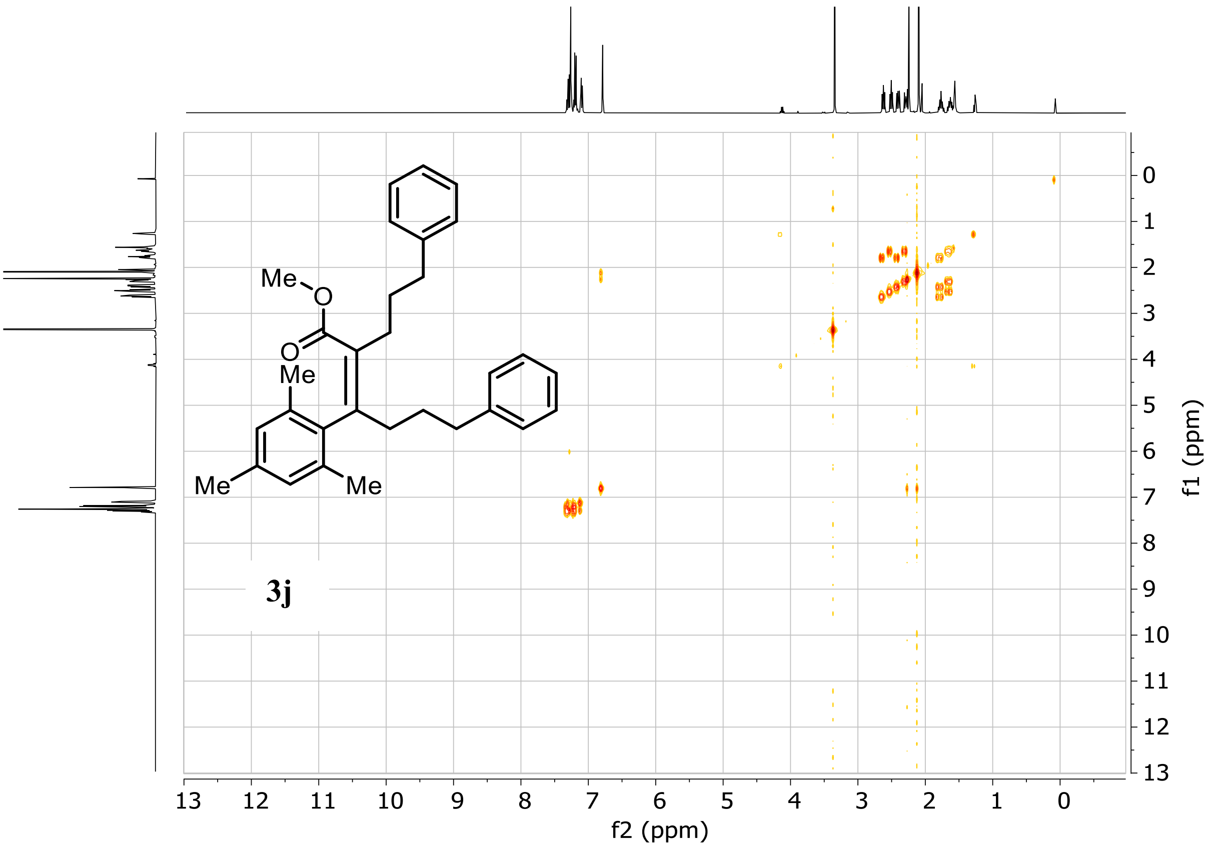
Me
O
O
Me
Me
Me
3j
f2 (ppm)
f1 (ppm)

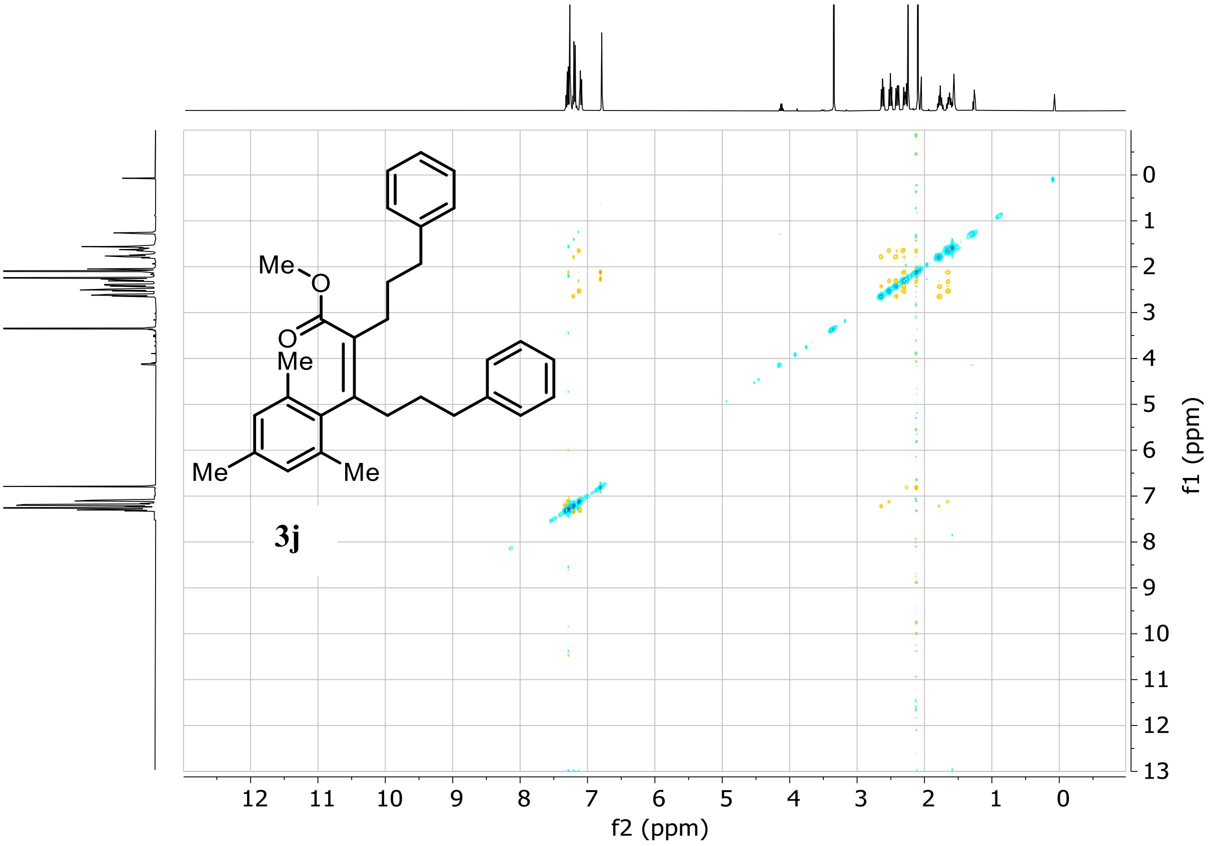
Me
O
O
Me
Me
Me
3j
f2 (ppm)
f1 (ppm)

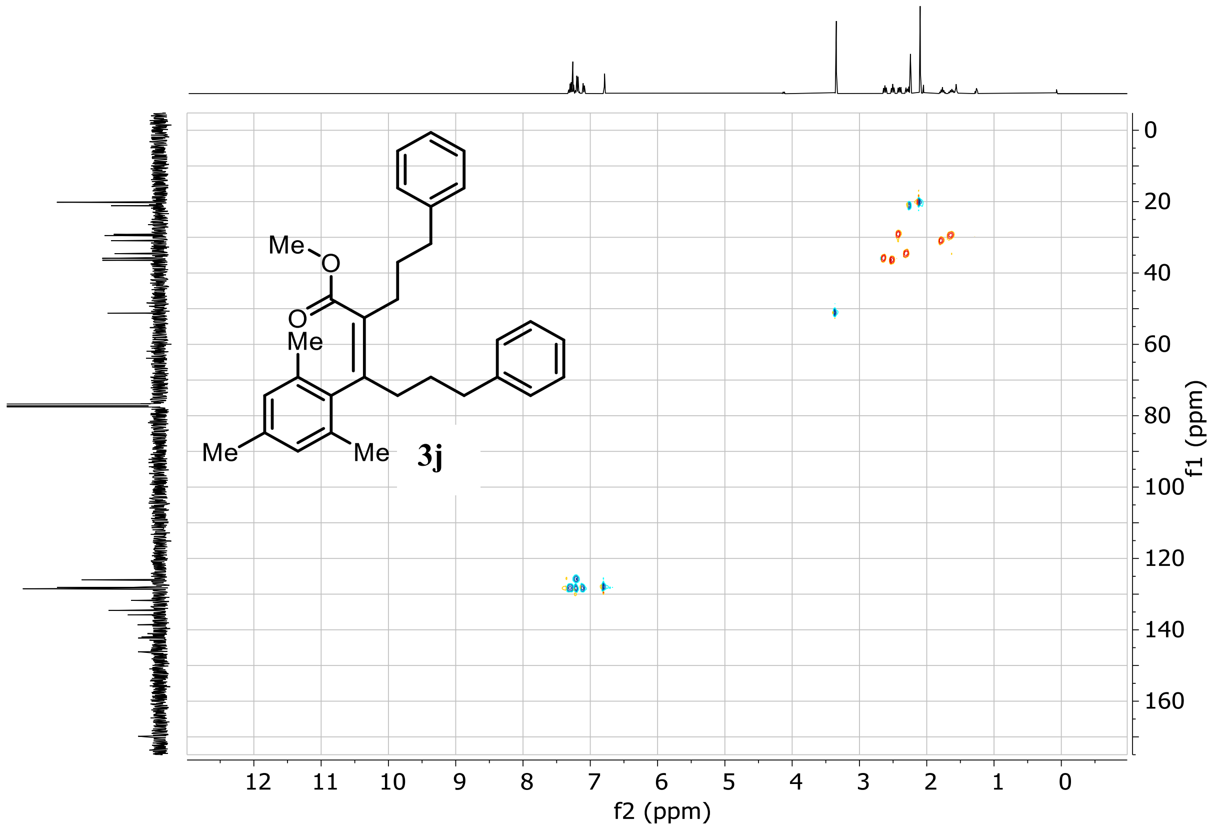
Me
O
O
Me
Me
Me
3j
f1 (ppm)
f2 (ppm)

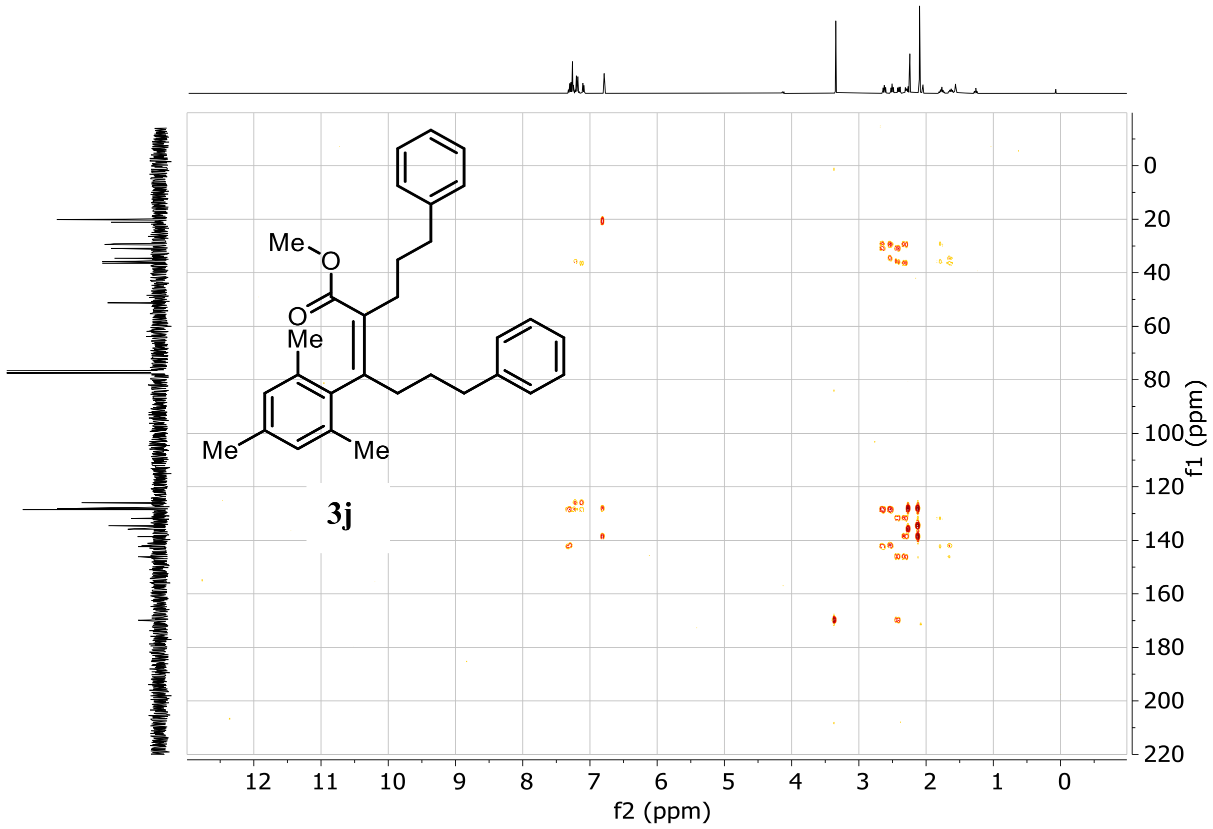
Me
O
O
Me
Me
Me
3j
f1 (ppm)
f2 (ppm)

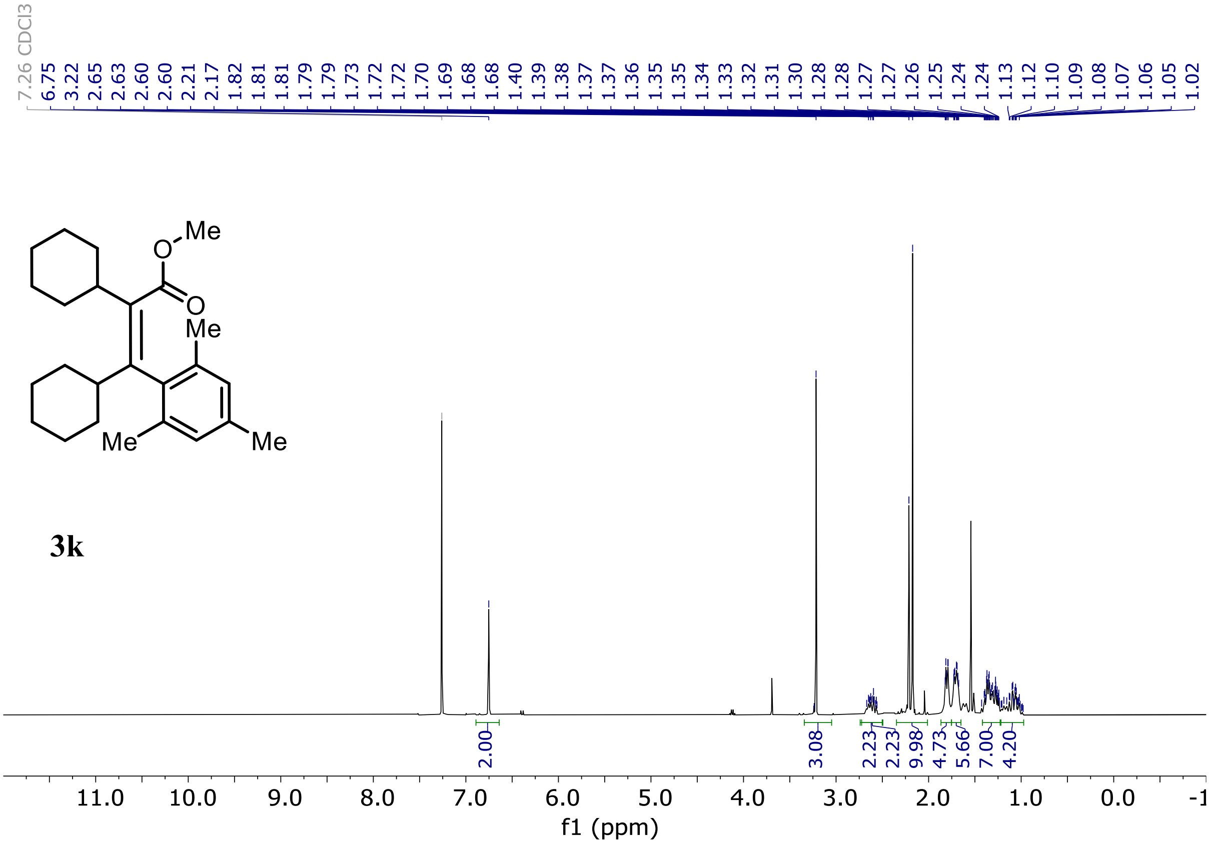
Me
O
O
Me
Me
Me
3k
7.26 CDCl3
6.75
3.22
2.65
2.63
2.60
2.60
2.21
2.17
1.82
1.81
1.81
1.79
1.79
1.73
1.72
1.72
1.70
1.69
1.68
1.68
1.40
1.39
1.38
1.37
1.37
1.36
1.35
1.35
1.34
1.33
1.32
1.31
1.30
1.28
1.28
1.27
1.27
1.26
1.25
1.24
1.24
1.13
1.12
1.10
1.09
1.08
1.07
1.06
1.05
1.02
2.00
3.08
2.23
2.23
9.98
4.73
5.66
7.00
4.20
11.0 10.0 9.0 8.0 7.0 6.0 5.0 4.0 3.0 2.0 1.0 0.0 -1
f1 (ppm)

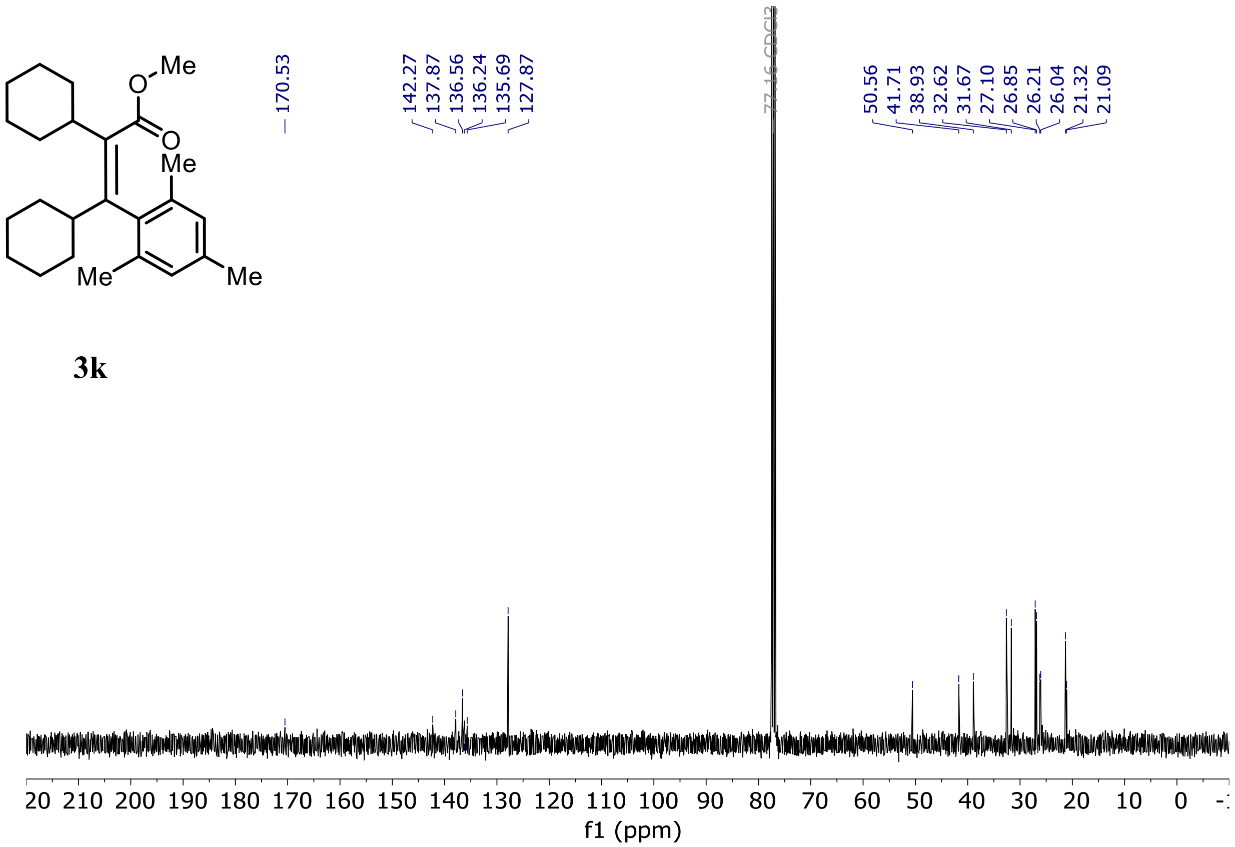
Me
O
O
Me
Me
Me
3k
170.53
142.27
137.87
136.56
136.24
135.69
127.87
77.16 CDCl3
50.56
41.71
38.93
32.62
31.67
27.10
26.85
26.21
26.04
21.32
21.09
20 210 200 190 180 170 160 150 140 130 120 110 100 90 80 70 60 50 40 30 20 10 0
f1 (ppm)

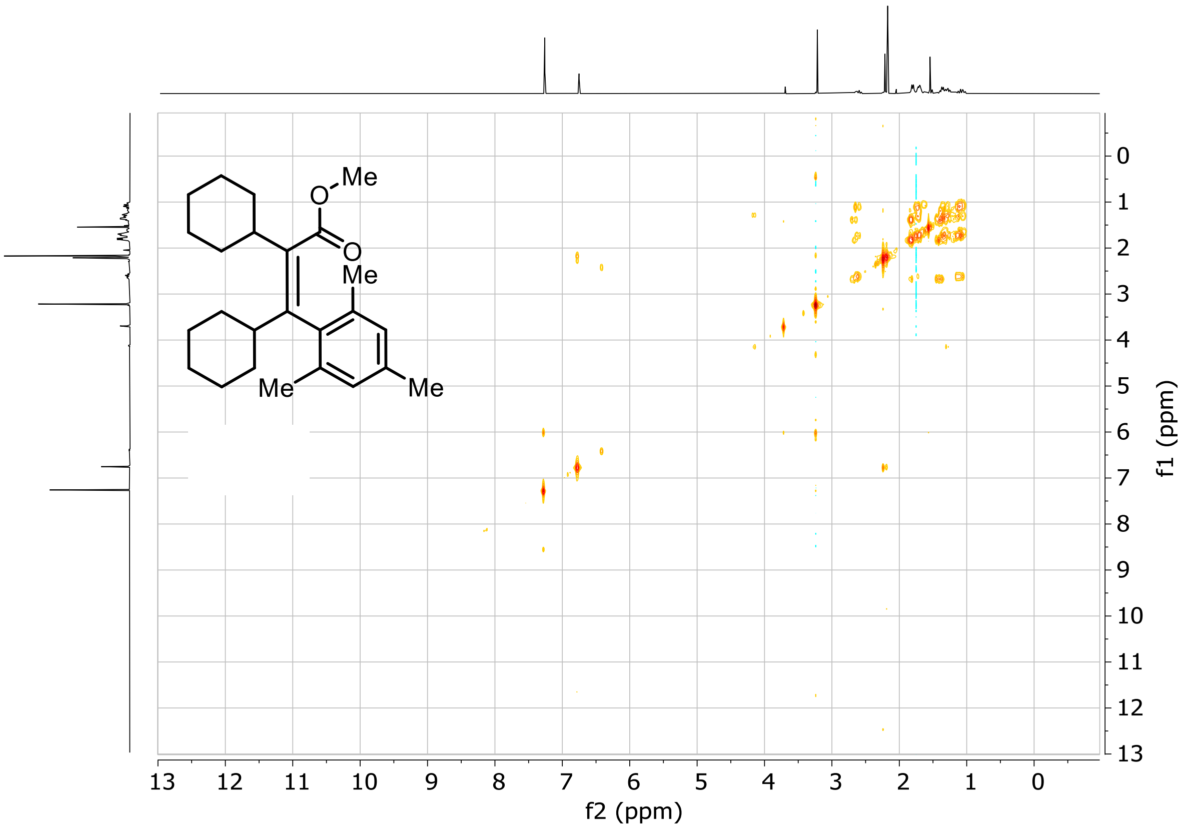

Me
O
O
Me
Me
Me
f1 (ppm)
f2 (ppm)

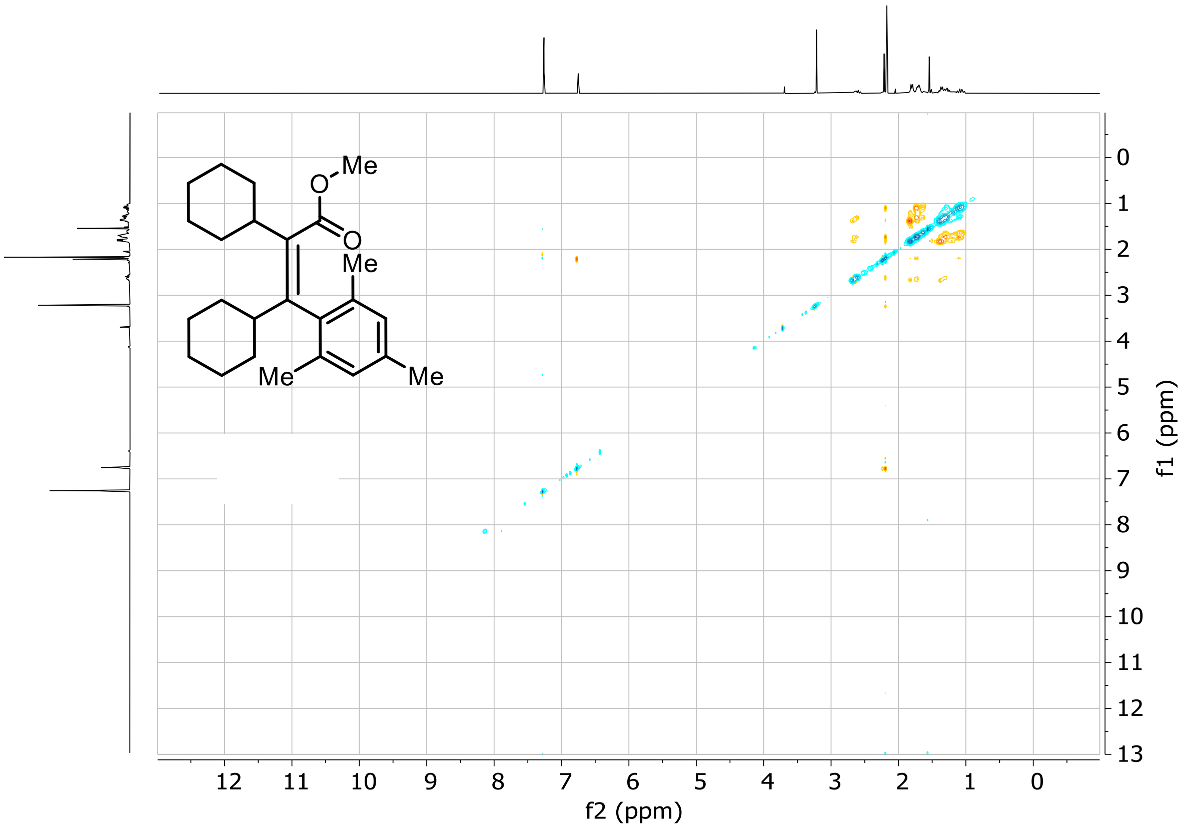

Me
O
O
Me
Me
Me
f1 (ppm)
f2 (ppm)

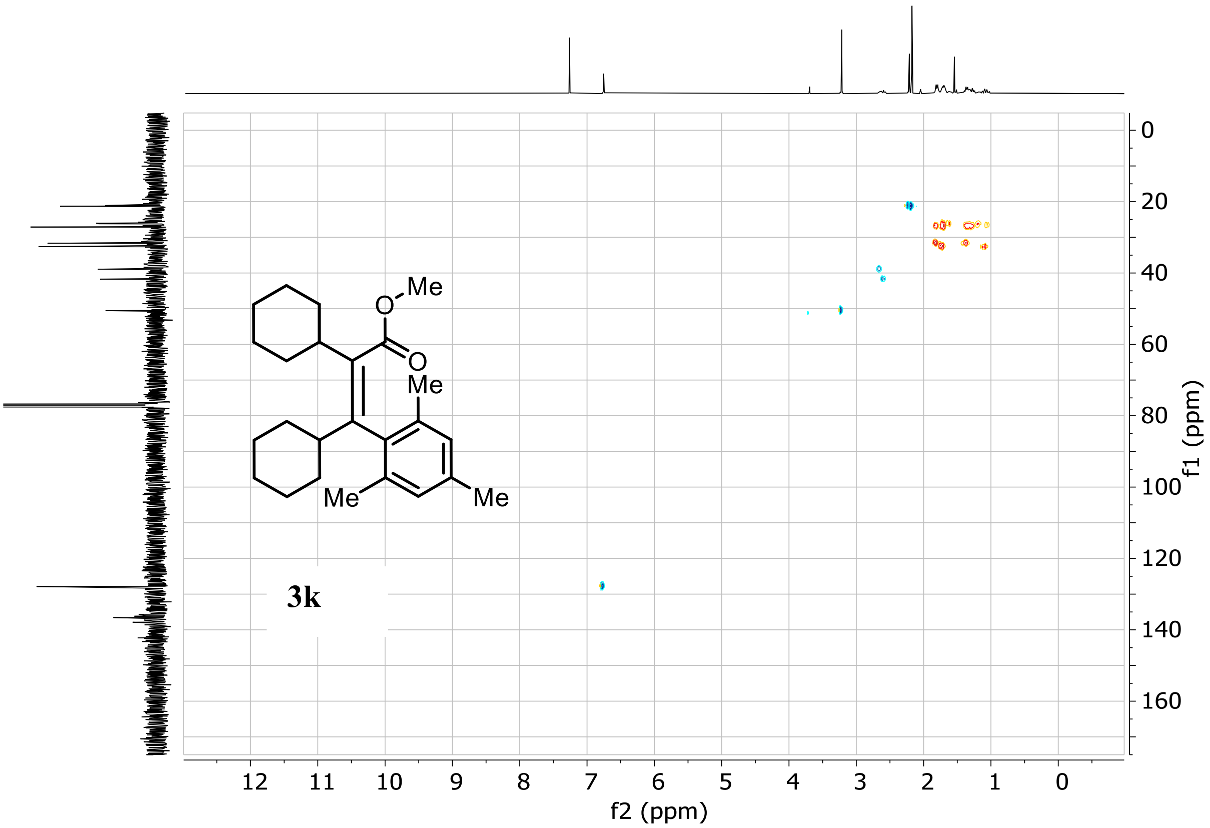
Me
O
O
Me
Me
Me
3k
f2 (ppm)
f1 (ppm)

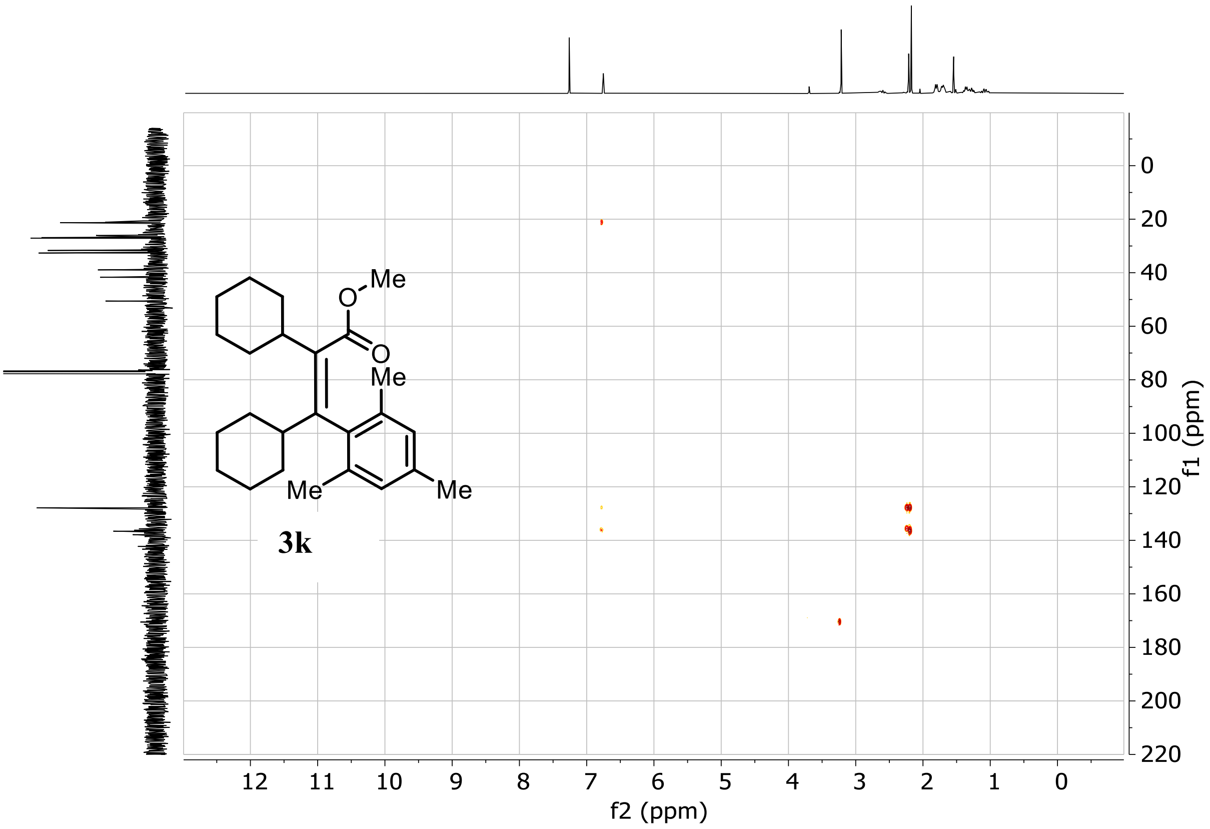
Me
O
O
Me
Me
Me
3k
f2 (ppm)
f1 (ppm)

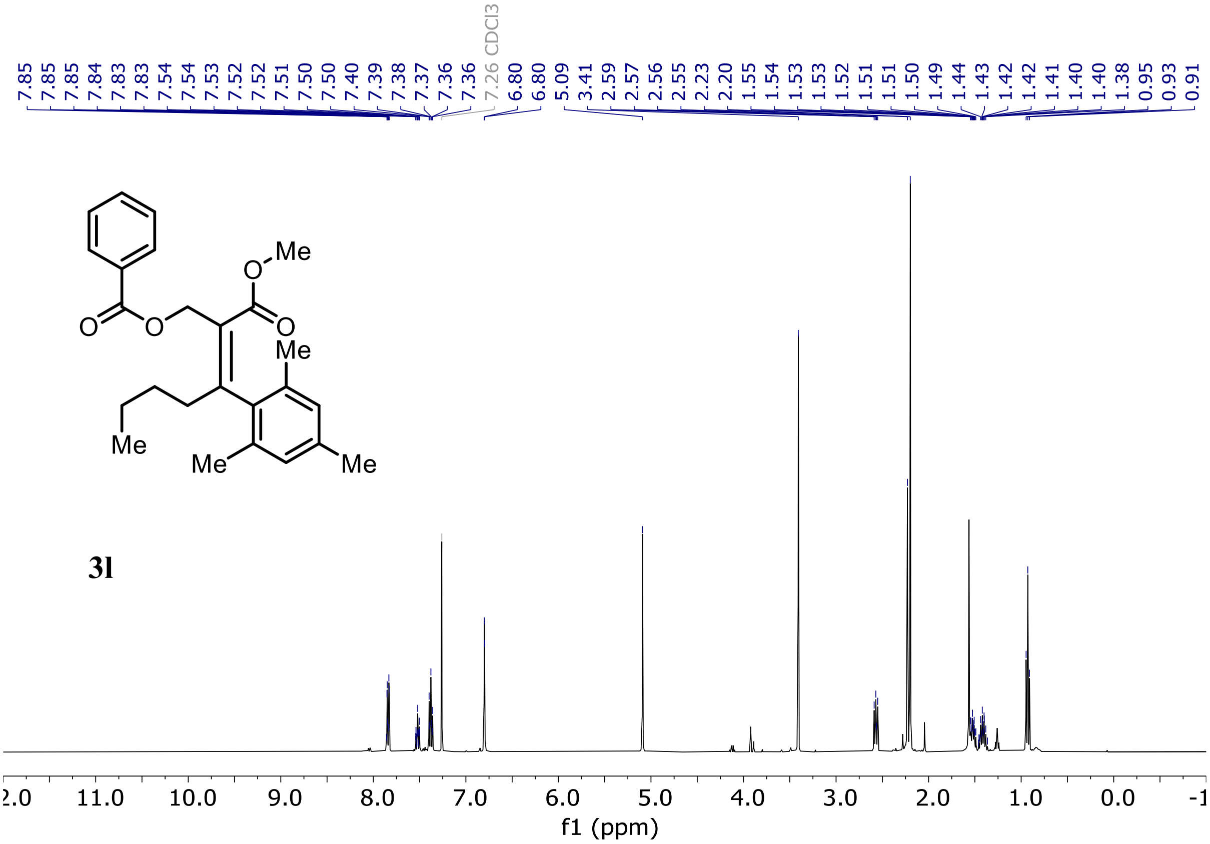
7.85 7.85 7.85 7.84 7.83 7.83 7.54 7.54 7.53 7.52 7.52 7.51 7.50 7.50 7.40 7.39 7.38 7.37 7.36 7.36 7.26 CDCl3 6.80 6.80 5.09 3.41 2.59 2.57 2.56 2.55 2.23 2.20 1.55 1.54 1.53 1.53 1.52 1.51 1.51 1.50 1.49 1.44 1.43 1.42 1.42 1.41 1.40 1.40 1.38 0.95 0.93 0.91
Me
O
O
O
O
Me
Me
Me
Me
3l
2.0 11.0 10.0 9.0 8.0 7.0 6.0 5.0 4.0 3.0 2.0 1.0 0.0 -1
f1 (ppm)

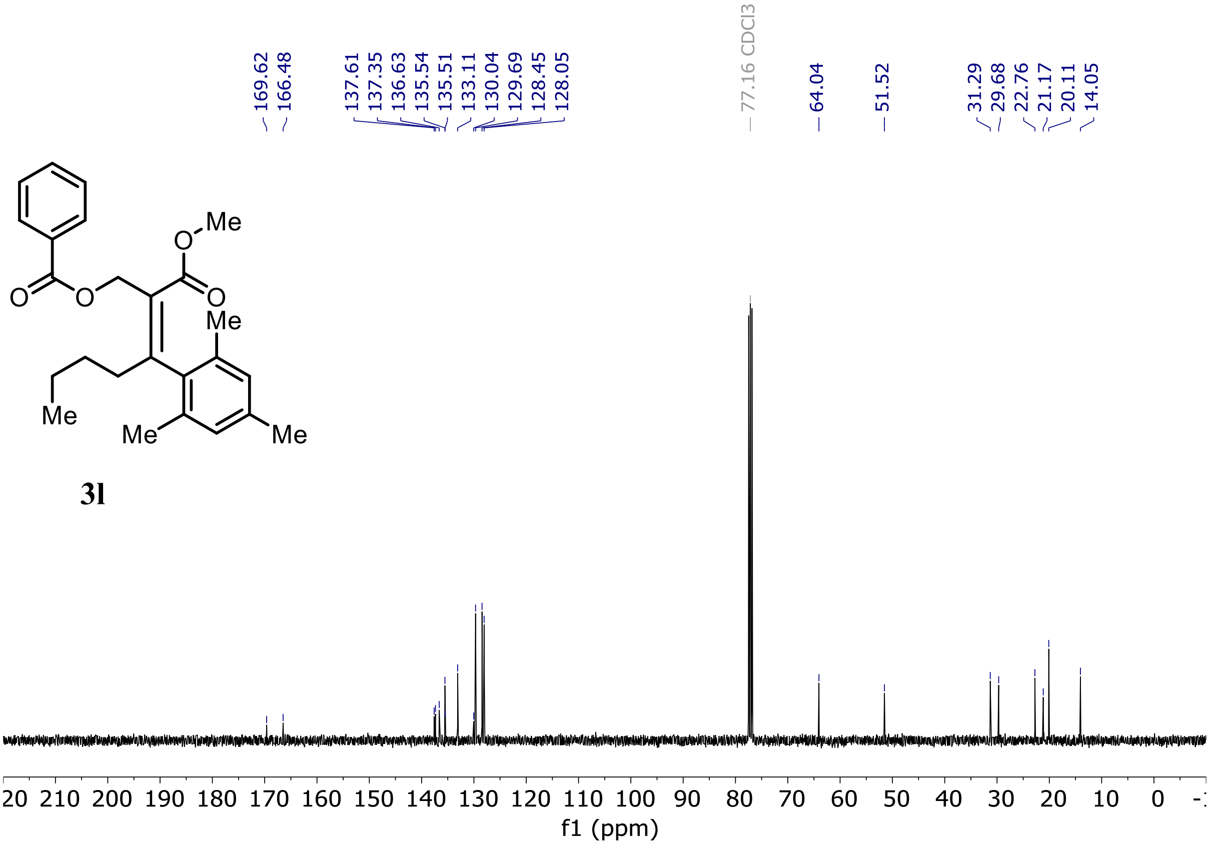
169.62 166.48 137.61 137.35 136.63 135.54 135.51 133.11 130.04 129.69 128.45 128.05 77.16 CDCl3 64.04 51.52 31.29 29.68 22.76 21.17 20.11 14.05
Me
O
O
O
O
Me
Me
Me
Me
3l
20 210 200 190 180 170 160 150 140 130 120 110 100 90 80 70 60 50 40 30 20 10 0 -1
f1 (ppm)

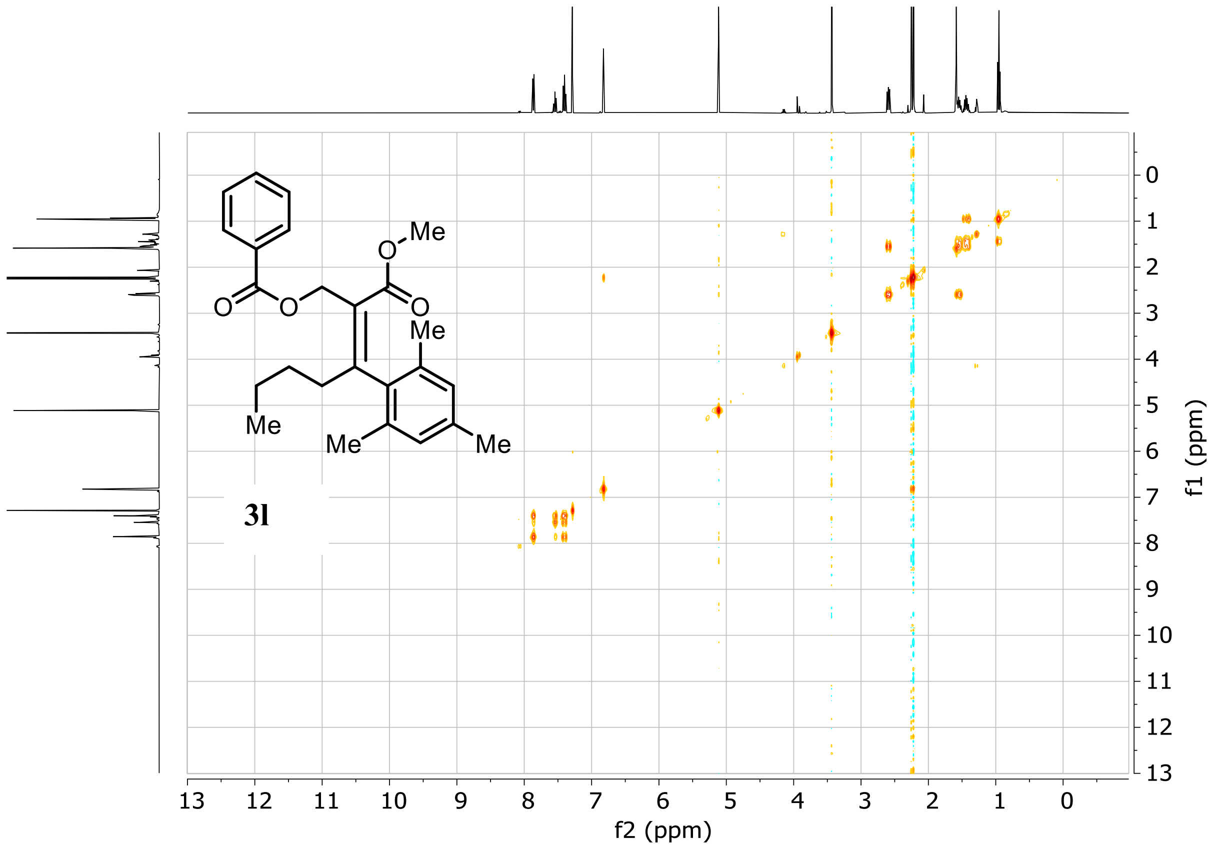
3l
Me
O
Me
Me
Me
Me
f2 (ppm)
f1 (ppm)

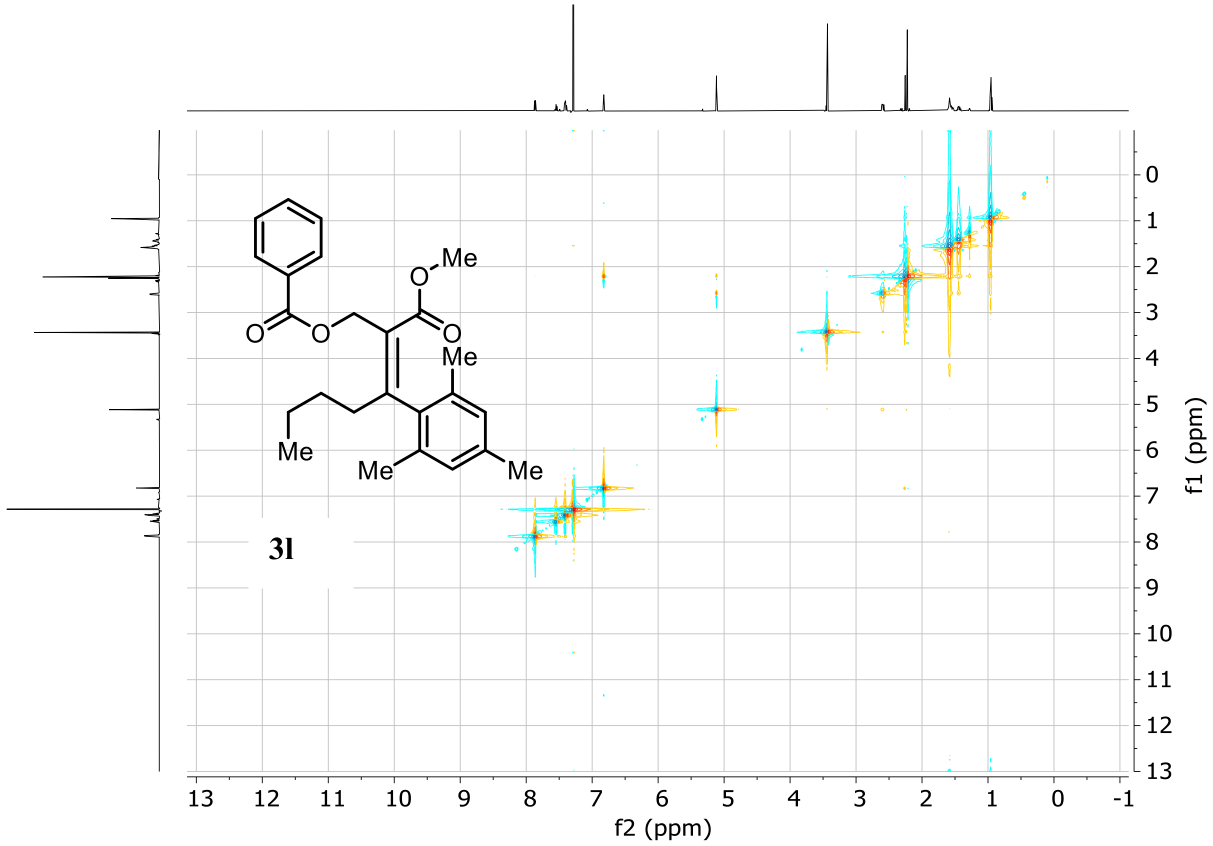
3l
Me
O
Me
Me
Me
Me
f2 (ppm)
f1 (ppm)

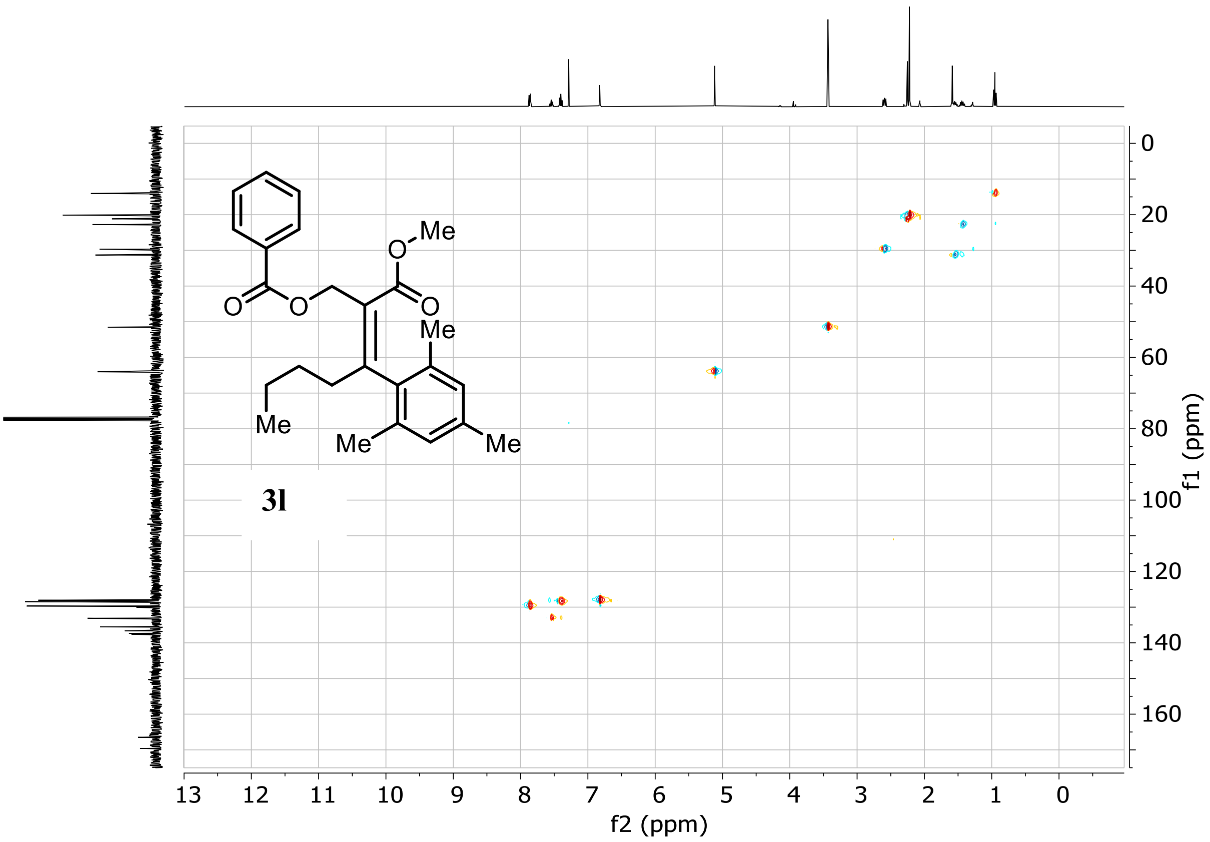
Me
O
O
O
O
Me
Me
Me
Me
3l
f1 (ppm)
f2 (ppm)

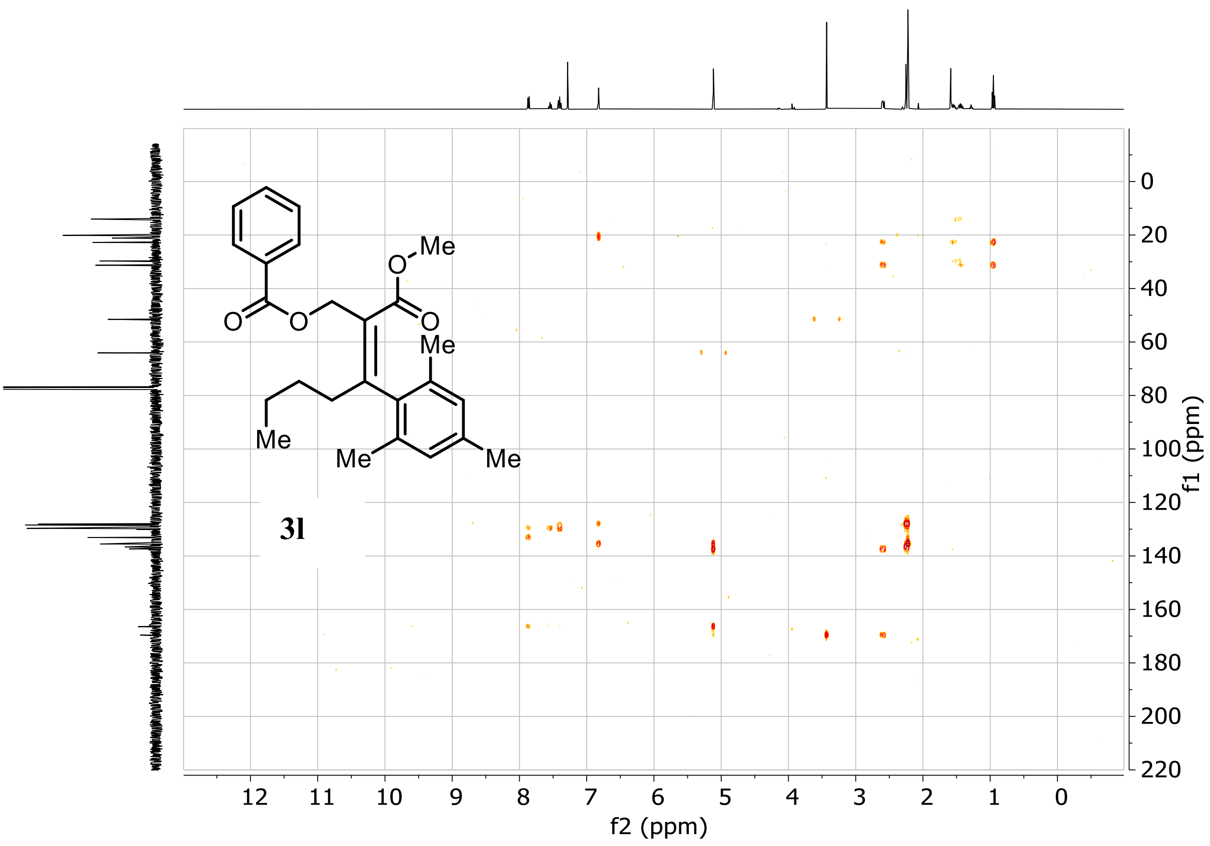
Me
O
O
O
O
Me
Me
Me
Me
3l
f1 (ppm)
f2 (ppm)

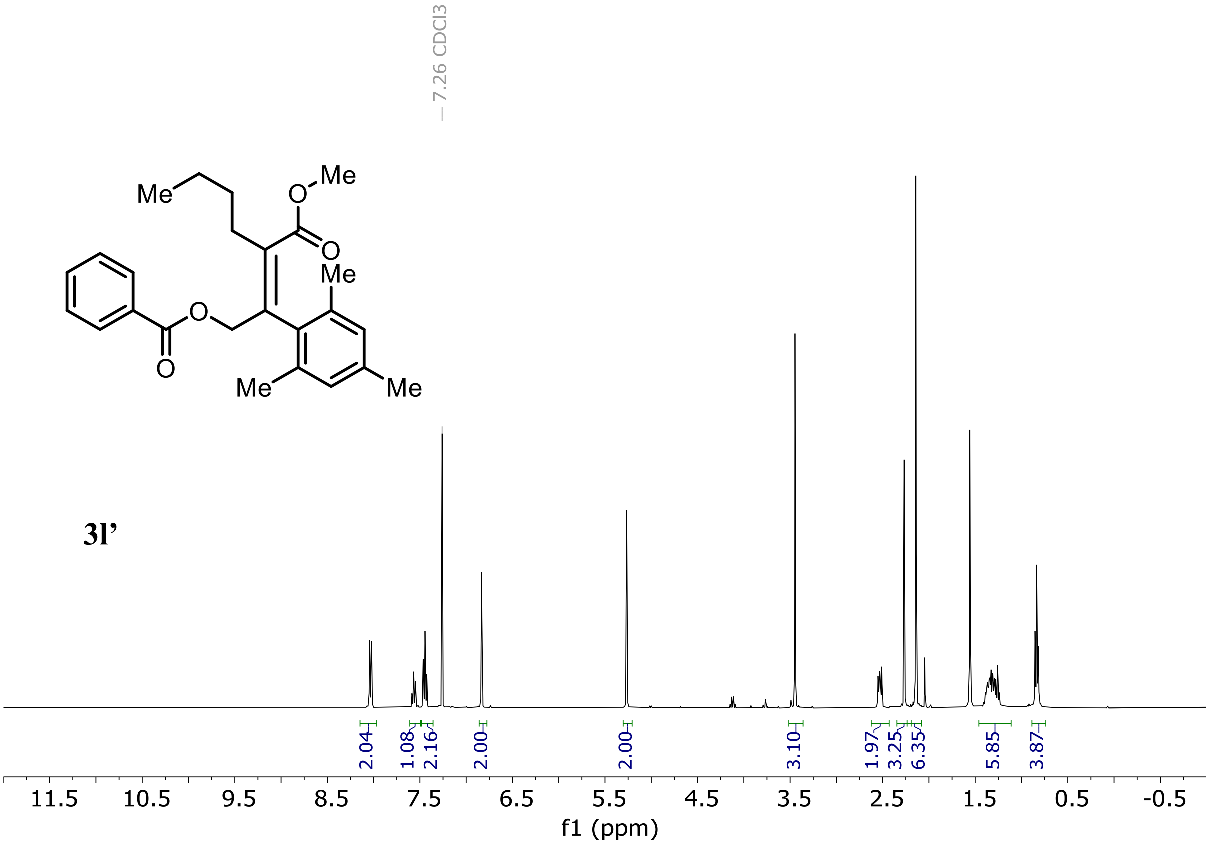
7.26 CDCl3
Me
O
Me
O
Me
O
O
Me
Me
2.04
1.08
2.16
2.00
2.00
3.10
1.97
3.25
6.35
5.85
3.87
11.5
10.5
9.5
8.5
7.5
6.5
5.5
4.5
3.5
2.5
1.5
0.5
-0.5
f1 (ppm)

3l'

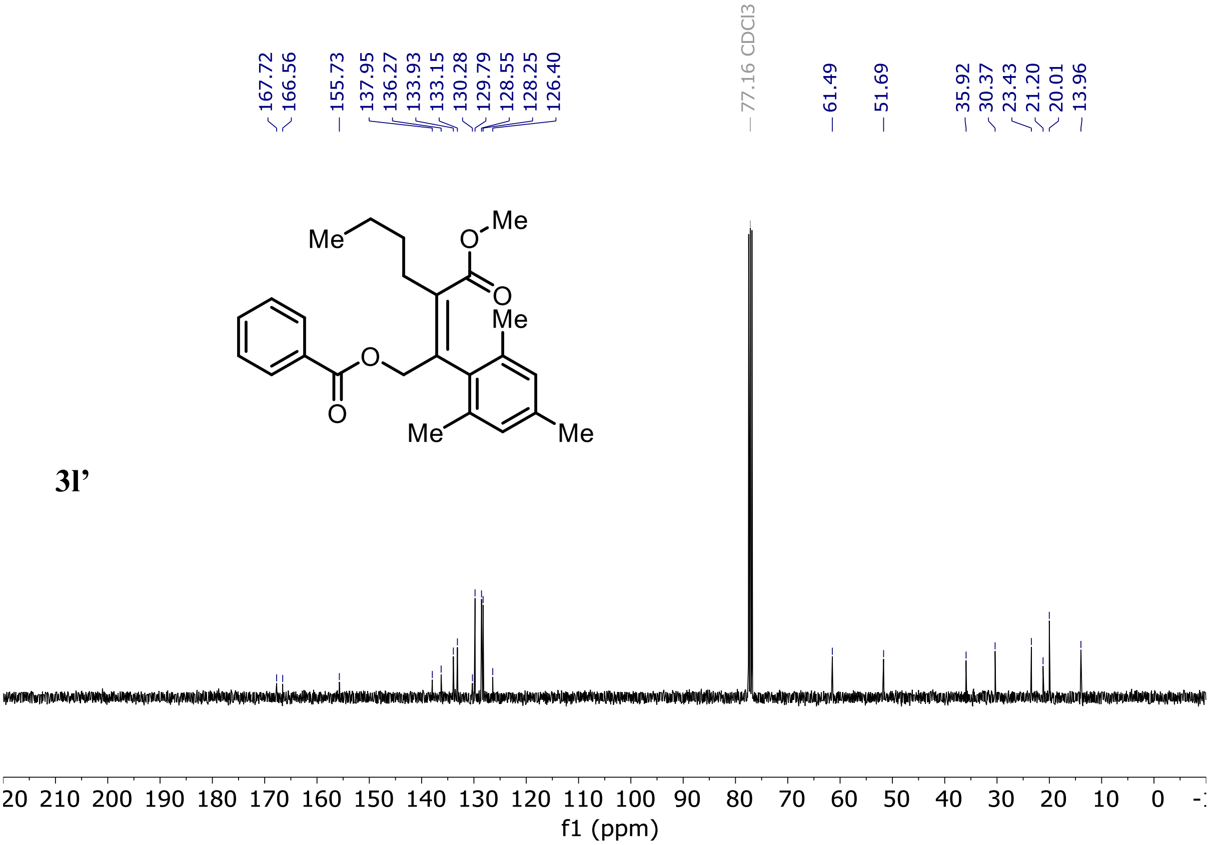
167.72
166.56
155.73
137.95
136.27
133.93
133.15
130.28
129.79
128.55
128.25
126.40
77.16 CDCl3
61.49
51.69
35.92
30.37
23.43
21.20
20.01
13.96
Me
O
Me
O
Me
O
O
Me
Me
210
200
190
180
170
160
150
140
130
120
110
100
90
80
70
60
50
40
30
20
10
0
f1 (ppm)

3l'

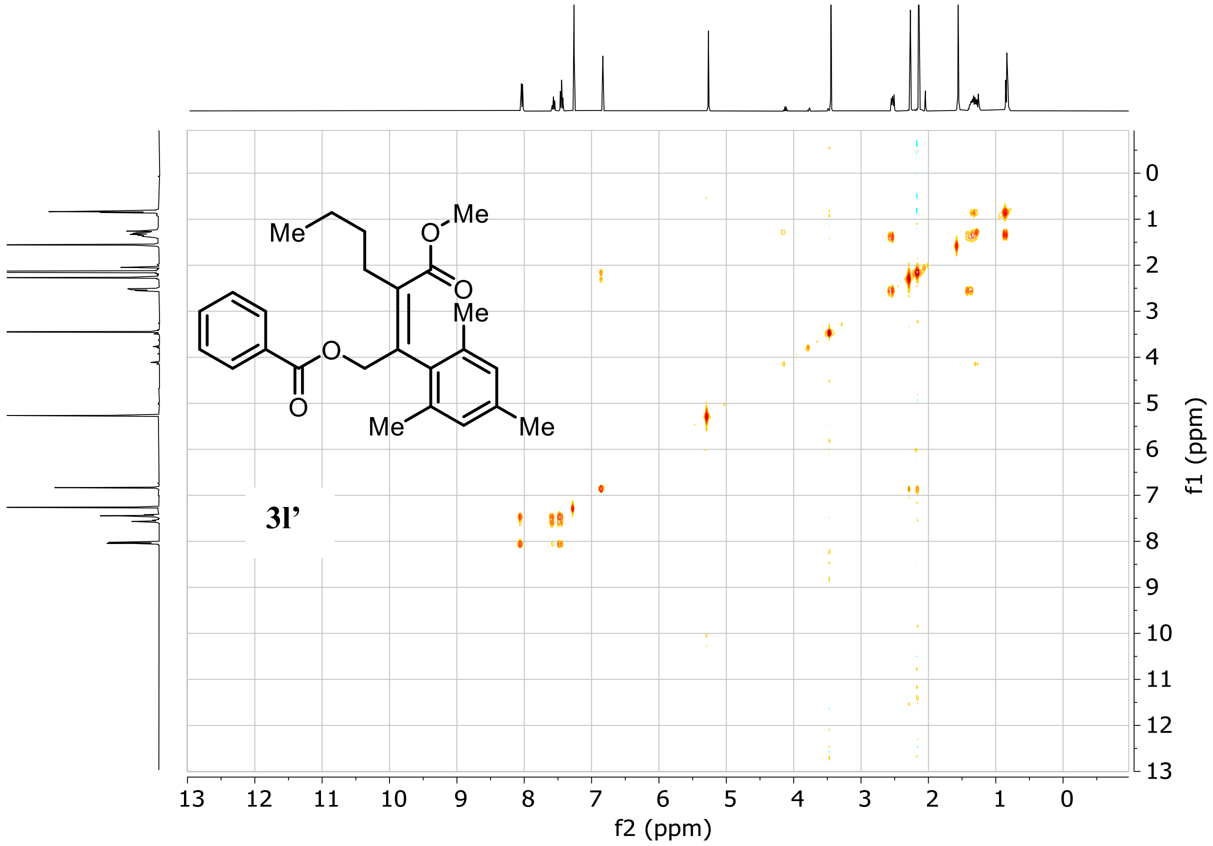
Me
O
Me
O
Me
O
O
Me
Me
3l’
f2 (ppm)
f1 (ppm)

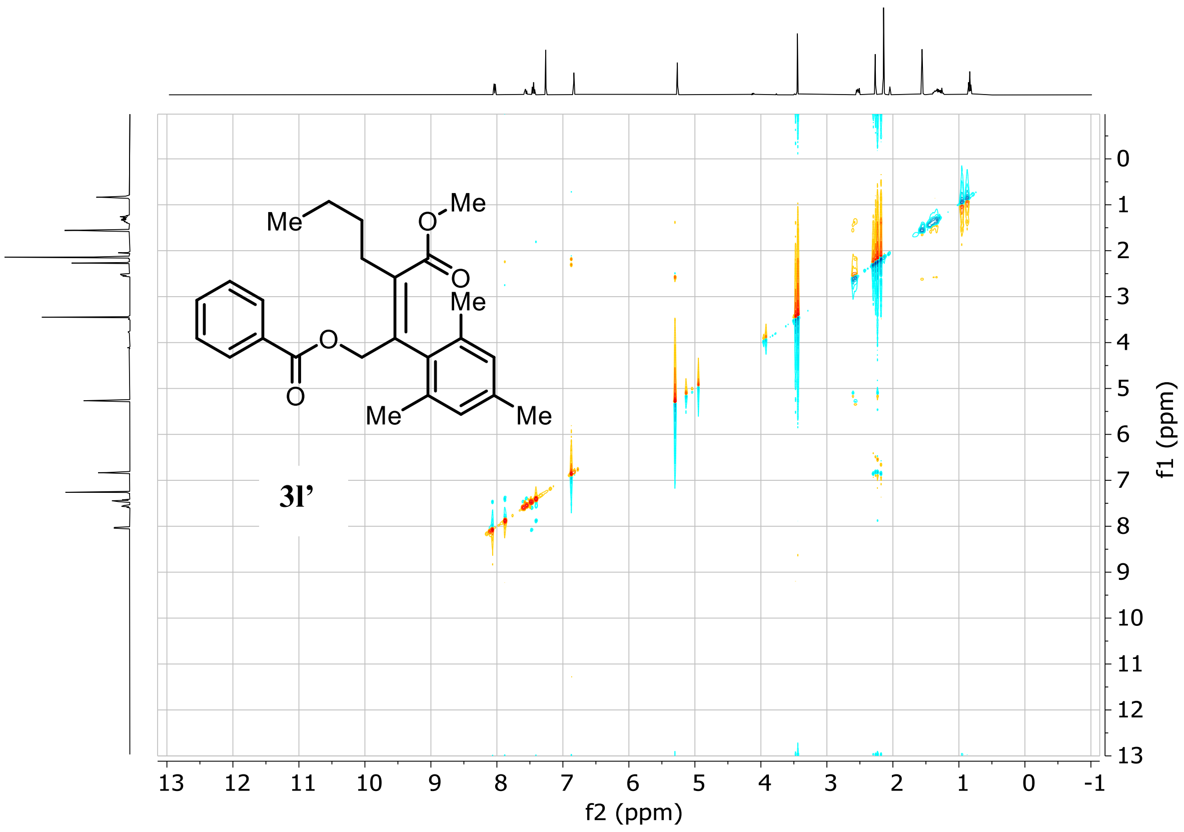
Me
O
Me
O
Me
O
O
Me
Me
3l’
f2 (ppm)
f1 (ppm)

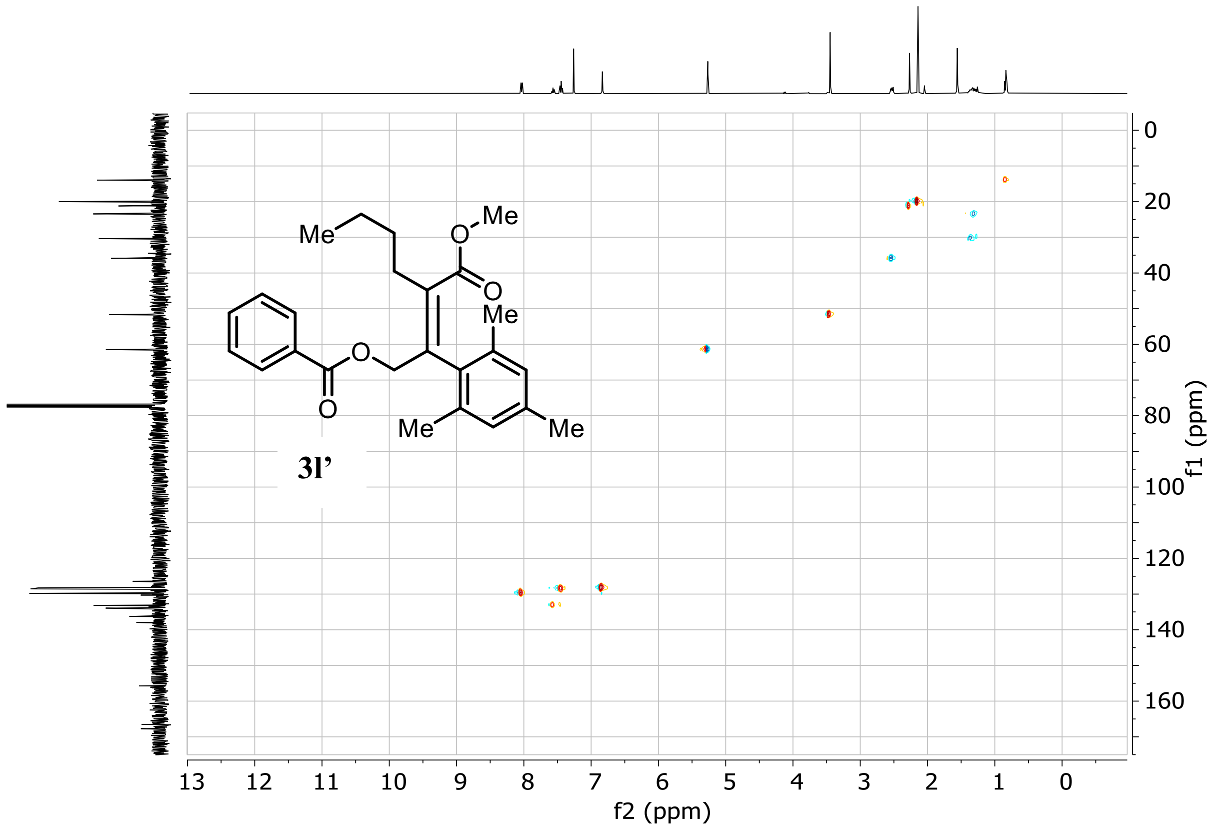
3l'
f1 (ppm)
f2 (ppm)

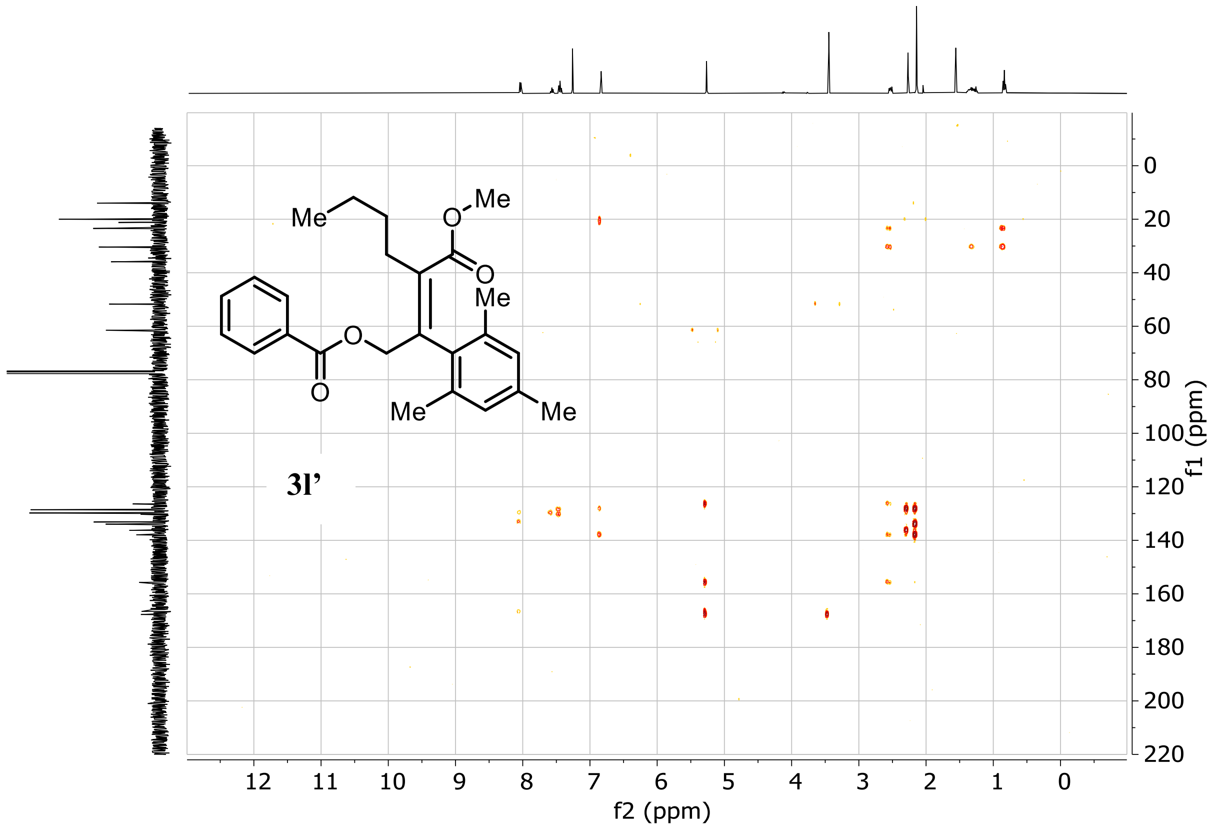
3l'
f1 (ppm)
f2 (ppm)

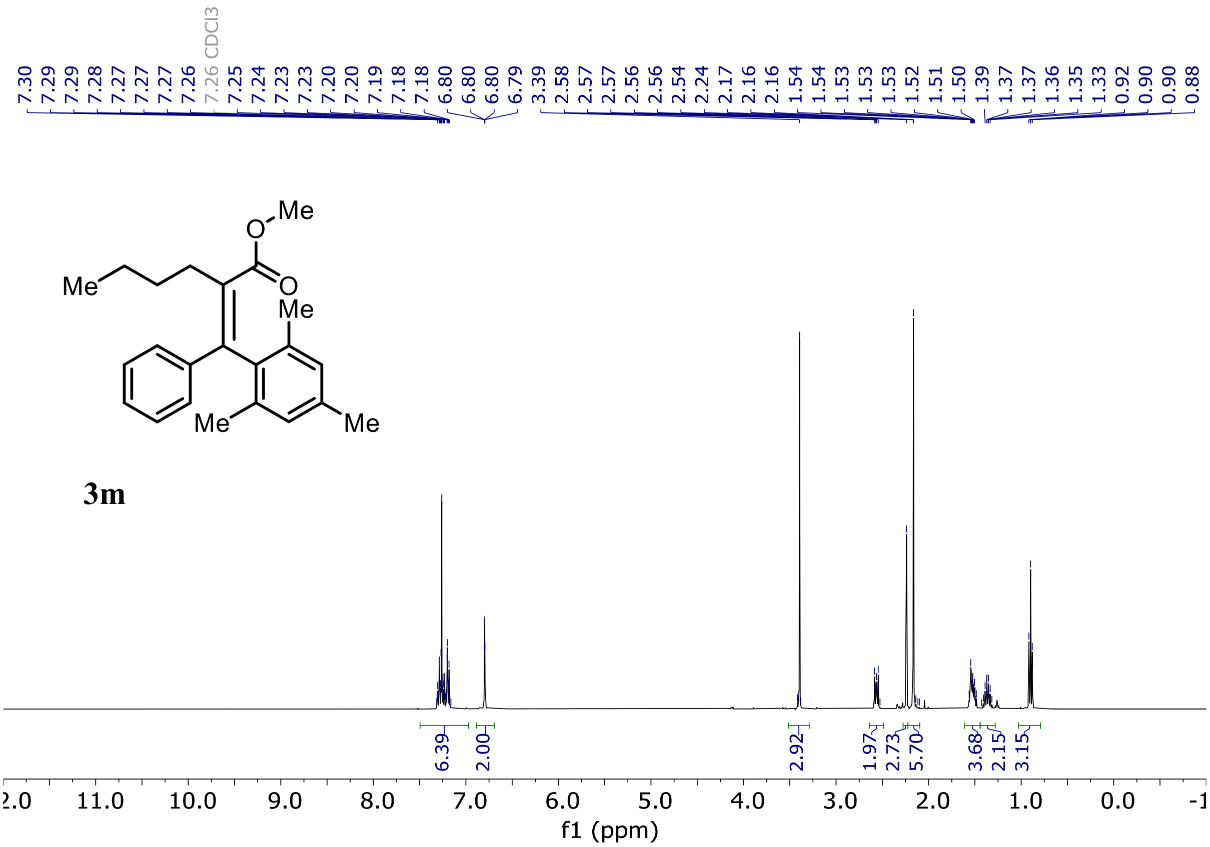
7.30
7.29
7.29
7.28
7.27
7.27
7.27
7.26
7.26 CDCl3
7.25
7.24
7.23
7.23
7.20
7.20
7.19
7.18
7.18
6.80
6.80
6.80
6.79
3.39
2.58
2.57
2.57
2.56
2.56
2.54
2.24
2.17
2.16
2.16
1.54
1.54
1.53
1.53
1.53
1.52
1.51
1.50
1.39
1.37
1.37
1.36
1.35
1.33
0.92
0.90
0.90
0.88
Me
O
O
Me
Me
Me
Me
6.39
2.00
2.92
1.97
2.73
5.70
3.68
2.15
3.15
f1 (ppm)

3m

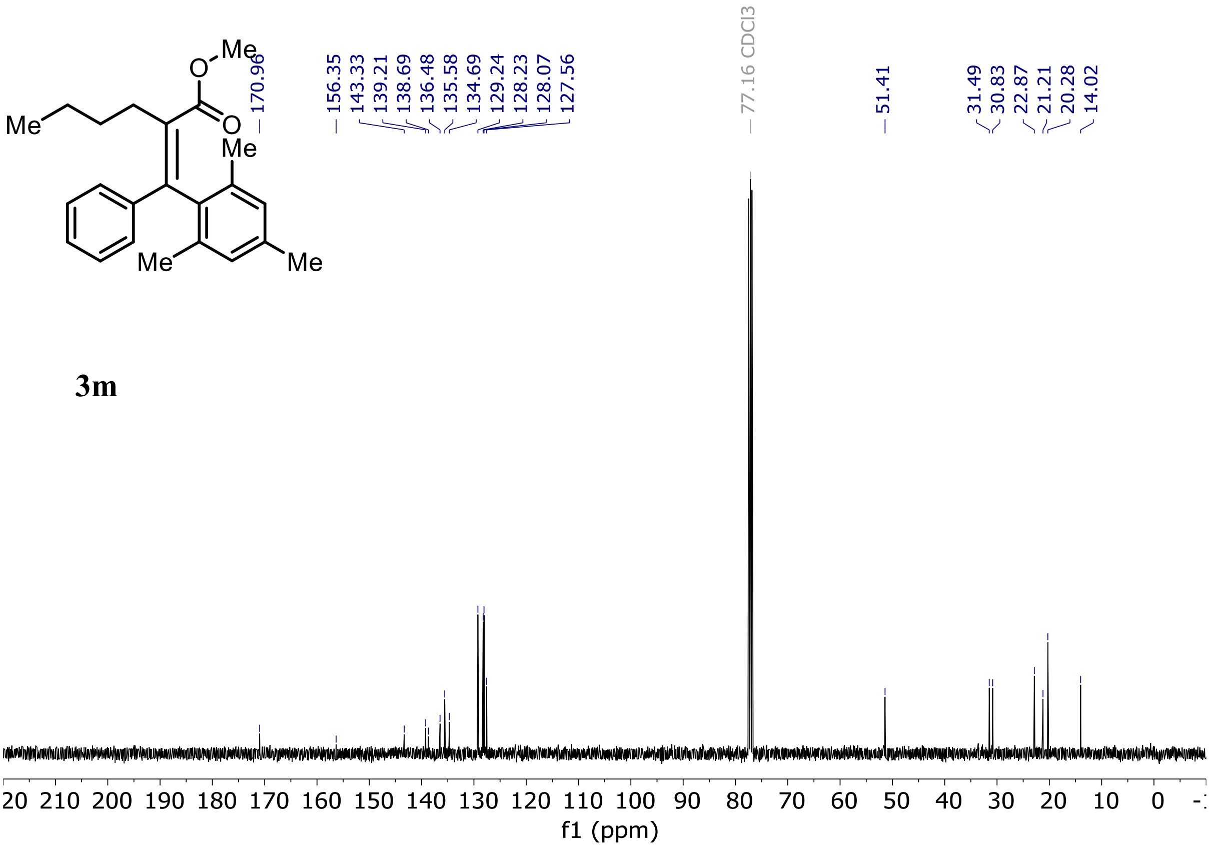
170.96
156.35
143.33
139.21
138.69
136.48
135.58
134.69
129.24
128.23
128.07
127.56
77.16 CDCl3
51.41
31.49
30.83
22.87
21.21
20.28
14.02
Me
O
O
Me
Me
Me
Me
f1 (ppm)

3m

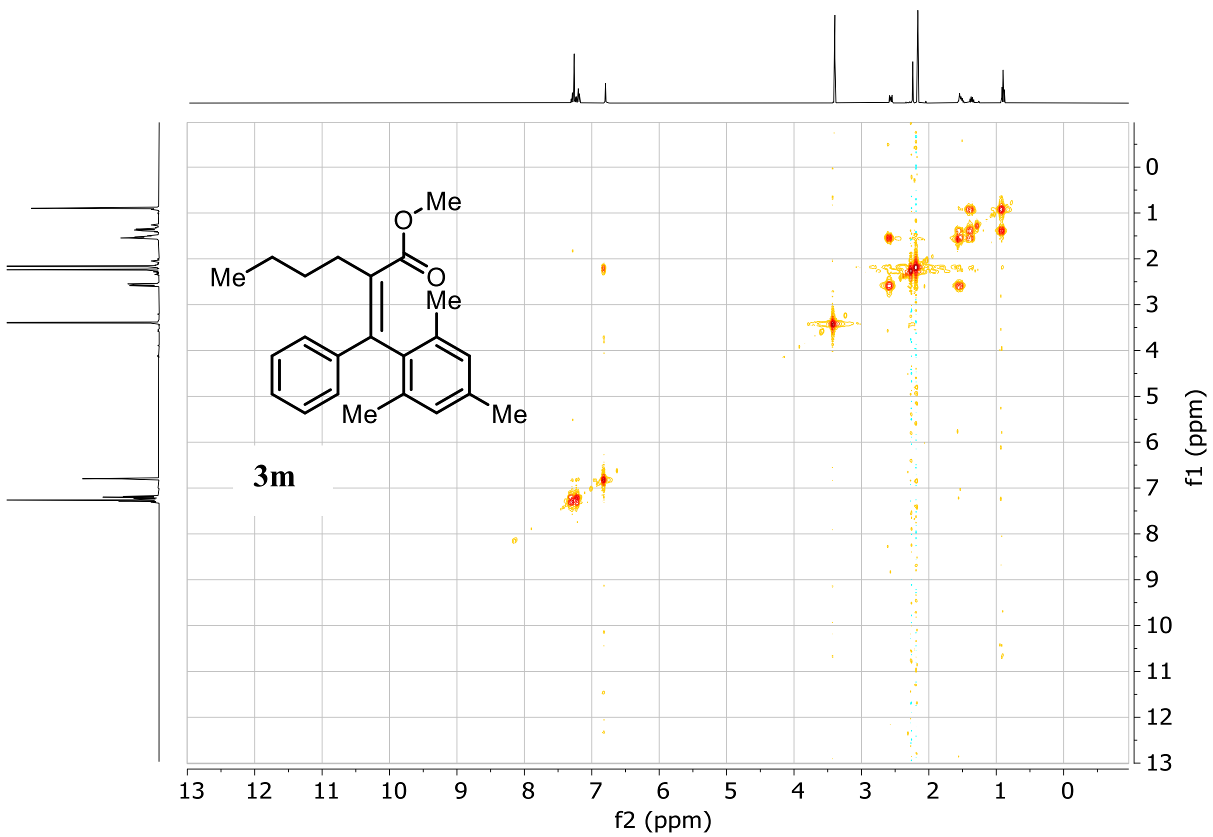
Me
O
O
Me
Me
Me
Me
3m
f2 (ppm)
f1 (ppm)

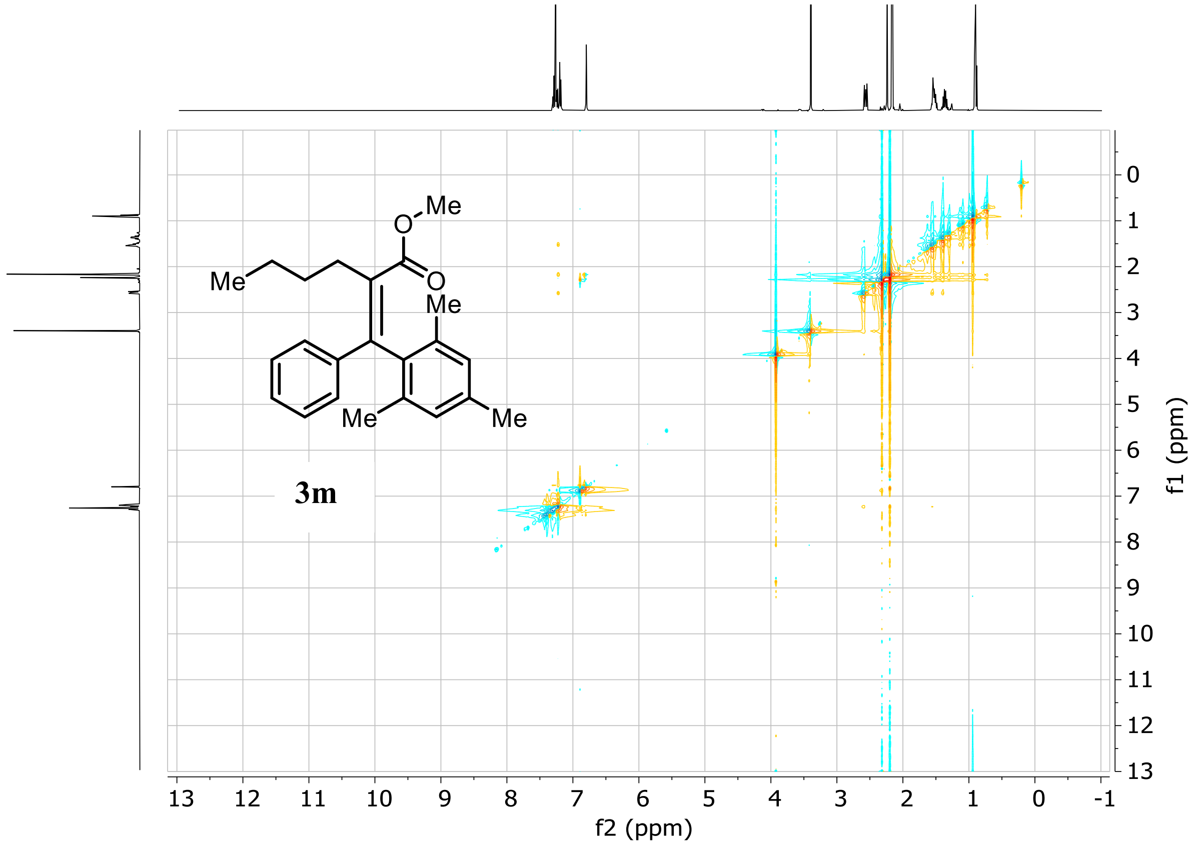
Me
O
O
Me
Me
Me
Me
3m
f2 (ppm)
f1 (ppm)

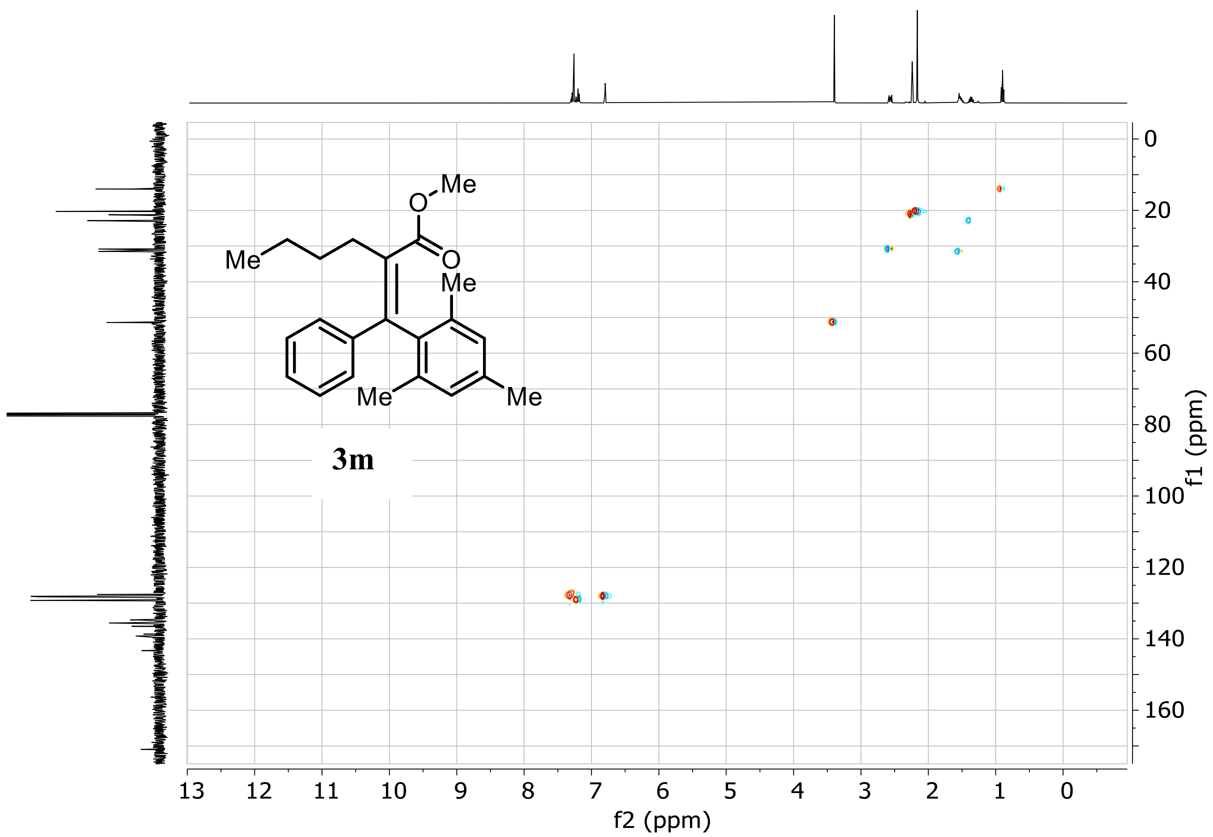
Me
O
O
Me
Me
Me
Me
3m
f2 (ppm)
f1 (ppm)

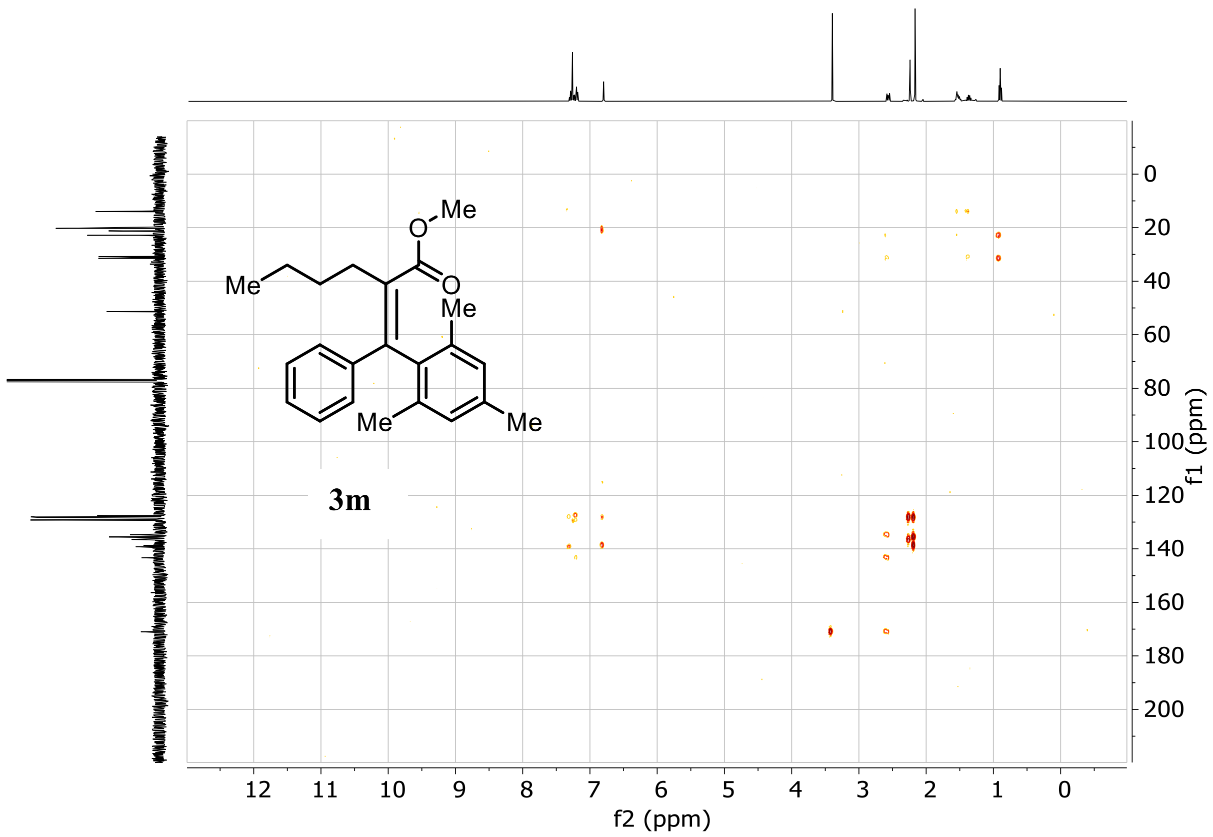
Me
O
O
Me
Me
Me
Me
3m
f2 (ppm)
f1 (ppm)

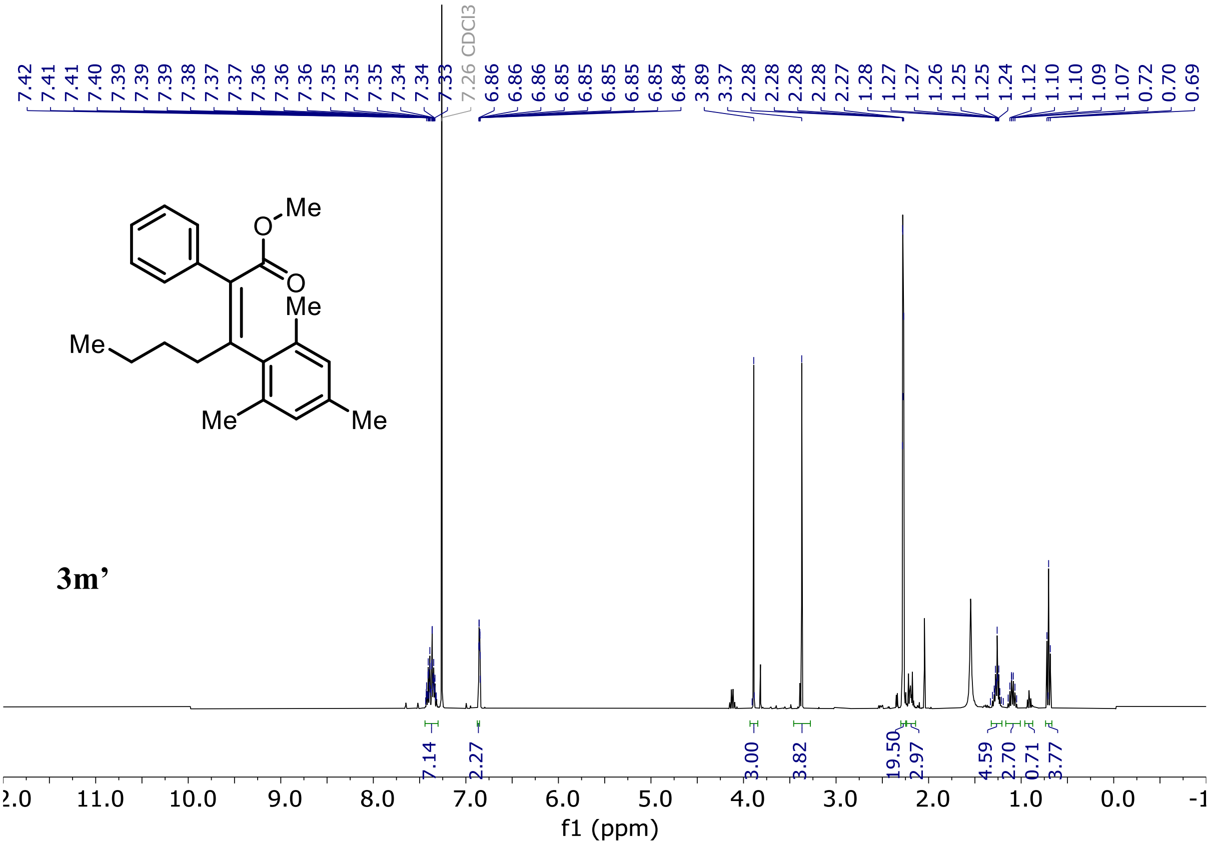
7.42 7.41 7.41 7.40 7.39 7.39 7.39 7.38 7.37 7.37 7.36 7.36 7.36 7.35 7.35 7.35 7.34 7.34 7.33
7.26 CDCl3
6.86 6.86 6.86 6.85 6.85 6.85 6.85 6.85 6.84
3.89 3.37 2.28 2.28 2.28 2.27 1.28 1.27 1.27 1.26 1.25 1.25 1.24 1.12 1.10 1.10 1.09 1.07 0.72 0.70 0.69
O
Me
O
Me
Me
Me
Me
3m’
7.14
2.27
3.00
3.82
19.50
2.97
4.59
2.70
0.71
3.77
f1 (ppm)

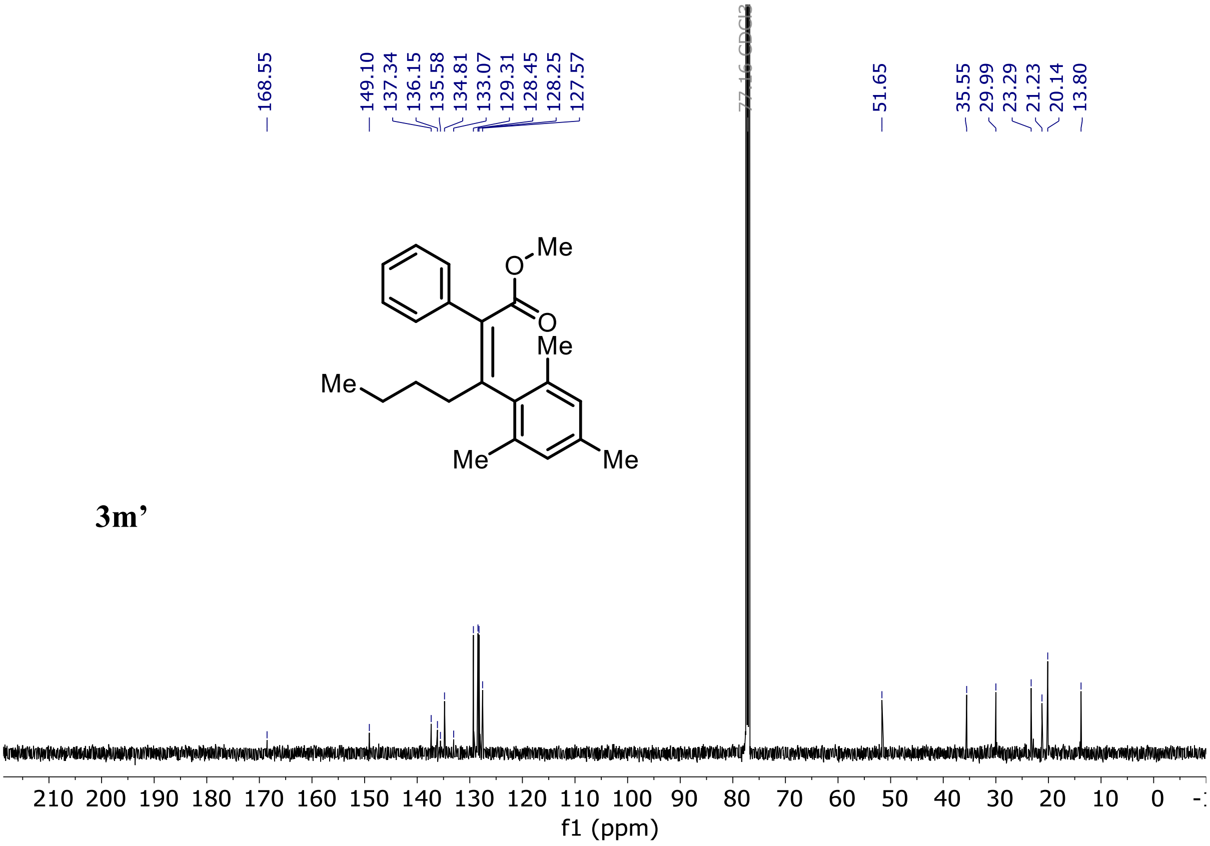
168.55
149.10 137.34 136.15 135.58 134.81 133.07 129.31 128.45 128.25 127.57
77.16 CDCl3
51.65
35.55 29.99 23.29 21.23 20.14 13.80
O
Me
O
Me
Me
Me
Me
3m’
f1 (ppm)

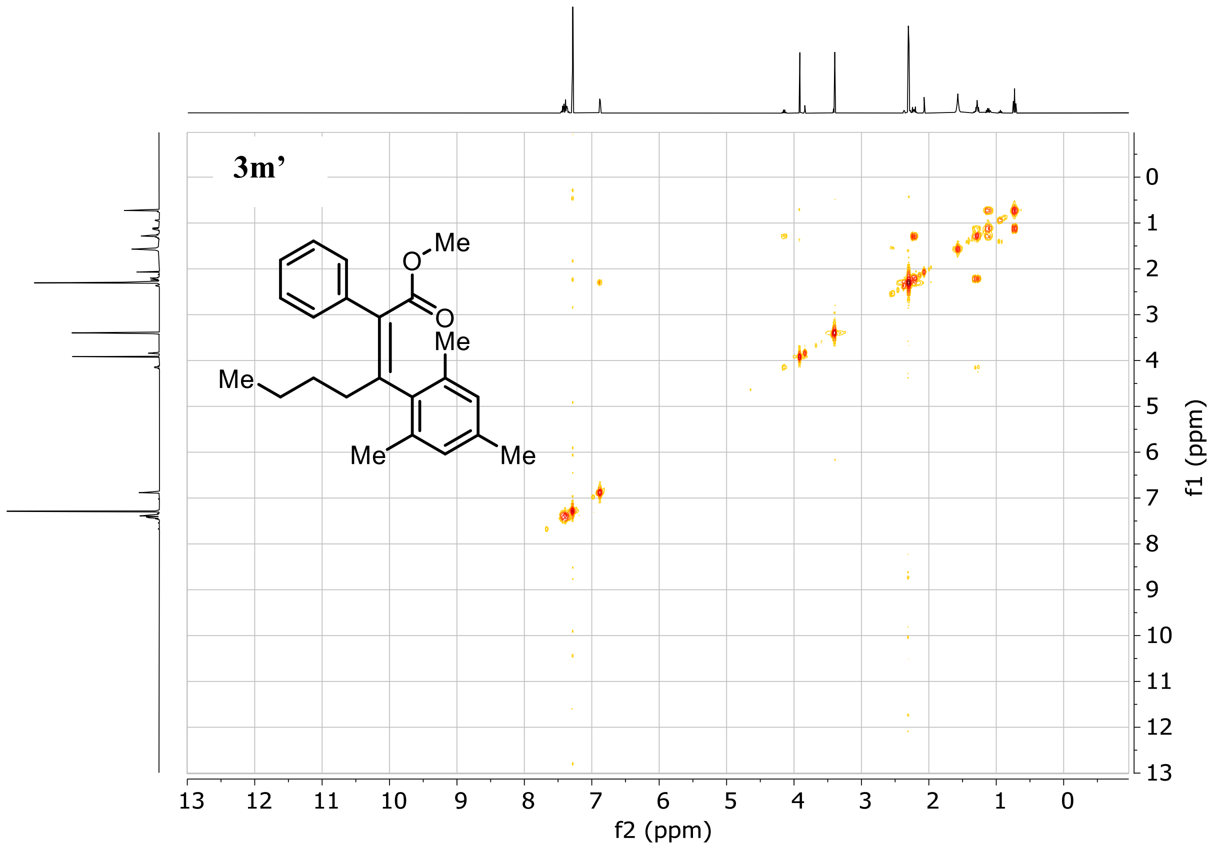
3m’
O
Me
O
Me
Me
Me
Me
f1 (ppm)
f2 (ppm)

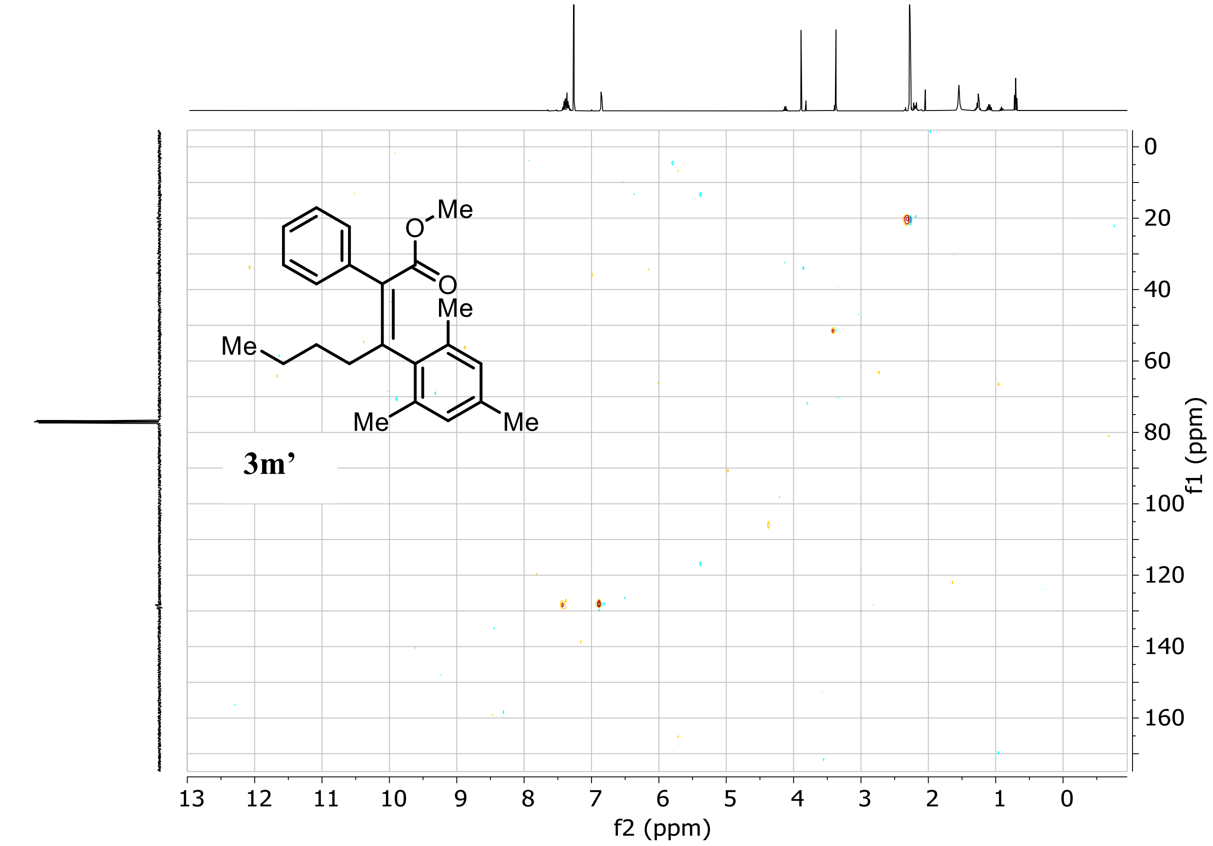
3m’
O
Me
O
Me
Me
Me
Me
f1 (ppm)
f2 (ppm)

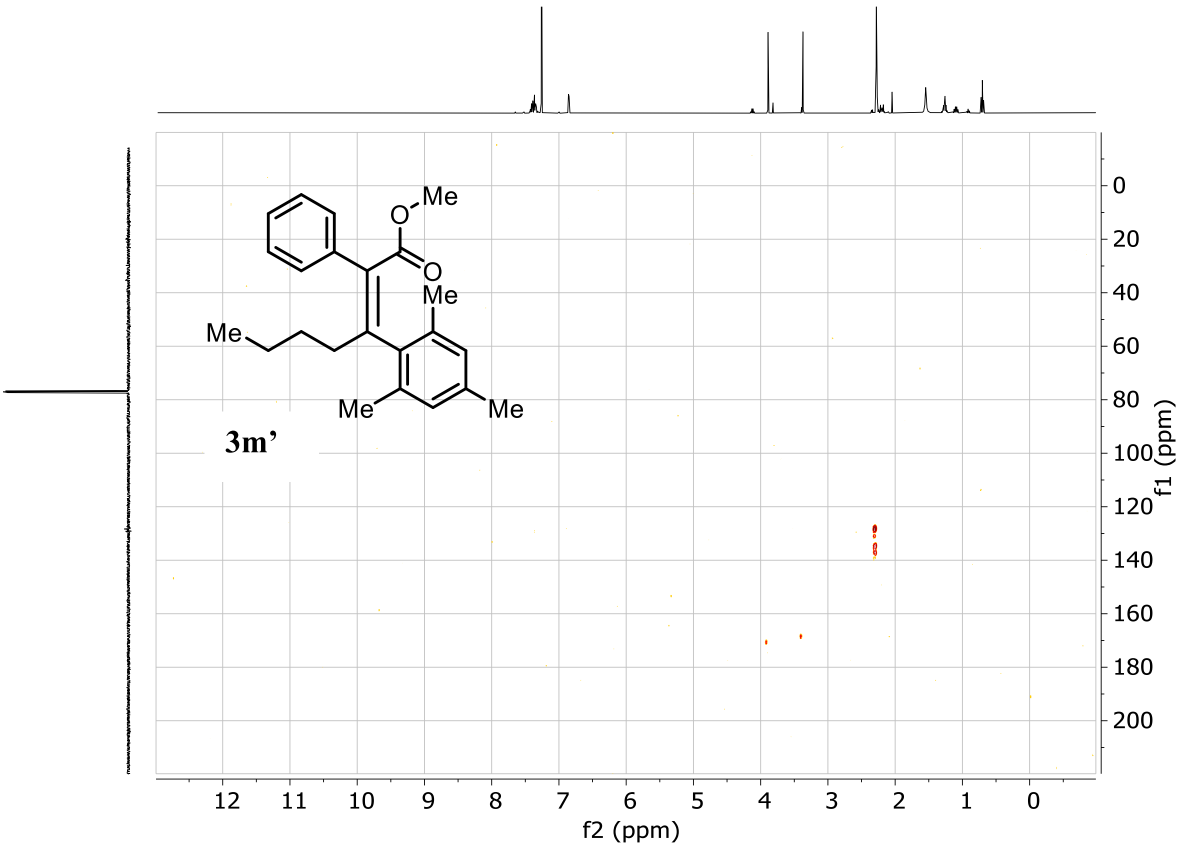

Me
O
O
Me
Me
Me
Me
3m’
12 11 10 9 8 7 6 5 4 3 2 1 0
f2 (ppm)
0 20 40 60 80 100 120 140 160 180 200
f1 (ppm)

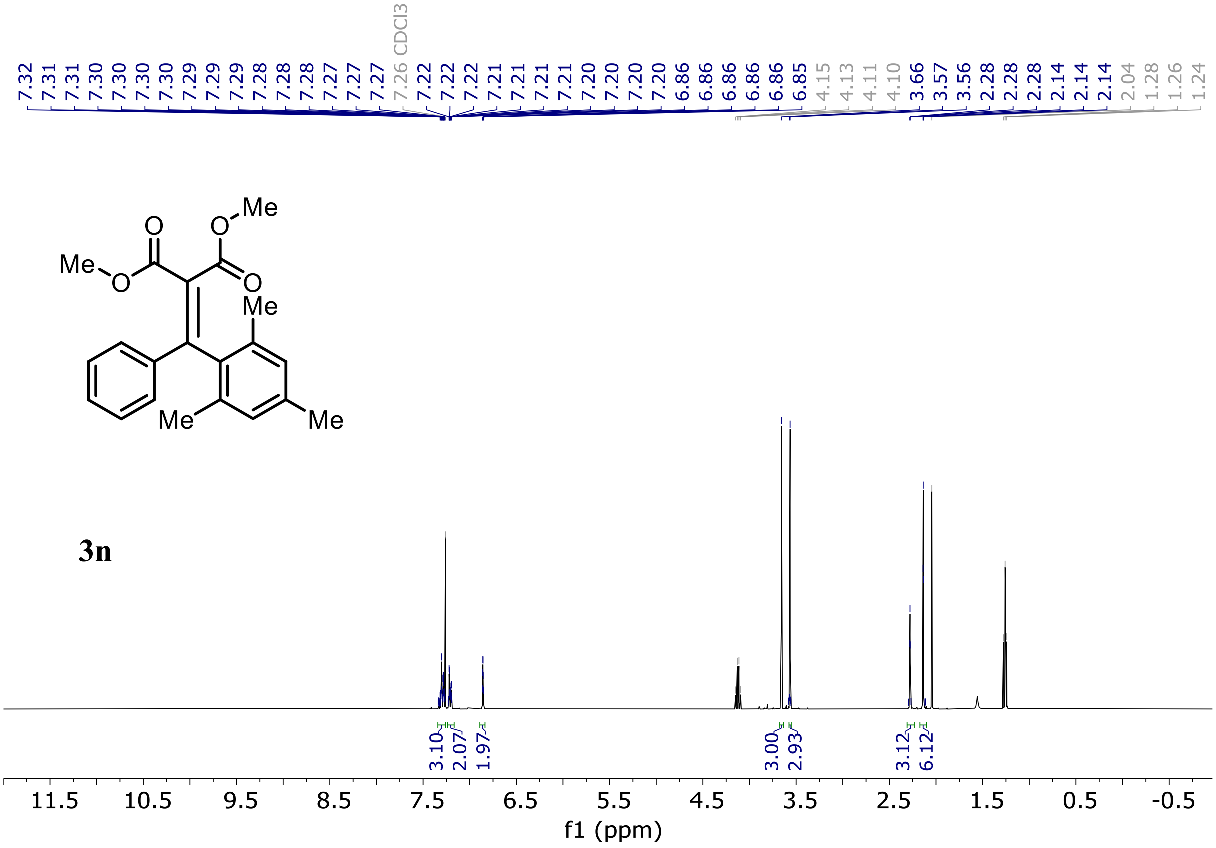

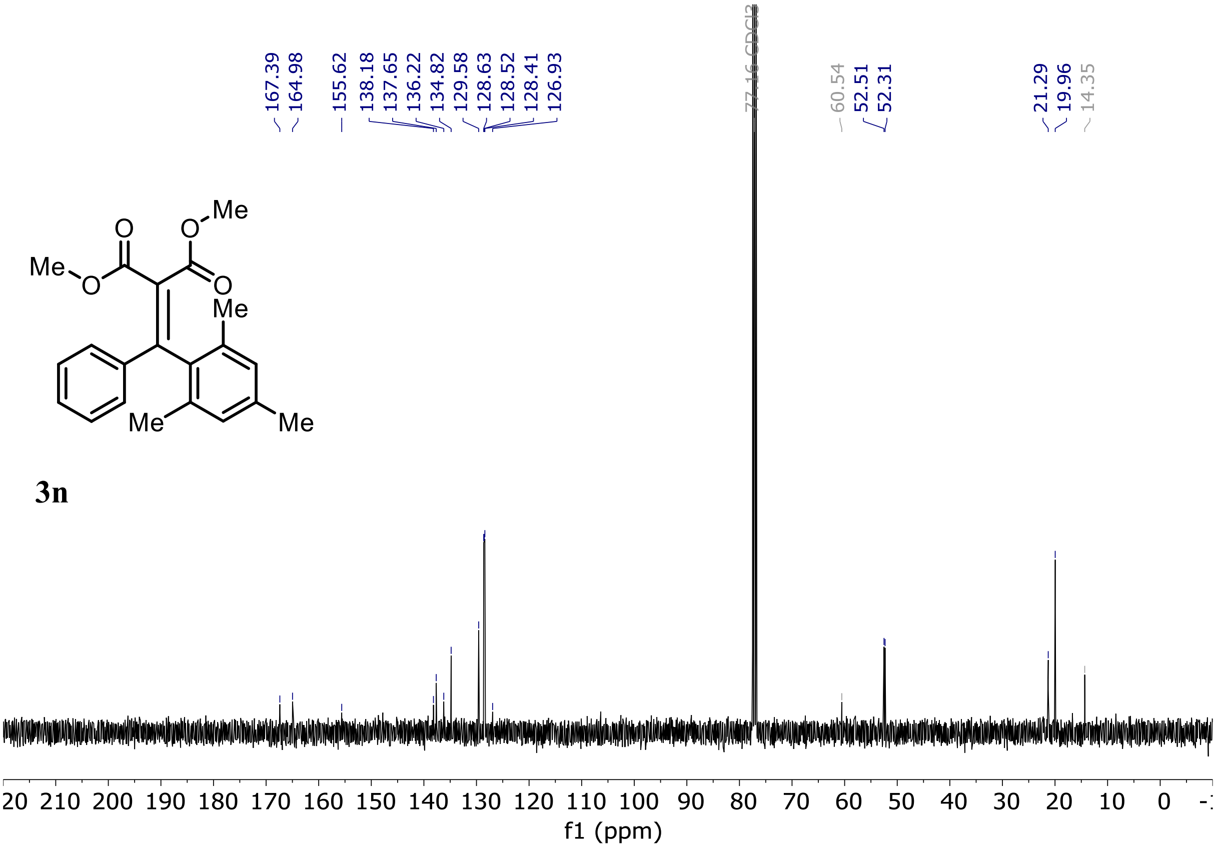

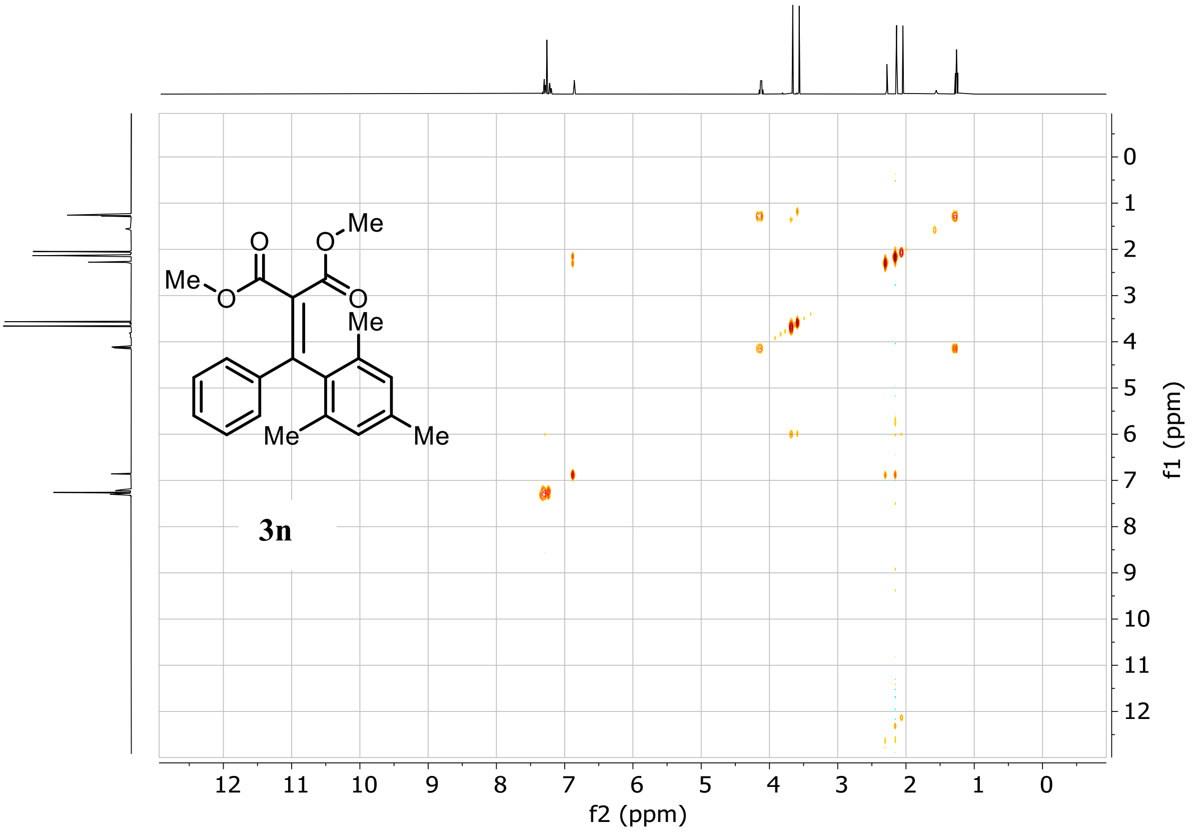
Me
O
O
Me
O
O
Me
Me
Me
3n
f2 (ppm)
f1 (ppm)

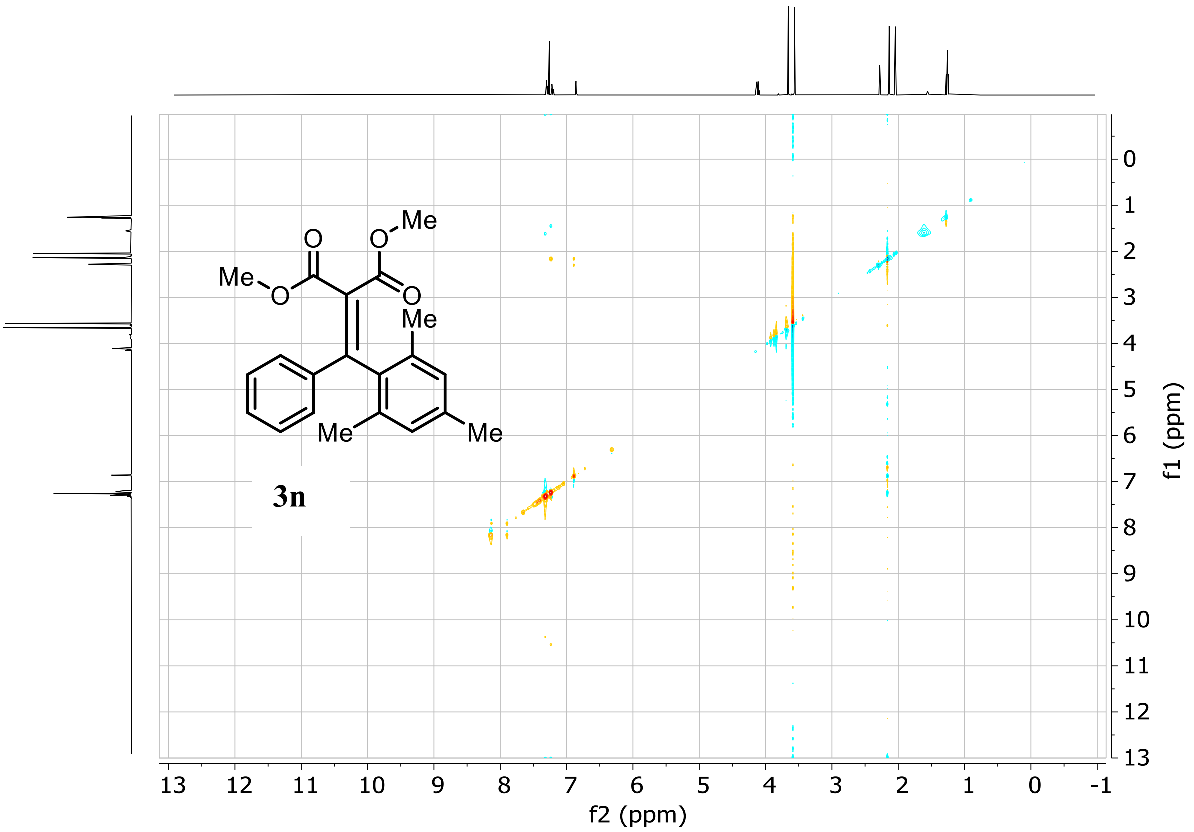
Me
O
O
Me
O
O
Me
Me
Me
3n
f2 (ppm)
f1 (ppm)

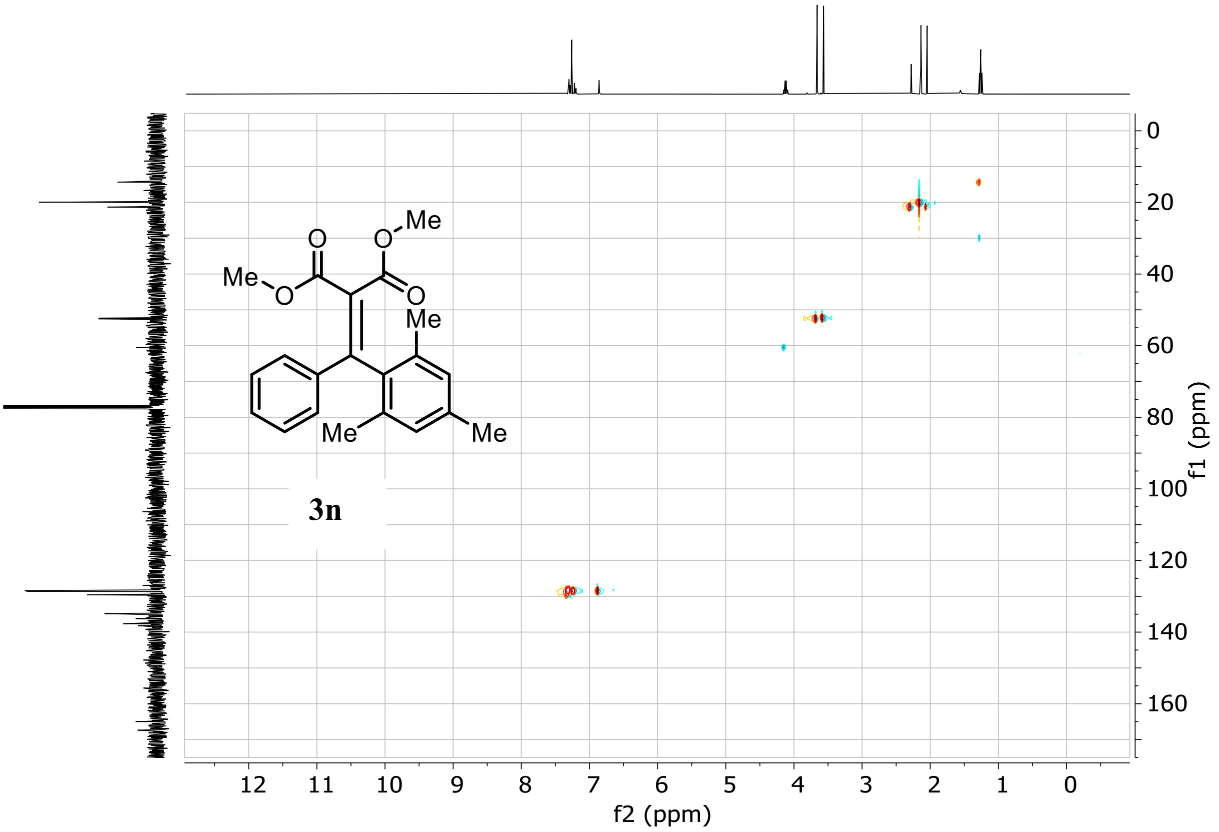

Me
O
O
Me
O
O
Me
Me
Me
3n
f2 (ppm)
f1 (ppm)

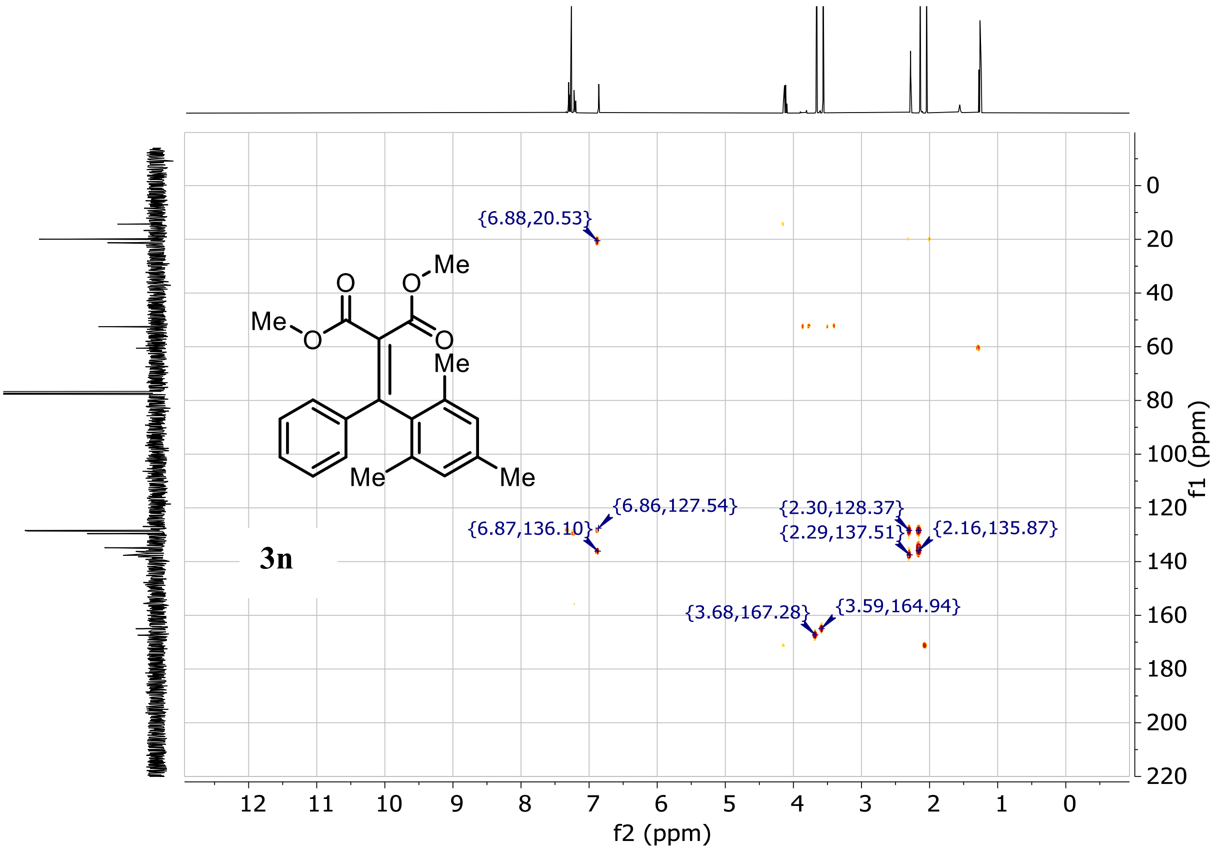

{6.88,20.53}
{6.86,127.54}
{6.87,136.10}
{2.30,128.37}
{2.29,137.51}
{2.16,135.87}
{3.68,167.28}
{3.59,164.94}
Me
O
O
Me
O
O
Me
Me
Me
3n
f2 (ppm)
f1 (ppm)

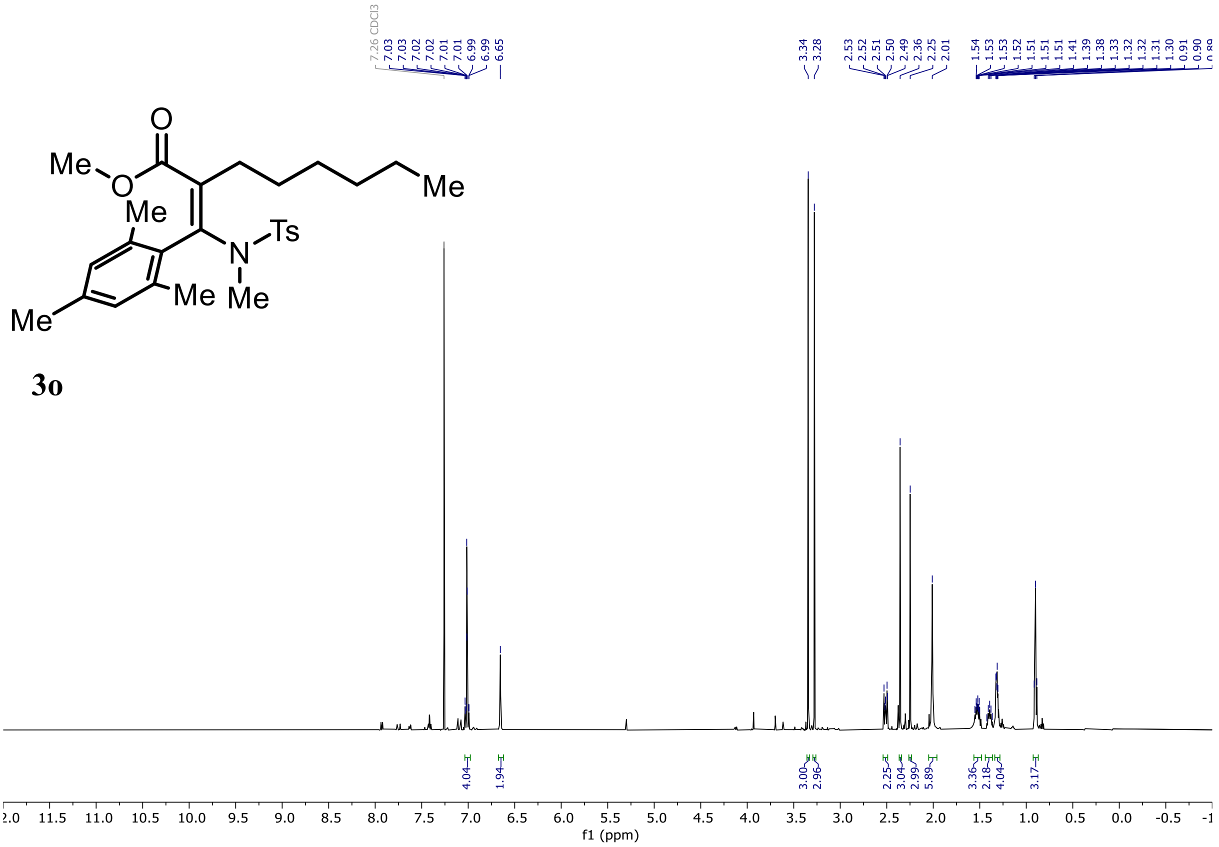

3o

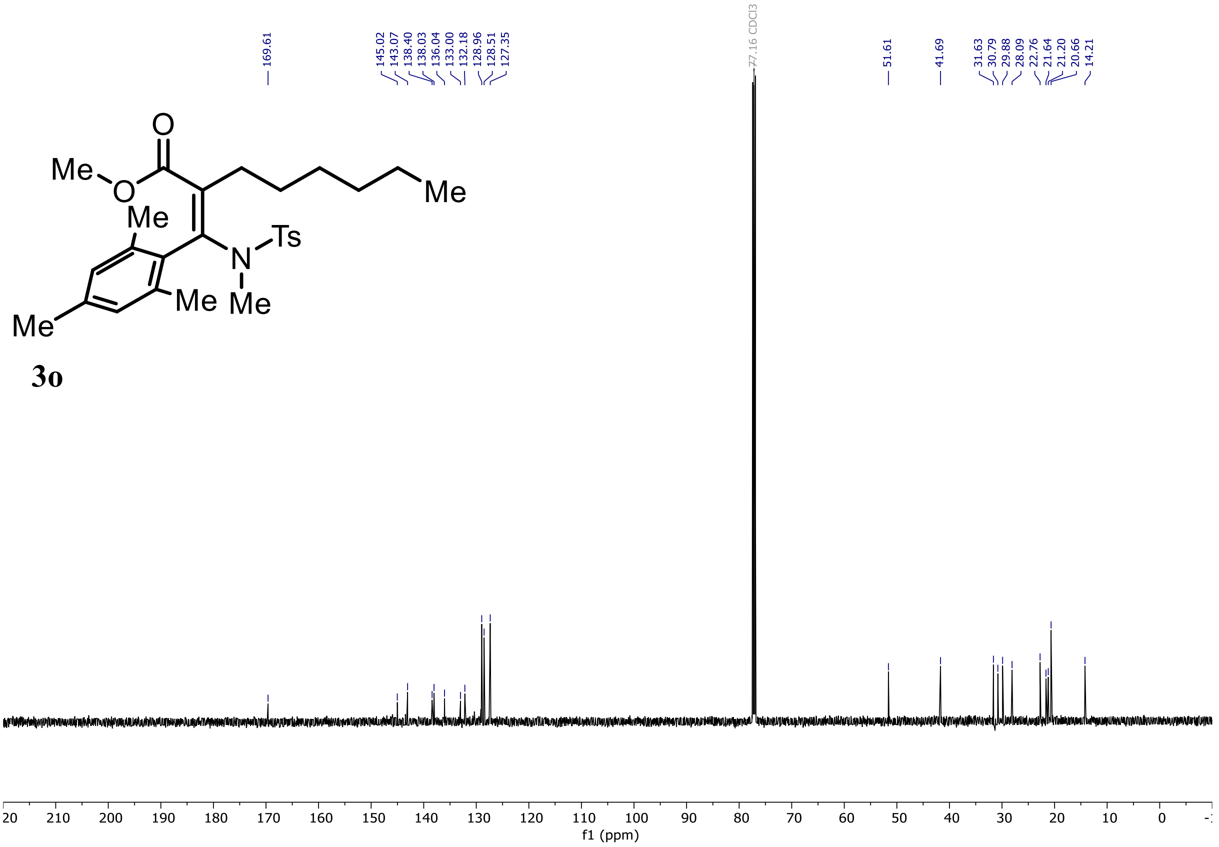

3o

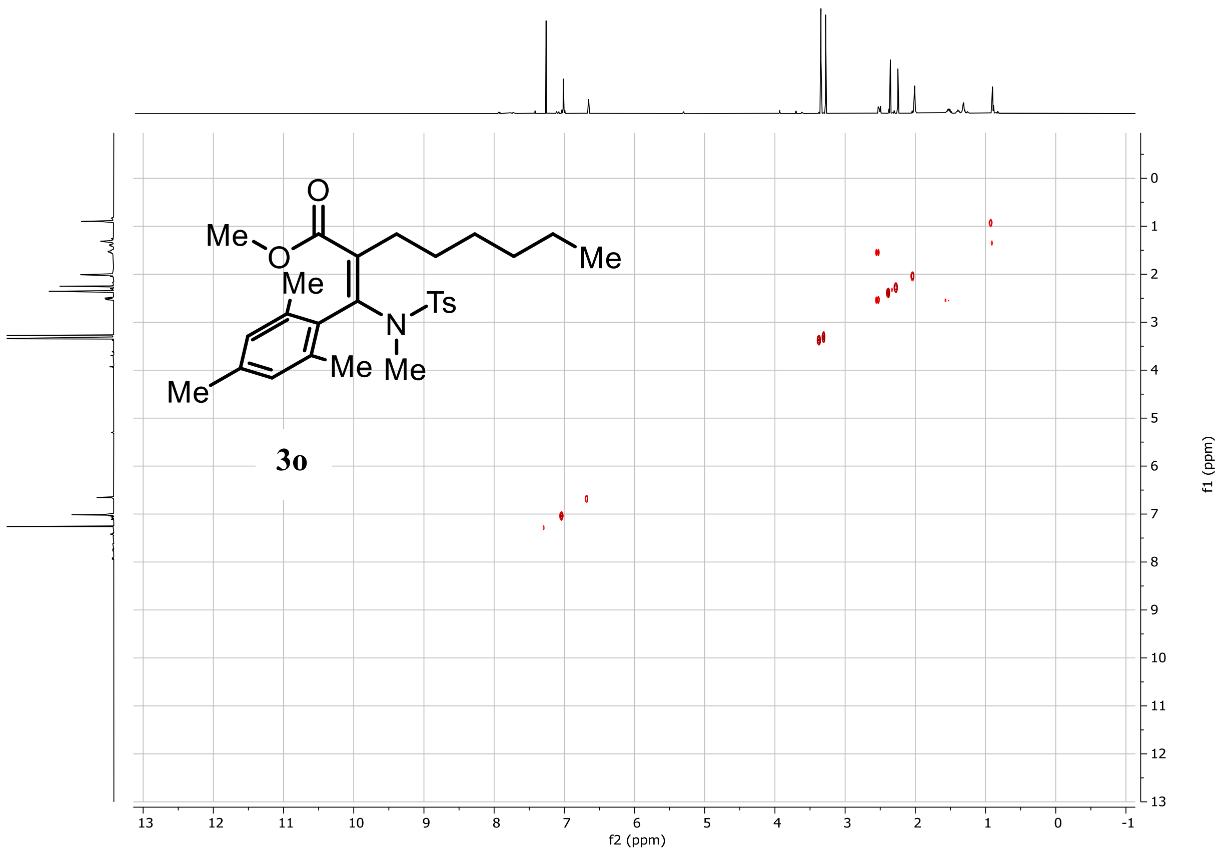
3o
f2 (ppm)
f1 (ppm)

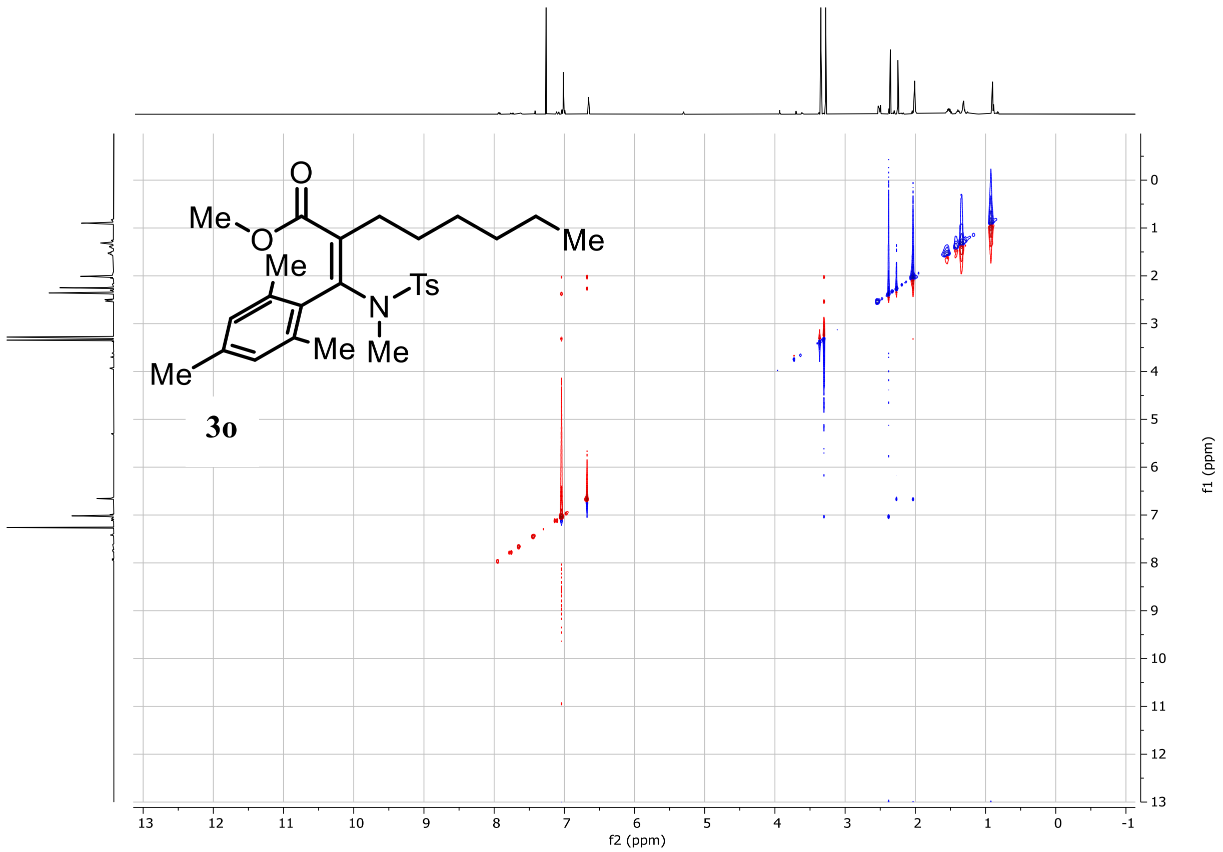
3o
f2 (ppm)
f1 (ppm)

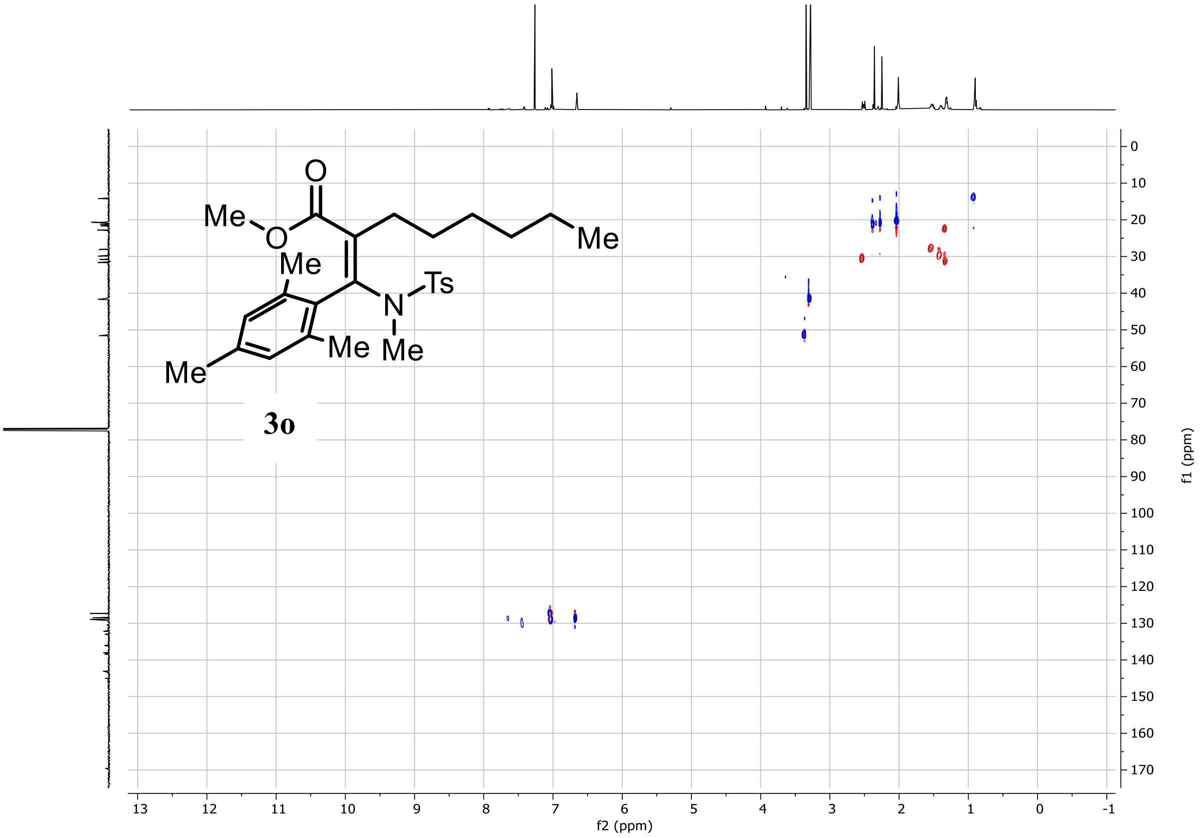
Me
O
O
Me
Me
Me
N
Ts
Me
Me
Me
3o
f2 (ppm)
f1 (ppm)

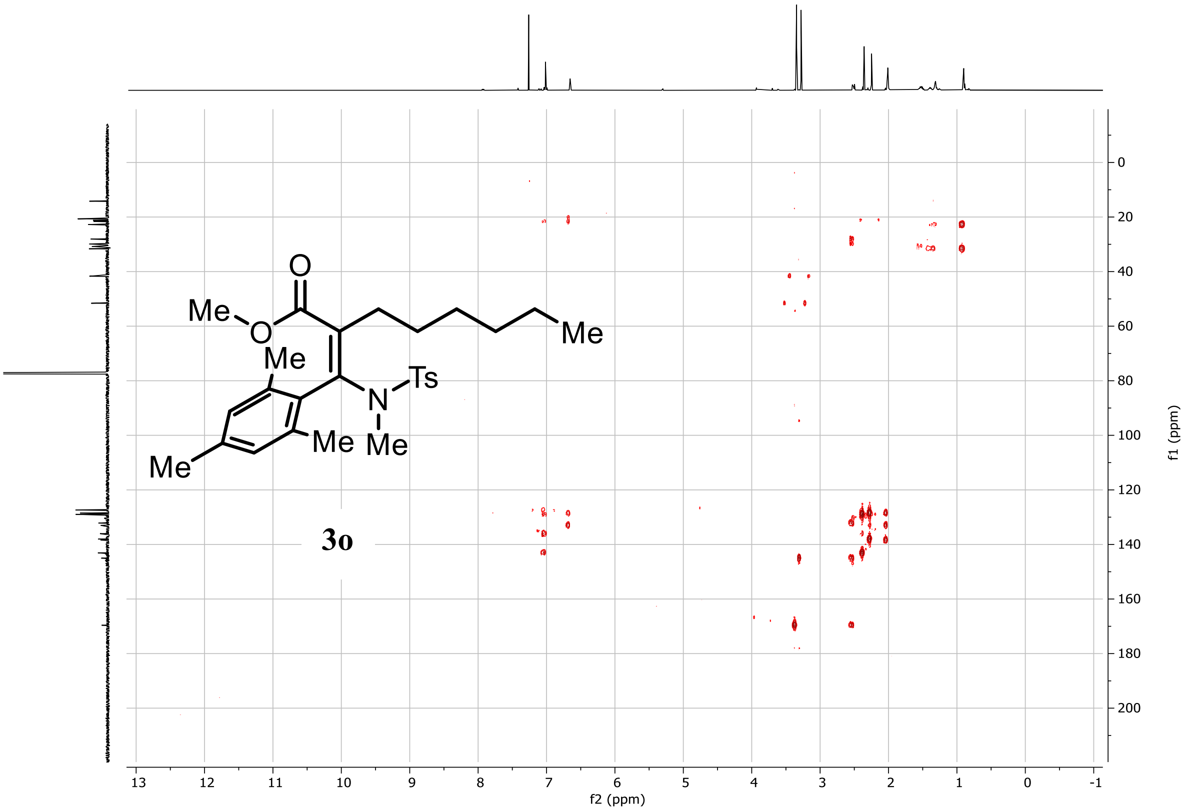
Me
O
O
Me
Me
Me
N
Ts
Me
Me
Me
3o
f2 (ppm)
f1 (ppm)

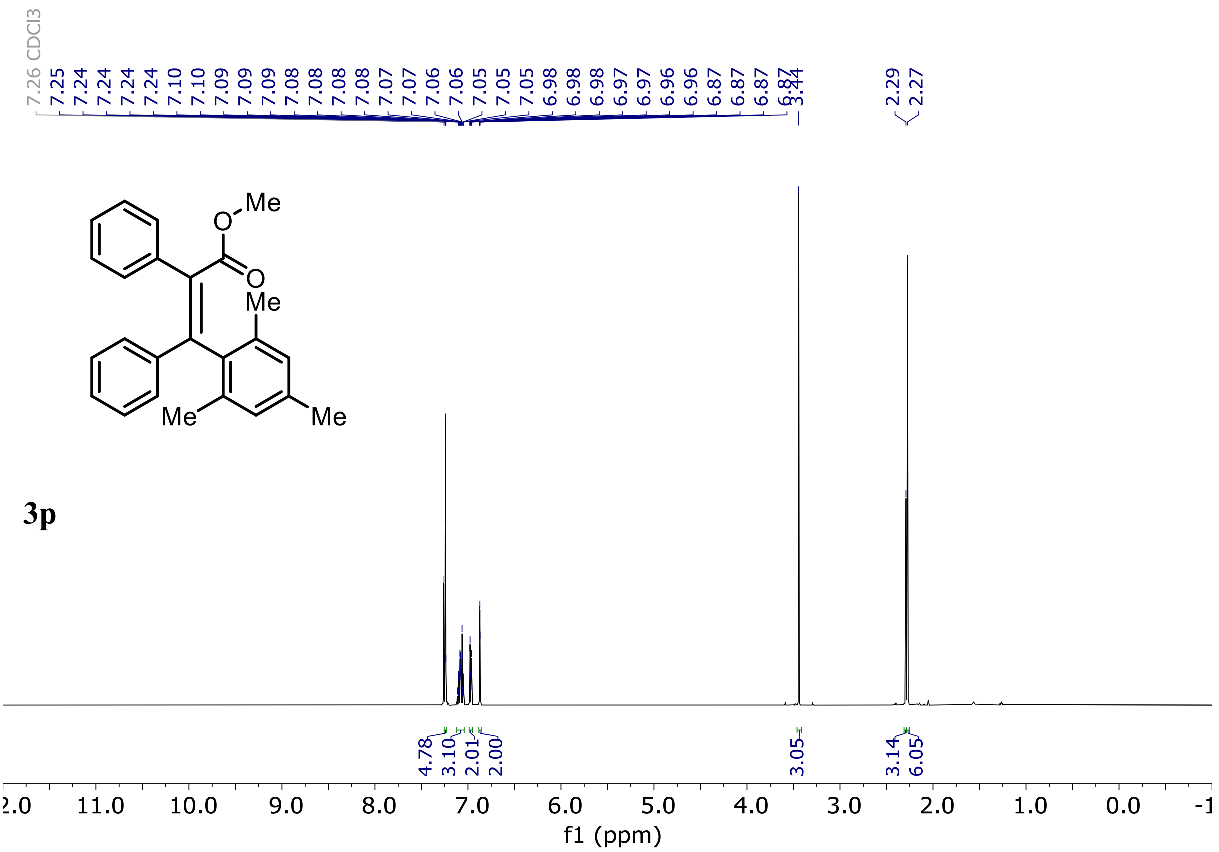

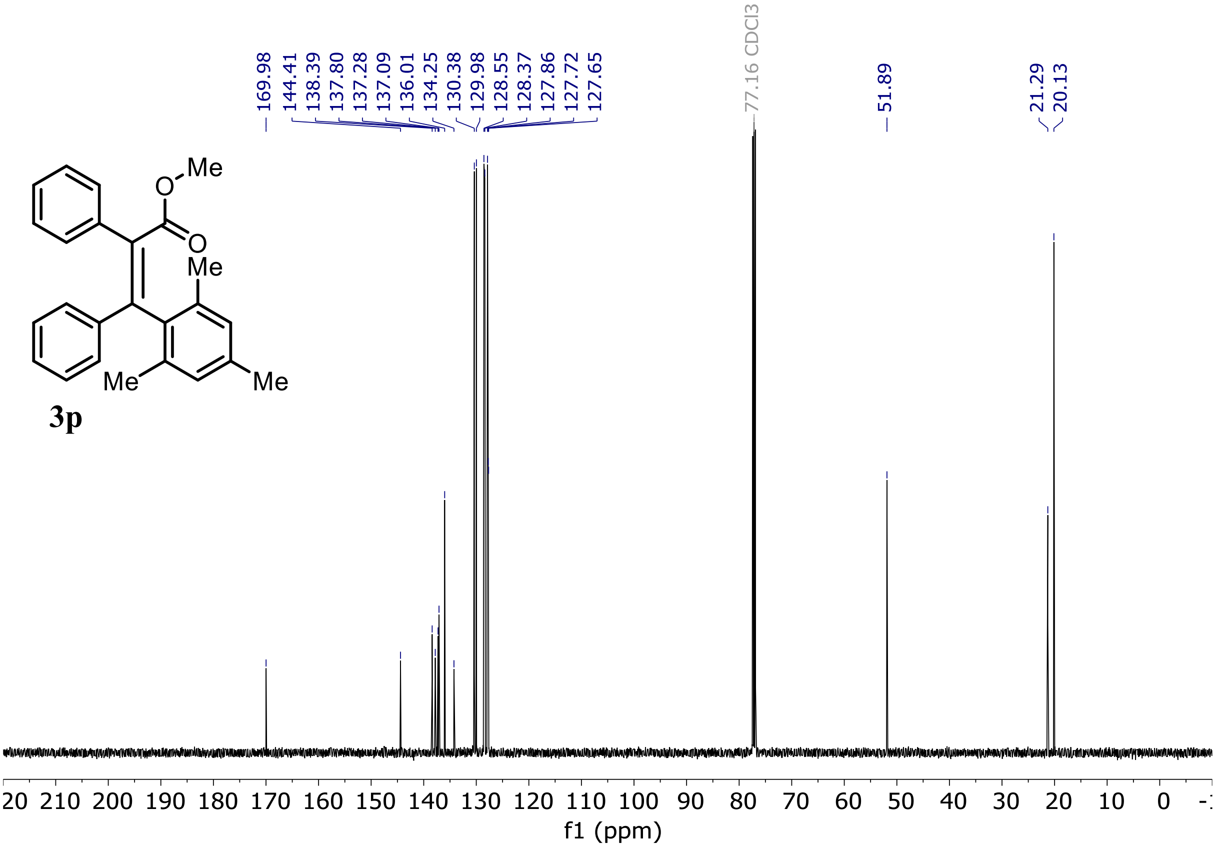

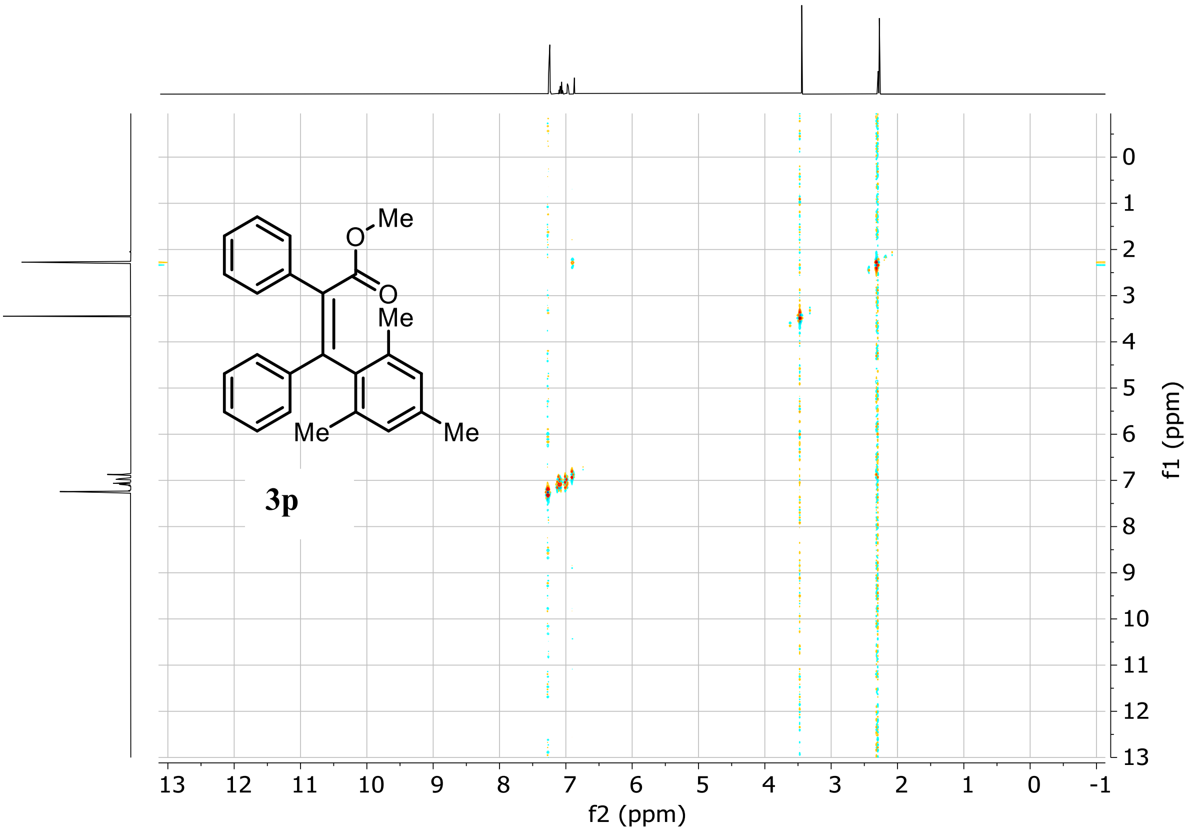
Me
O
O
Me
Me
Me
3p
f2 (ppm)
f1 (ppm)

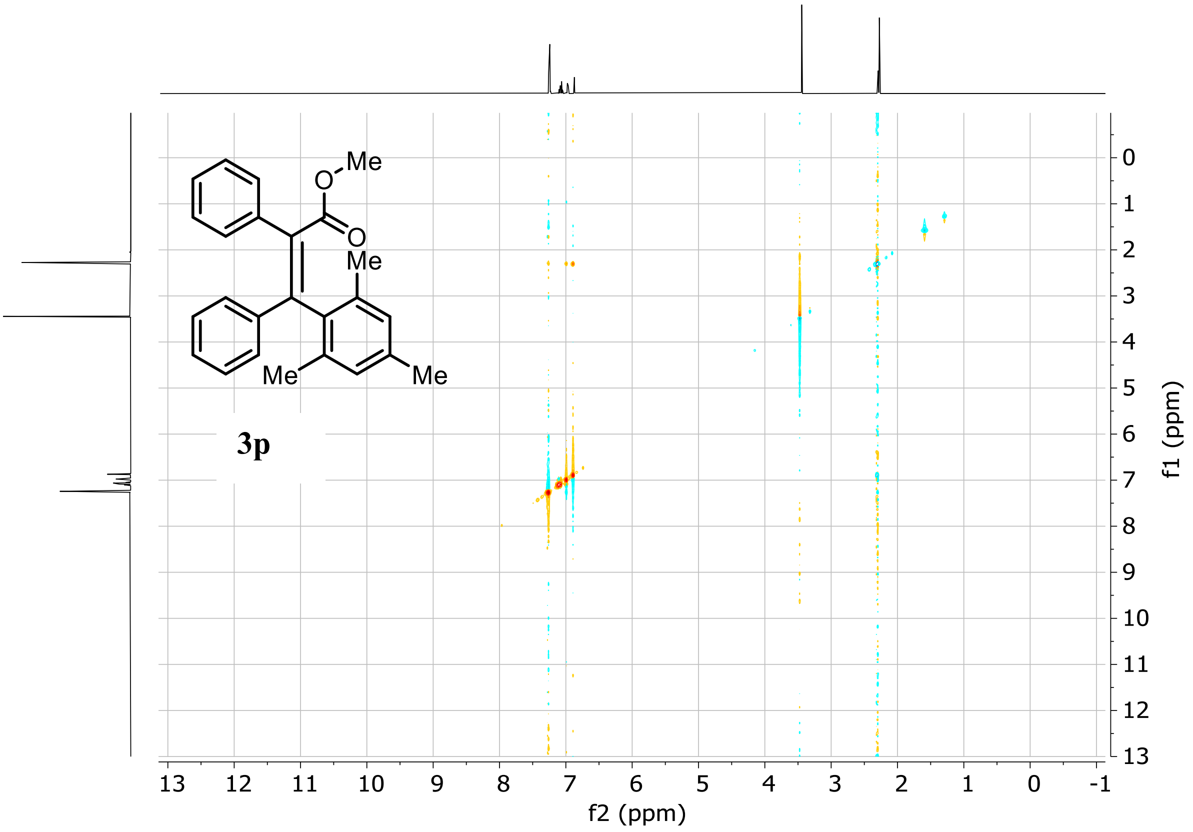
Me
O
O
Me
Me
Me
3p
f2 (ppm)
f1 (ppm)

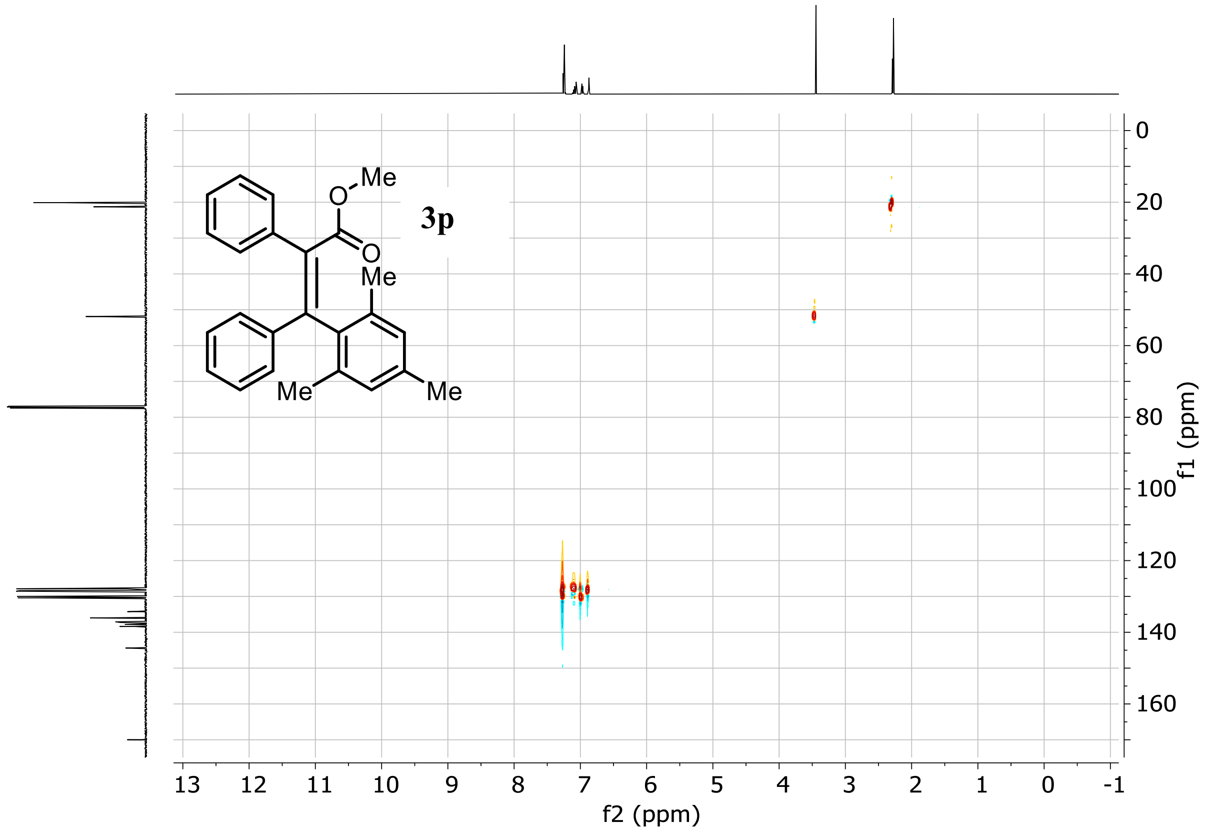

3p
Me
O
O
Me
Me
Me
f1 (ppm)
0
20
40
60
80
100
120
140
160
13
12
11
10
9
8
7
6
5
4
3
2
1
0
-1
f2 (ppm)

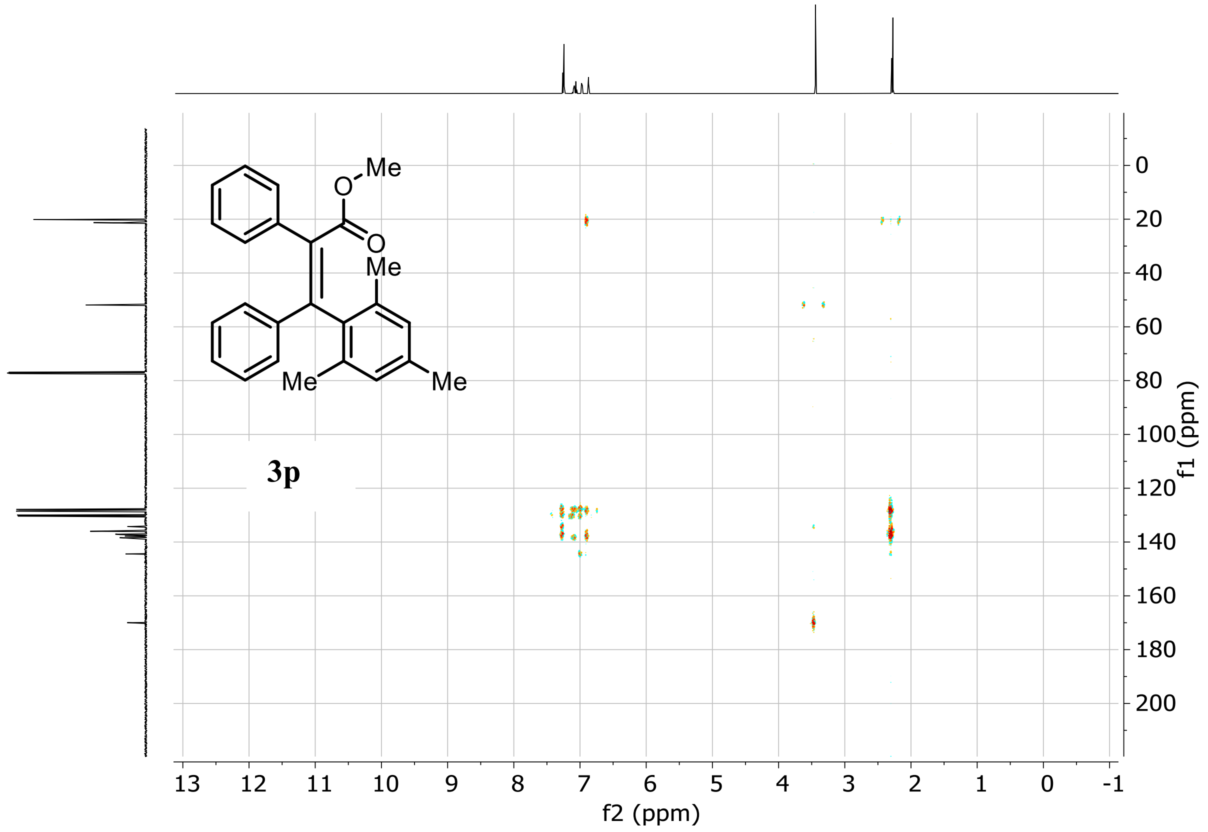

3p
Me
O
O
Me
Me
Me
f1 (ppm)
0
20
40
60
80
100
120
140
160
180
200
13
12
11
10
9
8
7
6
5
4
3
2
1
0
-1
f2 (ppm)

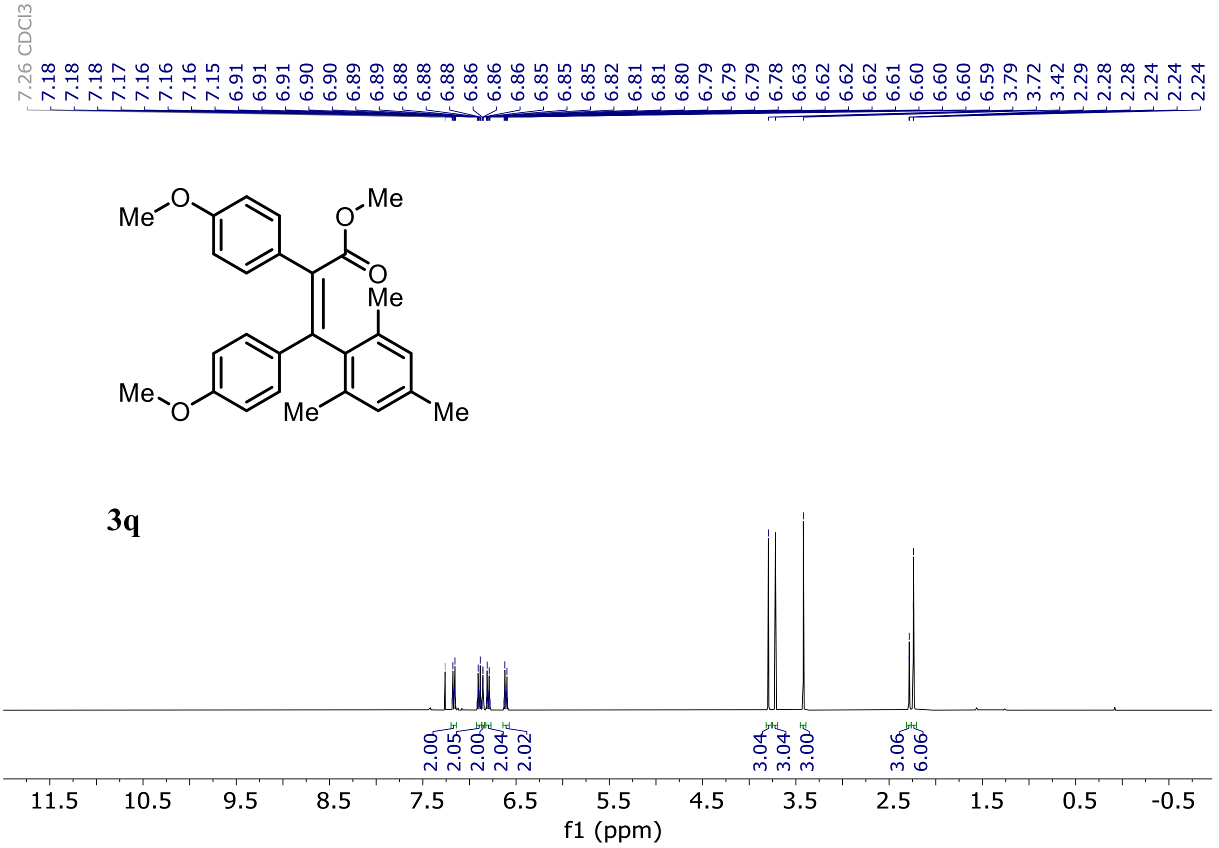
7.26 CDCl3
7.18
7.18
7.18
7.17
7.16
7.16
7.16
7.15
6.91
6.91
6.91
6.90
6.90
6.89
6.89
6.88
6.88
6.88
6.86
6.86
6.86
6.85
6.85
6.85
6.82
6.81
6.81
6.80
6.79
6.79
6.79
6.78
6.63
6.62
6.62
6.62
6.61
6.60
6.60
6.60
6.59
3.79
3.72
3.42
2.29
2.28
2.28
2.24
2.24
2.24
Me
O
Me
O
O
Me
Me
O
Me
Me
3q
2.00
2.05
2.00
2.04
2.02
3.04
3.04
3.00
3.06
6.06
11.5
10.5
9.5
8.5
7.5
6.5
5.5
4.5
3.5
2.5
1.5
0.5
-0.5
f1 (ppm)

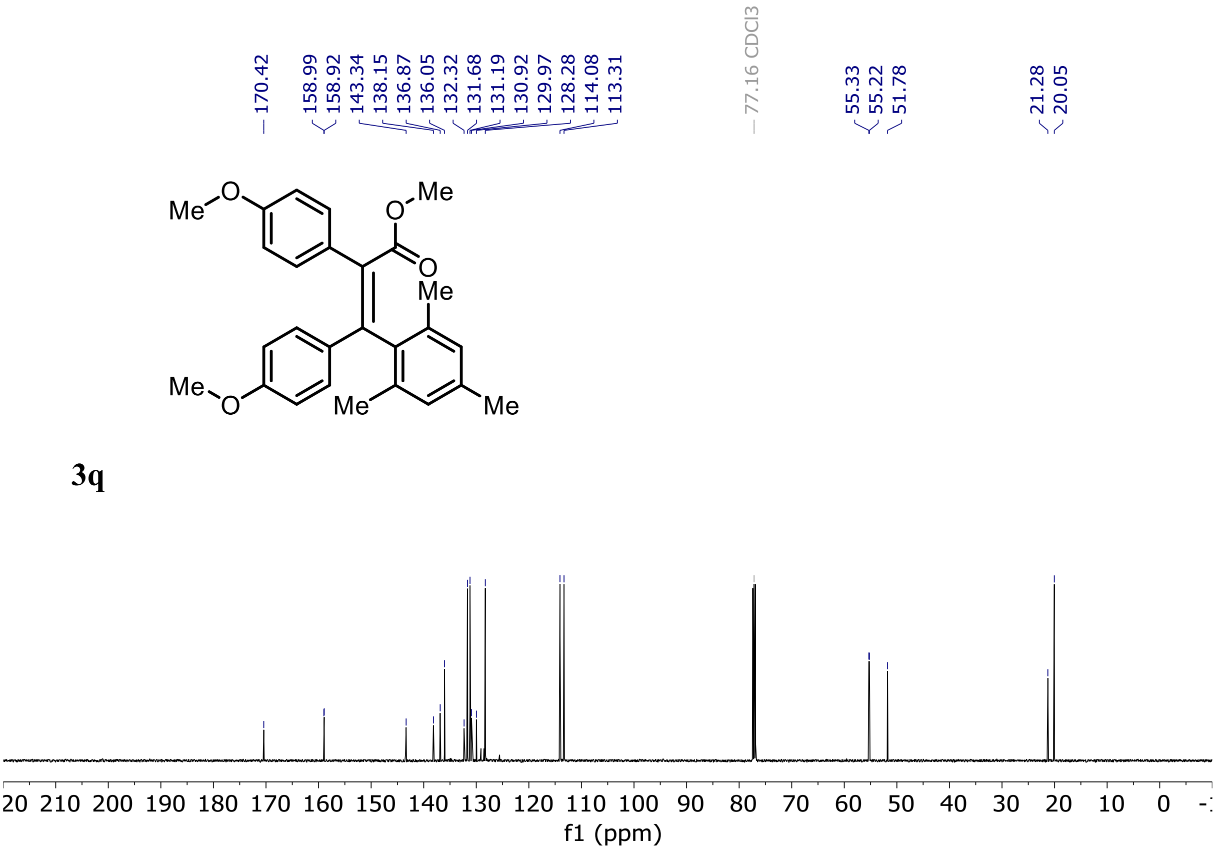
170.42
158.99
158.92
143.34
138.15
136.87
136.05
132.32
131.68
131.19
130.92
129.97
128.28
114.08
113.31
77.16 CDCl3
55.33
55.22
51.78
21.28
20.05
Me
O
Me
O
O
Me
Me
O
Me
Me
3q
20
210
200
190
180
170
160
150
140
130
120
110
100
90
80
70
60
50
40
30
20
10
0
f1 (ppm)

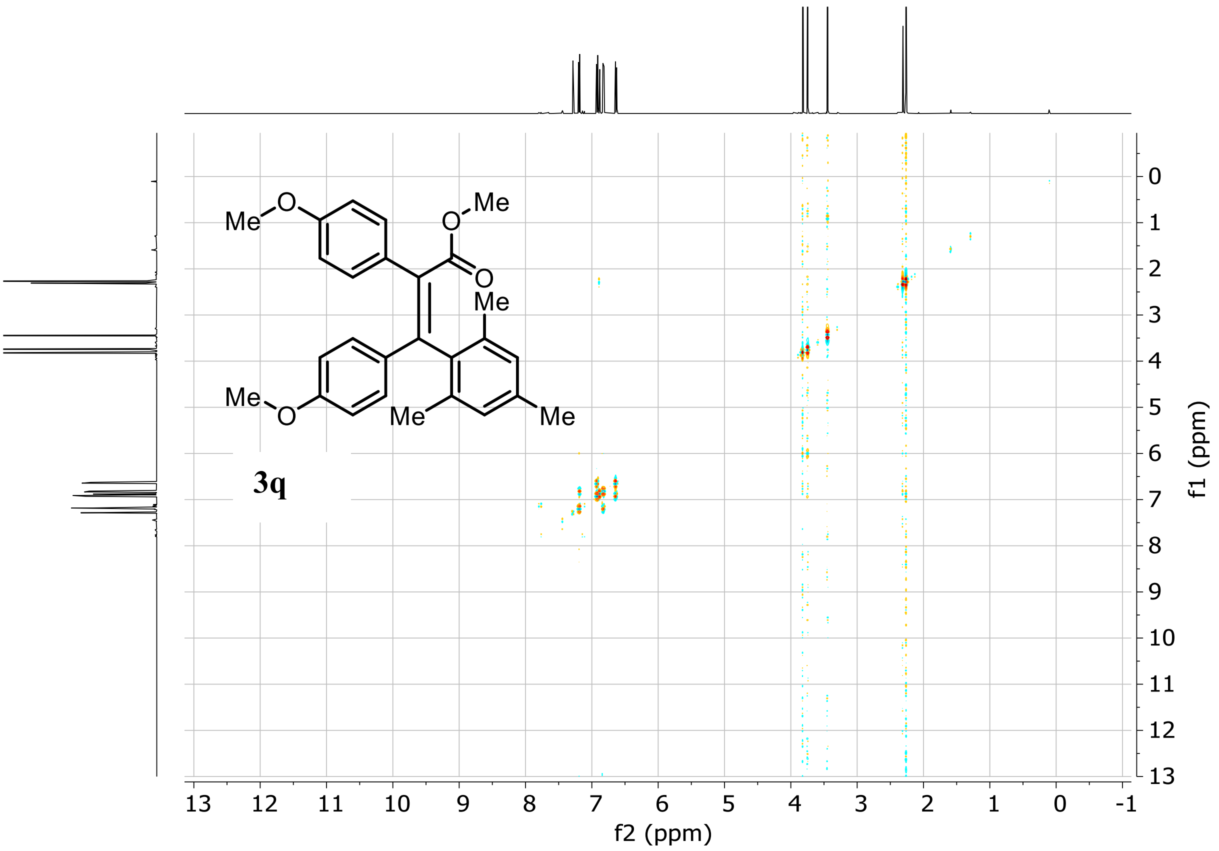
Me
O
O
Me
O
Me
Me
O
Me
Me
3q
f1 (ppm)
f2 (ppm)

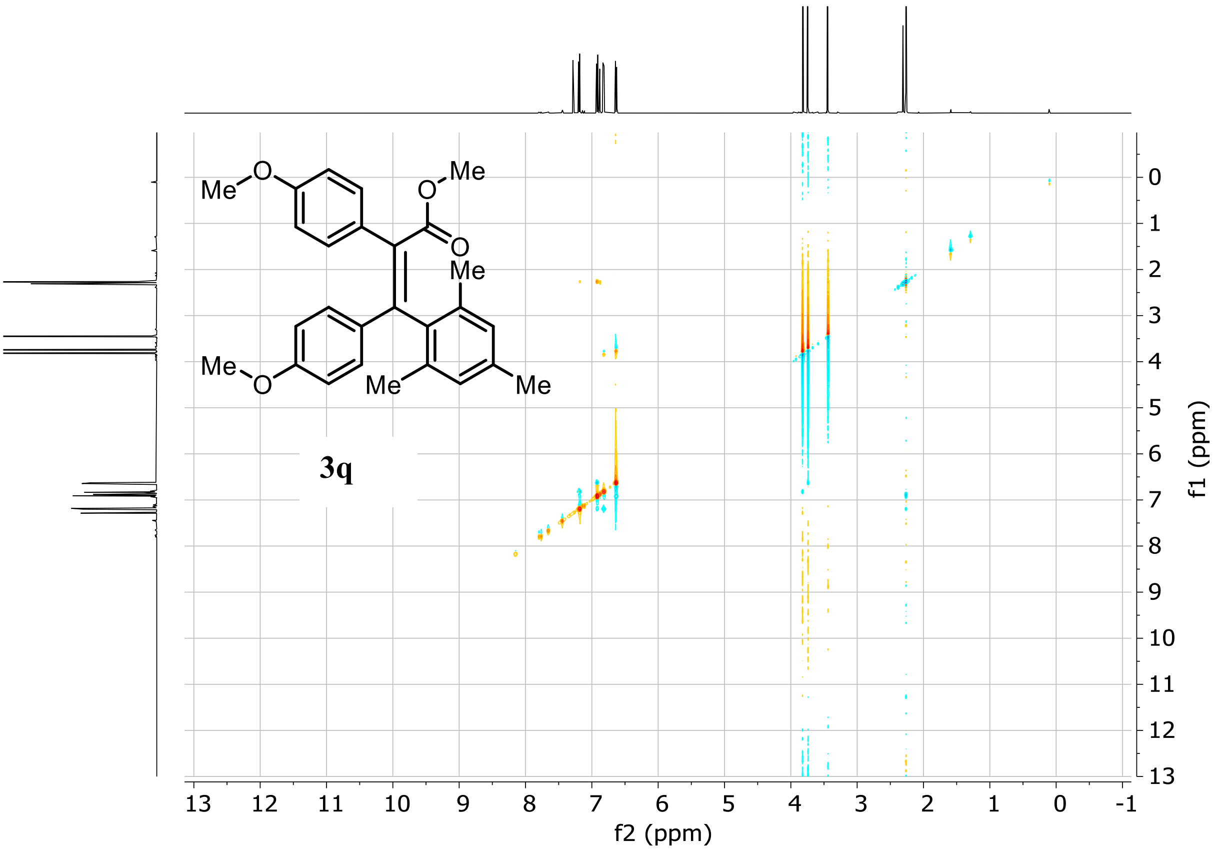
Me
O
O
Me
O
Me
Me
O
Me
Me
3q
f1 (ppm)
f2 (ppm)

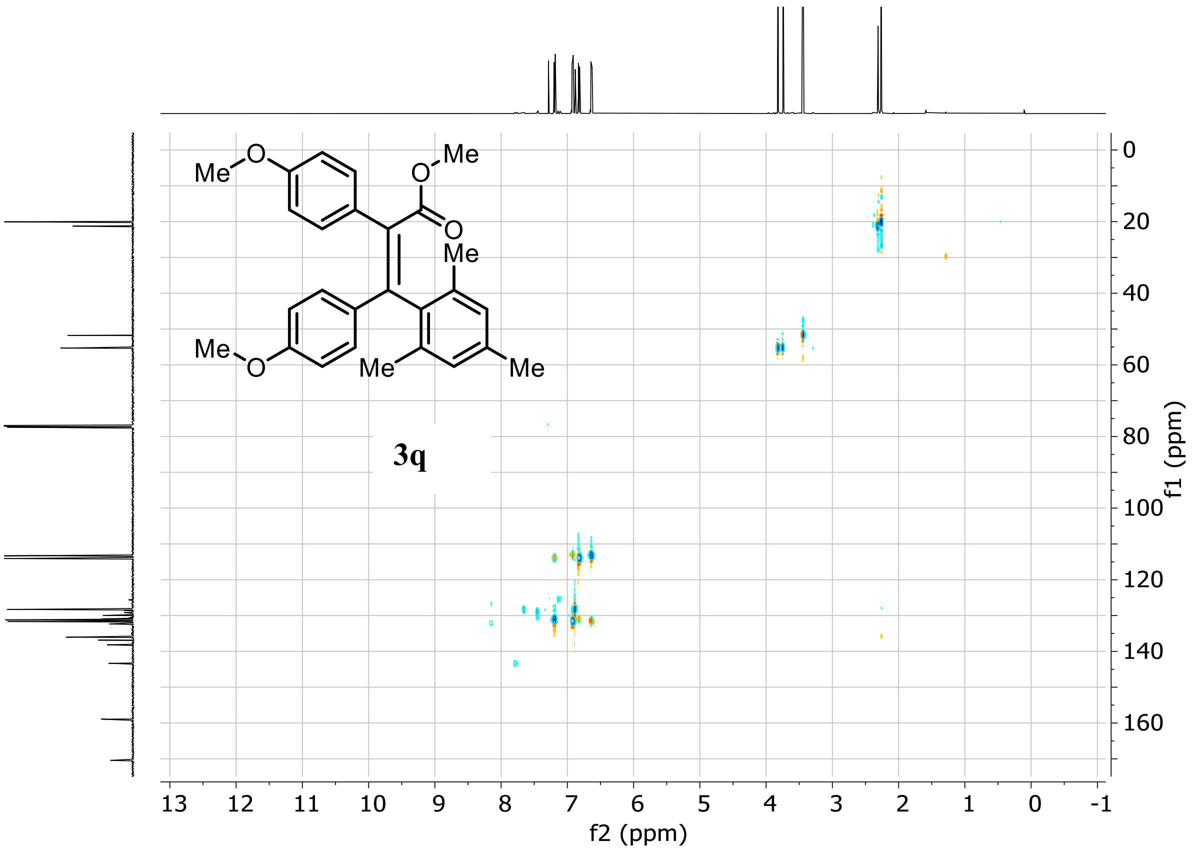
Me
O
O
Me
O
Me
Me
O
Me
Me
3q
f1 (ppm)
f2 (ppm)

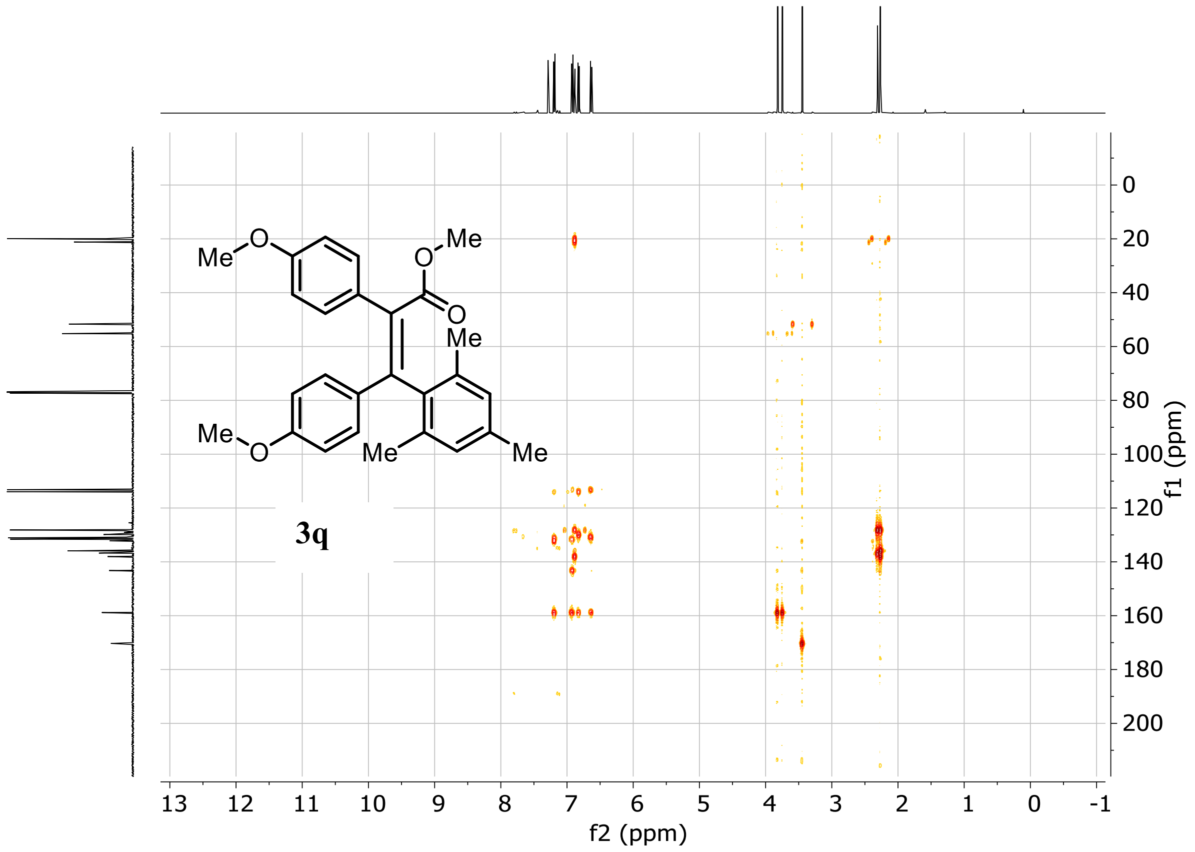
Me
O
O
Me
O
Me
Me
O
Me
Me
3q
f1 (ppm)
f2 (ppm)

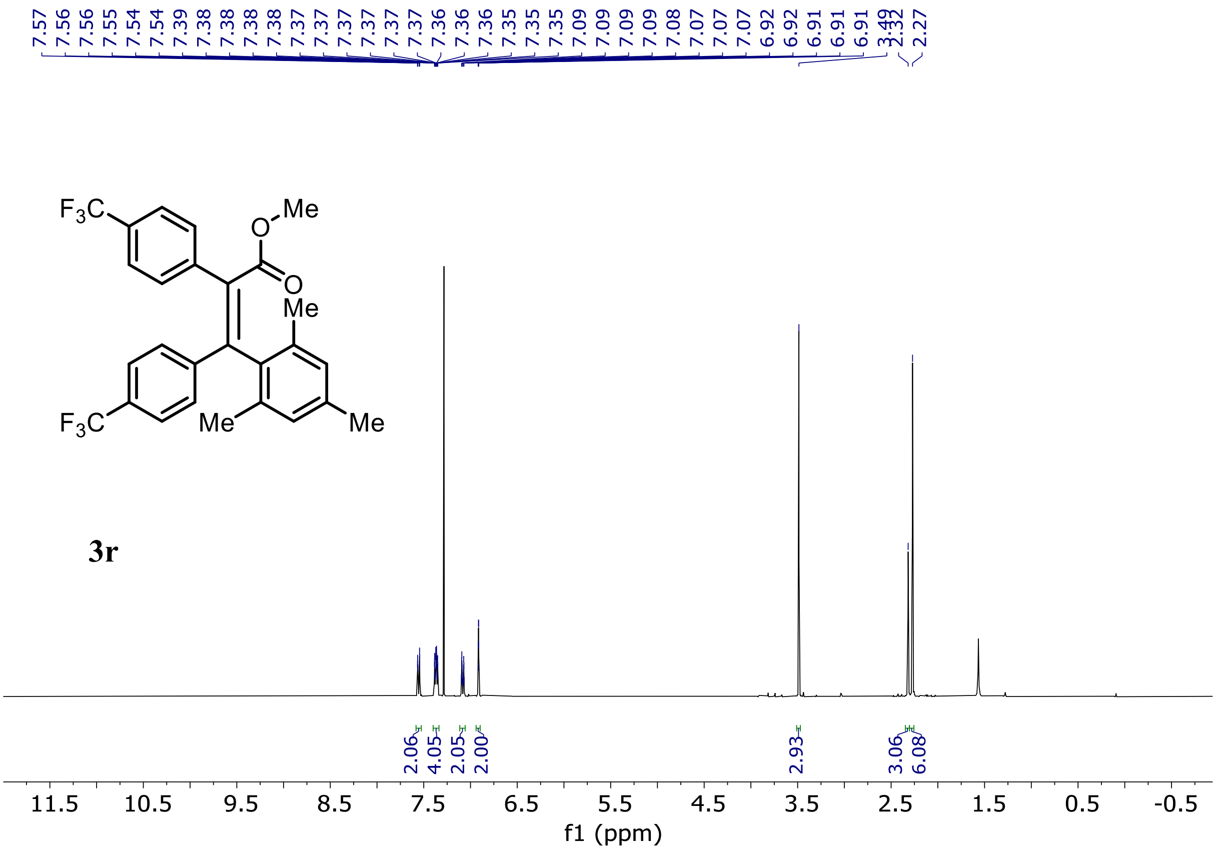
7.57
7.56
7.56
7.55
7.54
7.54
7.39
7.38
7.38
7.38
7.38
7.37
7.37
7.37
7.37
7.37
7.36
7.36
7.36
7.35
7.35
7.35
7.09
7.09
7.09
7.08
7.07
7.07
7.07
6.92
6.91
6.91
6.91
3.49
2.32
2.27
F3C
O
Me
O
Me
F3C
Me
Me
3r
2.06
4.05
2.05
2.00
2.93
3.06
6.08
11.5 10.5 9.5 8.5 7.5 6.5 5.5 4.5 3.5 2.5 1.5 0.5 -0.5
f1 (ppm)

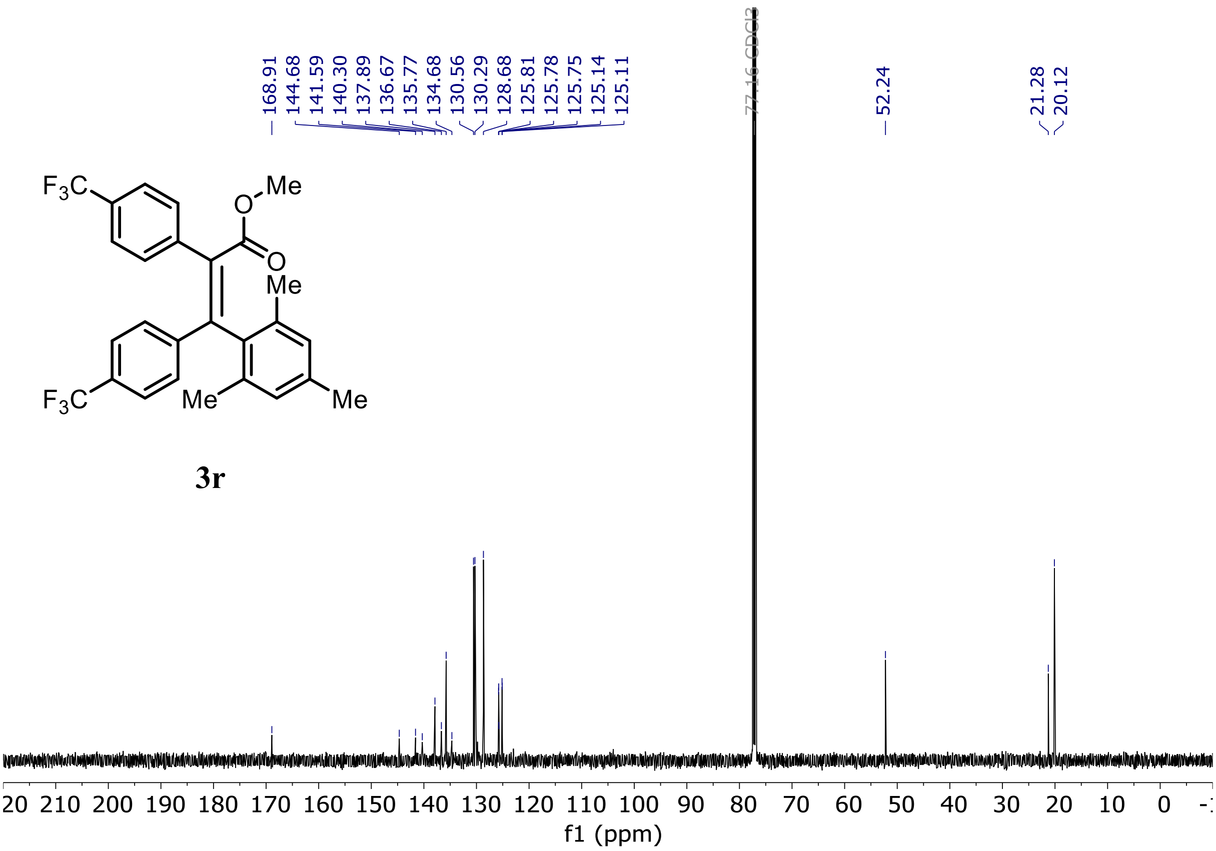
168.91
144.68
141.59
140.30
137.89
136.67
135.77
134.68
130.56
130.29
128.68
125.81
125.78
125.75
125.14
125.11
77.16 CDCl3
52.24
21.28
20.12
F3C
O
Me
O
Me
F3C
Me
Me
3r
20 210 200 190 180 170 160 150 140 130 120 110 100 90 80 70 60 50 40 30 20 10 0
f1 (ppm)

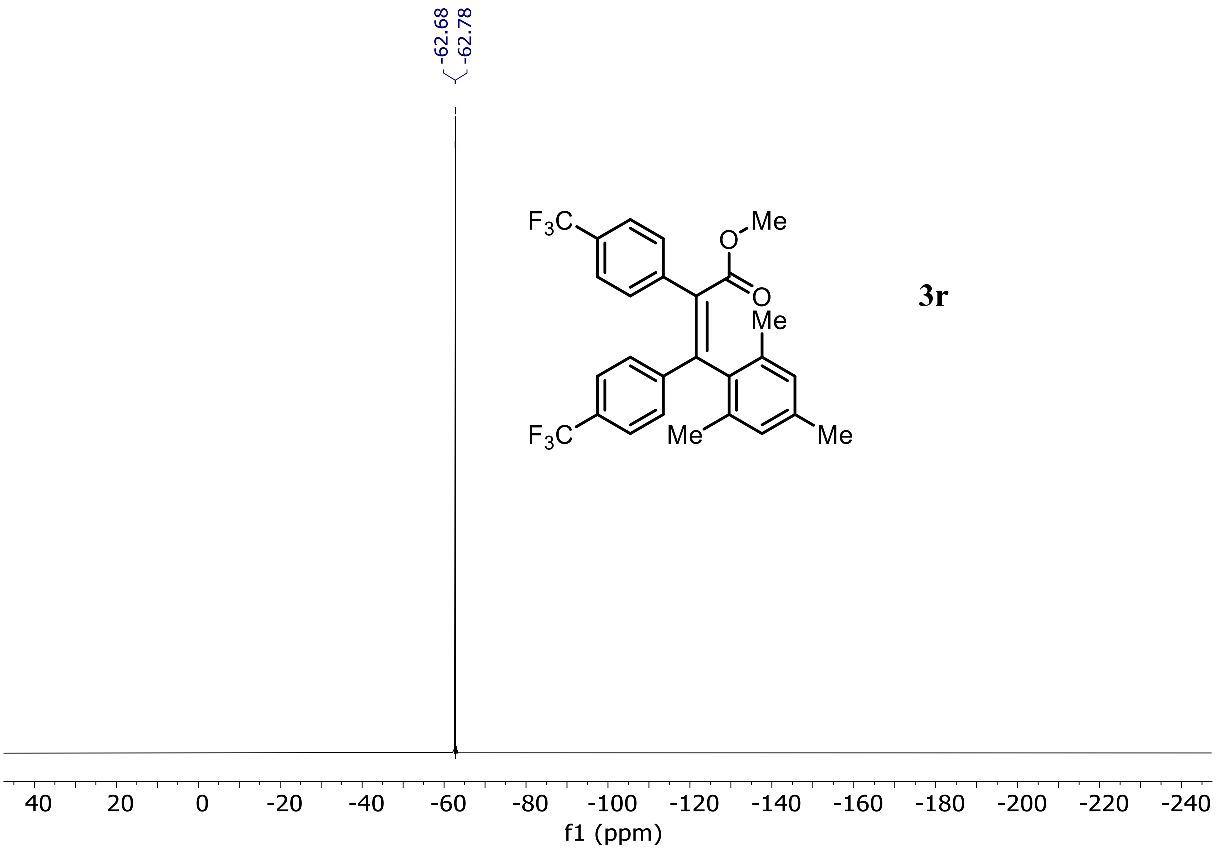
-62.68
-62.78
F3C
O
Me
O
3r
Me
F3C
Me
Me
40
20
0
-20
-40
-60
-80
-100
-120
-140
-160
-180
-200
-220
-240
f1 (ppm)

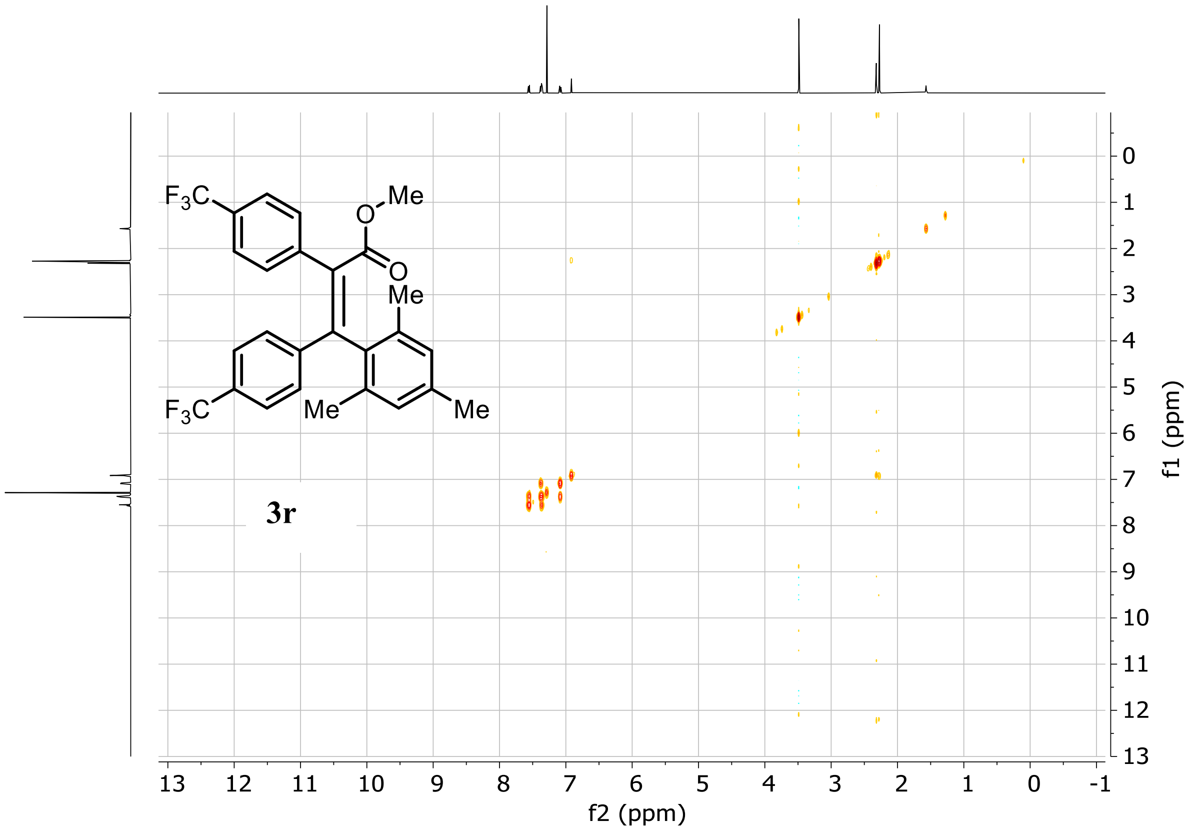
F3C
O
Me
O
Me
F3C
Me
Me
3r
f2 (ppm)
f1 (ppm)

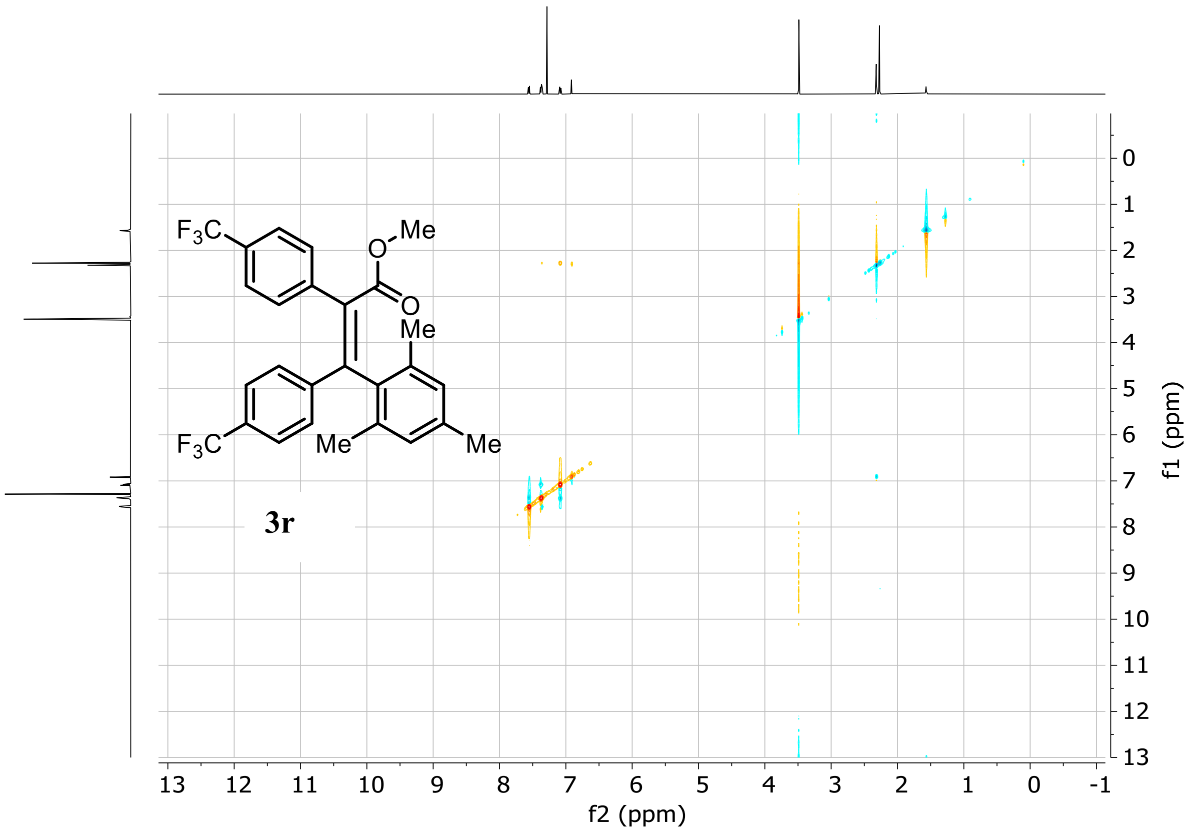

F3C
O
Me
O
Me
F3C
Me
Me
3r
f2 (ppm)
f1 (ppm)

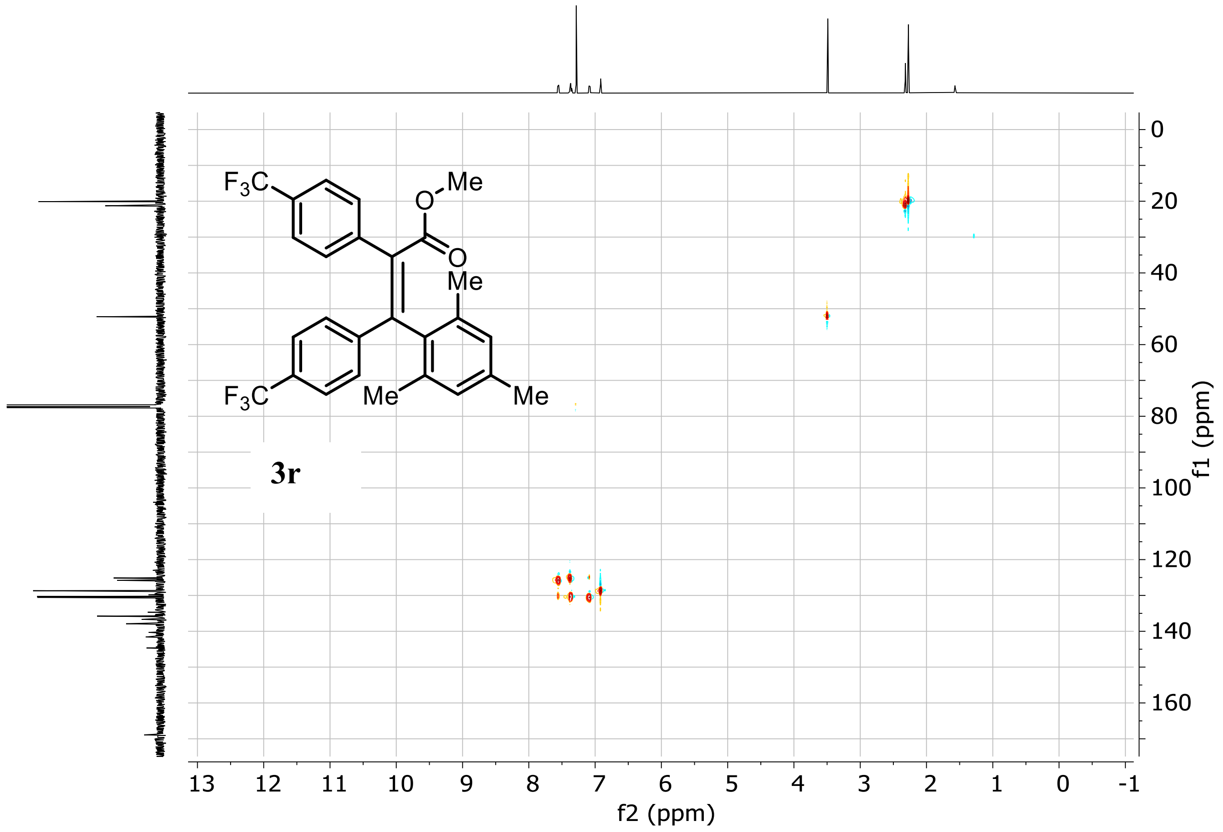

F3C
O
Me
O
Me
F3C
Me
Me
3r
f2 (ppm)
f1 (ppm)

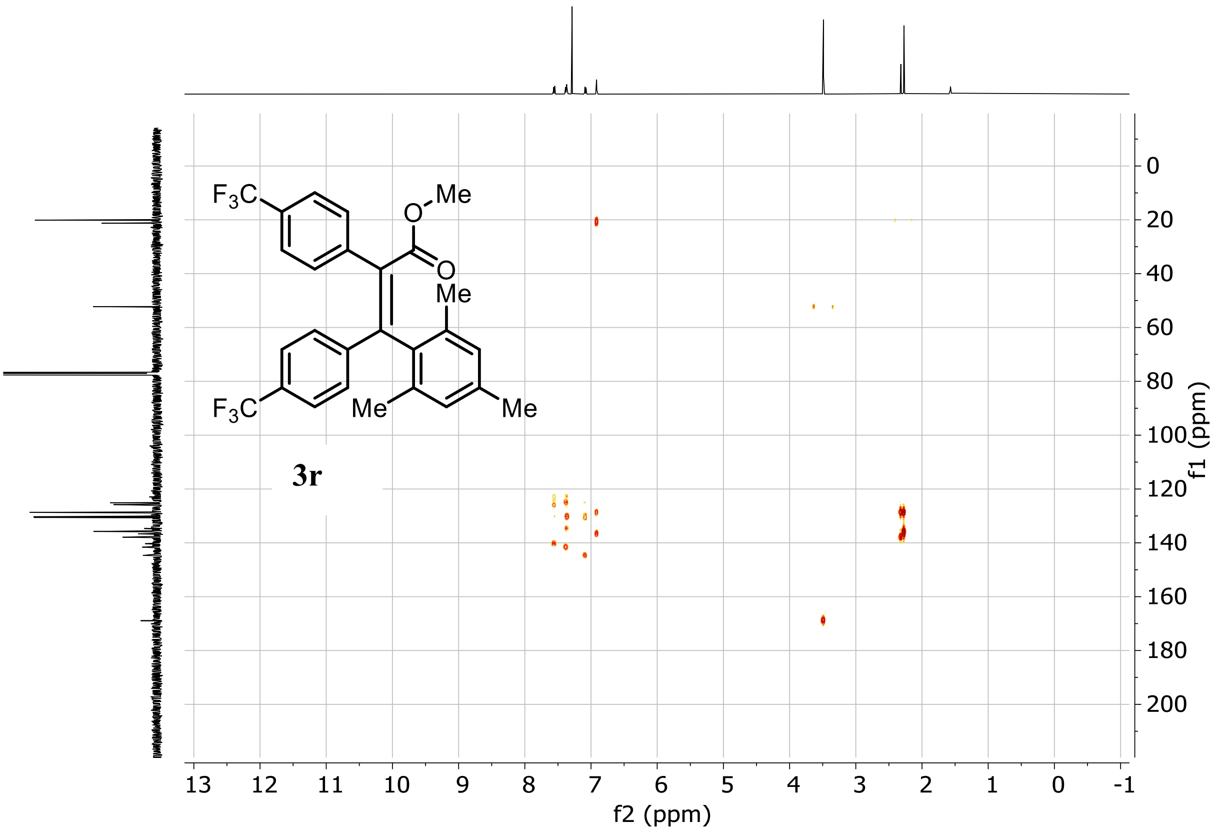
F3C
O
Me
O
Me
F3C
Me
Me
3r
f2 (ppm)
f1 (ppm)

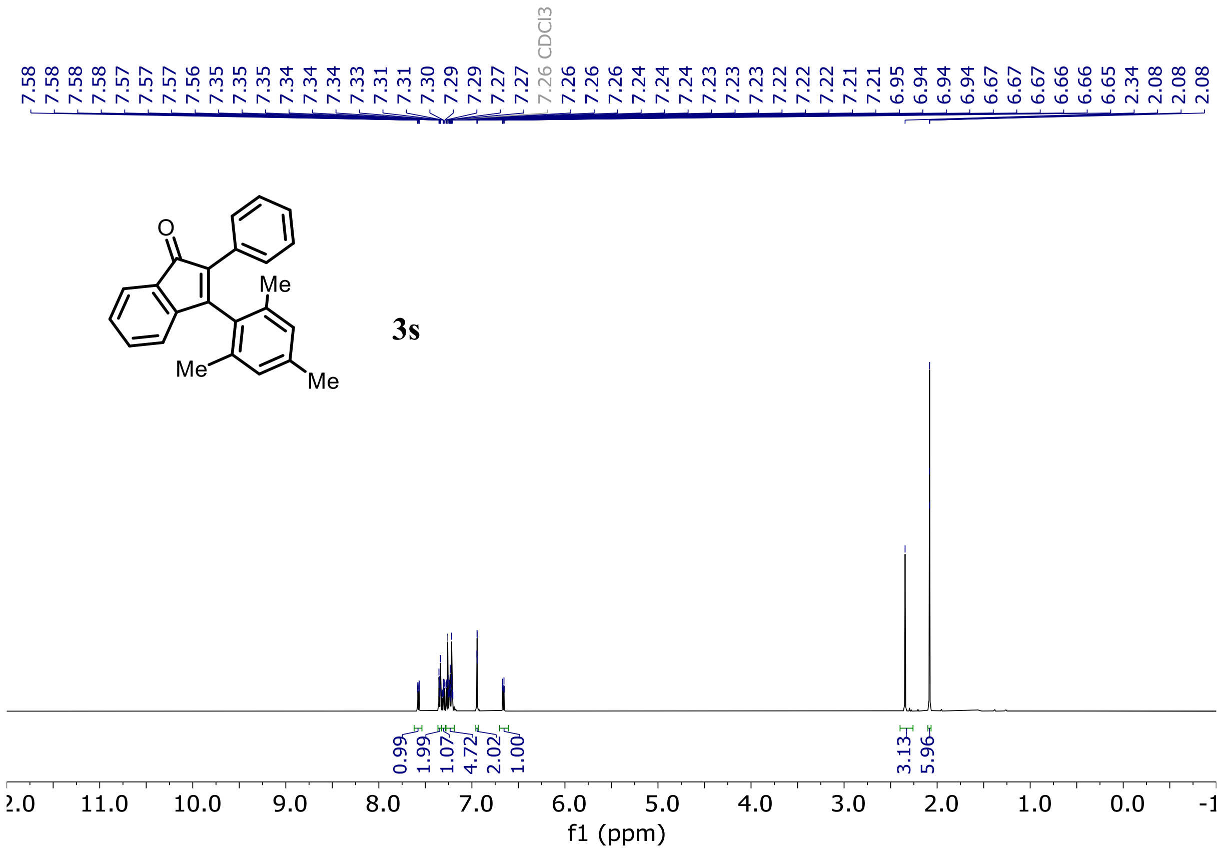

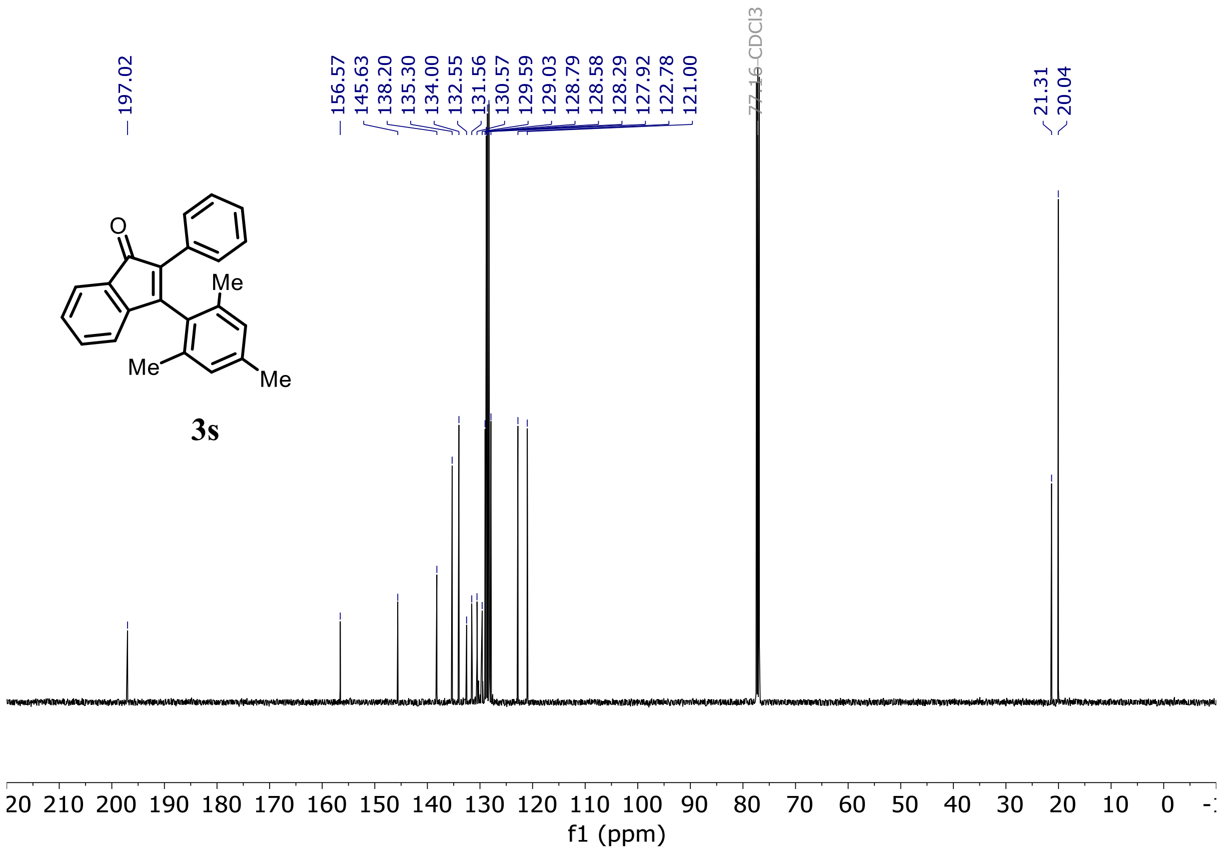

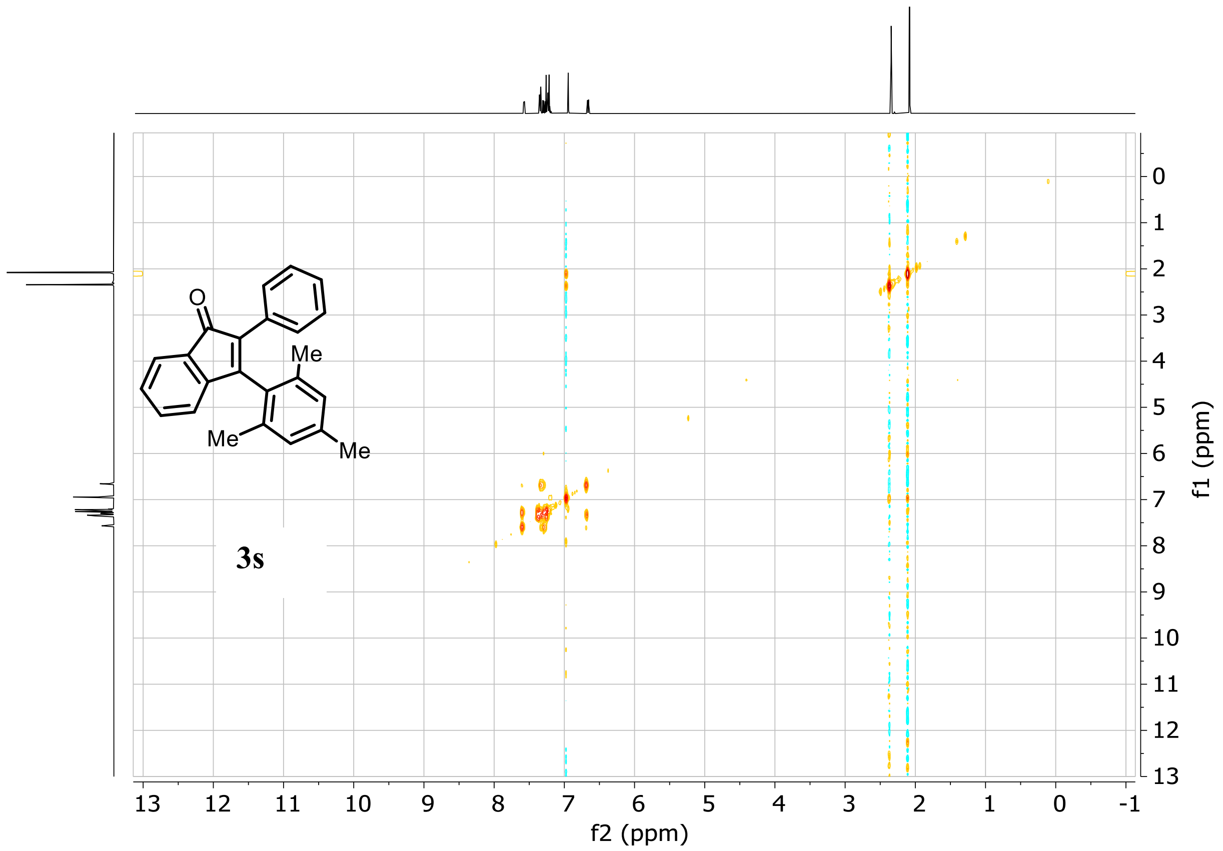
O
Me
Me
Me
3s
f2 (ppm)
f1 (ppm)

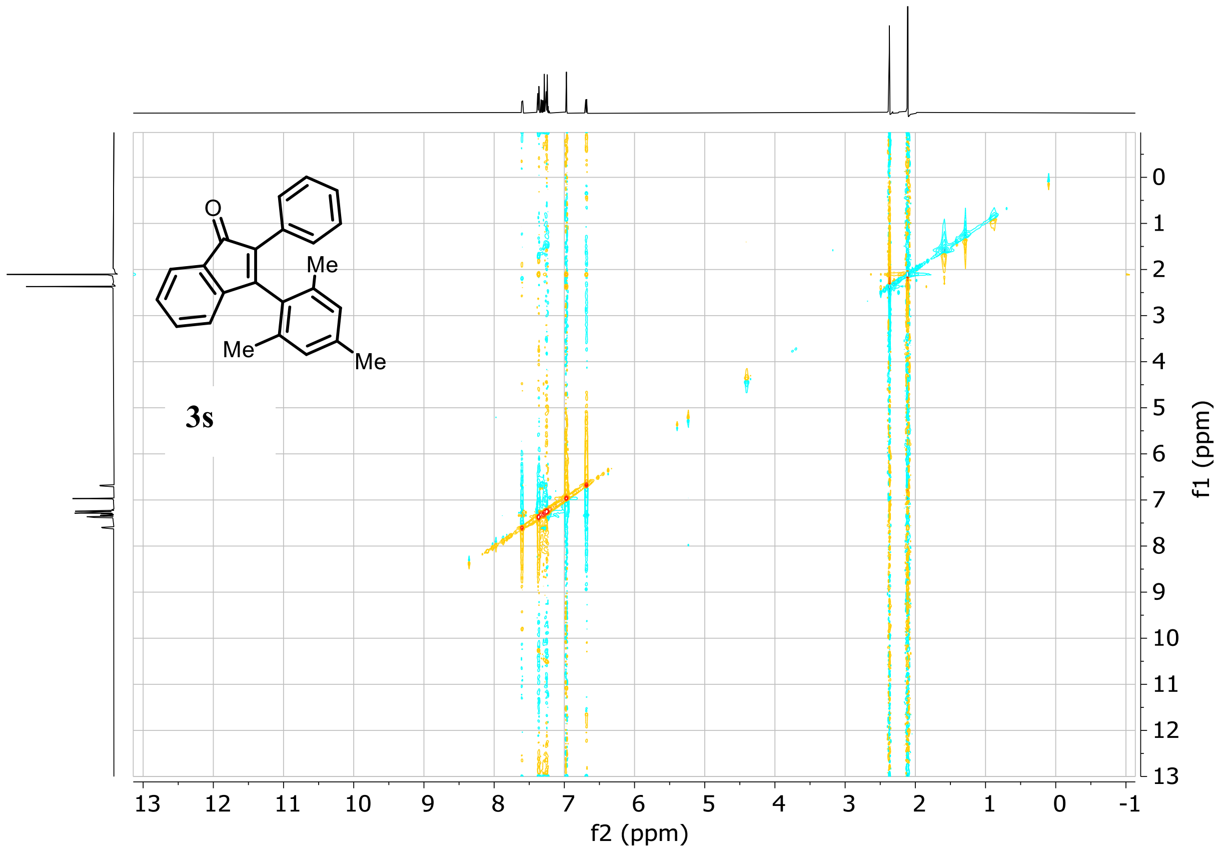
O
Me
Me
Me
3s
f2 (ppm)
f1 (ppm)

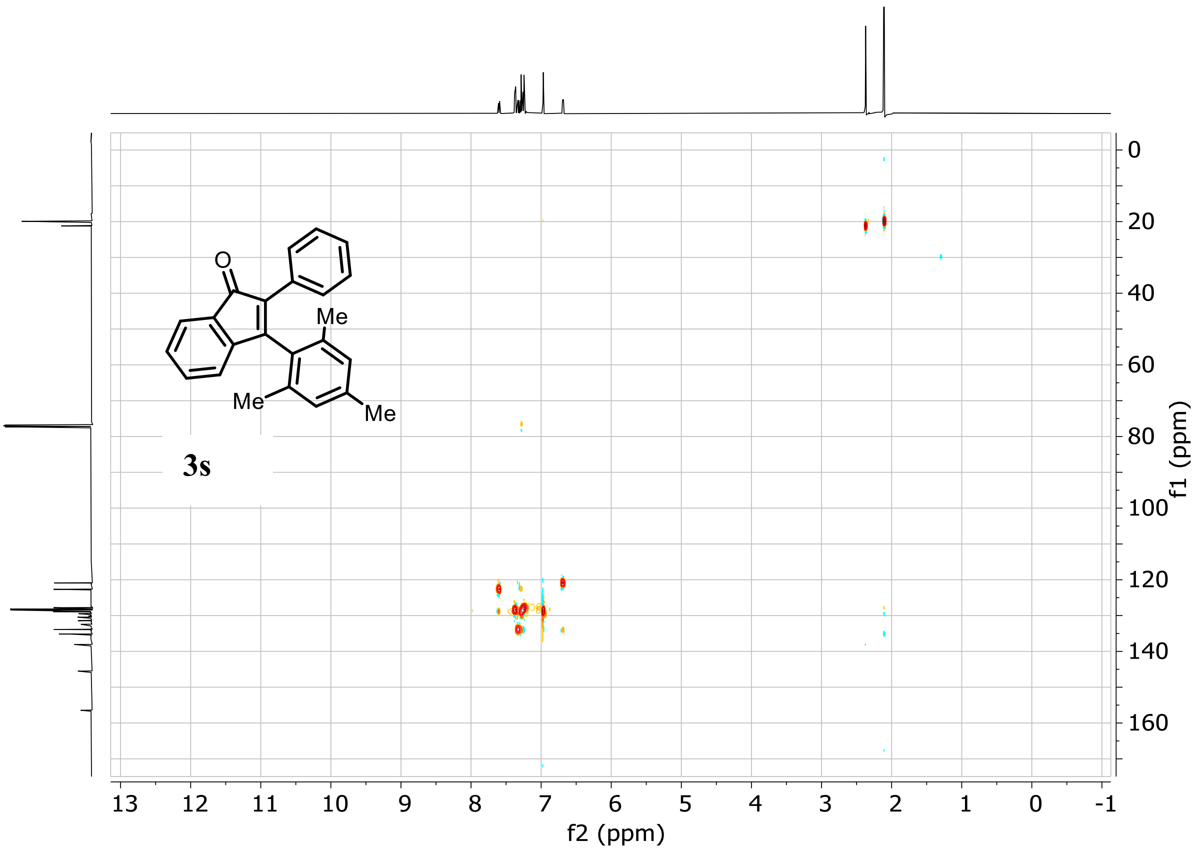

O
Me
Me
Me
3s
f2 (ppm)
f1 (ppm)

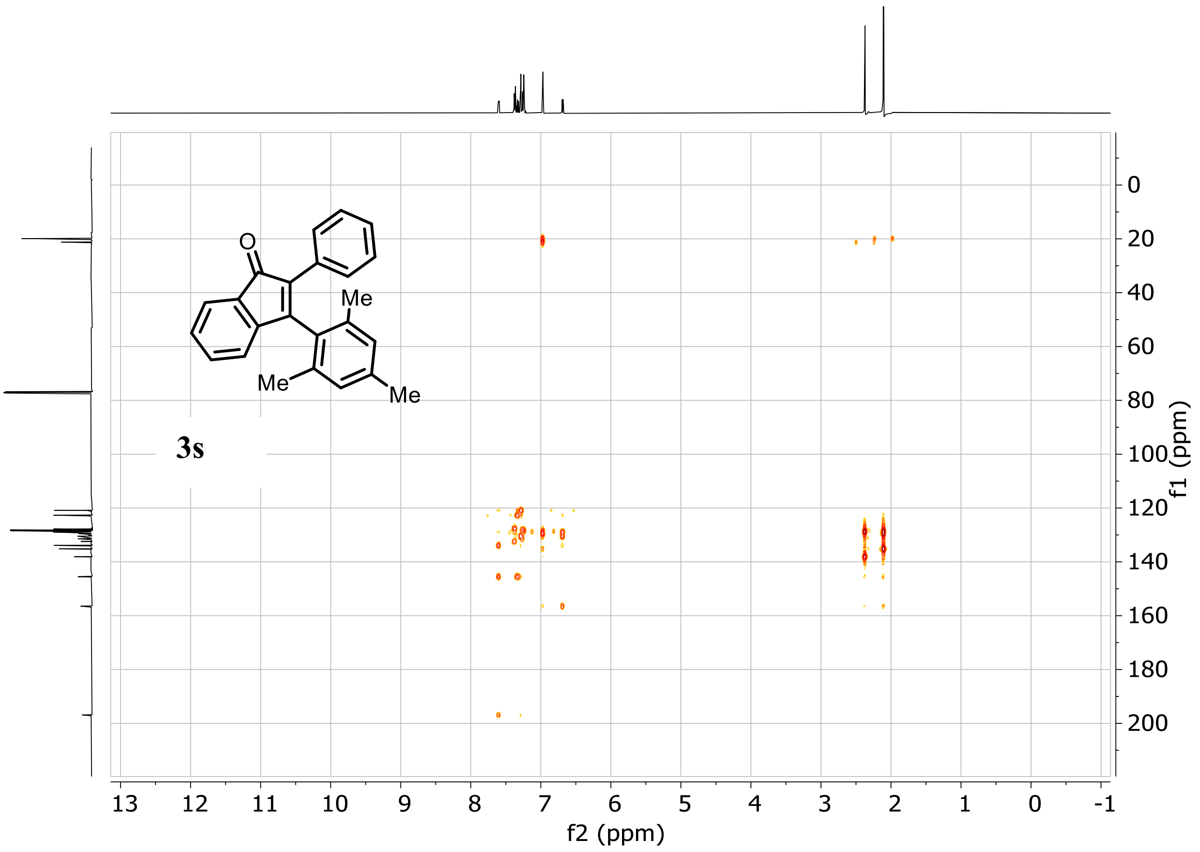

O
Me
Me
Me
3s
f2 (ppm)
f1 (ppm)

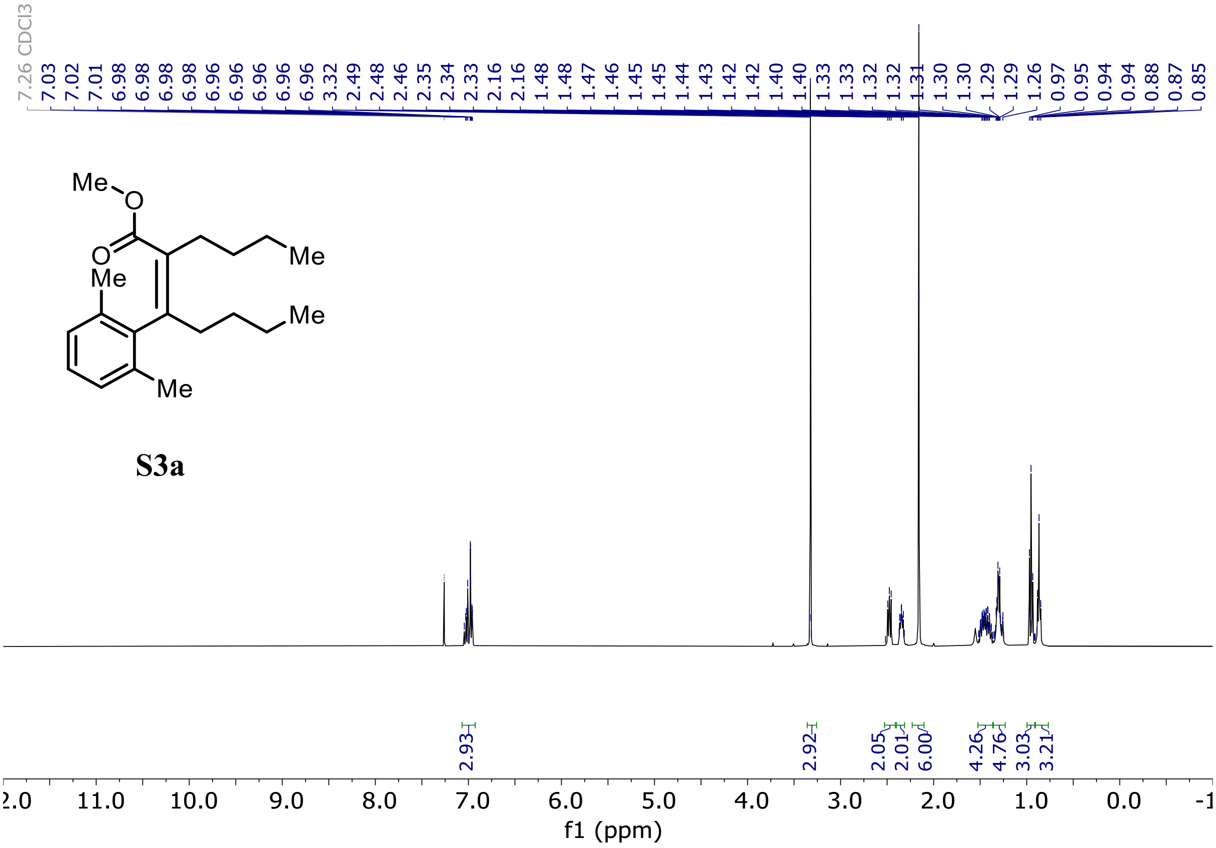

S3a

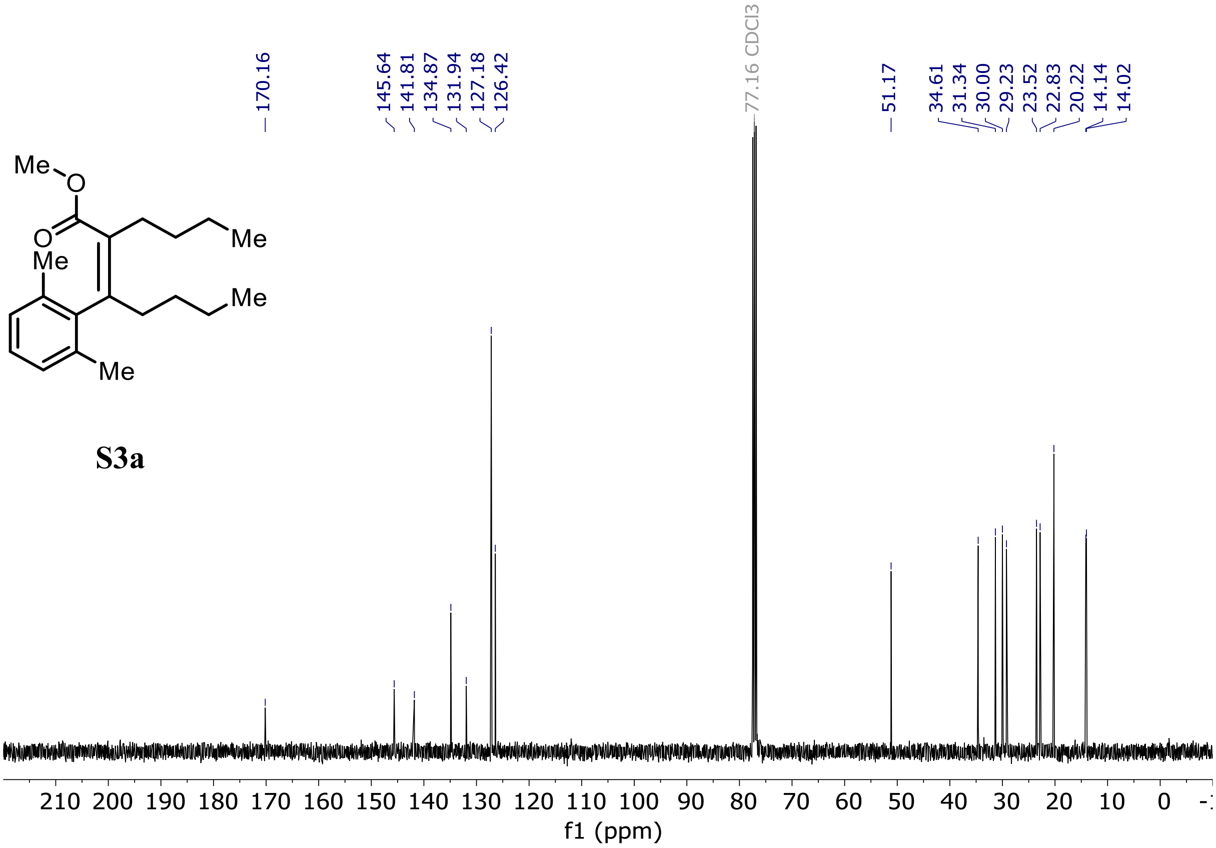

S3a

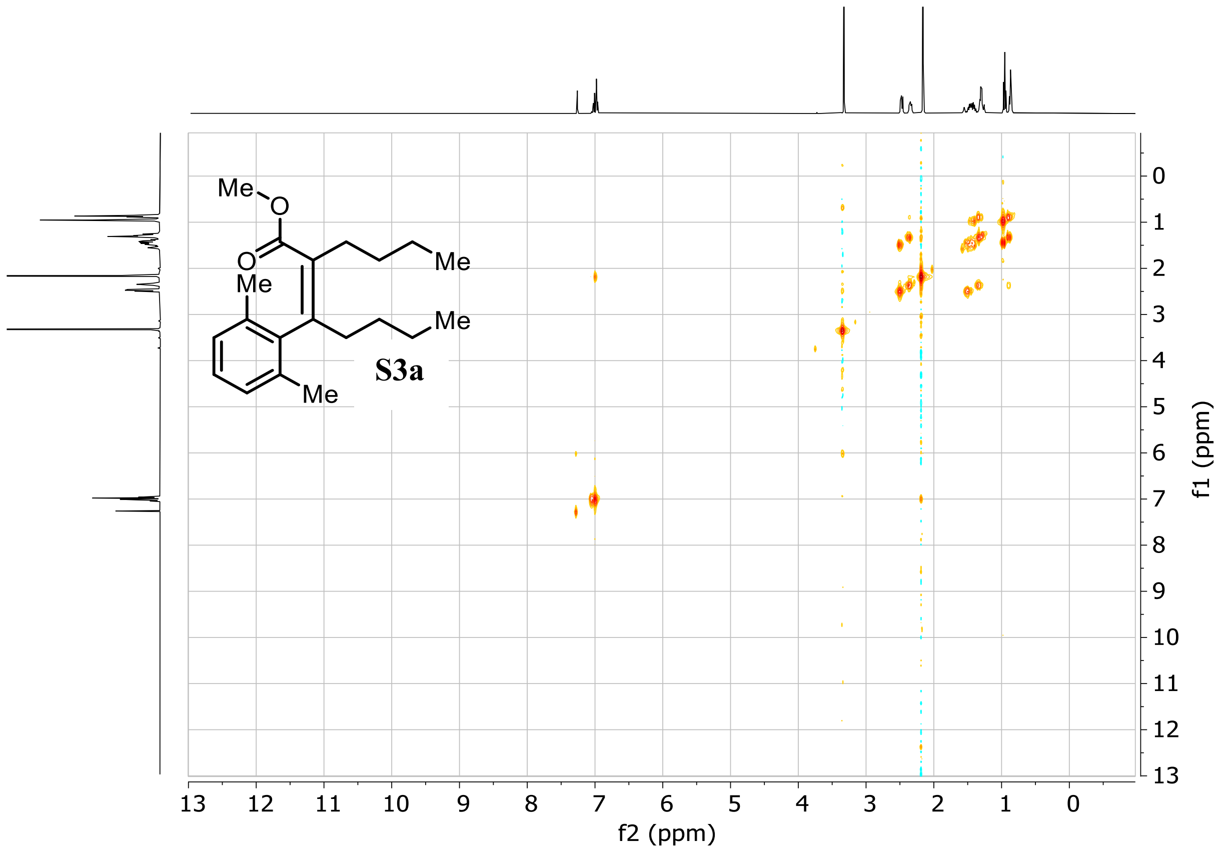

Me
O
O
Me
Me
Me
Me
S3a
f2 (ppm)
f1 (ppm)

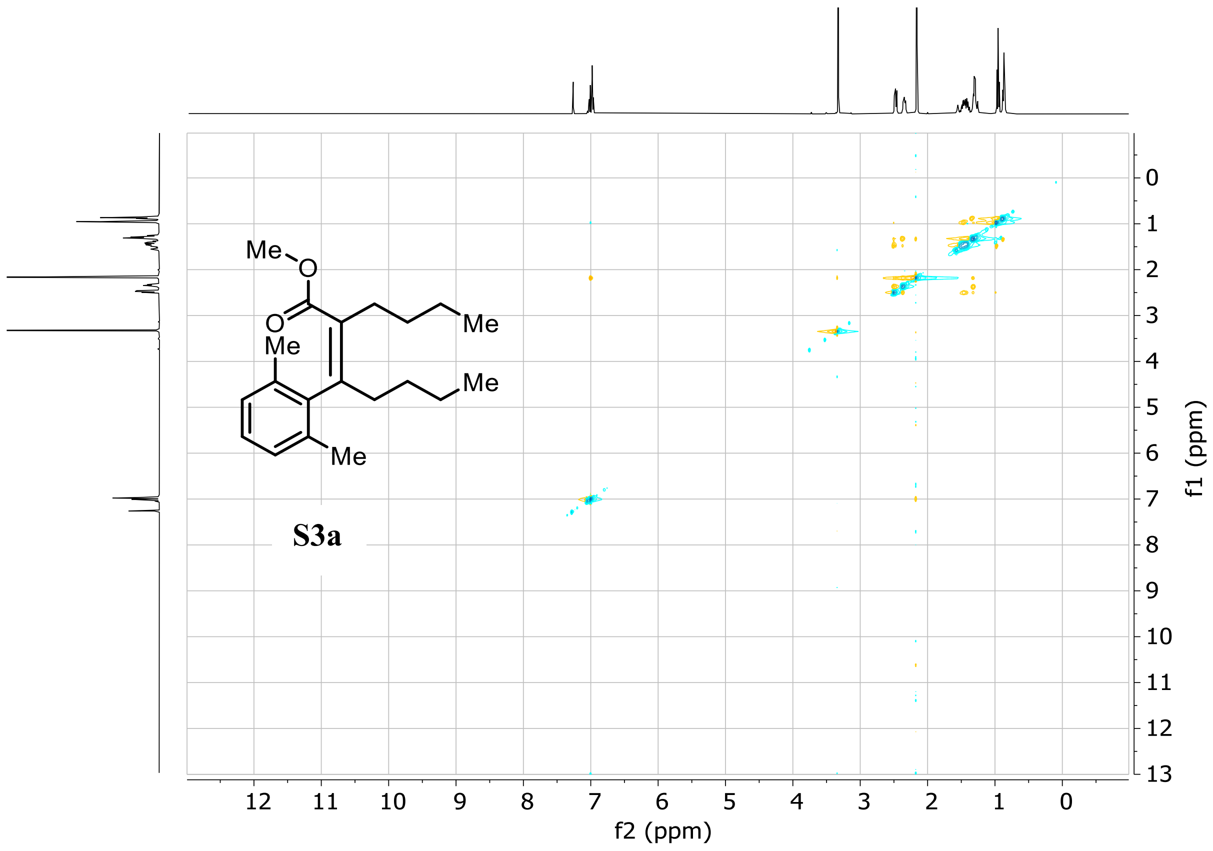

Me
O
O
Me
Me
Me
Me
S3a
f2 (ppm)
f1 (ppm)

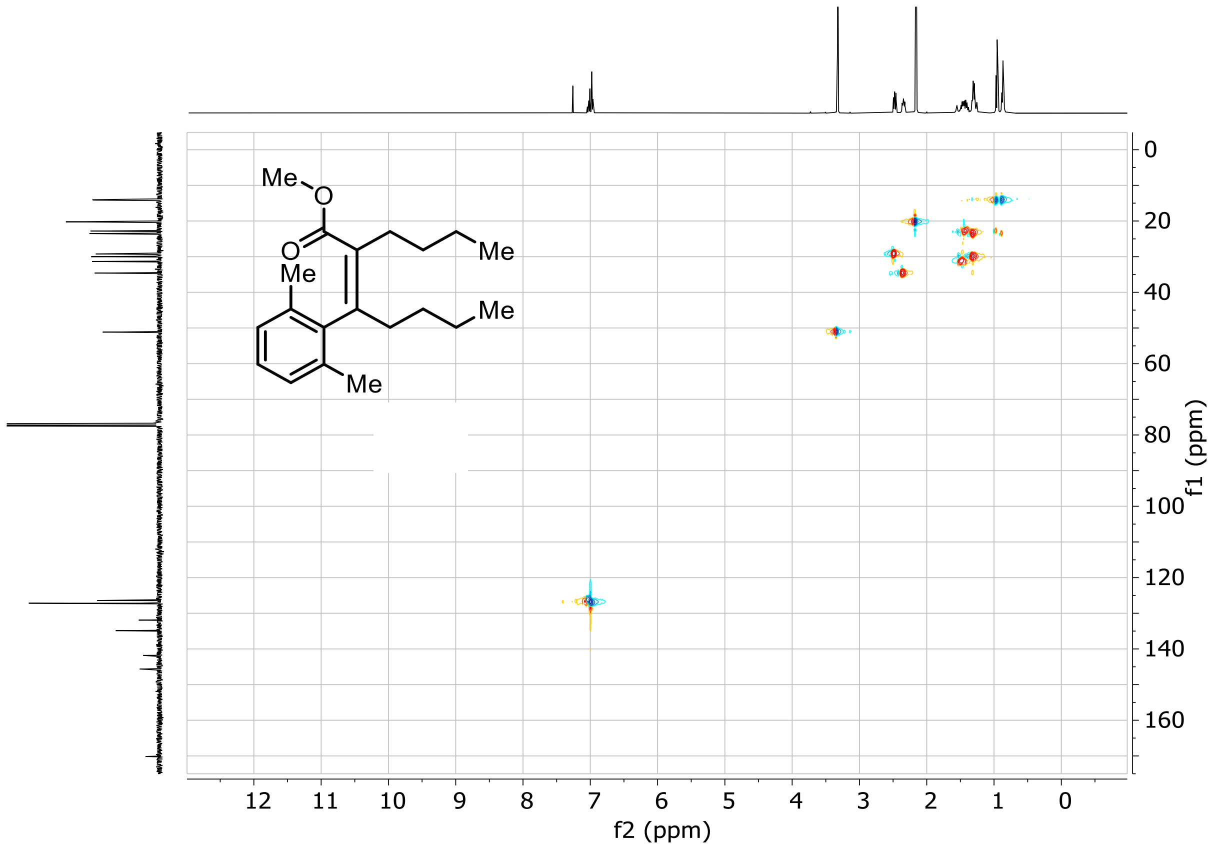
Me
O
O
Me
Me
Me
Me
f1 (ppm)
f2 (ppm)

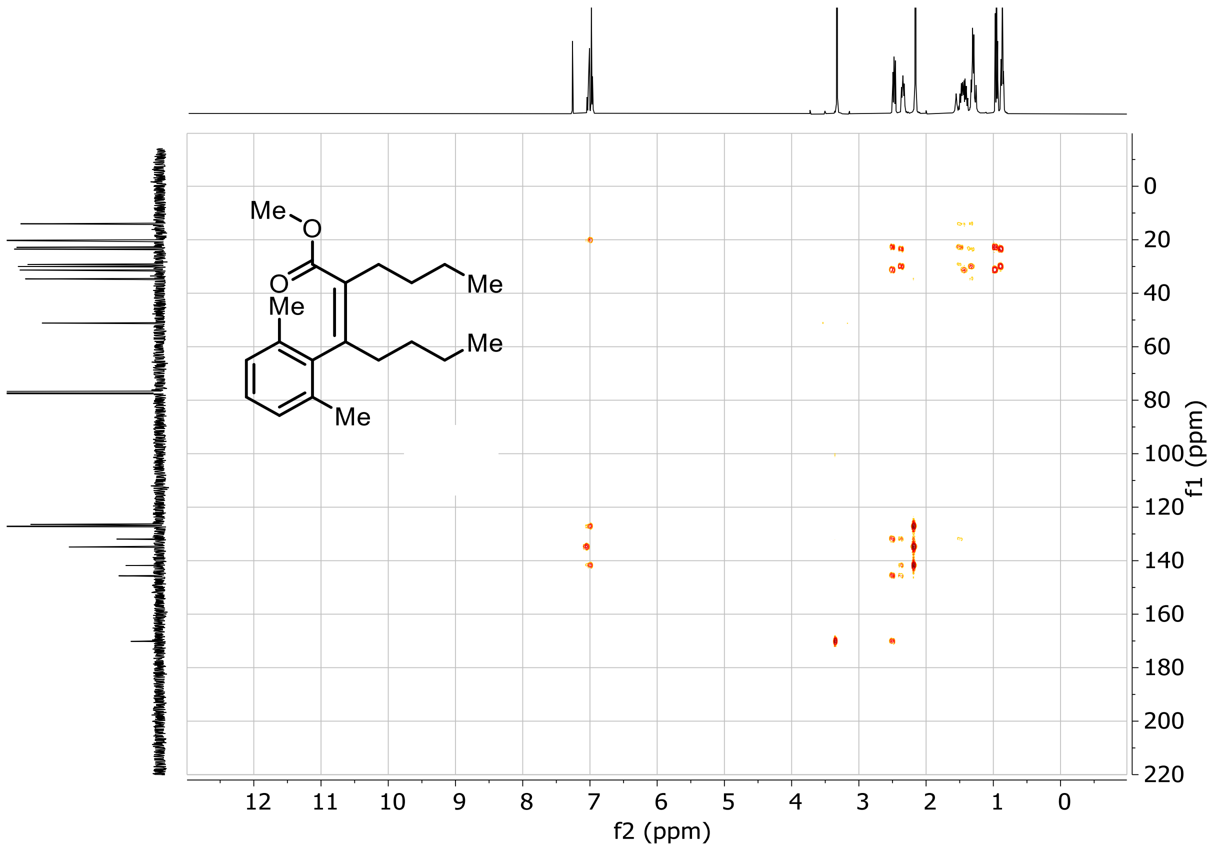
Me
O
O
Me
Me
Me
Me
f1 (ppm)
f2 (ppm)

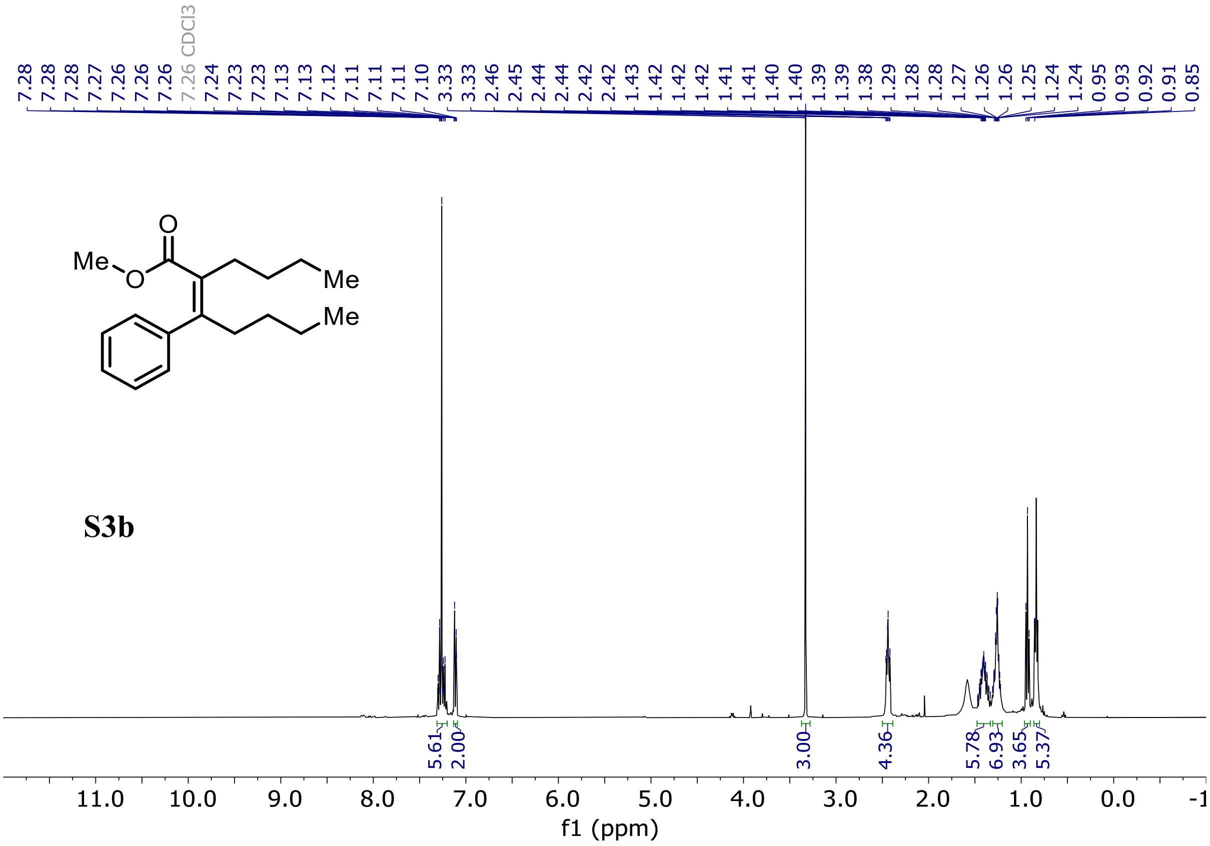
7.28
7.28
7.28
7.27
7.26
7.26
7.26
7.26 CDCl3
7.24
7.23
7.23
7.13
7.13
7.12
7.11
7.11
7.11
7.10
3.33
3.33
2.46
2.45
2.44
2.44
2.42
2.42
1.43
1.42
1.42
1.42
1.41
1.41
1.40
1.40
1.39
1.39
1.38
1.29
1.28
1.28
1.27
1.26
1.26
1.25
1.24
1.24
0.95
0.93
0.92
0.91
0.85
Me
O
O
Me
Me
S3b
5.61
2.00
3.00
4.36
5.78
6.93
3.65
5.37
11.0 10.0 9.0 8.0 7.0 6.0 5.0 4.0 3.0 2.0 1.0 0.0 -1
f1 (ppm)

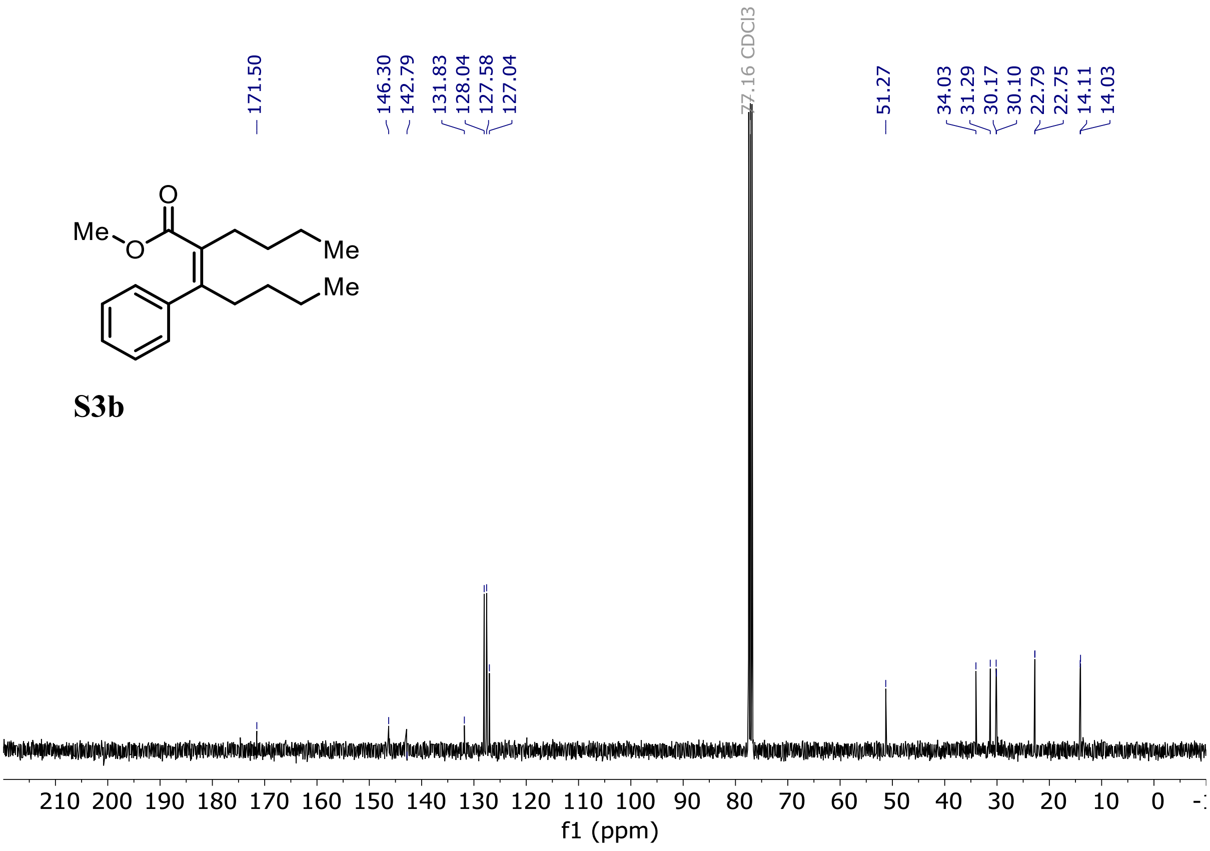
171.50
146.30
142.79
131.83
128.04
127.58
127.04
77.16 CDCl3
51.27
34.03
31.29
30.17
30.10
22.79
22.75
14.11
14.03
Me
O
O
Me
Me
S3b
210 200 190 180 170 160 150 140 130 120 110 100 90 80 70 60 50 40 30 20 10 0
f1 (ppm)

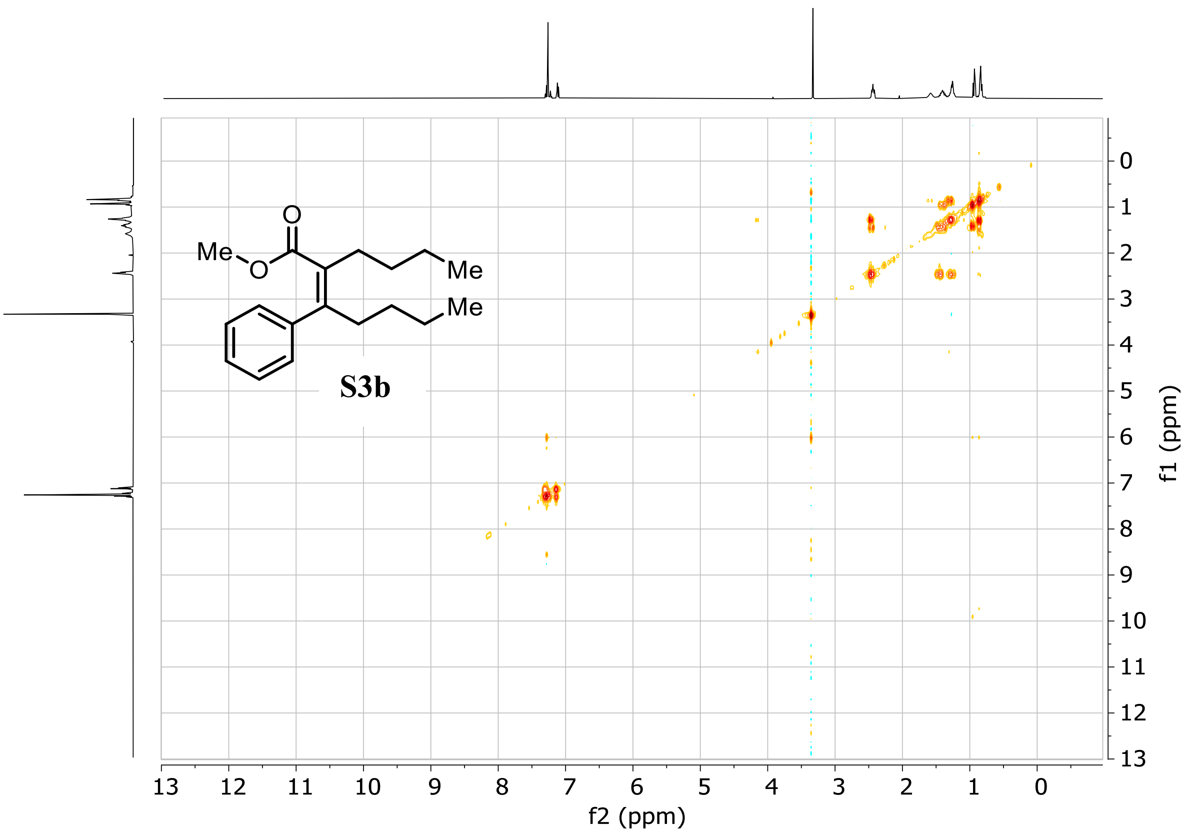

Me
O
O
Me
Me
S3b
f2 (ppm)
f1 (ppm)

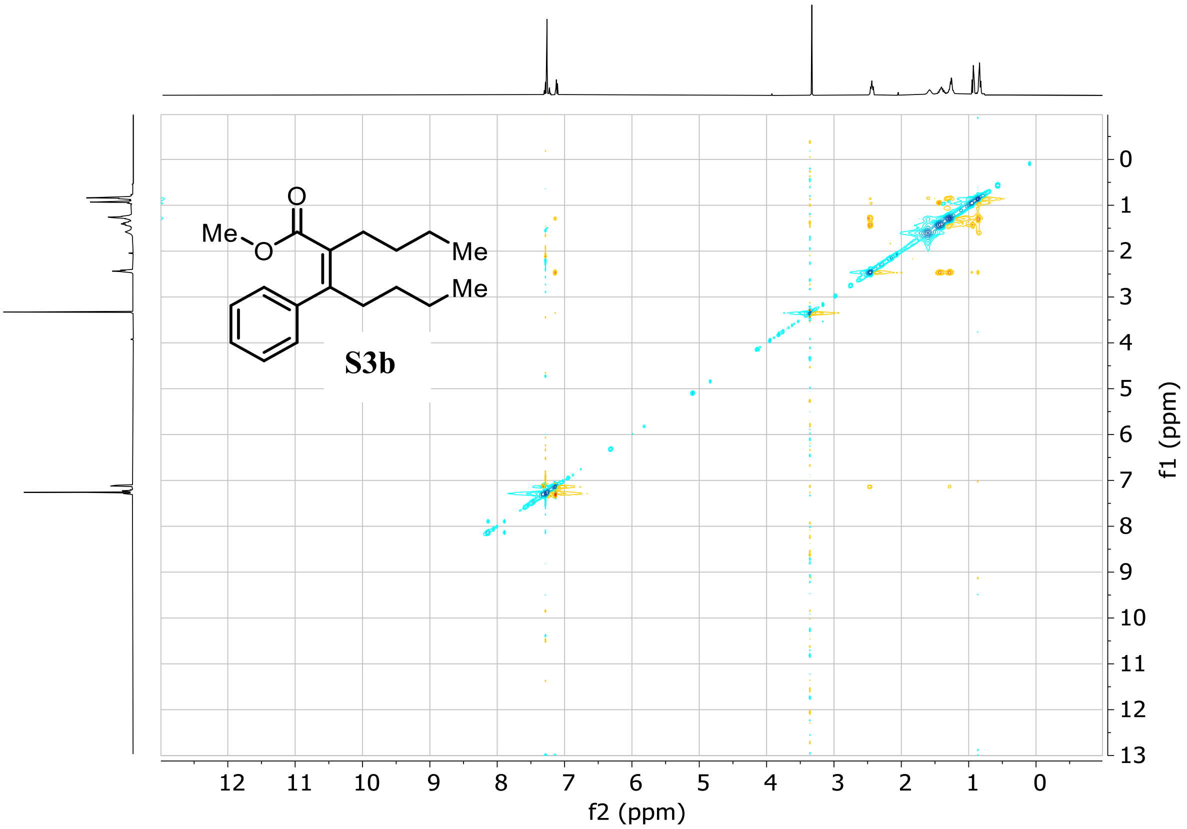

Me
O
O
Me
Me
S3b
f2 (ppm)
f1 (ppm)

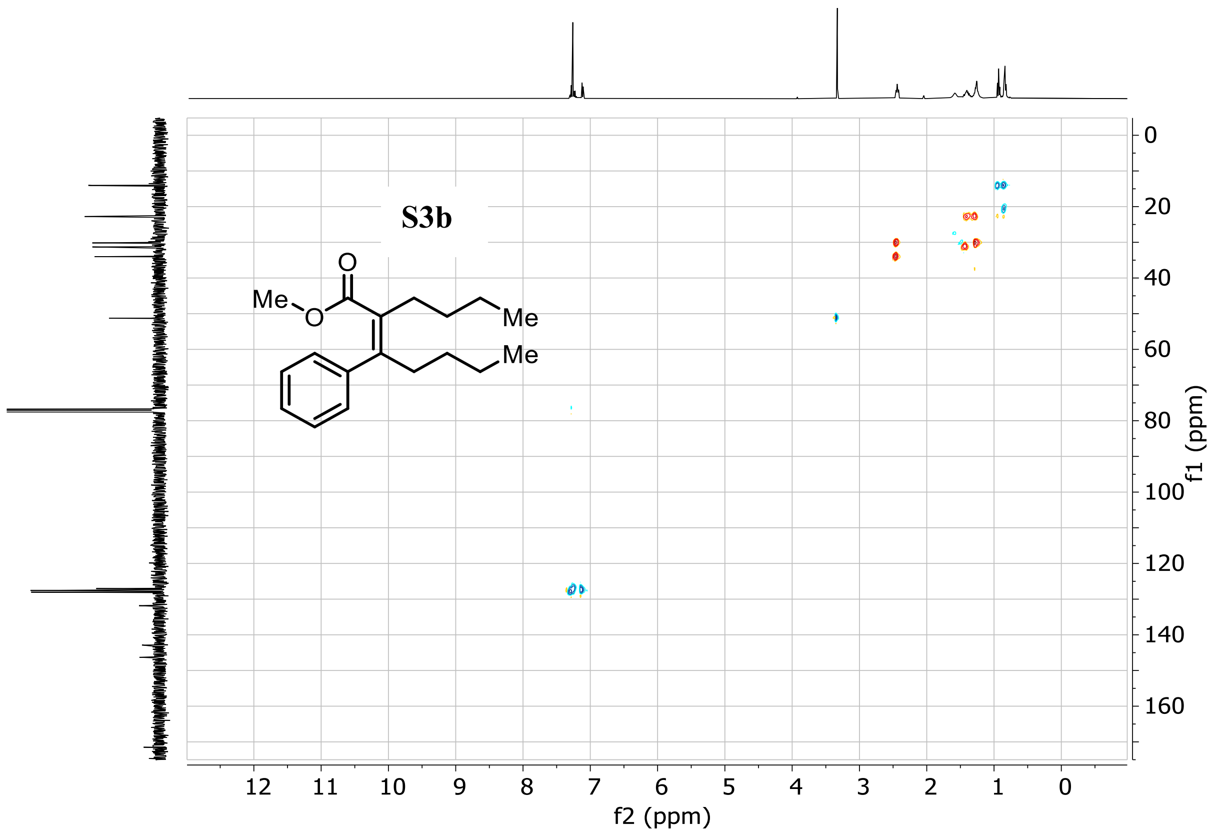
S3b
O
Me
O
Me
Me
0
20
40
60
80
100
120
140
160
f1 (ppm)
12
11
10
9
8
7
6
5
4
3
2
1
0
f2 (ppm)

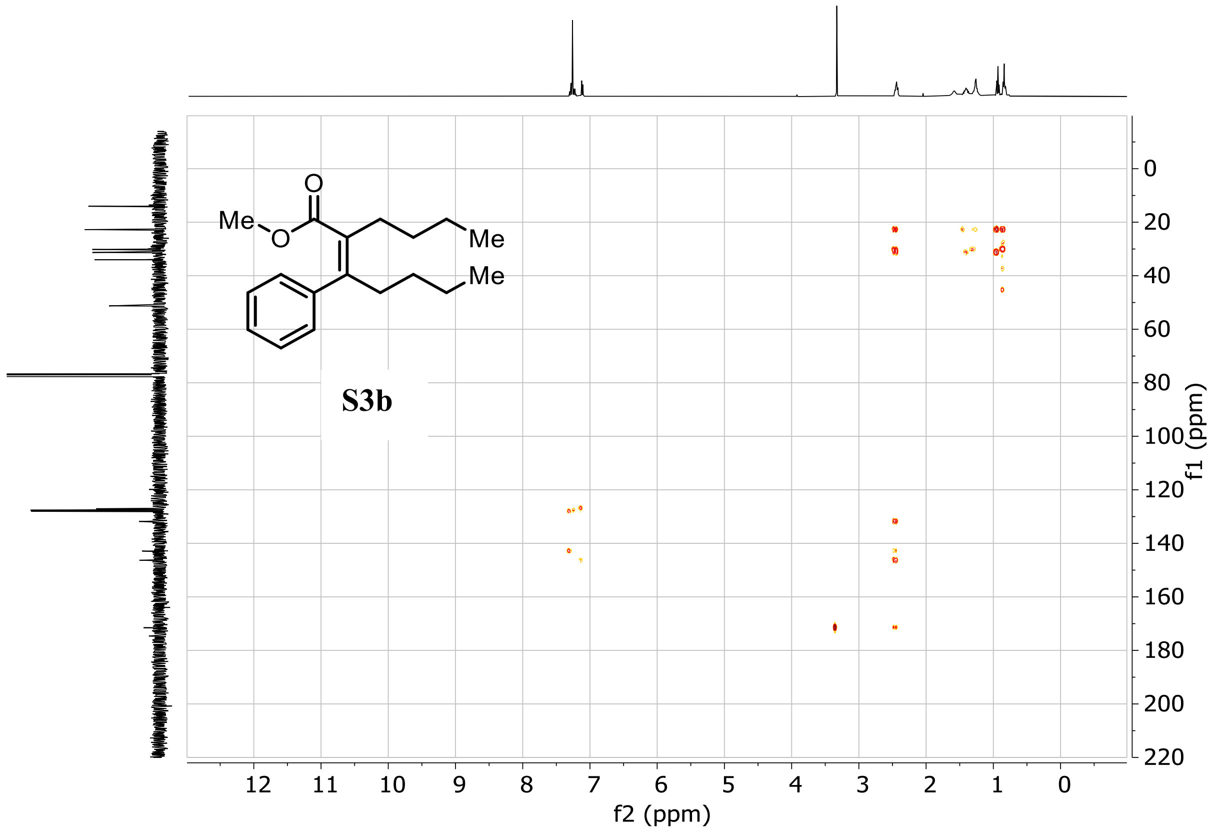
O
Me
O
Me
Me
S3b
0
20
40
60
80
100
120
140
160
180
200
220
f1 (ppm)
12
11
10
9
8
7
6
5
4
3
2
1
0
f2 (ppm)

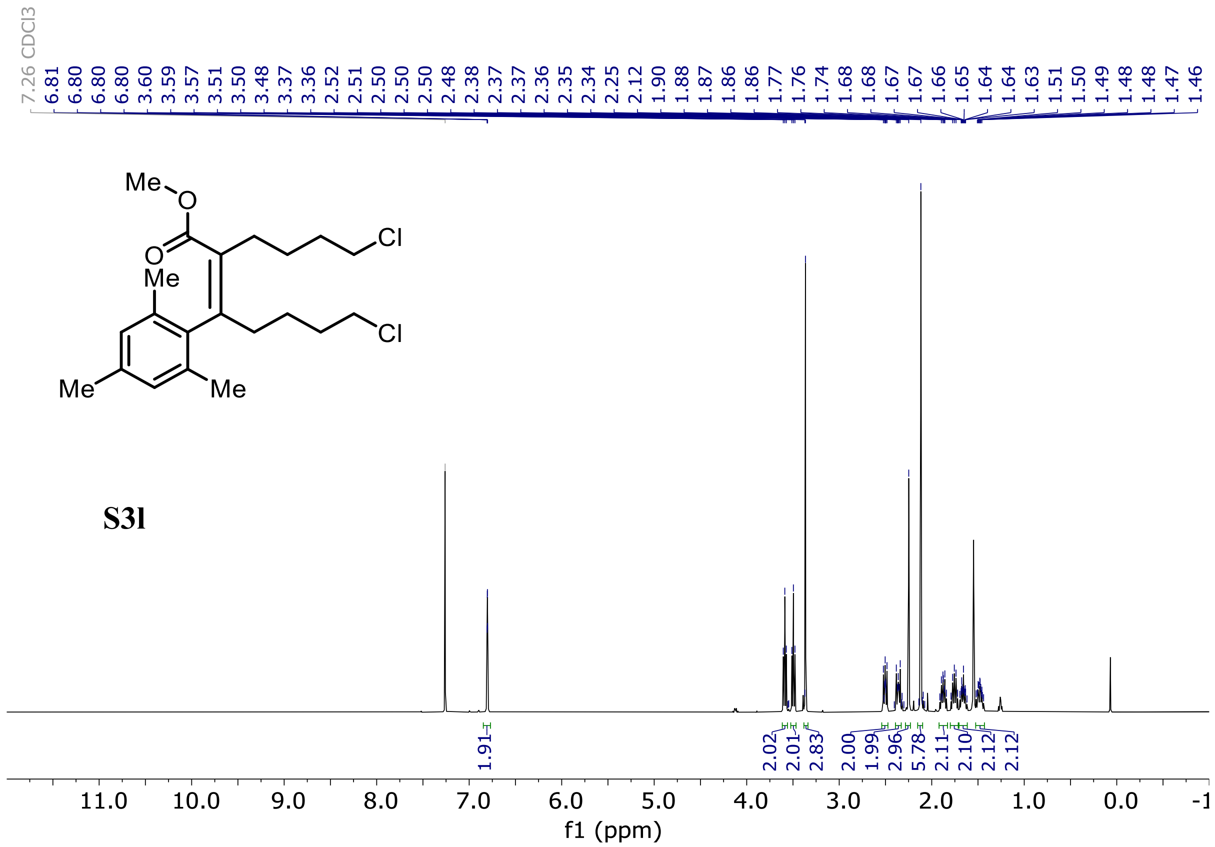
7.26 CDCl3
6.81
6.80
6.80
6.80
3.60
3.59
3.57
3.51
3.50
3.48
3.37
3.36
2.52
2.51
2.50
2.50
2.50
2.48
2.38
2.37
2.37
2.36
2.35
2.34
2.25
2.12
1.90
1.88
1.87
1.86
1.86
1.77
1.76
1.74
1.68
1.68
1.67
1.67
1.66
1.65
1.64
1.64
1.63
1.51
1.50
1.49
1.48
1.48
1.47
1.46
Me
O
O
Cl
Me
Cl
Me
Me
S3l
1.91
2.02
2.01
2.83
2.00
1.99
2.96
5.78
2.11
2.10
2.12
2.12
11.0
10.0
9.0
8.0
7.0
6.0
5.0
4.0
3.0
2.0
1.0
0.0
-1
f1 (ppm)

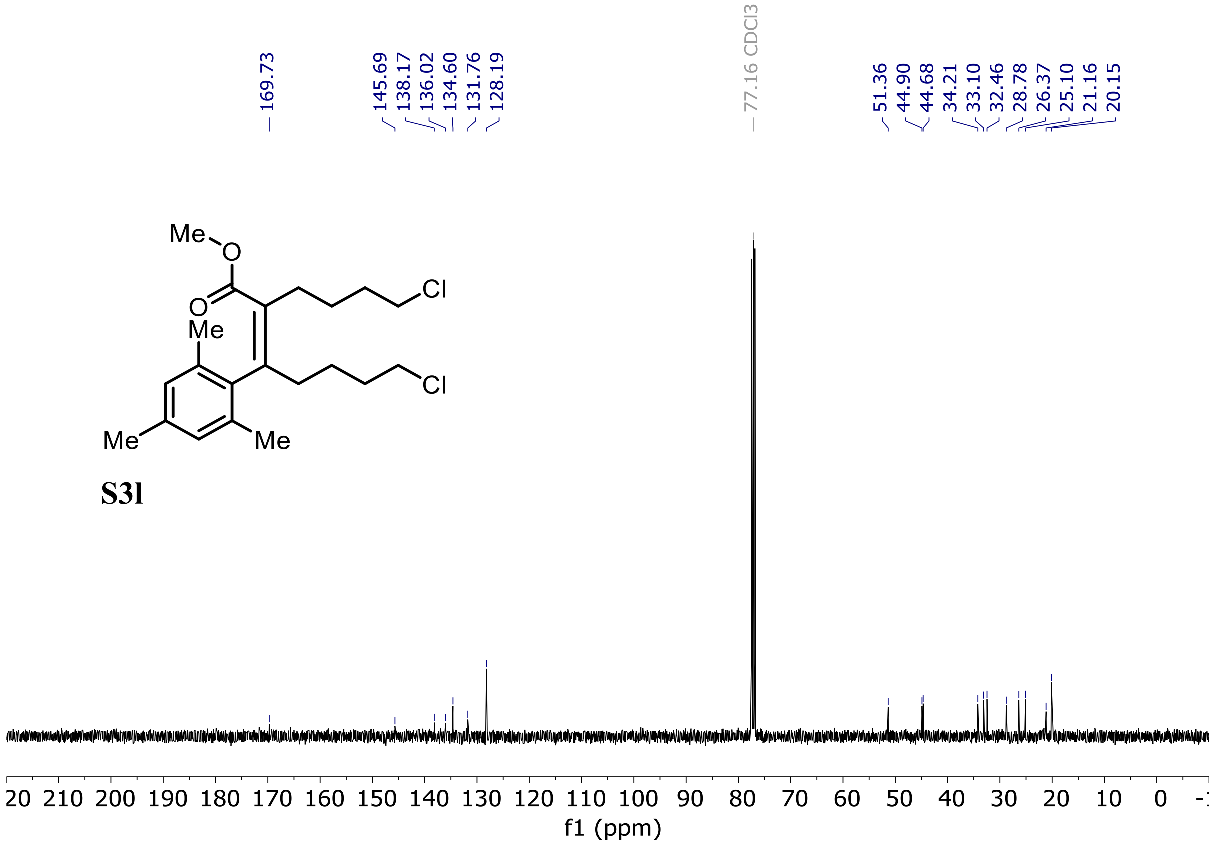
169.73
145.69
138.17
136.02
134.60
131.76
128.19
77.16 CDCl3
51.36
44.90
44.68
34.21
33.10
32.46
28.78
26.37
25.10
21.16
20.15
Me
O
O
Cl
Me
Cl
Me
Me
S3l
20 210 200 190 180 170 160 150 140 130 120 110 100 90 80 70 60 50 40 30 20 10 0
f1 (ppm)

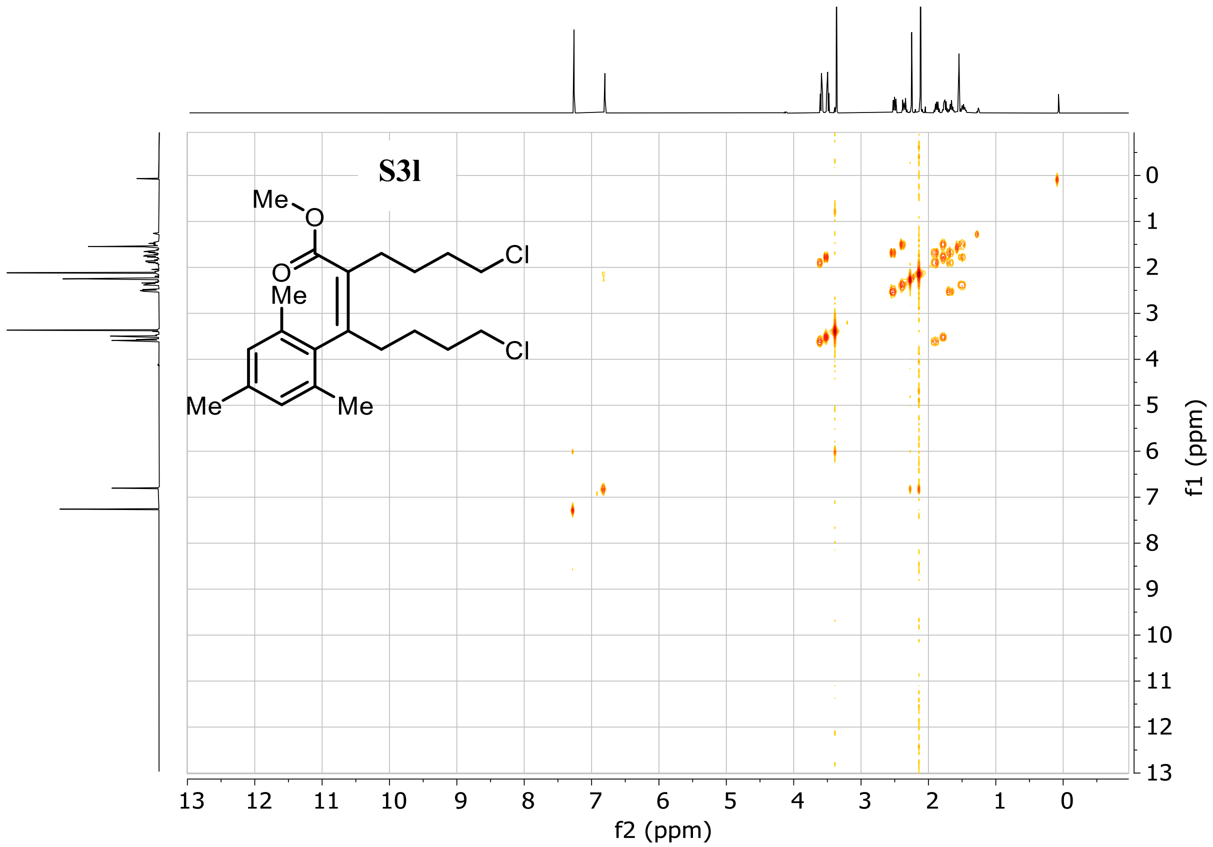
S3l
Me
O
O
Cl
Me
Cl
Me
Me
f2 (ppm)
f1 (ppm)

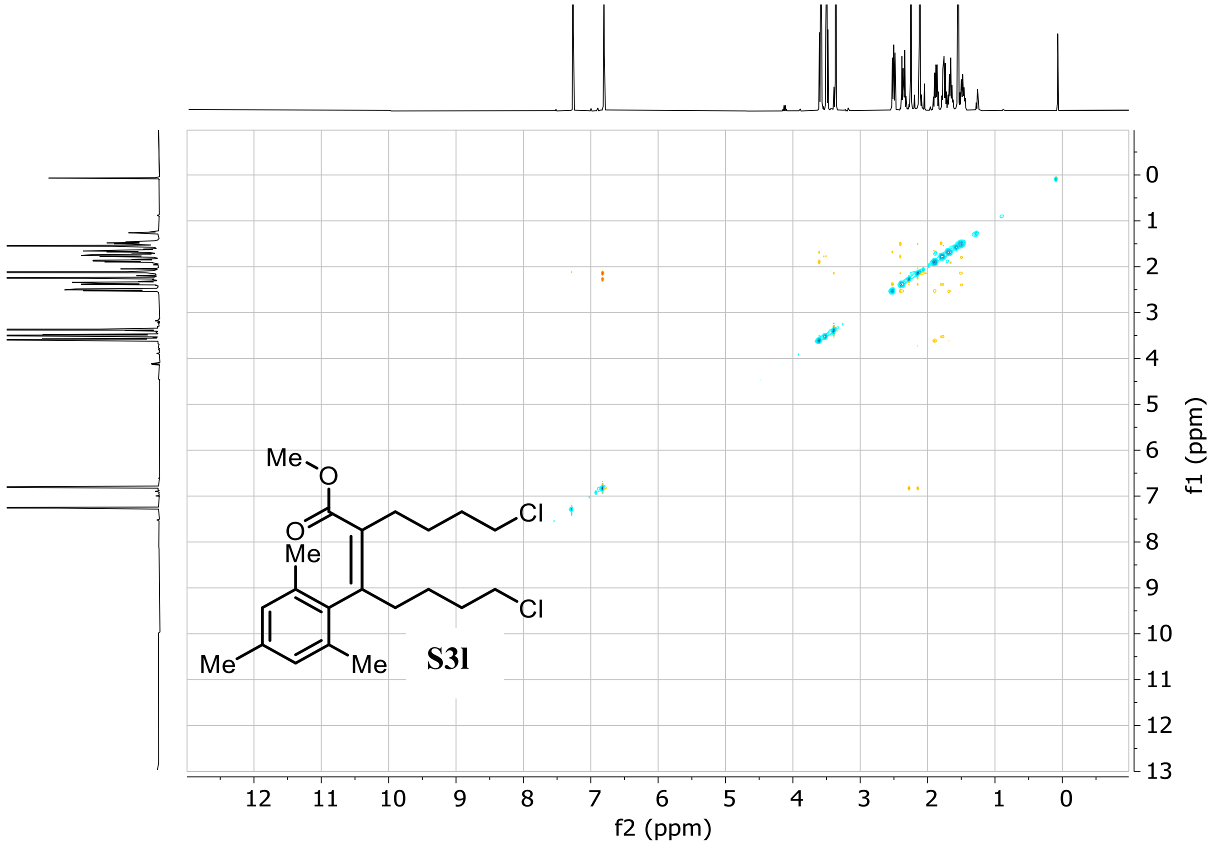
Me
O
O
Cl
Me
Cl
Me
Me
S3l
f2 (ppm)
f1 (ppm)

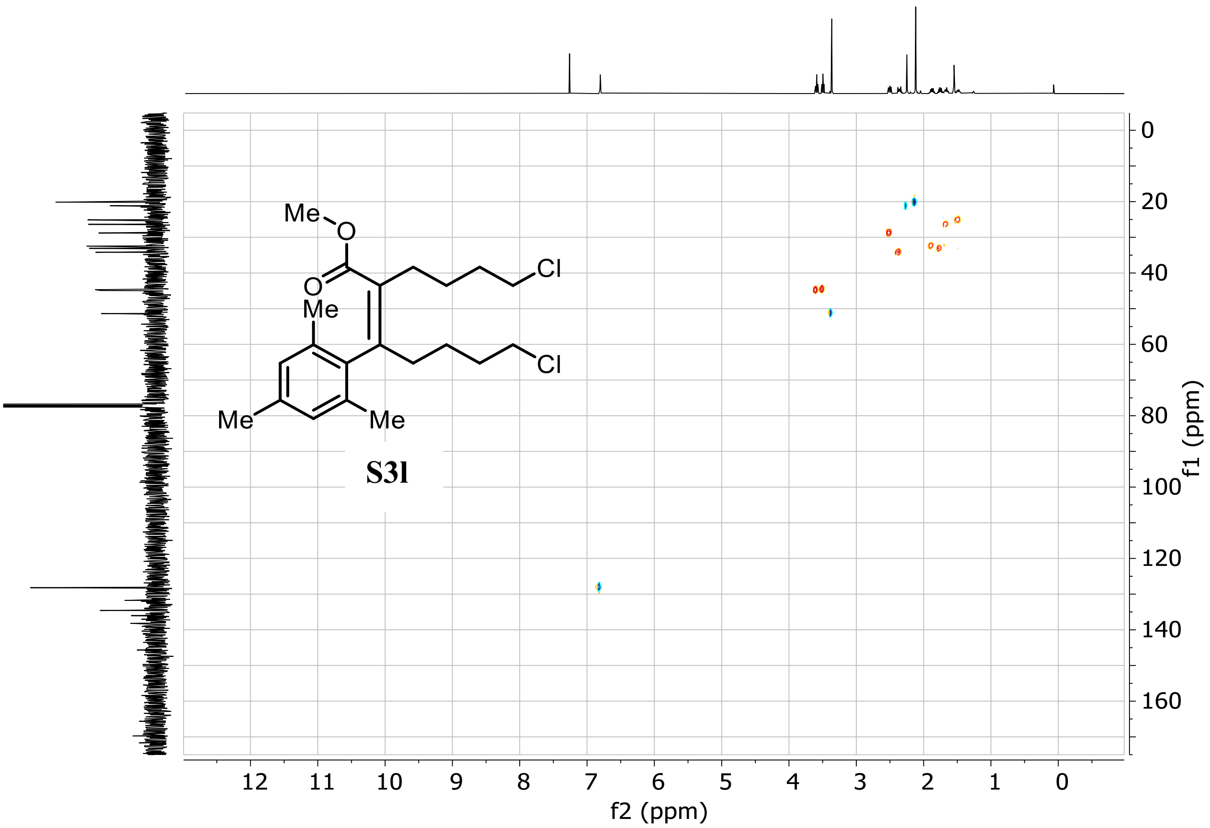

Me
O
O
Cl
Me
Cl
Me
Me
S3l
f2 (ppm)
f1 (ppm)

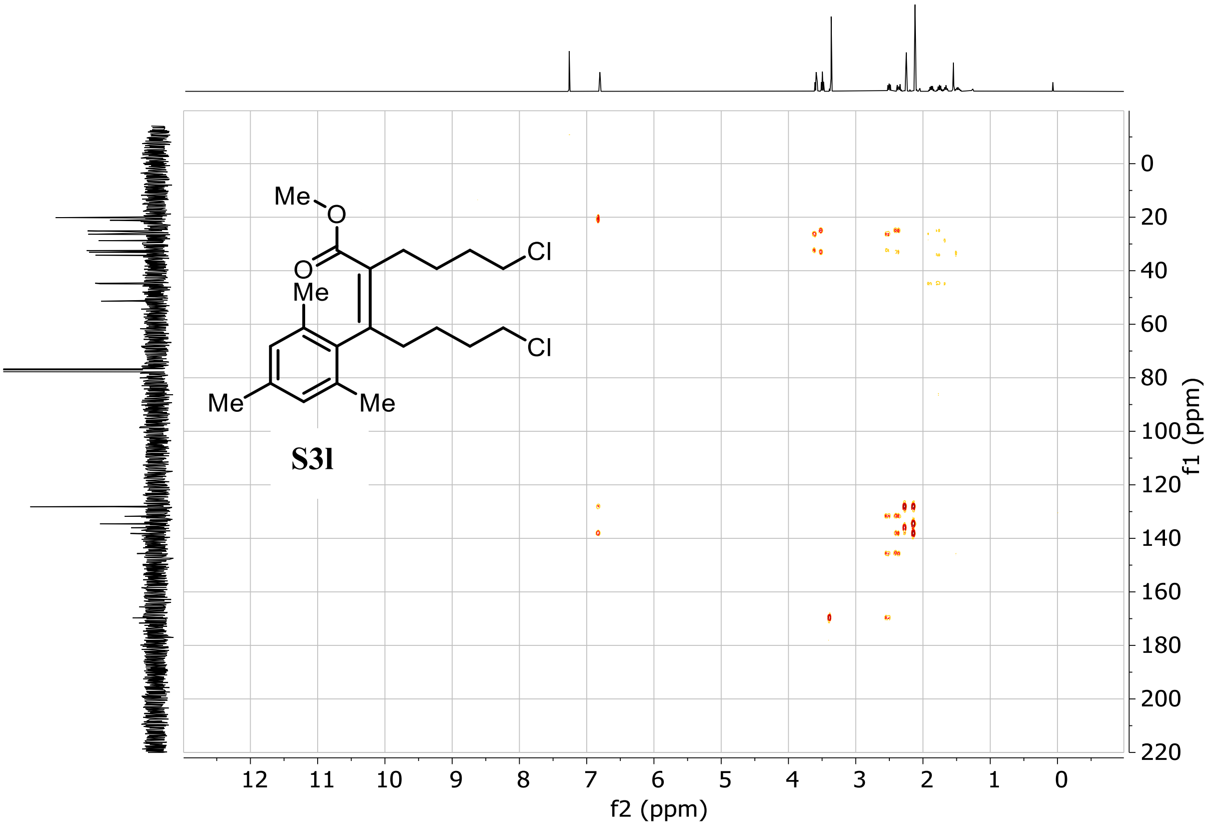

Me
O
O
Cl
Me
Cl
Me
Me
S3l
f2 (ppm)
f1 (ppm)

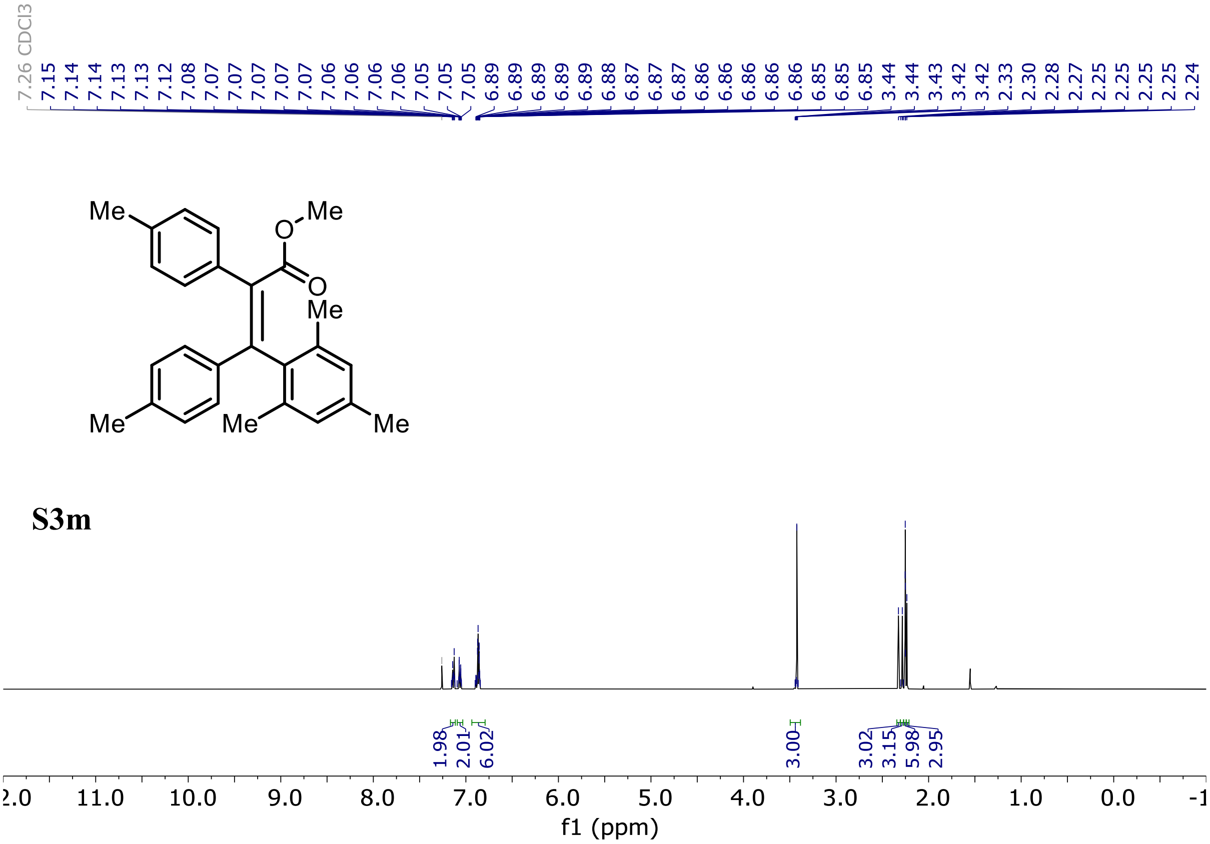
7.26 CDCl3
7.15 7.14 7.14 7.13 7.13 7.12 7.08 7.07 7.07 7.07 7.07 7.07 7.06 7.06 7.06 7.06 7.05 7.05 7.05 6.89 6.89 6.89 6.89 6.88 6.87 6.87 6.86 6.86 6.86 6.86 6.86 6.85 6.85 3.44 3.44 3.43 3.42 3.42 2.33 2.30 2.28 2.27 2.25 2.25 2.25 2.25 2.24
Me
O
Me
O
Me
Me
Me
Me
S3m
1.98
2.01
6.02
3.00
3.02
3.15
5.98
2.95
f1 (ppm)

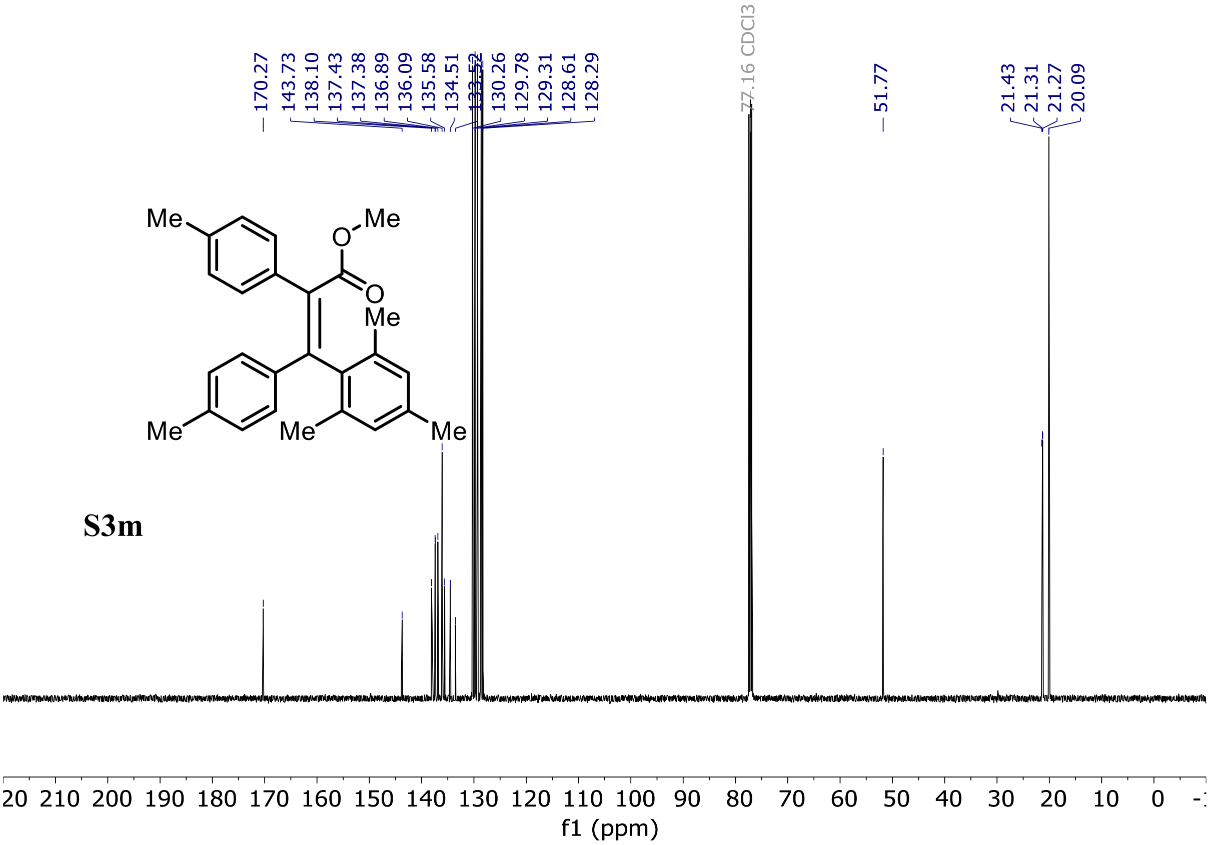
170.27
143.73 138.10 137.43 137.38 136.89 136.09 135.58 134.51 133.52 130.26 129.78 129.31 128.61 128.29
77.16 CDCl3
51.77
21.43 21.31 21.27 20.09
Me
O
Me
O
Me
Me
Me
Me
S3m
f1 (ppm)

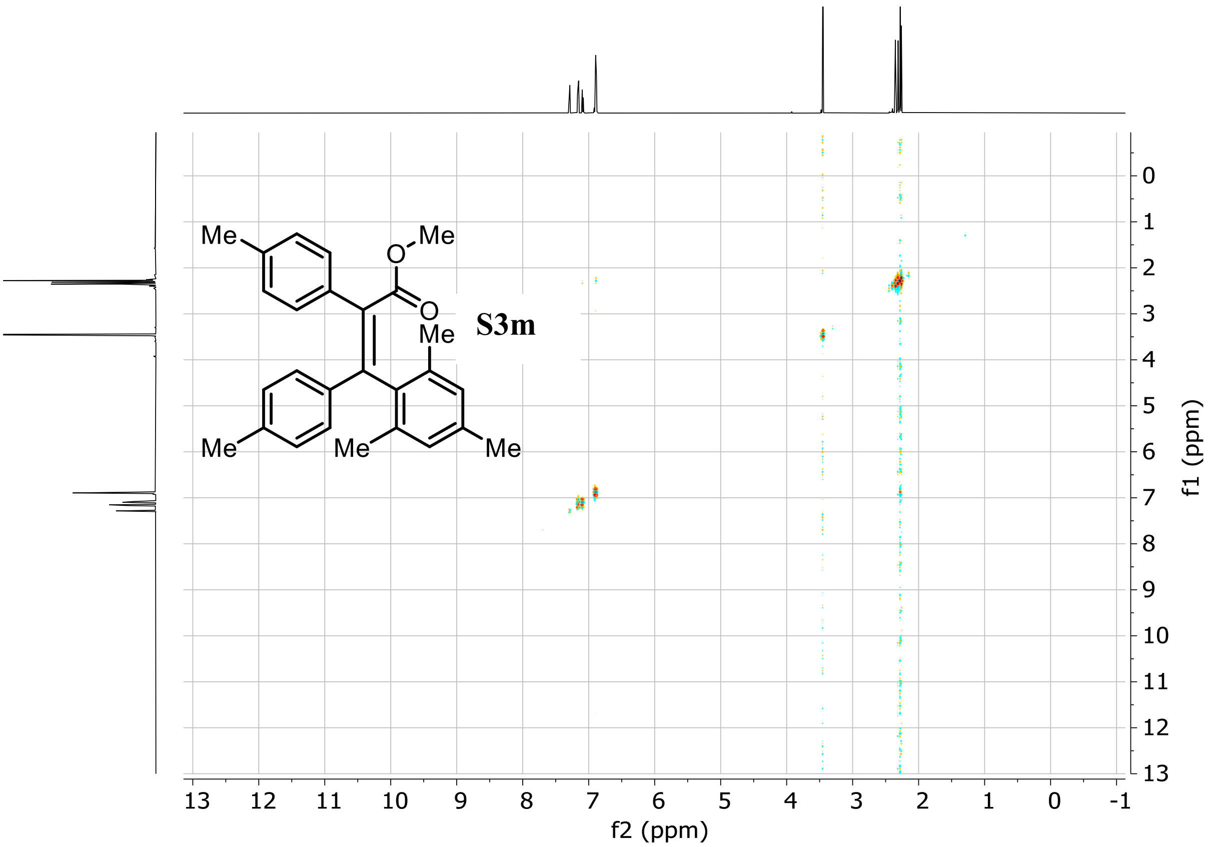
Me
O
Me
O
Me
S3m
Me
Me
Me
f1 (ppm)
f2 (ppm)

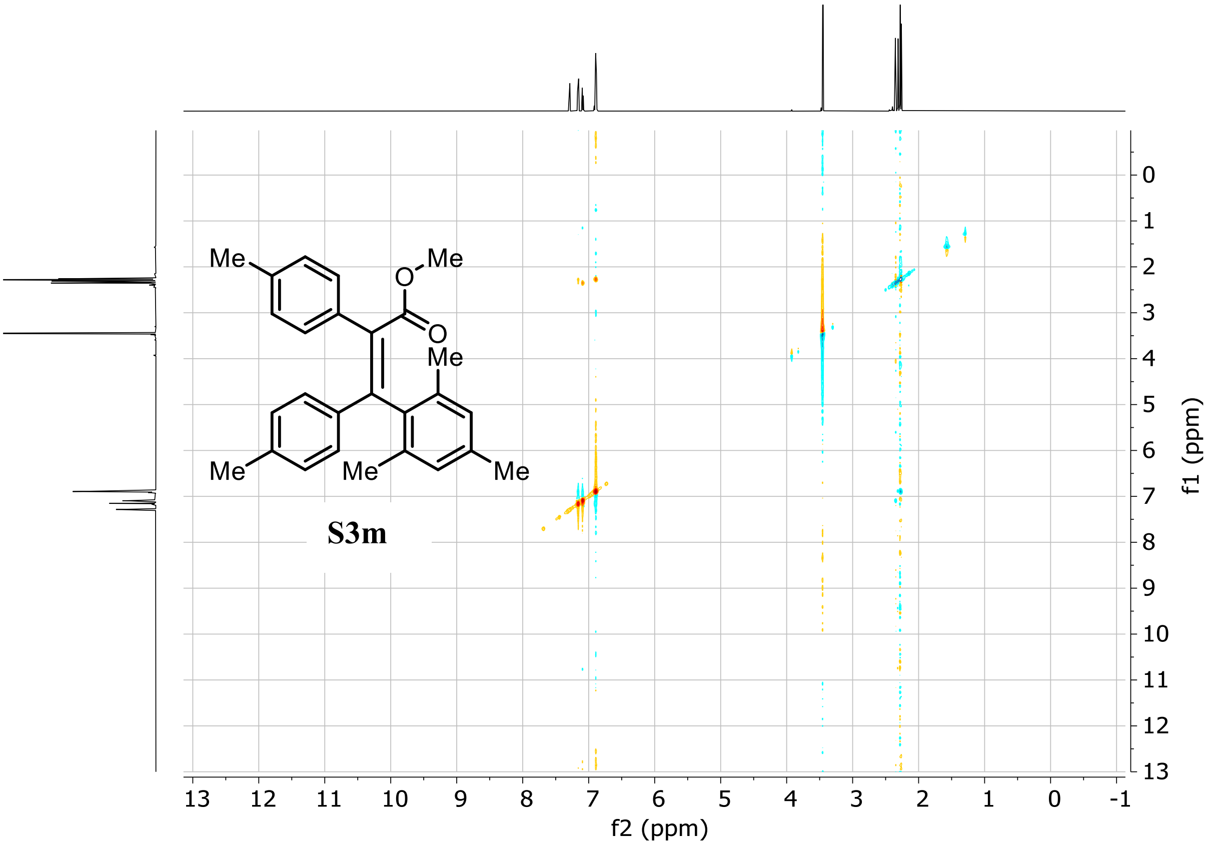
Me
O
Me
O
Me
Me
Me
Me
S3m
f1 (ppm)
f2 (ppm)

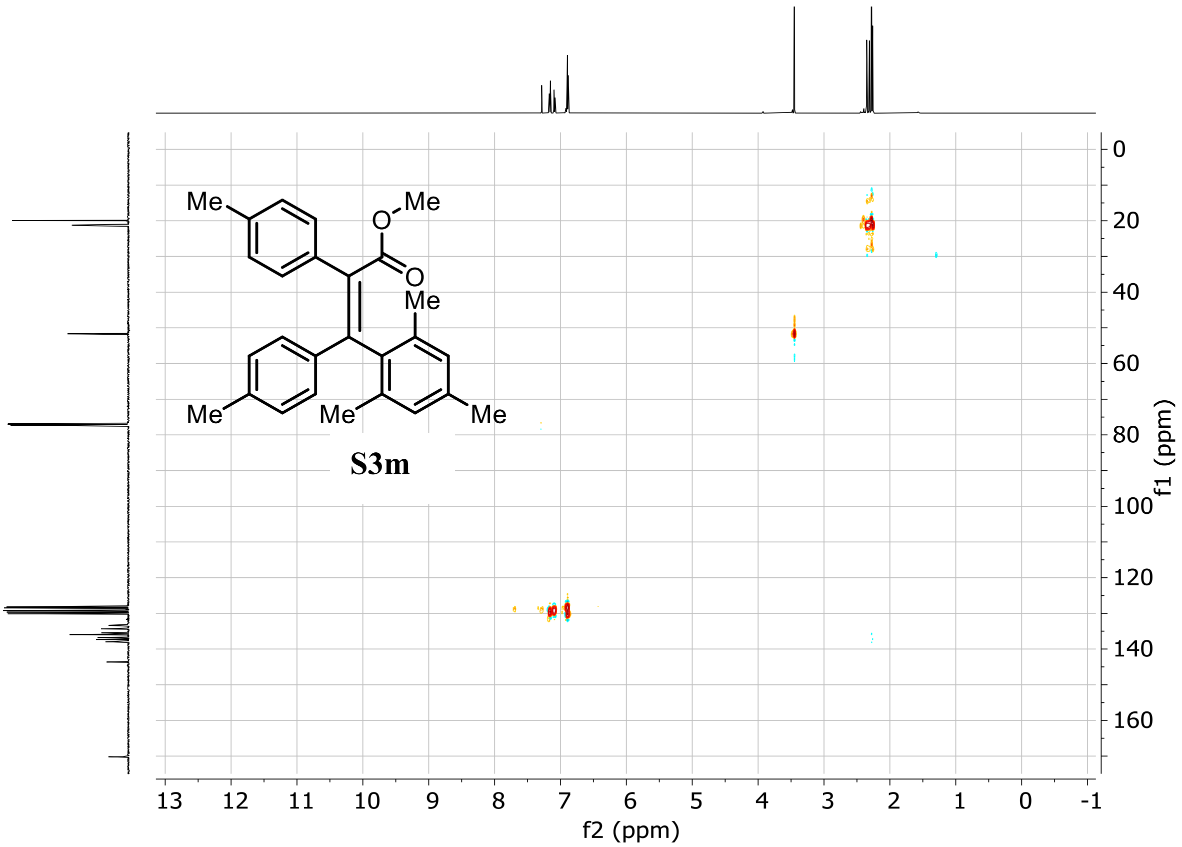
Me
O
Me
O
Me
Me
Me
Me
S3m
0
20
40
60
80
100
120
140
160
f1 (ppm)
13
12
11
10
9
8
7
6
5
4
3
2
1
0
-1
f2 (ppm)

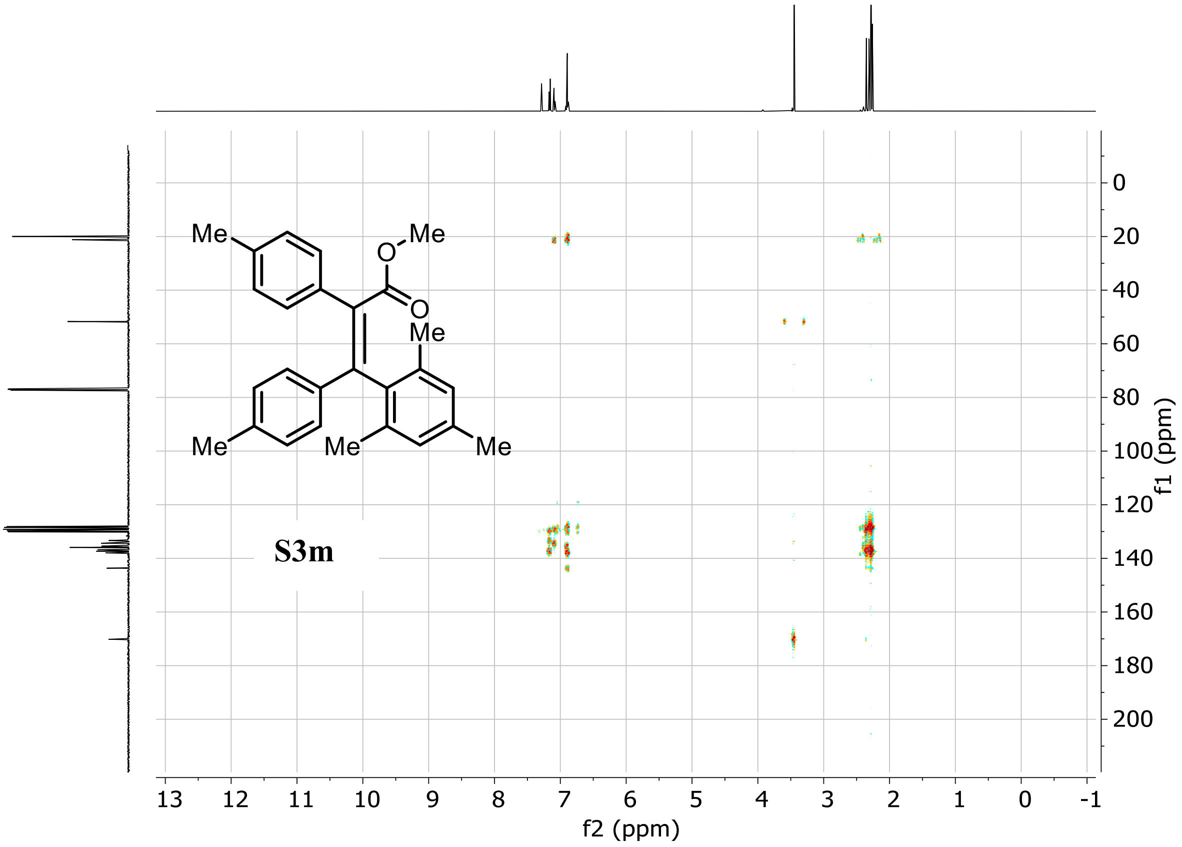
Me
O
Me
O
Me
Me
Me
Me
S3m
0
20
40
60
80
100
120
140
160
180
200
f1 (ppm)
13
12
11
10
9
8
7
6
5
4
3
2
1
0
-1
f2 (ppm)

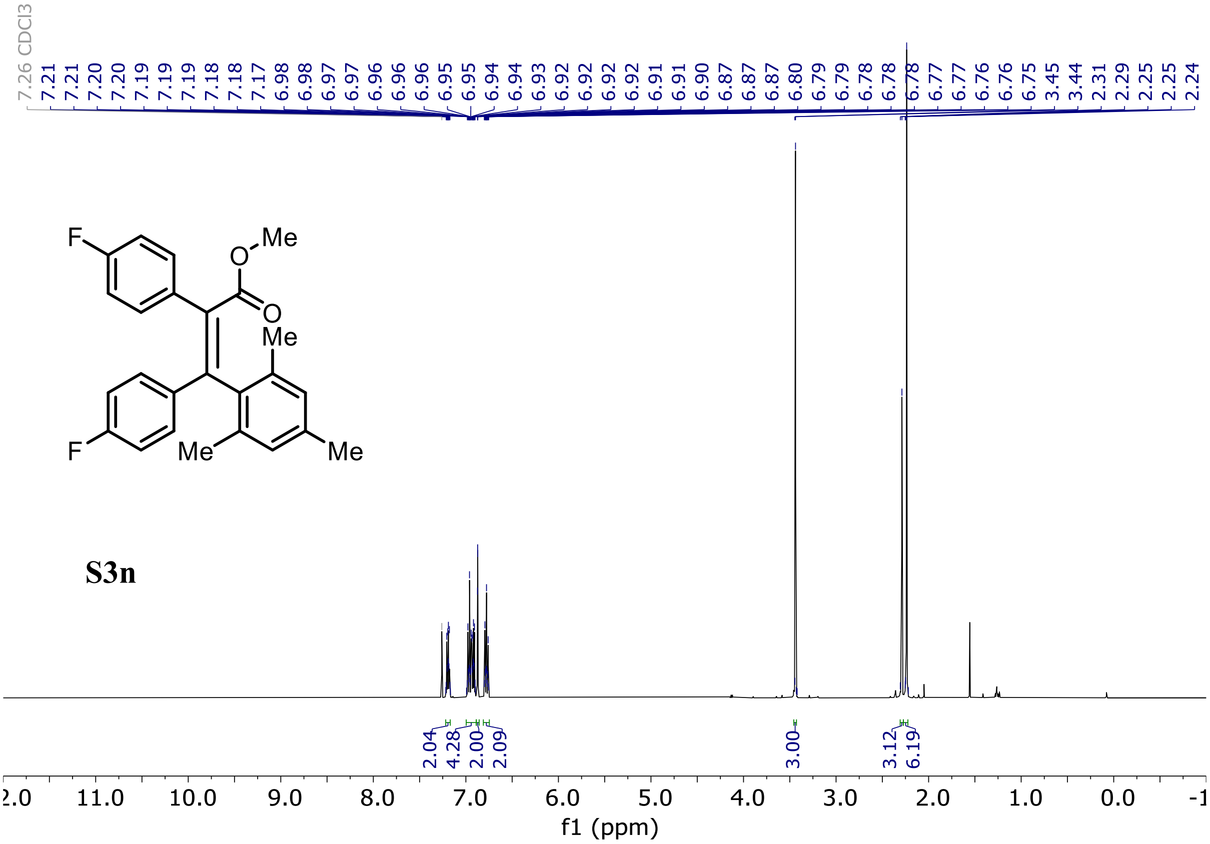

7.26 CDCl3
7.21 7.21 7.20 7.20 7.19 7.19 7.19 7.18 7.18 7.17 6.98 6.98 6.97 6.97 6.96 6.96 6.96 6.95 6.95 6.94 6.93 6.92 6.92 6.92 6.92 6.91 6.91 6.90 6.87 6.87 6.87 6.80 6.79 6.78 6.78 6.78 6.77 6.77 6.76 6.75 3.45 3.44 2.31 2.29 2.25 2.25 2.24
F
O
Me
O
Me
F
Me
Me
S3n
2.04 4.28 2.00 2.09 3.00 3.12 6.19
f1 (ppm)

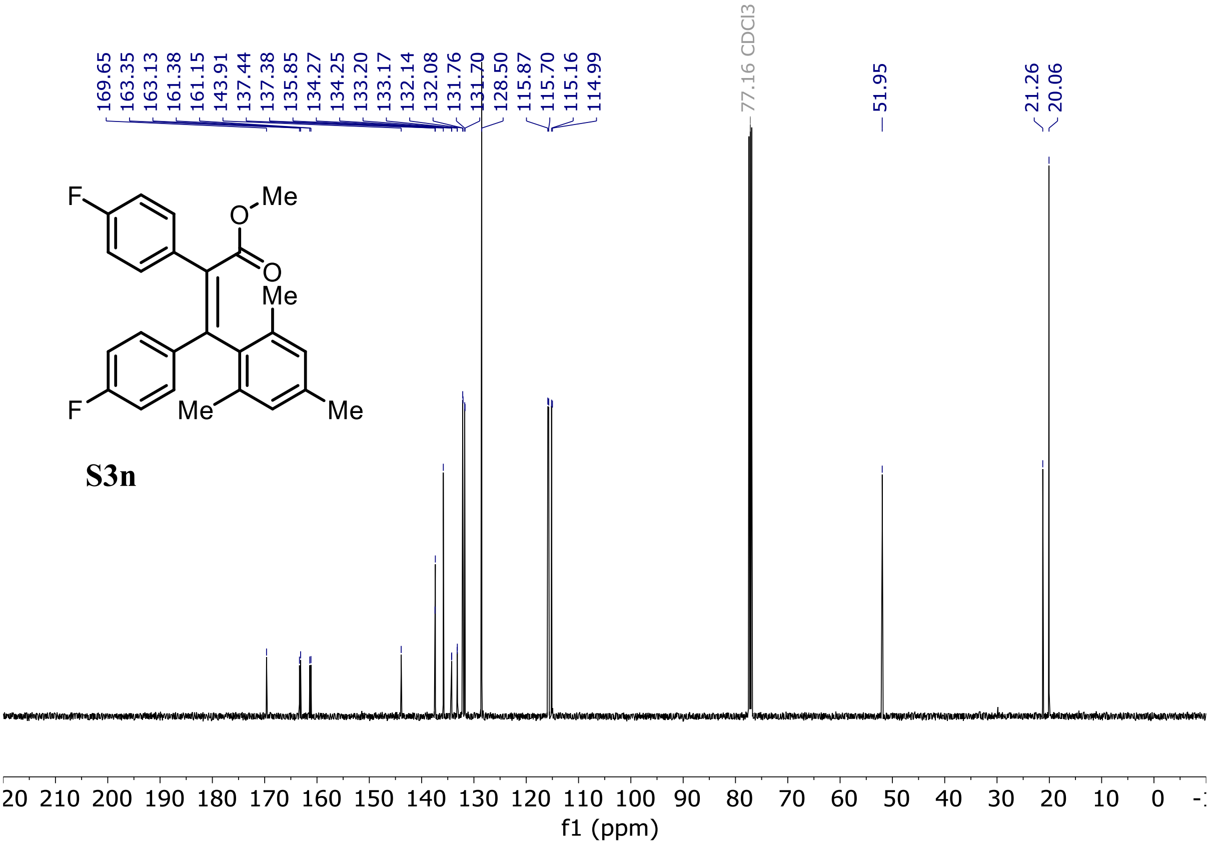

169.65 163.35 163.13 161.38 161.15 143.91 137.44 137.38 135.85 134.27 134.25 133.20 133.17 132.14 132.08 131.76 131.70 128.50 115.87 115.70 115.16 114.99
77.16 CDCl3
51.95
21.26 20.06
F
O
Me
O
Me
F
Me
Me
S3n
f1 (ppm)

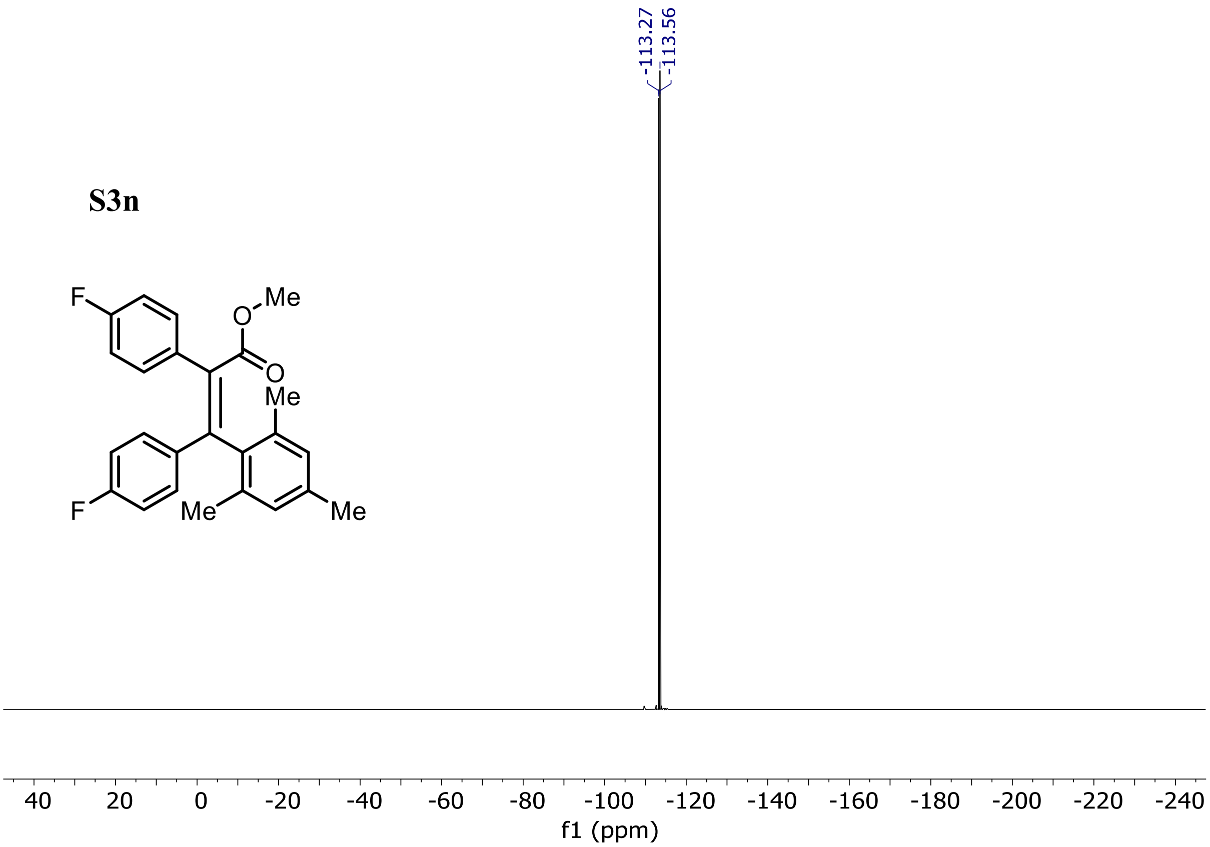
-113.27
-113.56
S3n
F
O
Me
O
Me
F
Me
Me
40 20 0 -20 -40 -60 -80 -100 -120 -140 -160 -180 -200 -220 -240
f1 (ppm)

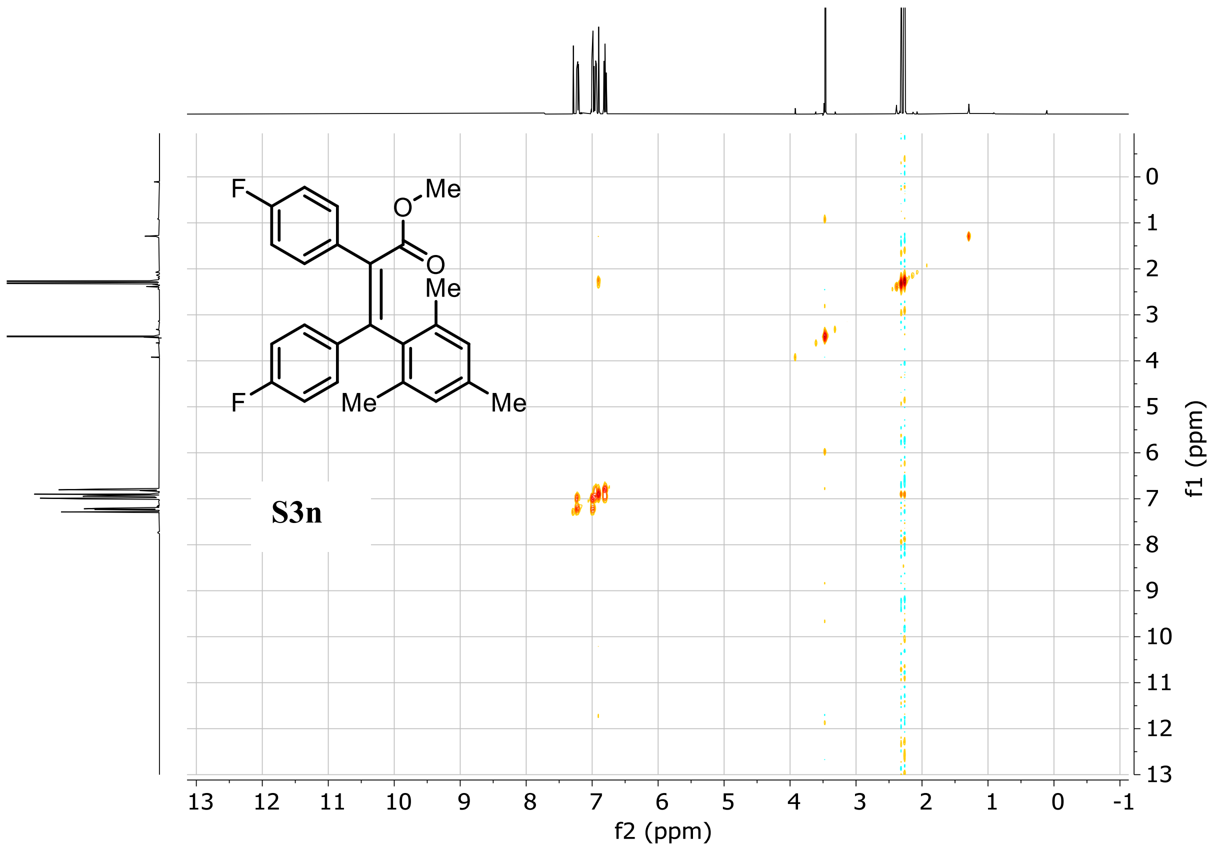
F
O
Me
O
Me
F
Me
Me
S3n
13 12 11 10 9 8 7 6 5 4 3 2 1 0 -1
f2 (ppm)
0 1 2 3 4 5 6 7 8 9 10 11 12 13
f1 (ppm)

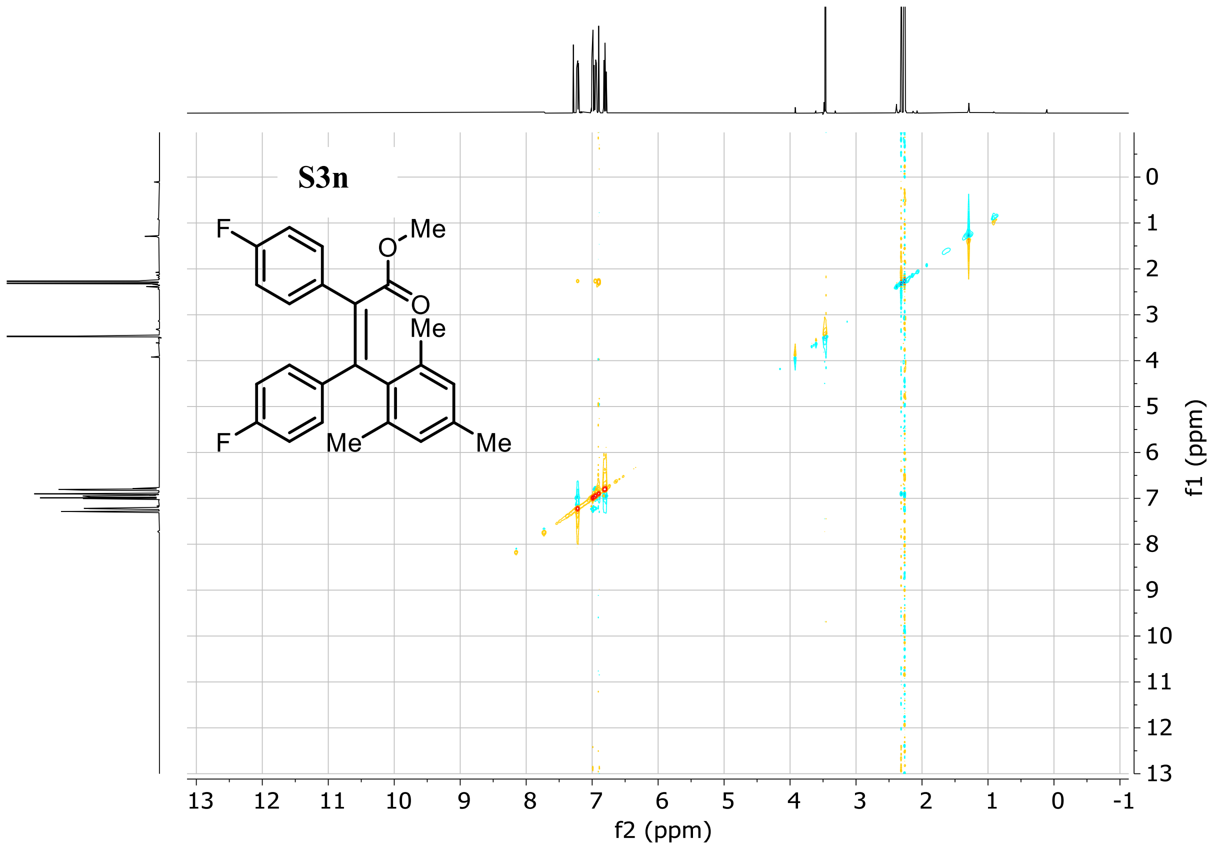
S3n
F
O
Me
O
Me
F
Me
Me
f2 (ppm)
f1 (ppm)

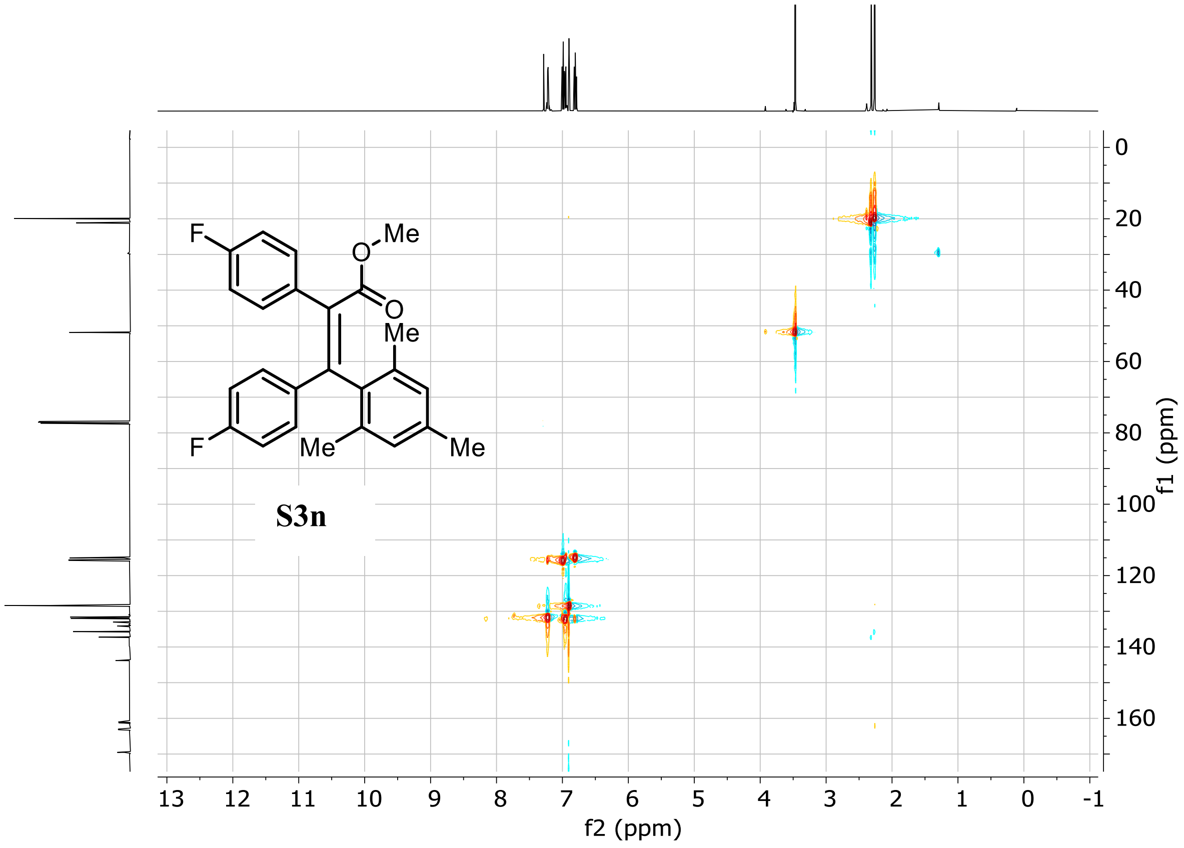
F
O
Me
O
Me
F
Me
Me
S3n
f2 (ppm)
f1 (ppm)

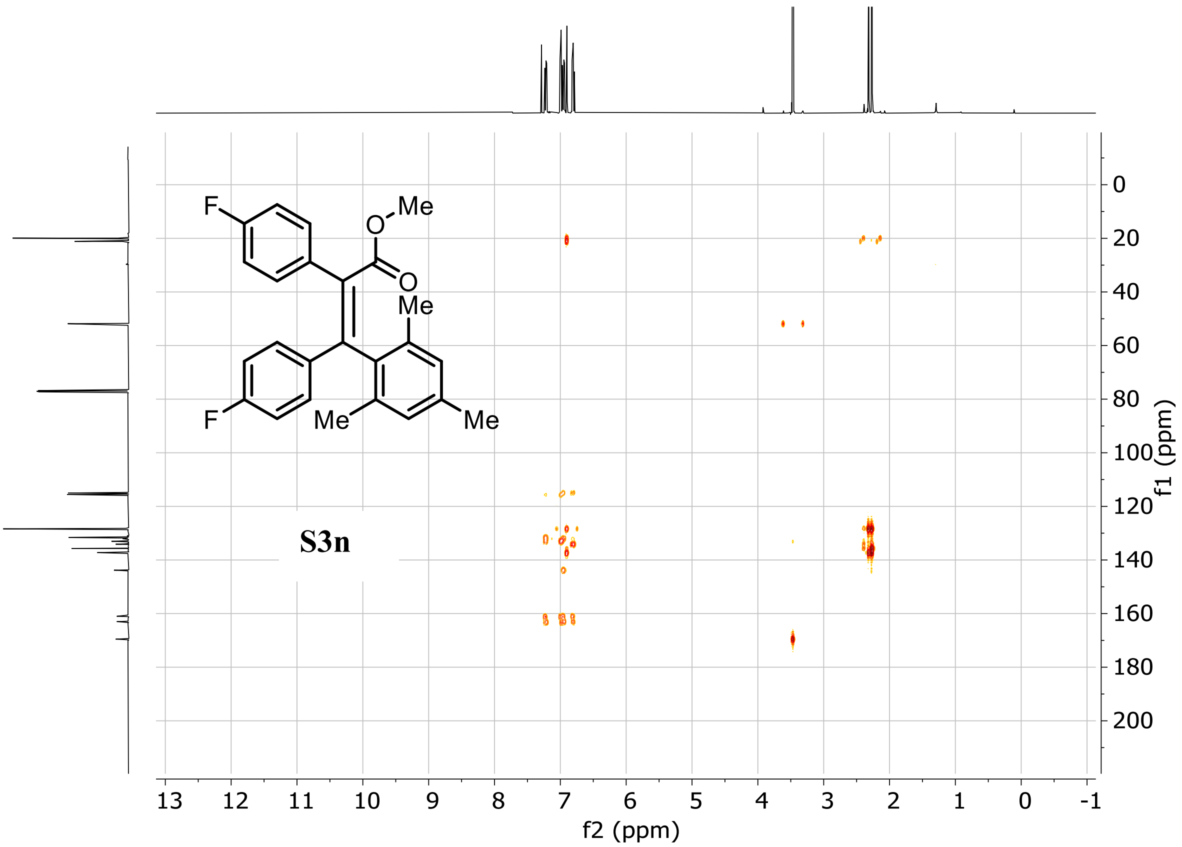

Cyclopentenones

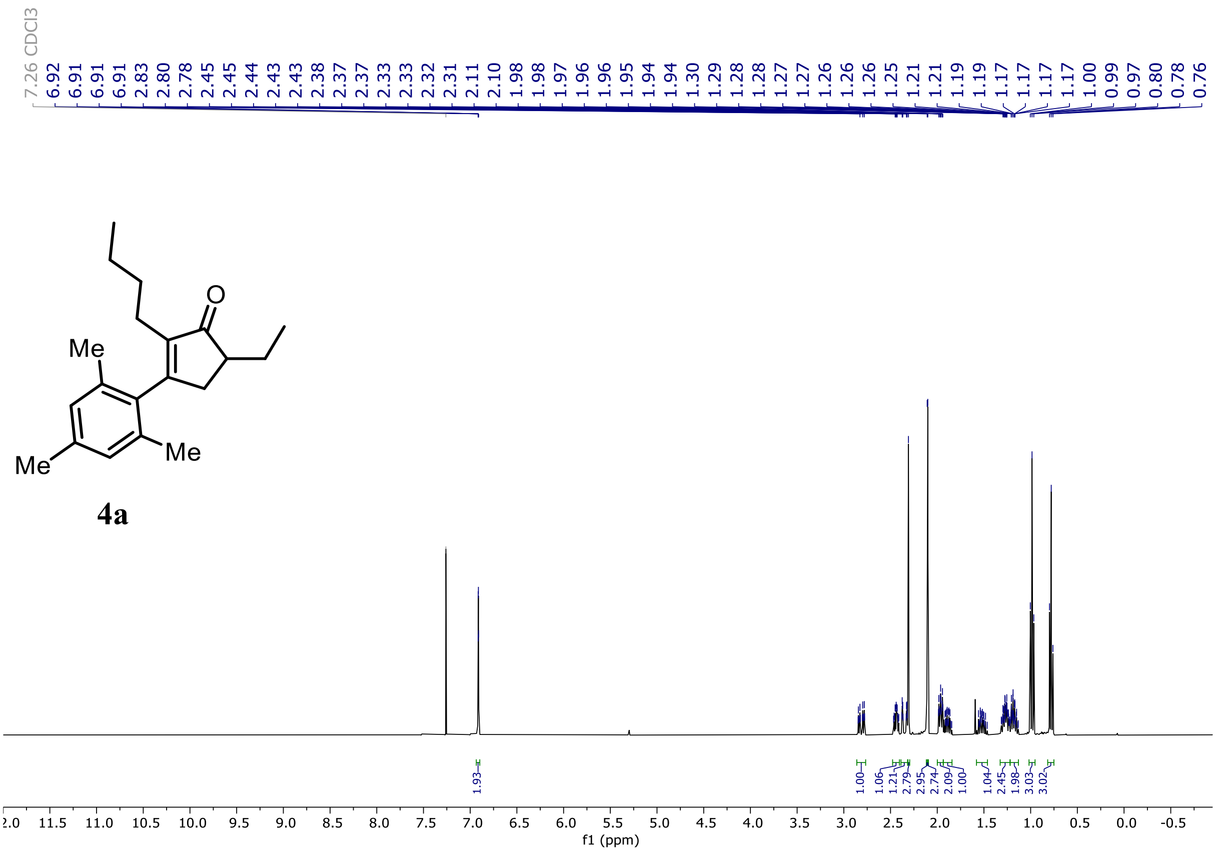

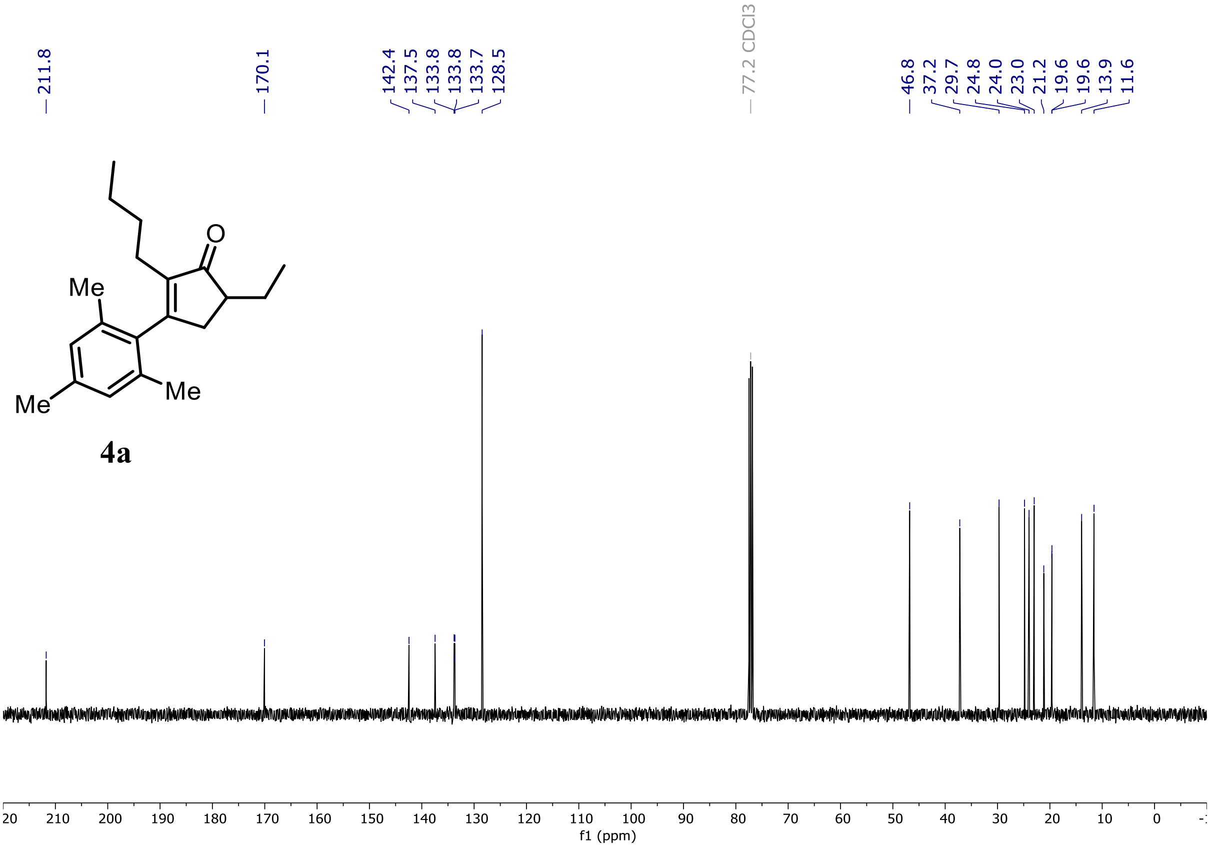

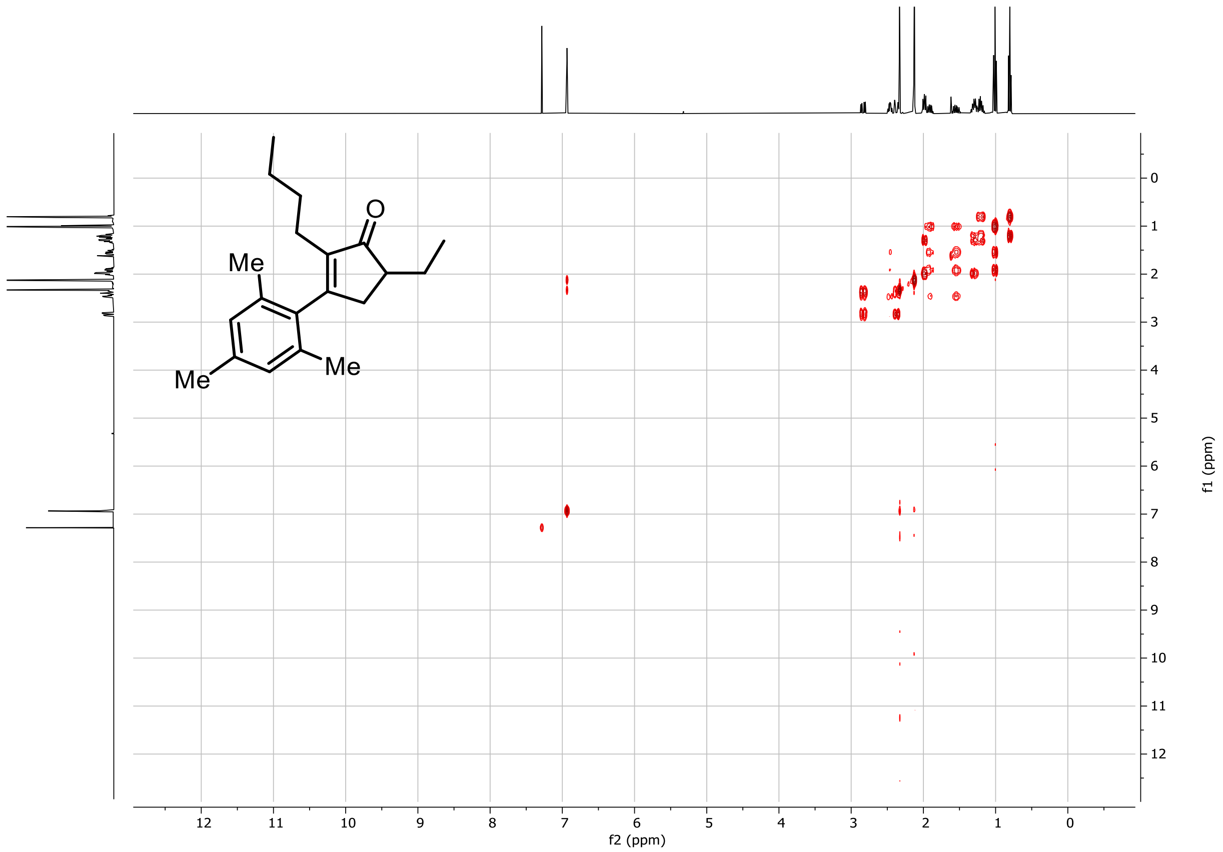
Me
Me
Me
O
f1 (ppm)
f2 (ppm)

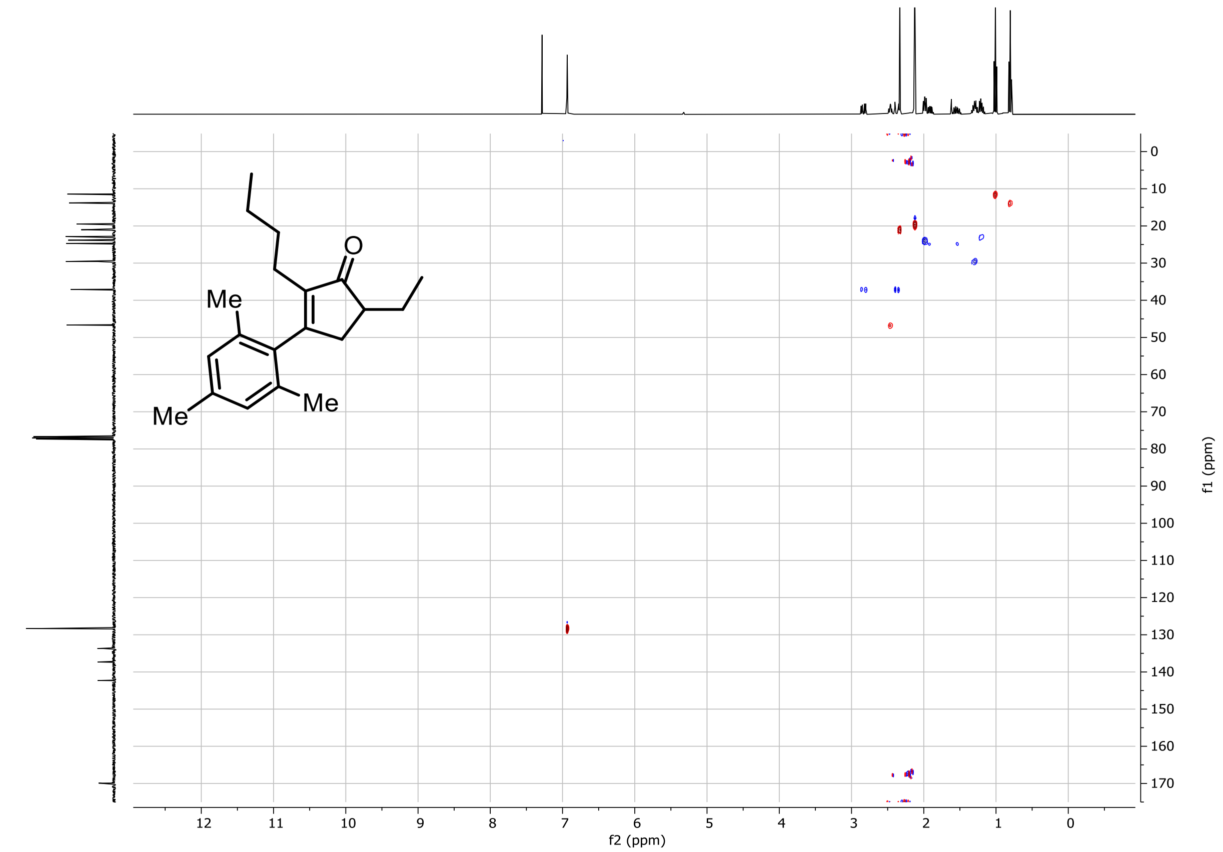
Me
Me
Me
O
f1 (ppm)
f2 (ppm)

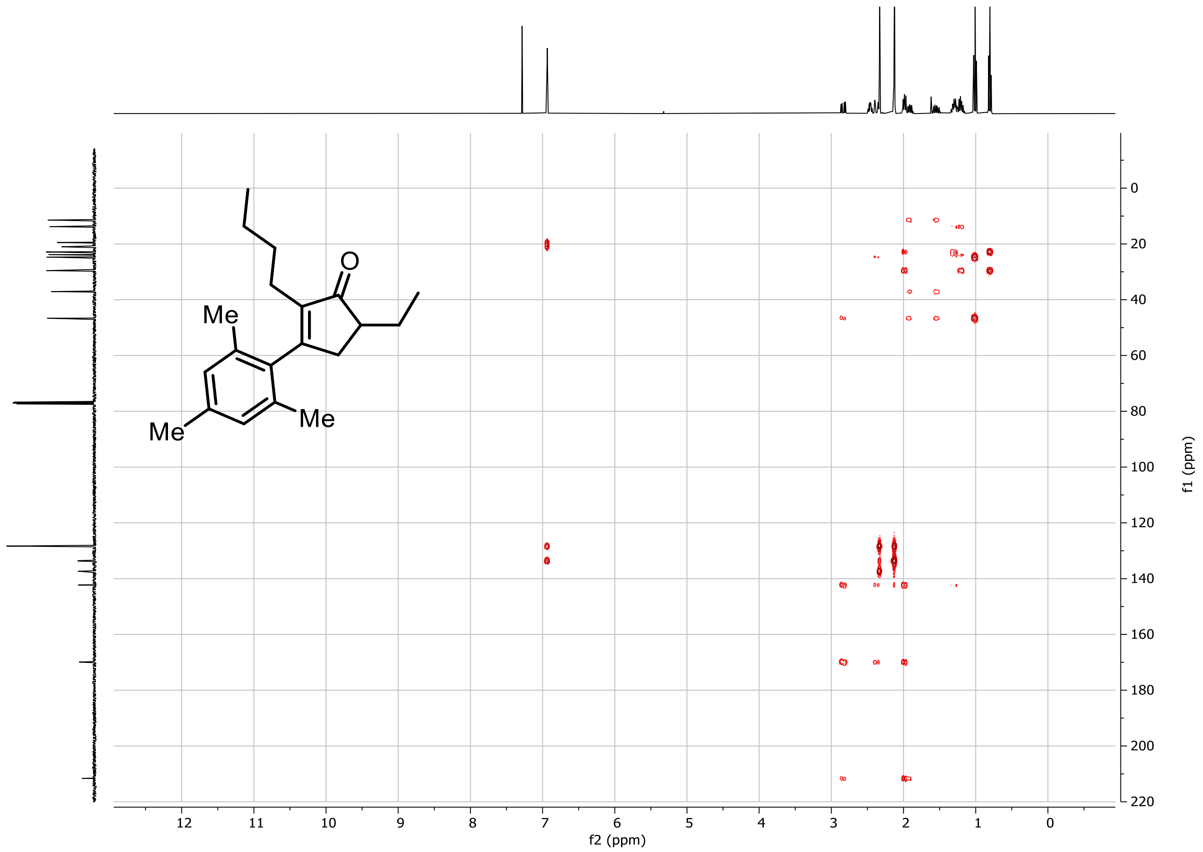

Me
Me
Me
O
f1 (ppm)
f2 (ppm)

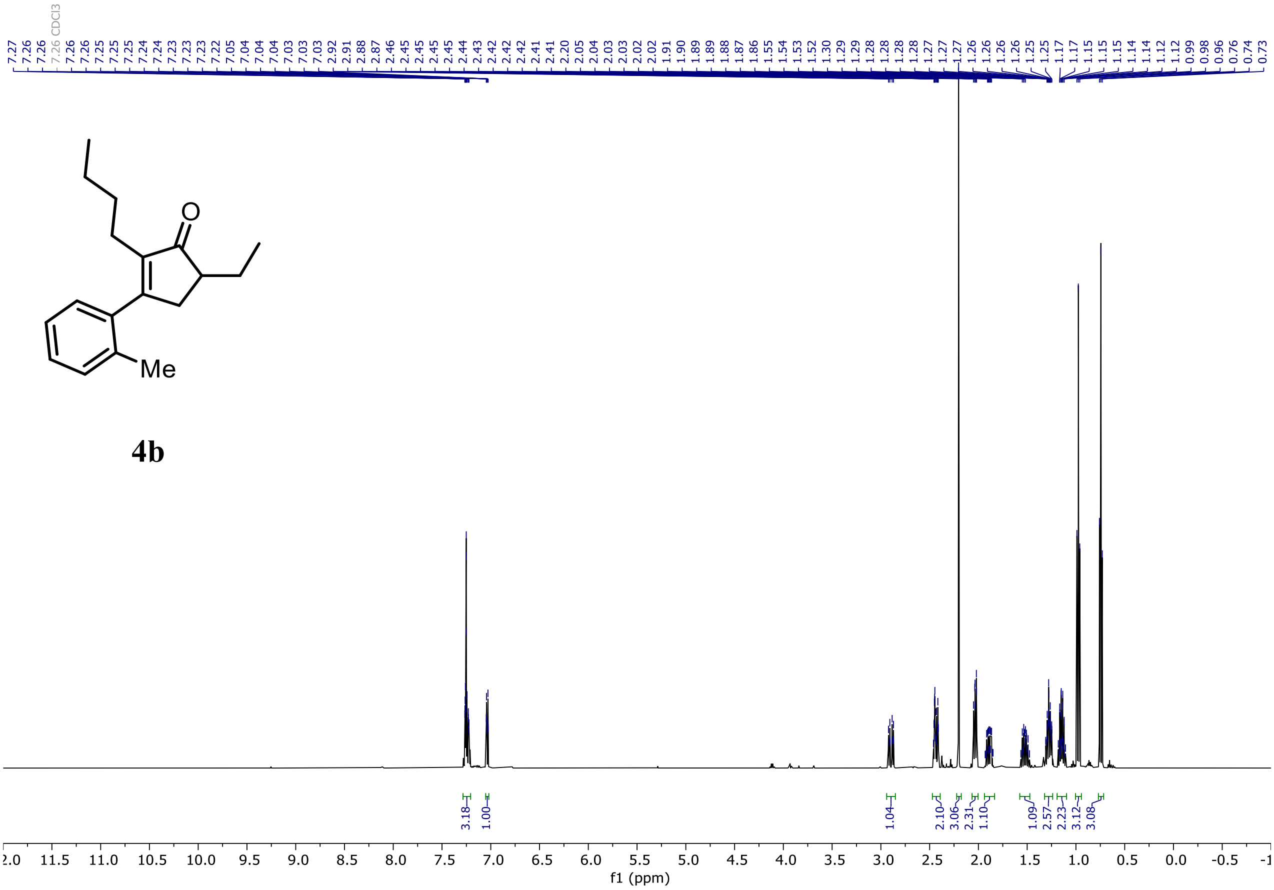

7.27 7.26 7.26 CDCl3 7.26 7.26 7.25 7.25 7.25 7.24 7.24 7.23 7.23 7.23 7.22 7.05 7.04 7.04 7.03 7.03 7.03 2.92 2.91 2.88 2.87 2.46 2.45 2.45 2.45 2.44 2.43 2.42 2.42 2.41 2.41 2.20 2.05 2.04 2.03 2.03 2.02 2.02 1.91 1.90 1.89 1.88 1.87 1.86 1.55 1.54 1.54 1.53 1.52 1.30 1.29 1.29 1.28 1.28 1.28 1.28 1.27 1.27 1.27 1.26 1.26 1.26 1.26 1.25 1.25 1.17 1.17 1.15 1.15 1.15 1.14 1.12 0.99 0.96 0.76 0.74 0.73
O
Me
4b
3.18 1.00 1.04 2.10 3.06 2.31 1.10 1.09 2.57 2.23 3.12 3.08
f1 (ppm)

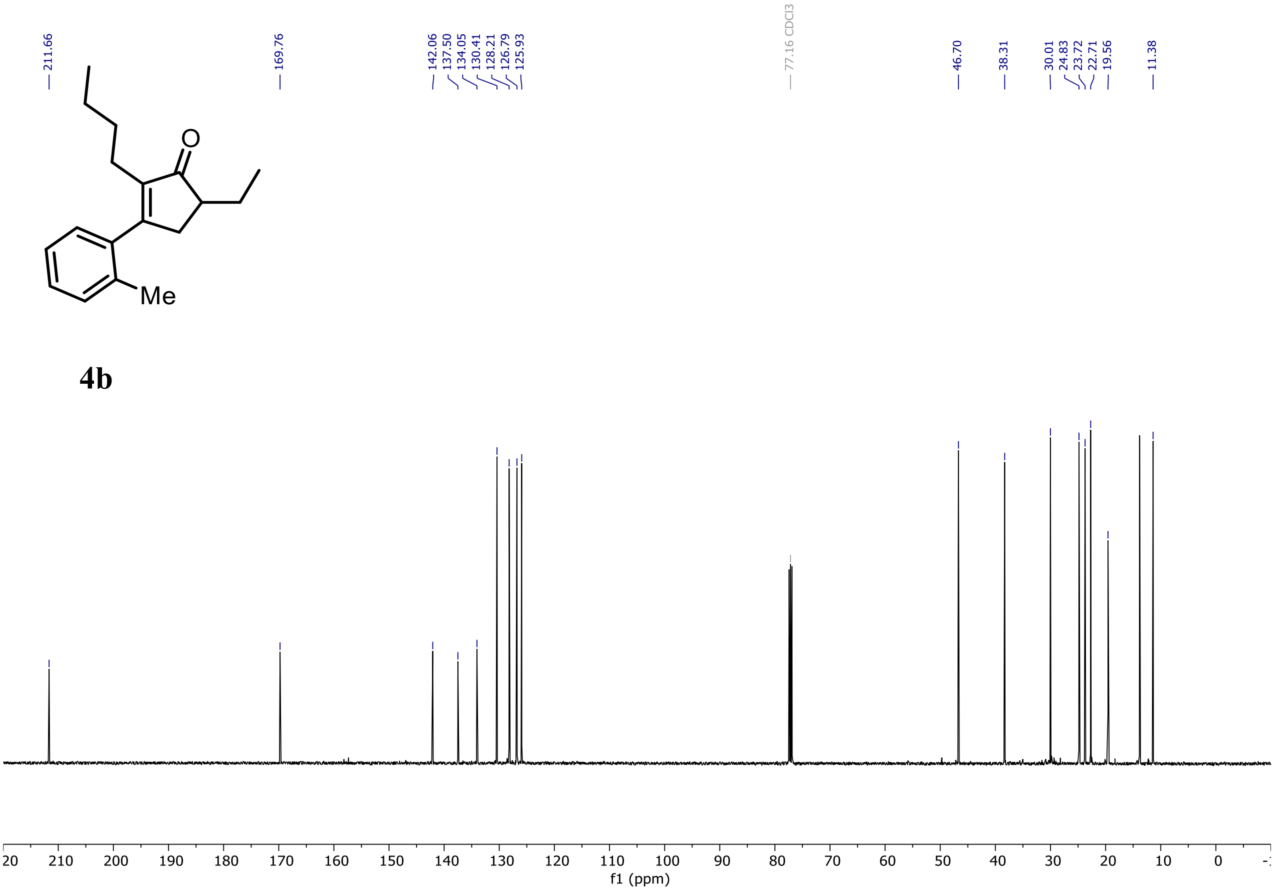

211.66
169.76
142.06 137.50 134.05 130.41 128.21 126.79 125.93
77.16 CDCl3
46.70
38.31
30.01 24.83 23.72 22.71 19.56
11.38
O
Me
4b
f1 (ppm)

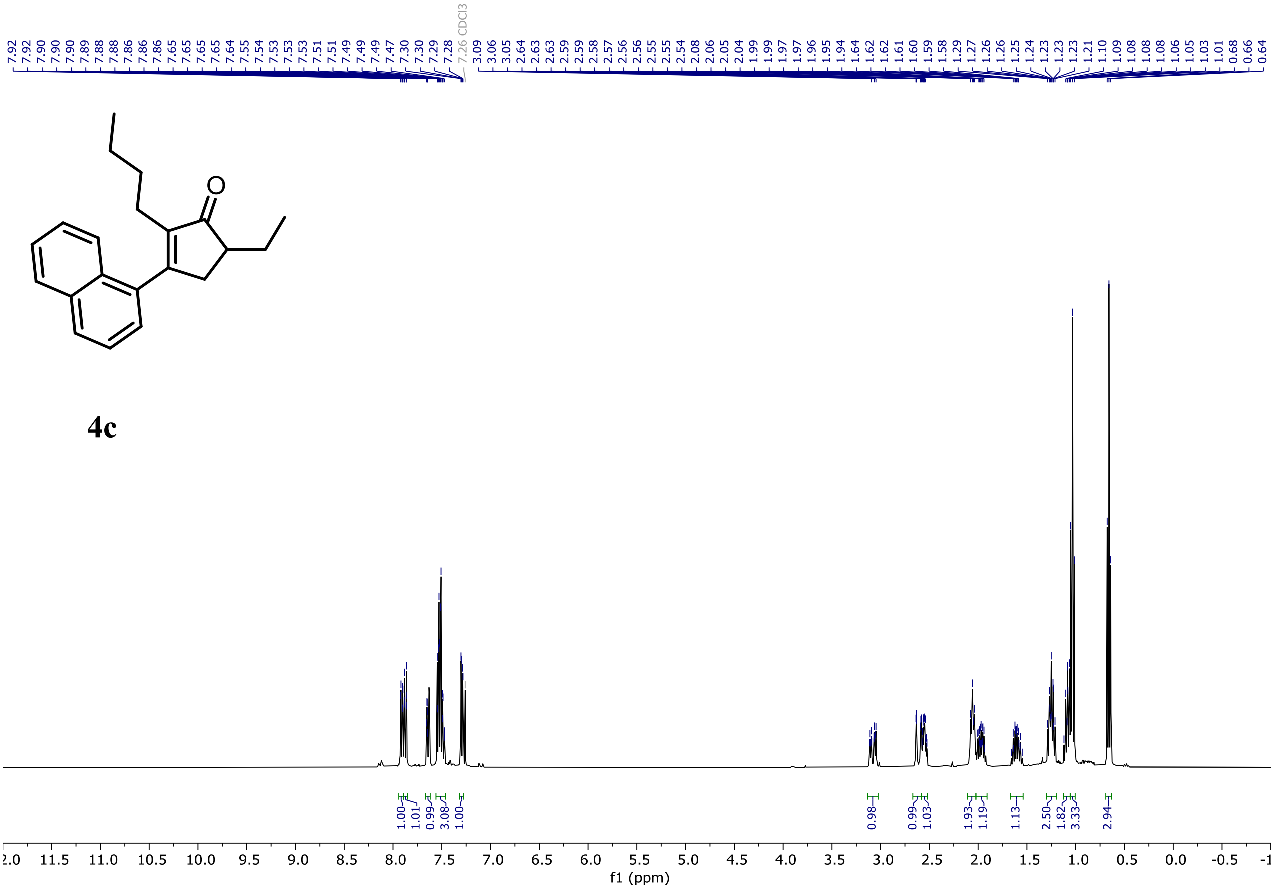

4c

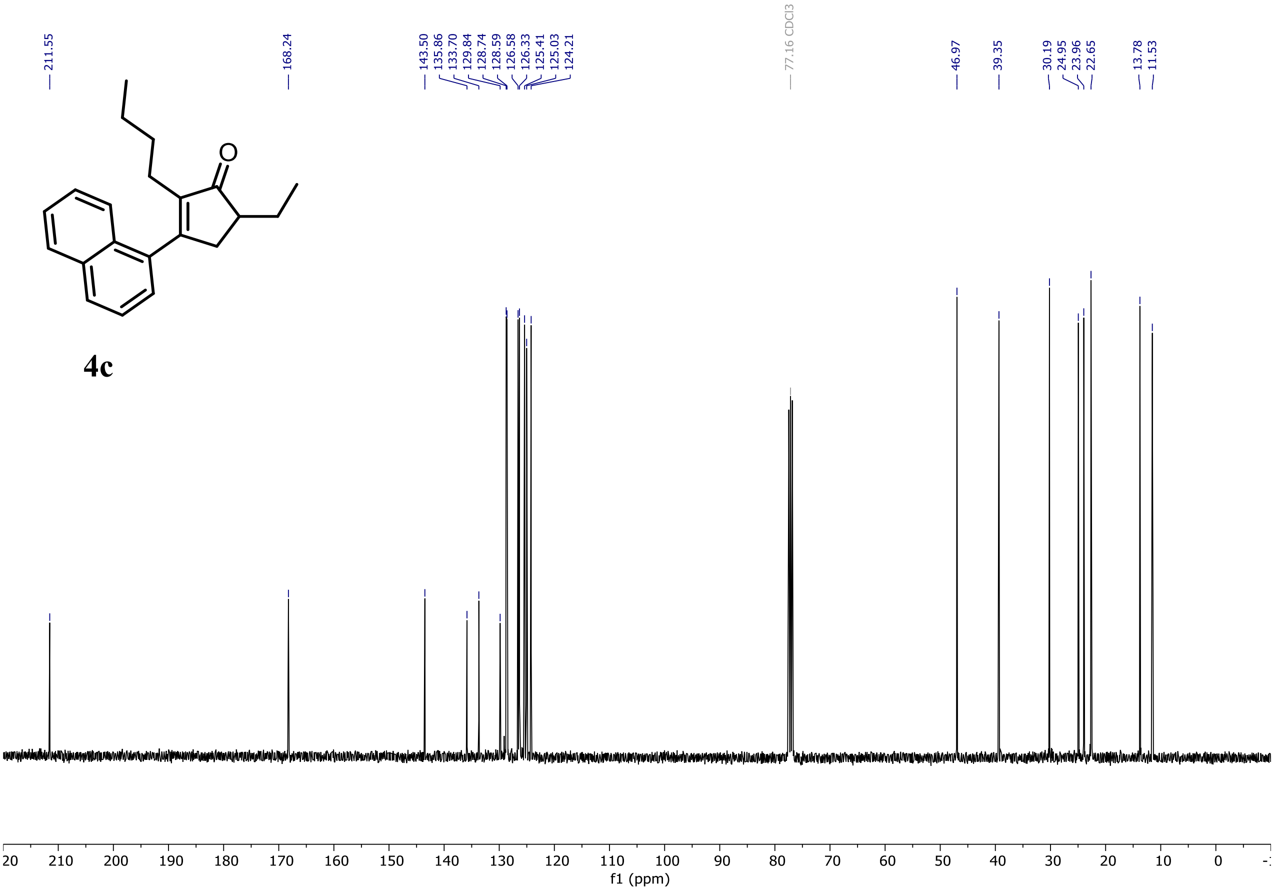

4c

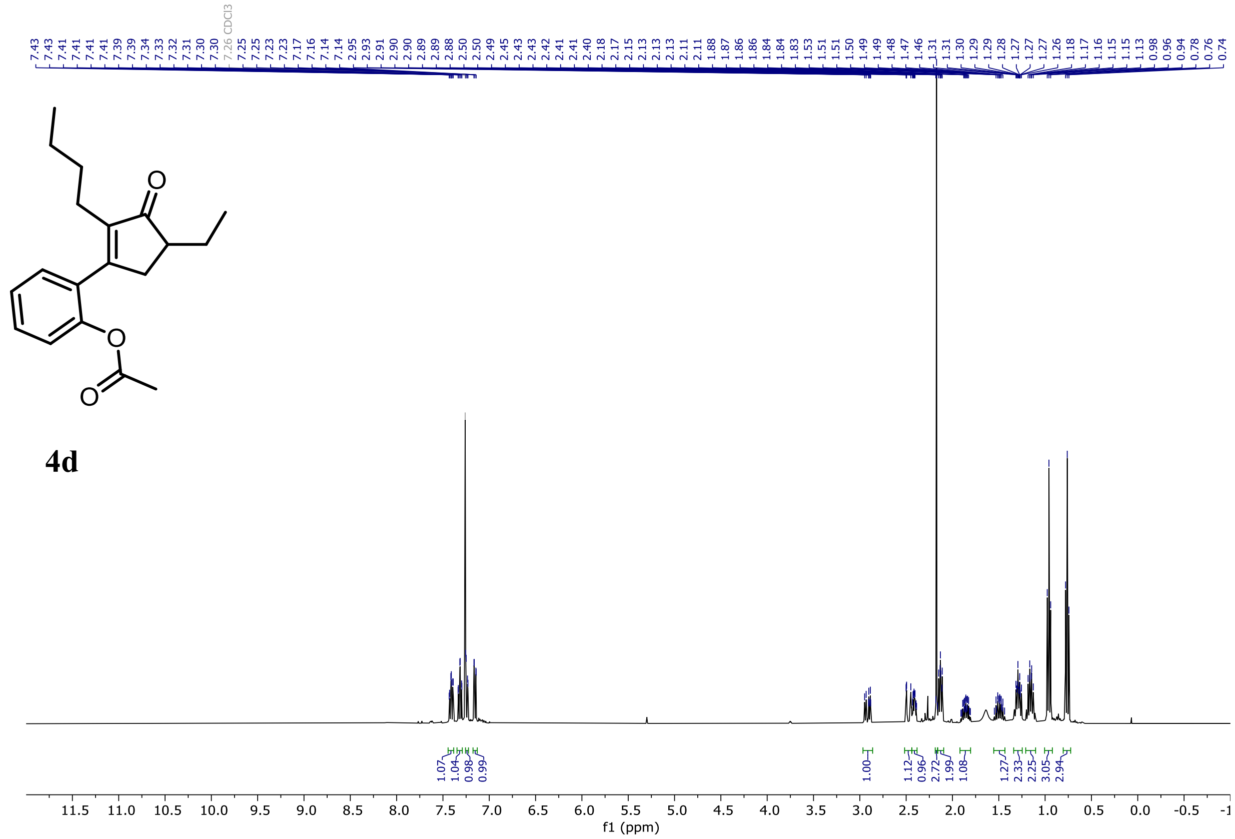
7.43 7.43 7.41 7.41 7.41 7.39 7.39 7.34 7.33 7.32 7.31 7.30 7.30 7.26 CDCl3 7.25 7.25 7.23 7.23 7.17 7.16 7.14 7.14 2.95 2.93 2.91 2.90 2.90 2.89 2.88 2.50 2.50 2.49 2.45 2.43 2.43 2.42 2.41 2.41 2.40 2.18 2.17 2.15 2.13 2.13 2.13 2.11 2.11 1.88 1.87 1.86 1.86 1.84 1.84 1.83 1.53 1.51 1.51 1.50 1.49 1.49 1.48 1.47 1.46 1.31 1.31 1.30 1.29 1.29 1.28 1.27 1.27 1.27 1.26 1.18 1.17 1.16 1.15 1.15 1.13 0.98 0.96 0.94 0.78 0.76 0.74
4d
1.07 1.04 0.98 0.99 1.00 1.12 0.96 2.72 1.99 1.08 1.27 2.33 2.25 3.05 2.94
11.5 11.0 10.5 10.0 9.5 9.0 8.5 8.0 7.5 7.0 6.5 6.0 5.5 5.0 4.5 4.0 3.5 3.0 2.5 2.0 1.5 1.0 0.5 0.0 -0.5 -1
f1 (ppm)

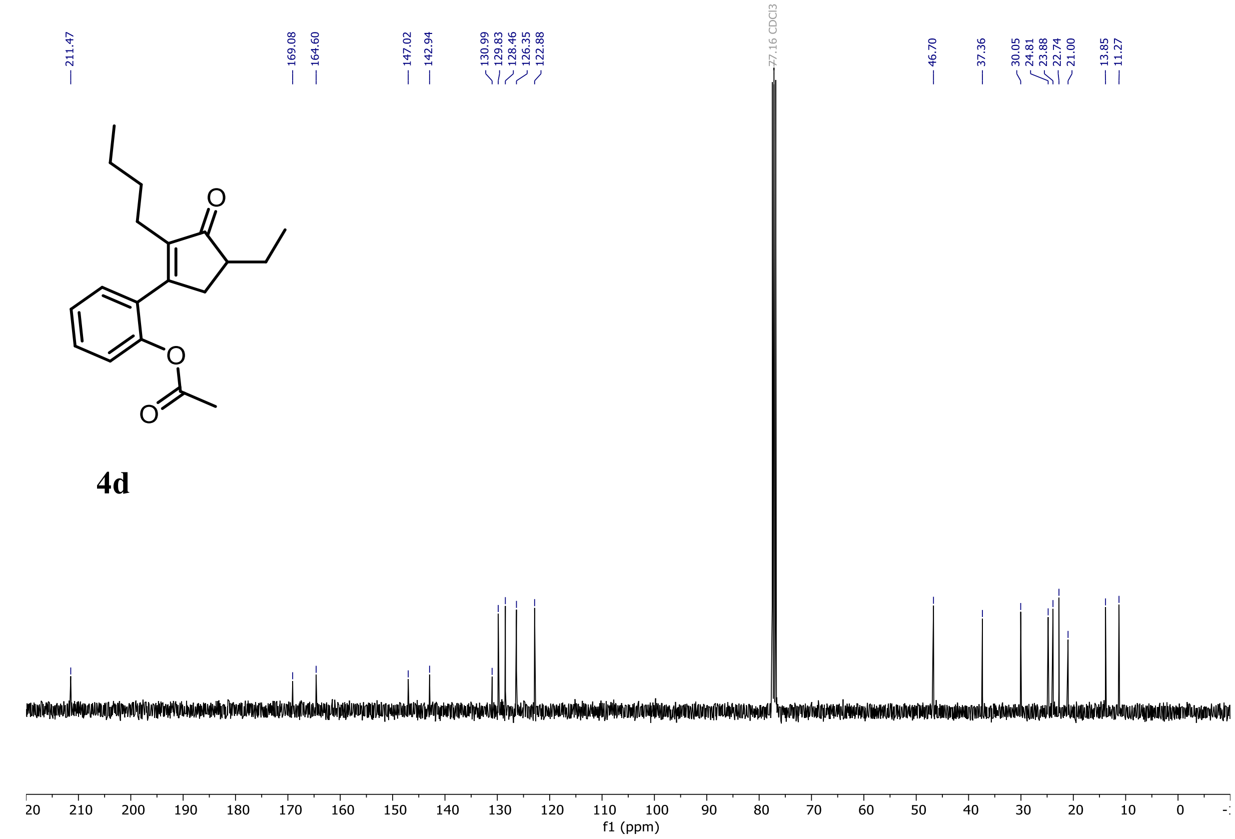
211.47
169.08 164.60
147.02 142.94
130.99 129.83 128.46 126.35 122.88
77.16 CDCl3
46.70 37.36 30.05 24.81 23.88 22.74 21.00 13.85 11.27
4d
20 210 200 190 180 170 160 150 140 130 120 110 100 90 80 70 60 50 40 30 20 10 0
f1 (ppm)

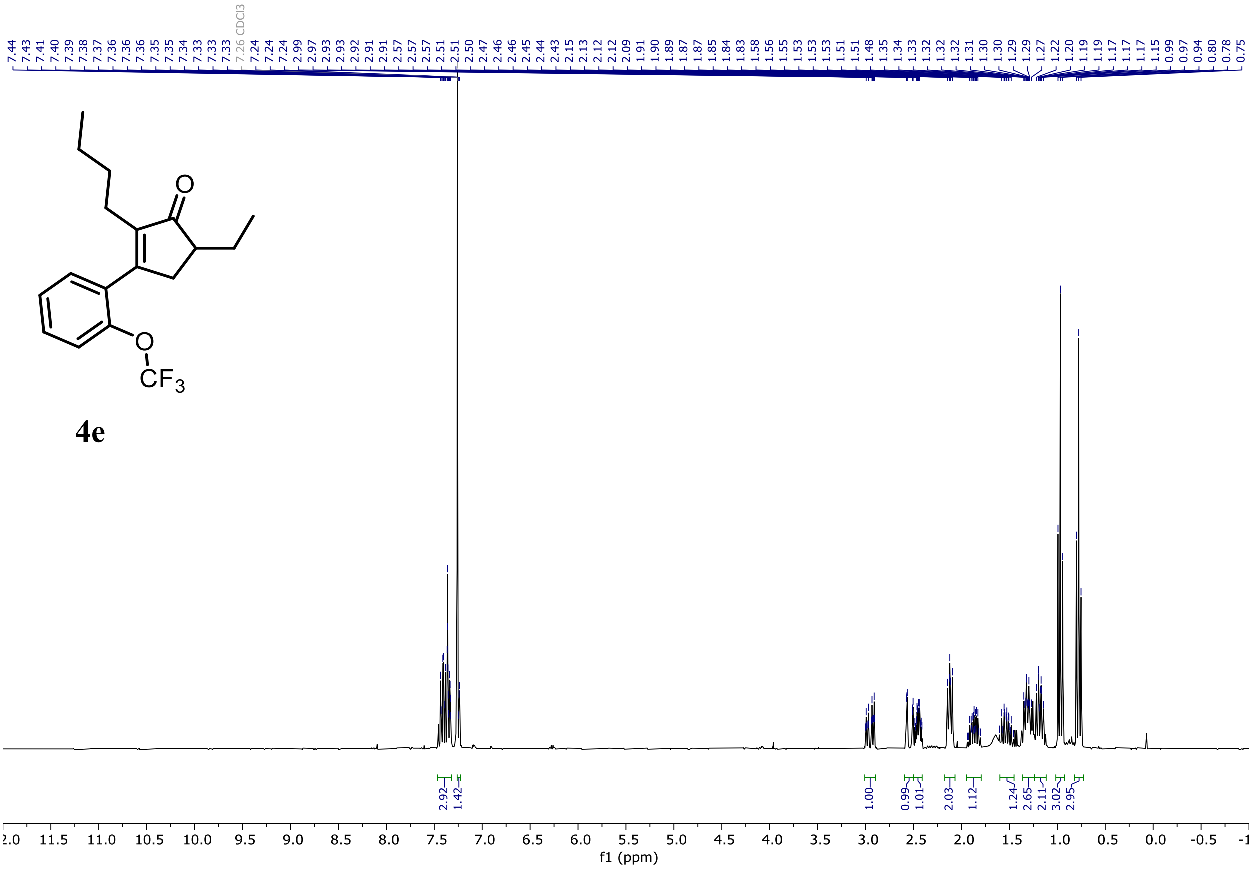

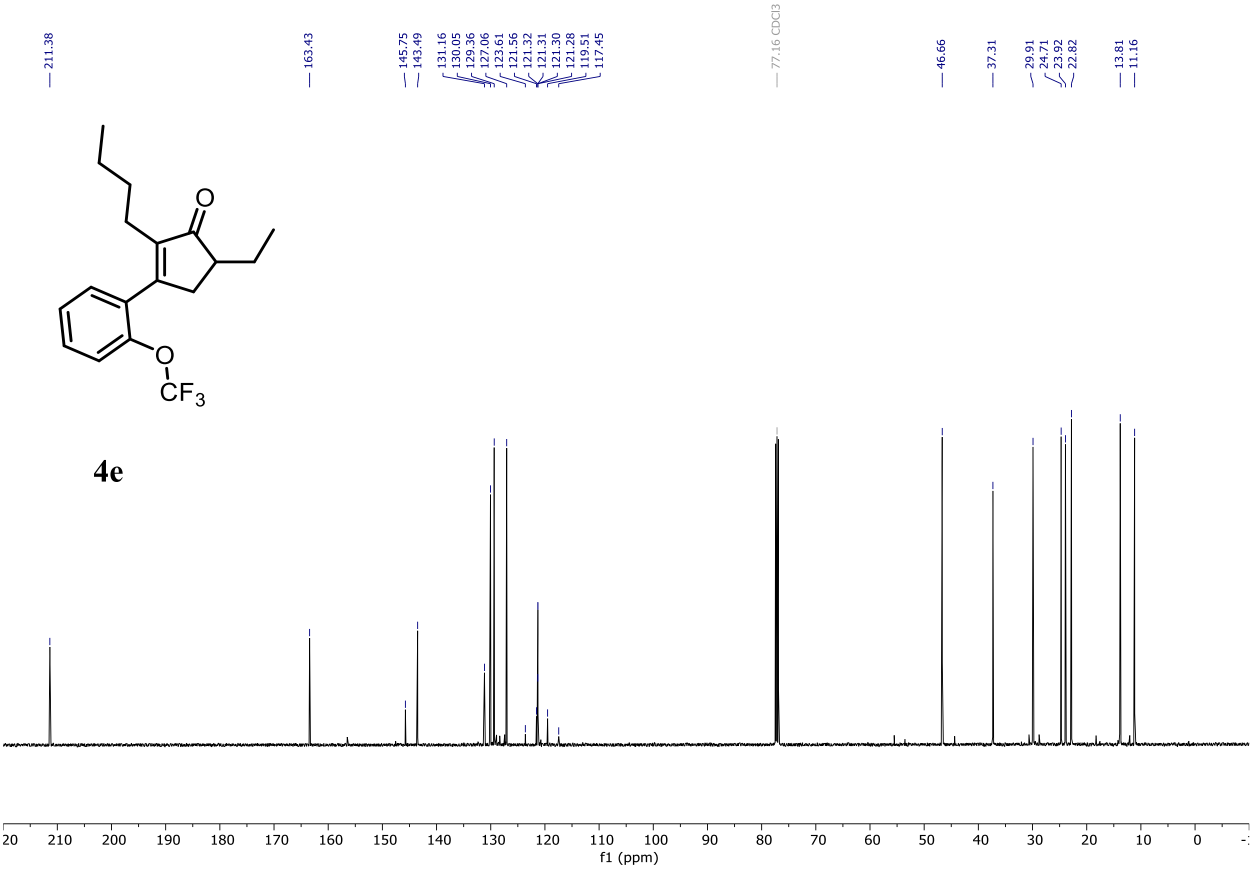

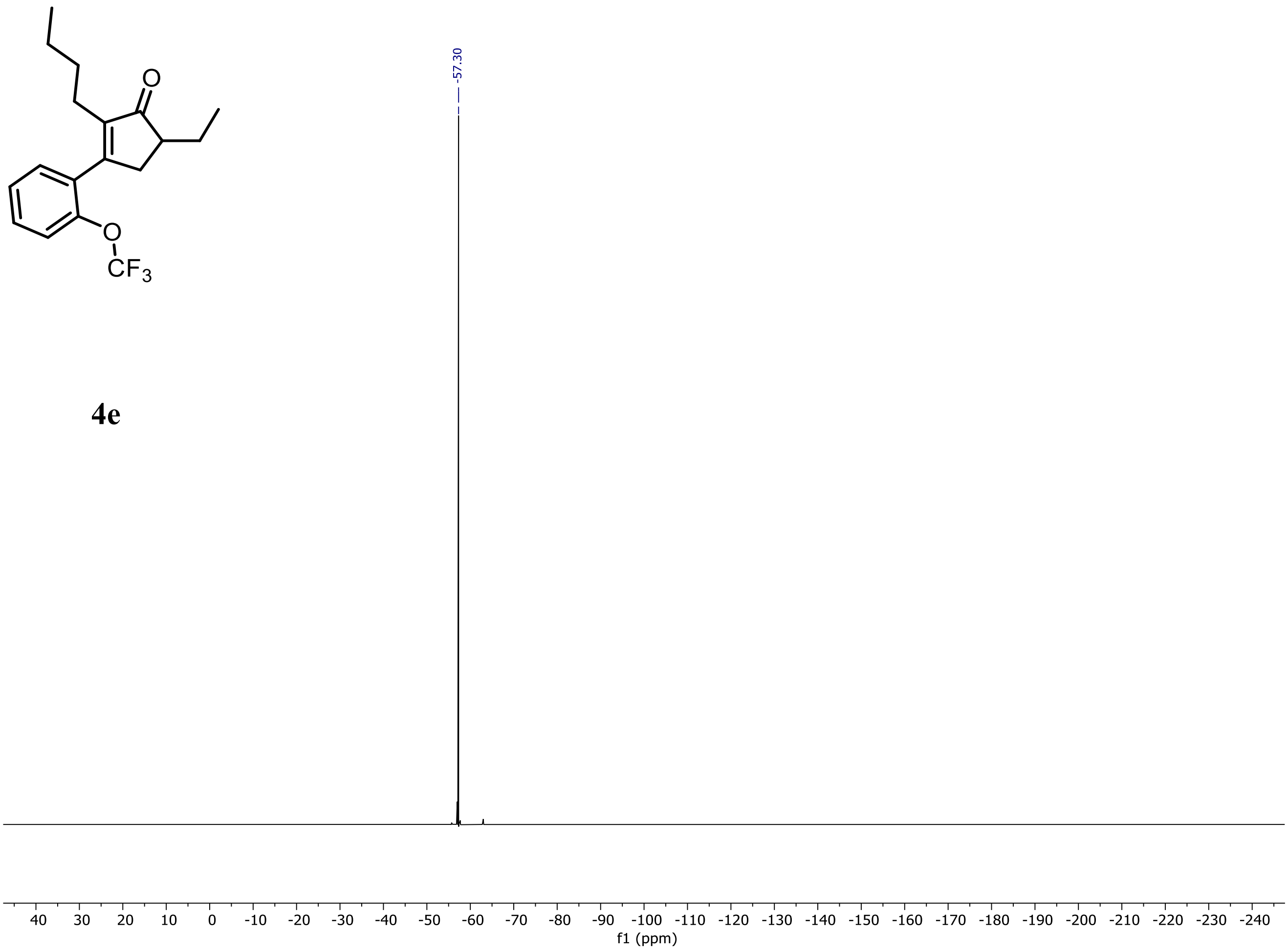
O
O
CF_3
-57.30
40 30 20 10 0 -10 -20 -30 -40 -50 -60 -70 -80 -90 -100 -110 -120 -130 -140 -150 -160 -170 -180 -190 -200 -210 -220 -230 -240
f1 (ppm)

4e

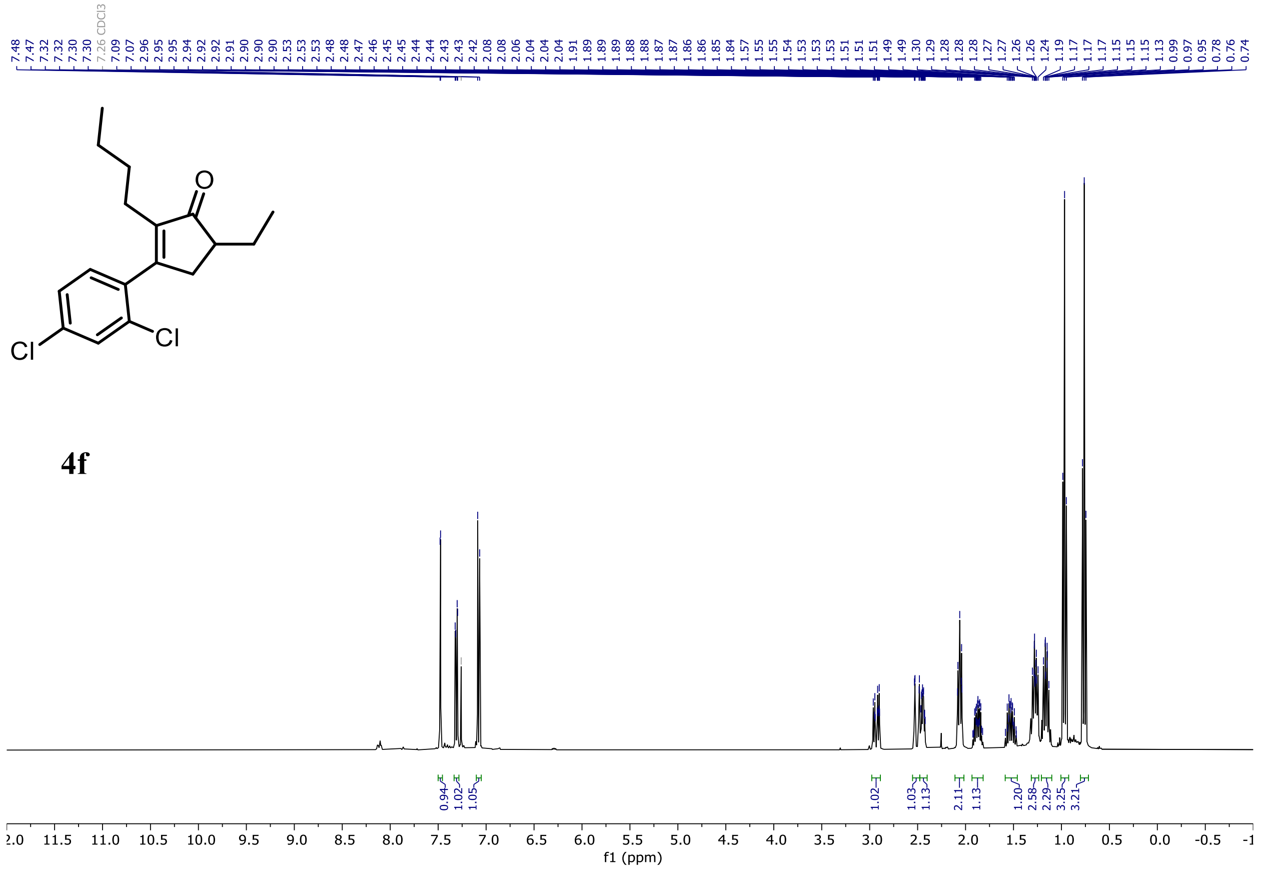

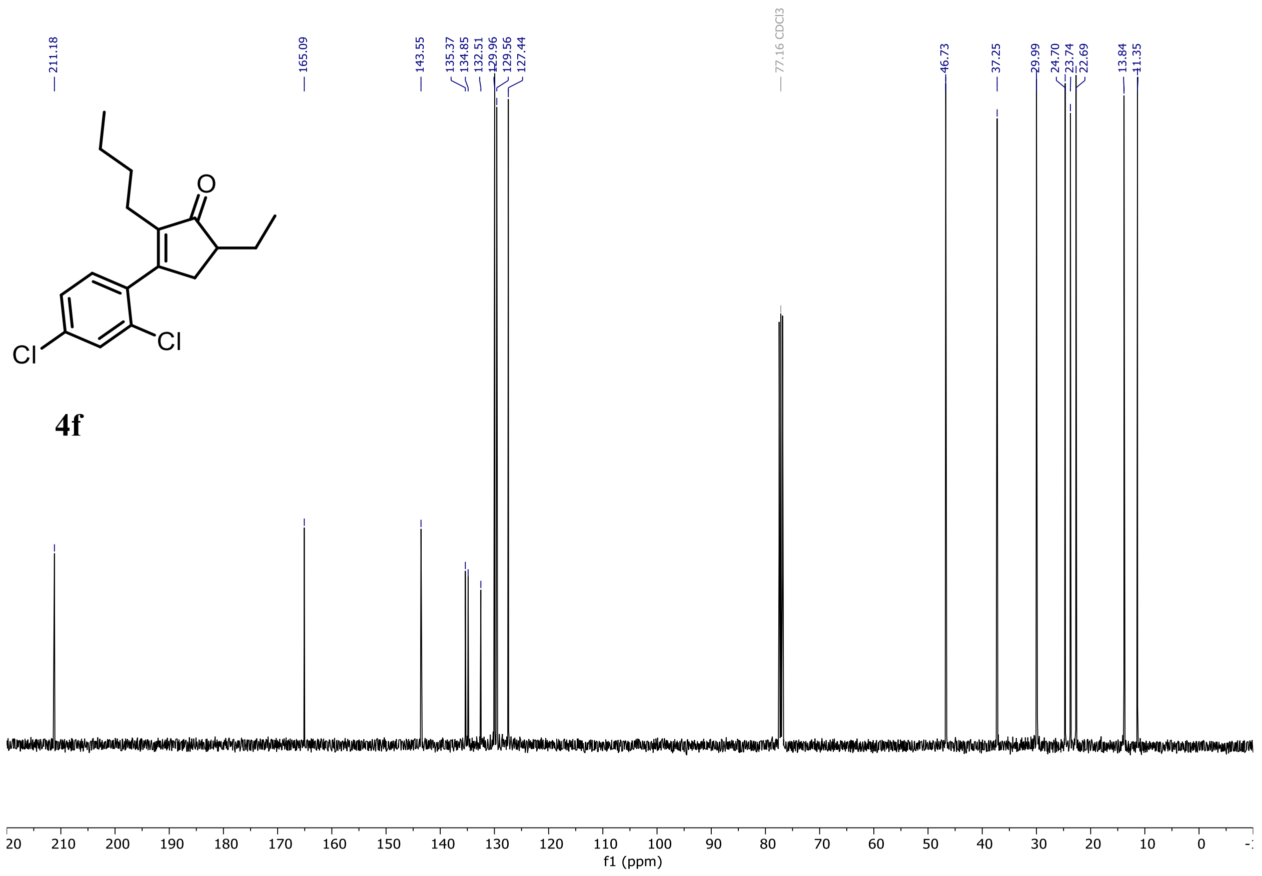

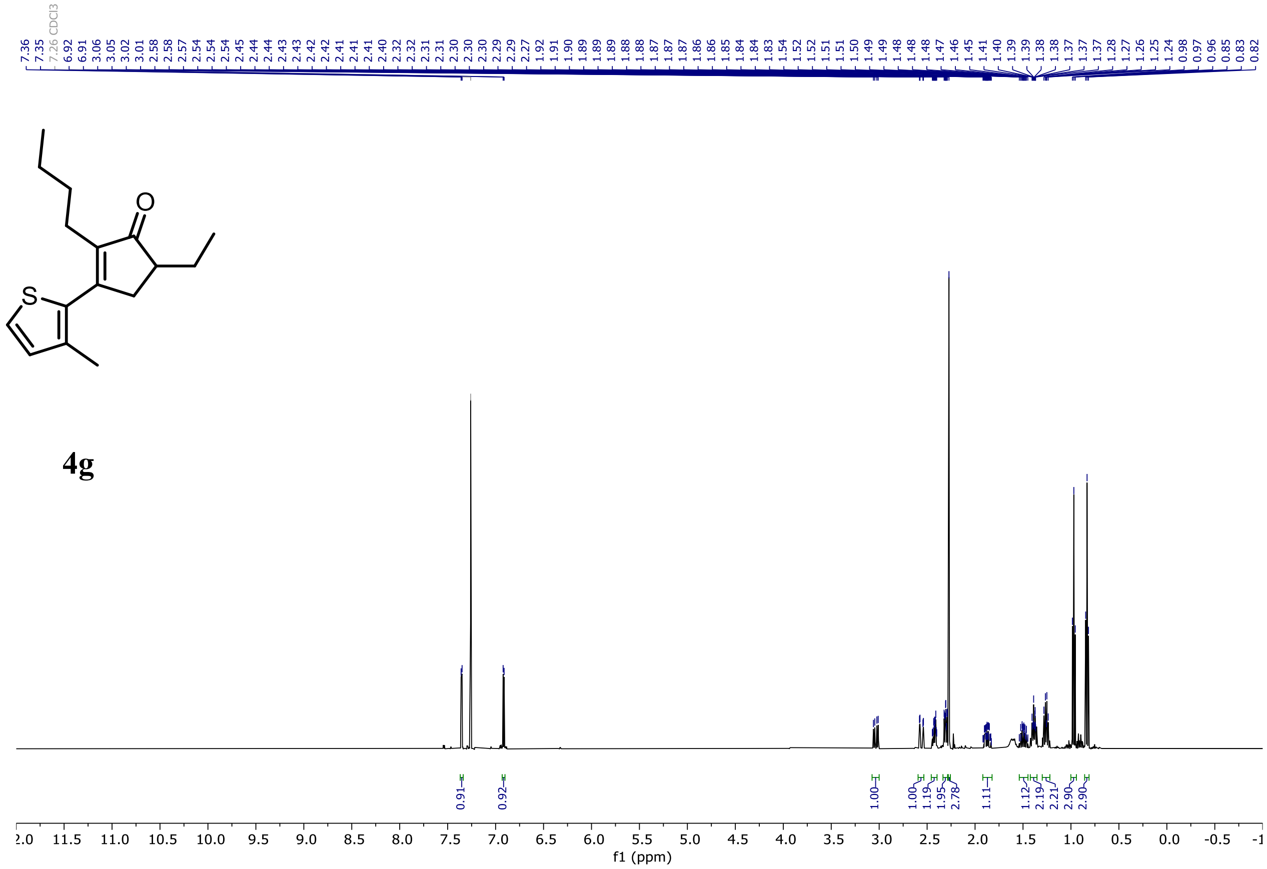
7.36
7.35
7.26 CDCl3
6.92
6.91
3.06
3.05
3.02
3.01
2.58
2.58
2.57
2.54
2.54
2.54
2.45
2.44
2.44
2.43
2.43
2.42
2.42
2.41
2.41
2.40
2.32
2.31
2.30
2.30
2.30
2.29
2.29
2.27
1.92
1.91
1.90
1.89
1.89
1.88
1.87
1.87
1.87
1.86
1.85
1.84
1.84
1.83
1.54
1.52
1.52
1.51
1.51
1.50
1.49
1.49
1.48
1.48
1.47
1.46
1.45
1.41
1.40
1.39
1.39
1.38
1.38
1.37
1.37
1.37
1.28
1.27
1.26
1.25
1.24
0.98
0.97
0.96
0.85
0.83
0.82
S
O
0.91
0.92
1.00
1.00
1.19
1.95
2.78
1.11
1.12
2.19
2.21
2.90
2.90
2.0 11.5 11.0 10.5 10.0 9.5 9.0 8.5 8.0 7.5 7.0 6.5 6.0 5.5 5.0 4.5 4.0 3.5 3.0 2.5 2.0 1.5 1.0 0.5 0.0 -0.5
f1 (ppm)

4g

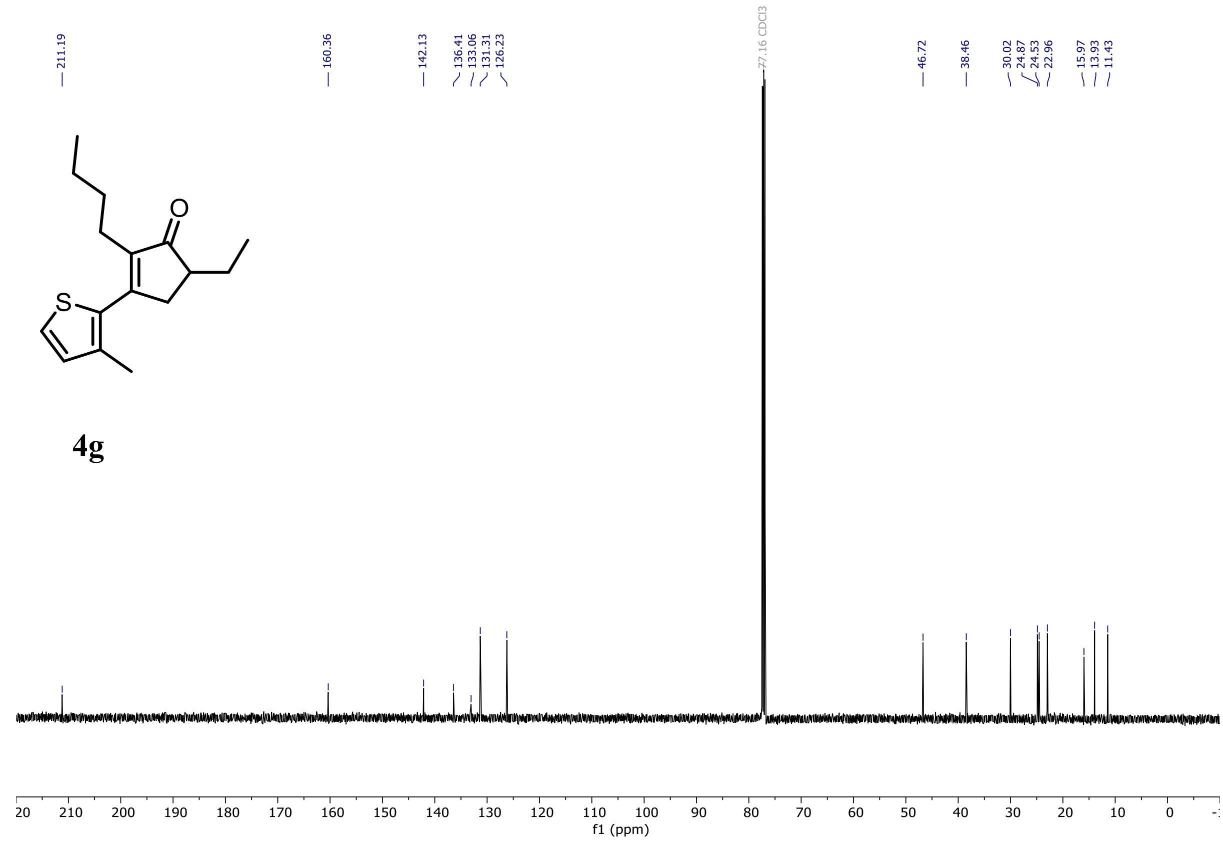
211.19
160.36
142.13
136.41
133.06
131.31
126.23
77.16 CDCl3
46.72
38.46
30.02
24.87
24.53
22.96
15.97
13.93
11.43
S
O
20 210 200 190 180 170 160 150 140 130 120 110 100 90 80 70 60 50 40 30 20 10 0
f1 (ppm)

4g

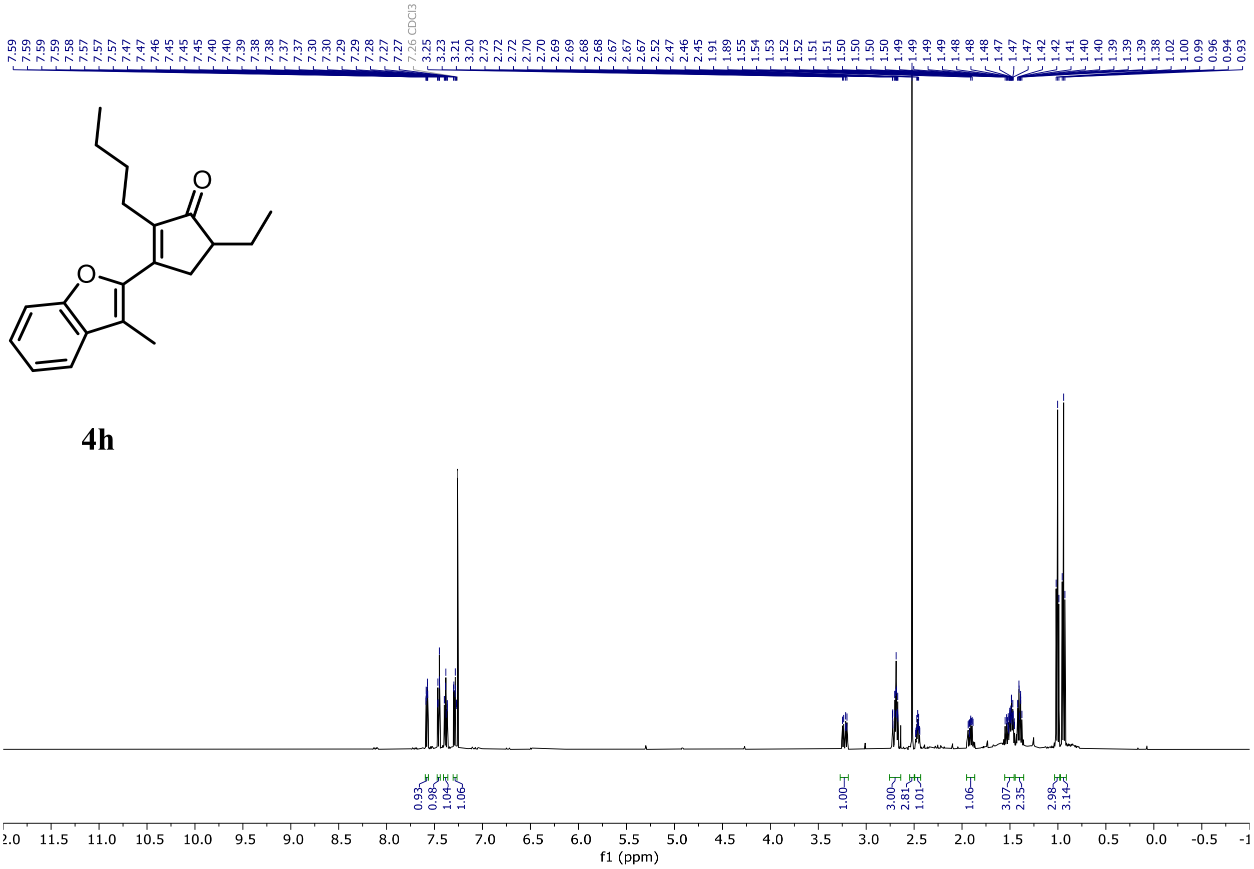
4h
7.59 7.59 7.59 7.58 7.57 7.57 7.57 7.47 7.47 7.46 7.45 7.45 7.45 7.40 7.40 7.39 7.38 7.38 7.37 7.37 7.30 7.30 7.29 7.29 7.28 7.27 7.27 7.26 CDCl3 3.25 3.23 3.21 3.20 2.73 2.72 2.72 2.70 2.70 2.69 2.69 2.68 2.67 2.67 2.67 2.52 2.47 2.46 2.45 1.91 1.89 1.55 1.54 1.53 1.52 1.52 1.51 1.51 1.50 1.50 1.50 1.50 1.49 1.49 1.49 1.49 1.48 1.48 1.48 1.47 1.47 1.47 1.42 1.42 1.41 1.40 1.40 1.39 1.39 1.39 1.38 1.02 1.00 0.99 0.96 0.94 0.93
0.93 0.98 1.04 1.06
1.00
3.00 2.81 1.01
1.06
3.07 2.35
2.98 3.14
f1 (ppm)

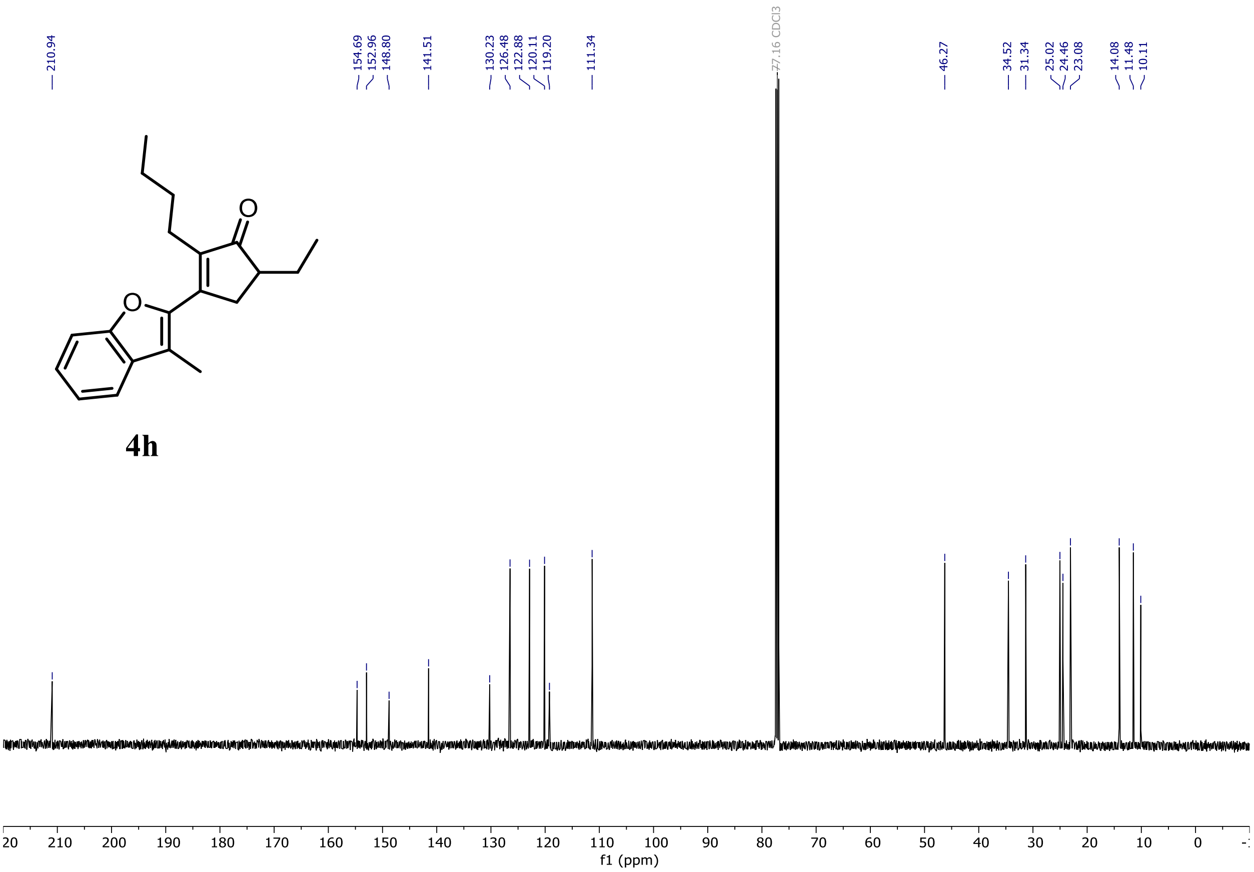
4h
210.94
154.69 152.96 148.80
141.51
130.23 126.48 122.88 120.11 119.20
111.34
77.16 CDCl3
46.27
34.52 31.34
25.02 24.46 23.08
14.08 11.48 10.11
f1 (ppm)

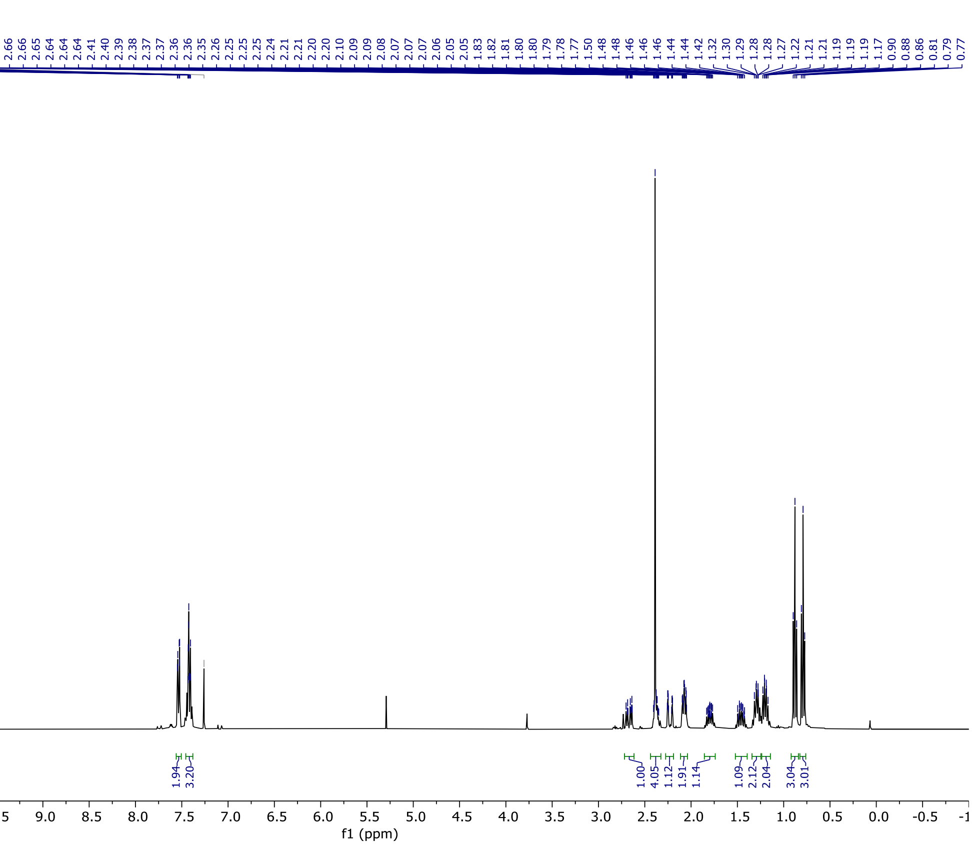
7.55 7.54 7.53 7.52 7.52 7.43 7.43 7.43 7.42 7.42 7.41 7.26 CDCl3 2.70 2.69 2.69 2.68 2.66 2.65 2.64 2.64 2.64 2.41 2.40 2.39 2.38 2.37 2.37 2.36 2.36 2.35 2.26 2.25 2.25 2.24 2.21 2.21 2.20 2.20 2.10 2.09 2.09 2.08 2.07 2.07 2.07 2.06 2.05 2.05 1.83 1.82 1.82 1.81 1.80 1.80 1.79 1.78 1.77 1.50 1.48 1.46 1.46 1.46 1.44 1.44 1.42 1.32 1.30 1.29 1.28 1.28 1.27 1.22 1.21 1.21 1.19 1.19 1.19 1.17 0.90 0.88 0.86 0.81 0.79 0.77
1.94 3.20 1.00 4.05 1.12 1.91 1.14 1.09 2.12 2.04 3.04 3.01
f1 (ppm)
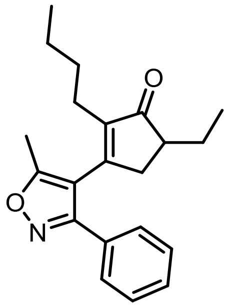

4i

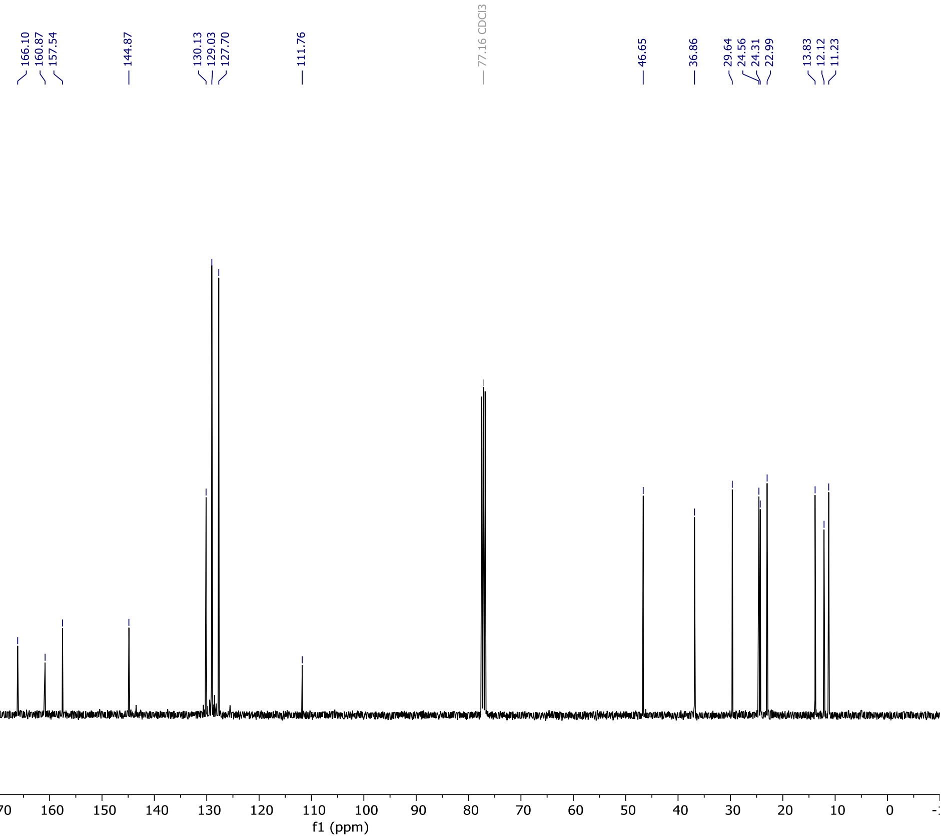
211.04 166.10 160.87 157.54 144.87 130.13 129.03 127.70 111.76 77.16 CDCl3 46.65 36.86 29.64 24.56 24.31 22.99 13.83 12.12 11.23
f1 (ppm)
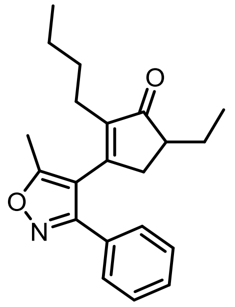

4i

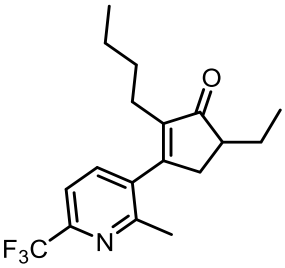
O
N
F3C

4j

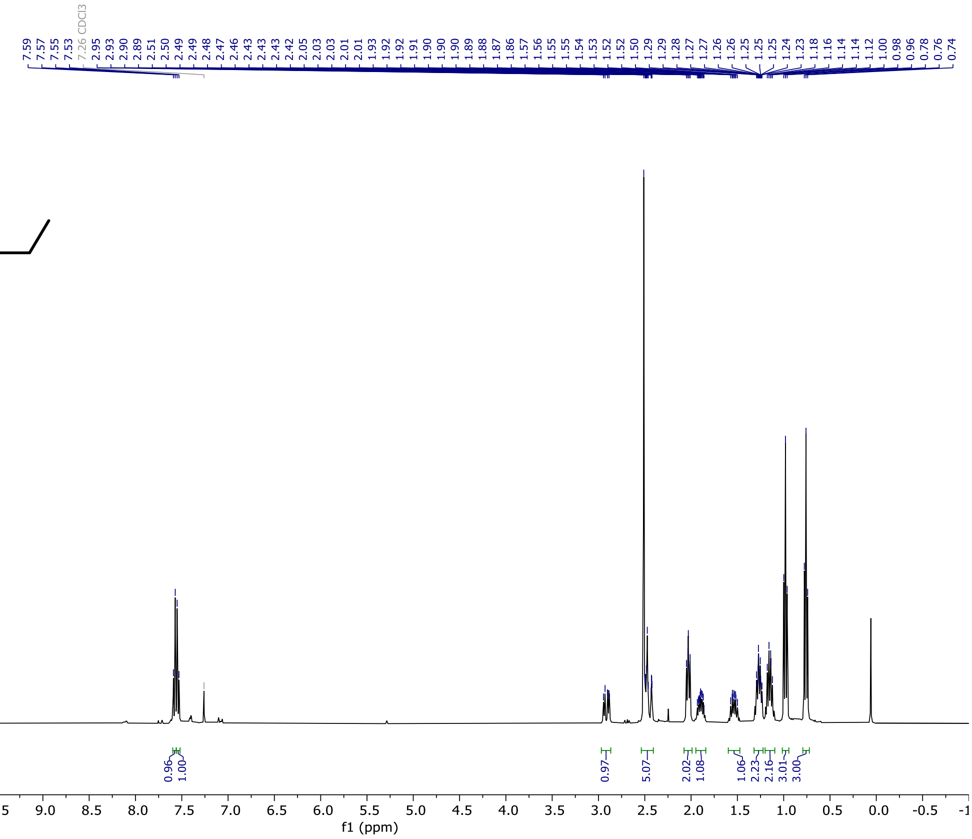
7.59 7.57 7.55 7.53 7.26 CDCl3 2.95 2.93 2.90 2.89 2.51 2.50 2.49 2.49 2.48 2.47 2.46 2.43 2.43 2.42 2.05 2.03 2.03 2.01 2.01 1.93 1.92 1.92 1.91 1.90 1.90 1.90 1.89 1.88 1.87 1.86 1.57 1.56 1.55 1.55 1.54 1.53 1.52 1.52 1.50 1.29 1.29 1.28 1.27 1.27 1.26 1.26 1.25 1.25 1.25 1.24 1.23 1.18 1.16 1.14 1.14 1.12 1.00 0.98 0.96 0.78 0.76 0.74
0.96 1.00 0.97 5.07 2.02 1.08 1.06 2.23 2.16 3.01 3.00
f1 (ppm)

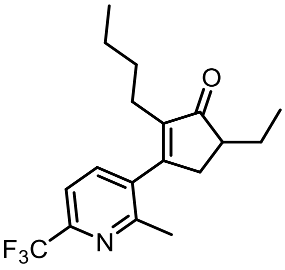
O
N
F3C

4j

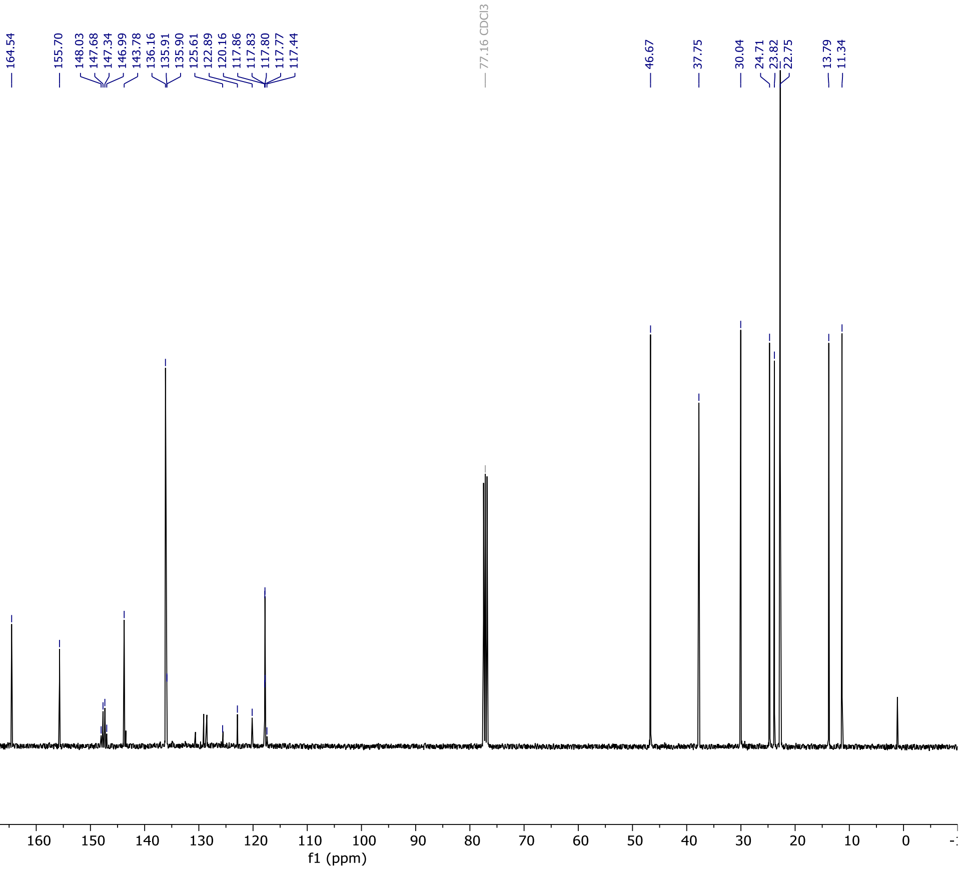
210.48 164.54 155.70 148.03 147.68 147.34 146.99 143.78 136.16 135.91 135.90 125.61 122.89 120.16 117.86 117.83 117.80 117.77 117.44 77.16 CDCl3 46.67 37.75 30.04 24.71 23.82 22.75 13.79 11.34
f1 (ppm)

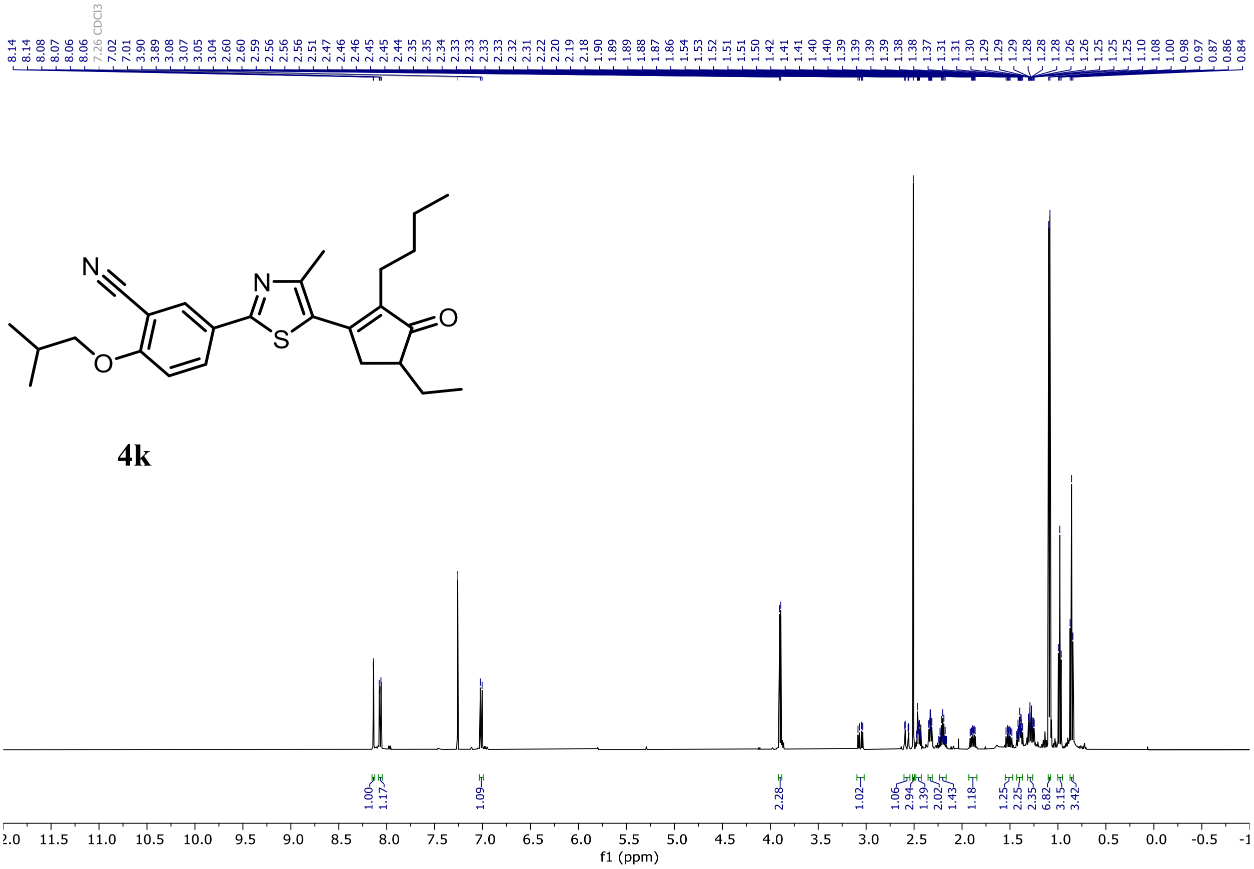
4k
f1 (ppm)

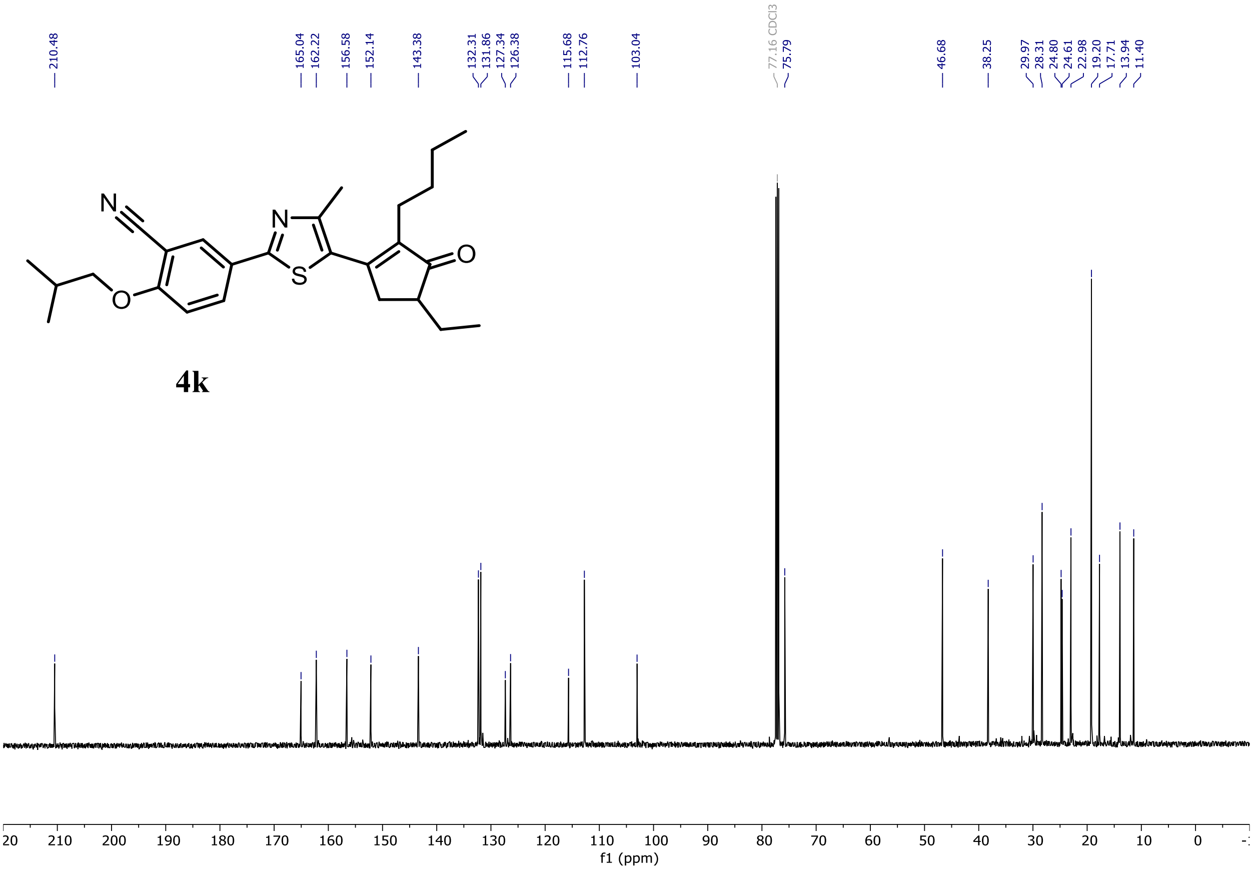
4k
f1 (ppm)

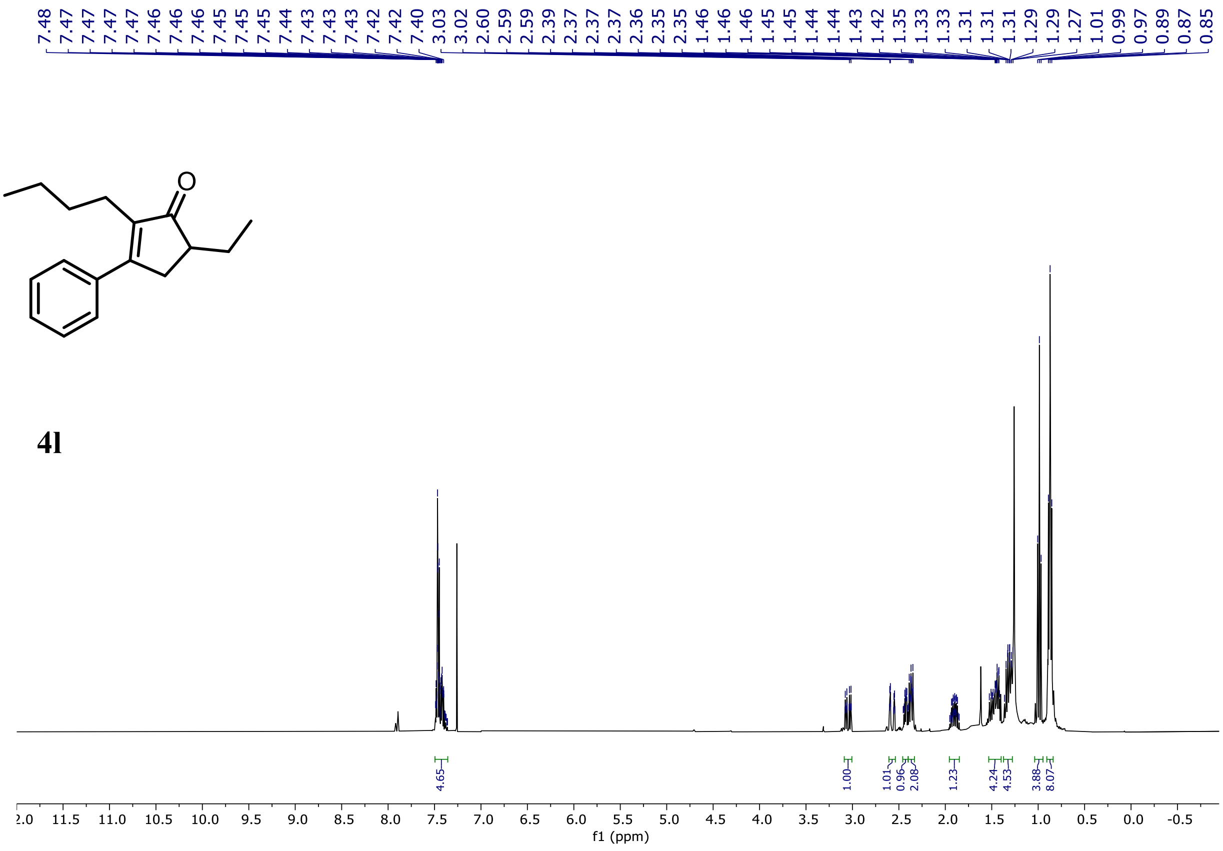
7.48 7.47 7.47 7.47 7.47 7.46 7.46 7.46 7.45 7.45 7.45 7.44 7.43 7.43 7.43 7.42 7.42 7.40 3.03 3.02 2.60 2.59 2.59 2.39 2.37 2.37 2.37 2.36 2.35 2.35 1.46 1.46 1.46 1.45 1.45 1.44 1.43 1.42 1.35 1.33 1.33 1.31 1.31 1.31 1.29 1.29 1.27 1.01 0.99 0.97 0.89 0.87 0.85
O
4l
4.65
1.00
1.01
0.96
2.08
1.23
4.24
4.53
3.88
8.07
f1 (ppm)

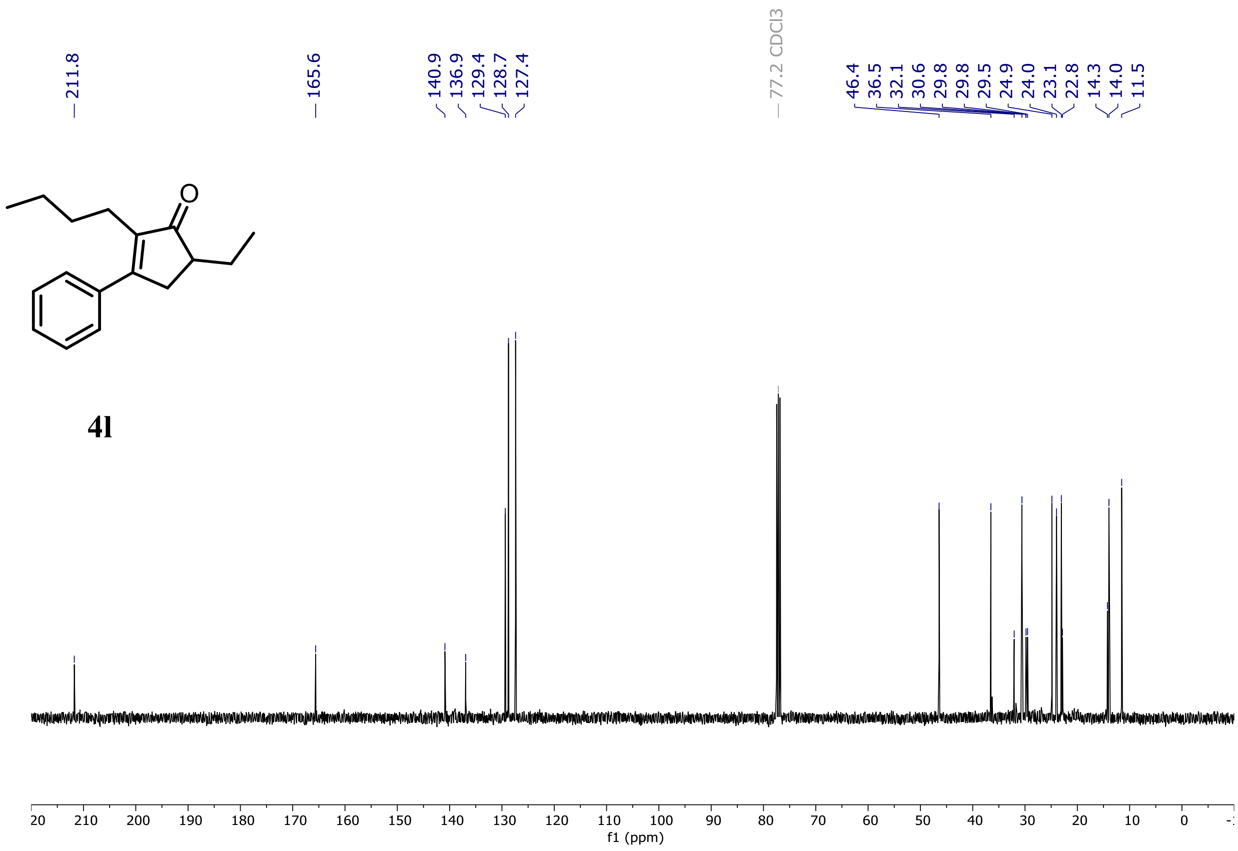
211.8
165.6
140.9 136.9 129.4 128.7 127.4
77.2 CDCl3
46.4 36.5 32.1 30.6 29.8 29.8 29.5 24.9 24.0 23.1 22.8 14.3 14.0 11.5
O
4l
f1 (ppm)

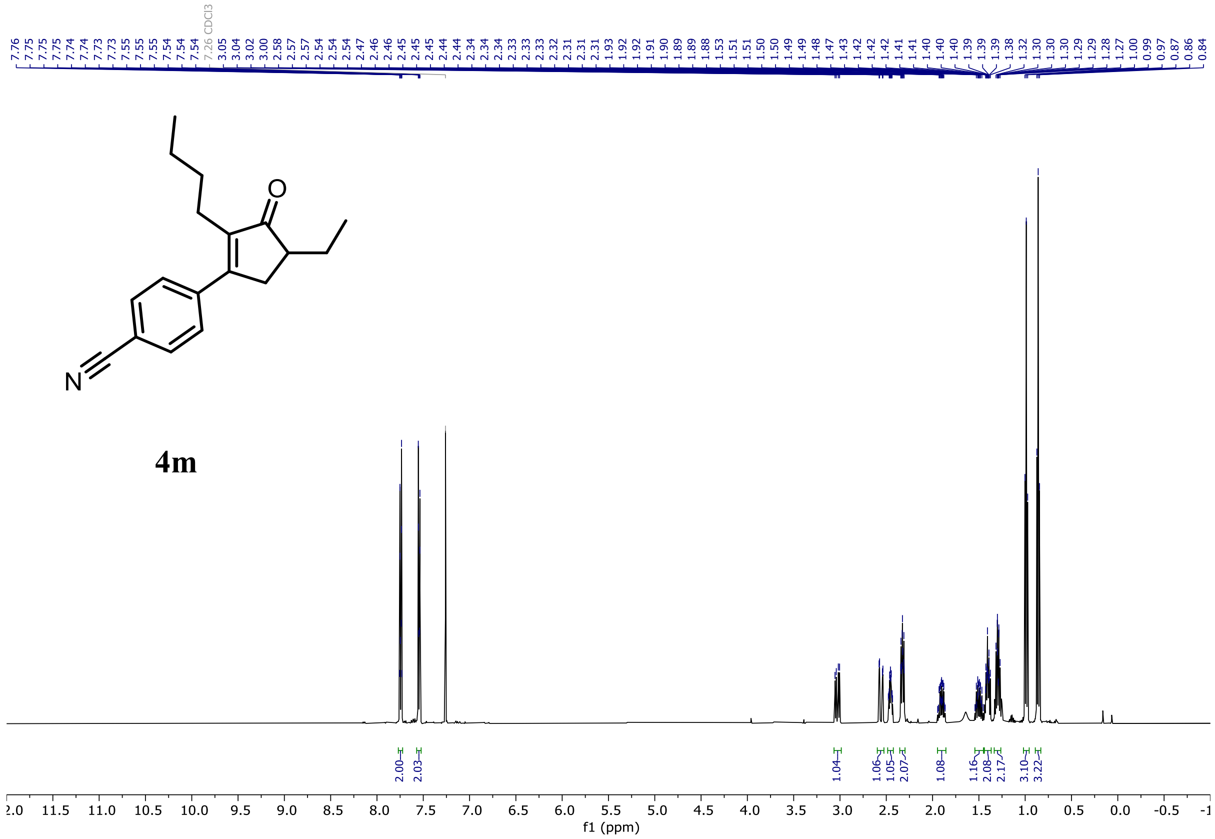
4m
f1 (ppm)

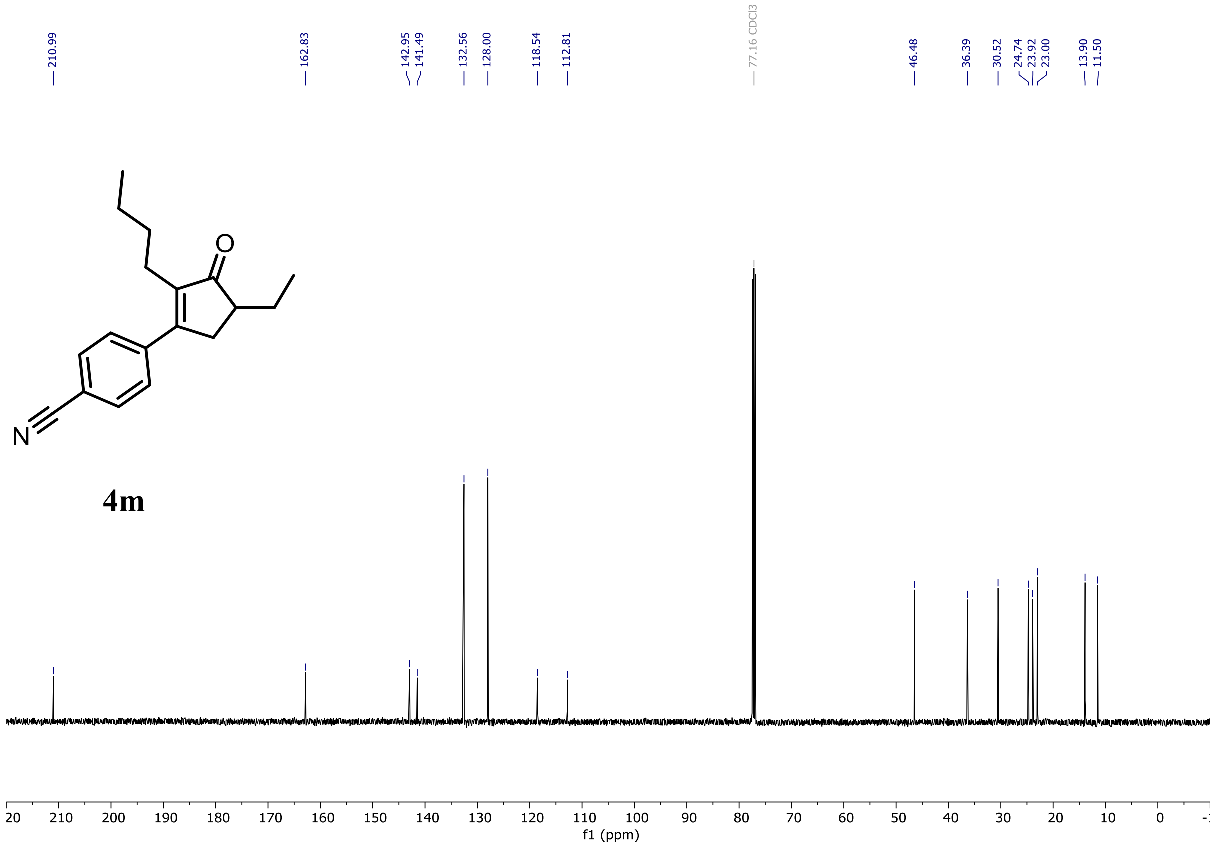
210.99
162.83
142.95
141.49
132.56
128.00
118.54
112.81
77.16 CDCl3
46.48
36.39
30.52
24.74
23.92
23.00
13.90
11.50
4m
f1 (ppm)

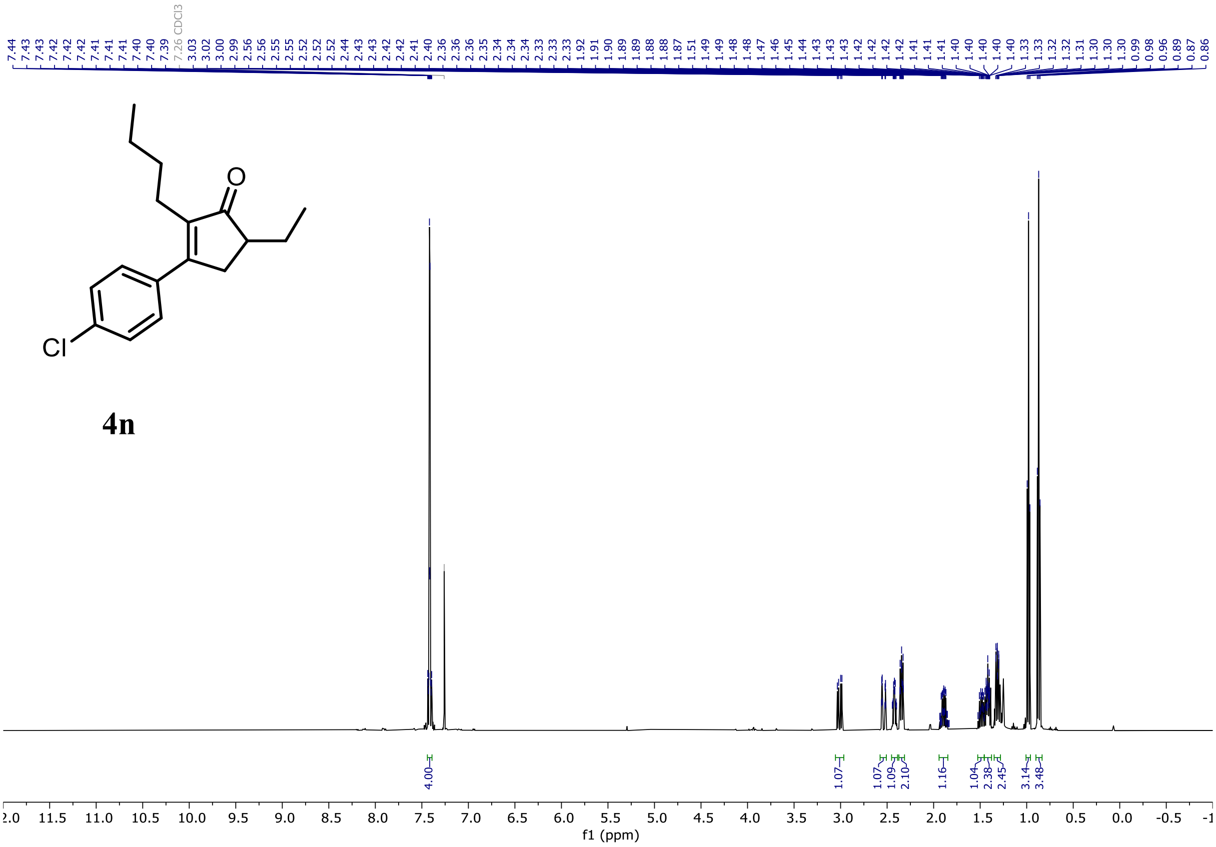
7.44 7.43 7.43 7.42 7.42 7.41 7.41 7.41 7.40 7.39 7.26 CDCl3 3.03 3.02 3.00 2.99 2.56 2.56 2.55 2.55 2.52 2.52 2.52 2.44 2.43 2.43 2.42 2.42 2.41 2.40 2.36 2.36 2.36 2.35 2.34 2.34 2.34 2.33 2.33 2.33 1.92 1.91 1.90 1.89 1.89 1.88 1.88 1.87 1.51 1.49 1.49 1.48 1.48 1.47 1.46 1.45 1.44 1.43 1.43 1.43 1.42 1.42 1.42 1.41 1.41 1.41 1.40 1.40 1.40 1.40 1.40 1.33 1.33 1.32 1.32 1.31 1.30 1.30 0.99 0.98 0.96 0.89 0.87 0.86
O
Cl
4n
4.00
1.07
1.07
1.09
2.10
1.16
1.04
2.38
2.45
3.14
3.48
f1 (ppm)

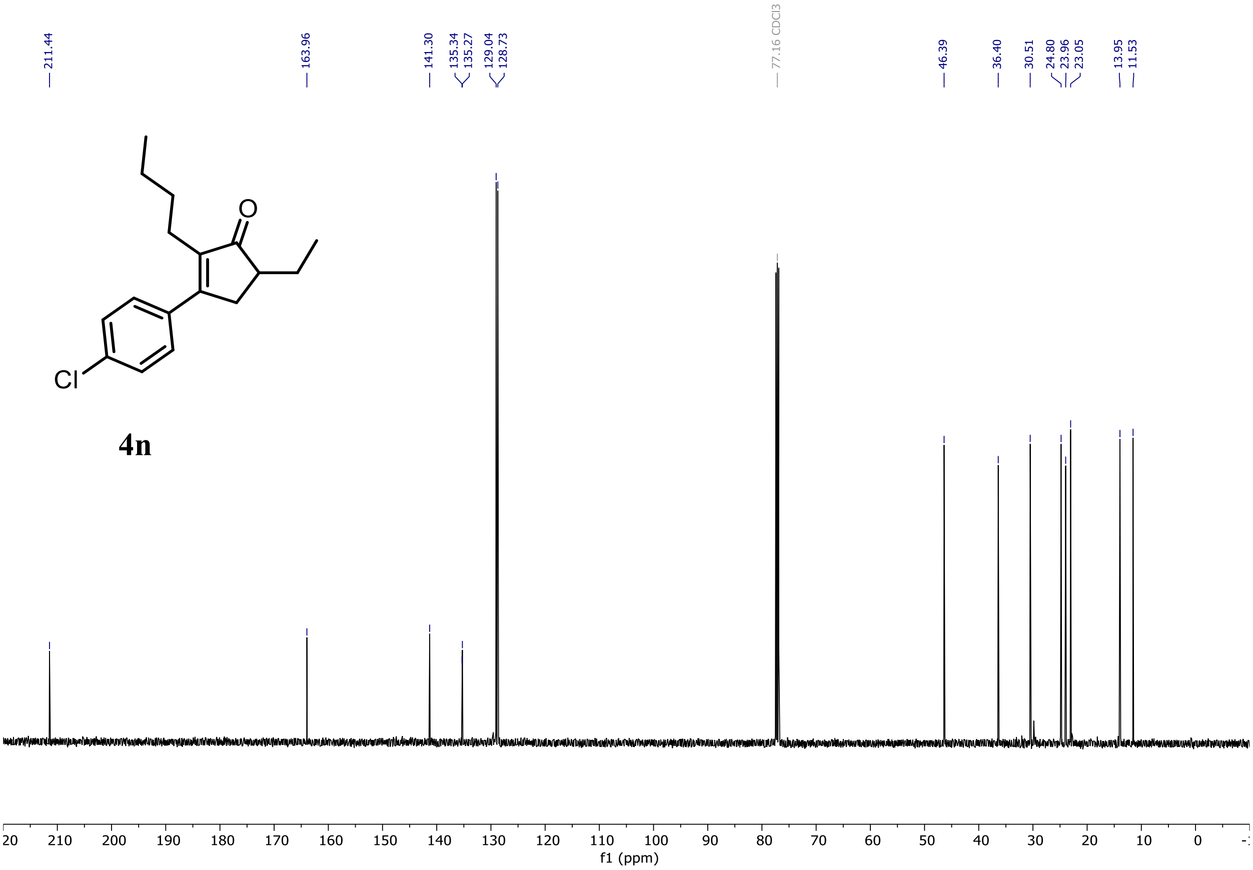
211.44
163.96
141.30
135.34
135.27
129.04
128.73
77.16 CDCl3
46.39
36.40
30.51
24.80
23.96
23.05
13.95
11.53
O
Cl
4n
f1 (ppm)

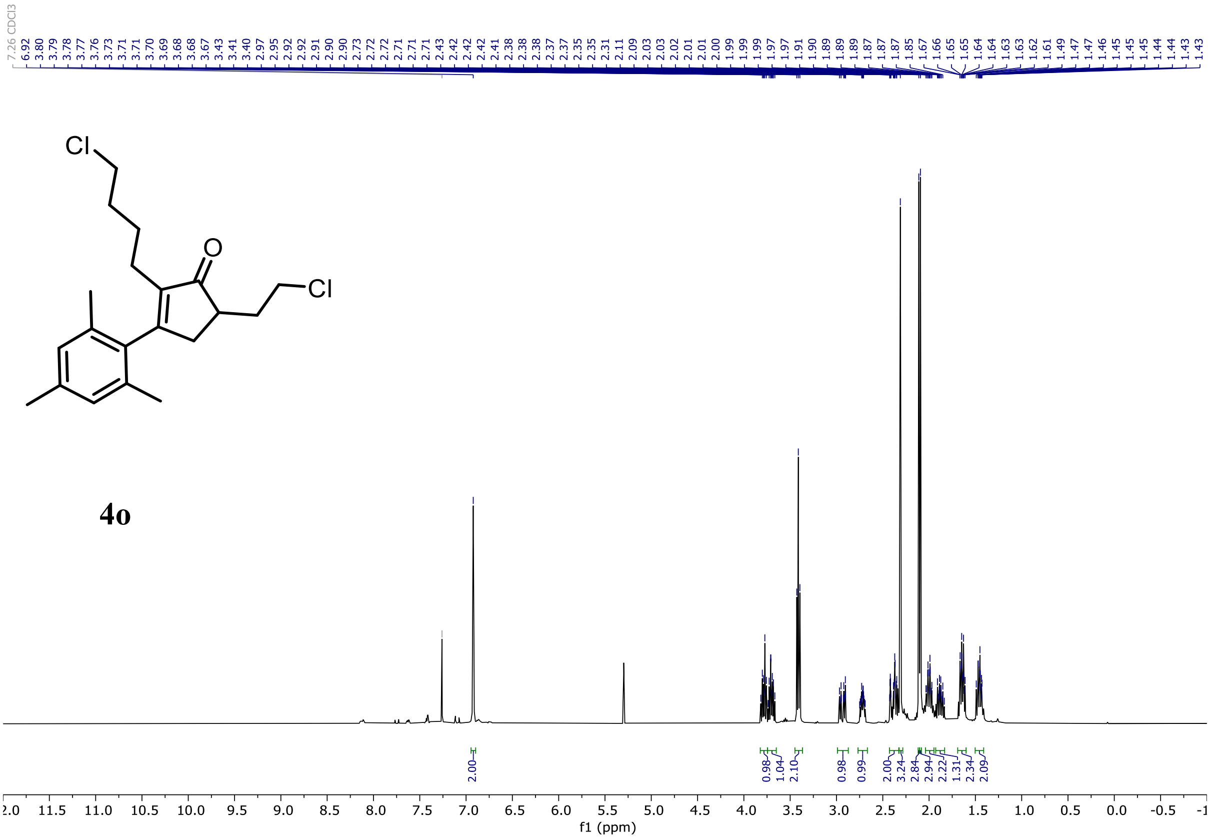
7.26 CDCl3
6.92 3.80 3.79 3.78 3.77 3.76 3.73 3.71 3.71 3.70 3.69 3.68 3.68 3.67 3.43 3.41 3.40 2.97 2.95 2.92 2.92 2.91 2.90 2.90 2.73 2.72 2.72 2.71 2.71 2.71 2.43 2.42 2.42 2.42 2.41 2.38 2.38 2.38 2.37 2.35 2.35 2.31 2.11 2.09 2.03 2.03 2.02 2.01 2.01 2.00 1.99 1.99 1.99 1.97 1.97 1.91 1.90 1.89 1.89 1.89 1.87 1.87 1.87 1.85 1.67 1.66 1.65 1.65 1.64 1.64 1.63 1.63 1.62 1.61 1.49 1.47 1.47 1.46 1.45 1.45 1.44 1.44 1.43 1.43
Cl
O
Cl
4o
2.00 0.98 1.04 2.10 0.98 0.99 2.00 3.24 2.84 2.94 2.22 1.31 2.34 2.09
f1 (ppm)

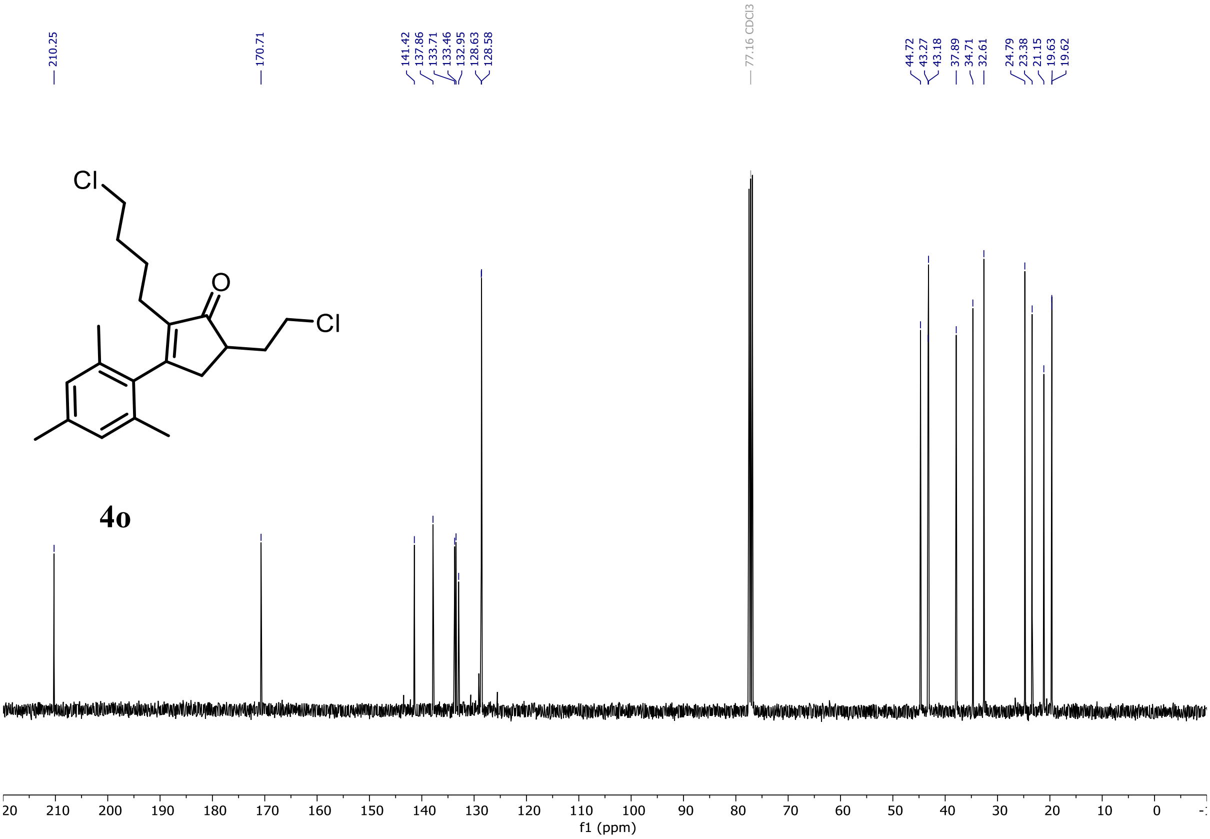
210.25
170.71
141.42 137.86 133.71 133.46 132.95 128.63 128.58
77.16 CDCl3
44.72 43.27 43.18 37.89 34.71 32.61 24.79 23.38 21.15 19.63 19.62
Cl
O
Cl
4o
f1 (ppm)

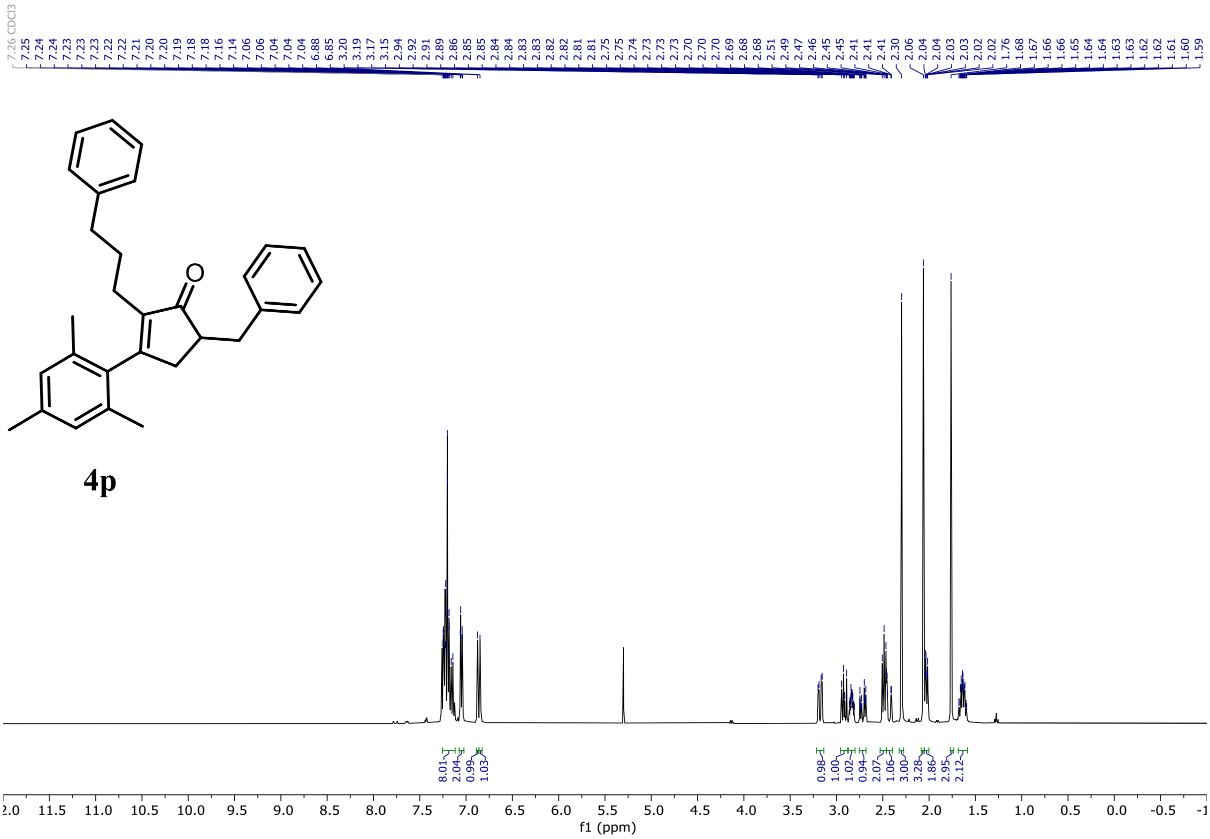
7.26 CDCl3, 7.25, 7.24, 7.24, 7.23, 7.23, 7.23, 7.22, 7.22, 7.21, 7.20, 7.20, 7.19, 7.18, 7.18, 7.16, 7.14, 7.06, 7.06, 7.04, 7.04, 7.04, 6.88, 6.85, 3.20, 3.19, 3.17, 3.15, 2.94, 2.92, 2.91, 2.89, 2.86, 2.85, 2.85, 2.84, 2.84, 2.83, 2.83, 2.82, 2.82, 2.81, 2.81, 2.75, 2.74, 2.73, 2.73, 2.73, 2.70, 2.70, 2.69, 2.68, 2.51, 2.49, 2.47, 2.46, 2.45, 2.41, 2.41, 2.41, 2.30, 2.06, 2.04, 2.04, 2.03, 2.03, 2.02, 2.02, 1.76, 1.68, 1.67, 1.66, 1.66, 1.65, 1.64, 1.64, 1.63, 1.63, 1.62, 1.62, 1.61, 1.60, 1.59
O
4p
8.01, 2.04, 0.99, 1.03, 0.98, 1.00, 1.02, 0.94, 2.07, 1.06, 3.00, 3.28, 1.86, 2.95, 2.12
f1 (ppm)

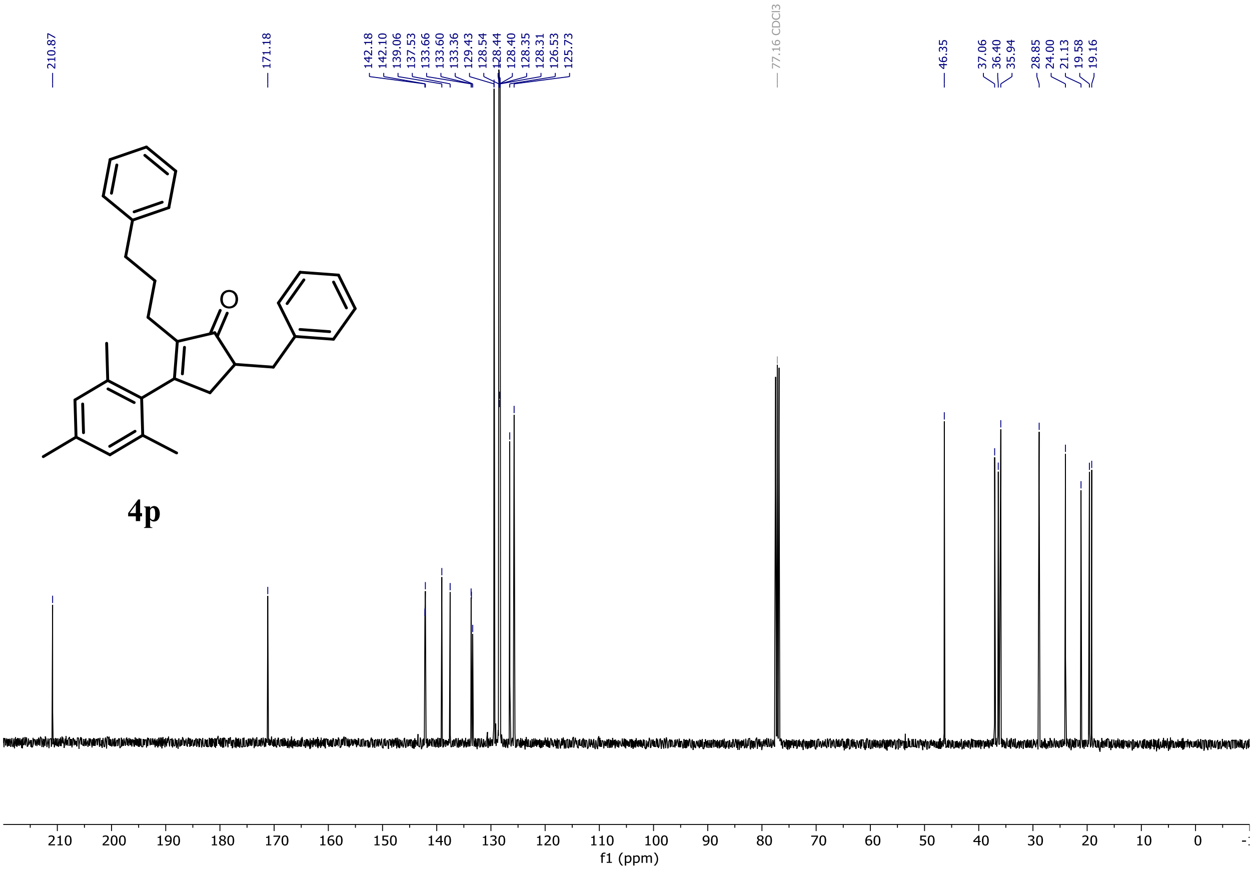
210.87, 171.18, 142.18, 142.10, 139.06, 137.53, 133.66, 133.60, 133.36, 129.43, 128.54, 128.44, 128.40, 128.35, 128.31, 126.53, 125.73, 77.16 CDCl3, 46.35, 37.06, 36.40, 35.94, 28.85, 24.00, 21.13, 19.58, 19.16
O
4p
f1 (ppm)

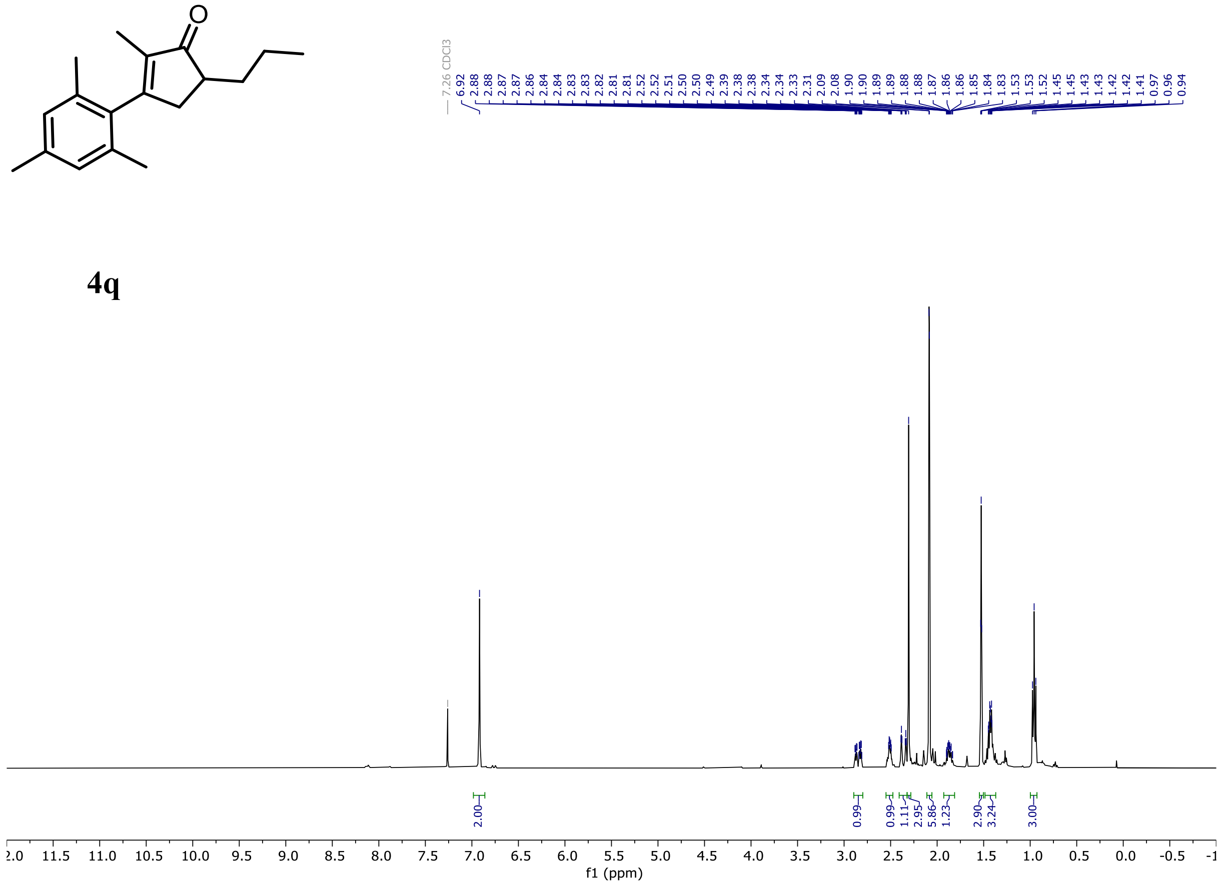

7.26 CDCl3
6.92
2.88
2.88
2.87
2.87
2.86
2.84
2.84
2.83
2.83
2.82
2.81
2.81
2.52
2.52
2.51
2.50
2.50
2.49
2.39
2.38
2.38
2.34
2.34
2.33
2.31
2.09
2.08
1.90
1.90
1.89
1.89
1.88
1.88
1.87
1.86
1.86
1.85
1.84
1.83
1.53
1.53
1.52
1.45
1.45
1.43
1.43
1.42
1.41
0.97
0.96
0.94
2.00
0.99
0.99
1.11
2.95
5.86
1.23
2.90
3.24
3.00
f1 (ppm)

4q

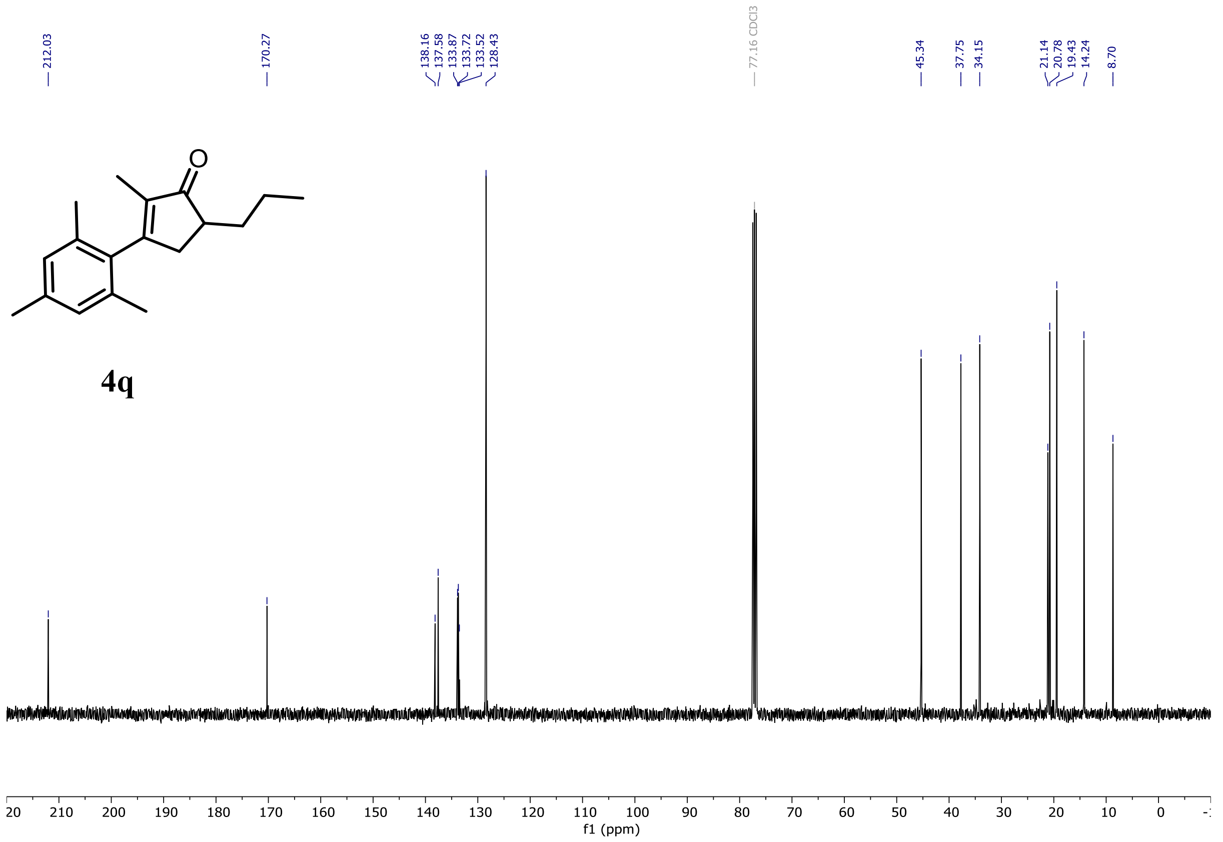

212.03
170.27
138.16
137.58
133.87
133.72
133.52
128.43
77.16 CDCl3
45.34
37.75
34.15
21.14
20.78
19.43
14.24
8.70
f1 (ppm)

4q

Mechanistic investigations

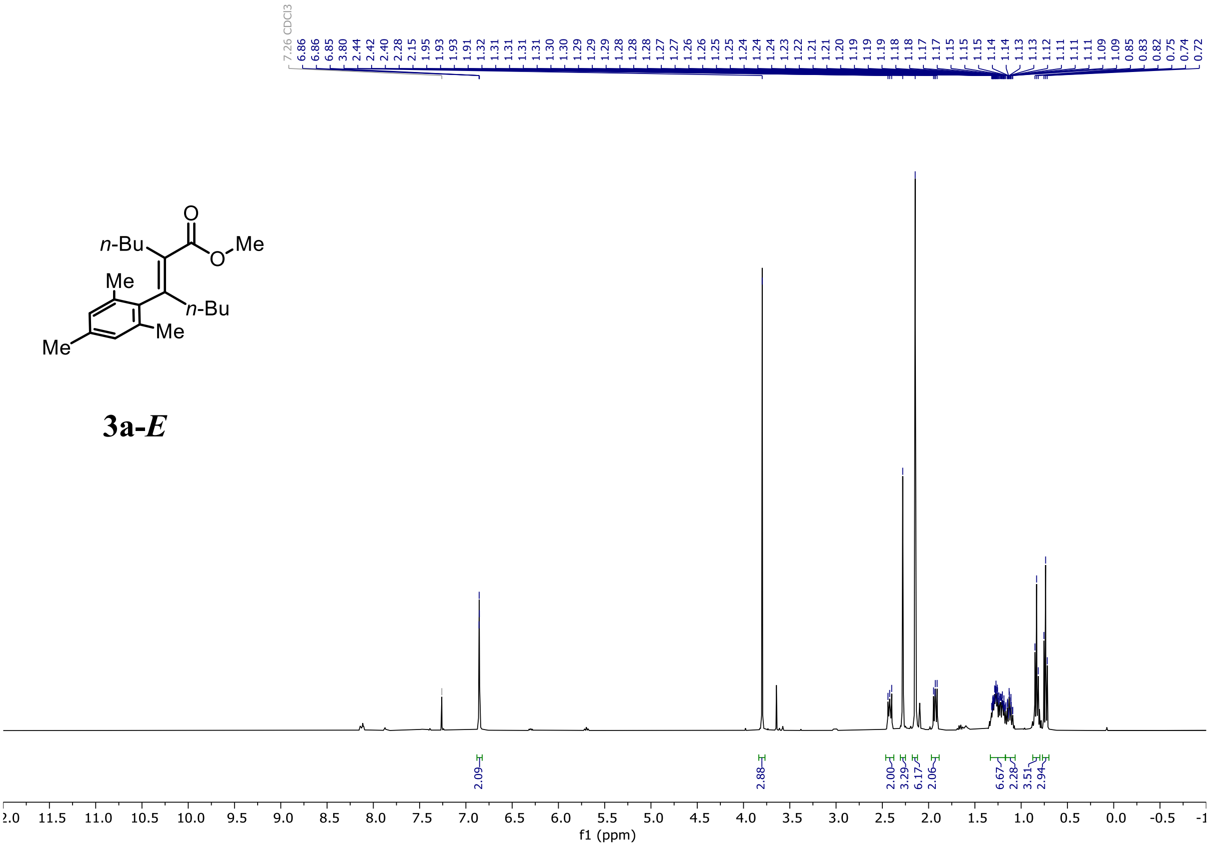
7.26 CDCl3
6.86
6.86
6.85
3.80
2.44
2.42
2.40
2.28
2.15
1.95
1.93
1.93
1.91
1.32
1.31
1.31
1.31
1.30
1.29
1.29
1.29
1.28
1.28
1.28
1.27
1.27
1.26
1.25
1.25
1.24
1.24
1.24
1.23
1.22
1.21
1.21
1.20
1.19
1.19
1.19
1.18
1.18
1.17
1.17
1.15
1.15
1.15
1.14
1.14
1.14
1.13
1.13
1.12
1.11
1.11
1.11
1.09
0.85
0.83
0.82
0.75
0.74
0.72
n-Bu
O
O
Me
Me
n-Bu
Me
Me
3a-E
2.09
2.88
2.00
3.29
6.17
2.06
6.67
2.28
3.51
2.94
f1 (ppm)

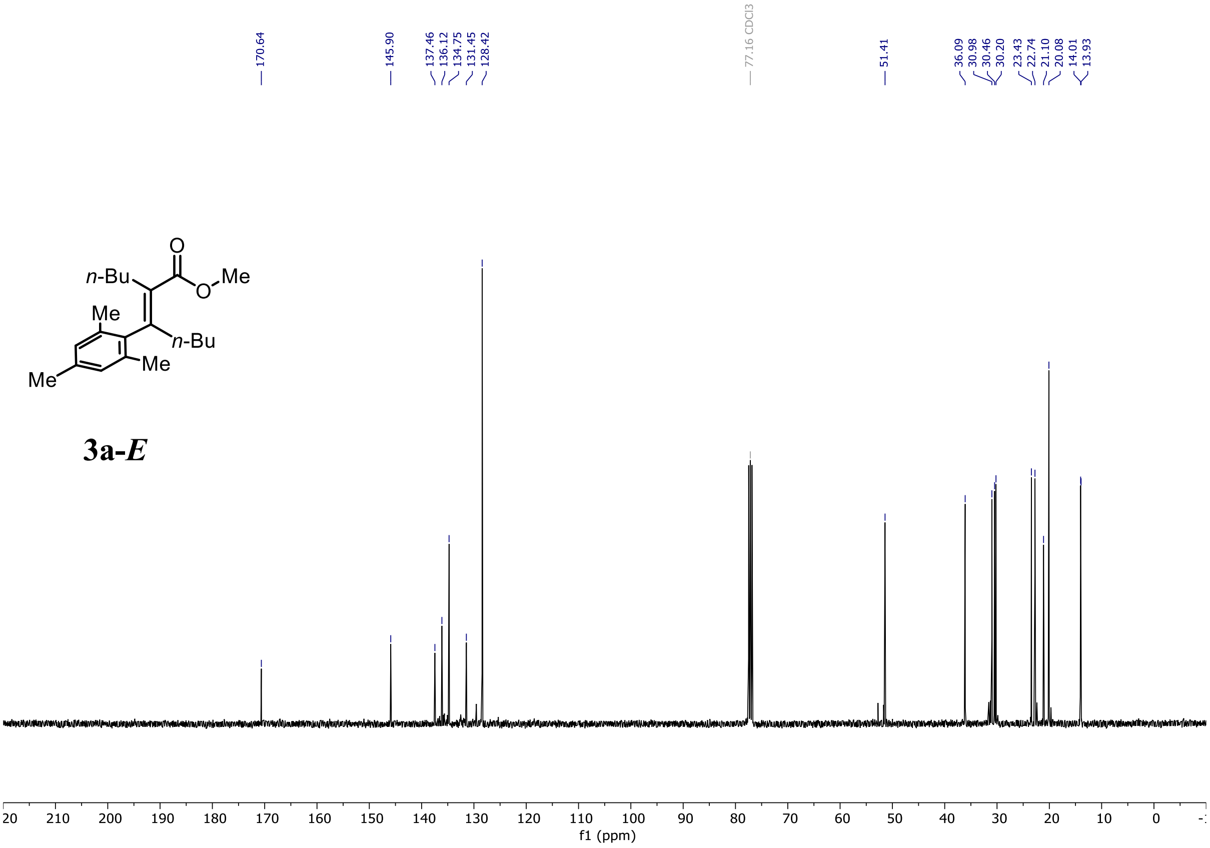
170.64
145.90
137.46
136.12
134.75
131.45
128.42
77.16 CDCl3
51.41
36.09
30.98
30.46
30.20
23.43
22.74
21.10
20.08
14.01
13.93
n-Bu
O
O
Me
Me
n-Bu
Me
Me
3a-E
f1 (ppm)

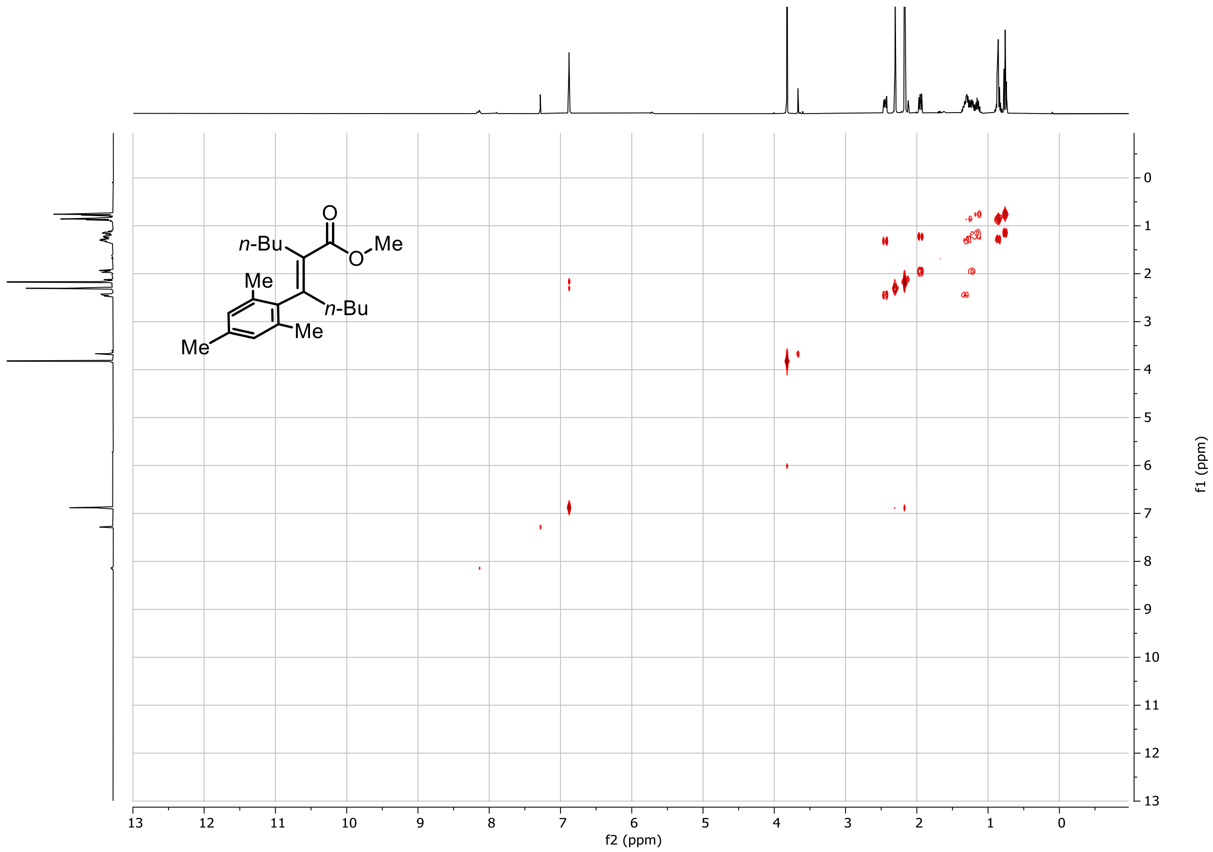
n-Bu
O
O
Me
Me
n-Bu
Me
Me
f2 (ppm)
f1 (ppm)

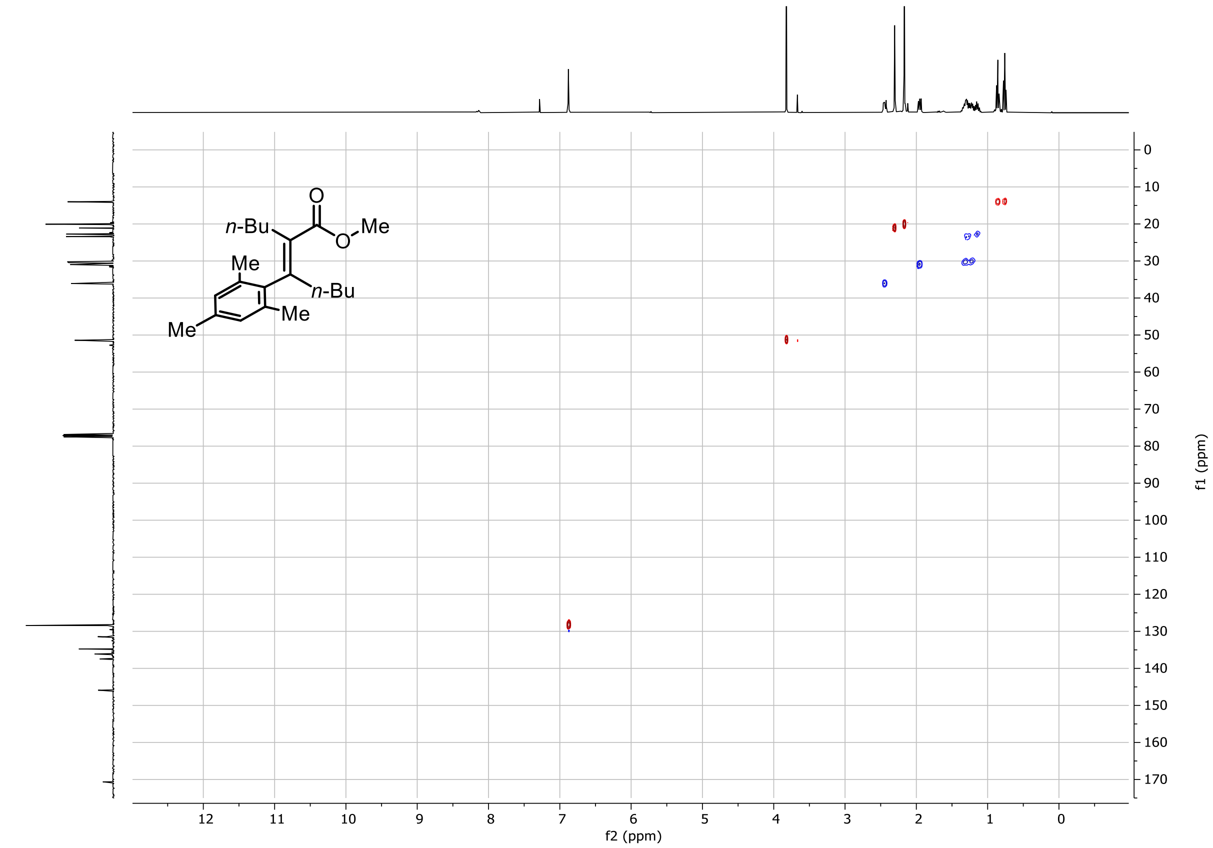
n-Bu
O
O
Me
Me
n-Bu
Me
Me
f2 (ppm)
f1 (ppm)

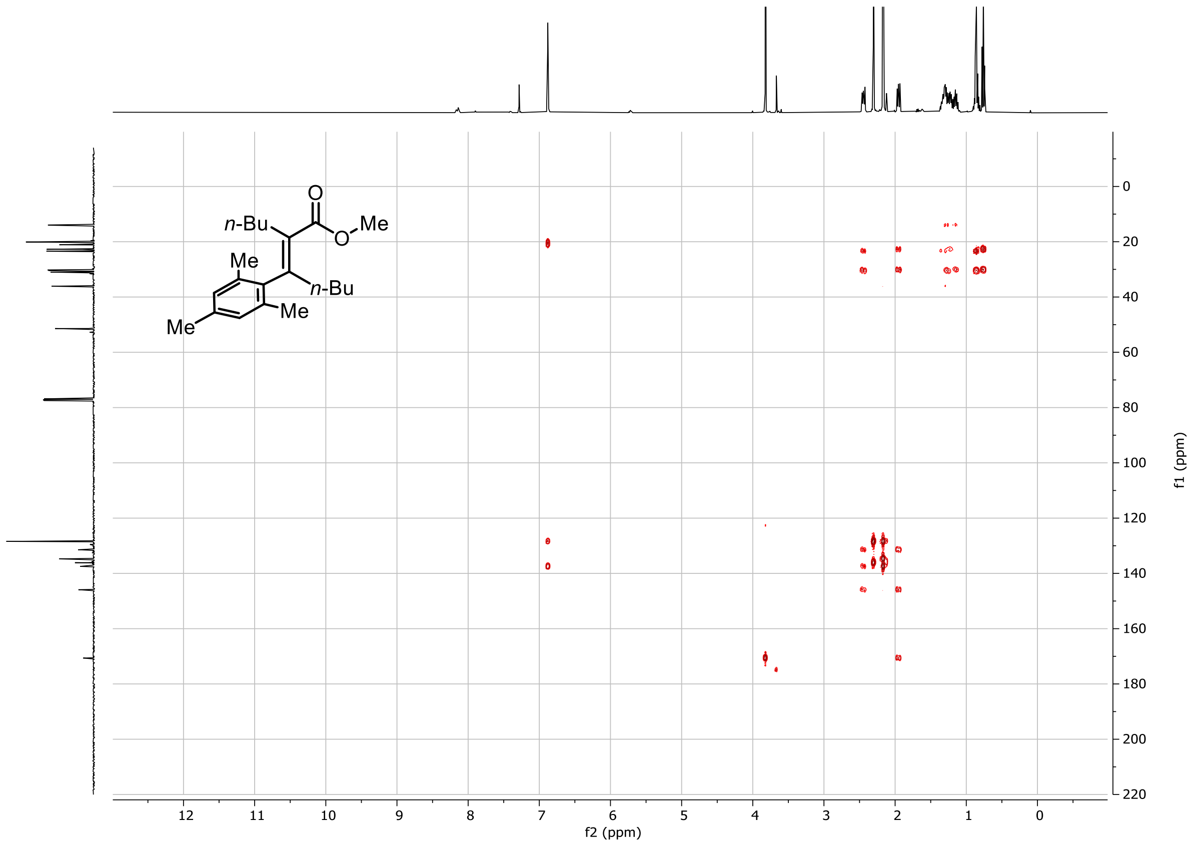

n-Bu
O
Me
Me
n-Bu
Me
Me
f1 (ppm)
f2 (ppm)

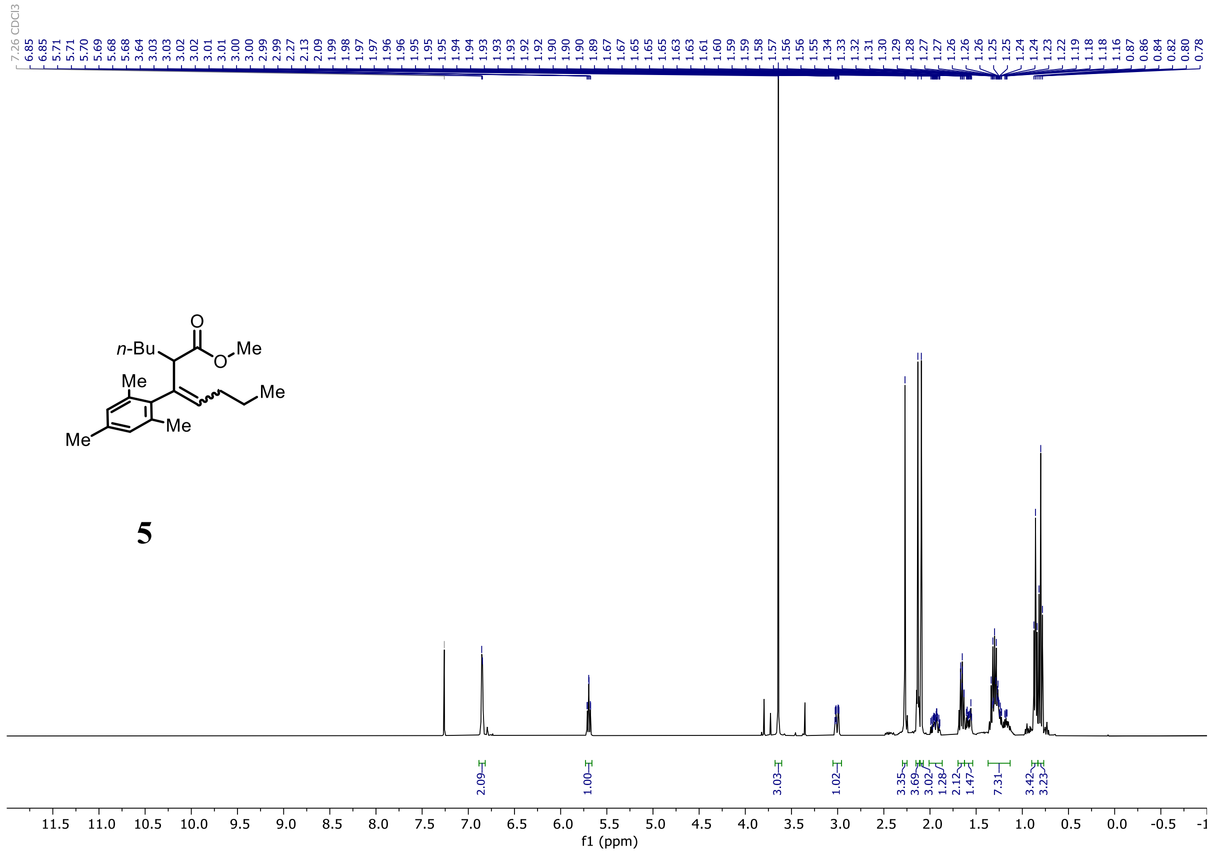

n-Bu
O
Me
Me
Me
Me
Me
5
f1 (ppm)

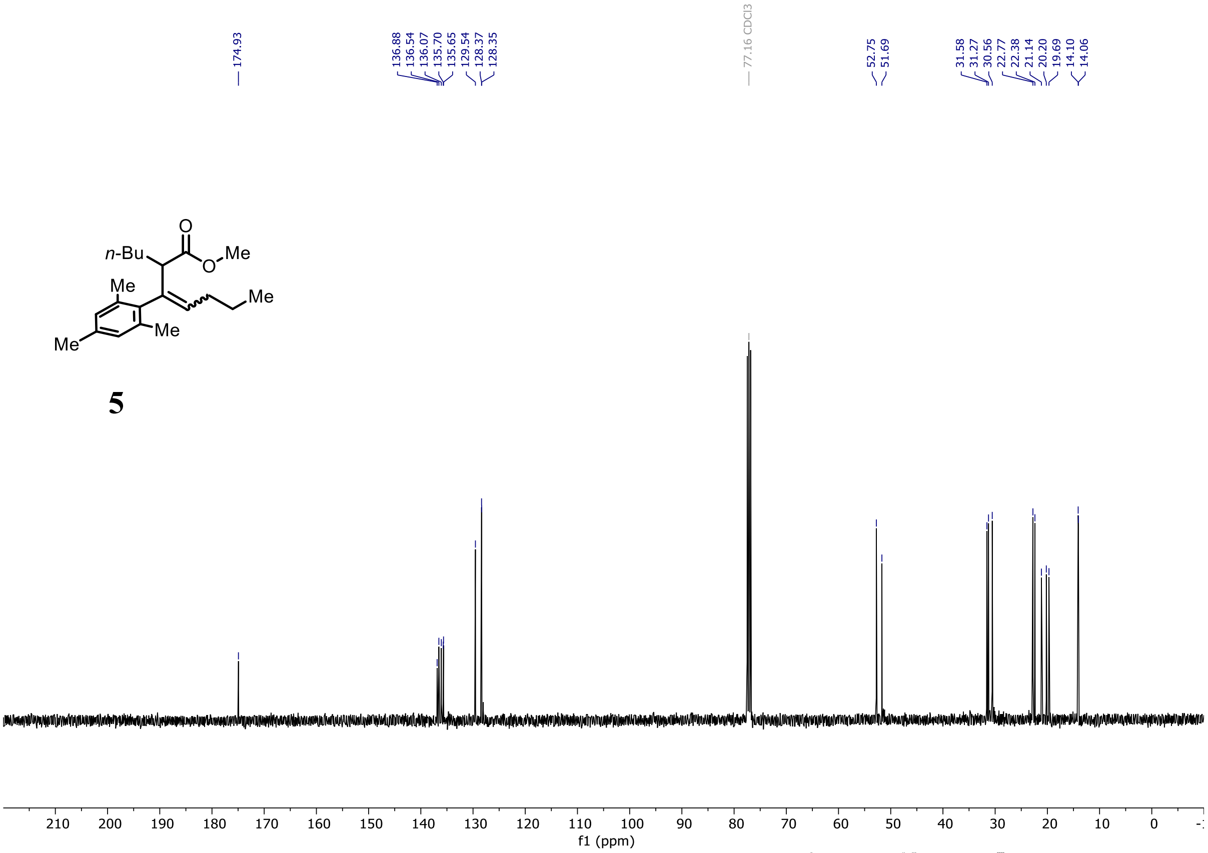

174.93
136.88
136.54
136.07
135.70
135.65
129.54
128.37
128.35
77.16 CDCl3
52.75
51.69
31.58
31.27
30.56
22.77
22.38
21.14
20.20
19.69
14.10
14.06
O
n-Bu
O
Me
Me
Me
Me
Me
210 200 190 180 170 160 150 140 130 120 110 100 90 80 70 60 50 40 30 20 10 0
f1 (ppm)

5

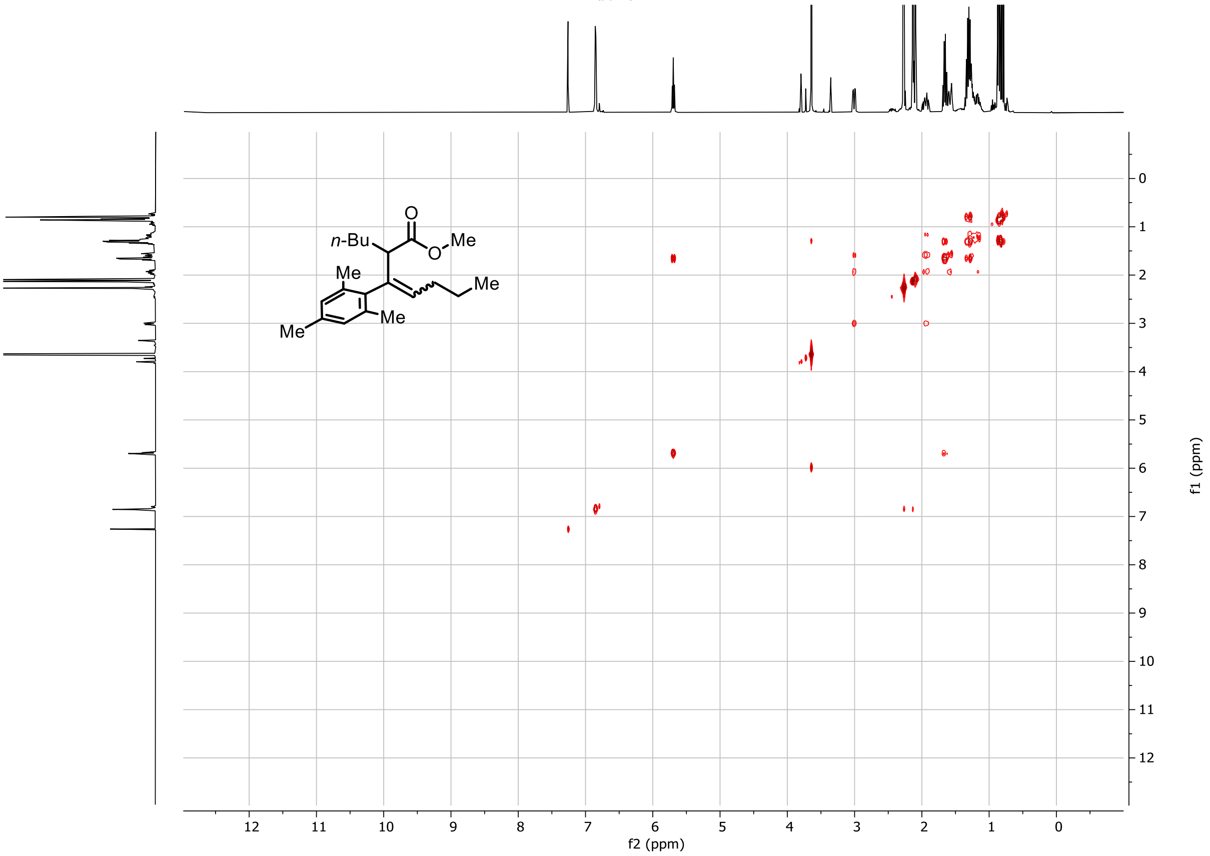

O
n-Bu
O
Me
Me
Me
Me
Me
12 11 10 9 8 7 6 5 4 3 2 1 0
f2 (ppm)
0 1 2 3 4 5 6 7 8 9 10 11 12
f1 (ppm)

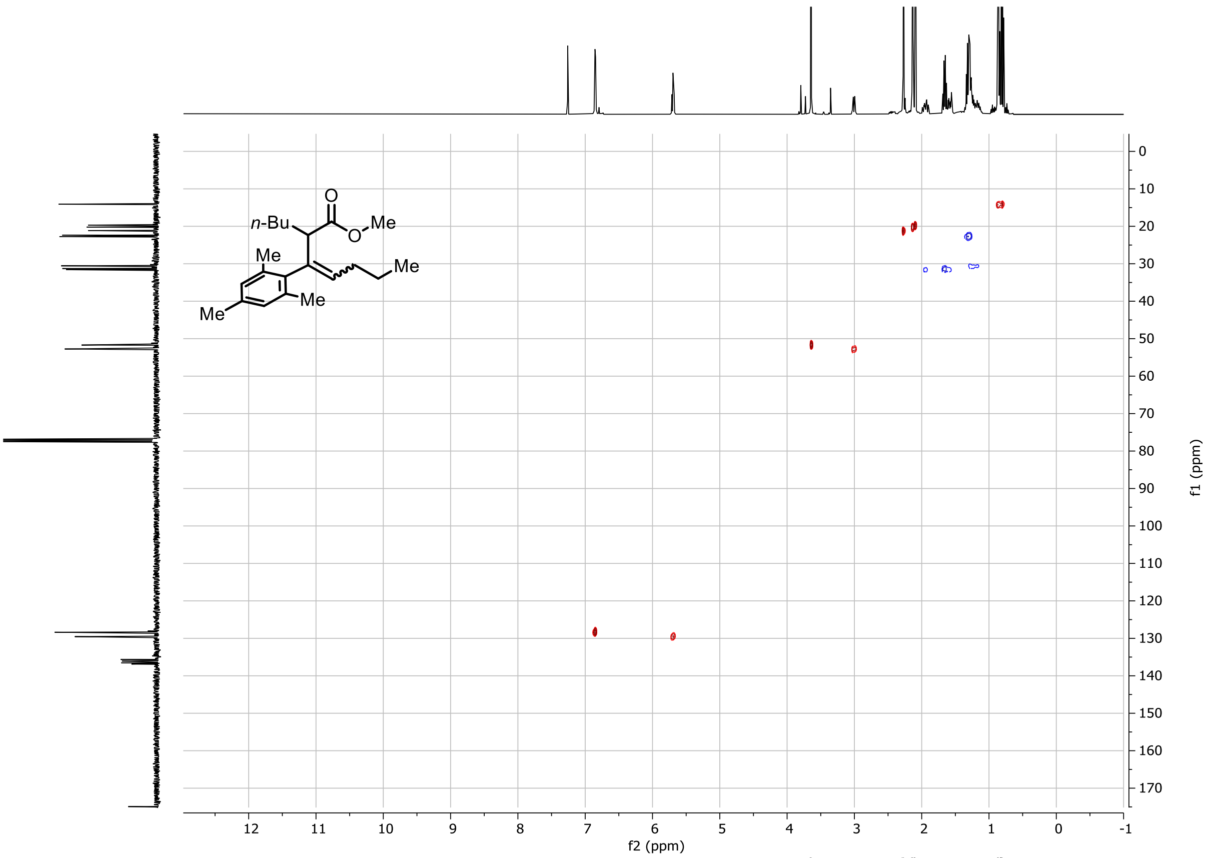
n-Bu
O
Me
Me
Me
Me
f2 (ppm)
f1 (ppm)

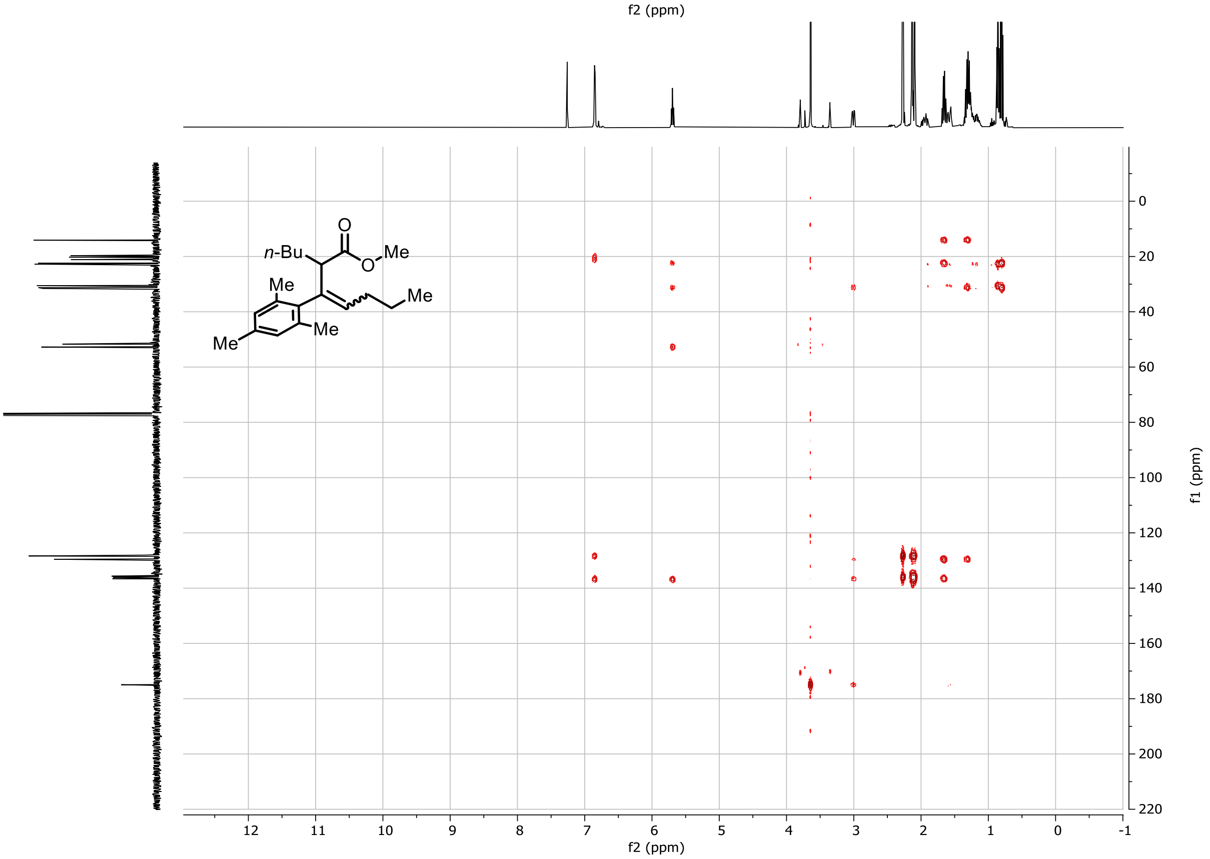
n-Bu
O
Me
Me
Me
Me
f2 (ppm)
f1 (ppm)

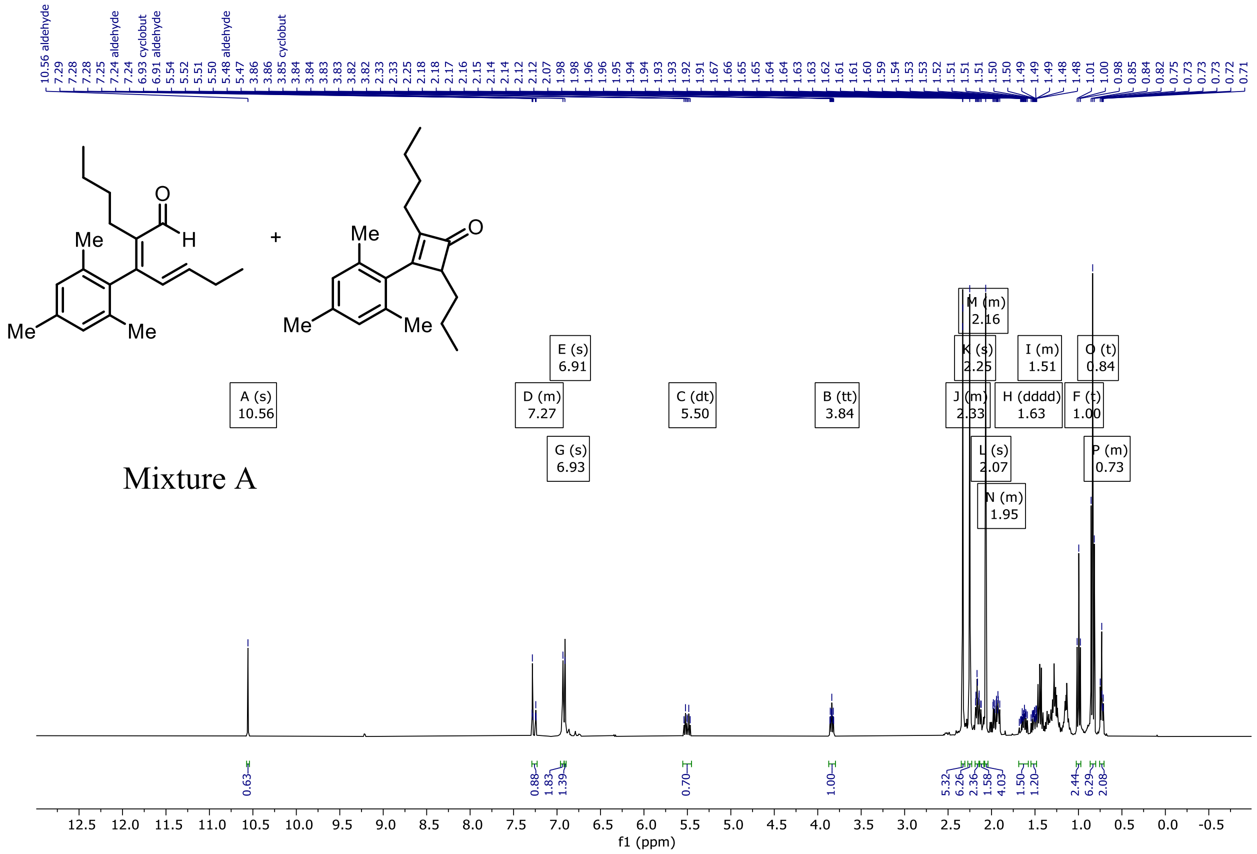
Me
O
H
+
Me
O
Me
Me
Me
Me
Mixture A
A (s)
10.56
B (tt)
3.84
C (dt)
5.50
D (m)
7.27
E (s)
6.91
F (t)
1.00
G (s)
6.93
H (dddd)
1.63
I (m)
1.51
J (m)
2.33
K (s)
2.25
L (s)
2.07
M (m)
2.16
N (m)
1.95
O (t)
0.84
P (m)
0.73
f1 (ppm)

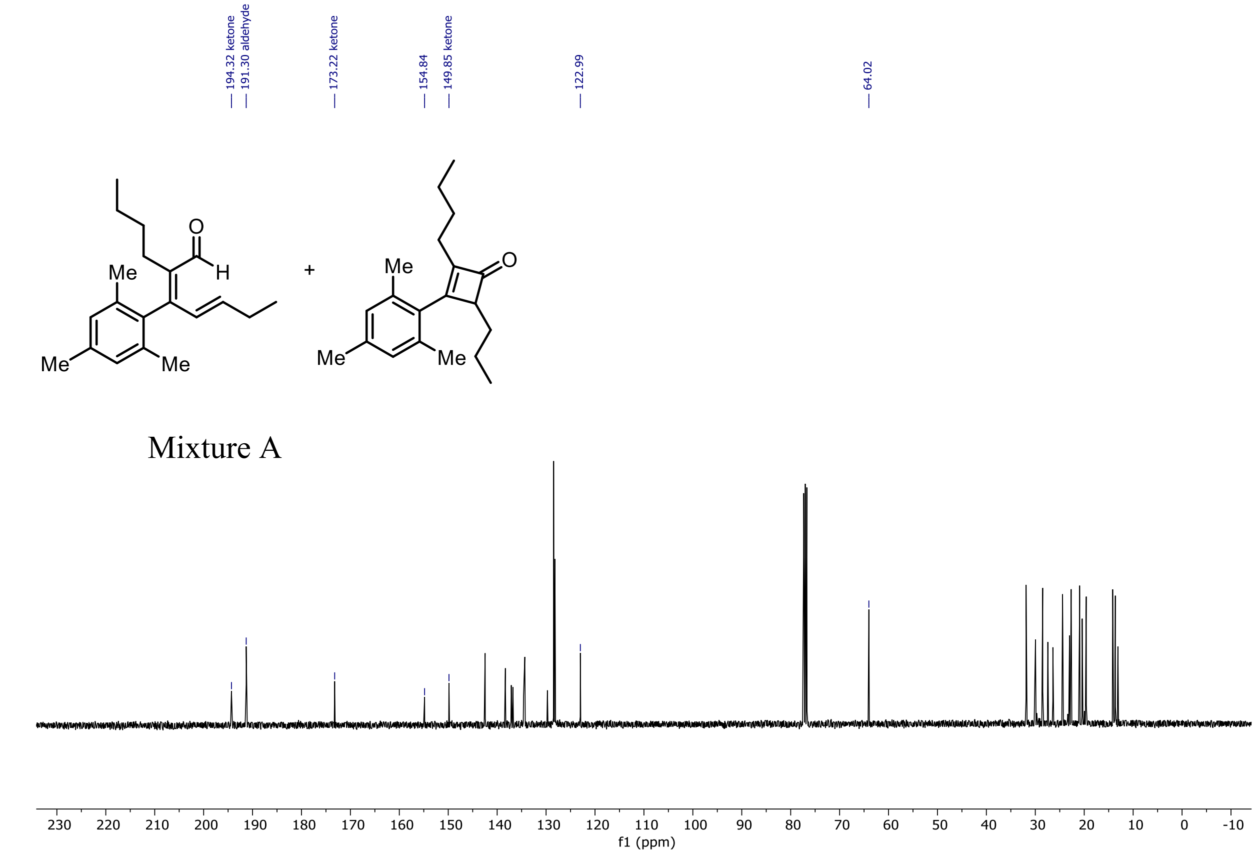
194.32 ketone
191.30 aldehyde
173.22 ketone
154.84
149.85 ketone
122.99
64.02
Mixture A
f1 (ppm)

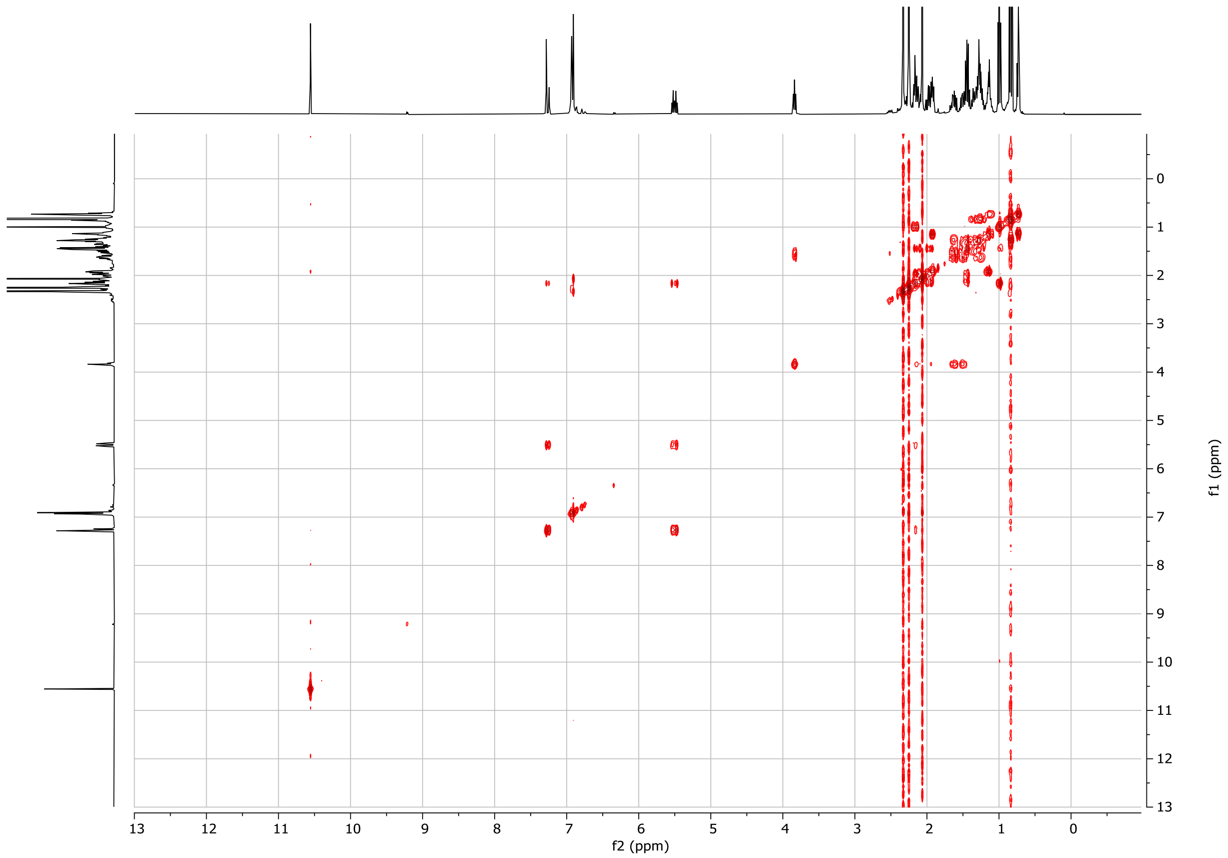
f2 (ppm)
f1 (ppm)

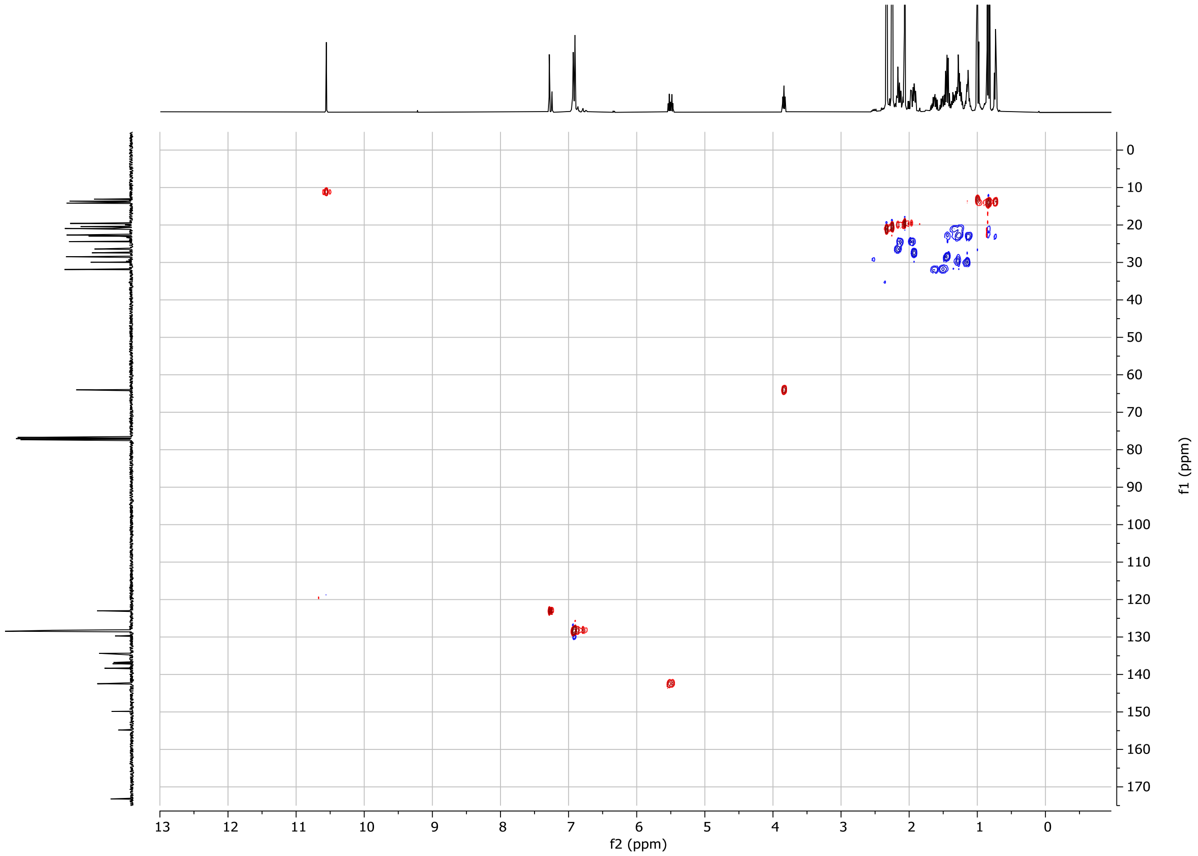
f2 (ppm)
f1 (ppm)

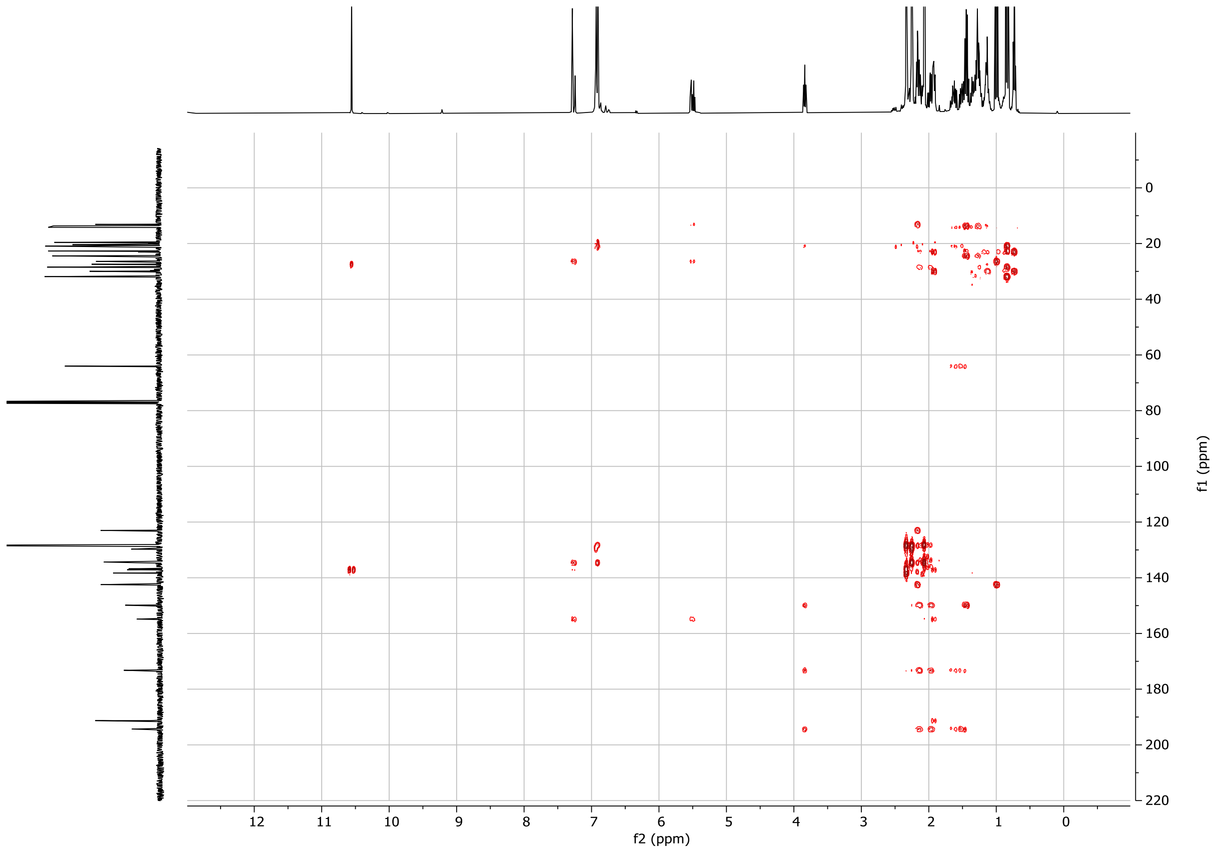

f1 (ppm)
f2 (ppm)

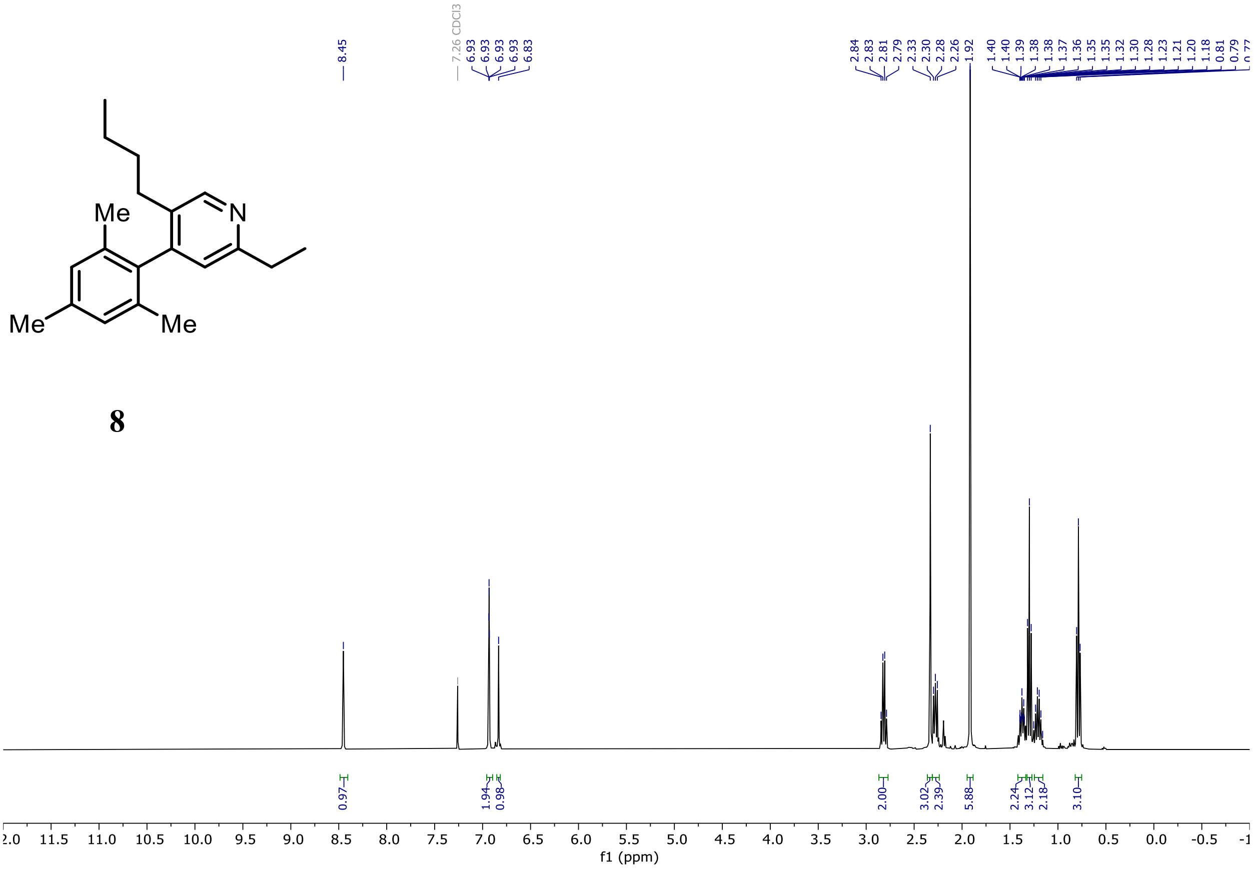

8.45
7.26 CDCl3
6.93
6.93
6.93
6.93
6.83
2.84
2.83
2.81
2.79
2.33
2.30
2.28
2.26
1.92
1.40
1.40
1.39
1.38
1.38
1.37
1.36
1.35
1.35
1.32
1.30
1.28
1.23
1.21
1.20
1.18
0.81
0.79
Me
N
Me
Me
8
0.97
1.94
0.98
2.00
3.02
2.39
5.88
2.24
3.12
2.18
3.10
f1 (ppm)

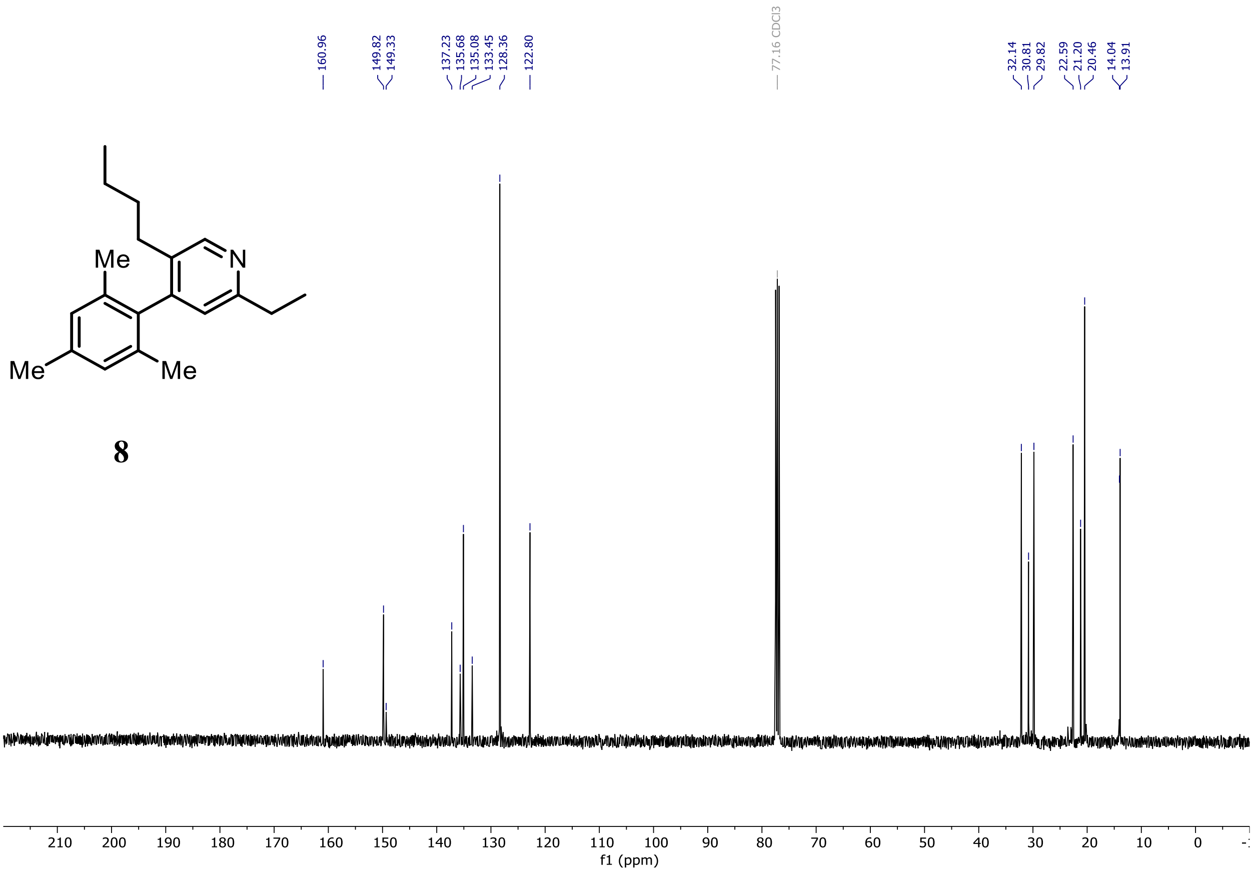

160.96
149.82
149.33
137.23
135.68
135.08
133.45
128.36
122.80
77.16 CDCl3
32.14
30.81
29.82
22.59
21.20
20.46
14.04
13.91
Me
N
Me
Me
8
f1 (ppm)
